# Isothiourea‐Catalyzed Acylative Kinetic Resolution of Tertiary α‐Hydroxy Esters

**DOI:** 10.1002/anie.202004354

**Published:** 2020-07-16

**Authors:** Shen Qu, Samuel M. Smith, Víctor Laina‐Martín, Rifahath M. Neyyappadath, Mark D. Greenhalgh, Andrew D. Smith

**Affiliations:** ^1^ EaStChem School of Chemistry University of St Andrews North Haugh St Andrews Fife KY16 9ST UK

**Keywords:** acyl transfer, enantioselectivity, kinetic resolution, organocatalysis, tertiary alcohols

## Abstract

A highly enantioselective isothiourea‐catalyzed acylative kinetic resolution (KR) of acyclic tertiary alcohols has been developed. Selectivity factors of up to 200 were achieved for the KR of tertiary alcohols bearing an adjacent ester substituent, with both reaction conversion and enantioselectivity found to be sensitive to the steric and electronic environment at the stereogenic tertiary carbinol centre. For more sterically congested alcohols, the use of a recently‐developed isoselenourea catalyst was optimal, with equivalent enantioselectivity but higher conversion achieved in comparison to the isothiourea HyperBTM. Diastereomeric acylation transition state models are proposed to rationalize the origins of enantiodiscrimination in this process. This KR procedure was also translated to a continuous‐flow process using a polymer‐supported variant of the catalyst.

## Graphical abstract

**Lewis base catalyzed** kinetic resolution of acyclic tertiary alcohols was achieved with selectivity factors of up to 200. Enantiodiscrimination is proposed to be reliant on the alcohol substrate containing a carbonyl recognition motif. The method, which tolerates aryl, heteroaryl, vinyl, and alkyl substituents, was also translated to continuous flow using a solid‐supported variant of the catalyst.

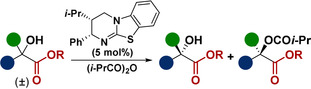

## Introduction

Tertiary alcohols and their derivatives are present within many natural products and bioactive molecules, however, their synthesis in enantiopure form remains a significant challenge.^1^ Towards this goal, the most commonly investigated method is the enantioselective addition of carbon‐centred nucleophiles to ketones.1a, 1b, 1c, 1d, 1e Challenging facial differentiation and the potential for unwanted side reactions currently impacts the scope and effectiveness of these methods. The catalytic kinetic resolution (KR)^2^, ^3^ of tertiary alcohols therefore represents a potentially attractive option. KRs are equally applicable to racemic and scalemic substrates, thus allowing KRs to be used as either alternative or complimentary processes. In contrast to the catalytic KR of secondary alcohols,^4^ there are currently very few efficient methods for the KR of tertiary alcohols. The challenges associated with the KR of tertiary alcohols are two‐fold: 1) tertiary alcohols are sterically hindered, thus reducing their nucleophilicity; and 2) the catalyst is required to differentiate between three non‐hydrogen substituents at the stereogenic carbinol centre.

**Figure 1:**
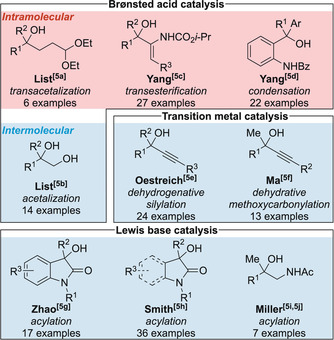
Approaches reported for the catalytic KR of tertiary alcohols.

To date, only nine methods have been reported for the non‐enzymatic catalytic KR of tertiary alcohols in which an enantioenriched chiral product is obtained (Figure 1).^5^, ^6^ Chiral phosphoric acid catalysis has been exploited by the List5a, 5b and Yang5c, 5d groups in intra‐ and intermolecular approaches for the KR of tertiary alcohols, amino alcohols, and diols; whilst the KR of tertiary propargylic alcohols has been reported by the Oestreich5e and Ma5f using Cu and co‐operative Pd/phosphoric acid catalysis, respectively. The acylative KR of alcohols is a particularly attractive option as simple separation of products, coupled with facile ester hydrolysis, provides straightforward access to both enantiomers of the alcohol. The Lewis base catalyzed acylative KR of heterocyclic tertiary alcohols has been achieved by Zhao and co‐workers5g and our group5h using oxidative NHC catalysis^7^ and isothiourea catalysis, respectively. To date, the only example of the catalytic acylative KR of acyclic alcohols was reported by Miller and co‐workers using a pentapeptide catalyst.5i, 5j Relatively high catalyst loading (10 mol %) and excess anhydride (50 equiv) was required for the KR of seven amino alcohol substrates, thus demonstrating the remaining challenge associated with the acylative KR of this important class of tertiary alcohol.

**Figure 2:**
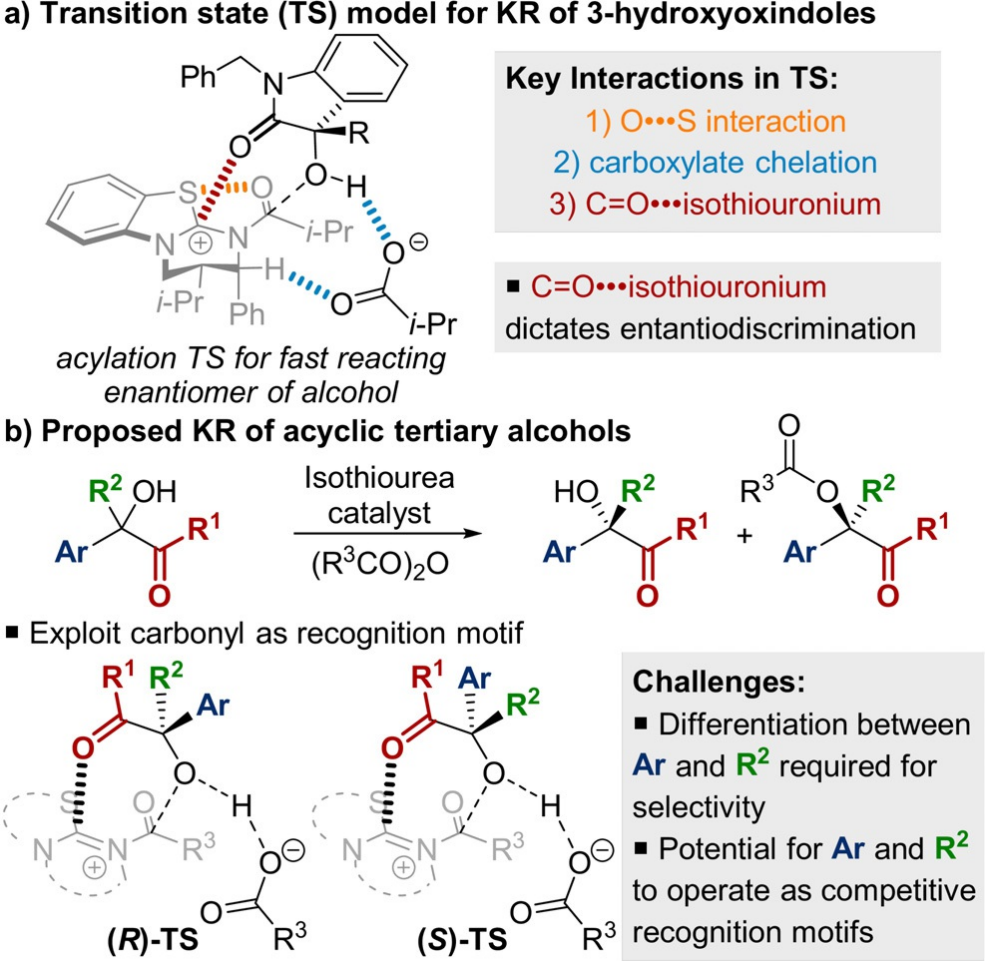
Proposed transition‐state models.

Lewis basic isothiourea catalysts^8^ have been applied for the acylative KR of a wide range of alcohols, including primary alcohols,^9^ secondary alcohols,^10^ and diols.^11^ We recently reported an isothiourea‐catalyzed KR of tertiary heterocyclic alcohols,5h in which coordinated experimental and computational studies were used to identify the origins of enantiodiscrimination (Figure 2 a). Interrogation of the acylation transition state structure for the fast‐reacting enantiomer of the alcohol revealed three key interactions: 1) an O⋅⋅⋅S interaction,^12^ which holds the acyl group of the acylated catalyst *syn*‐coplanar to the isothiouronium core; 2) chelation of the carboxylate counterion through non‐classical C−H⋅⋅⋅O hydrogen bonding;^13^ and 3) a C=O⋅⋅⋅isothiouronium interaction, which is primarily electrostatic in nature. The transition state structure for the slow‐reacting enantiomer lacked the C=O⋅⋅⋅isothiouronium interaction, and therefore it was hypothesized this interaction was critical for effective enantiodiscrimination.

In contrast to our previous work on the KR of heterocyclic alcohols,5h the additional conformational flexibility of acyclic substrates (Figure 2 b) presents additional challenges to overcome: 1) increased steric hindrance at the carbinol centre attenuating the rate of acylation; and 2) the potential for the other carbinol substituents to act as competitive recognition motifs, thus resulting in reduced enantiodiscrimination. We report herein the development of the acylative KR of acyclic tertiary alcohols using isothiourea catalysis. Key to this transformation is the incorporation of a suitable carbonyl donor adjacent to the tertiary stereogenic carbinol centre to act as a recognition motif for the acylated catalyst.

## Results and Discussion

Initial studies probed the feasibility of the acylative KR of acyclic α‐hydroxy carbonyl derivatives, using the isothiourea HyperBTM (**1**) as catalyst (Table 1). The attempted KR of tertiary amide **2** led to no conversion (entry 1), however the use of different secondary amide derivatives provided some promise.^14^ Following optimization the KR of secondary amide **3** was achieved with good conversion but moderate selectivity (*s*=7),^15^ which could not be improved upon further (entry 2). Next, the KR of α‐hydroxy ketones and esters was investigated (entries 3 and 4). Whilst low selectivity was obtained for both substrates (*s*=3), the KR of α‐hydroxy ester **5** was achieved using lower catalyst loading and with fewer equivalents of anhydride, thus indicating greater potential for further optimization through variation of solvent, temperature, base, and anhydride (see the Supporting Information for full details). Improved selectivity was obtained when the reaction was conducted in Et_2_O (*s*=15, entry 5), with additional optimization to *s*=60 achieved by using isobutyric anhydride as the acylating agent (entry 6). Finally, in the absence of an auxiliary base (NEt_3_), good conversion and excellent selectivity was maintained (entry 7). Under these optimized conditions, variation of the ester group was investigated, with the highest selectivity obtained using benzyl ester **8** (*c*=50 %, *s*=130, entry 10).^16^ The scalability of the method was demonstrated, with comparable conversion and selectivity obtained when conducting the KR on a gram scale (*c*=50 %, *s*=120, entry 11).

**Table 1:** Carbonyl group screening. 
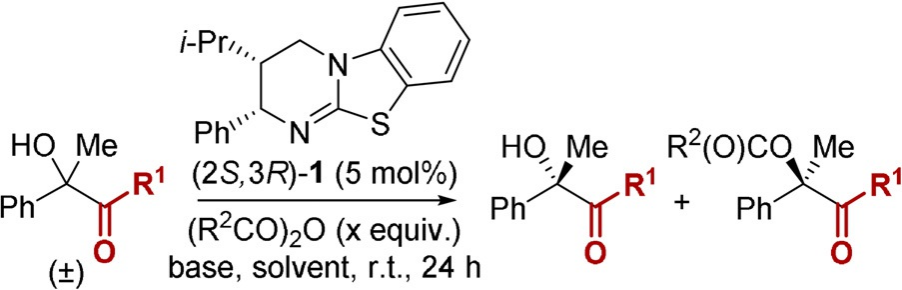

Entry	R^1^	R^2^ (*x*)	Base (equiv)	Solvent	c	*s*
1^[a,b]^	NMe_2_ (**2**)	Me (5)	TMP (10)	CHCl_3_	0	–
2^[a]^	NHPh (**3**)	*i*‐Pr (5)	TMP (2)	CH_2_Cl_2_	59	7
3^[a,c]^	Ph (**4**)	Me (3)	NEt_3_ (5)	CH_2_Cl_2_	36	3
4	OMe (**5**)	Me (1)	NEt_3_ (1)	CH_2_Cl_2_	44	3
5	OMe (**5**)	Me (1)	NEt_3_ (1)	Et_2_O	43	15
6	OMe (**5**)	*i*‐Pr (2)	NEt_3_ (3)	Et_2_O	47	60
7	OMe (**5**)	*i*‐Pr (2)	none	Et_2_O	41	70
8	OEt (**6**)	*i*‐Pr (2)	none	Et_2_O	32	60
9	O*t*‐Bu (**7**)	*i*‐Pr (2)	none	Et_2_O	15	7
10	OBn (**8**)	*i*‐Pr (2)	none	Et_2_O	50	130
11^[d]^	OBn (**8**)	*i*‐Pr (2)	none	Et_2_O	50	120

Conversion (c) and selectivity factor (*s*) calculated using the enantiomeric ratios of recovered alcohol and ester (see Ref. 3a). *s* values rounded according to estimated errors (see Ref. 3b). Reactions performed on 0.16–0.32 mmol scale, see SI for full details. TMP=2,2,6,6‐tetramethylpiperidine. [a] 10 mol % catalyst used. [b] Reaction at 50 °C. [c] Reaction at 40 °C. [d] 1.02 g (4 mmol) scale.

**Scheme 1:**
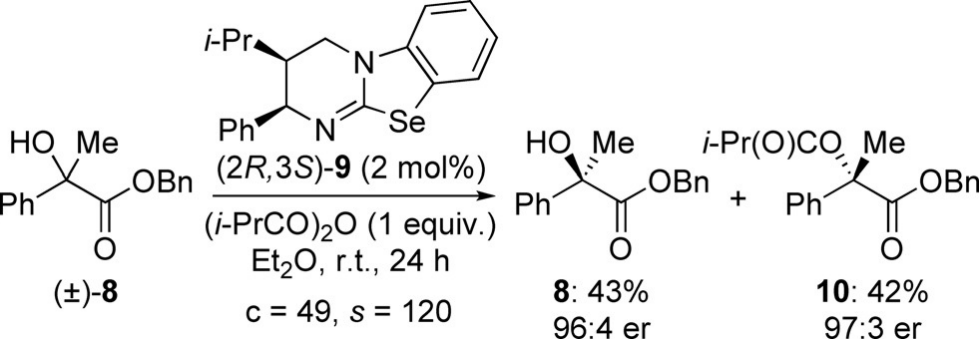
KR of (±)‐**8** using isoselenourea catalyst HyperSe (**9**).

We recently reported the isoselenourea HyperSe (**9**) as a highly efficient catalyst for a range of processes, including the KR of heterocyclic tertiary alcohols at catalyst loadings as low as 500 ppm.^17^ Applying this catalyst to the current KR procedure allowed reduction in both catalyst loading and equivalents of anhydride, whilst maintaining comparable conversion and selectivity (Scheme 1). Despite this improved activity, the reaction scope was initially investigated using the commercially available isothiourea, HyperBTM,^18^ with the isoselenourea HyperSe (**9**) reserved for the KR of particularly challenging substrates.

The scope and limitations of the newly developed KR process was first evaluated through the incorporation of electronically and sterically differentiated aryl substituents at the carbinol centre (Table 2). The KR of substrates **11**—**15**, which bear electron‐neutral and electron‐donating aromatic substituents (naphthyl, tolyl, anisolyl), was achieved with good conversion and high selectivity (*s*=60–140). The incorporation of a sterically demanding *ortho*‐anisolyl substituent, however, resulted in only 6 % conversion. This is consistent with our previous work on the KR of heterocyclic tertiary alcohols,5h where sterically encumbered substrates were less efficiently acylated. By applying the newly developed isoselenourea catalyst HyperSe (**9**) for the KR of **16**, significantly improved conversion (*c*=35 %) and good selectivity (*s*=20) was obtained. The KR of **17**, which bears an electron‐withdrawing aromatic substituent, was achieved with high conversion when using HyperBTM (**1**), but reduced selectivity obtained (*s*=12). Substrates bearing heterocycles were also well tolerated, with 2‐thienyl‐ and 2‐pyridyl‐substituted tertiary alcohols **18** and **19** resolved with good conversion and excellent selectivity (*s*=46–60).

**Table 2:** Substrate scope I: Aromatic‐substituent variation. 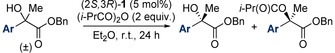

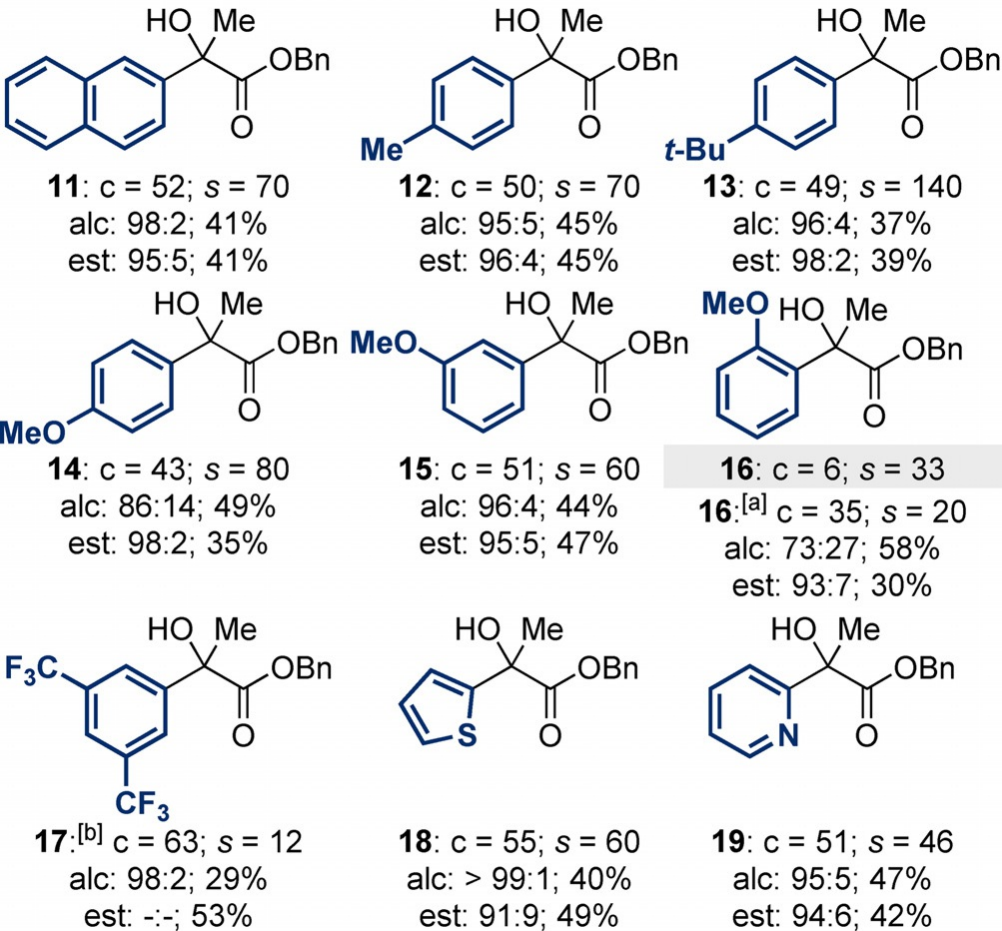

Conversion (c) and selectivity factor (*s*) calculated using the enantiomeric ratios of recovered alcohol and ester (see Ref. 3a). *s* values rounded according to estimated errors (see Ref. 3b). Reactions performed on 0.16–0.64 mmol scale; see the Supporting Information for full details. alc=alcahol, est=ester. [a] (2*R*,3*S*)‐HyperSe (**9**; 5 mol %) used; the alcohol and ester were obtained in the opposite enantiomeric series to that shown in the Scheme due to the (2*R*,3*S*) configuration of **9**. [b] (*i*‐PrCO)_2_O (1 equiv) used; separation of the ester enantiomers was not possible by HPLC, conversion based on ^1^H NMR spectroscopic analysis of crude reaction product mixture.

Next, the effect of varying the alkyl substituent at the carbinol centre was evaluated (Table 3). Replacing the methyl group with more sterically‐demanding substituents led to significantly lower conversion when using HyperBTM and (*i*‐PrCO)_2_O. For example, the KR of homoallylic alcohol **20** under the standard KR conditions provided only 4 % conversion. By replacing (*i*‐PrCO)_2_O with (MeCO)_2_O, good conversion and reasonable selectivity was obtained (*c*=42 %, *s*=9). The introduction of an ethyl or *n*‐butyl substituent at the carbinol centre also resulted in very low conversion (<2 %), however a combination of the isoselenourea HyperSe (**9**; 2 mol %) and (EtCO)_2_O allowed the KR of **21** and **22** with good conversion and selectivity (*c*=56–57 %, *s*=9–10). The KR of trifluoromethyl‐substituted tertiary alcohol **23** also benefitted from the use of HyperSe (**9**) to increase reaction conversion from 26 % to 48 %. The catalytic system was further challenged through the introduction of an additional π‐system at the carbinol centre to provide substrates with three potential recognition motifs. The KR of allylic tertiary alcohol **24** was achieved with good selectivity (*s*=20), with the use of the isoselenourea HyperSe (**9**) as catalyst again proving beneficial for increasing conversion. Finally, the KR of propargylic alcohol **25** was achieved with slightly reduced selectivity (*s*=6). Consistent with the lower steric hindrance of this substituent, good conversion was obtained when using HyperBTM (**1**).

**Table 3:** Substrate scope II: Alkyl‐substituent variation. 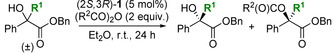

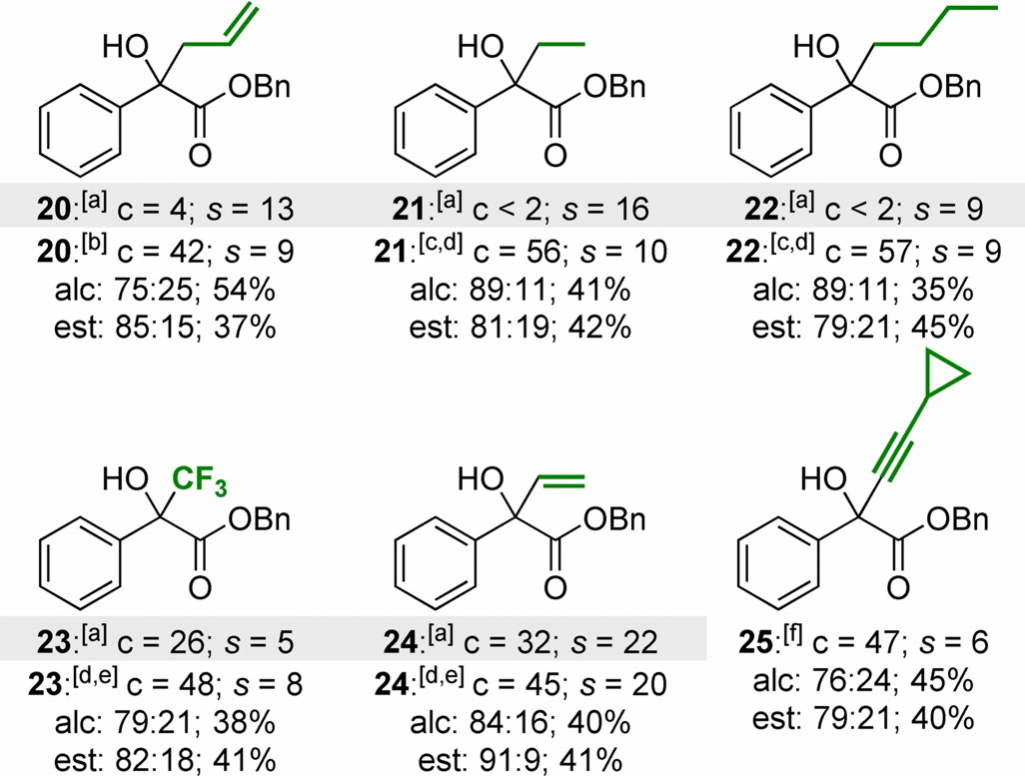

Conversion (c) and selectivity factor (*s*) calculated using the enantiomeric ratios of recovered alcohol and ester (see Ref. 3a). Reactions performed on 0.2–0.32 mmol scale; see the Supporting Information for full details. [a] (*i*‐PrCO)_2_O used. [b] (MeCO)_2_O used; [c] (2*R*,3*S*)‐HyperSe (**9**; 2 mol %), (EtCO)_2_O, and NEt_3_ (2 equiv) used. [d] The alcohol and ester were obtained in the opposite enantiomeric series to that shown in the Scheme due to the (2*R*,3*S*) configuration of **9**; [e] (2*R*,3*S*)‐**9** (2 mol %) and (*i*‐PrCO)_2_O used. [f] (*i*‐PrCO)_2_O (0.55 equiv) used.

Based on the lower selectivity values obtained for the KR of substrates bearing longer alkyl chains (Table 3), it was hypothesized that catalyst discrimination between the aryl and alkyl substituents may predominantly originate from steric differences.^19^ To investigate this hypothesis, the aryl substituent was replaced with a series of sterically‐differentiated groups (Table 4). As expected, the KR of alcohols **26** and **27**, which bear small alkynyl or vinyl substituents at the carbinol centre, was achieved with relatively low selectivity (*s*=2–7).^20^ Increasing the steric hindrance of the vinyl substituent through the introduction of two β‐methyl groups resulted in a small improvement in selectivity (*s*=10); however the introduction of an α‐methyl group had a significant effect, with alcohol **29** resolved with excellent selectivity (*s*>200). Based on these results, the KR of substrates bearing two sterically differentiated alkyl substituents at the carbinol centre was investigated. The introduction of a cyclopentyl or cyclohexyl group at this position resulted in only moderate conversion under the standard KR conditions (*c*≈20 %); however the use of HyperSe (**9**) enabled the KR of **30** and **31** with good conversion and selectivity (*c*=40–47 %, *s*=19–24). The importance of steric differentiation between the carbinol substituents was further supported by the attempted KR of an electronically differentiated di‐aryl‐substituted alcohol, which resulted in essentially no selectivity.^14^

**Table 4:** Substrate scope III: Further structural variation. 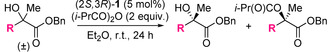

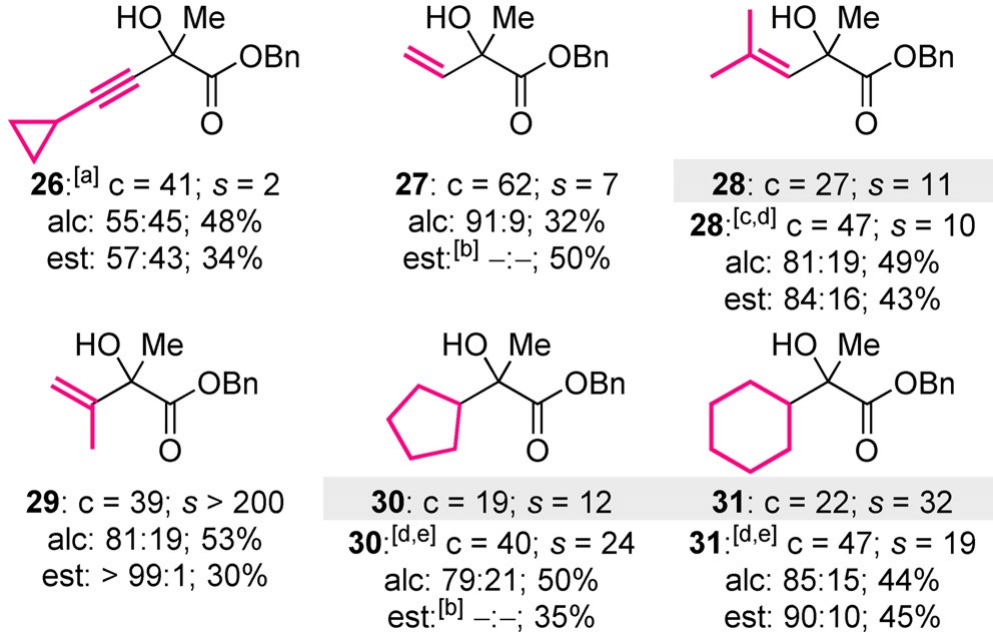

Conversion (c) and selectivity factor (*s*) calculated using the enantiomeric ratios of recovered alcohol and ester (see Ref. 3a). *s* values rounded according to estimated errors (see Ref. 3b). Reactions performed on 0.13–0.32 mmol scale; see the Supporting Information for full details. [a] (*i*‐PrCO)_2_O (0.55 equiv) used. [b] Separation of enantiomers not possible by HPLC analysis, conversion based on ^1^H NMR spectroscopic analysis of crude reaction product mixture. [c] (2*R*,3*S*)‐HyperSe (**9**; 2 mol %) used. [d] Alcohol and ester obtained in the opposite enantiomeric series to that shown in the scheme. [e] (2*R*,3*S*)‐**9** (5 mol %) used.

A common perceived drawback of organocatalysis is the use of relatively high loadings of the catalyst, which is typically discarded following a given reaction. One potentially general solution is immobilization of the organocatalyst on a heterogeneous support, provided that the catalyst maintains activity and displays high stability.^21^, ^22^ We recently addressed this issue through the development of a polymer‐supported isothiourea catalyst (**32**), which could be applied for the KR of alcohols in batch and flow with no reduction in either activity or selectivity observed upon recycling.^23^ Application of this continuous‐flow technology to the KR of acyclic tertiary alcohols was therefore targeted (Table 5). Since Merrifield‐resin‐supported catalyst **32** does not swell in Et_2_O, process optimization focused on the application of alternative solvents.^14^ The use of toluene proved to be optimal, with excellent conversion and selectivity obtained for the KR of **8** (*c*=50 %, *s*=50). A further four structurally diverse substrates were applied under the optimal conditions. Variation of the aryl substituent was well tolerated, with **13** and **15** resolved with good conversion and good to excellent selectivity (*c*≈50 %, *s*=29–80). The KR of allylic alcohols **24** and **29** was also successful. Although slightly lower conversion was observed under the standard continuous‐flow conditions, good to excellent selectivity was obtained in each case (*s*=21–60). To the best of our knowledge, this work represents the first example of the KR of acyclic tertiary alcohols in a continuous‐flow process.

**Table 5:** KR in continuous flow. 
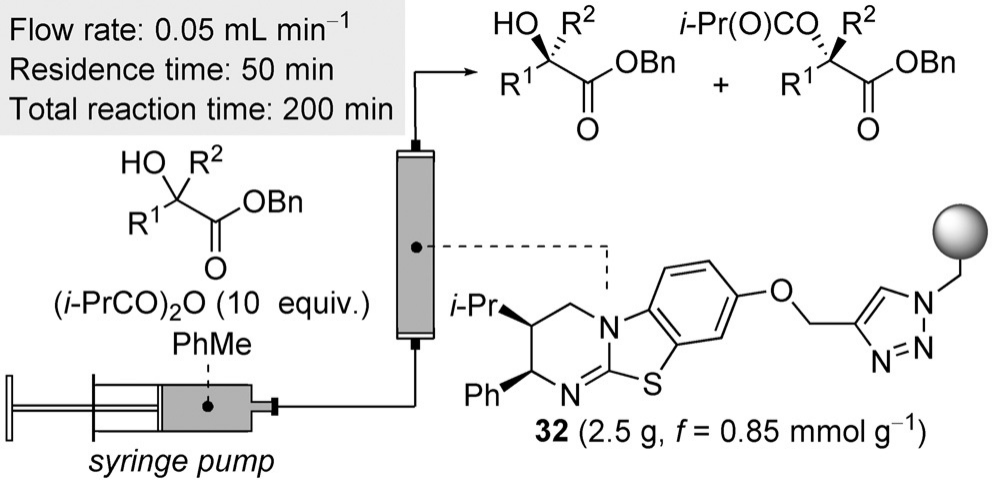

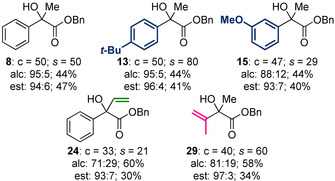

Conversion (c) and selectivity factor (*s*) calculated using the enantiomeric ratios of recovered alcohol and ester (see Ref. 3a). *s* values rounded according to estimated errors (see Ref. 3b). Reactions performed on 0.2–0.22 mmol scale; see the Supporting Information for full details.

**Figure 3:**
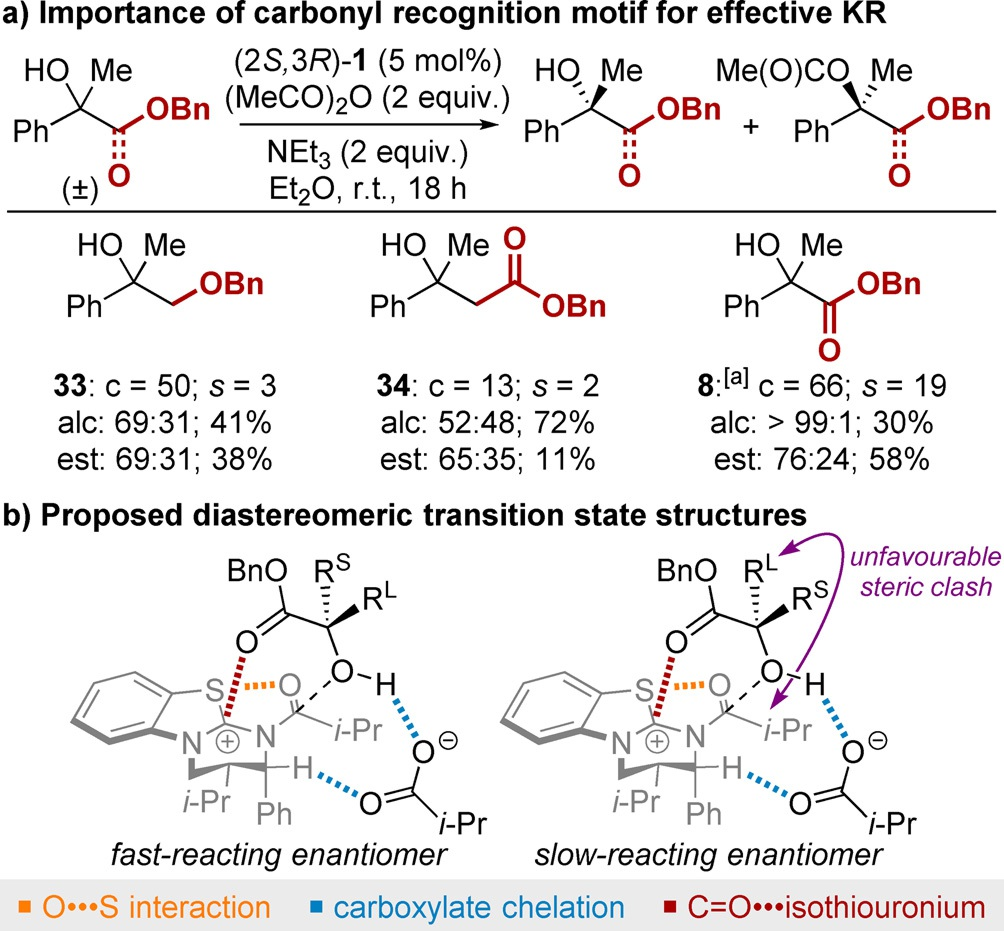
Experimental insights and proposed origin of enantiodiscrimination, where R^L^ is sterically larger than R^S^. [a] (MeCO)_2_O (1 equiv) used.

Finally, the importance of the carbonyl recognition motif was investigated to provide insight into the origins of enantiodiscrimination in this KR process (Figure 2). Attempted KR of benzyl ether **33** or homologated benzyl ester **34** resulted in essentially no conversion under the standard KR conditions. The resolution of these substrates could be achieved by switching to (MeCO)_2_O as the acyl donor, however low selectivity was obtained (*s*<3) (Figure 3 a). In contrast, the KR of ester **8** under analogous conditions was achieved with *s*=19. This demonstrates that the presence and proximity of the ester functionality is essential to promote acylation and allow effective enantiodiscrimination. The absolute configuration of the recovered alcohol within each substrate class [aryl/alkyl (**5**,**20**); alkenyl/alkyl (**29**); alkyl/alkyl (**31**)] was determined by comparison of specific rotations to reported values.^14^ Based on these data and previous computational studies,5h, 10h, 10q we propose that the ester functionality operates as a recognition motif within the acylation transition states of this KR by engaging in a stabilizing C=O⋅⋅⋅isothiouronium interaction with the acylated catalyst (Figure 3 b). The preferential acylation of the fast‐reacting enantiomer for each substrate class can then be rationalized through minimization of unfavourable steric contacts between the substrate and the acyl group of the acylated catalyst. This model helps explain why substrates bearing alkyl substituents larger than methyl at the carbinol centre were challenging to resolve and required the use of less sterically hindered anhydrides as the acyl donor.^24^

## Conclusion

In conclusion, a highly enantioselective isothiourea‐catalyzed acylative kinetic resolution (KR) of acyclic tertiary alcohols has been developed. Through utilizing an adjacent carbonyl substituent as a recognition motif for the acylated catalyst, the KR of 25 α‐hydroxy ester derivatives was achieved with selectivity factors of up to 200. Increased steric hindrance at the tertiary carbinol centre resulted in low conversion; however this issue was circumvented by performing the KR of these substrates by using the recently developed isoselenourea catalyst HyperSe (**9**). This new KR procedure was also applied in continuous flow using a polymer‐supported isothiourea catalyst to resolve acyclic tertiary alcohols with good to excellent selectivity. Based on mechanistic control reaction, and previous computational studies, it is proposed that stabilization and enantiodiscrimination within the acylation transition‐state structure originates through maximization of a C=O⋅⋅⋅isothiouronium interaction between the α‐hydroxy ester substrate and acylated catalyst. Although not demonstrated in this manuscript, the known derivatization^25^ of structurally related products with conservation of er should allow access to further tertiary‐alcohol‐containing motifs using this method.^26^

## Conflict of interest

The authors declare no conflict of interest.

## Supporting information

As a service to our authors and readers, this journal provides supporting information supplied by the authors. Such materials are peer reviewed and may be re‐organized for online delivery, but are not copy‐edited or typeset. Technical support issues arising from supporting information (other than missing files) should be addressed to the authors.

Supplementary
