# Supplementary material for: Isothiourea‐Catalyzed Acylative Kinetic Resolution of Tertiary α‐Hydroxy Esters
Source: Angew Chem Int Ed Engl. 2020 Jul 16;59(38):16572–8. doi: 10.1002/anie.202004354 (PMC7540711; doi:10.1002/anie.202004354)
Supplement: Supplementary file 1 — Supplementary [file ANIE-59-16572-s001.pdf]

Angewandte Chemie
Eine Zeitschrift der Gesellschaft Deutscher Chemiker

Supporting Information

Isothiourea-Catalyzed Acylative Kinetic Resolution of Tertiary α-Hydroxy Esters

*Shen Qu[+], Samuel M. Smith[+], Víctor Laina-Martín, Rifahath M. Neyyappadath, Mark D. Greenhalgh, and Andrew D. Smith**

anie_202004354_sm_miscellaneous_information.pdf

Supporting Information

Table of Contents

General Experimental S2

General Procedures S3

Preparation of Acyclic Tertiary Alcohols [Data: mp, NMR, IR, HRMS] S5

Preparation of Racemic Esters [Data: mp, NMR, IR, HRMS] S27

Catalytic Kinetic Resolution: Optimisation S47

Catalytic Kinetic Resolution: Scope [Data for enantioenriched compounds ($[\alpha]_D^{20}$, Chiral HPLC, HPLC traces)] S52

Catalytic Kinetic Resolution in Continuous Flow: Optimisation S100

Catalytic Kinetic Resolution in Continuous Flow: Scope [Data for enantioenriched compounds (Chiral HPLC, HPLC traces)] S102

NMR Traces S107

References S263

General Experimental

Moisture sensitive reactions were carried out under a nitrogen (N_2) atmosphere using standard vacuum line techniques and anhydrous solvents. All glassware used was flame-dried and allowed to cool to room temperature under vacuum before use. Anhydrous solvents (THF and Et_2O) were obtained after passing through an alumina column (Mbraun SPS-800). All other solvents and commercial reagents were used as supplied without further purification.

Room temperature (r.t.) refers to 20-25 °C. Temperatures of 0 °C were obtained using ice/water bath while −25 °C obtained using NaCl/ice baths. *In vacuo* refers to the use of a Büchi Rotavapor R-2000 or a Heidolph Laborota 4001 rotary evaporator with a vacuum controller.

Analytical thin layer chromatography was performed on pre-coated aluminium plates (Kieselgel 60 F254 silica). TLC visualisation was carried out with ultraviolet light (254 nm), followed by staining with a 1% aqueous $KMnO_4$ solution. Automated chromatography was performed on a Biotage Isolera Four running Biotage OS578 with a UV/Vis detector using the method stated and cartridges filled with Kieselgel 60 silica.

Melting points were recorded on an Electrothermal 9100 melting point apparatus and are uncorrected. Infrared spectra (ν_{max} /cm^{-1}) were recorded on a Shimadzu IRAffinity-1 using a Pike attenuated total reflectance (ATR) accessory. Only the characteristic peaks are quoted.

^{1}H, ^{13}C {^{1}H} and ^{19}F {^{1}H} nuclear magnetic resonance (NMR) spectra were acquired on a Bruker Avance II 400 (500 MHz, ^{1}H, 127 MHz ^{13}C) spectrometer at r.t. in $CDCl_3$ (solvent reference peak for $CDCl_3$ in the ^{1}H NMR = 7.26 ppm, and in ^{13}C{^{1}H} NMR = 77.16). All chemical shifts are quoted in parts per million (ppm) relative to the residual solvent as the internal standard. All coupling constants, *J*, are quoted in Hertz (Hz). Multiplicities are indicated by: s (singlet), d (doublet), t (triplet), dd (doublet of doublets), ddd (doublet of doublet of doublets), ddt (doublet of doublet of triplets), dq (doublet of quadruplets), tt (triplet of triplets), hept (heptet) and m (multiplet). The abbreviation Ar is used to denote aromatic, br is used to denote broad peaks. NMR peak assignments were confirmed using 2D ^{1}H correlated spectroscopy (COSY), 2D ^{1}H–^{13}C heteronuclear multiple-bond correlation spectroscopy (HMBC), and 2D ^{1}H–^{13}C heteronuclear single quantum coherence spectroscopy (HSQC) where necessary.

HPLC traces of enantiomerically enriched compounds were compared with authentic racemic spectra. HPLC analyses were obtained operated on a Shimadzu HPLC instrument consisting of a DGU20A5 degasser, LC-20AT liquid chromatograph, SIL-20AHT autosampler, CMB-20A communications bus module, SPD-M20A diode array detector and a CTO-20A column oven which allowed the temperature to be set from 25-40 °C. Separation was achieved using either a DAICEL CHIRALCEL OD-H and OJ-H column or DAICEL CHIRALPAK AD-H, AS-H and IB columns using the method stated.

Mass spectrometry (m/z) data were acquired by atmospheric solids analysis probe (ASAP) or nanospray ionisation (NSI) at the EPSRC UK National Mass Spectrometry Facility at Swansea University. Masses are reported to 4 dp, with ppm errors calculated using unrounded values.

Optical rotations were measured on a Perkin Elmer Precisely/Model-341 polarimeter operating at the sodium D line with a 100 mm path cell at r.t. in $CHCl_3$.

General Procedures

General procedure A: *Esterification of acids via an intermediate acid chloride*

Following the method by Zhao *et al*,[1] to a solution of acid (1 equiv.) in anhydrous CH_2Cl_2 (25 mL), was added oxalyl chloride (1.2 equiv.) and a drop of DMF. The mixture was stirred for 2 h at r.t. before the solvent and redundant oxalyl chloride were removed under reduced pressure. After which, the relevant alcohol (2 equiv.) and pyridine (1 equiv.) were added to the resulting α-acyl chloride in CH_2Cl_2 at 0 °C and stirred for 1 h. Upon completion, the mixture was poured into water (20 mL) and extracted with CH_2Cl_2 (3 × 20 mL). The organic phases were combined, dried over $MgSO_4$ filtered and concentrated *in vacuo*. The ester products were purified by column chromatography.

General procedure B: *Preparation of α-hydroxy esters through α-oxidation*

Following the method by Liang *et al*,[2] the desired ester (1 equiv.), triethyl phosphite (2 equiv.) and cesium carbonate (20 mol%) were mixed under a O_2 atmosphere. DMSO [0.25 M] was added and mixture stirred under O_2 (1 atm) at r.t. for 24-72 h. On completion, the mixture was diluted with Et_2O and washed with brine (2 × 10 mL), extracted with Et_2O (3 × 20 mL), dried over Na_2SO_4, filtered and concentrated *in vacuo*. The α-hydroxy ester products were purified by column chromatography.

General procedure C: *Preparation of Grignard reagent*

Magnesium turnings (3 equiv) were added to anhydrous THF in an oven-dried multi-necked round-bottomed flask under a N_2 atmosphere. The desired bromide (2 equiv) was dissolved in anhydrous THF and 10% of this solution was added to the magnesium. The reaction was stirred at r.t. until the reaction temperature increased. If the reaction temperature did not increase, a small iodine crystal was added and stirred until the brown colour disappeared and temperature increased. The remaining bromide solution was added over 15 minutes and stirred for a further 2 h, then left cool and settle. The concentration of the prepared Grignard reagent was determined by titration using 2-hydroxybenzaldehyde phenylhydrazone.

General procedure D: *Synthesis of tertiary alcohols through Grignard addition*

A solution of α-hydroxy ester or α-hydroxy acid (1 equiv) was dissolved in anhydrous THF under a N_2 atmosphere and cooled to –78 °C. A Grignard reagent (1-2 equiv) was added dropwise and the solution stirred at –78 °C for 20 mins, then at 0 °C for 30 mins. Further Grignard reagent (0.2 equiv) was added at 0 °C and reaction monitored by TLC until completion. The reaction mixture reaction was poured into aqueous NH_4Cl solution (20 mL) and extracted with EtOAc (3 × 20 mL). The organic layers were combined, dried over $MgSO_4$, filtered and concentrated *in vacuo*. The product was purified *via* column chromatography.

General procedure E: *Synthesis of tertiary alcohol through CDI coupling*

Racemic α-hydroxy acid (1 equiv) and CDI (1.1 equiv) were stirred in CH_2Cl_2 for 10 mins. Amine or alcohol (1.1 equiv) was added slowly and the resulted solution was stirred 18 h at r.t. The reaction was quenched by H_2O, aqueous phase was extracted with CH_2Cl_2, washed with sat. $NaHCO_3$ and H_2O. The combined organic phase was dried over $MgSO_4$, filtered and concentrated *in vacuo*. The product was purified *via* column chromatography.

General procedure F*: Esterification of alcohol with anhydrides and DMAP*

An anhydride (4 equiv) and DMAP (10 mol%) were added to a solution of alcohol (1 equiv) in CH_2Cl_2. Base (6 equiv) was added and the reaction mixture was stirred at r.t. for 24 h. On completion, the mixture was diluted with EtOAc and washed sequentially with HCl (2 x10 mL) and sat. aq. $NaHCO_3$ (2 × 10 mL), dried over $MgSO_4$, filtered and concentrated *in vacuo*. The ester products were purified by column chromatography and analysed by chiral HPLC.

General procedure G*: Preparation of α-substituted arylacetic acids*

Following a modified method outlined by Rao *et al*,[3] *n*-BuLi (2.2 equiv) was added to a solution of *i*-Pr_2NH (2.2 equiv) in anhydrous THF in a flame-dried round-bottomed flask under a N_2 atmosphere at 0 °C. The LDA solution was stirred for 30 minutes, the arylacetic acid (1.0 equiv) was added and the reaction mixture stirred for 1 h at 0 °C. The specified alkyl halide (2.2 equiv) was added and the reaction stirred overnight at r.t.. HCl (20 mL) was added until pH 1 was reached. The aqueous layer was extracted with EtOAc (3 × 20 mL). The organic layers were combined, dried over Na_2SO_4, filtered and concentrated *in vacuo* to give the crude α-substituted aryl acetic acid.

General procedure H*: Kinetic resolution of tertiary alcohols with anhydrides and isothioureas*

An isothiourea catalyst (1-10 mol%) was added to a solution of alcohol (1 equiv) in the required solvent. The reaction was cooled to the required temperature and anhydride (0.6-2.0 equiv) and base (0-4.0 equiv) were added. The reaction mixture was stirred for the required time. On completion, the mixture was diluted with EtOAc (20 mL) and washed with HCl (2 × 10 mL) and sat. aq. $NaHCO_3$ (2 × 10 mL), dried over $MgSO_4$, filtered and concentrated *in vacuo*. The alcohol and ester were purified by column chromatography and analysed by chiral HPLC.

General procedure I*: Kinetic resolution of tertiary alcohols in continuous flow*

A packed bed reactor consisting of a vertically-mounted Omnifit glass chromatography column (10 mm pore size and up to maximal 70 mm of adjustable bed height), with a glass cooling jacket was loaded with PS-HyperBTM resin (1.2 g; *f* = 0.85 mmol g^{-1}). The resin was allowed to swell to its maximum volume by pumping solvent at 0.5 mL min^{-1} for 30 min at r.t. using a P 4.1S Azura pump developed by Knauer. The flow rate was then changed to the specified reaction flow rate for 30 min. A Legato 200 series syringe pump by KR Scientific was used to inject samples through syringes. The syringe was filled with a mixture of the appropriate alcohol (1 equiv.) and isobutyric anhydride in specified solvent. The solution was injected at specified flow rate. After addition of reagents is completed, a P 4.1S Azura pump developed by Knauer was connected and pumped solvent at specified flow rate for 120 min to ensure elution of the products and achieve regeneration of the column for the next reaction. The collected reaction mixture was concentrated *in vacuo*. The alcohol and ester were purified by column chromatography and analysed by chiral HPLC.

Preparation of Acyclic Tertiary Alcohols

2-Hydroxy-2-phenylpropanoic acid S1

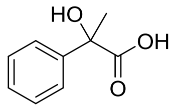

To a solution of phenylglyoxylic acid (1.5 g, 10 mmol) in anhydrous Et_2O (25 mL) was added MeMgBr (7.3 mL, 3 M, 22 mmol) dropwise at −25 °C. After stirring for 24 h, the reaction was acidified with HCl (1 M) to pH 4 and extracted with EtOAc. The organic phases were combined, washed with H_2O and brine, dried over $MgSO_4$, filtered and concentrated *in vacuo* to give, after recrystallisation from EtOAc/hexane, 2-hydroxy-2-phenylpropanoic acid **S1** as a colourless solid (1.27 g, 7.6 mmol, 76%); **mp** 81-83 °C [lit[4] 89-91 °C]; **^{1}H NMR** (500 MHz, $CDCl_3$) δ_H: 1.83 (3H, s, C(2)CH_3), 7.29-7.41 (3H, m, PhC(2,4,6)*H*), 7.55-7.61 (2H, m, PhC(3,5)*H*). Data in agreement with literature.[5]

2-Hydroxy-*N,N*-dimethyl-2-phenylpropanamide 2

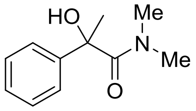

Following general procedure E, racemic 2-hydroxy-2-phenylpropanoic acid **S1** (415 mg, 2.5 mmol), CDI (446 mg, 2.75 mmol) and dimethylamine (2.8 mL, 2 M in THF) in CH_2Cl_2 (15 mL) gave, after recrystallisation from EtOAc/hexane, 2-hydroxy-*N*,*N*-dimethyl-2-phenylpropanamide **2** as colourless crystals (577 mg, 3.0 mmol, 60%); **mp** 93-95 °C [lit[6] 97-99 °C]; **IR** ν_{max} (film): 3358 (O-H), 2932 (C-H), 1618 (C=O), 1447 (C=C), 1090 (C-O); **^{1}H NMR** (500 MHz, $CDCl_3$) δ_H: 1.82 (3H, s, C(2)CH_3), 2.61 (3H, s, N(CH_3)CH_3), 3.02 (3H, s, N(CH_3)CH_3), 5.31 (1H, s, O*H*), 7.27-7.31 (1H, m, PhC(4)*H*), 7.35-7.36 (4H, m, PhC(2,3,5,6)*H*); **^{13}C NMR** (125 MHz, $CDCl_3$) δ_C: 24.7 (C(OH)CH_3), 37.7 (N(CH_3)CH_3), 38.3 (N(CH_3)CH_3), 74.7 (*C*(2)OH), 125.5 (2 × Ph*C*H), 127.9 (Ph*C*(4)H), 128.9 (2 × Ph*C*H), 143.0 (Ph*C*(1)), 175.2 (*C*=O); **HRMS** (ESI$^+$) $C_{11}H_{15}NNaO_2^+$ ($[M+Na]^+$) requires 216.0989; found 216.0995 (−2.8 ppm).

2-Hydroxy-N-methoxy-N-methyl-2-phenylpropanamide S2

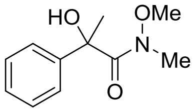

Following general procedure E, racemic 2-hydroxy-2-phenylpropanoic acid **S1** (250 mg, 1.50 mmol), CDI (268 mg, 1.65 mmol) and *N,O*-dimethylhydroxylamine hydrochloride (161 mg, 1.65 mmol) in CH_2Cl_2 (5 mL) gave 2-hydroxy-*N*-methoxy-*N*-methyl-2-phenylpropanamide **S2** as a colourless oil (205 mg, 0.98 mmol, 65%); **IR** ν_{max} (film): 3420 (O-H), 2940 (C-H), 1639 (C=O), 1447 (C=C), 1354 (N-O), 1067 (C-O); **^{1}H NMR** (500 MHz, $CDCl_3$) δ_H: 1.77 (3H, s, C(2)CH_3), 2.71 (3H, s, OCH_3), 3.19 (3H, s, N(OCH_3)CH_3), 4.84 (1H, s, O*H*), 7.24-7.27 (1H, m, PhC(4)*H*), 7.33-7.36 (2H, m, PhC(3,5)*H*), 7.40-7.42 (2H, m, PhC(2,6)*H*); **^{13}C NMR** (125 MHz, $CDCl_3$) δ_C: 24.7 (C(OH)CH_3), 33.5 (N(OCH_3)CH_3), 59.9 (OCH_3), 75.8 (*C*(2)OH), 125.7 (Ph*C*(2,6)H),

127.6 (Ph*C*(4)H), 128.4 (Ph*C*(3,5)H), 143.6 (Ph*C*(1)), 175.3 (*C*=O); **HRMS** (NSI^+) $C_{11}H_{16}NO_3^+$ ($[M+H]^+$) requires 210.1125; found 210.1124 (−0.3 ppm).

2-Hydroxy-*N*,2-diphenylpropanamide 3

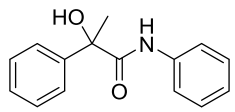

Following general procedure E, racemic 2-hydroxy-2-phenylpropanoic acid **S1** (499 mg, 3.0 mmol), CDI (535 mg, 3.3 mmol) and aniline (0.3 mL, 307 mg, 3.3 mmol) in CH_2Cl_2 (10 mL) gave, after recrystallisation from EtOAc/hexane, 2-hydroxy-*N*,2-diphenylpropanamide **3** as colourless crystals (504 mg, 2.1 mmol, 70%); **mp** 128-130 °C; **IR** ν_{max} (film): 3366 (O-H, N-H), 1665 (C=O), 1525, 1443 (C=C), 1313 (C=C); **^{1}H NMR** (500 MHz, $CDCl_3$) δ_H: 1.92 (3H, s, C*H*$_3$), 2.99 (1H, s, O*H*), 7.08-7.12 (1H, m, NHPhC(4)*H*), 7.28-7.34 (3H, m, NHPhC(3,5)*H*, C(2)PhC(4)*H*), 7.37-7.41 (2H, m, C(2)PhC(3,5)*H*), 7.52-7.55 (2H, m, NHPhC(2,6)*H*), 7.62-7.65 (2H, m, C(2)PhC(2,6)*H*), 8.52 (1H, s, N*H*); **^{13}C NMR** (125 MHz, $CDCl_3$) δ_C: 27.5 (*C*H$_3$), 77.1 (*C*(2)OH), 119.7 (NHPh*C*(2,6)H), 124.6 (NHPh*C*(4)H), 125.4 (C(2)Ph*C*(2,6)H), 128.3 (C(2)Ph*C*(4)H), 128.8 (C(2)Ph*C*(3,5)H), 129.1 (NHPh*C*(3,5)H), 137.5 (NHPh*C*(1)), 142.9 (C(2)Ph*C*(1)), 172.4 (*C*=O); **HRMS** (NSI^+) $C_{15}H_{16}NO_2^+$ ($[M+H]^+$) requires 242.1176; found 242.1178 (+1.0 ppm).

***N*-Benzyl-2-hydroxy-2-phenylpropanamide S3**

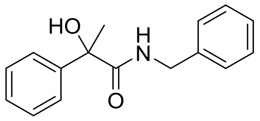

Following general procedure E, racemic 2-hydroxy-2-phenylpropanoic acid **S1** (499 mg, 3.0 mmol), CDI (535 mg, 3.3 mmol) and benzylamine (0.36 mL, 354 mg, 3.3 mmol) in CH_2Cl_2 (10 mL) gave, after recrystallisation from EtOAc/hexane, *N*-benzyl-2-hydroxy-2-phenylpropanamide **S3** as colourless crystals (696 mg, 2.73 mmol, 91%); **mp** 114-116 °C; **IR** ν_{max} (film): 3345 (O-H, N-H), 1651 (C=O), 1522, 1495 (C=C), 1447 (C=C); **^{1}H NMR** (500 MHz, $CDCl_3$) δ_H: 1.86 (3H, s, C*H*$_3$), 3.12 (1H, s, O*H*), 4.40 (2H, app. d, *J* 5.9, C*H*$_2$Ph), 6.80 (1H, s, N*H*), 7.16-7.17 (2H, m, CH$_2$PhC(2,6)*H*), 7.24-7.32 (4H, m, CH$_2$PhC(3,4,5)*H*, C(2)PhC(4)*H*), 7.34-7.38 (2H, m, C(2)PhC(3,5)*H*), 7.55-7.58 (2H, m, C(2)PhC(2,6)*H*); **^{13}C NMR** (125 MHz, $CDCl_3$) δ_C: 27.3 (*C*H$_3$), 43.7 (*C*H$_2$Ph), 76.5 (*C*(2)OH), 125.5 (C(2)Ph*C*(2,6)H), 127.6 (CH$_2$Ph*C*(2,6)H, CH$_2$Ph*C*(4)H), 128.1 (C(2)Ph*C*(4)H), 128.7 (C(2)Ph*C*(3,5)H), 128.8 (CH$_2$Ph*C*(3,5)H), 135.1 (CH$_2$Ph*C*(1)), 143.2 (C(2)Ph*C*(1)), 174.4 (*C*=O); **HRMS** (NSI^+) $C_{16}H_{18}NO_2^+$ ($[M+H]^+$) requires 256.1332; found 256.1336 (+1.5 ppm).

2-Hydroxy-*N*-isopropyl-2-phenylpropanamide S4

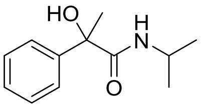

Following general procedure E, racemic 2-hydroxy-2-phenylpropanoic acid **S1** (415 mg, 2.5 mmol), CDI (446 mg, 2.75 mmol) and isopropylamine (0.23 mL, 163 mg, 2.75 mmol) in CH_2Cl_2 (15 mL) gave, after recrystallisation from EtOAc/hexane, 2-hydroxy-*N*-isopropyl-2-phenylpropanamide **S4** as colourless crystals (270 mg, 1.3 mmol, 52%); **mp** 63-64 °C; **IR** ν_{max} (film): 3387 (O-H, N-H), 2974 (C-H), 1651 (C=O), 1522, 1368 (C=C), 1146 (C-O); ^{1}**H NMR** (500 MHz, $CDCl_3$) δ_H: 1.09 (3H, d, *J* 6.6, CH(C*H₃*)CH₃), 1.11 (3H, d, *J* 6.6, CH(CH₃)C*H₃*), 1.78 (3H, s, C(2)C*H₃*), 3.37 (1H, s, O*H*), 3.99 (1H, m, C*H*), 6.26 (1H, d, *J* 5.8, N*H*), 7.27-7.31 (1H, m, PhC(4)*H*), 7.33-7.37 (2H, m, PhC(3,5)*H*), 7.52-7.54 (2H, m, PhC(2,6)*H*); ^{13}**C NMR** (125 MHz, $CDCl_3$) δ_C: 22.7 (2 × C, -CH(CH₃)*C*H₃), 27.2 (C(2)*C*H₃), 41.7 (*C*H), 76.2 (*C*(2)), 125.5 (Ph*C*(2,6)H), 128.0 (Ph*C*(4)H), 128.6 (Ph*C*(3,5)H), 143.3 (Ph*C*(1)), 173.9 (C=O); **HRMS** (NSI$^+$) $C_{12}H_{18}NO_2^+$ ([M+H]$^+$) requires 208.1332; found 208.1332 (+0.0 ppm).

***N*-Cyclopropyl-2-hydroxy-2-phenylpropanamide S5**

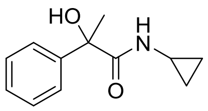

Following general procedure E, racemic 2-hydroxy-2-phenylpropanoic acid **S1** (415 mg, 2.5 mmol), CDI (446 mg, 2.75 mmol) and cyclopropylamine (0.19 mL, 157 mg, 2.75 mmol) in CH_2Cl_2 (10 mL) gave, after recrystallisation from EtOAc/hexane, *N*-cyclopropyl-2-hydroxy-2-phenylpropanamide **S5** as colourless crystals (230 mg, 1.1 mmol, 49%); **mp** 73-75 °C; **IR** ν_{max} (film): 3375 (O-H, N-H), 3005 (C-H), 1647 (C=O), 1510, 1447 (C=C), 1364 (C=C); ^{1}**H NMR** (500 MHz, $CDCl_3$) δ_H: 0.36-0.49 (2H, m, 2 × CH_aH$_b$), 0.69-0.77 (2H, m, 2 × CH$_a$$H_b$), 1.76 (3H, s, C*H₃*), 2.62-2.67 (1H, m, C*H*), 3.50 (1H, s, O*H*), 6.58 (1H, s, N*H*), 7.26-7.30 (1H, m, PhC(4)*H*), 7.32-7.35 (2H, m, PhC(3,5)*H*), 7.51-7.53 (2H, m, PhC(2,6)*H*); ^{13}**C NMR** (125 MHz, $CDCl_3$) δ_C: 6.59 (*C*H₂), 6.61 (*C*H₂), 22.7 (*C*H), 27.3 (*C*H₃), 76.2 (*C*(2)), 125.4 (Ph*C*(2,6)H), 127.9 (Ph*C*(4)H), 128.6 (Ph*C*(3,5)H), 143.3 (Ph*C*(1)), 176.3 (C=O); **HRMS** (NSI$^+$) $C_{12}H_{16}NO_2^+$ ([M+H]$^+$) requires 206.1176; found 206.1176 (+0.2 ppm).

***N*-Cyclohexyl-2-hydroxy-2-phenylpropanamide S6**

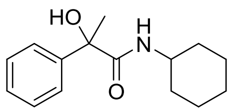

Following general procedure E, racemic 2-hydroxy-2-phenylpropanoic acid **S1** (415 mg, 2.5 mmol), CDI (446 mg, 2.75 mmol) and cyclohexylamine (0.32 mL, 273 mg, 2.75 mmol) in CH_2Cl_2 (15 mL) gave, after recrystallisation from EtOAc/hexane, *N*-cyclohexyl-2-hydroxy-2-phenylpropanamide **S6** as colourless crystals (348 mg, 1.41 mmol, 56%); **mp** 66-68 °C; **IR** ν_{max} (film): 3391 (O-H, N-H), 2930 (C-H), 1647 (C=O), 1520, 1447 (C=C), 1366 (C=C); ^{1}**H NMR** (500 MHz, $CDCl_3$) δ_H: 1.05-1.18 (3H, m, C(3′,5′)H_aH$_b$, C(4′)H_aH$_b$), 1.27-1.38 (2H, m, C(2′,6′)H_aH$_b$), 1.56-1.61 (1H, m, C(4′)H$_a$$H_b$), 1.63-1.67 (2H, m, C(2′,6′)H$_a$$H_b$), 1.80 (3H, s, C*H₃*), 1.82-1.86 (2H,

m, C(3',5')H_aH_b), 3.29 (1H, s, O*H*), 3.66-3.73 (1H, m, C(1')*H*), 6.24 (1H, d, *J* 7.2, N*H*), 7.28-7.31 (1H, m, PhC(4)*H*), 7.34-7.37 (2H, m, PhC(3,5)*H*), 7.52-7.55 (2H, m, PhC(2,6)*H*); **^{13}C NMR** (125 MHz, $CDCl_3$) δ_C: 24.8 (*C*(2',6')H), 25.6 (*C*(4')H), 33.0 (*C*(3',5')H), 76.2 (*C*(2)), 125.5 (Ph*C*(2,6)H), 128.0 (Ph*C*(4)H), 128.6 (Ph*C*(3,5)H), 143.4 (Ph*C*(1)), 173.9 (C=O); **HRMS** (NSI^+) $C_{15}H_{22}NO_2^+$ ($[M+H]^+$) requires 248.1645; found 248.1647 (+0.8 ppm).

2-Hydroxy-1,2-diphenylpropan-1-one 4

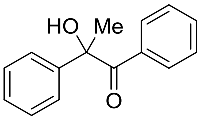

Following general procedure D, benzil (2.10 g, 10 mmol) and methylmagnesium bromide (4 mL, 12 mmol, 3.0 M) in anhydrous THF (50 mL) gave, after column chromatography (CH_2Cl_2:EtOAc, 9:1; R_F 0.45), 2-hydroxy-1,2-diphenylpropan-1-one **4** as a colourless solid (1.28 g, 5.7 mmol, 57%); **mp** 49-51 °C; **IR** ν_{max} (film): 3437 (OH), 2995, 1667 (C=O), 1595, 1445; **^{1}H NMR** (400 MHz, $CDCl_3$) δ_H: 1.90 (3H, s, C(2)C*H*$_3$), 4.77 (1H, s, O*H*), 7.27-7.35 (3H, m, C(2)PhC(3,4,5)*H*), 7.36-7.42 (2H, m, C(O)PhC(3,5)*H*), 7.43-7.48 (3H, m, C(2)PhC(2,6)*H*, C=OPhC(4)*H*), 7.66-7.70 (2H, m, C(O)PhC(2,6)*H*). Data in agreement with literature.[7]

Methyl 2-hydroxy-2-phenypropanoate 5

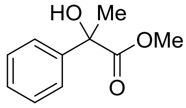

Following the procedure outlined by Ruiz,[8] *n*-BuLi (8.8 mL, 22 mmol, 2.5 M) was added to a solution of *i*-Pr_2NH (3.1 mL, 22 mmol) in anhydrous THF (50 mL) under a N_2 atmosphere at 0 °C. The LDA solution was stirred for 30 minutes, cooled to –78 °C and methyl mandelate (1.66 g, 10 mmol) was added and the reaction mixture stirred for 15 mins. Methyl iodide (623 µL, 10 mmol) was added and the reaction mixture stirred overnight at r.t.. HCl (20 mL) was added until pH 1 was reached. The aqueous layer was extracted with EtOAc (3 × 30 mL). The organic layers were combined, dried over $MgSO_4$, filtered and concentrated *in vacuo* to give, after column chromatography (Petrol/EtOAc, 4:1; R_F 0.28), methyl 2-hydroxy-2-phenypropanoate **5** as a yellow oil (242 mg, 1.3 mmol, 13%); **IR** ν_{max} (film): 3493 (OH), 2955, 1724 (C=O), 1447, 1250, 1144; **^{1}H NMR** (500 MHz, $CDCl_3$) δ_H: 1.79 (3H, s, C(2)C*H*$_3$), 3.76 (1H, s, O*H*), 3.78 (3H, s, OC*H*$_3$), 7.27-7.32 (1H, m, PhC(4)*H*), 7.33-7.39 (2H, m, PhC(3,5)*H*), 7.53-7.57 (2H, m, PhC(2,6)*H*). Data in agreement with literature.[8]

Ethyl 2-hydroxy-2-phenylpropanoate 6

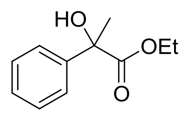

Following the procedure outlined by Ruiz,[8] *n*-BuLi (17.6 mL, 44 mmol, 2.5 M) was added to a solution of *i*-Pr_2NH (6.2 mL, 44 mmol) in anhydrous THF (50 mL) under a N_2 atmosphere at 0

°C. The LDA solution was stirred for 30 minutes, cooled to –78 °C and ethyl mandelate (3.60 g, 20 mmol) was added and the reaction mixture stirred for 15 mins. Methyl iodide (1.25 mL, 20 mmol) was added and the reaction mixture stirred overnight at r.t.. HCl (20 mL) was added until pH 1 was reached. The aqueous layer was extracted with EtOAc (3 × 30 mL). The organic layers were combined, dried over $MgSO_4$, filtered and concentrated *in vacuo* to give, after column chromatography (Petrol/EtOAc, 4:1; R_F 0.41), ethyl 2-hydroxy-2-phenylpropanoate **6** as a yellow oil (1.82 g, 4.7 mmol, 47%); **IR** ν_{max} (film): 3501 (OH), 2982, 1721 (C=O), 1447, 1244, 1144; **^{1}H NMR** (400 MHz, $CDCl_3$) δ_H: 1.25 (3H, t, *J* 7.1, OCH_2CH_3), 1.79 (3H, s, C(2)CH_3), 3.92 (1H, br s, O*H*), 4.15-4.30 (2H, m, OCH_2CH$_3$), 7.26-7.31 (1H, m, PhC(4)*H*), 7.32-7.38 (2H, m, PhC(2,6)*H*), 7.55-7.60 (2H, m, PhC(3,5)*H*). Data in agreement with literature.[2]

***tert*-Butyl 2-hydroxy-2-phenylpropanoate 7**

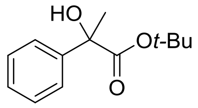

Following general procedure A, 2-phenylpropionic acid (1.37 g, 10 mmol), oxalyl chloride (1.05 mL, 12 mmol), DMF (1 drop), *tert*-butyl alcohol (1.44 mL, 15 mmol) and pyridine (0.81 mL, 10 mmol) in anhydrous CH_2Cl_2 (25 mL) gave, after column chromatography (eluent Petrol/Et_2O, 9:1), the impure ester as a colourless oil (1.79 g). Following general procedure B, the impure ester (1.86 g, 9 mmol), triethyl phosphite (3.09 mL, 18 mmol), cesium carbonate (635 mg, 1.8 mmol, 20 mol%) in DMSO (36 mL) under a O_2 atmosphere was stirred for 48 h to give, after column chromatography (eluent Petrol/Et_2O, 9:1; R_F 0.16), *tert*-butyl 2-hydroxy-2-phenylpropanoate **7** as a colourless oil (644 mg, 2.9 mmol, 29%); **IR** ν_{max} (film): 3503 (OH), 2980, 1715 (C=O), 1447, 1369, 1256, 1140; **^{1}H NMR** (400 MHz, $CDCl_3$) δ_H: 1.44 (9H, s, C(CH_3)$_3$), 1.74 (1H, s, C(2)CH_3), 3.89 (1H, s, O*H*), 7.25-7.30 (1H, m, PhC(4)*H*), 7.32-7.37 (2H, m, Ph(3,5)*H*), 7.54-7.59 (2H, m, PhC(2,6)*H*); **^{13}C NMR** (100 MHz, $CDCl_3$) δ_C: 26.7 (C(2)*C*H$_3$), 27.8 (C(*C*H$_3$)$_3$), 75.6 (*C*(2)), 83.0 (O*C*(CH$_3$)$_3$), 125.2 (Ph*C*(2,6)H), 127.5 (Ph*C*(4)H), 128.2 (Ph*C*(3,5)H), 143.3 (C(2)Ph*C*(1)), 174.9 (*C*=O); **HRMS** (NSI$^+$) $C_{13}H_{18}O_3Na^+$ ($[M+Na]^+$) requires 245.1148; found 245.1150 (+0.8 ppm).

Benzyl 2-hydroxy-2-phenypropanoate 8

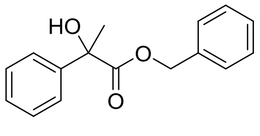

Following general procedure E, racemic 2-hydroxy-2-phenylpropanoic acid (415 mg, 2.5 mmol), CDI (446 mg, 2.75 mmol) and benzyl alcohol (0.29 mL, 2.75 mmol) in CH_2Cl_2 (15 mL) gave, after column chromatography (Isolera 4, Et_2O in petrol, 0% to 25% over 30 CV; R_F 0.14 (10% Et_2O in petrol)), benzyl 2-hydroxy-2-phenypropanoate **8** as a colourless oil (514 mg, 2.0 mmol, 80%); **IR** ν_{max} (film): 3503 (OH), 3032, 1722 (C=O), 1447, 1234, 1142; **^{1}H NMR** (400 MHz, $CDCl_3$) δ_H:1.82 (3H, s, CH_3), 3.79 (1H, s, O*H*), 5.17 (1H, d, *J* 12.3, OCH_aH$_b$Ph), 5.24 (1H, d, *J* 12.4, OCH$_a$$H_b$Ph), 7.23-7.27 (2H, m, CH$_2$PhC(2,6)*H*), 7.29-7.38 (6H, m, C(2)Ph(3,4,5)*H*, CH$_2$PhC(3,4,5)*H*), 7.53-7.58 (2H, m, C(2)Ph(2,6)*H*); **^{13}C NMR** (100 MHz, $CDCl_3$) δ_C: 26.6 (*C*H$_3$), 67.9 (O*C*H$_2$Ph), 75.8 (*C*(2)), 125.2 (C(2)Ph*C*(2,6)H), 127.8 (C(2)Ph*C*(4)H), 127.9 (CH$_2$Ph*C*(2,6)H),

128.3 (CH_2PhC(3,5)H), 128.5 (CH_2PhC(4)H), 128.6 (C(2)Ph*C*(3,5)H), 135.1 (CH_2PhC(1)), 142.6 (C(2)Ph*C*(1)), 175.5 (*C*=O); **HRMS** (NSI^+) $C_{16}H_{16}O_3Na^+$ ($[M+Na]^+$) requires 279.0992; found 279.0992 (+0.1 ppm).

Benzyl 2-hydroxy-2-(naphthalene-2-yl)propanoate 11

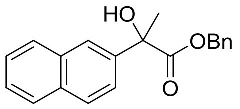

Following general procedure G, *n*-BuLi (8.8 mL, 22 mmol, 2.5 M in hexanes), *i*-Pr_2NH (3.1 mL, 22 mmol), 2-(naphthalen-2-yl)acetic acid (1.86 g, 10 mmol) and iodomethane (1.37 mL, 22 mmol) in anhydrous THF (50 mL) gave 2-(naphthalen-2-yl)propanoic acid (2.00 g, ~10 mmol), which was used without further purification. Following general procedure A, the acid (2.00 g, ~10 mmol), oxalyl chloride (1.05 mL, 12 mmol), DMF (1 drop), benzyl alcohol (1.06 mL, 15 mmol) and pyridine (0.81 mL, 10 mmol) in anhydrous CH_2Cl_2 (25 mL) gave, after column chromatography (eluent Petrol/Et_2O, 9:1; R_F 0.54), the impure ester as a colourless oil (2.41 g, ~8.3 mmol). Following general procedure B, the impure ester (2.41 g, ~8.3 mmol), triethyl phosphite (2.84 mL, 16.6 mmol), cesium carbonate (586 mg, 1.66 mmol, 20 mol%) in DMSO (33 mL) under a O_2 atmosphere was stirred for 24 h to give, after column chromatography (eluent Petrol/Et_2O, 9:1; R_F 0.16), benzyl 2-hydroxy-2-(naphthalene-2-yl)propanoate **11** as a colourless oil (1.07 g, 4.2 mmol, 42%) over 3 steps; **IR** ν_{max} (film): 3489 (OH), 2982, 1724 (C=O), 1454, 1233, 1126; **^{1}H NMR** (400 MHz, $CDCl_3$) δ_H: 1.92 (3H, s, CH_3), 3.82 (1H, br s, O*H*), 5.20 (1H, d, *J* 12.3, OCH_aH_bPh), 5.25 (1H, d, *J* 12.3, OCH_aH_bPh), 7.24-7.29 (2H, m, PhC(2,6)*H*), 7.30-7.35 (3H, m, Ph(3,4,5)*H*), 7.47-7.52 (2H, m, C(2)ArC(6,7)*H*), 7.66 (1H, dd, *J* 8.7, 1.9, C(2)ArC(3)*H*), 7.79-7.86 (3H, m, C(2)ArC(4,5,8)*H*), 8.02 (1H, d, *J* 1.8, C(2)ArC(1)*H*); **^{13}C NMR** (100 MHz, $CDCl_3$) δ_C: 26.6 (CH_3), 68.0 (CH_2Ph), 76.0 (*C*(2)), 123.5 (C(2)Ar*C*(3)H), 124.2 (C(2)Ar*C*(1)H), 126.26 (C(2)Ar*C*(6)H), 126.28 (C(2)Ar*C*(7)H), 127.5 (C(2)Ar*C*(5)H), 128.08 (Ph*C*(2,6)H), 128.13 (C(2)Ar*C*(8)H), 128.4 (Ph*C*(4)H), 128.5 (C(2)Ar*C*(4)H), 128.6 (Ph*C*(3,5)H), 132.8 (C(2)Ar*C*(8a)), 133.0 (C(2)Ar*C*(4a)), 135.1 (Ph*C*(1)), 140.0 (C(2)Ar*C*(2)), 175.5 (*C*=O); **HRMS** (NSI^+) $C_{20}H_{18}O_3Na^+$ ($[M+Na]^+$) requires 329.1148; found 329.1144 (–1.3 ppm).

Benzyl 2-hydroxy-2-(*p*-tolyl)propanoate 12

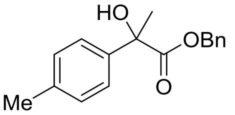

Following general procedure G, *n*-BuLi (8.8 mL, 22 mmol, 2.5 M in hexanes), *i*-Pr_2NH (3.1 mL, 22 mmol), *p*-tolylacetic acid (1.51 g, 10 mmol) and methyl iodide (1.37 mL, 22 mmol) in anhydrous THF (50 mL) gave 2-phenylhexanoic acid (1.64 g, ~10 mmol), which was used without further purification. Following general procedure A, the acid (1.64 g, ~10 mmol), oxalyl chloride (1.05 mL, 12 mmol), DMF (1 drop), benzyl alcohol (1.06 mL, 15 mmol) and pyridine (0.81 mL, 10 mmol) in anhydrous CH_2Cl_2 (25 mL) gave, after column chromatography (eluent Petrol/Et_2O, 9:1), the impure ester as a colourless oil (1.97 g, ~7.75 mmol). Following general procedure B, the impure ester (1.97 g, ~7.75 mmol), triethyl phosphite (2.66 mL, 15.5 mmol), cesium carbonate (547 mg, 1.55 mmol, 20 mol%) in DMSO (31 mL) under a O_2 atmosphere was stirred for 24 h to give, after column chromatography

(eluent Petrol/Et_2O, 9:1; R_F 0.08), benzyl 2-hydroxy-2-(*p*-tolyl)propanoate **12** as a colourless oil (1.36 g, 5.0 mmol, 65%) over 3 steps; **IR** ν_{max} (film): 3507 (OH), 2982, 1724 (C=O), 1454, 1234, 1143; **^{1}H NMR** (400 MHz, $CDCl_3$) δ_H: 1.82 (3H, s, C(2)C*H*$_3$), 2.37 (3H, s, ArC(4)C*H*$_3$), 3.67 (1H, s, O*H*), 5.17 (1H, d, *J* 12.4, OC*H*$_a$H$_b$Ph), 5.25 (1H, d, *J* 12.4, OCH$_a$*H*$_b$Ph), 7.15-7.20 (2H, m, C(2)ArC(3,5)*H*), 7.25-7.30 (2H, m, PhC(2,6)*H*), 7.33-7.39 (3H, m, PhC(3,4,5)*H*), 7.44-7.48 (2H, m, C(2)PhC(2,6)*H*); **^{13}C NMR** (100 MHz, $CDCl_3$) δ_C: 21.1 (ArC(4)*C*H$_3$), 26.6 (C(2)*C*H$_3$), 67.9 (O*C*H$_2$Ph), 75.7 (*C*(2)), 125.2 (C(2)Ar*C*(2,6)H), 128.0 (Ph*C*(2,6)H), 128.5 (Ph*C*(4)H), 128.6 (Ph*C*(3,5)H), 129.0 (C(2)Ar*C*(3,5)H), 135.2 (Ph*C*(1)), 137.6 (C(2)Ar*C*(1)), 139.8 (C(2)Ar*C*(4)), 175.6 (*C*=O); **HRMS** (NSI^+) $C_{17}H_{18}O_3Na^+$ ($[M+Na]^+$) requires 293.1148; found 293.1148 (–0.1 ppm).

2-(4-(*tert*-Butyl)phenyl)-2-hydroxypropanoic acid S7

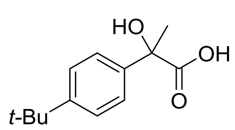

Following general procedures C and D, 1-bromo-4-(*tert*-butyl)benzene (4.3 mL, 25 mmol), magnesium turnings (972 mg, 40 mmol) and pyruvic acid (0.7 mL, 10 mmol) in anhydrous THF (50 mL) gave, after recrystallisation from EtOAc/hexane, 2-(4-(*tert*-butyl)phenyl)-2-hydroxypropanoic acid **S7** as colourless crystals (487 mg, 2.19 mmol, 22%); **mp** 130-132 °C [lit[9] 110-111 °C]; **IR** ν_{max} (film): 3420 (O-H, CO_2-H), 2955 (C-H), 1740 (C=O), 1244 (C=C), 1148 (C-O); **^{1}H NMR** (500 MHz, $CDCl_3$) δ_H: 1.31 (9H, s, C(C*H*$_3$)$_3$), 1.82 (3H, s, C(2)C*H*$_3$), 7.37-7.40 (2H, m, ArC(3,5)*H*), 7.49-7.52 (2H, m, ArC(2,6)*H*); **^{13}C NMR** (125 MHz, $CDCl_3$) δ_C: 26.7 (C(2)*C*H$_3$), 31.4 (C(*C*H$_3$)$_3$), 34.7 (*C*(CH$_3$)$_3$), 75.7 (*C*(2)), 125.0 (Ar*C*(2,6)H), 125.6 (Ar*C*(3,5)H), 138.9 (Ar*C*(1)), 151.3 (Ar*C*(4)), 180.3(*C*O$_2$H); **HRMS** (NSI^-) $C_{13}H_{17}O_3^-$ ($[M-H]^-$) requires 221.1183; found 221.1180 (–1.4 ppm).

Benzyl 2-(4-(*tert*-butyl)phenyl)-2-hydroxypropanoate 13

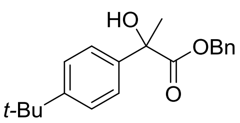

Following general procedure E, 2-(4-(*tert*-butyl)phenyl)-2-hydroxypropanoic acid **S7** (556 mg, 2.5 mmol), CDI (447 mg, 2.75 mmol) and benzyl alcohol (0.29 mL, 2.75 mmol) in CH_2Cl_2 (15 mL) gave, after column chromatography (Isolera 4, Et_2O in petrol, 0% to 10% over 30 CV; R_F 0.24), benzyl 2-(4-(*tert*-butyl)phenyl)-2-hydroxypropanoate **13** as a colourless solid (493 mg, 1.3 mmol, 79%); **mp** 62-63 °C; **IR** ν_{max} (film): 3500 (O-H), 2961 (C-H), 1728 (C=O), 1456 (C=C), 1150 (C-O); **^{1}H NMR** (500 MHz, $CDCl_3$) δ_H: 1.32 (9H, s, C(C*H*$_3$)$_3$), 1.80 (3H, s, C(2)C*H*$_3$), 3.71 (1H, s, O*H*), 5.16 (1H, d, *J* 12.5, OC*H*$_a$H$_b$Ph), 5.23 (1H, d, *J* 12.5, OCH$_a$*H*$_b$Ph), 7.22-7.24 (m, 2H, PhC(2,6)*H*), 7.31-7.37 (5H, m, PhC(3,4,5)*H*, C(2)ArC(3,5)*H*), 7.44-7.47 (2H, m, C(2)ArC(2,6)*H*); **^{13}C NMR** (125 MHz, $CDCl_3$) δ_C: 26.6 (C(2)*C*H$_3$), 31.5 (C(*C*H$_3$)$_3$), 34.6 (*C*(CH$_3$)$_3$), 67.9 (*C*H$_2$), 75.8 (*C*(2)), 125.1 (C(2)Ar*C*(2,6)H), 125.4 (C(2)Ar*C*(3,5)H), 128.0 (Ph*C*(2,6)H), 128.5 (Ph*C*(4)H), 128.7 (Ph*C*(3,5)H), 135.3 (Ph*C*(1)), 139.7 (C(2)Ar*C*(1)), 150.9 (C(2)Ar*C*(4)), 175.7 (*C*=O); **HRMS** (NSI^+) $C_{20}H_{24}NaO_3^+$ ($[M+Na]^+$) requires 335.1618; found 335.1611 (−2.0 ppm).

Benzyl pyruvate S8

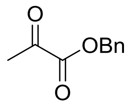

Following the procedure outlined by Yao,[3] to a solution pyruvic acid (695 µL, 10 mmol), benzyl alcohol (2.08 mL, 10 mmol) and pyridine (2.01 mL, 25 mmol) in THF (10 mL) was added dropwise methanesulfonyl chloride (929 µL, 12 mmol) at 0 °C. After stirring for 30 min, the reaction mixture was warmed to r.t. and stirred and monitored by TLC until completion. The reaction was then quenched with water (20 mL), and extracted with Et_2O (3 × 20 mL). The organic phases were combined, dried over Na_2SO_4, filtered and concentrated *in vacuo* to give, after column chromatography (eluent CH_2Cl_2/EtOAc, 9:1), benzyl pyruvate **S8** as a yellow oil (1.02 g, 5.7 mmol, 57%); **IR** ν_{max} (film): 3034, 1728 (C=O), 1497, 1454, 1290, 1265, 1130; **^{1}H NMR** (500 MHz, $CDCl_3$) δ_H: 2.45 (3H, s, C*H*$_3$), 5.26 (2H, s, C*H*$_2$), 7.32-7.46 (5H, m, PhC*H*). Data in agreement with literature.[10]

Benzyl 2-hydroxy-2-(4-methoxyphenyl)propanoate 14

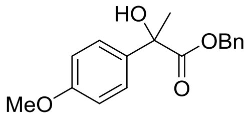

Following general procedure C and D, 4-bromoanisole (1.24 mL, 10 mmol), magnesium turnings (362 mg, 15 mmol) [conc. of Grignard's 0.3 M] and benzyl pyruvate **S8** (890 mg, 5 mmol) in anhydrous THF (20 mL) gave, after column chromatography (Petrol/Et_2O, 9:1; R_F 0.11), benzyl 2-hydroxy-2-(4-methoxyphenyl)propanoate **14** as a colourless oil (412 mg, 1.44 mmol, 29%); **IR** ν_{max} (film): 3491 (OH), 2936, 1724 (C=O), 1608, 1510, 1248; **^{1}H NMR** (400 MHz, $CDCl_3$) δ_H: 1.78 (3H, s, C(2)C*H*$_3$), 3.73 (1H, s, O*H*), 3.80 (3H, s, OC*H*$_3$), 5.15 (1H, d, *J* 12.4, OC*H*$_a$H$_b$Ph), 5.22 (1H, d, *J* 12.4, OCH$_a$*H*$_b$Ph), 6.84-6.88 (2H, m, C(2)ArC(3,5)*H*), 7.22-7.27 (2H, m, PhC(2,6)*H*), 7.31-7.36 (3H, m, PhC(3,4,5)*H*), 7.42-7.47 (2H, m, C(2)ArC(2,6)*H*); **^{13}C NMR** (100 MHz, $CDCl_3$) δ_C: 26.6 (C(2)*C*H$_3$), 55.3 (O*C*H$_3$), 67.8 (O*C*H$_2$Ph), 75.4 (*C*(2)), 113.6 (C(2)Ar*C*(3,5)H), 126.5 (C(2)Ar*C*(2,6)H), 127.9 (Ph*C*(2,6)H), 128.4 (Ph*C*(4)H), 128.5 (Ph*C*(3,5)H), 134.7 (Ph*C*(1)), 135.7 (C(2)Ar*C*(1)), 159.2 (C(2)Ar*C*(4)), 175.7 (*C*=O); **HRMS** (NSI$^+$) $C_{17}H_{18}O_4Na^+$ ([M+Na]$^+$) requires 309.1097; found 309.1097 (–0.1 ppm).

2-Hydroxy-2-(3-methoxyphenyl)propanoic acid S9

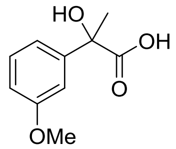

Following general procedures C and D, 3-bromoanisole (3.1 mL, 25 mmol), magnesium turnings (972 mg, 40 mmol) and pyruvic acid (0.7 mL, 10 mmol) in anhydrous THF (50 mL) gave, after recrystallisation from EtOAc/hexane, 2-hydroxy-2-(3-methoxyphenyl)propanoic acid **S9** as colourless crystals (404 mg, 2.06 mmol, 21%), **mp** 104-106 °C; **IR** ν_{max} (film): 3375 (O-H, CO_2-H), 2943 (C-H), 1713 (C=O), 1593, 1256 (C=C), 1138 (C-O); **^{1}H NMR** (500 MHz, $CDCl_3$) δ_H: 1.82 (3H, s, C(2)C*H*$_3$), 3.51 (1H, s, O*H*), 3.82 (3H, s, OC*H*$_3$), 6.85-6.87 (1H, m,

ArC(4)*H*), 7.15-7.17 (2H, m, ArC(2,6)*H*), 7.28-7.31 (1H, m, ArC(5)*H*), 10.64 (1H, br, CO_2*H*); **^{13}C NMR** (125 MHz, $CDCl_3$) δ_C: 26.8 (C(2)*C*H_3), 55.4 (O*C*H_3), 75.8 (*C*(2)), 111.3 (Ar*C*(2)H), 113.6 (Ar*C*(4)H), 117.6 (Ar*C*(6)H), 129.7 (Ar*C*(5)H), 143.5 (Ar*C*(3)), 159.8 (Ar*C*(1)), 179.8 (*C*O_2H); **HRMS** (NSI^+) $C_{10}H_{12}NaO_4^+$ ($[M+Na]^+$) requires 219.0628; found 219.0624 (–1.7 ppm).

Benzyl 2-hydroxy-2-(3-methoxyphenyl)propanoate 15

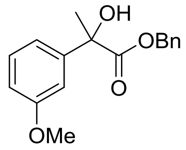

Following general procedure E, racemic 2-hydroxy-2-(3-methoxyphenyl)propanoic acid **S9** (491 mg, 2.5 mmol), CDI (447 mg, 2.75 mmol) and benzyl alcohol (0.29 mL, 2.75 mmol) in CH_2Cl_2 (15 mL), gave, after column chromatography (Isolera 4, Et_2O in petrol, 0% to 10% over 30 CV; R_F 0.24 (20% Et_2O in petrol)), benzyl 2-hydroxy-2-(3-methoxyphenyl)propanoate **15** as a yellow oil (349 mg, 1.2 mmol, 49%); **IR** ν_{max} (film): 3499 (O-H), 2940 (C-H), 1724 (C=O), 1599, 1454 (C=C), 1256, 1234, 1140 (C-O), 1043 (C-O); **^{1}H NMR** (500 MHz, $CDCl_3$) δ_H: 1.82 (3H, s, C(2)C*H_3*), 3.78 (3H, s, OC*H_3*), 3.87 (1H, s, O*H*), 5.20 (1H, d, *J* 12.3, OC*H_a*H_bPh), 5.24 (1H, d, *J* 12.5, OCH_a*H_b*Ph), 6.85-6.87 (1H, m, C(2)ArC(4)*H*), 7.14-7.14 (2H, m, C(2)ArC(2,6)*H*), 7.26-7.29 (3H, m, C(2)ArC(5)*H*, PhC(2,6)*H*), 7.33-7.39 (3H, m, PhC(3,4,5)*H*); **^{13}C NMR** (125 MHz, $CDCl_3$) δ_C: 26.7 (C(2)*C*H_3), 55.2 (O*C*H_3), 67.9 (*C*H_2), 75.9 (*C*(2)), 111.0 (C(2)Ar*C*(2)H), 113.5 (C(2)Ar*C*(4)H), 117.6 (C(2)Ar*C*(6)H), 128.1 (Ph*C*(2,6)H), 128.5 (Ph*C*(4)H), 128.7 (Ph*C*(3,5)H), 129.4 (C(2)Ar*C*(5)H), 135.2 (Ph*C*(1)), 144.4 (C(2)Ar*C*(1)), 159.7 (C(2)Ar*C*(3)), 175.4 (*C*O_2H); **HRMS** (NSI^+) $C_{17}H_{18}NaO_4^+$ ($[M+Na]^+$) requires 309.1097; found 309.1088 (−2.9 ppm).

2-Hydroxy-2-(2-methoxyphenyl)propanoic acid S10

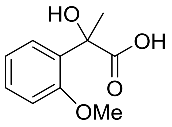

Following general procedures C and D, 2-bromoanisole (3.1 mL, 25 mmol), magnesium turnings (972 mg, 40 mmol) and pyruvic acid (0.7 mL, 10 mmol) in Et_2O (50 mL) gave, after trituration from Et_2O, 2-hydroxy-2-(2-methoxyphenyl)propanoic acid **S10** as colourless crystals (652 mg, 3.32 mmol, 33%); **mp** 134-136 °C; **IR** ν_{max} (film): 3491 (CO_2-H), 3078 (O-H), 2978 (C-H), 1728 (C=O), 1493 (C=C), 1246 (C=C), 1138 (C-O); **^{1}H NMR** (500 MHz, $CDCl_3$) δ_H: 1.85 (3H, s, C(2)C*H_3*), 3.87 (3H, s, OC*H_3*), 4.30 (1H, s, O*H*), 6.92-6.94 (1H, m, ArC(3)*H*), 7.01-7.04 (1H, m, ArC(5)*H*), 7.32-7.36 (1H, m, ArC(4)*H*), 7.45-7.47 (1H, m, ArC(6)*H*), 10.44 (1H, broad, CO_2*H*); **^{13}C NMR** (125 MHz, $CDCl_3$) δ_C: 24.3 (C(2)*C*H_3), 55.7 (O*C*H_3), 74.8 (*C*(2)), 111.4 (Ar*C*(3)H), 121.4 (Ar*C*(5)H), 126.8 (Ar*C*(6)H), 130.0 (Ar*C*(1)), 130.1 (Ar*C*(4)H), 156.6 (Ar*C*(2)), 179.5 (*C*O_2H); **HRMS** (NSI^+) $C_{10}H_{12}NaO_4^+$ ($[M+Na]^+$) requires 219.0628; found 219.0625 (–1.3 ppm).

Benzyl 2-hydroxy-2-(2-methoxyphenyl)propanoate 16

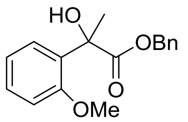

Following general procedure E, 2-hydroxy-2-(2-methoxyphenyl)propanoic acid **S10** (981 mg, 5 mmol), CDI (894 mg, 5.5 mmol) and benzyl alcohol (0.57 mL, 5.5 mmol) in CH_2Cl_2 (15 mL), gave, after column chromatography (Isolera 4, Et_2O in petrol, 0% to 20% over 30 CV; R_F 0.20) benzyl 2-hydroxy-2-(2-methoxyphenyl)propanoate **16** as a colourless oil (631 mg, 2.2 mmol, 44%); **IR** ν_{max} (film): 3468 (O-H), 2940 (C-H), 1738 (C=O), 1491 (C=C), 1246, 1126 (C-O); **^{1}H NMR** (500 MHz, $CDCl_3$) δ_H: 1.81 (3H, s, C(2)C*H*$_3$), 3.55 (3H, s, OC*H*$_3$), 4.03 (1H, s, O*H*), 5.14 (1H, d, *J* 12.4, OC*H*$_a$H$_b$Ph), 5.18 (1H, d, *J* 12.5, OCH$_a$*H*$_b$Ph), 6.81-6.83 (1H, m, C(2)ArC(5)*H*), 6.98-7.02 (2H, m, C(2)ArC(3)*H*), 7.19-7.22 (PhC(2,6)*H*), 7.27-7.32 (4H, m, C(2)ArC(4)*H*, PhC(3,4,5)*H*), 7.44-7.46 (1H, m, C(2)ArC(6)*H*); **^{13}C NMR** (125 MHz, $CDCl_3$) δ_C: 24.2 (C(2)*C*H$_3$), 55.1 (O*C*H$_3$), 66.9 (*C*H$_2$), 74.4 (*C*(2)), 110.9 (C(2)Ar*C*(5)H), 120.9 (C(2)Ar*C*(3)H), 126.4 (C(2)Ar*C*(6)H), 128.2 (Ph*C*(2,4,6)H), 128.5 (Ph*C*(3,5)H), 129.6 (C(2)Ar*C*(4)H), 131.1 (C(2)Ar*C*(1)), 136.0 (Ph*C*(1)), 156.7 (C(2)Ar*C*(2)), 175.9 (*C*=O); **HRMS** (ESI$^+$) $C_{17}H_{18}NaO_4^+$ ([M+Na]$^+$) requires 309.1097; found 309.1090 (−2.2 ppm).

Benzyl 2-(3,5-bis(trifluoromethyl)phenyl)-2-hydroxypropanoate 17

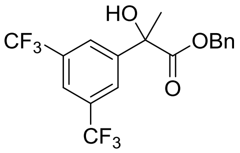

Following general procedure C and D, 1-bromo-3,5-bis(trifluoromethyl)benzene (1.74 mL, 10 mmol), magnesium turnings (362 mg, 15 mmol) [conc. of Grignard's 0.33 M] and benzyl pyruvate **S8** (890 mg, 5 mmol) and (3,5-bis(trifluoromethyl)phenyl)magnesium bromide (18 mL, 6 mmol, 0.33 M) in anhydrous THF (20 mL) gave, after column chromatography (Petrol/Et_2O, 9:1; R_F 0.11), benzyl 2-(3,5-bis(trifluoromethyl)phenyl)-2-hydroxypropanoate **S17** as a light yellow oil (767 mg, 1.95 mmol, 39%); **IR** ν_{max} (film): 3495 (OH), 2967, 1732 (C=O), 1371, 1275, 1124; **^{1}H NMR** (400 MHz, $CDCl_3$) δ_H: 1.82 (3H, s, C*H*$_3$), 3.99 (1H, s, O*H*), 5.19 (1H, d, *J* 12.0, OC*H*$_a$H$_b$Ph), 5.26 (1H, d, *J* 12.0, OCH$_a$*H*$_b$Ph), 7.22-7.26 (2H, m, PhC(3,5)*H*), 7.32-7.37 (3H, m, Ph(2,4,6)*H*), 7.81 (1H, s, C(2)ArC(4)*H*), 8.06 (2H, s, C(2)ArC(2,6)*H*); **^{13}C NMR** (125 MHz, $CDCl_3$) δ_C: 27.5 (*C*H$_3$), 68.9 (*C*H$_2$), 75.2 (*C*(2)), 121.9 (m, C(2)Ar*C*(4)H), 123.2 (q, *J* 273, 2 × *C*F$_3$), 126.0 (Ph*C*(2,6)H), 128.2 (Ph*C*(3,5)H), 128.9 (Ph*C*(4)H), 131.6 (q, *J* 33, C(2)Ar*C*(3,5)H), 134.3 (Ph*C*(1)), 145.0 (C(2)Ar*C*(1)), 174.2 (*C*=O); **^{19}F NMR** (376 MHz, $CDCl_3$) δ_F: –62.8 (2 × C*F*$_3$); **HRMS** (ESI$^+$) no match found, structure of ester derivative **S37** confirmed by NMR spectroscopy and HRMS.

Benzyl 2-hydroxy-2-(thiophene-2-yl)propanoate 18

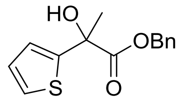

Following general procedure G, *n*-BuLi (8.8 mL, 22 mmol, 2.5 M in hexanes), *i*-Pr_2NH (3.1 mL, 22 mmol), 2-thiopheneacetic acid (1.42 g, 10 mmol) and iodomethane (1.37 mL, 22 mmol) in anhydrous THF (50 mL) gave 2-(thiophen-2-yl)propanoic acid, which was used without further purification (1.54 g, ~10 mmol). Following general procedure A, the acid (1.54 g, ~10 mmol), oxalyl chloride (1.05 mL, 12 mmol), DMF (1 drop), benzyl alcohol (1.06 mL, 15 mmol) and pyridine (0.81 mL, 10 mmol) in anhydrous CH_2Cl_2 (25 mL) gave, after column chromatography (eluent Petrol/Et_2O, 9:1; R_F 0.63), the impure ester (1.83 g, ~7.6 mmol) as a dark red oil. Following general procedure B, the impure ester (1.83 g, ~7.6 mmol), triethyl phosphite (2.62 mL, 15.3 mmol), cesium carbonate (539 mg, 1.53 mmol, 20 mol%) in DMSO (30 mL) under a O_2 atmosphere was stirred for 24 h to give, after column chromatography (eluent Petrol/Et_2O, 9:1; R_F 0.09), benzyl 2-hydroxy-2-(thiophene-2-yl)propanoate **18** as a colourless oil (708 mg, 2.7 mmol, 27%) over 3 steps; **IR** ν_{max} (film): 3495 (OH), 2984, 1724 (C=O), 1454, 1258, 1225, 1134; **^{1}H NMR** (400 MHz, $CDCl_3$) δ_H: 1.85 (3H, s, C*H₃*), 4.02 (1H, s, O*H*), 5.19 (1H, d, *J* 12.3, OC*H*$_a$H$_b$Ph), 5.26 (1H, d, *J* 12.3, OCH$_a$*H*$_b$Ph), 6.95 (1H, dd, *J* 5.1, 3.6, C(2)ArC(4)*H*), 7.05 (1H, dd, *J* 3.6, 1.2, C(2)ArC(3)*H*), 7.23 (1H, dd, *J* 5.1, 1.2, C(2)ArC(5)*H*), 7.27-7.31 (2H, m, PhC(2,6)*H*), 7.32-7.39 (3H, m, PhC(3,4,5)*H*); **^{13}C NMR** (100 MHz, $CDCl_3$) δ_C: 27.8 (*C*H_3), 68.3 (*C*H_2), 74.4 (*C*(2)), 124.2 (C(2)Ar*C*(3)H), 125.2 (C(2)Ar*C*(5)H), 127.0 (C(2)Ar*C*(4)H), 128.0 (Ph*C*(2,6)H), 128.6 (Ph*C*(4)H), 128.7 (Ph*C*(3,5)H), 134.9 (Ph*C*(1)), 147.4 (C(2)Ar*C*(2)), 174.5 (*C*=O); **HRMS** (NSI$^+$) $C_{14}H_{14}O_3SNa^+$ ([M+Na]$^+$) requires 285.0556; found 285.0555 (–0.3 ppm).

Benzyl 2-hydroxy-2-(pyridin-2-yl)propanoate 19

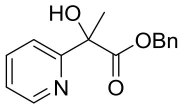

Pyruvic acid (1.1 mL, 15 mmol) and triethylamine (2.1 mL, 15 mmol) were dissolved in CH_2Cl_2 (anhydrous, 150 mL). Benzyl chloroformate (2.1 mL, 15 mmol) was added dropwise and the resulted brown solution was left stirring for 1 h. The solution was diluted with Et_2O, washed with HCl (1 M), $NaHCO_3$ (sat. aq.) and brine. The organic layer was dried over $MgSO_4$, filtered and concentrated *in vacuo*, the crude benzyl pyruvate (12 mmol max) was used with no further purification. In a separate flask, 2-bromopyridine (1.26 mL, 13.2 mmol) was dissolved in anhydrous THF (10 mL) at r.t. under N_2. *i*-PrMgCl (6.6 mL, 2 M in THF, 13.2 mmol) was added dropwise and the resulting solution was stirred for 5 h. To a solution of benzyl pyruvate in anhydrous THF (30 mL) was added newly formed Grignard reagent dropwise at −25 °C. After stirring for 16 h, the reaction was acidified with HCl (1 M) to pH 4 and extracted with EtOAc. The organic phases were combined, washed with brine, dried over $MgSO_4$, filtered and concentrated *in vacuo*. The crude was purified by column chromatography (Isolera 4, Et_2O in petrol, 0% to 50% over 30 CV; R_F 0.28) to give benzyl 2-hydroxy-2-(pyridin-2-yl)propanoate **19** as a yellow oil (140 mg, 0.55 mmol, 5%); **IR** ν_{max} (film): 3468 (O-H), 2988 (C-H), 1732 (C=O), 1589, 1433 (C=C), 1261 (C=C), 1242, 1152, 1113 (C-O); **^{1}H NMR** (500 MHz, $CDCl_3$) δ_H: 1.84 (3H, s, C*H*$_3$), 5.14 (1H, d, *J* 12.3, OCH$_a$*H*$_b$Ph), 5.18 (1H, d, *J* 12.3, OCH$_a$*H*$_b$Ph), 5.51 (1H, s, O*H*), 7.21-7.23 (2H, m, PhC(2,6)*H*), 7.25-7.27 (1H, m, C(2)ArC(5)*H*), 7.28-7.30 (3H,

m, PhC(3,4,5)*H*), 7.52-7.54 (1H, m, C(2)ArC(3)*H*), 7.69-7.73 (1H, m, C(2)ArC(4)*H*), 8.54-8.55 (1H, m, C(2)ArC(6)*H*); ^{13}**C NMR** (125 MHz, $CDCl_3$) δ_C: 25.3 (*C*H_3), 67.4 (*C*H_2), 74.4 (*C*(2)), 120.3 (C(2)Ar*C*(3)H), 123.2 (C(2)Ar*C*(5)H), 127.9 (Ph*C*(2,6)H), 128.3 (Ph*C*(4)H), 128.6 (Ph*C*(3,5)H), 135.6 (Ph*C*(1)), 137.4 (C(2)Ar*C*(4)H), 148.0 (C(2)Ar*C*(6)H), 159.6 (C(2)Ar*C*(2)H), 174.1 (*C*=O); **HRMS** (ESI^+) $C_{15}H_{16}O_3N^+$ ($[M+H]^+$) requires 258.1125; found 258.1122 (−1.1 ppm).

Benzyl 2-hydroxy-2-phenylpent-4-enoate 20

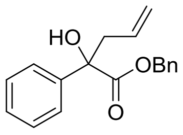

Following general procedure G, *n*-BuLi (8.8 mL, 22 mmol, 2.5 M in hexanes), *i*-Pr_2NH (3.1 mL, 22 mmol), phenylacetic acid (1.36 g, 10 mmol) and allyl bromide (1.90 mL, 22 mmol) in anhydrous THF (50 mL) gave 2-phenylpent-4-enoic acid (1.76 g, ~10 mmol), which was used without further purification. Following general procedure A, the acid (1.76 g, 10 mmol), oxalyl chloride (1.05 mL, 12 mmol), DMF (1 drop), benzyl alcohol (1.06 mL, 15 mmol) and pyridine (0.81 mL, 10 mmol) in anhydrous CH_2Cl_2 (25 mL) gave, after column chromatography (eluent Petrol/Et_2O, 9:1), the impure ester (1.79 g, ~6.73 mmol) as a colourless oil. Following general procedure B, the impure ester (1.79 g, ~6.73 mmol), triethyl phosphite (2.3 mL, 13.5 mmol), cesium carbonate (476 mg, 1.35 mmol, 20 mol%) in DMSO (27 mL) under a O_2 atmosphere was stirred for 24 h to give, after column chromatography (eluent Petrol/Et_2O, 9:1; R_F 0.11), benzyl 2-hydroxy-2-phenylpent-4-enoate **20** as a colourless oil (1.04 g, 3.7 mmol, 37%) over 3 steps; **IR** ν_{max} (film): 3503 (OH), 3034, 1724 (C=O), 1497, 1449, 1263; ^{1}**H NMR** (400 MHz, $CDCl_3$) δ_H: 2.79 (1H, ddt, *J* 14.0, 6.5, 1.3, C(2)CH_aH$_b$CH=CH$_2$), 3.00 (1H, ddt, *J* 14.0, 7.6, 0.9, C(2)CH$_a$$H_b$CH=CH$_2$), 3.77 (1H, br s, O*H*), 5.08-5.17 (3H, m, C(2)CH$_2$CH=CH_2, OCH_aH$_b$Ph), 5.23 (1H, d, *J* 12.2, OCH$_a$$H_b$Ph), 5.77 (1H, dddd, *J* 17.0, 10.3, 7.6, 6.6, C(2)CH$_2$C*H*=CH$_2$), 7.24-7.39 (8H, m, C(2)PhC(3,4,5)*H*, OCH$_2$PhC*H*), 7.58-7.64 (2H, m, C(2)PhC(2,6)*H*); ^{13}**C NMR** (100 MHz, $CDCl_3$) δ_C: 44.0 (C(2)*C*H$_2$CH=CH$_2$), 68.1 (O*C*H$_2$Ph), 78.1 (*C*(2)), 119.5 (C(2)CH$_2$CH=*C*H$_2$), 125.6 (C(2)Ph*C*(2,6)H), 127.9 (C(2)Ph*C*(4)H), 128.2 (OCH$_2$Ph*C*(2,6)H), 128.3 (OCH$_2$Ph*C*(3,5)H), 128.56 (OCH$_2$Ph*C*(4)H), 128.62 (C(2)Ph*C*(3,5)H), 132.2 (C(2)CH$_2$*C*H=CH$_2$), 135.0 (OCH$_2$Ph*C*(1)), 141.2 (C(2)Ph*C*(1)), 174.5 (*C*=O); **HRMS** (NSI^+) $C_{18}H_{18}O_3Na^+$ ($[M+Na]^+$) requires 305.1148; found 305.1150 (+0.6 ppm). Data in agreement with literature.[29]

Benzyl 2-hydroxy-2-phenylbutanoate 21

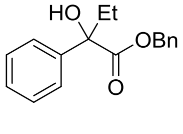

Following general procedure G, *n*-BuLi (8.8 mL, 22 mmol, 2.5 M in hexanes), *i*-Pr_2NH (3.1 mL, 22 mmol), phenylacetic acid (1.36 g, 10 mmol) and bromoethane (1.62 mL, 22 mmol) in anhydrous THF (50 mL) gave 2-phenylbutanoic acid (1.64 g, ~10 mmol), which was used without further purification. Following general procedure A, the acid (1.64 g, ~10 mmol), oxalyl chloride (1.05 mL, 12 mmol), DMF (1 drop), benzyl alcohol (1.06 mL, 15 mmol) and pyridine (0.81 mL, 10 mmol) in anhydrous CH_2Cl_2 (25 mL) gave, after column chromatography (eluent Petrol/Et_2O, 9:1; R_F 0.63), the impure ester (1.98 g, ~7.8 mmol) as a

colourless oil. Following general procedure B, the impure ester (1.98 g, ~7.8 mmol), triethyl phosphite (2.67 mL, 15.6 mmol), cesium carbonate (550 mg, 1.56 mmol, 20 mol%) in DMSO (31 mL) under a O_2 atmosphere was stirred for 24 h to give, after column chromatography (eluent Petrol/Et_2O, 9:1; R_F 0.16), benzyl 2-hydroxy-2-phenylbutanoate **21** as a colourless oil (945 mg, 3.5 mmol, 35%) over 3 steps; **IR** ν_{max} (film): 3516 (OH), 2968, 1721 (C=O), 1497, 1449, 1225, 1142; **^{1}H NMR** (400 MHz, $CDCl_3$) δ_H: 0.89 (3H, t, *J* 7.3, CH_2CH_3), 2.05 (1H, dq, *J* 14.5, 7.3 C(2)$CH_aH_bCH_3$), 2.26 (1H, dq, *J* 14.5, 7.3 C(2)$CH_aH_bCH_3$), 3.77 (1H, s, O*H*), 5.15 (1H, d, *J* 12.3, OCH_aH_bPh), 5.25 (1H, d, *J* 12.3, OCH_aH_bPh), 7.24-7.37 (8H, m, OCH_2PhCH, C(2)PhC(3,4,5)*H*), 7.57-7.62 (2H, m, C(2)PhC(2,6)*H*); **^{13}C NMR** (100 MHz, $CDCl_3$) δ_C: 8.0 (CH_2CH_3), 32.5 (CH_2CH_3), 68.0 (OCH_2Ph), 78.8 (*C*(2)), 125.7 (C(2)Ph*C*(2,6)H), 127.7 (C(2)Ph*C*(4)H), 128.1 (OCH_2Ph*C*(2,6)H), 128.2 (OCH_2Ph*C*(3,5)H), 128.5 (OCH_2Ph*C*(4)H), 128.6 (C(2)Ph*C*(3,5)H), 135.1 (OCH_2Ph*C*(1)), 141.6 (C(2)Ph*C*(1)), 175.3 (*C*=O); **HRMS** (NSI^+) $C_{17}H_{18}O_3Na^+$ ($[M+Na]^+$) requires 293.1148; found 293.1148 (–0.1 ppm). Data in agreement with literature.[11]

Benzyl 2-hydroxy-2-phenylhexanoate 22

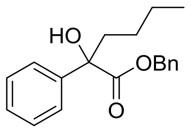

Following general procedure G, *n*-BuLi (8.8 mL, 22 mmol, 2.5 M in hexanes), *i*-Pr_2NH (3.1 mL, 22 mmol), phenylacetic acid (1.36 g, 10 mmol) and *n*-butyl bromide (1.90 mL, 22 mmol) in anhydrous THF (50 mL) gave 2-phenylhexanoic acid (1.92 g, ~10 mmol), which was used without further purification. Following general procedure A, the acid (1.92 g, ~10 mmol), oxalyl chloride (1.05 mL, 12 mmol), DMF (1 drop), benzyl alcohol (1.06 mL, 15 mmol) and pyridine (0.81 mL, 10 mmol) in anhydrous CH_2Cl_2 (25 mL) gave, after column chromatography (eluent Petrol/Et_2O, 9:1), the impure ester (2.33 g, ~8.26 mmol) as a colourless oil. Following general procedure B, the impure ester (2.33 g, ~8.26 mmol), triethyl phosphite (2.83 mL, 16.5 mmol), cesium carbonate (582 mg, 1.65 mmol, 20 mol%) in DMSO (33 mL) under a O_2 atmosphere was stirred for 24 h to give, after column chromatography (eluent Petrol/Et_2O, 9:1; R_F 0.09), benzyl 2-hydroxy-2-phenylhexanoate **22** as a colourless oil (1.28 g, 4.3 mmol, 43%) over 3 steps; **IR** ν_{max} (film): 3503 (OH), 2957, 1722 (C=O), 1497, 1456, 1213, 1144; **^{1}H NMR** (400 MHz, $CDCl_3$) δ_H: 0.85 (3H, t, *J* 7.2, C(6)H_3), 1.10-1.21 (1H, m, C(4)H_aH_b), 1.29 (2H, m, C(5)H_2), 1.34-1.45 (1H, m, C(4)H_aH_b), 2.02 (1H, ddd, *J* 13.7, 11.9, 4.3, C(3)H_aH_b), 2.20 (1H, ddd, *J* 13.7, 11.6, 4.4, C(3)H_aH_b), 3.73 (1H, br s, O*H*), 5.15 (1H, d, *J* 12.3, OCH_aH_bPh), 5.26 (1H, d, *J* 12.3, OCH_aH_bPh), 7.24-7.38 (8H, m, C(2)PhC(3,4,5)*H*, OCH_2PhCH), 7.58-7.63 (2H, m, C(2)PhC(2,6)*H*); **^{13}C NMR** (100 MHz, $CDCl_3$) δ_C: 14.0 (*C*(6)H_3), 22.8 (*C*(5)H_2), 25.8 (*C*(4)H_2), 39.3 (*C*(3)H_2), 68.0 (OCH_2Ph), 78.4 (*C*(2)), 125.6 (C(2)Ph*C*(2,6)H), 127.7 (C(2)Ph*C*(4)H), 128.1 (OCH_2Ph*C*(2,6)H), 128.2 (OCH_2Ph*C*(3,5)H), 128.5 (OCH_2Ph*C*(4)H), 128.6 (C(2)Ph*C*(3,5)H), 135.1 (OCH_2Ph*C*(1)), 141.8 (C(2)Ph*C*(1)), 174.5 (*C*=O); **HRMS** (NSI^+) $C_{19}H_{22}O_3Na^+$ ($[M+Na]^+$) requires 321.1461; found 321.1462 (+0.3 ppm).

Benzyl 2-oxo-2-phenylacetate S11

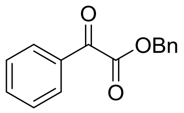

Following general procedure A, phenylglyoxylic acid (3.00 g, 20 mmol), oxalyl chloride (2.09 mL, 24 mmol), DMF (1 drop), benzyl alcohol (3.12 mL, 30 mmol) and pyridine (1.61 mL, 20 mmol) in anhydrous CH_2Cl_2 (25 mL) gave, after column chromatography (eluent CH_2Cl_2/Et_2O, 9:1), benzyl 2-oxo-2-phenylacetate **S11** as a yellow oil (3.42 g, 14.3 mmol, 71%); **IR** ν_{max} (film): 3065, 1732 (C=O), 1686 (C=O), 1595, 1450, 1292, 1192, 1171; **^{1}H NMR** (400 MHz, $CDCl_3$) δ_H: 5.42 (2H, s, OC*H*$_2$Ph), 7.34-7.53 (7H, m, C(2)PhC(3,5)*H*, OCH$_2$PhC(2,3,4,5,6)*H*), 7.62-7.68 (1H, m, C(2)PhC(4)*H*), 7.94-8.00 (2H, m, C(2)PhC(2,6)*H*); Data in agreement with literature.[1]

Benzyl 3,3,3-trifluoro-2-hydroxy-2-phenylpropanoate 23

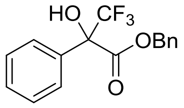

Following the method by de Frugulhetti *et al*,[12] benzyl 2-oxo-2-phenylacetate **S11** (1.28 g, 5.34 mmol) was dissolved in anhydrous THF (10 mL). (Trifluoromethyl)trimethylsilane (1.48 mL, 10.7mmol) was added, followed by cesium fluoride (81 mg, 0.53 mmol, 10 mol%) and the reaction stirred at r.t. for 24 h under N_2. On completion, the solution was extracted with water (20 mL). The aqueous phase was then extracted with CH_2Cl_2 (3 × 15 mL). The organic layers were combined, dried ($MgSO_4$), filtered and concentrated *in vacuo*. The crude mixture was then purified *via* column chromatography (eluent hexane/Et_2O, 9:1) to give the TMS-protected alcohol as a yellow oil. The product was dissolved in in THF/H_2O (12 mL, 5:1), treated with HCl (1 M) and allowed to stir for 1 h. The reaction mixture was then quenched with $NaHCO_3$ and the organic layer extracted with EtOAc, washed with brine, dried ($MgSO_4$), filtered and concentrated *in vacuo* to give, after column chromatography (eluent Petrol/Et_2O, 9:1; R_F 0.21), benzyl 3,3,3-trifluoro-2-hydroxy-2-phenylpropanoat **23** as a pale yellow solid (1.28 g, 4.1 mmol, 78%); **IR** ν_{max} (film): 3483 (OH), 3036, 1736 (C=O), 1452, 1279, 1163; **^{1}H NMR** (400 MHz, $CDCl_3$) δ_H: 4.33 (1H, s, O*H*), 5.35 (1H, d, *J* 12.2, OC*H*$_a$H$_b$Ph), 5.41 (1H, d, *J* 12.2, OCH$_a$*H*$_b$Ph), 7.33-7.44 (8H, m, C(2)Ph(3,4,5)*H*, OCH$_2$PhC*H*), 7.75-7.81 (2H, m, C(2)PhC(2,6)*H*); **^{13}C NMR** (100 MHz, $CDCl_3$) δ_C: 69.8 (O*C*H$_2$Ph), 77.9 (*C*(2)), 123.0 (q, *J* 286, *C*F$_3$), 126.8 (C(2)Ph*C*(2,6)H), 128.2 (OCH$_2$Ph*C*(2,6)H), 128.4 (OCH$_2$Ph*C*(3,5)H), 128.8 (OCH$_2$Ph*C*(4)H), 129.0 (C(2)Ph*C*(3,5)H), 129.6 (C(2)Ph*C*(4)H), 132.7 (C(2)Ph*C*(1)), 133.9 (OCH$_2$Ph*C*(1)), 168.9 (*C*=O); **^{19}F NMR** (376 MHz, $CDCl_3$) δ_F: –76.1 (C*F*$_3$); **HRMS** (NSI$^+$) $C_{16}H_{13}O_3F_3Na^+$ ([M+Na]$^+$) requires 333.0709; found 333.0712 (+0.9 ppm).

Benzyl 2-hydroxy-2-phenylbut-3-enoate 24

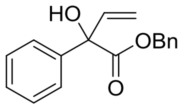

Following general procedure D, benzyl 2-oxo-2-phenylacetate **S11** (1.32 g mg, 5.5 mmol) and vinylmagnesium chloride (4.12 mL, 6 mmol, 1.6 M) in anhydrous THF (25 mL) gave, after column chromatography (Petrol/Et_2O, 9:1; R_F 0.19), benzyl 2-hydroxy-2-phenylbut-3-enoate **24** as a colourless oil (560 mg, 2.09 mmol, 38%); **IR** ν_{max} (film): 3501 (OH), 3304, 1724 (C=O), 1449, 1233, 1150; **^{1}H NMR** (400 MHz, $CDCl_3$) δ_H: 3.86 (1H, s, O*H*), 5.20 (1H, d, *J* 12.3, OC$H_a$$H_b$Ph), 5.26 (1H, d, *J* 12.3, OC$H_a$$H_b$Ph), 5.34 (1H, dd, *J* 10.5, 1.2, RCH=C$H_{cis}$$H_{trans}$), 5.63 (1H, dd, *J* 17.0, 1.2, RCH=C$H_{cis}$$H_{trans}$), 6.44 (1H, dd, *J* 17.0, 10.5, RC*H*=CH_2), 7.22-7.27 (2H, m, OCH_2PhC(3,5)*H*), 7.28-7.37 (6H, m, OCH_2PhC(2,4,6)*H*, C(2)PhC(3,4,5)*H*), 7.48-7.53 (2H, m, C(2)PhC(2,6)*H*); **^{13}C NMR** (100 MHz, $CDCl_3$) δ_C: 68.2 (OCH_2Ph), 78.5 (*C*(2)), 116.0 (C(2)CH=CH_2), 126.1 (C(2)Ph*C*(2,6)H), 128.0 (OCH_2Ph*C*(2,6)H), 128.1 (C(2)Ph*C*(4)H), 128.4 (OCH_2Ph*C*(3,5)H), 128.5 (OCH_2Ph*C*(4)H), 128.6 (C(2)Ph*C*(3,5)H), 134.9 (OCH_2Ph*C*(1)), 137.6 (C(2)*C*H=CH_2), 141.0 (C(2)Ph*C*(1)), 174.0 (*C*=O); **HRMS** (NSI^+) $C_{17}H_{16}O_3Na^+$ ($[M+Na]^+$) requires 291.0992; found 291.0990 (–0.6 ppm).

Benzyl 4-cyclopropyl-2-hydroxy-2-phenybut-3-ynoate 25

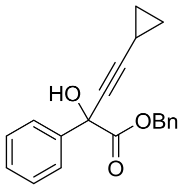

Benzyl 2-oxo-2-phenylacetate **S11** (1.20 g, 5 mmol) was dissolved in THF (25 mL) and cooled to –78 °C. Separately, cyclopropylacetylene (508 µL, 6 mmol) was dissolved in THF (15 mL) and cooled to –78 °C. To the alkyne solution, *n*-BuLi (2.2 mL, 5.5 mmol, 2.5 M) was added dropwise and the solution stirred for 25 mins. The lithiated-alkyne solution was then transferred to the α-keto ester solution at –78 °C, warmed to r.t. and stirred for a further 3 h. On completion, the solution was poured into NH_4Cl (20 mL) and extracted with EtOAc (2 × 30 mL). The organic layers were combined, washed with water (2 × 30 mL) and brine (2 × 30 mL), dried ($MgSO_4$), filtered and concentrated *in vacuo* to give, after column chromatography (eluent Petrol/Et_2O, 9:1; R_F 0.17), benzyl 4-cyclopropyl-2-hydroxy-2-phenybut-3-ynoate **25** as a yellow oil (1.09 g, 3.55 mmol, 71%); **IR** ν_{max} (film): 3487 (OH), 3032, 2237 (C≡C), 1730 (C=O), 1450, 1227, 1067; **^{1}H NMR** (400 MHz, $CDCl_3$) δ_H: 0.73-0.87 (4H, m, cyclopropyl-(CH_2)$_2$), 1.37 (1H, tt, *J* 8.1, 5.1, C*H*), 4.18 (1H, s, O*H*), 5.13 (1H, d, *J* 12.5, OC$H_A$$H_B$Ph), 5.29 (1H, d, *J* 12.5, OC$H_A$$H_B$Ph), 7.16-7.22 (2H, m, OCH_2PhC(2,6)*H*), 7.29-7.40 (6H, m, OCH_2PhC(3,4,5,)*H*, C(2)PhC(3,4,5)*H*), 7.66-7.70 (2H, m, C(2)PhC(2,6)*H*); **^{13}C NMR** (100 MHz, $CDCl_3$) δ_C: –0.34 (cyclopropyl-*C*H), 8.53 (cyclopropyl-(*C*H_2)$_2$), 68.3 (OCH_2Ph), 73.1 (*C*(2)), 73.3 (C(2)*C*≡C), 90.8 (C(2)C≡*C*), 126.4 (C(2)Ph*C*(2,6)H), 127.5 (OCH_2Ph*C*(2,6)H), 128.28 (OCH_2Ph*C*(3,5)H), 128.31 (OCH_2Ph*C*(4)H), 128.5 (C(2)Ph*C*(3,5)H), 128.6 (C(2)Ph*C*(4)H), 135.1 (C(2)Ph*C*(1)), 139.6 (OCH_2Ph*C*(1)), 171.9 (*C*=O); **HRMS** (NSI^+) $C_{20}H_{18}O_3Na^+$ ($[M+Na]^+$) requires 329.1148; found 329.1149 (+0.3 ppm).

Benzyl 2-hydroxy-2,3-diphenylpropanoate S12

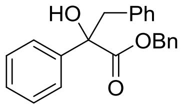

Following general procedure G, *n*-BuLi (8.8 mL, 22 mmol, 2.5 M in hexanes), *i*-Pr_2NH (3.1 mL, 22 mmol), phenylacetic acid (1.36 g, 10 mmol) and benzyl bromide (2.62 mL, 22 mmol) in anhydrous THF (50 mL) gave 2,3-diphenylpropionic acid (2.26 g, ~10 mmol), which was used without further purification. Following general procedure A, the acid (2.26 g, ~10 mmol), oxalyl chloride (1.05 mL, 12 mmol), DMF (1 drop), benzyl alcohol (1.06 mL, 15 mmol) and pyridine (0.81 mL, 10 mmol) in anhydrous CH_2Cl_2 (25 mL) gave, after column chromatography (eluent Petrol/Et_2O, 9:1), the impure ester (2.21 g, ~7 mmol) as a colourless oil. Following general procedure B, the impure ester (2.21 g, ~7 mmol), triethyl phosphite (2.4 mL, 14 mmol), cesium carbonate (494 mg, 1.4 mmol, 20 mol%) in DMSO (28 mL) under a O_2 atmosphere was stirred for 24 h to give, after column chromatography (eluent Petrol/Et_2O, 9:1; R_F 0.13), benzyl 2-hydroxy-2,3-diphenylpropanoate **S12** as a colourless oil (488 mg, 1.47 mmol, 21%); **IR** ν_{max} (film): 3524 (OH), 3032, 1728 (C=O), 1497, 1454, 1260, 1196; **^{1}H NMR** (400 MHz, $CDCl_3$) δ_H: 3.22 (1H, d, *J* 13.6, C(3)H_aH_bPh), 3.61 (1H, d, *J* 13.6, C(3)H_aH_bPh), 3.64 (1H, br s, O*H*), 5.12 (1H, d, *J* 12.2, OCH_aH_bPh), 5.16 (1H, d, *J* 12.2, OCH_aH_bPh), 7.13-7.28 (7H, m, C(2)PhC(3,4,5)*H*, C(3)H_2PhC(3,4,5)*H*, OCH_2PhC(4)*H*), 7.30-7.41 (6H, m, C(3)H_2Ph(2,6)*H*, OCH_2PhC(2,3,5,6)*H*), 7.68-7.73 (2H, m, C(2)PhC(2,6)*H*); **^{13}C NMR** (100 MHz, $CDCl_3$) δ_C: 45.8 (*C*(3)H), 68.1 (O*C*H_2Ph), 78.8 (*C*(2)), 125.8 (C(2)Ph*C*(2,6)H), 126.9 (C(3)H_2Ph*C*(4)H), 127.9 (C(2)Ph*C*(4)H), 128.1 (OCH_2Ph*C*(2,6)H), 128.3 (OCH_2Ph*C*(3,5)H), 128.5 (C(3)H_2Ph*C*(2,6)H), 128.6 (OCH_2Ph*C*(4)H), 128.7 (C(2)Ph*C*(3,5)H), 130.6 (C(3)H_2Ph*C*(3,5)H), 134.8 (OCH_2Ph*C*(1)), 138.6 (C(3)H_2Ph*C*(1)), 141.5 (C(2)Ph*C*(1)), 174.8 (*C*=O); **HRMS** (NSI^+) $C_{22}H_{20}O_3Na^+$ ($[M+Na]^+$) requires 355.1305; found 355.1309 (+1.2 ppm).

Benzyl 2-hydroxy-3,3-dimethyl-2-phenylbutanoate S13

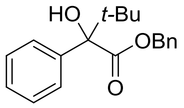

Following general procedure D, benzyl 2-oxo-2-phenylacetate **S11** (1.20 g mg, 5 mmol) and *tert*-butylmagnesium chloride (33 mL, 6 mmol, 0.18 M) in anhydrous THF (25 mL) gave, after column chromatography (Petrol/Et_2O, 9:1; R_F 0.32), benzyl 2-hydroxy-3,3-dimethyl-2-phenylbutanoate **S13** as a colourless oil (471 mg, 1.58 mmol, 32%); **IR** ν_{max} (film): 3509 (OH), 2959, 1713 (C=O), 1447, 1202, 1173; **^{1}H NMR** (400 MHz, $CDCl_3$) δ_H: 1.04 (9H, s, C(CH_3)$_3$), 3.77 (1H, br s, O*H*), 5.32 (2H, s, CH_2), 7.29-7.35 (3H, m, OCH_2PhC(2,6)*H*, C(2)PhC(4)*H*), 7.37-7.45 (5H, m, OCH_2Ph(3,4,5)*H*, C(2)PhC(3,5)*H*), 7.73-7.78 (2H, m, C(2)PhC(2,6)*H*); **^{13}C NMR** (100 MHz, $CDCl_3$) δ_C: 25.8 (C(*C*H_3)$_3$), 39.2 (*C*(CH_3)$_3$), 68.2 (*C*H_2), 83.1 (*C*(2)), 127.3 (C(2)Ph*C*(2,6)H), 127.48 (OCH_2Ph*C*(2,6)H), 127.53 (C(2)Ph*C*(4)H), 128.7 (3C, OCH_2Ph*C*(4)H, C(2)Ph*C*(3,5)H), 128.8 (OCH_2Ph*C*(3,5)H), 134.9 (OCH_2Ph*C*(1)), 139.1 (C(2)Ph*C*(1)), 174.8 (*C*=O); **HRMS** (NSI^+) $C_{19}H_{22}O_3Na^+$ ($[M+Na]^+$) requires 321.1461; found 321.1461 (–0.0 ppm).

Benzyl 2-cyclohexyl-2-hydroxy-2-phenylacetate S14

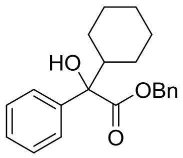

Following general procedure C and D, phenylglyoxylic acid (1.5 g, 10 mmol) cyclohexylMgCl (22 mL, 1 M, 22 mmol) and benzylalcohol (0.85 mL, 8.2 mmol) gave, after column chromatography (Isolera 4, Et_2O in petrol, 0% to 5% over 30 CV; R_F 0.31 (10% Et_2O in petrol)), benzyl 2-cyclohexyl-2-hydroxy-2-phenylacetate **S14** as a yellow oil (718 mg, 2.21 mmol, 30%); **IR** ν_{max} (film): 3512 (O-H), 2930 (C-H), 1721 (C=O), 1447, 1223 (C=C), 1147, 1121 (C-O); **^{1}H NMR** (500 MHz, $CDCl_3$) δ_H: 1.05-1.18 (3H, m, C_6H_{11}), 1.20-1.29 (2H, m, C_6H_{11}), 1.37-1.46 (2H, m, C_6H_{11}), 1.60-1.70 (2H, m, C_6H_{11}), 1.74-1.78 (1H, m, C_6H_{11}), 1.24-1.30 (1H, m, C*H*), 3.72 (1H, s, O*H*), 5.15 (1H, d, *J* 12.3, OCH_aH_bPh), 5.24 (1H, d, *J* 12.3, OCH_aH_bPh), 7.26-7.30 (3H, m, OCH_2PhC(2,6)*H*, C(2)PhC(4)*H*), 7.33-7.38 (5H, m, C(2)PhC(3,5)*H*, OCH_2PhC(3,4,5)*H*), 7.64-7.67 (2H, m, C(2)PhC(2,6)*H*); **^{13}C NMR** (125 MHz, $CDCl_3$) δ_C: 25.6 (C_6H_{11}), 26.3 (C_6H_{11}), 26.4 (C_6H_{11}), 26.4 (C_6H_{11}), 27.4 (C_6H_{11}), 45.7 (*C*H), 68.2 (OCH_2Ph), 81.1 (*C*(2)), 126.2 (C(2)Ph*C*(2,6)H), 127.5 (C(2)Ph*C*(4)H), 128.2 (4 × Ph*C*H), 128.6 (OCH_2Ph*C*(4)H), 128.7 (2 × Ph*C*H), 135.2 (OCH_2Ph*C*(1)), 140.7 (C(2)Ph*C*(1)), 175.7 (*C*=O); **HRMS** (NSI$^+$) $C_{21}H_{24}O_3Na^+$ ([M+Na]$^+$) requires 347.1618; found 347.1608 (−2.8 ppm)

Benzyl 4-cyclopropyl-2-hydroxy-2-methylbut-3-ynoate 26

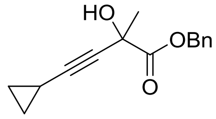

Benzyl pyruvate **S8** (1.02 g, 5 mmol) was dissolved in THF (25 mL) and cooled to –78 °C. Separately, cyclopropylacetylene (508 µL, 6 mmol) was dissolved in THF (15 mL) and cooled to –78 °C. To the alkyne solution, *n*-BuLi (2.2 mL, 5.5 mmol, 2.5 M) was added dropwise and the solution stirred for 25 mins. The lithiated-alkyne solution was then transferred to the pyruvate solution at –78 °C, warmed to r.t. and stirred for a further 3 h. On completion, the solution was poured into NH_4Cl (20 mL) and extracted with EtOAc (2 × 30 mL). The organic layers were combined, washed with water (2 × 30 mL) and brine (2 × 30 mL), dried ($MgSO_4$), filtered and concentrated *in vacuo* to give, after column chromatography (eluent Petrol/Et_2O, 9:1; R_F 0.14), benzyl 4-cyclopropyl-2-hydroxy-2-methylbut-3-ynoate **26** as a yellow oil (294 mg, 1.21 mmol, 24%); **IR** ν_{max} (film): 3466 (OH), 3009, 2245 (C≡C), 1736 (C=O), 1454, 1238, 1132; **^{1}H NMR** (500 MHz, $CDCl_3$) δ_H: 0.63-0.67 (2H, m, cyclopropyl-CH_2), 0.73-0.79 (2H, m, cyclopropyl-CH_2), 1.20 (1H, m, C*H*), 1.65 (3H, s, CH_3), 3.39 (1H, s, O*H*), 5.23 (1H, d, *J* 12.4, OCH_aH_bPh), 5.29 (1H, d, *J* 12.4, OCH_aH_bPh), 7.32-7.41 (5H, m, PhC*H*); **^{13}C NMR** (100 MHz, $CDCl_3$) δ_C: –0.63 (*C*H), 8.25 (cyclopropyl-(CH_2)$_2$), 27.2 (*C*H_3), 68.0 (OCH_2Ph), 68.1 (*C*(2)), 74.8 (C(2)*C*≡C), 88.3 (C(2)C≡*C*), 127.9 (Ph*C*(2,6)H), 128.5 (Ph*C*(4)H), 128.6 (Ph*C*(3,5)H), 135.2 (Ph*C*(1)), 172.8 (*C*=O); **HRMS** (NSI$^+$) $C_{15}H_{20}NO_3^+$ ([M+NH$_4$]$^+$) requires 262.1438; found 262.1441 (+1.3 ppm).

Benzyl 2-hydroxy-2-methylbut-3-enoate 27

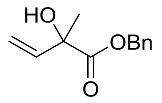

Pyruvic acid (1.1 mL, 15 mmol) and triethylamine (2.1 mL, 15 mmol) were dissolved in CH_2Cl_2 (anhydrous, 150 mL). Benzyl chloroformate (2.1 mL, 15 mmol) was added dropwise and the resulting brown solution was allowed to stir for 1 h. The solution was diluted with Et_2O, washed with HCl (1 M), $NaHCO_3$ (sat. aq.) and brine. The organic layer was dried over $MgSO_4$, filtered and concentrated *in vacuo*, the crude benzyl pyruvate (15 mmol max) was used without further purification. To a solution of benzyl pyruvate in anhydrous Et_2O (30 mL) was added vinylMgBr (13.2 mL, 1 M in THF, 13.2 mmol) dropwise at −25 °C. After stirring for 5.5 h, the reaction was acidified with HCl (1 M) to pH 4 and extracted with EtOAc. The organic phases were combined, washed with brine, dried over $MgSO_4$, filtered and concentrated *in vacuo*. The crude was purified by column chromatography (Isolera 4, Et_2O in petrol, 0% to 20% over 30 CV; R_F 0.28) to give benzyl 2-hydroxy-2-methylbut-3-enoate **27** as a yellow oil (299 mg, 1.45 mmol, 10%); **IR** ν_{max} (film): 3509 (O-H), 2982 (C-H), 1728 (C=O), 1456 (C=C), 1260 (C=C), 1186, 1119 (C-O); **^{1}H NMR** (500 MHz, $CDCl_3$) δ_H: 1.59 (3H, s, C*H*$_3$), 3.38 (1H, s, O*H*), 5.17-5.21 (2H, m, CH=C$H_{trans}H_{cis}$, OCH_aH_bPh), 5.23 (1H, d, *J* 12.3, OCH_aH_bPh), 5.48 (1H, dd, *J* 1.1, 17.2, CH=C$H_{trans}H_{cis}$), 6.04 (1H, dd, *J* 12.1, 17.2, C*H*=CH$_2$), 7.32-7.41 (m, 5H, PhC*H*); **^{13}C NMR** (125 MHz, $CDCl_3$) δ_C: 25.8 (*C*H$_3$), 67.8 (*C*H$_2$), 74.9 (*C*(2)), 114.8 (CH=*C*H$_2$) 128.1 (2 × Ph*C*H), 128.6 (Ph*C*(4)H), 128.7 (2 × Ph*C*H), 135.3 (Ph*C*(1)), 139.5 (*C*H=CH$_2$), 175.4 (*C*=O); **HRMS** (ESI$^+$) $C_{12}H_{14}O_3Na^+$ ([M+Na]$^+$) requires 229.0835; found 229.0830 (−2.3 ppm). Data in agreement with literature.[13]

Benzyl 2-hydroxy-2,4-dimethylpent-3-enoate 28

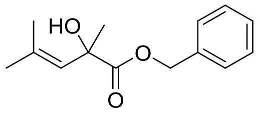

Following general procedure D, pyruvic acid (0.7 mL, 10 mmol) and 2-methyl-1-propenylMgBr (44 mL, 0.5 M in THF, 22 mmol) were reacted in anhydrous Et_2O (25 mL) to give impure 2-hydroxy-2,4-dimethylpent-3-enoic acid (721 mg, ~5 mmol). Following the procedure outlined by Kobayashi,[14] 2-hydroxy-2,4-dimethylpent-3-enoic acid (721 mg, ~5 mmol), $NaHCO_3$ (420 mg, 5 mmol), benzyl bromide (0.65 mL, 941 mg, 5.5 mmol) and tetrabutylammonium iodide (1.77 g, 5.5 mmol) were stirred in a mixture of CH_2Cl_2 (10 mL) and H_2O (10 mL) for 3 days at r.t. Half of the solvent was removed *in vacuo*, the rest was extracted with Et_2O, combined organic layer was dried over dried over $MgSO_4$, filtered and concentrated *in vacuo*. The crude was purified by column chromatography (Isolera 4, Et_2O in petrol, 0% to 20% over 30 CV; R_F 0.15) to give benzyl 2-hydroxy-2,4-dimethylpent-3-enoate **28** as a pale yellow oil (207 mg, 0.88 mmol, 18%); **IR** ν_{max} (film): 3491 (O-H), 2976 (C-H), 1724 (C=O), 1454 (C=C), 1115 (C=C); **^{1}H NMR** (500 MHz, $CDCl_3$) δ_H: 1.53 (3H, s, C(2)C*H*$_3$), 1.64 (3H, d, *J* 1.0, CH=C(C*H*$_3$)$_2$), 1.71 (3H, d, *J* 1.0, CH=C(C*H*$_3$)$_2$), 3.24-3.28 (1H, m, O*H*), 5.21 (2H, s, C*H*$_2$), 5.35-5.36 (1H, m, C*H*), 7.32-7.39 (5H, m, PhC*H*); **^{13}C NMR** (125 MHz, $CDCl_3$) δ_C: 18.7 (CH=C(*C*H$_3$)$_2$), 26.9 (CH=C(*C*H$_3$)$_2$), 28.5 (C(2)*C*H$_3$), 67.6 (*C*H$_2$), 73.3 (*C*(2)), 126.2 (*C*H=C(CH$_3$)$_2$), 128.2 (2 × Ph*C*H), 128.5 (Ph*C*(4)H), 128.7 (2 × Ph*C*H), 135.6 (Ph*C*(1)), 138.8 (CH=*C*(CH$_3$)$_2$), 177.0 (*C*=O); **HRMS** (NSI$^+$) $C_{14}H_{22}NO_3^+$ ([M+NH$_4$]$^+$) requires 252.1594; found 252.1599 (+1.9 ppm). Data in agreement with literature.[15]

Benzyl 2-hydroxy-2,3-dimethylbut-3-enoate 29

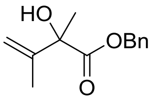

Pyruvic acid (1.1 mL, 15 mmol) and triethylamine (2.1 mL, 15 mmol) were dissolved in anh. CH_2Cl_2 (150 mL). Benzyl chloroformate (2.1 mL, 15 mmol) was added dropwise and the resulting brown solution was allowed to stir for 1 h. The solution was diluted with Et_2O, washed with HCl (1 M), $NaHCO_3$ (sat. aq.) and brine. The organic layer was dried over $MgSO_4$, filtered and concentrated *in vacuo*, the crude benzyl pyruvate was used without further purification. To a solution of benzyl pyruvate **S8** (~7 mmol) in anhydrous Et_2O (30 mL) was added isopropenylMgBr (26.4 mL, 0.5 M in THF, 13.2 mmol) dropwise at −25 °C. After stirring for 5.5 h, the reaction was acidified with HCl (1 M) to pH 4 and extracted with EtOAc. The organic phases were combined, washed with brine, dried over $MgSO_4$, filtered and concentrated *in vacuo*, The crude was purified by column chromatography (Isolera 4, Et_2O in petrol, 0% to 10% over 30 CV; R_F 0.33) to give benzyl 2-hydroxy-2,3-dimethylbut-3-enoate **29** as a yellow oil (336 mg, 1.53 mmol, 10%); **IR** ν_{max} (film): 3510 (O-H), 2982 (C-H), 1724 (C=O), 1454 (C=C), 1225 (C=C), 1144, 1113 (C-O); **^{1}H NMR** (500 MHz, $CDCl_3$) δ_H: 1.57 (3H, s, C(2)C*H*$_3$), 1.75 (3H, s, C(3)C*H$_3$*), 3.46 (1H, s, O*H*), 4.97 (1H, s, =C*H$_a$*H$_b$), 5.15 (1H, s, =CH$_a$*H$_b$*), 5.13 (2H, s, C*H*$_2$), 7.32-7.40 (m, 5H, PhC*H*); **^{13}C NMR** (125 MHz, $CDCl_3$) δ_C: 18.7 (C(3)CH$_3$), 23.9 (C(2)*C*H$_3$), 67.7 (*C*H$_2$), 76.8 (*C*(2)), 112.5 (=*C*H$_2$), 128.2 (2 × Ph*C*H), 128.5 (Ph*C*(4)H), 128.7 (2 × Ph*C*H), 135.5 (Ph*C*(1)), 145.7 (*C*(3)), 175.5 (*C*=O); **HRMS** (ESI$^+$) $C_{13}H_{16}O_3Na^+$ ($[M+Na]^+$) requires 243.0992; found 243.0986 (−2.3 ppm).

2-Hydroxy-2,3-dimethylbut-3-enoic acid S15

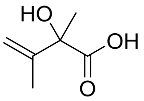

Benzyl 2-hydroxy-2,3-dimethylbut-3-enoate **29** (25 mg, 0.12 mmol) heated at 85 °C in a mixture of NaOH (2 M):THF (1:1) for 24hrs. The resulted mixture was neutralized with HCl (1 M) and extracted with EtOAc. The organic layer was dried over $MgSO_4$ and concentrated *in vacuo* to give 2-hydroxy-2,3-dimethylbut-3-enoic acid **S15** (20.3 mg, 0.1 mmol, 83%); **IR** ν_{max} (film): 3447 (O-H, alcohol), 2926 (O-H, acid), 1716 (C=O), 1456 (C=C), 1246, 1140 (C-O); **^{1}H NMR** (500 MHz, $CDCl_3$) δ_H: 1.62 (3H, s, C(2)C*H*$_3$), 1.84 (3H, s, C(3)C*H$_3$*), 3.36 (1H, br, -O*H*), 5.03 (1H, s, =C*H$_a$*H$_b$), 5.22 (1H, s, =CH$_a$*H$_b$*),;Data in agreement with literature.[16]

Benzyl 2-cyclopentyl-2-hydroxypropanoate 30

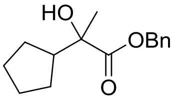

Following general procedure C, benzyl pyruvate **S8** (1.11 g, 6.25 mmol) and cyclopentylMgCl (6.9 mL, 2 M in THF, 13.8 mmol) in anhydrous Et_2O (30 mL) gave, after column chromatography (Isolera 4, Et_2O in petrol, 0% to 10% over 30 CV; R_F 0.18), benzyl 2-cyclopentyl-2-hydroxypropanoate **30** as a yellow oil (258 mg, 1.04 mmol, 17%); **IR** ν_{max} (film):

3528 (O-H), 2951 (C-H), 2868 (C-H), 1724 (C=O), 1454 (C=C), 1184 (C-O); **^{1}H NMR** (500 MHz, $CDCl_3$) δ_H: 1.27-1.39 (2H, m, C_5H_9), 1.41 (3H, s, CH_3), 1.43-1.75 (6H, m, C_5H_9), 2.19-2.26 (1H, m, C*H*), 3.14 (1H, s, O*H*), 5.18 (1H, d, *J* 12.3, OCH_aH_bPh), 5.21 (1H, d, *J* 12.3, OCH_aH_bPh), 7.30-7.40 (m, 5H, PhC*H*); **^{13}C NMR** (125 MHz, $CDCl_3$) δ_C: 24.8 (CH_3), 25.8 (C_5H_9), 25.9 (C_5H_9), 26.1 (C_5H_9), 26.8 (C_5H_9), 47.5 (*C*H), 67.5 (OCH_2Ph), 75.5 (*C*(2)), 128.3 (Ph*C*(2,6)H), 128.6 (Ph*C*(4)H), 128.7 (Ph*C*(3,5)H), 135.5 (Ph*C*(1)), 177.6 (*C*=O); **HRMS** (ESI^+) $C_{15}H_{20}O_3Na^+$ ($[M+Na]^+$) requires 271.1305; found 271.1302 (−0.9 ppm).

Benzyl 2-cyclohexyl-2-hydroxypropanoate 31

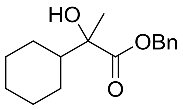

Following general procedure C, benzyl pyruvate **S8** (1.11 g, 6.25 mmol) and cyclohexylMgCl (13.8 mL, 1 M in 2-methyltetrahydrofuran, 13.8 mmol) in anhydrous Et_2O (30 mL) gave, after column chromatography (Isolera 4, Et_2O in petrol, 0% to 10% over 30 CV; R_F 0.15), benzyl 2-cyclohexyl-2-hydroxypropanoate **31** as a yellow oil (423 mg, 1.6 mmol, 26%); **IR** ν_{max} (film): 3524 (O-H), 2928 (C-H), 2853 (C-H), 1724 (C=O), 1452 (C=C), 1152 (C-O); **^{1}H NMR** (500 MHz, $CDCl_3$) δ_H: 1.03-1.23 (5H, m, C_6H_{11}), 1.31-1.33 (1H, m, C_6H_{11}), 1.37 (3H, s, CH_3), 1.59-1.64 (2H, m, C_6H_{11}), 1.68-1.70 (1H, m, C_6H_{11}), 1.75-1.82 (2H, m, C_6H_{11})), 3.05 (1H, s, O*H*), 5.20 (2H, s, OCH_2Ph), 7.34-7.39 (m, 5H, PhC*H*); **^{13}C NMR** (125 MHz, $CDCl_3$) δ_C: 23.3 (CH_3), 25.8 (C_6H_{11}), 26.3 (C_6H_{11}), 26.4 (C_6H_{11}), 26.4 (C_6H_{11}), 27.4 (C_6H_{11}), 45.7 (*C*H), 67.5 (OCH_2Ph), 77.0 (*C*(2)), 128.3 (Ph*C*(2,6)H), 128.6 (Ph*C*(4)H), 128.7 (Ph*C*(3,5)H), 135.5 (Ph*C*(1)), 177.5 (*C*=O); **HRMS** (ESI^+) $C_{16}H_{22}O_3Na^+$ ($[M+Na]^+$) requires 285.1461; found 285.1457 (−1.3 ppm). Data in agreement with literature.[17]

Benzyl 2-hydroxy-2-(4-methoxyphenyl)-2-phenylacetate S16

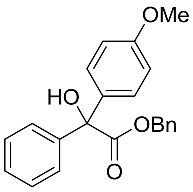

Following general procedure C and D, 4-bromoanisole (0.45 mL, 3.6 mmol), magnesium turnings (109 mg, 4.5 mmol) and benzyl pyruvate **S8** (721 mg, 3 mmol) in anhydrous THF (30 mL) gave, after column chromatography (Isolera 4, CH_2Cl_2 in toluene, 0% to 90% over 30 CV; R_F 0.26), benzyl 2-hydroxy-2-(4-methoxyphenyl)-2-phenylacetate **S16** as a yellow oil (433 mg, 1.2 mmol, 41%); **IR** ν_{max} (film): 3487 (O-H), 2957 (C-H), 1724 (C=O), 1609, 1508, 1248 (C=C), 1163 (C-O); **^{1}H NMR** (500 MHz, $CDCl_3$) δ_H: 3.81 (3H, s, OCH_3), 4.22 (1H, s, O*H*), 5.30 (2H, s, CH_2), 6.84-6.87 (2H, m, C(2)ArC(3',5')*H*), 7.22-7.24 (2H, m, OCH_2PhC(2,6)*H*), 7.32-7.36 (8H, m, C(2)PhC(3,4,5)*H*, OCH_2PhC(3,4,5)*H*, C(2)ArC(2',6')*H*), 7.42-7.45 (2H, m, C(2)PhC(2,6)*H*); **^{13}C NMR** (125 MHz, $CDCl_3$) δ_C: 55.3 (OCH_3), 68.4 (CH_2), 80.9 (*C*(2)), 113.5 (C(2)Ar*C*(3',5')H), 127.5 (C(2)Ph*C*(2,6)H), 128.1 (C(2)Ph*C*(4)H), 128.1 (2 × Ar*C*H), 128.2 (2 × Ar*C*H), 128.6 (OCH_2Ph*C*(4)H), 128.6 (2 × Ar*C*H), 128.8 (2 × Ar*C*H), 134.1 (C(2)Ar*C*(1')), 134.9 (OCH_2Ph*C*(1)), 142.1 (C(2)Ph*C*(1)), 159.4 (C(2)Ar*C*(4')), 174.5 (C=O); **HRMS** (ESI^+) $C_{22}H_{20}NaO_4^+$ ($[M+Na]^+$) requires 371.1254; found 371.1241 (–3.5 ppm). Data in agreement with literature.[18]

2-Phenylpropane-1,2-diol S17

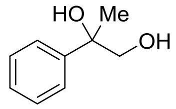

A solution of 2-hydroxy-2-phenylpropanoic acid **S1** (500 mg, 3 mmol) in anh. Et_2O (5 mL) was added dropwise to a suspension of $LiAlH_4$ (2.4 M in THF, 2.5 mL, 6 mmol) in anh. Et_2O (10 mL) under N_2 at 0 °C. The reaction mixture was allowed to stir at r.t. overnight. The reaction was quenched by the addition 2M HCl and the mixture was extracted with Et_2O (2 × 20 mL). The organic layers were combined, and washed with sat. aq. $NaHCO_3$ solution (2 × 20 mL), brine (2 × 20 mL), dried over $MgSO_4$, filtered and concentrated *in vacuo* to obtain 2-phenylpropane-1,2-diol **S17** (403 mg, 2.6 mmol, 88%) as a yellow oil; **IR** ν_{max} (film): 3354, 1495, 1447, 1041, 1026; **^{1}H NMR** (400 MHz, $CDCl_3$) δ_H: 1.54 (3H, s, CH_3), 3.65 (1H, d, *J* 11.1, C$H_a$$H_b$OH), 3.81 (1H, d, *J* 11.1, C$H_a$$H_b$OH), 7.27-7.31 (1H, m, PhC*H*), 7.35-7.40 (2H, m, PhC*H*), 7.44-7.49 (2H, m, PhC*H*). Data in agreement with literature.[19]

1-(Benzyloxy)-2-phenylpropan-2-ol 33

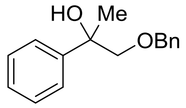

Following the method by McCarthy *et al*,[20] to a solution 2-phenylpropane-1,2-diol **S17** (350 mg, 2.3 mmol) in DMF (7 mL) under N_2 was added Cs_2CO_3 (820 mg, 2.5 mmol) and benzyl bromide (0.3 mL, 2.5 mmol). The reaction was stirred overnight under N_2 at r.t. The reaction was then quenched by water (10 mL) and acidified with 2M HCl to pH 5. The mixture was extracted with EtOAc and the organic layer was washed with water (3 × 10 mL), dried over $MgSO_4$, filtered and concentrated *in vacuo* to give the crude product, which was purified by column chromatography (Isolera 4, Et_2O in petrol, 10% to 70% over 35CV) to give 1-(benzyloxy)-2-phenylpropan-2-ol **33** as a colourless oil (200 mg, 0.83 mmol, 36%); **IR** ν_{max} (film): 3421, 1495, 1447, 1092, 1067; **^{1}H NMR** (400 MHz, $CDCl_3$) δ_H: 1.53 (3H, s, CH_3), 3.56 (1H, d, *J* 9.2, C$H_a$$H_b$OBn), 3.63 (1H, d, *J* 9.2, C$H_a$$H_b$OBn), 4.55 (2H, s, OC$H_2$Ph), 7.23-7.28 (4H, m, PhC*H*), 7.29-7.37 (4H, m, PhC*H*), 7.43-7.47 (2H, m, PhC*H*). Data in agreement with literature.[21]

3-Hydroxy-3-phenylbutanoic acid S18

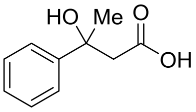

Following the method by Oestreich *et al*,[22] Zinc dust (1.96 g, 30 mmol), $CeCl_3{\cdot}7H_2O$ (168 mg, 0.45 mmol) and acetophenone (1.8 mL, 15 mmol) were added to anhydrous THF (50 mL) under N_2. The mixture was cooled to 0 °C and stirred for 10 min. Ethyl bromoacetate (2.5 mL, 22.5 mmol) was added dropwise and the reaction mixture was stirred at r.t. overnight. The reaction was quenched by the addition of sat. aq. NH_4Cl solution. The mixture was filtered through a short pad of Celite and washed with ethyl acetate. The aqueous layer of

the filtrate was extracted with EtOAc (2 × 20 mL) and the combined organic layers were washed with water (2 × 20 mL), dried over $MgSO_4$, filtered and concentrated *in vacuo*. The corresponding β-hydroxy ester was obtained and directly subjected to the next step. The crude β-hydroxy ester was dissolved in methanol (20 mL) and KOH (2.1 g, 37.5 mmol), and the mixture stirred for 5 h at 40 °C. Water was added, and the majority of the methanol was removed *in vacuo*. The aqueous residue was acidified with 2M HCl and extracted with CH_2Cl_2 (3 × 30 mL). The combined organic extracts were dried over $MgSO_4$, filtered and concentrated *in vacuo* to give 3-hydroxy-3-phenylbutanoic acid **S18** (2.25 g, 13 mmol, 83%) as a yellow oil; **IR** ν_{max} (film): 2978, 1701 (C=O), 1446, 1379, 1180; **^{1}H NMR** (400 MHz, $CDCl_3$) δ_H: 1.58 (3H, s, C*H*$_3$), 2.85 (1H, d, *J* 16.3, C(2)*H*$_a$H$_b$), 3.04 (1H, d, *J* 16.3, C(2)H$_a$*H*$_b$), 7.26-7.28 (1H, m, PhC*H*), 7.32-7.38 (2H, m, PhC*H*), 7.41-7.47 (2H, m, PhC*H*). Data in agreement with literature.[23]

Benzyl 3-hydroxy-3-phenylbutanoate 34

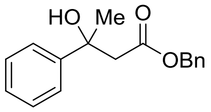

Following the method by McCarthy *et al*,[24] to a solution of 3-hydroxy-3-phenylbutanoic acid **S18** (2 g, 11.1 mmol) in DMF (50 mL) under N_2 was added Cs_2CO_3 (5.4 g, 16.7 mmol) and benzyl bromide (2 mL, 16.7 mmol). The reaction was stirred overnight under N_2 at r.t. The reaction was quenched by the addition water (50 mL) and acidified with 2M HCl to pH 5. The mixture was extracted by EtOAc and the organic layer was washed with water (3 × 30 mL), dried over $MgSO_4$, filtered and concentrated *in vacuo* to give the crude product, which was purified by column chromatography (Isolera 4, Et_2O in petrol, 0% to 20% over 25CV) to give benzyl 3-hydroxy-3-phenylbutanoate **34** as a colourless oil (1.90 g, 7.0 mmol, 64%); **IR** ν_{max} (film): 3509, 1712 (C=O), 1495, 1447, 1383, 1329, 1173; **^{1}H NMR** (400 MHz, $CDCl_3$) δ_H: 1.54 (3H, s, C*H*$_3$), 2.85 (1H, d, *J* 15.9, C(2)*H*$_a$H$_b$), 3.05 (1H, d, *J* 15.9, C(2)H$_a$*H*$_b$), 5.03 (2H, s, OCH_2Ph), 7.14-7.18 (2H, m, PhC*H*), 7.22-7.25 (1H, m, PhC*H*), 7.35-7.28 (5H, m, PhC*H*), 7.42-7.46 (2H,m, PhC*H*). Data in agreement with literature.[25]

Preparation of Racemic Esters

1-Oxo-2-phenyl-1-(phenylamino)propan-2-yl isobutyrate S19

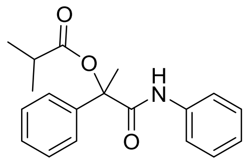

Following general procedure F, 2-hydroxy-*N*,2-diphenylpropanamide **3** (39 mg, 0.16 mmol), isobutyric anhydride (106 µL, 0.64 mmol), DMAP (2 mg, 0.016 mmol, 10 mol%) and NEt_3 (133 µL, 0.96 mmol) were reacted in CH_2Cl_2 (1 mL) to give the crude product, which was purified by column chromatography (Isolera 4, EtOAc in petrol, 0% to 15% over 30 CV; R_F 0.38) to give 1-oxo-2-phenyl-1-(phenylamino)propan-2-yl isobutyrate **S19** as a colourless solid (34 mg, 0.11 mmol, 69%); **mp** 103-105 °C; **IR** ν_{max} (film): 3335 (N-H), 2974 (C-H), 1746 (C=O), 1678 (C=O), 1526 (N-H), 1441 (C=C), 1244 (C-N), 1130 (C-O); **^{1}H NMR** (500 MHz, $CDCl_3$) δ_H: 1.29 (6H, d, *J* 7.0, CH(C*H*$_3$)$_2$), 2.09 (3H, s, C(2)C*H*$_3$), 2.75 (1H, hept, *J* 7.0, C*H*(CH$_3$)$_2$), 7.09-7.12 (1H, m, NHPhC(4)*H*), 7.29-7.34 (3H, m, NHPhC(3,5)*H*, C(2)PhC(4)*H*), 7.36-7.40 (2H, m, C(2)PhC(3,5)*H*), 7.47-7.51 (4H, m, NHPhC(2,6)*H*, C(2)PhC(2,6)*H*), 7.78 (1H, s, N*H*); **^{13}C NMR** (125 MHz, $CDCl_3$) δ_C: 19.1 (2C, CH(*C*H$_3$)$_2$), 23.5 (C(2)*C*H$_3$), 34.9 (*C*H(CH$_3$)$_2$), 84.2 (*C*(2)), 119.9 (NHPh*C*(2,6)H), 124.7 (NHPh*C*(4)H), 127.8 (C(2)Ph*C*(2,6)H), 128.4 (C(2)Ph*C*(4)H), 128.9 (C(2)Ph*C*(3,5)H), 129.2 (NHPh*C*(3,5)H), 137.4 (NHPh*C*(1)), 140.5 (C(2)Ph*C*(1)), 169.3 (*C*(1)=O), 174.5 (*C*(O)*i*-Pr); **HRMS** (NSI$^+$) $C_{19}H_{22}NO_3^+$ ([M+H]$^+$) requires 312.1594; found 312.1598 (+1.2 ppm).

1-(Benzylamino)-1-oxo-2-phenylpropan-2-yl isobutyrate S20

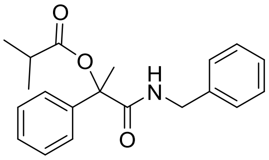

Following general procedure F, *N*-benzyl-2-hydroxy-2-phenylpropanamide **S3** (41 mg, 0.16 mmol), isobutyric anhydride (106 µL, 0.64 mmol), DMAP (2 mg, 0.016 mmol, 10 mol%) and NEt_3 (133 µL, 0.96 mmol) were reacted in CH_2Cl_2 (1 mL) to give the crude product, which was purified by column chromatography (Isolera 4, EtOAc in petrol, 0% to 25% over 25 CV) to give 1-(benzylamino)-1-oxo-2-phenylpropan-2-yl isobutyrate **S20** as a colourless solid (31 mg, 0.10 mmol, 60%), **mp** 88-90 °C; **IR** ν_{max} (film): 3337 (N-H), 2974 (C-H), 1742 (C=O), 1663 (C=O), 1526, 1449 (C=C), 1150 (C-O); **^{1}H NMR** (500 MHz, $CDCl_3$) δ_H: 1.17 (3H, d, *J* 7.0, CH(C*H*$_3$)(CH$_3$)), 1.20 (3H, d, *J* 7.0, CH(CH$_3$)(C*H*$_3$)), 2.04 (3H, s, C(2)C*H*$_3$), 2.64 (1H, app. hept, *J* 7.0, C*H*(CH$_3$)$_2$), 4.36 (1H, dd, *J* 14.9, 5.4, NHC*H*$_a$H$_b$Ph), 4.53 (1H, dd, *J* 14.9, 6.2 NHCH$_a$*H*$_b$Ph), 6.22-6.25 (1H, m, N*H*), 7.15-7.18 (2H, m, NHCH$_2$PhC(2,6)*H*), 7.24-7.27 (1H, m, NHCH$_2$ArC(4)*H*), 7.28-7.33 (3H, m, NHCH$_2$PhC(3,5)*H*, C(2)PhC(4)*H*), 7.35-7.38 (2H, m, C(2)PhC(3,5)*H*), 7.44-7.46 (2H, m, C(2)PhC(2,6)*H*); **^{13}C NMR** (125 MHz, $CDCl_3$) δ_C: 18.9 (CH(*C*H$_3$)(CH$_3$)), 18.1 (CH(CH$_3$)(*C*H$_3$)) 23.4 (C(2)*C*H$_3$), 34.7 (*C*H(CH$_3$)$_2$), 40.7 (*C*H$_2$Ph), 83.8 (*C*(2)), 124.7 (C(2)Ph*C*(2,6)H), 127.7 (NHCH$_2$Ph*C*(4)H), 127.8 (NHCH$_2$Ph*C*(2,6)H), 128.2 (C(2)Ph*C*(4)H), 128.7 (NHCH$_2$Ph*C*(3,5)H), 128.8 (C(2)Ph*C*(3,5)H), 138.1 (NHCH$_2$Ph*C*(1)), 141.0 (128.2 (C(2)Ph*C*(1)), 171.3 (*C*(1)=O), 174.6 (C(O)*i*-Pr); **HRMS** (NSI$^+$) $C_{20}H_{24}NO_3^+$ ([M+H]$^+$) requires 326.1751; found 326.1756 (+1.6 ppm).

1-(Isopropylamino)-1-oxo-2-phenylpropan-2-yl isobutyrate S21

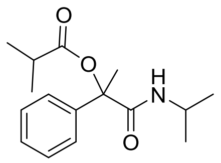

Following general procedure F, 2-hydroxy-*N*-isopropyl-2-phenylpropanamide **S4** (33 mg, 0.16 mmol), isobutyric anhydride (130 µL, 0.8 mmol), DMAP (2 mg, 0.016 mmol, 10 mol%) and TMP (270 µL, 1.60 mmol) were reacted in CH_2Cl_2 (1 mL) to give the crude product, which was purified by column chromatography (Isolera 4, EtOAc in petrol, 0% to 25% over 30 CV; R_F 0.48 (30% EtOAc in petrol)) to give 1-(isopropylamino)-1-oxo-2-phenylpropan-2-yl isobutyrate **S21** as a colourless solid (22 mg, 0.08 mmol, 50%); **mp** 132-134 °C; **IR** ν_{max} (film): 3281 (N-H), 2974 (C-H), 1742 (C=O), 1651 (C=O), 1533, 1368 (C=C), 1192 (C-N), 1146 (C-O); **^{1}H NMR** (500 MHz, $CDCl_3$) δ_H: 1.08 (3H, d, *J* 6.6, NHCH(C*H*$_3$)$_2$), 1.14 (3H, d, *J* 6.6, NHCH(C*H*$_3$)$_2$), 1.24 (6H, d, *J* 7.0, C(O)CH(CH$_3$)(C*H*$_3$)), 1.98 (3H, s, C(2)C*H*$_3$), 2.67 (1H, app. hept, *J* 7.0, C(O)C*H*(CH$_3$)$_2$), 4.01-4.10 (1H, m, NC*H*(CH$_3$)$_2$), 5.79 (1H, d, *J* 8.1, N*H*), 7.27-7.31 (1H, m, PhC(4)*H*), 7.33-7.37 (2H, m, PhC(3,5)*H*), 7.41-7.43 (2H, m, PhC(2,6)*H*); **^{13}C NMR** (125 MHz, $CDCl_3$) δ_C: 19.1 (2C, C(O)CH(*C*H$_3$)$_2$), 22.6 (NCH(*C*H$_3$)(CH$_3$)), 22.8 (-NCH(CH$_3$)(*C*H$_3$)), 23.4 (C(2)*C*H$_3$), 34.8 (C(O)*C*H(CH$_3$)$_2$), 41.6 (N*C*H(CH$_3$)$_2$), 83.7 (*C*(2)), 124.7 (Ph*C*(2,6)H), 128.0 (Ph*C*(4)H), 128.4 (Ph*C*(3,5)H), 141.2 (Ph*C*(1)), 170.4 (*C*(1)=O), 174.5 (*C*(O)*i*-Pr); **HRMS** (ESI$^+$) $C_{16}H_{23}NNaO_3^+$ ([M+Na]$^+$) requires 300.1570; found 300.1559 (–3.7 ppm).

1-(Cyclopropylamino)-1-oxo-2-phenylpropan-2-yl isobutyrate S22

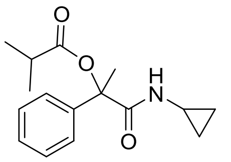

Following general procedure F, *N*-cyclopropyl-2-hydroxy-2-phenylpropanamide **S5** (33 mg, 0.16 mmol), isobutyric anhydride (130 µL, 0.8 mmol), DMAP (2 mg, 0.016 mmol, 10 mol%) and TMP (270 µL, 1.60 mmol) were reacted in CH_2Cl_2 (1 mL) to give the crude product, which was purified by column chromatography (Isolera 4, EtOAc in petrol, 0% to 30% over 30 CV; R_F 0.30) to give 1-(cyclopropylamino)-1-oxo-2-phenylpropan-2-yl isobutyrate **S22** as a colourless solid (30 mg, 0.11 mmol, 69%); **mp** 114-116 °C; **IR** ν_{max} (film): 3310 (N-H), 2974 (C-H), 1744 (C=O), 1661 (C=O), 1516, 1151 (C-O); **^{1}H NMR** (500 MHz, $CDCl_3$) δ_H: 0.34-0.39 (1H, m, cyclopropyl-C*H*$_2$), 0.47-0.52 (1H, m, cyclopropyl-C*H*$_2$), 0.70-0.80 (2H, m, cyclopropyl-C*H*$_2$), 1.22 (6H, 2 x d, *J* 7.1, CH(C*H*$_3$)$_2$), 1.97 (3H, s, C(2)C*H*$_3$), 2.61-2.80 (2H, m, C*H*(CH$_3$)$_2$, NC*H*(CH$_2$)$_2$), 6.03 (1H, s, N*H*), 7.27-7.31 (1H, m, PhC(4)*H*), 7.34-7.37 (2H, m, PhC(3,5)*H*), 7.41-7.42 (2H, m, PhC(2,6)*H*); **^{13}C NMR** (125 MHz, $CDCl_3$) δ_C: 6.75 (cyclopropyl-CH$_2$), 6.89 (cyclopropyl-*C*H$_2$), 19.1 (2C, CH(*C*H$_3$)$_2$), 22.7 (NH*C*H), 23.5 (C(2)*C*H$_3$), 34.7 (C(O)*C*H), 124.6 (Ph*C*(2,6)H), 128,1 (Ph*C*(4)H), 128.7 (Ph*C*(3,5)H), 141.0 (Ph*C*(1)), 172.7 (*C*(1)=O), 172.6 (*C*(O)*i*-Pr); **HRMS** (ESI$^+$) $C_{16}H_{21}NNaO_3^+$ ([M+Na]$^+$) requires 298.1414; found 298.1411 (–0.9 ppm).

1-(Cyclohexylamino)-1-oxo-2-phenylpropan-2-yl isobutyrate S23

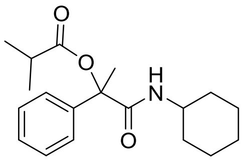

Following general procedure F, *N*-cyclohexyl-2-hydroxy-2-phenylpropanamide **S6** (40 mg, 0.16 mmol), isobutyric anhydride (106 µL, 0.64 mmol), DMAP (2 mg, 0.016 mmol, 10 mol%) and NEt_3 (133 µL, 0.96 mmol) were reacted in CH_2Cl_2 (1 mL) to give the crude product, which was purified by column chromatography (Isolera 4, EtOAc in petrol, 0% to 15% over 30 CV; R_F 0.45 (20% EtOAc in petrol)) to give 1-(cyclohexylamino)-1-oxo-2-phenylpropan-2-yl isobutyrate **S23** as a colourless solid (11 mg, 0.03 mmol, 21%); **mp** 102-104 °C; **IR** ν_{max} (film): 3341 (N-H), 2931 (C-H), 1744 (C=O), 1659 (C=O), 1524, 1150 (C-O); **^{1}H NMR** (500 MHz, $CDCl_3$) δ_H: 1.03-1.16 (3H, m, NC(3)H_aH$_b$, NC(5)H_aH$_b$, NC(4)H_aH$_b$), 1.24 (6H, d, *J* 7.1, CH(CH_3)$_2$), 1.32-1.40 (2H, m, NC(2)H_aH$_b$, NC(6)H_aH$_b$), 1.57-1.69 (3H, m, NC(4)H$_a$$H_b$, NC(2)H$_a$$H_b$, NC(6)H$_a$$H_b$,), 1.80-1.84 (1H, m, NC(3)H$_a$$H_b$), 1.88-1.94 (1H, m, NC(5)H$_a$$H_b$,), 1.98 (3H, s, C(2)C$H_3$), 2.67 (1H, hept, *J* 6.7, C*H*(CH$_3$)$_2$), 3.72-3.78 (1H, m, NHC*H*), 5.88 (1H, d, *J* 7.2, N*H*), 7.27-7.30 (1H, m, PhC(4)*H*), 7.33-7.36 (2H, m, PhC(3,5)*H*), 7.41-7.43 (2H, m, PhC(2,6)*H*); **^{13}C NMR** (125 MHz, $CDCl_3$) δ_C: 19.1 (2C, CH(*C*H$_3$)$_2$), 23.4 (C(2)*C*H$_3$), 24.8 (N*C*(2,6)H$_2$), 25.6 (N*C*(4)H$_2$), 33.0 (N*C*(3,5)H$_2$), 34.8 (*C*H(CH$_3$)$_2$), 48.2 (NH*C*(1)H), 83.8 (*C*(2)), 124.7 (Ph*C*(2,6)H), 128.0 (Ph*C*(4)H), 128.6 (Ph*C*(3,5)H), 141.3 (Ph*C*(1)), 170.3 (*C*(1)=O), 174.5 (*C*(O)*i*-Pr); **HRMS** (ESI$^+$) $C_{19}H_{27}NNaO_3^+$ ([M+Na]$^+$) requires 340.1883; found 340.1872 (–3.3 ppm).

1-Oxo-1,2-diphenylpropan-2-yl isobutyrate S24

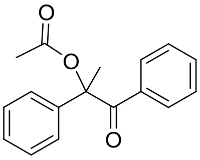

Following general procedure F, 2-hydroxy-1,2-diphenylpropan-1-one **4** (36 mg, 0.16 mmol), acetic anhydride (61 µL, 0.64 mmol), DMAP (2 mg, 0.016 mmol, 10 mol %) and NEt_3 (133 µL, 0.96 mmol) were reacted in CH_2Cl_2 (1 mL) at 50 °C to give the crude product, which was product was purified by column chromatography (eluent Petrol/EtOAc, 9:1; R_F 0.29) to give 1-oxo-1,2-diphenylpropan-2-yl isobutyrate **S24** as a yellow oil (37 mg, 0.13 mmol, 84%); **ν_{max} (ATR)** 3026, 1738 (C=O), 1690 (C=O), 1447, 1368, 1211; **^{1}H NMR** (400 MHz, $CDCl_3$) δ_H: 1.95 (3H, s, CH_3), 2.00 (C=OCH_3), 7.23-7.29 (2H, m, C(2)ArC(3,5)*H*), 7.29-7.34 (1H, m, C(2)ArC(4)*H*), 7.36-7.42 (3H, m, C=OArC(3,4,5)*H*), 7.52-7.56 (2H, m, C(2)ArC(2,6)*H*), 7.68-7.73 (2H, m, C=OArC(2,6)*H*); **^{13}C NMR** (100 MHz, $CDCl_3$) δ_C: 21.4 (*C*H$_3$), 26.8 (C=O*C*H$_3$), 87.1 (*C*(2)C=OPh), 124.2 (C(2)Ar*C*(2,6)H), 128.0 (C(2)Ar*C*(4)H), 128.1 (C(2)Ar*C*(3,5)H), 129.0 (C=OAr*C*(3,5)H), 129.1 (C=OAr*C*(2,6)H), 132.2 (C=OAr*C*(4)H), 135.0 (C=OAr*C*(1)), 140.3 (C(2)Ar*C*(1)), 169.5 (*C*=OCH$_3$), 196.8 (*C*=OPh); ***m/z* (NSI)** 286 ([M+NH$_4$]$^+$, 100%) $C_{17}H_{20}NO_3^+$ ([M+NH$_4$]$^+$) requires 286.1438; found 286.1440 (+0.8 ppm).

Methyl 2-acetoxy-2-phenylpropanoate S25

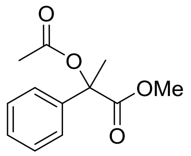

Following general procedure F, methyl 2-hydroxy-2-phenypropanoate **5** (29 mg, 0.16 mmol), acetic anhydride (61 µL, 0.64 mmol), DMAP (2 mg, 0.016 mmol, 10 mol%) and NEt_3 (133 µL, 0.96 mmol) were reacted in CH_2Cl_2 (1 mL) to give the crude product, which was purified by column chromatography (eluent Petrol/EtOAc, 4:1; R_F 0.32) to give methyl 2-acetoxy-2-phenylpropanoate **S25** as a colourless oil (31 mg, 0.14 mmol, 87%); **IR** ν_{max} (film): 2953, 1736 (C=O), 1371, 1253, 1221; **^{1}H NMR** (400 MHz, $CDCl_3$) δ_H:1.95 (3H, s, C(2)C*H*$_3$), 2.21 (3H, s, C(O)C*H*$_3$), 3.69 (3H, s, OC*H*$_3$), 7.30-7.41 (3H, m, PhC(3,4,5)*H*), 7.49-7.54 (2H, m, PhC(2,6)*H*); **^{13}C NMR** (100 MHz, $CDCl_3$) δ_C: 21.4 (C(O)*C*H$_3$), 24.1 (C(2)*C*H$_3$), 52.8 (O*C*H$_3$), 87.1 (*C*(2)), 124.7 (Ph*C*(2,6)H), 128.3 (Ph*C*(4)H), 128.6 (Ph*C*(3,5)H), 139.7 (Ph*C*(1)), 169.9 (*C*(O)Me), 171.4 (*C*(O)OMe); **HRMS** (NSI^+) $C_{12}H_{18}NO_4^+$ ($[M+NH_4]^+$) requires 240.1230; found 240.1232 (+0.7 ppm).

Methyl 2-phenyl-2-(propionyloxy)propanoate S26

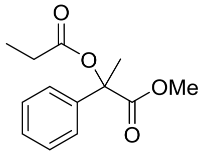

Following general procedure F, methyl 2-hydroxy-2-phenypropanoate **5** (29 mg, 0.16 mmol), propionic anhydride (82 µL, 0.64 mmol), DMAP (2 mg, 0.016 mmol, 10 mol%) and NEt_3 (133 µL, 0.96 mmol) were reacted in CH_2Cl_2 (1 mL) to give the crude product, which was purified by column chromatography (eluent CH_2Cl_2/EtOAc, 95:5; R_F 0.75) to give methyl 2-phenyl-2-(propionyloxy)propanoate **S26** as a colourless oil (27 mg, 0.11 mmol, 71%); **IR** ν_{max} (film): 2951, 1740 (C=O), 1261, 1177, 1119; **^{1}H NMR** (400 MHz, $CDCl_3$) δ_H: 1.22 (3H, t, *J* 7.6, CH$_2$C*H*$_3$), 1.95 (3H, s, C(2)C*H*$_3$), 2.41-2.58 (2H, m, C*H*$_2$CH$_3$), 3.69 (3H, s, OC*H*$_3$), 7.30-7.35 (1H, m, PhC(4)*H*), 7.35-7.40 (2H, m, PhC(3,5)*H*), 7.49-7.54 (2H, m, PhC(2,6)*H*); **^{13}C NMR** (100 MHz, $CDCl_3$) δ_C: 9.1 (CH$_2$*C*H$_3$), 24.1 (C(2)*C*H$_3$), 28.0 (*C*H$_2$CH$_3$), 52.7 (O*C*H$_3$), 81.4 (*C*(2)), 124.7 (Ph*C*(2,6)H), 128.2 (Ph*C*(4)H), 128.6 (Ph*C*(3,5)H), 139.9 (Ph*C*(1)), 171.4 (*C*(O)Et), 173.3 (*C*(O)OMe); **HRMS** (NSI^+) $C_{13}H_{20}NO_4^+$ ($[M+NH_4]^+$) requires 254.1387; found 254.1389 (+0.8 ppm).

Methyl 2-(isobutyryloxy)-2-phenylpropanoate S27

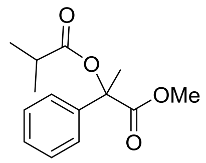

Following general procedure F, methyl 2-hydroxy-2-phenypropanoate **5** (29 mg, 0.16 mmol), isobutyric anhydride (106 µL, 0.64 mmol), DMAP (2 mg, 0.016 mmol, 10 mol%) and NEt_3 (133 µL, 0.96 mmol) were reacted in CH_2Cl_2 (1 mL) to give the crude product, which was purified by column chromatography (eluent CH_2Cl_2/EtOAc, 95:5; R_F 0.75) to give methyl 2-(isobutyryloxy)-2-phenylpropanoate **S27** as a colourless oil (37 mg, 0.15 mmol, 92%); **IR** ν_{max} (film): 2976, 1740 (C=O), 1260, 1153, 1119; **^{1}H NMR** (400 MHz, $CDCl_3$) δ_H: 1.26 (6H, app t, *J* 7.2, $CH(CH_3)_2$), 1.94 (3H, s, $C(2)CH_3$), 2.71 (1H, m, $CH(CH_3)_2$), 3.68 (3H, s, OCH_3), 7.30-7.35 (1H, m, PhC(4)*H*), 7.35-7.40 (2H, m, PhC(3,5)*H*), 7.50-7.54 (2H, m, PhC(2,6)*H*); **^{13}C NMR** (125 MHz, $CDCl_3$) δ_C: 18.8 ($CH(CH_3)_a(CH_3)_b$), 18.9 ($CH(CH_3)_a(CH_3)_b$), 24.0 ($C(2)CH_3$), 34.3 ($CH(CH_3)_2$), 52.7 (OCH_3), 81.3 (*C*(2)), 124.7 (Ph*C*(2,6)H), 128.2 (Ph*C*(4)H), 128.6 (Ph*C*(3,5)H), 140.0 (Ph*C*(1)), 171.4 (*C*(O)OMe), 175.8 (*C*(O)*i*-Pr); **HRMS** (NSI^+) $C_{14}H_{22}NO_4^+$ ($[M+NH_4]^+$) requires 268.1543; found 268.1546 (+1.0 ppm).

Ethyl 2-(isobutyryloxy)-2-phenylpropanoate S28

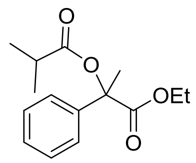

Following general procedure F, ethyl 2-hydroxy-2-phenylpropanoate **6** (31 mg, 0.16 mmol), isobutyric anhydride (106 µL, 0.64 mmol), DMAP (2 mg, 0.016 mmol, 10 mol%) and NEt_3 (133 µL, 0.96 mmol) were reacted in CH_2Cl_2 (1 mL) to give the crude product, which was purified by column chromatography (eluent CH_2Cl_2/EtOAc, 95:5; R_F 0.65) to give ethyl 2-(isobutyryloxy)-2-phenylpropanoate **S28** as a colourless oil (38 mg, 0.14 mmol, 89%); **IR** ν_{max} (film): 2976, 1736 (C=O), 1449, 1258, 1117; **^{1}H NMR** (400 MHz, $CDCl_3$) δ_H: 1.18 (3H, app t, *J* 7.1, OCH_2CH_3), 1.25 (3H, d, *J* 7.0, $CH(CH_3)_a(CH_3)_b$), 1.27 (3H, d, *J* 7.0, $CH(CH_3)_a(CH_3)_b$), 1.94 (3H, s, $C(2)CH_3$), 2.71 (1H, app hept, *J* 7.0, $CH(CH_3)_2$), 4.08 (1H, dq, *J* 10.8, 7.1, $OCH_aH_bCH_3$), 4.20 (1H, dq, *J* 10.8, 7.1, $OCH_aH_bCH_3$), 7.29-7.34 (1H, m, PhC(4)*H*), 7.34-7.40 (2H, m, PhC(3,5)*H*), 7.50-7.55 (2H, m, PhC(2,6)*H*); **^{13}C NMR** (100 MHz, $CDCl_3$) δ_C: 13.9 (CH_2CH_3), 18.8 ($CH(CH_3)_a(CH_3)_b$), 18.9 ($CH(CH_3)_a(CH_3)_b$), 24.0 ($C(2)CH_3$), 34.3 ($CH(CH_3)_2$), 61.6 (OCH_2CH_3), 81.3 (*C*(2)), 124.7 (Ph*C*(2,6)H), 128.1 (Ph*C*(4)H), 128.5 (Ph*C*(3,5)H), 140.2 (Ph*C*(1)), 170.8 (*C*(O)OEt), 175.7 (*C*(O)*i*-Pr); **HRMS** (NSI^+) $C_{15}H_{20}O_4Na^+$ ($[M+Na]^+$) requires 287.1254; found 287.1256 (+0.8 ppm).

tert-Butyl 2-(isobutyryloxy)-2-phenylpropanoate S29

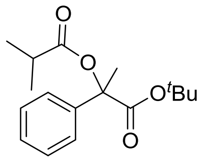

Following general procedure F, *tert*-butyl 2-hydroxy-2-phenylpropanoate **7** (36 mg, 0.16 mmol), isobutyric anhydride (106 µL, 0.64 mmol), DMAP (2 mg, 0.016 mmol, 10 mol%) and NEt_3 (133 µL, 0.96 mmol) were reacted in CH_2Cl_2 (1 mL) to give the crude product, which was purified by column chromatography (eluent Petrol/Et_2O, 9:1; R_F 0.31) to give *tert*-butyl 2-(isobutyryloxy)-2-phenylpropanoate **S29** as a colourless oil (36 mg, 0.12 mmol, 78%); **IR** ν_{max} (film): 2976, 1734 (C=O), 1448, 1368, 1277, 1119; **^{1}H NMR** (400 MHz, $CDCl_3$) δ_H:1.25 (3H, d, *J* 7.0, $CH(CH_3)_a(CH_3)_b$), 1.28 (3H, d, *J* 7.0, $CH(CH_3)_a(CH_3)_b$), 1.37 (9H, s, $C(CH_3)_3$), 1.89 (1H, s, $C(2)CH_3$), 2.69 (1H, app hept, *J* 7.0, $CH(CH_3)_2$), 7.28-7.33 (1H, m, PhC(4)*H*), 7.34-7.40 (2H, m, Ph(3,5)*H*), 7.50-7.55 (2H, m, PhC(2,6)*H*); **^{13}C NMR** (100 MHz, $CDCl_3$) δ_C: 18.8 ($CH(CH_3)_a(CH_3)_b$), 19.1 ($CH(CH_3)_a(CH_3)_b$), 23.9 ($C(2)CH_3$), 27.6 ($C(CH_3)_3$), 34.3 ($CH(CH_3)_2$), 81.7 (*C*(2)), 81.8 ($OC(CH_3)_3$), 124.7 (Ph*C*(2,6)H), 127.8 (Ph*C*(4)H), 128.3 (Ph*C*(3,5)H), 140.7 (Ph*C*(1)), 169.7 (*C*(O)OBn), 175.4 (*C*(O)*i*-Pr); **HRMS** (NSI^+) $C_{17}H_{28}NO_4^+$ ($[M+NH_4]^+$) requires 310.2013; found 310.2016 (+1.0 ppm).

Benzyl 2-(isobutyryloxy)-2-phenylpropanoate 10

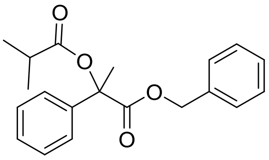

Following general procedure F, benzyl 2-hydroxy-2-phenypropanoate **8** (41 mg, 0.16 mmol), isobutyric anhydride (106 µL, 0.64 mmol), DMAP (2 mg, 0.016 mmol, 10 mol%) and NEt_3 (133 µL, 0.96 mmol) were reacted in CH_2Cl_2 (1 mL) to give the crude product, which was purified by column chromatography (eluent Petrol/Et_2O, 9:1; R_F 0.43) to give benzyl 2-(isobutyryloxy)-2-phenylpropanoate **10** as a colourless oil (45 mg, 0.14 mmol, 87%); **IR** ν_{max} (film): 2974, 1736 (C=O), 1449, 1256, 1113; **^{1}H NMR** (400 MHz, $CDCl_3$) δ_H:1.18 (3H, d, *J* 7.0, $CH(CH_3)_a(CH_3)_b$), 1.20 (3H, d, *J* 7.0, $CH(CH_3)_a(CH_3)_b$), 1.97 (3H, s, $C(2)CH_3$), 2.67 (1H, app hept, *J* 7.0, $CH(CH_3)_2$), 5.06 (1H, d, *J* 12.3, OCH_aH_bPh), 5.16 (1H, d, *J* 12.3, OCH_aH_bPh), 7.17-7.22 (2H, m, $OCH_2PhC(2,6)H$), 7.26-7.38 (6H, m, C(2)Ph(3,4,5)*H*, $OCH_2PhC(3,4,5)H$), 7.48-7.53 (2H, m, C(2)PhC(2,6)*H*); **^{13}C NMR** (100 MHz, $CDCl_3$) δ_C:18.76 ($CH(CH_3)_a(CH_3)_b$), 18.79 ($CH(CH_3)_a(CH_3)_b$), 23.8 ($C(2)CH_3$), 34.3 ($CH(CH_3)_2$), 67.2 (OCH_2Ph), 81.3 (*C*(2)), 124.8 (C(2)Ph*C*(2,6)H), 128.1 (4C, $OCH_2PhC(2,3,5,6)H$), 128.4 (2C, $OCH_2PhC(4)H$, C(2)Ph*C*(4)H), 128.5 (C(2)Ph*C*(3,5)H), 135.4 ($OCH_2PhC(1)$), 139.9 (C(2)Ph*C*(1)), 170.7 (*C*(O)OBn), 175.7 (*C*(O)*i*-Pr); **HRMS** (NSI^+) $C_{20}H_{22}O_4Na^+$ ($[M+Na]^+$) requires 349.1410; found 349.1411 (+0.3 ppm).

Benzyl 2-acetoxy-2-phenylpropanoate S30

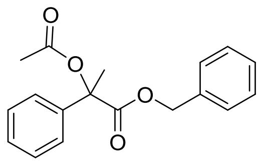

Following general procedure F, benzyl 2-hydroxy-2-phenylpropanoate **8** (69 mg, 0.27 mmol), acetic anhydride (102 µL, 1.08 mmol), DMAP (3.3 mg, 0.027 mmol, 10 mol%) and NEt_3 (226 µL, 1.62 mmol) were reacted in CH_2Cl_2 (2 mL) to give the crude product, which was purified by column chromatography (Isolera 4, Et_2O in petrol, 0% to 20% over 30CV; R_F 0.21 for Petrol/Et_2O 9:1) to give benzyl 2-acetoxy-2-phenylpropanoate **S30** as a colourless oil (45 mg, 0.15 mmol, 56%); **IR** ν_{max} (film): 2945, 1740 (C=O), 14496, 1255, 1220, 1119; **^{1}H NMR** (500 MHz, $CDCl_3$) δ_H: 1.98 (3H, s, C(2)C*H*$_3$), 2.18 (3H, s, C(O)C*H*$_3$), 5.08-5.17 (2H, m, OC*H*$_2$Ph), 7.17-7.21 (2H, m, PhC*H*), 7.27-7.38 (6H, m, PhC*H*), 7.47-7.51 (2H, m, PhC*H*); **^{13}C NMR** (125 MHz, $CDCl_3$) δ_C: 21.5 (C(O)*C*H$_3$), 24.0 (*C*(2)*C*H$_3$), 67.4 (O*C*H$_2$Ph), 81.9 (*C*(2)), 125.0 (2 x Ph*C*H), 128.1 (2 x Ph*C*H), 128.3 (Ph*C*H), 128.4 (2 x Ph*C*H), 128.6 (2 x Ph*C*H), 128.7 (Ph*C*H), 135.6 (Ph*C*(1)), 139.7 (Ph*C*(1)), 170.1 (*C*(O)CH$_3$), 170.8 (*C*(O)OBn); **HRMS** (ESI$^+$) $C_{18}H_{18}O_4Na^+$ ([M+Na]$^+$) requires 321.1097; found 321.1089 (–2.5 ppm).

Benzyl 2-(isobutyryloxy)-2-(naphthalen-2-yl)propanoate S31

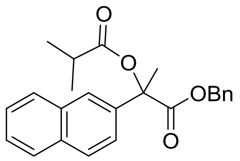

Following general procedure F, benzyl 2-hydroxy-2-(naphthalene-2-yl)propanoate **11** (49 mg, 0.16 mmol), isobutyric anhydride (106 µL, 0.64 mmol), DMAP (2 mg, 0.016 mmol, 10 mol%) and NEt_3 (133 µL, 0.96 mmol) were reacted in CH_2Cl_2 (1 mL) to give the crude product, which was purified by column chromatography (eluent Petrol/Et_2O, 9:1; R_F 0.39) to give benzyl 2-(isobutyryloxy)-2-(naphthalen-2-yl)propanoate **S31** as a colourless oil (43 mg, 0.11 mmol, 71%); **IR** ν_{max} (film): 2974, 1736 (C=O), 1373, 1256, 1110; **^{1}H NMR** (400 MHz, $CDCl_3$) δ_H: 1.22 (3H, d, *J* 7.0, CH(C*H*$_3$)$_a$(CH$_3$)$_b$), 1.24 (3H, d, *J* 7.0, CH(CH$_3$)$_a$(C*H*$_3$)$_b$), 2.06 (3H, s, C(2)C*H*$_3$), 2.73 (1H, app hept, *J* 7.0, C*H*(CH$_3$)$_2$), 5.06 (1H, d, *J* 12.3, OC*H*$_a$H$_b$Ph), 5.18 (1H, d, *J* 12.3, OCH$_a$*H*$_b$Ph), 7.17-7.22 (2H, m, PhC(2,6)*H*), 7.23-7.29 (3H, m, PhC(3,4,5)*H*), 7.47-7.53 (2H, m, C(2)ArC(6,7)*H*), 7.62 (1H, dd, *J* 8.7, 1.9, C(2)ArC(3)*H*), 7.78-7.87 (3H, m, C(2)ArC(4,5,8)*H*), 7.94 (1H, d, *J* 1.8, C(2)ArC(1)*H*); **^{13}C NMR** (100 MHz, $CDCl_3$) δ_C: 18.8 (2C, CH(*C*H$_3$)$_2$), 23.9 (C(2)*C*H$_3$), 34.3 (*C*H(CH$_3$)$_2$), 67.4 (O*C*H$_2$Ph), 81.4 (*C*(2)), 122.7 (C(2)Ar*C*(3)H), 124.0 (C(2)Ar*C*(1)H), 126.4 (C(2)Ar*C*(6)H), 126.5 (C(2)Ar*C*(7)H), 127.5 (C(2)Ar*C*(5)H), 128.19 (C(2)Ar*C*(8)H), 128.27 (Ph*C*(2,6)H), 128.33 (Ph*C*(4)H), 128.35 (Ph*C*(3,5)H), 128.39 (C(2)Ar*C*(4)H), 132.9 (C(2)Ar*C*(8a)), 133.0 (C(2)Ar*C*(4a)), 135.3 (Ph*C*(1)), 137.2 (C(2)Ar*C*(2)), 170.7 (*C*(O)OBn), 175.7 (*C*(O)*i*-Pr); **HRMS** (NSI$^+$) $C_{24}H_{28}NO_4^+$ ([M+NH$_4$]$^+$) requires 394.2013; found 394.2008 (–1.2 ppm).

Benzyl 2-(isobutyryloxy)-2-(*p*-tolyl)propanoate S32

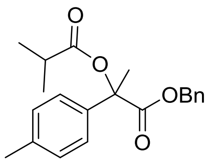

Following general procedure F, benzyl 2-hydroxy-2-(*p*-tolyl)propanoate **12** (43 mg, 0.16 mmol), isobutyric anhydride (106 µL, 0.64 mmol), DMAP (2 mg, 0.016 mmol, 10 mol%) and NEt_3 (133 µL, 0.96 mmol) were reacted in CH_2Cl_2 (1 mL) to give the crude product, which was purified by column chromatography (eluent Petrol/Et_2O, 9:1; R_F 0.19) to give benzyl 2-(isobutyryloxy)-2-(*p*-tolyl)propanoate **S32** as a colourless oil (46 mg, 0.14 mmol, 85%); **IR** ν_{max} (film): 2974, 1736 (C=O), 1456, 1258, 1155, 1099; ^{1}**H NMR** (400 MHz, $CDCl_3$) δ_H: 1.17 (3H, d, *J* 7.0, $CH(CH_3)_a(CH_3)_b$), 1.18 (3H, d, *J* 7.0, $CH(CH_3)_a(CH_3)_b$), 1.95 (3H, s, C(2)CH_3), 2.35 (3H, s, ArC(4)CH_3), 2.65 (1H, hept, *J* 7.0, C*H*$(CH_3)_2$), 5.04 (1H, d, *J* 12.3, OCH_aH_bPh), 5.15 (1H, d, *J* 12.3, OCH_aH_bPh), 7.14-7.18 (2H, m, C(2)ArC(3,5)*H*), 7.19-7.22 (2H, m, PhC(2,6)*H*), 7.27-7.31 (3H, m, PhC(3,4,5)*H*), 7.37-7.42 (2H, m, C(2)ArC(2,6)*H*); ^{13}**C NMR** (100 MHz, $CDCl_3$) δ_C: 18.76 ($CH(CH_3)_a(CH_3)_b$), 18.79 ($CH(CH_3)_a(CH_3)_b$), 21.1 (ArC(4)CH_3), 23.7 (C(2)CH_3), 34.2 (*C*H$(CH_3)_2$), 67.2 (OCH_2Ph), 81.2 (*C*(2)), 124.2 (C(2)Ar*C*(2,6)H), 128.1 (Ph*C*(4)H), 128.2 (Ph*C*(2,6)H), 128.3 (Ph*C*(3,5)H), 129.2 (C(2)Ar*C*(3,5)H), 135.4 (Ph*C*(1)), 137.0 (C(2)Ar*C*(4)), 138.0 (C(2)Ar*C*(1)), 170.9 (*C*(O)OBn), 175.8 (*C*(O)*i*-Pr); **HRMS** (NSI^+) $C_{21}H_{28}NO_4^+$ ($[M+NH_4]^+$) requires 358.2013; found 358.2016 (+0.9 ppm).

Benzyl 2-(4-(*tert*-butyl)phenyl)-2-(isobutyryloxy)propanoate S33

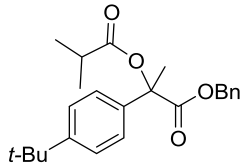

Following general procedure F, benzyl 2-(4-(*tert*-butyl)phenyl)-2-hydroxypropanoate **13** (50 mg, 0.16 mmol), isobutyric anhydride (106 µL, 0.64 mmol), DMAP (2 mg, 0.016 mmol, 10 mol%) and NEt_3 (133 µL, 0.96 mmol) were reacted in CH_2Cl_2 (1 mL) to give the crude product, which was purified by column chromatography (Isolera 4, Et_2O in petrol, 0% to 10% over 30 CV; R_F 0.48) to give benzyl 2-(4-(*tert*-butyl)phenyl)-2-(isobutyryloxy)propanoate **S33** as a colourless oil (41 mg, 0.11 mmol, 67%); **IR** ν_{max} (film): 2965 (C-H), 1740 (C=O), 1456 (C=C), 1260 (C=C), 1115 (C-O); ^{1}**H NMR** (500 MHz, $CDCl_3$) δ_H: 1.18 (3H, d, *J* 7.0, $CH(CH_3)_2$), 1.19 (3H, d, *J* 7.0, $CH(CH_3)_2$), 1.32 (9H, s, $C(CH_3)_3$), 1.97 (3H, s, C(2)CH_3), 2.65 (1H, app hept, *J* 7.0, C*H*$(CH_3)_2$), 5.07 (1H, d, *J* 12.3, OCH_aH_bPh), 5.16 (1H, d, *J* 12.3, OCH_aH_bPh), 7.17-7.21 (2H, m, Ph*C*(2,6)H), 7.27-7.29 (3H, m, Ph*C*(3,4,5)H) 7.36-7.38 (2H, m, ArC(3,5)*H*), 7.42-7.45 (2H, m, ArC(2,6)*H*); ^{13}**C NMR** (125 MHz, $CDCl_3$) δ_C: 18.9 (2C, $CH(CH_3)_2$), 23.7 (C(2)CH_3), 31.4 ($C(CH_3)_3$), 34.4 (*C*H), 34.7 (*C*$(CH_3)_3$), 67.2 (CH_2), 81.3 (*C*(2)), 124.7 (C(2)Ar*C*(2,6)H), 125.6 (C(2)Ar*C*(3,5)H), 128.2 (3C, Ph*C*(2,6)H, Ph*C*(4)H), 128.5 (Ph*C*(3,5)H), 135.6 (Ph*C*(1)), 136.9 (C(2)Ar*C*(1)), 151.3 (C(2)Ar*C*(4)), 171.0 (*C*(O)OBn), 175.9 (*C*(O)*i*-Pr); **HRMS** (ESI^+) $C_{24}H_{30}NaO_4^+$ ($[M+Na]^+$) requires 405.2036; found 405.2022 (–3.5 ppm).

Benzyl 2-(isobutyryloxy)-2-(4-methoxyphenyl)propanoate S34

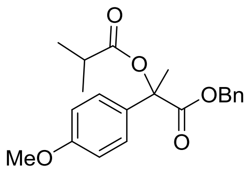

Following general procedure F, benzyl 2-hydroxy-2-(4-methoxyphenyl)propanoate **14** (46 mg, 0.16 mmol), isobutyric anhydride (106 µL, 0.64 mmol), DMAP (2 mg, 0.016 mmol, 10 mol%) and NEt_3 (133 µL, 0.96 mmol) were reacted in CH_2Cl_2 (1 mL) to give the crude product, which was purified by column chromatography (eluent Petrol/Et_2O, 4:1; R_F 0.34) to give benzyl 2-(isobutyryloxy)-2-(4-methoxyphenyl)propanoate **S34** as a colourless oil (39 mg, 0.11 mmol, 69%); **IR** ν_{max} (film): 2972, 1736 (C=O), 1611, 1572, 1456, 1250; **^{1}H NMR** (500 MHz, $CDCl_3$) δ_H: 1.17 (3H, d, *J* 7.0, $CH(CH_3)_a(CH_3)_b$), 1.18 (3H, d, *J* 7.0, $CH(CH_3)_a(CH_3)_b$), 1.96 (3H, s, C(2)CH_3), 2.64 (1H, app hept, *J* 7.0, C*H*$(CH_3)_2$), 3.81 (3H, s, OCH_3), 5.05 (1H, d, *J* 12.3, OCH_aH_bPh), 5.14 (1H, d, *J* 12.3, OCH_aH_bPh), 6.85-6.90 (2H, m, C(2)ArC(3,5)*H*), 7.18-7.22 (2H, m, PhC(2,6)*H*), 7.27-7.32 (3H, m, PhC(3,4,5)*H*), 7.41-7.46 (2H, m, C(2)ArC(2,6)*H*); **^{13}C NMR** (100 MHz, $CDCl_3$) δ_C: 18.75 ($CH(CH_3)_a(CH_3)_b$), 18.80 ($CH(CH_3)_a(CH_3)_b$), 23.5 (C(2)CH_3), 34.2 ($CH(CH_3)_2$), 55.3 (OCH_3), 67.2 (OCH_2Ph), 81.0 (*C*(2)), 113.8 (C(2)Ar*C*(3,5)H), 126.3 (C(2)Ar*C*(2,6)H), 128.1 (Ph*C*(2,6)H), 128.4 (Ph*C*(3,4,5)H), 131.9 (Ph*C*(1)), 135.4 (C(2)Ar*C*(1)), 159.4 (C(2)Ar*C*(4)), 170.9 (*C*(O)OBn), 175.8 (*C*(O)*i*-Pr); **HRMS** (NSI^+) $C_{21}H_{28}NO_5^+$ ($[M+NH_4]^+$) requires 374.1962; found 371.1963 (+0.3 ppm).

Benzyl 2-(isobutyryloxy)-2-(3-methoxyphenyl)propanoate S35

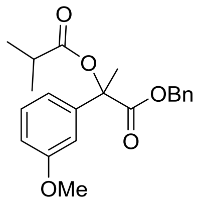

Following general procedure F, benzyl 2-hydroxy-2-(3-methoxyphenyl)propanoate **15** (46 mg, 0.16 mmol), isobutyric anhydride (106 µL, 0.64 mmol), DMAP (2 mg, 0.016 mmol, 10 mol%) and NEt_3 (133 µL, 0.96 mmol) were reacted in CH_2Cl_2 (1 mL) to give the crude product, which was purified by column chromatography (Isolera 4, Et_2O in petrol, 0% to 15% over 25 CV; R_F 0.49) to give benzyl 2-(isobutyryloxy)-2-(3-methoxyphenyl)propanoate **S35** as a colourless oil (51 mg, 0.14 mmol, 89%); **IR** ν_{max} (film): 2974 (C-H), 1740 (C=O), 1585, 1456 (C=C), 1260 (C=C), 1117 (C-O); **^{1}H NMR** (500 MHz, $CDCl_3$) δ_H: 1.19 (6H, app. t, *J* 6.7, $CH(CH_3)_2$), 1.95 (3H, m, C(2)CH_3), 2.66 (1H, app hept, *J* 6.9, C*H*$(CH_3)_2$), 3.77 (3H, s, OCH_3), 5.06 (1H, d, *J* 12.4Hz, OCH_aH_bPh), 5.16 (1H, d, *J* 12.4, OCH_aH_bPh), 6.84-6.87 (1H, m, C(2)ArC(4)*H*), 7.05-7.09 (2H, m, C(2) ArC(2,6)*H*), 7.19-7.23 (2H, m, PhC(2,6)*H*), 7.25-7.31 (4H, m, C(2)ArC(5)*H*, PhC(3,4,5)*H*); **^{13}C NMR** (100 MHz, $CDCl_3$) δ_C:18.9 ($CH(CH_3)(CH_3)$), 18.9 ($CH(CH_3)(CH_3)$), 24.1 (C(2)CH_3), 34.4 (*C*H), 55.3 (OCH_3) 67.4 (CH_2), 81.2 (*C*(2)), 111.0 (C(2)Ar*C*(2)H), 113.5 (C(2)Ar*C*(4)H), 117.2 (C(2)Ar*C*(6)H), 128.3 (Ph*C*(4)H), 128.3 (Ph*C*(2,6)H), 128.5 (Ph*C*(3,5)H), 129.7 (C(2)Ar*C*(5)H), 135.5 (Ph*C*(1)), 141.6 (C(2)Ar*C*(1)), 159.7 (C(2)Ar*C*(3)), 170.7 (*C*(O)OBn), 175.7 (*C*(O)*i*-Pr); **HRMS** (ESI^+) $C_{21}H_{24}NaO_5^+$ ($[M+Na]^+$) requires 379.1516; found 379.1502 (–3.7 ppm).

Benzyl 2-(isobutyryloxy)-2-(2-methoxyphenyl)propanoate S36

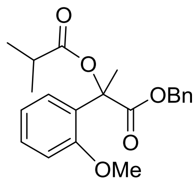

Following general procedure F, benzyl 2-hydroxy-2-(2-methoxyphenyl)propanoate **16** (45.8 mg, 0.16 mmol), isobutyric anhydride (106 µL, 0.64 mmol), DMAP (2 mg, 0.016 mmol, 10 mol%) and NEt_3 (133 µL, 0.96 mmol) were reacted in CH_2Cl_2 (1 mL) to give the crude product, which was purified by column chromatography (Isolera 4, Et_2O in petrol, 0% to 20% over 25 CV; R_F 0.26) to give benzyl 2-(isobutyryloxy)-2-(2-methoxyphenyl)propanoate **S36** as a colourless oil (8.8 mg, 0.03 mmol, 15%); **IR** ν_{max} (film): 2974 (C-H), 1761 (C=O), 1738 (C=O), 1491 (C=C), 1254 (C=C), 1115 (C-O); **^{1}H NMR** (500 MHz, $CDCl_3$) δ_H: 1.18 (3H, d, *J* 7.0, CH(CH_3)$_2$), 1.21 (3H, d, *J* 7.0, CH(CH_3)$_2$), 1.92 (3H, s, C(2)CH_3), 2.66 (1H, app hept, *J* 7.0, C*H*(CH_3)$_2$), 3.55 (3H, s, OCH_3), 5.15 (1H, d, *J* 12.5, OC$H_a$$H_b$Ph), 5.21 (1H, d, *J* 12.5, OC$H_a$$H_b$Ph), 6.81-6.83 (1H, m, C(2)ArC(3)*H*), 6.98-7.01 (1H, m, C(2)ArC(5)*H*), 7.27-7.32 (6H, m, C(2)ArC(4)*H*, PhC*H*), 7.62-7.64 (1H, m, C(2)ArC(6)*H*); **^{13}C NMR** (125 MHz, $CDCl_3$) δ_C: 18.9 (CH(*C*H_3)(CH_3)), 19.1 (CH(CH_3)(*C*H_3)), 22.9 (C(2)*C*H_3), 34.4 (*C*H), 55.2 (O*C*H_3) 66.9 (*C*H_2), 80.9 (*C*(2)), 111.4 (C(2)Ar*C*(3)H), 120.8 (C(2) Ar*C*(5)H), 126.8 (C(2)Ar*C*(6)H), 128.1 (Ph*C*(4)H), 128.4 (2 × Ph*C*H), 128.5 (2 × Ph*C*H), 129.4 (C(2)Ar*C*(4)H), 129.6 (C(2)Ar*C*(1)), 136.2 (Ph*C*(1)), 155.7 (C(2)Ar*C*(2)), 170.9 (*C*(O)OBn), 175.4 (*C*(O)*i*-Pr); **HRMS** (ESI^+) $C_{21}H_{24}NaO_5^+$ ($[M+Na]^+$) requires 379.1516; found 379.1508 (–2.1 ppm).

Benzyl 2-(3,5-bis(trifluoromethyl)phenyl)-2-(isobutyryloxy)propanoate S37

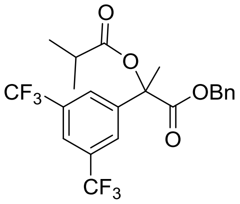

Following general procedure F, benzyl 2-(3,5-bis(trifluoromethyl)phenyl)-2-hydroxypropanoate **17** (63 mg, 0.16 mmol), isobutyric anhydride (106 µL, 0.64 mmol), DMAP (2 mg, 0.016 mmol, 10 mol%) and NEt_3 (133 µL, 0.96 mmol) were reacted in CH_2Cl_2 (1 mL) to give the crude product, which was purified by column chromatography (eluent Petrol/Et_2O, 9:1; R_F 0.43) to give benzyl 2-(3,5-bis(trifluoromethyl)phenyl)-2-(isobutyryloxy)propanoate **S37** as a colourless oil (57 mg, 0.12 mmol, 77%); **IR** ν_{max} (film): 2978, 1744 (C=O), 1373, 1277, 1126; **^{1}H NMR** (400 MHz, $CDCl_3$) δ_H: 1.20 (3H, d, *J* 7.0, CH(CH_3)$_a$(CH_3)$_b$), 1.21 (3H, d, *J* 7.0, CH(CH_3)$_a$(CH_3)$_b$), 1.99 (3H, s, C(2)CH_3), 2.71 (1H, app hept, *J* 7.0, C*H*(CH_3)$_2$), 5.10 (1H, d, *J* 12.1, OC$H_a$$H_b$Ph), 5.16 (1H, d, *J* 12.1, OC$H_a$$H_b$Ph), 7.17-7.21 (2H, m, PhC(3,5)*H*), 7.27-7.32 (3H, m, PhC(2,4,6)*H*), 7.83 (1H, s, C(2)ArC(4)*H*), 7.94 (2H, s, C(2)ArC(2,6)*H*); **^{13}C NMR** (100 MHz, $CDCl_3$) δ_C: 18.67 (CH(*C*H_3)$_a$(CH_3)$_b$), 18.71 (CH(CH_3)$_a$(*C*H_3)$_b$), 24.1 (C(2)*C*H_3), 34.1 (*C*H), 68.0 (*C*H_2), 80.3 (*C*(2)), 122.3 (m, C(2)Ar*C*(4)H), 123.1 (q, *J* 273, 2 × *C*F_3), 125.3 (m, C(2)Ar*C*(2,6)H), 128.4 (Ph*C*(3,5)H), 128.5 (Ph*C*(2,6)H), 128.6 (Ph*C*(4)H), 131.9 (q, *J* 34, C(2)Ar*C*(3,5)H), 134.7 (Ph*C*(1)), 142.5 (C(2)Ar*C*(1)), 169.4 (*C*(O)OBn), 174.2 (*C*(O)*i*-Pr); **^{19}F NMR** (376 MHz, $CDCl_3$) δ_F: –62.9 (2 × *C*F_3); **HRMS** (NSI^+) $C_{22}H_{24}NO_4F_6^+$ ($[M+NH_4]^+$) requires 480.1604; found 480.1593 (–2.3 ppm).

Benzyl 2-(isobutyryloxy)-2-(thiophene-2-yl)propanoate S38

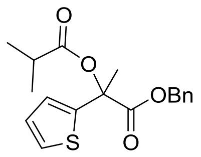

Following general procedure F, benzyl 2-hydroxy-2-(thiophene-2-yl)propanoate **18** (42 mg, 0.16 mmol), isobutyric anhydride (106 µL, 0.64 mmol), DMAP (2 mg, 0.016 mmol, 10 mol%) and NEt_3 (133 µL, 0.96 mmol) were reacted in CH_2Cl_2 (1 mL) to give the crude product, which was purified by column chromatography (eluent Petrol/Et_2O, 9:1; R_F 0.26) to give benzyl 2-(isobutyryloxy)-2-(thiophene-2-yl)propanoate **S38** as a colourless oil (46 mg, 0.14 mmol, 87%); **IR** ν_{max} (film): 2974, 1736 (C=O), 1456, 1456, 1260, 1113; **^{1}H NMR** (400 MHz, $CDCl_3$) δ_H: 1.15 (6H, app d, *J* 7.0, CH(CH_3)$_2$), 2.06 (3H, s, C(2)CH_3), 2.60 (1H, app hept, *J* 7.0, C*H*(CH_3)$_2$), 5.11 (1H, d, *J* 12.3, OC$H_a$$H_b$Ph), 5.17 (1H, d, *J* 12.3, OC$H_a$$H_b$Ph), 6.96 (1H, dd, *J* 5.1, 3.7, C(2)ArC(4)*H*), 7.09 (1H, dd, *J* 3.7, 1.2, C(2)ArC(3)*H*), 7.24-7.35 (6H, m, C(2)ArC(5)*H*, PhC*H*); **^{13}C NMR** (100 MHz, $CDCl_3$) δ_C: 18.6 (CH(CH_3)$_a$(CH_3)$_b$), 18.7 (CH(CH_3)$_a$(CH_3)$_b$), 24.2 (C(2)CH_3), 34.1 (*C*H), 67.5 (OCH_2Ph), 79.7 (*C*(2)), 124.9 (C(2)Ar*C*(3)H), 125.8 (C(2)Ar*C*(5)H), 126.7 (C(2)Ar*C*(4)H), 128.18 (Ph*C*(2,6)H), 128.24 (Ph*C*(4)H), 128.4 (Ph*C*(3,5)H), 135.2 (Ph*C*(1)), 143.0 (C(2)Ar*C*(2)), 169.8 (*C*(O)OBn), 175.6 (*C*(O)*i*-Pr); **HRMS** (NSI^+) $C_{18}H_{24}NO_4S^+$ ($[M+ NH_4]^+$) requires 350.1421; found 350.1425 (+1.3 ppm).

Benzyl 2-(isobutyryloxy)-2-(pyridin-2-yl)propanoate S39

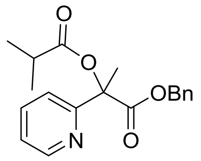

Following general procedure F, benzyl 2-hydroxy-2-(pyridin-2-yl)propanoate **19** (41 mg, 0.16 mmol), isobutyric anhydride (106 µL, 0.64 mmol), DMAP (2 mg, 0.016 mmol, 10 mol%) and NEt_3 (133 µL, 0.96 mmol) were reacted in CH_2Cl_2 (1 mL) to give the crude product, which was purified by column chromatography (Isolera 4, Et_2O in petrol, 0% to 40% over 30 CV; R_F 0.4) to give benzyl 2-(isobutyryloxy)-2-(pyridin-2-yl)propanoate **S39** as a colourless oil (35 mg, 0.11 mmol, 67%); **IR** ν_{max} (film): 1742 (C=O), 1468, 1256 (C=C), 1121 (C-O); **^{1}H NMR** (500 MHz, $CDCl_3$) δ_H: 1.18 (3H, d, *J* 7.0, CH(CH_3)(CH_3)), 1.20 (3H, d, *J* 7.0, CH(CH_3)(CH_3)), 2.00 (3H, s, C(2)CH_3), 2.66 (1H, app hept, *J* 7.0, C*H*(CH_3)$_2$), 5.13 (1H, d, *J* 12.5, OC$H_a$$H_b$Ph), 5.19 (1H, d, *J* 12.5, OC$H_a$$H_b$Ph), 7.22-7.24 (3H, m, C(2)ArC(5)*H*, PhC(2,6)*H*), 7.26-7.31 (3H, m, PhC(3,4,5)*H*), 7.65-7.67 (1H, m, C(2)ArC(3)*H*), 7.71-7.74 (1H, m, C(2)ArC(4)*H*), 8.55-8.56 (1H, m, C(2)ArC(6)*H*); **^{13}C NMR** (125 MHz, $CDCl_3$) δ_C: 18.9 (CH(CH_3)$_2$), 23.0 (C(2)CH_3), 34.3 (*C*H(CH_3)$_2$), 67.3 (CH_2), 82.9 (*C*(2)), 120.3 (C(2)Ar*C*(3)H), 123.1 (C(2)Ar*C*(5)H), 128.2 (Ph*C*(4)H), 128.3 (2 × Ph*C*H), 128.4 (2 × Ph*C*H), 135.7 (Ph*C*(1)), 137.0 (C(2)Ar*C*(4)H), 149.0 (C(2)Ar*C*(6)H), 159.0 (C(2)Ar*C*(2)), 170.0 (*C*(O)OBn), 175.6 (*C*(O)*i*-Pr); **HRMS** (ESI^+) $C_{19}H_{22}NO_4^+$ ($[M+H]^+$) requires 328.1543; found 328.1539 (–1.7 ppm).

Benzyl 2-(isobutyryloxy)-2-phenylpent-4-enoate S40

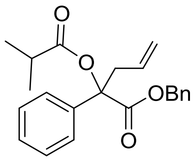

Following general procedure F, benzyl 2-hydroxy-2-phenylpent-4-enoate **20** (45 mg, 0.16 mmol), isobutyric anhydride (106 µL, 0.64 mmol), DMAP (2 mg, 0.016 mmol, 10 mol%) and NEt_3 (133 µL, 0.96 mmol) were reacted in CH_2Cl_2 (1 mL) to give the crude product, which was purified by column chromatography (eluent Petrol/Et_2O, 9:1; R_F 0.30) to give benzyl 2-(isobutyryloxy)-2-phenylpent-4-enoate **S40** as a colourless oil (49 mg, 0.14 mmol, 87%); **IR** ν_{max} (film): 2976, 1736 (C=O), 1449, 1229, 1150; **^{1}H NMR** (400 MHz, $CDCl_3$) δ_H: 1.18 (3H, d, *J* 7.0, $CH(CH_3)_a(CH_3)_b$), 1.20 (3H, d, *J* 7.0, $CH(CH_3)_a(CH_3)_b$), 1.97 (3H, s, C(2)CH_3), 2.67 (1H, app hept, *J* 7.0, C*H*$(CH_3)_2$), 3.16 (1H, ddt, *J* 15.1, 5.7, 1.5, C(2)CH_aH_b), 3.47 (1H, app dd, *J* 15.1, 8.7, C(2)CH_aH_b), 4.94-5.02 (2H, m, CH=CH_2), 5.04 (1H, d, *J* 12.3, OCH_aH_bPh), 5.17 (1H, d, *J* 12.3, OCH_aH_bPh), 5.46 (1H, dddd, *J* 17.0, 10.1, 8.7, 5.7, C*H*=CH_2), 7.15-7.21 (2H, m, OCH_2PhC(3,5)*H*), 7.26-7.38 (6H, m, C(2)Ph(3,4,5)*H*, OCH_2PhC(2,4,6)*H*), 7.47-7.53 (2H, m, C(2)PhC(2,6)*H*); **^{13}C NMR** (100 MHz, $CDCl_3$) δ_C: 18.8 ($CH(CH_3)_a(CH_3)_b$), 19.0 ($CH(CH_3)_a(CH_3)_b$), 34.2 (*C*H$(CH_3)_2$), 39.8 (C(2)*C*H_2), 67.3 (O*C*H_2Ph), 82.5 (*C*(2)), 119.1 (CH=*C*H_2), 125.1 (C(2)Ph*C*(2,6)H), 128.11 (C(2)Ph*C*(4)H), 128.14 (OCH_2Ph*C*(2,6)H), 128.2 (OCH_2Ph*C*(4)H), 128.4 (OCH_2Ph*C*(3,5)H), 128.5 (C(2)Ph*C*(3,5)H), 131.3 (*C*H=CH_2), 135.3 (OCH_2Ph*C*(1)), 137.6 (C(2)Ph*C*(1)), 170.4 (*C*(O)OBn), 175.4 (*C*(O)*i*-Pr); **HRMS** (NSI^+) $C_{20}H_{28}NO_4^+$ ($[M+NH_4]^+$) requires 370.2013; found 370.2015 (+0.6 ppm).

Benzyl 2-acetoxy-2-phenylpent-4-enoate S41

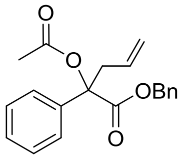

Following general procedure F, benzyl 2-hydroxy-2-phenylpent-4-enoate **20** (40 mg, 0.14 mmol), acetic anhydride (53 µL, 0.56 mmol), DMAP (1.7 mg, 0.014 mmol, 10 mol%) and NEt_3 (117 µL, 0.84 mmol) were reacted in CH_2Cl_2 (1 mL) to give the crude product, which was purified by column chromatography (eluent Petrol/Et_2O, 9:1; R_F 0.30) to give benzyl 2-acetoxy-2-phenylpent-4-enoate **S41** as a colourless oil (34 mg, 0.11 mmol, 75%); **IR** ν_{max} (film): 2953, 1743 (C=O), 1732, 1252, 1219, 1043; **^{1}H NMR** (400 MHz, $CDCl_3$) δ_H: 2.18 (3H, s, C(O)CH_3), 3.15 (1H, ddt, *J* 15.0, 5.8, 1.5, C(2)CH_aH_b), 3.46 (1H, app dd, *J* 15.0, 8.6, C(2)CH_aH_b), 4.95-5.05 (2H, m, CH=CH_2), 5.11 (1H, d, *J* 12.4, OCH_aH_bPh), 5.14 (1H, d, *J* 12.3, OCH_aH_bPh), 5.42 (1H, dddd, *J* 17.1, 10.1, 8.7, 5.8, C*H*=CH_2), 7.14-7.19 (2H, m, OCH_2PhC(3,5)*H*), 7.26-7.39 (6H, m, C(2)Ph(3,4,5)*H*, OCH_2PhC(2,4,6)*H*), 7.46-7.51 (2H, m, C(2)PhC(2,6)*H*); **^{13}C NMR** (100 MHz, $CDCl_3$) δ_C: 21.1 (C(O)*C*H_3), 39.8 (C(2)*C*H_2), 67.8 (O*C*H_2Ph), 83.1 (*C*(2)), 119.1 (CH=*C*H_2), 125.1 (C(2)Ph*C*(2,6)H), 127.9 (C(2)Ph*C*(4)H), 128.2 (OCH_2Ph*C*(2,6)H), 128.4 (OCH_2Ph*C*(3,5)H), 128.5 (3C, C(2)Ph*C*(3,5)H, OCH_2Ph*C*(4)H), 131.3 (*C*H=CH_2), 135.4 (OCH_2Ph*C*(1)), 137.3 (C(2)Ph*C*(1)), 169.5 (*C*(O)CH_3), 170.3 (*C*(O)OBn); **HRMS** (NSI^+) $C_{20}H_{24}NO_4^+$ ($[M+NH_4]^+$) requires 342.1700; found 342.1702 (+0.6 ppm).

Benzyl 2-(isobutyryloxy)-2-phenylbutanoate S42

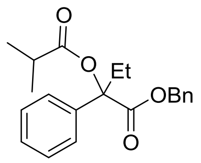

Following general procedure F, benzyl 2-hydroxy-2-phenylbutanoate **21** (43 mg, 0.16 mmol), isobutyric anhydride (106 µL, 0.64 mmol), DMAP (2 mg, 0.016 mmol, 10 mol%) and NEt_3 (133 µL, 0.96 mmol) were reacted in CH_2Cl_2 (1 mL) to give the crude product, which was purified by column chromatography (eluent Petrol/Et_2O, 9:1; R_F 0.30) to give benzyl 2-(isobutyryloxy)-2-phenylbutanoate **S42** as a colourless oil (50 mg, 0.15 mmol, 91%); **IR** ν_{max} (film): 2974, 1736 (C=O), 1449, 1234, 1128; **^{1}H NMR** (400 MHz, $CDCl_3$) δ_H: 0.64 (3H, t, *J* 7.5, C(2)CH_2CH_3), 1.20 (3H, d, *J* 7.0, CH$(CH_3)_a(CH_3)_b$), 1.23 (3H, d, *J* 7.0, CH$(CH_3)_a(CH_3)_b$), 2.38 (1H, dq, *J* 15.0, 7.5, C(2)$CH_aH_bCH_3$), 2.65-2.78 (2H, m, C(2)$CH_aH_bCH_3$, $CH(CH_3)_2$), 5.03 (1H, d, *J* 12.4, OCH_aH_bPh), 5.14 (1H, d, *J* 12.4, OCH_aH_bPh), 7.14-7.19 (2H, m, OCH_2PhC(3,5)*H*), 7.25-7.38 (6H, m, C(2)Ph(3,4,5)*H*, OCH_2PhC(2,4,6)*H*), 7.47-7.51 (2H, m, C(2)PhC(2,6)*H*); **^{13}C NMR** (100 MHz, $CDCl_3$) δ_C:6.9 (CH_2CH_3), 18.9 (CH$(CH_3)_a(CH_3)_b$), 19.0 (CH$(CH_3)_a(CH_3)_b$), 28.1 (CH_2CH_3), 34.3 ($CH(CH_3)_2$), 67.1 (OCH_2Ph), 84.0 (*C*(2)), 125.1 (C(2)Ph*C*(3,5)H), 127.9 (C(2)Ph*C*(4)H), 128.1 (3C, OCH_2Ph*C*(2,6)H, OCH_2Ph*C*(4)H), 128.3 (OCH_2Ph*C*(3,5)H), 128.4 (C(2)Ph*C*(2,6)H), 135.4 (OCH_2Ph*C*(1)), 137.7 (C(2)Ph*C*(1)), 170.7 (*C*(O)OBn), 175.7 (*C*(O)*i*-Pr); **HRMS** (NSI^+) $C_{21}H_{28}NO_4^+$ ($[M+NH_4]^+$) requires 358.2013; found 358.2016 (+0.9 ppm).

Benzyl 2-phenyl-2-(propionyloxy)butanoate S43

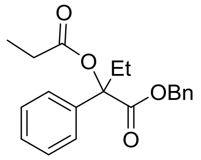

Following general procedure F, benzyl 2-hydroxy-2-phenylbutanoate **21** (73 mg, 0.27 mmol), propionic anhydride (138 µL, 1.08 mmol), DMAP (3.3 mg, 0.027 mmol, 10 mol%) and NEt_3 (226 µL, 1.62 mmol) were reacted in CH_2Cl_2 (2 mL) to give the crude product, which was purified by column chromatography (Isolera 4, Et_2O in petrol, 0% to 7% over 35CV; R_F 0.19 for Petrol/Et_2O 9:1) to give benzyl 2-phenyl-2-(propionyloxy)butanoate **S43** as a colourless oil (56 mg, 0.17 mmol, 64%); **IR** ν_{max} (film): 2980, 1738 (C=O), 1449, 1230, 1175; **^{1}H NMR** (500 MHz, $CDCl_3$) δ_H: 0.65 (3H, t, *J* 7.5, 3H, C(2)CH_2CH_3), 1.19 (3H, t, *J* 7.6, C(O)CH_2CH_3), 2.37 (1H, dq, *J* 15.0, 7.5, C(2)$CH_aH_bCH_3$), 2.49 (2H, 2 x q, *J* 7.6, C(O)CH_2CH_3), 2.73 (1H, dq, *J* 14.9, 7.4, C(2)$CH_aH_bCH_3$), 5.07 (1H, d, *J* 12.4, OCH_aH_bPh), 5.13 (1H, d, *J* 12.4, OCH_aH_bPh), 7.14-7.18 (2H, m, OCH_2PhC(3,5)*H*), 7.26-7.37 (6H, m, C(2)PhC(3,4,5)*H*, OCH2Ph(2,4,6)*H*), 7.46-7.49 (2H, m, , C(2)PhC(2,6)*H*); **^{13}C NMR** (125 MHz, $CDCl_3$) δ_C: δ 7.2 (C(2)CH_2CH_3), 9.3 (C(O)CH_2CH_3), 28.0 (C(2)CH_2CH_3), 28.3 (C(O)CH_2CH_3), 67.3 (OCH_2Ph), 84.4 (*C*(2)), 125.3 (2 xPh*C*H), 128.1 (Ph*C*H), 128.2 (2 x Ph*C*H), 128.3 (Ph*C*H), 128.5 (2 x Ph*C*H), 128.6 (2 x Ph*C*H), 135.7 (Ph*C*(1)), 137.7 (Ph*C*(1)), 171.0 (*C*(O)OBn), 173.0 (*C*(O)Et); **HRMS** (ESI^+) $C_{20}H_{22}O_4Na^+$ ($[M+Na]^+$) requires 349.1410; found 349.1406 (–1.2 ppm).

Benzyl 2-(isobutyryloxy)-2-phenylhexanoate S44

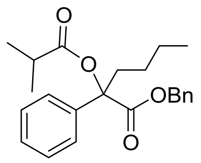

Following general procedure F, benzyl 2-hydroxy-2-phenylhexanoate **22** (48 mg, 0.16 mmol), isobutyric anhydride (106 µL, 0.64 mmol), DMAP (2 mg, 0.016 mmol, 10 mol%) and NEt_3 (133 µL, 0.96 mmol) were reacted in CH_2Cl_2 (1 mL) to give the crude product, which was purified by column chromatography (eluent Petrol/Et_2O, 9:1; R_F 0.21) to give benzyl 2-(isobutyryloxy)-2-phenylhexanoate **S44** as a colourless oil (45 mg, 0.12 mmol, 77%); **IR** ν_{max} (film): 2961, 1736 (C=O), 1466, 1225, 1152; **^{1}H NMR** (400 MHz, $CDCl_3$) δ_H: 0.78 (3H, t, *J* 7.3, C(6)H_3), 0.89-1.09 (2H, m, C(4)H_2), 1.13-1.28 (8H, m, CH(CH_3)$_2$, C(5)H_2), 2.34 (1H, ddd, *J* 14.6, 12.0, 4.6, C(3)H_aH$_b$), 2.62-2.75 (2H, m, C*H*(CH$_3$)$_2$, C(3)H$_a$$H_b$), 5.02 (1H, d, *J* 12.3, OCH_aH$_b$Ph), 5.14 (1H, d, *J* 12.3, OCH$_a$$H_b$Ph), 7.13-7.19 (2H, m, OCH$_2$PhC(2,6)*H*), 7.28-7.37 (6H, m, OCH$_2$PhC(3,4,5,)*H*, C(2)PhC(3,4,5)*H*), 7.48-7.53 (2H, m, C(2)PhC(2,6)*H*); **^{13}C NMR** (100 MHz, $CDCl_3$) δ_C: 13.9 (*C*(6)H$_3$), 18.9 (CH(*C*H$_3$)$_a$(CH$_3$)$_b$), 19.0 (CH(CH$_3$)$_a$(*C*H$_3$)$_b$), 22.5 (*C*(5)H$_2$), 24.8 (*C*(4)H$_2$), 34.3 (*C*H(CH$_3$)$_2$), 34.7 (*C*(3)H$_2$), 67.1 (O*C*H$_2$Ph), 83.7 (*C*(2)), 125.0 (C(2)Ph*C*(3,5)H), 127.9 (C(2)Ph*C*(4)H), 128.1 (OCH$_2$Ph*C*(2,6)H), 128.3 (OCH$_2$Ph*C*(3,5)H), 128.4 (3C, OCH$_2$Ph*C*(4)H, C(2)Ph*C*(2,6)H), 135.4 (OCH$_2$Ph*C*(1)), 138.1 (C(2)Ph*C*(1)), 170.9 (*C*(O)OBn), 175.9 (*C*(O)*i*-Pr); **HRMS** (NSI$^+$) $C_{23}H_{32}NO_4^+$ ([M+NH$_4$]$^+$) requires 386.2326; found 386.2324 (–0.5 ppm).

Benzyl 2-phenyl-2-(propionyloxy)hexanoate S45

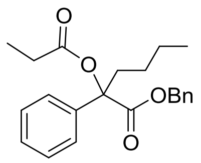

Following general procedure F, benzyl 2-hydroxy-2-phenylhexanoate **22** (80 mg, 0.27 mmol), propionic anhydride (138 µL, 1.08 mmol), DMAP (3.3 mg, 0.027 mmol, 10 mol%) and NEt_3 (226 µL, 1.62 mmol) were reacted in CH_2Cl_2 (2 mL) to give the crude product, which was purified by column chromatography (Isolera 4, Et_2O in petrol, 0% to 8% over 35CV; R_F 0.19 for Petrol/Et_2O 9:1) to give benzyl 2-phenyl-2-(propionyloxy)hexanoate **S45** as a colourless oil (77 mg, 0.22 mmol, 81%); **IR** ν_{max} (film): 2956, 1740 (C=O), 1497, 1449, 1227, 1172; **^{1}H NMR** (500 MHz, $CDCl_3$) δ_H: 0.79 (3H, t, *J* 7.3, C(6)CH$_3$), 0.89-1.09 (2H, m, C(4)H_2), 1.15-1.30 (5H, m, C(O)CH$_2$CH_3, C(5)H_2), 2.34 (1H, ddd, *J* 14.6, 12.1, 4.5, C(3)H_aH$_b$), 2.48 (2H, 2 x q, *J* 7.6, Hz, C(O)CH_2CH$_3$), 2.68 (1H, ddd, *J* 14.6, 12.1, 5.0, C(3)H$_a$$H_b$), 5.06 (1H, d, *J* 12.4, OCH_aH$_b$Ph), 5.12 (1H, d, *J* 12.4, OCH$_a$$H_b$Ph), 7.13-7.17 (2H, m, OCH$_2$PhC(2,6)*H*), 7.26-7.37 (6H, m, OCH$_2$PhC(3,4,5,)*H*, C(2)PhC(3,4,5)*H*), 7.46-7.50 (2H, m, C(2)PhC(2,6)*H*); **^{13}C NMR** (125 MHz, $CDCl_3$) δ_C: 9.3 (C(O)CH$_2$*C*H$_3$), 14.1 (*C*(6)H$_3$), 22.7 (*C*(5)H$_2$), 25.0 (*C*(4)H$_2$), 28.0 (C(O)*C*H$_2$CH$_3$), 35.0 (*C*(3)H$_2$), 67.3 (O*C*H$_2$Ph), 84.1 (*C*(2)), 125.2 (2 x Ph*C*H), 128.1 (Ph*C*H), 128.1 (2 x Ph*C*H), 128.2 (Ph*C*H), 128.5 (2 x Ph*C*H), 128.6 (2 x Ph*C*H), 135.7 (Ph*C*(1)), 138.1 (Ph*C*(1)), 171.0 (*C*(O)OBn), 173.0 (*C*(O)Et); **HRMS** (ESI$^+$) $C_{22}H_{26}O_4Na^+$ ([M+Na]$^+$) requires 377.1723; found 377.1720 (–0.9 ppm).

Benzyl 3,3,3-trifluoro-2-(isobutyryloxy)-2-phenylpropanoate S46

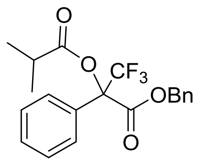

Following general procedure F, benzyl 3,3,3-trifluoro-2-hydroxy-2-phenylpropanoate **23** (50 mg, 0.16 mmol), isobutyric anhydride (106 µL, 0.64 mmol), DMAP (2 mg, 0.016 mmol, 10 mol%) and NEt_3 (133 µL, 0.96 mmol) were reacted in CH_2Cl_2 (1 mL) to give the crude product, which was purified by column chromatography (eluent Petrol/EtOAc, 4:1; R_F 0.66) to give benzyl 3,3,3-trifluoro-2-(isobutyryloxy)-2-phenylpropanoate **S46** as a colourless oil (56 mg, 0.15 mmol, 92%); **IR** ν_{max} (film): 2978, 1755 (C=O), 1452, 1273, 1177, 1020; ^{1}**H NMR** (400 MHz, $CDCl_3$) δ_H: 1.19 (3H, d, *J* 7.0, $CH(CH_3)_a(CH_3)_b$), 1.21 (3H, d, *J* 7.0, $CH(CH_3)_a(CH_3)_b$), 2.76 (1H, app hept, *J* 7.0, $CH(CH_3)_2$), 5.23 (1H, d, *J* 12.2, OCH_aH_bPh), 5.27 (1H, d, *J* 12.2, OCH_aH_bPh), 7.25-7.44 (8H, m, C(2)Ph(3,4,5)*H*, OCH_2PhCH), 7.54-7.59 (2H, m, C(2)PhC(2,6)*H*); ^{13}**C NMR** (125 MHz, $CDCl_3$) δ_C: 18.5 ($CH(CH_3)_a(CH_3)_b$), 18.6 ($CH(CH_3)_a(CH_3)_b$), 34.0 ($CH(CH_3)_2$), 68.3 (OCH_2Ph), 80.6 (*C*(2)), 122.2 (q, *J* 286, CF_3), 126.6 (C(2)Ph*C*(2,6)H), 128.4 (OCH_2PhC(2,6)H), 128.5 (OCH_2PhC(4)H), 128.6 (3C, OCH_2PhC(3,5)H, C(2)Ph*C*(4)H), 129.6 (C(2)Ph*C*(3,5)H), 131.1 (C(2)Ph*C*(1)), 134.4 (OCH_2PhC(1)), 164.5 (*C*(O)OBn), 173.7 (*C*(O)*i*-Pr); ^{19}**F NMR** (376 MHz, $CDCl_3$) δ_F: –73.7 (CF_3); **HRMS** (NSI^+) $C_{20}H_{23}NO_4F_3^+$ ($[M+NH_4]^+$) requires 398.1574; found 398.1569 (–1.2 ppm).

Benzyl 2-(isobutyryloxy)-2-phenylbut-3-enoate S47

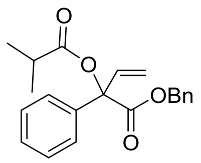

Following general procedure F, benzyl 2-hydroxy-2-phenylbut-3-enoate **24** (43 mg, 0.16 mmol), isobutyric anhydride (106 µL, 0.64 mmol), DMAP (2 mg, 0.016 mmol, 10 mol%) and NEt_3 (133 µL, 0.96 mmol) were reacted in CH_2Cl_2 (1 mL) to give the crude product, which was purified by column chromatography (eluent Petrol/Et_2O, 9:1; R_F 0.27) to give benzyl 2-(isobutyryloxy)-2-phenylbut-3-enoate **S47** as a colourless oil (49 mg, 0.14 mmol, 90%); **IR** ν_{max} (film): 2974, 1740 (C=O), 1449, 1233, 1140, 1049; ^{1}**H NMR** (400 MHz, $CDCl_3$) δ_H: 1.19 (3H, d, *J* 7.0, $CH(CH_3)_a(CH_3)_b$), 1.20 (3H, d, *J* 7.0, $CH(CH_3)_a(CH_3)_b$), 2.69 (1H, app hept, *J* 7.0, $CH(CH_3)_2$), 5.08 (1H, d, *J* 12.3, OCH_aH_bPh), 5.11 (1H, dd, *J* 17.5, 0.6, $CH=CH_{cis}H_{trans}$), 5.19 (1H, d, *J* 12.3, OCH_aH_bPh), 5.33 (1H, dd, *J* 10.9, 0.6, $CH=CH_{cis}H_{trans}$), 6.88 (1H, dd, *J* 17.5, 10.8, $CH=CH_2$), 7.17-7.24 (2H, m, OCH_2PhC(3,5)*H*), 7.27-7.37 (6H, m, OCH_2PhC(2,4,6)*H*, C(2)PhC(3,4,5)*H*), 7.48-7.54 (2H, m, C(2)PhC(2,6)*H*); ^{13}**C NMR** (100 MHz, $CDCl_3$) δ_C: 18.69 ($CH(CH_3)_a(CH_3)_b$), 18.72 ($CH(CH_3)_a(CH_3)_b$), 34.0 ($CH(CH_3)_2$), 67.5 (OCH_2Ph), 82.5 (*C*(2)), 117.4 ($CH=CH_2$), 126.1 (C(2)Ph*C*(2,6)H), 128.2 (OCH_2PhC(2,6)H), 128.3 (OCH_2PhC(3,5)H), 128.35 (C(2)Ph*C*(4)H), 128.39 (3C, OCH_2PhC(4)H, C(2)Ph*C*(3,5)H), 135.2 (OCH_2PhC(1)), 137.1 ($CH=CH_2$), 138.1 (C(2)Ph*C*(1)), 169.7 (*C*(O)OBn), 174.0 (*C*(O)*i*-Pr); **HRMS** (NSI^+) $C_{21}H_{26}NO_4^+$ ($[M+NH_4]^+$) requires 356.1856; found 356.1858 (+0.5 ppm).

Benzyl 4-cyclopropyl-2-(isobutyryloxy)-2-phenylbut-3-ynoate S48

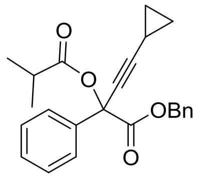

Following general procedure F, benzyl 4-cyclopropyl-2-hydroxy-2-phenybut-3-ynoate **25** (49 mg, 0.16 mmol), isobutyric anhydride (106 µL, 0.64 mmol), DMAP (2 mg, 0.016 mmol, 10 mol%) and NEt_3 (133 µL, 0.96 mmol) were reacted in CH_2Cl_2 (1 mL) to give the crude product, which was purified by column chromatography (eluent Petrol/EtOAc, 4:1; R_F 0.32) to give benzyl 4-cyclopropyl-2-(isobutyryloxy)-2-phenylbut-3-ynoate **S48** as a colourless oil (45 mg, 0.12 mmol, 75%); **IR** ν_{max} (film): 2974, 2245 (C≡C), 1744 (C=O), 1450, 1213, 1136; ^{1}**H NMR** (400 MHz, $CDCl_3$) δ_H: 0.72-0.84 (4H, m, cyclopropyl-(CH_2)$_2$), 1.21 (3H, d, *J* 7.0, CH(CH_3)$_a$(CH$_3$)$_b$), 1.22 (3H, d, *J* 7.0, CH(CH$_3$)$_a$(CH_3)$_b$), 1.33-1.41 (1H, m, cyclopropyl-C*H*), 2.67 (1H, app hept, *J* 7.0, C*H*(CH$_3$)$_2$), 5.12 (2H, app s, OCH$_2$Ph), 7.16-7.21 (2H, m, OCH$_2$PhC(2,6)*H*), 7.20-7.30 (3H, m, C(2)PhC(3,4,5)*H*), 7.30-7.39 (3H, m, OCH$_2$PhC(3,4,5,)*H*), 7.68-7.75 (2H, m, C(2)PhC(2,6)*H*); ^{13}**C NMR** (100 MHz, $CDCl_3$) δ_C: –0.23 (cyclopropyl-*C*H), 8.54 (cyclopropyl-(*C*H$_2$)$_2$), 18.6 (CH(*C*H$_3$)$_a$(CH$_3$)$_b$), 18.8 (CH(CH$_3$)$_a$(*C*H$_3$)$_b$), 34.0 (*C*H(CH$_3$)$_2$), 67.8 (O*C*H$_2$Ph), 70.8 (C(2)*C*≡C), 76.6 (*C*(2)), 92.5 (C(2)C≡*C*), 126.4 (C(2)Ph*C*(2,6)H), 127.7 (OCH$_2$Ph*C*(2,6)H), 128.1 (OCH$_2$Ph*C*(4)H), 128.4 (4C, OCH$_2$Ph*C*(3,5)H, C(2)Ph*C*(2,6)H), 129.0 (C(2)Ph*C*(4)H), 135.2 (C(2)Ph*C*(1)), 136.6 (OCH$_2$Ph*C*(1)), 167.6 (*C*(O)OBn), 175.0 (*C*(O)*i*-Pr); **HRMS** (NSI$^+$) $C_{24}H_{28}NO_4^+$ ([M+NH$_4$]$^+$) requires 394.2013; found 394.2008 (–1.2 ppm).

Benzyl 4-cyclopropyl-2-(isobutyryloxy)-2-methylbut-3-ynoate S49

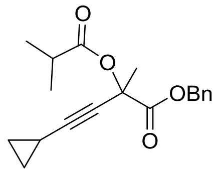

Following general procedure F, benzyl 4-cyclopropyl-2-hydroxy-2-methylbut-3-ynoate **26** (49 mg, 0.16 mmol), isobutyric anhydride (106 µL, 0.64 mmol), DMAP (2 mg, 0.016 mmol, 10 mol%) and NEt_3 (133 µL, 0.96 mmol) were reacted in CH_2Cl_2 (1 mL) to give the crude product, which was purified by column chromatography (eluent Petrol/EtOAc, 4:1; R_F 0.35) to give benzyl 4-cyclopropyl-2-(isobutyryloxy)-2-methylbut-3-ynoate **S49** as a colourless oil (41 mg, 0.13 mmol, 81%); **IR** ν_{max} (film): 2974, 2253 (C≡C), 1742 (C=O), 1456, 1223, 1113; ^{1}**H NMR** (400 MHz, $CDCl_3$) δ_H: 0.69-0.74 (2H, m, cyclopropyl-CH_2), 0.76-0.83 (2H, m, cyclopropyl-CH_2), 1.149 (3H, d, *J* 7.0, CH(CH_3)$_a$(CH$_3$)$_b$), 1.153 (3H, d, *J* 7.0, CH(CH$_3$)$_a$(CH_3)$_b$), 1.26-1.33 (1H, m, cyclopropyl-C*H*), 1.77 (3H, s, C(2)CH_3), 2.57 (1H, app hept, *J* 7.0, C*H*(CH$_3$)$_2$), 5.19 (1H, d, *J* 12.3, OCH_aH$_b$Ph), 5.23 (1H, d, *J* 12.3, OCH$_a$$H_b$Ph), 7.31-7.41 (5H, m, PhC*H*); ^{13}**C NMR** (125 MHz, $CDCl_3$) δ_C: –0.44 (cyclopropyl-*C*H), 8.43 (cyclopropyl-(*C*H$_2$)$_2$), 18.6 (CH(*C*H$_3$)$_a$(CH$_3$)$_b$), 18.7 (CH(CH$_3$)$_a$(*C*H$_3$)$_b$), 25.8 (C(2)*C*H$_3$), 33.7 (*C*H(CH$_3$)$_2$), 67.6 (O*C*H$_2$Ph), 71.2 (C(2)*C*≡C), 72.7 (*C*(2)), 90.3 (C(2)C≡*C*), 128.1 (Ph*C*(2,6)H), 128.3 (Ph*C*(4)H), 128.5 (Ph*C*(3,5)H), 135.4 (Ph*C*(1)), 168.9 (*C*(O)OBn), 175.3 (*C*(O)*i*-Pr); **HRMS** (NSI$^+$) $C_{19}H_{26}NO_4^+$ ([M+NH$_4$]$^+$) requires 332.1856; found 332.1860 (+1.1 ppm).

Benzyl 2-(isobutyryloxy)-2-methylbut-3-enoate S50

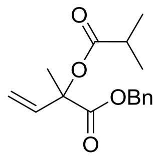

Following general procedure F, benzyl 2-hydroxy-2-methylbut-3-enoate **27** (33 mg, 0.16 mmol), isobutyric anhydride (106 µL, 0.64 mmol), DMAP (2 mg, 0.016 mmol, 10 mol%) and NEt_3 (133 µL, 0.96 mmol) were reacted in CH_2Cl_2 (1 mL) to give the crude product, which was purified by column chromatography (Isolera 4, Et_2O in petrol, 0% to 10% over 30 CV; R_F 0.40) to give benzyl 2-(isobutyryloxy)-2-methylbut-3-enoate **S50** as a colourless oil (27 mg, 0.10 mmol, 60%); **IR** ν_{max} (film): 2976 (C-H), 1740 (C=O), 1263 (C=C), 1121 (C-O); **^{1}H NMR** (500 MHz, $CDCl_3$) δ_H: 1.13 (6H, d, *J* 7.0, CH(C*H*$_3$)$_2$), 1.66 (3H, s, C(2)C*H*$_3$), 2.56 (1H, hept, *J* 7.0, C*H*(CH$_3$)$_2$), 5.15 (2H, s, OC*H*$_2$Ph), 5.24 (1H, d, *J* 10.5, CH=CH$_{cis}$*H*$_{trans}$), 5.36 (1H, d, *J* 17.5, CH=C*H*$_{cis}$H$_{trans}$), 6.19 (1H, dd, *J* 17.5, 10.8, C*H*=CH$_2$), 7.30-7.37 (m, 5H, PhC*H*); **^{13}C NMR** (125 MHz, $CDCl_3$) δ_C: 18.8 (CH(*C*H$_3$)(CH$_3$)), 18.8 (-CH(CH$_3$)(*C*H$_3$)), 22.9 (C(2)*C*H$_3$), 34.0 (*C*H(CH$_3$)$_2$), 67.4 (O*C*H$_2$Ph), 79.6 (*C*(2)), 115.9 (CH=*C*H$_2$), 128.4 (Ph*C*(4)H, 2 × Ph*C*H), 128.6 (2 × Ph*C*H), 135.6 (Ph*C*(1)), 136.9 (*C*H=CH$_2$), 170.9 (*C*(O)OBn), 175.7 (*C*(O)*i*-Pr); **HRMS** (ESI$^+$) $C_{16}H_{20}NaO_4^+$ ([M+Na]$^+$) requires 299.1254; found 299.1245 (–3.0 ppm).

Benzyl 2-(isobutyryloxy)-2,4-dimethylpent-3-enoate S51

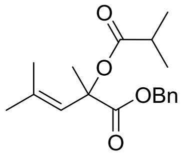

Following general procedure F, benzyl 2-hydroxy-2,4-dimethylpent-3-enoate **28** (38 mg, 0.16 mmol), isobutyric anhydride (106 µL, 0.64 mmol), DMAP (2 mg, 0.016 mmol, 10 mol%) and NEt_3 (133 µL, 0.96 mmol) were reacted in CH_2Cl_2 (1 mL) to give the crude product, which was purified by column chromatography (Isolera 4, Et_2O in petrol, 0% to 15% over 25 CV; R_F 0.58 (20% Et_2O in petrol)) to give benzyl 2-(isobutyryloxy)-2,4-dimethylpent-3-enoate **S51** as a colourless oil (43 mg, 0.10 mmol, 62%); **IR** ν_{max} (film): 2974 (C-H), 1734 (C=O), 1454 (C=C), 1256 (C=C), 1110 (C-O); **^{1}H NMR** (500 MHz, $CDCl_3$) δ_H: 1.11 (6H, 2 × d, *J* 7.0, CH(C*H*$_3$)$_2$), 1.69 (3H, s, C(2)C*H*$_3$), 1.72 (3H, d, *J* 1.3, C(4)(CH$_3$)$_a$(C*H*$_3$)$_b$), 1.74 (3H, d, *J* 1.3, C(4)(C*H*$_3$)$_a$(CH$_3$)$_b$), 2.54 (1H, hept, *J* 7.0, C*H*(CH$_3$)$_2$), 5.13 (1H, d, *J* 12.1, OC*H*$_a$H$_b$Ph), 5.16 (1H, d, *J* 12.1, OCH$_a$*H*$_b$Ph), 5.64 (1H, m, C(3)*H*), 7.29-7.36 (5H, m, PhC*H*); **^{13}C NMR** (125 MHz, $CDCl_3$) δ_C: 18.9 (CH(*C*H$_3$)$_2$), 19.1 (C(4)(*C*H$_3$)$_a$(CH$_3$)$_b$), 24.2 (C(2)*C*H$_3$), 27.2 (C(4)(CH$_3$)$_a$(*C*H$_3$)$_b$), 34.1 (*C*H(CH$_3$)$_2$), 67.2 (O*C*H$_2$Ph), 79.2 (*C*(2)), 123.6 (*C*(3)H), 128.3 (Ph*C*(4)H)), 128.4 (2 × Ph*C*H), 128.6 (2 × Ph*C*H), 135.7 (Ph*C*(1)), 137.3 (*C*(4)), 171.7 (*C*(O)OBn), 175.7 (*C*(O)*i*-Pr); **HRMS** (ESI$^+$) $C_{18}H_{24}NaO_4^+$ ([M+Na]$^+$) requires 327.1567; found 327.1564 (–0.9 ppm).

Benzyl 2-(isobutyryloxy)-2,3-dimethylbut-3-enoate S52

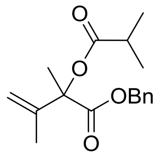

Following general procedure F, benzyl 2-hydroxy-2,3-dimethylbut-3-enoate **29** (35 mg, 0.16 mmol), isobutyric anhydride (106 µL, 0.64 mmol), DMAP (2 mg, 0.016 mmol, 10 mol%) and NEt_3 (133 µL, 0.96 mmol) were reacted in CH_2Cl_2 (1 mL) to give the crude product, which was purified by column chromatography (Isolera 4, Et_2O in petrol, 0% to 10% over 30 CV; R_F 0.40) to give benzyl 2-(isobutyryloxy)-2,3-dimethylbut-3-enoate **S52** as a colourless oil (23 mg, 0.08 mmol, 48%); **IR** ν_{max} (film): 2976 (C-H), 1740 (C=O), 1254 (C=C), 1117 (C-O); **^{1}H NMR** (500 MHz, $CDCl_3$) δ_H: 1.13 (6H, app. t, *J* 6.7, CH(C*H$_3$*)$_2$), 1.73 (3H, s, C(2)C*H$_3$*), 1.79 (3H, s, C(3)C*H$_3$*), 2.57 (1H, app hept, *J* 6.9, C*H*(CH$_3$)$_2$), 4.99 (1H, s, C(4)*H$_a$*H$_b$), 5.11 (1H, d, *J* 12.3, OC*H*$_a$H$_b$Ph), 5.15-5.17 (2H, m, OCH$_a$*H$_b$*Ph, C(4)H$_a$*H$_b$*), 7.29-7.36 (5H, m, PhC*H*); **^{13}C NMR** (125 MHz, $CDCl_3$) δ_C: 18.7 (C(3)*C*H$_3$), 18.8 (CH(*C*H$_3$)(CH$_3$)), 18.9 (CH(CH$_3$)(*C*H$_3$)), 21.5 (C(2)*C*H$_3$), 34.3 (*C*H) 67.3 (O*C*H$_2$Ph), 82.4 (*C*(2)), 113.0 (*C*(4)H$_2$), 128.4 (Ph*C*(4)H), 128.5 (2 × Ph*C*H), 128.6 (2 × Ph*C*H), 135.6 (Ph*C*(1)), 143.2 (*C*(3)), 170.5 (*C*(O)OBn), 175.5 (*C*(O)*i*-Pr); **HRMS** (ESI$^+$) $C_{17}H_{22}NaO_4^+$ ([M+Na]$^+$) requires 313.1410; found 313.1400 (–3.4 ppm).

Benzyl 2-cyclopentyl-2-(isobutyryloxy)propanoate S53

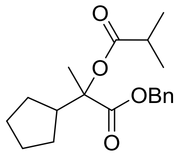

Following general procedure F, benzyl 2-cyclopentyl-2-hydroxypropanoate **30** (42 mg, 0.16 mmol), isobutyric anhydride (106 µL, 0.64 mmol), DMAP (2 mg, 0.016 mmol, 10 mol%) and NEt_3 (133 µL, 0.96 mmol) were reacted in CH_2Cl_2 (1 mL) to give the crude product, which was purified by column chromatography (Isolera 4, Et_2O in petrol, 0% to 10% over 25 CV; R_F 0.26) to give benzyl 2-cyclopentyl-2-(isobutyryloxy)propanoate **S53** as a colourless oil (15 mg, 0.05 mmol, 30%); **IR** ν_{max} (film): 2961 (C-H), 1738 (C=O), 1265 (C=C), 1155 (C-O); **^{1}H NMR** (500 MHz, $CDCl_3$) δ_H: 1.11 (3H, d, *J* 7.0, CH(C*H$_3$*)(CH$_3$)), 1.12 (3H, d, *J* 7.0, CH(CH$_3$)(C*H$_3$*)), 1.49-1.59 (7H, m, C$_5$*H$_9$*), 1.61 (3H, s, C(2)C*H$_3$*), 1.63-1.67 (1H, m, C$_5$*H$_9$*), 2.23-2.29 (1H, m, C$_5$*H$_9$*), 2.52 (1H, hept, *J* 7.0, C*H*(CH$_3$)$_2$), 5.11 (1H, d, *J* 12.5, OC*H*$_a$H$_b$Ph), 5.14 (1H, d, *J* 12.5, OCH$_a$*H$_b$*Ph) 7.24-7.37 (5H, m, PhC*H*); **^{13}C NMR** (125 MHz, $CDCl_3$) δ_C: 18.9 (CH(*C*H$_3$)(CH$_3$)), 18.9 (CH(CH$_3$)(*C*H$_3$)), 19.8 (C(2)*C*H$_3$), 25.6 (*C*H$_2$, *C*$_5$H$_9$), 25.7 (*C*H$_2$, *C*$_5$H$_9$), 26.9 (*C*H$_2$, *C*$_5$H$_9$), 27.0 (*C*H$_2$, *C*$_5$H$_9$), 34.3 (*C*H(CH$_3$)$_2$), 47.8 (*C*H, *C*$_5$H$_9$), 67.0 (O*C*H$_2$Ph), 81.9 (*C*(2)), 128.3 (Ph*C*(4)H), 128.5 (2 × Ph*C*H), 128.6 (2 × Ph*C*H), 135.8 Ph*C*(1)), 172.1 (*C*(O)OBn), 176.1 (*C*(O)*i*-Pr); **HRMS** (ESI$^+$) $C_{19}H_{26}NaO_4^+$ ([M+Na]$^+$) requires 341.1723; found 341.1715 (–2.4 ppm).

Benzyl 2-cyclohexyl-2-(isobutyryloxy)propanoate S54

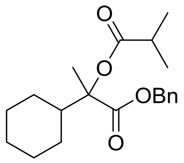

Following general procedure F, benzyl 2-cyclohexyl-2-hydroxypropanoate **31** (42 mg, 0.16 mmol), isobutyric anhydride (106 µL, 0.64 mmol), DMAP (2 mg, 0.016 mmol, 10 mol%) and NEt_3 (133 µL, 0.96 mmol) were reacted in Et_2O (1 mL) to give the crude product, which was purified by column chromatography (Isolera 4, Et_2O in petrol, 0% to 10% over 25 CV; R_F 0.2) to give benzyl 2-cyclohexyl-2-(isobutyryloxy)propanoate **S54** as a colourless oil (21 mg, 0.06 mmol, 40%); **IR** ν_{max} (film): 2934 (C-H), 1738 (C=O), 1454 (C=C), 1269 (C=C), 1115 (C-O); **^{1}H NMR** (500 MHz, $CDCl_3$) δ_H: 0.98-1.09 (2H, m, C_6H_{11}), 1.09-1.14 (7H, m, CH(CH_3)$_2$, C_6H_{11}), 1.16-1.25 (2H, C_6H_{11}), 1.53 (3H, s, C(2)CH_3), 1.56-1.57 (1H, m, C_6H_{11}), 1.64-1.67 (1H, m, C_6H_{11}), 1.71-1.82 (4H, m, C_6H_{11}), 2.52 (1H, hept, *J* 7.0, C*H*(CH_3)$_2$), 5.13 (2H, s, OCH_2Ph), 7.29-7.37 (5H, m, PhC*H*); **^{13}C NMR** (125 MHz, $CDCl_3$) δ_C: 17.7 (C(2)*C*H_3),18.9 (CH(*C*H_3)(CH_3)), 18.9 (CH(CH_3)(*C*H_3)), 26.4 (2 × *C*H_2, *C*$_6$$H_{11}$), 26.4 (*C*$H_2$, C_6H_{11}), 27.0 (*C*H_2, C_6H_{11}), 27.1 (*C*H_2, C_6H_{11}), 34.2 (*C*H(CH_3)$_2$), 45.8 (*C*H, C_6H_{11}), 66.9 (O*C*H_2Ph), 83.2 (*C*(2)), 128.3 (Ph*C*(4)H), 128.5 (2 × Ph*C*H), 128.6 (2 × Ph*C*H), 135.8 (Ph*C*(1)), 171.9 (*C*(O)OBn), 176.1 (*C*(O)*i*-Pr); **HRMS** (ESI$^+$) $C_{20}H_{28}NaO_4^+$ ([M+Na]$^+$) requires 355.1880; found 355.1871 (–2.5 ppm).

2-(Benzyloxy)-1-(4-methoxyphenyl)-2-oxo-1-phenylethyl isobutyrate S55

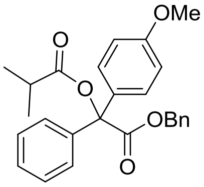

Following general procedure F, benzyl 2-hydroxy-2-(4-methoxyphenyl)-2-phenylacetate **S16** (56 mg, 0.16 mmol), isobutyric anhydride (106 µL, 0.64 mmol), DMAP (2 mg, 0.016 mmol, 10 mol%) and NEt_3 (133 µL, 0.96 mmol) were reacted in CH_2Cl_2 (1 mL) to give the crude product, which was purified by column chromatography (Isolera 4, Et_2O in petrol, 0% to 20% over 30 CV; R_F 0.44) to give 2-(benzyloxy)-1-(4-methoxyphenyl)-2-oxo-1-phenylethyl isobutyrate **S55** as a colourless oil (7.2 mg, 0.02 mmol, 11%); **IR** ν_{max} (film): 2972 (C-H), 1742 (C=O), 1510 (C=C), 1254 (C=C), 1173 (C-O); **^{1}H NMR** (500 MHz, $CDCl_3$) δ_H: 1.10 (3H, d, *J* 7.0, CH(CH_3)$_2$), 1.11 (3H, d, *J* 7.0, CH(CH_3)$_2$), 2.61 (1H, app hept, *J* 7.0, C*H*(CH_3)$_2$), 3.79 (3H, s, OCH_3), 5.15 (2H, s, CH_2), 6.80-6.83 (2H, m, C(2)ArC(3,5)*H*), 7.15-7.18 (2H, m, OCH_2PhC(2,6)*H*), 7.27-7.32 (6H, m, OCH_2PhC(3,4,5)*H*, C(2)PhC(3,4,5)*H*), 7.45-7.48 (2H, m, C(2)ArC(2,6)*H*), 7.52-7.56 (2H, m, C(2)PhC(2,6)*H*); **^{13}C NMR** (125 MHz, $CDCl_3$) δ_C: 18.7 (2C, CH(*C*H_3)$_2$), 34.3 (*C*H), 55.4 (O*C*H_3), 67.5 (*C*H_2), 83.9 (*C*(2)), 113.3 (C(2)Ar*C*(3,5)H), 127.8 (C(2)Ph*C*(2,6)H), 128.0 (C(2)Ph*C*(3,5)H), 128.1 (C(2)Ph*C*(4)H), 128.2 (OCH_2Ph*C*(2,6)H) 128.3 (OCH_2Ph*C*(4)H), 128.5 (OCH_2Ph*C*(3,5)H), 129.6 (C(2)Ar*C*(2,6)H), 132.3 (C(2)Ar*C*(1)), 135.4 (OCH_2Ph*C*(1)), 140.3 (C(2)Ph*C*(1)), 159.3 (C(2)Ar*C*(4)), 169.3 (*C*(O)OBn), 175.0 (*C*(O)*i*-Pr); **HRMS** (ESI$^+$) $C_{26}H_{26}NaO_5^+$ ([M+Na]$^+$) requires 441.1672; found 441.1659 (–3.0 ppm).

1-(Benzyloxy)-2-phenylpropan-2-yl acetate S56

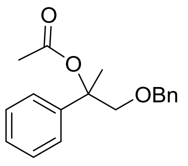

Following general procedure F, 1-(benzyloxy)-2-phenylpropan-2-ol **33** (48 mg, 0.2 mmol), acetic anhydride (76 µL, 0.8 mmol), DMAP (2.4 mg, 0.02 mmol, 10 mol%) and NEt_3 (167 µL, 1.2 mmol) were reacted in CH_2Cl_2 (2 mL) to give the crude product, which was purified by column chromatography (Isolera 4, Et_2O in petrol, 0% to 20% over 30CV) to give 1-(benzyloxy)-2-phenylpropan-2-yl acetate **S56** as a colourless oil (41 mg, 0.14 mmol, 71%); **IR** ν_{max} (film): 1734 (C=O), 1497, 1449, 1367, 1236; **^{1}H NMR** (500 MHz, $CDCl_3$) δ_H: 1.91 (3H, s, C(2)CH_3), 2.09 (3H, s, C(O)CH_3), 3.65 (1H, d, *J* 10.0, C(1)H_aH_b), 3.78 (1H, d, *J* 10.0, C(1)H_aH_b), 4.50 (1H, d, *J* 12.4, OCH_aH_bPh), 4.58 (1H, d, *J* 12.3, OCH_aH_bPh), 7.24-7.30 (4H, m, OCH_2PhC(2,4,6)*H*, C(2)PhC(4)*H*), 7.32-7.35 (6H, m, OCH_2PhC(3,5)*H*, C(2)PhC(2,3,5,6)*H*); **^{13}C NMR** (125 MHz, $CDCl_3$) δ_C: δ 22.3 (*C*(2)*C*H_3), 22.4 (C(O)*C*H_3), 73.6 (O*C*H_2Ph), 77.1 (*C*(1)H_2), 83.2 (*C*(2)), 125.1 (2 x Ph*C*H), 127.5 (Ph*C*H), 127.7 (2 x Ph*C*H), 127.8 (Ph*C*H), 128.4 (2 x Ph*C*H), 128.5 (2 x Ph*C*H), 138.3 (OCH_2Ph*C*(1)), 142.6 (C(2)Ph*C*(1)), 169.8 (*C*=O); **HRMS** (ESI^+) $C_{19}H_{20}NaO_4^+$ ($[M+Na]^+$) requires 307.1304; found 307.1303 (–0.4 ppm).

Benzyl 3-acetoxy-3-phenylbutanoate S57

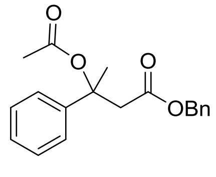

Following general procedure F, benzyl 3-hydroxy-3-phenylbutanoate **34** (73 mg, 0.27 mmol), acetic anhydride (102 µL, 1.08 mmol), DMAP (3.3 mg, 0.027 mmol, 10 mol%) and NEt_3 (226 µL, 1.62 mmol) were reacted in CH_2Cl_2 (2 mL) to give the crude product, which was purified by column chromatography (Isolera 4, Et_2O in petrol, 0% to 20% over 35CV) to give benzyl 3-acetoxy-3-phenylbutanoate **S57** as a colourless oil (67 mg, 0.22 mmol, 81%); **IR** ν_{max} (film): 1732 (C=O), 1497, 1449, 1236, 1153; **^{1}H NMR** (500 MHz, $CDCl_3$) δ_H: δ 1.92 (3H, s, C(3)CH_3), 1.99 (3H, s, C(O)CH_3), 3.19 (1H, d, *J* 14.1, C(2)H_aH_b), 3.29 (1H, d, *J* 14.1, C(2)H_aH_b), 5.00 (1H, d, *J* 12.3, OCH_aH_bPh), 5.06 (1H, d, *J* 12.3, OCH_aH_bPh), 7.21-7.24 (m, 2H, OCH_2PhC(2,6)*H*), 7.25-7.28 (m, 1H, C(3)PhC(4)*H*), 7.30-7.36 (m, 7H, OCH_2PhC(3,4,5)*H*, C(3)PhC(2,3,5,6)*H*); **^{13}C NMR** (125 MHz, $CDCl_3$) δ_C: δ 22.3 (C(O)*C*H_3), 26.4 (C(3)*C*H_3), 45.5 (*C*(2)H_2), 66.5 (O*C*H_2Ph), 81.2 (*C*(3)), 124.5 (2 x Ph*C*H), 127.6 (Ph*C*H), 128.4 (Ph*C*H), 128.4 (2 x Ph*C*H), 128.5 (2 x Ph*C*H), 128.7 (2 x Ph*C*H), 135.8 (OCH_2Ph*C*(1)), 143.9 (C(3)Ph*C*(1)), 169.6 (*C*(O)OBn), 169.8 (C(O)*C*H_3); **HRMS** (ESI^+) $C_{19}H_{20}NaO_4^+$ ($[M+Na]^+$) requires 335.1254; found 335.1250 (–1.1 ppm).

Catalytic Kinetic Resolution: Optimisation

Amide optimisation:

Table S1: Catalyst

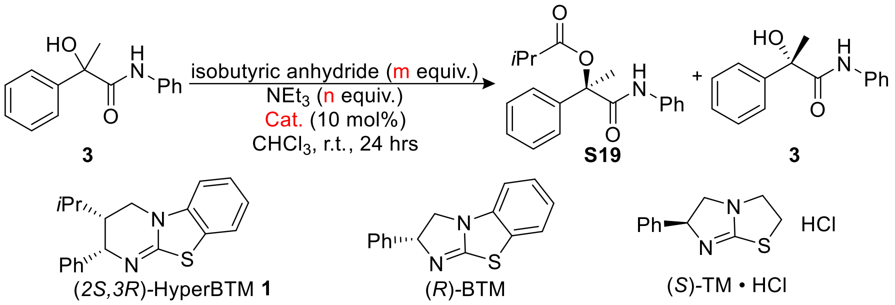

Entry	Cat.	Cat%	m	n	c	*s*
1	HyperBTM **1**	10	2	2	24	7
2	HyperBTM **1**	10	5	2	31	6
3	BTM	10	5	2	<1	-
4	TM·HCl	10	5	2	<1	-
5	HyperBTM **1**	10	10	0	3	3
6	HyperBTM **1**	0	5	2	<1	-

Table S2: Base

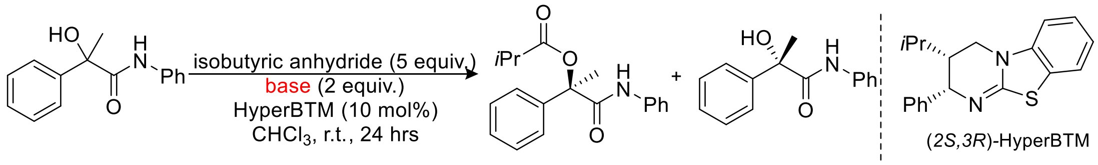

Entry	base	c	*s*	Entry	base	c	*s*
1	NEt_3	31	6	7	*i*Pr_2NEt	22	6
2	PS-BEMP	10	4	8	DABCO	3	3
3	TBD	2	3	9	2,6-lutidine	6	3
4	DBU	21	5	10	pyridine	8	3
5	TMP	56	7	11	Cs_2CO_3	9	6
6	*i*Pr_2NH	<1	-	12	K_3PO_4	19	4

BEMP: polymer-supported 2-*tert*-Butylimino-2-diethylamino-1,3-dimethylperhydro-1,3,2-diazaphosphorine;
TBD: 1,5,7-Triazabicyclo[4.4.0]dec-5-ene;
DBU: 1,8-Diazabicyclo[5.4.0]undec-7-ene
TMP: 2,2,6,6-Tetramethylpiperidine
DABCO: 1,4-diazabicyclo[2.2.2]octane

Table S3: Temperature

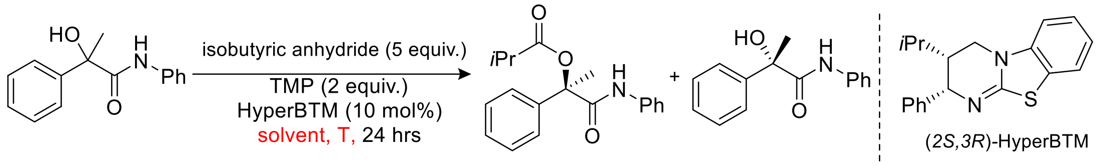

Entry	T/ °C	Solvent	c	*s*
1	r.t.	$CHCl_3$	56	7.1
2	r.t.	CH_2Cl_2	59	6.9
3	r.t.	Acetone	39	6.6
4	r.t.	DMF	49	5.9
5	r.t.	MeCN	70	6.3
6	0	$CHCl_3$	48	8.2
7	0	CH_2Cl_2	50	7.6
8	0	MeCN	72	6.7
9	−40	$CHCl_3$	18	5.4
10	−40	CH_2Cl_2	12	6.7
11	−40	MeCN	32	4.5

Table S4: Additives

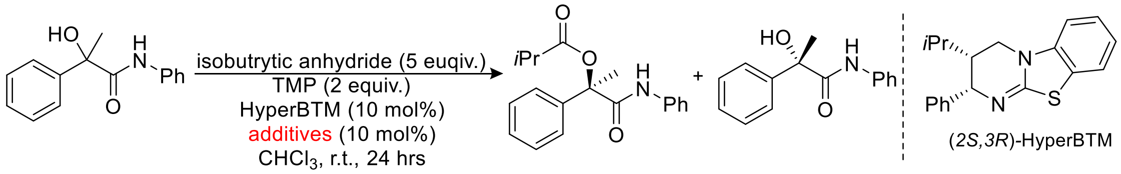

Entry	Additives	c	*s*
1	$IrCl_3{\cdot}xH_2O$	41	6.2
2	$FeCl_3$	52	7.0
3	$ZnCl_2$	56	7.1
4	$MgCl_2$	54	6.0
5	LiCl	57	6.5
6	TBAF	41	6.0

Table S5: Catalyst loading

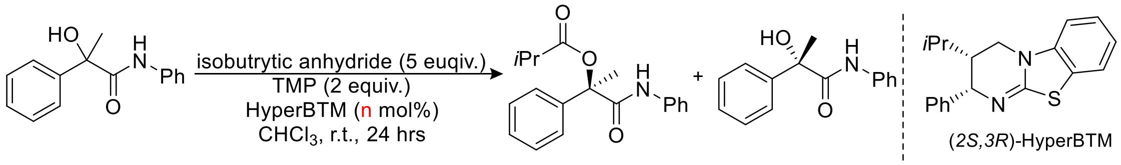

Entry	n	c	*s*
1	1	18	7.2
2	5	44	7.2
3	10	56	7.1

Table S6: KR of acyclic α-hydroxy amides

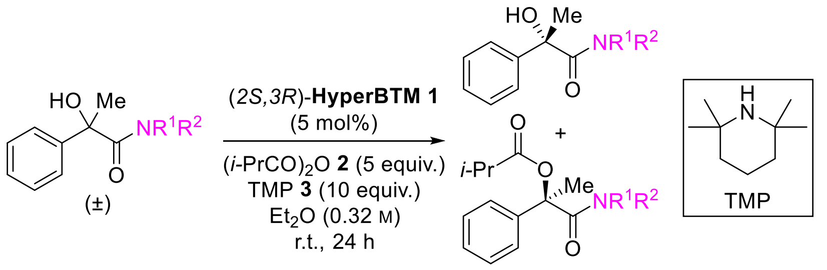

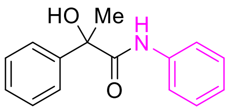

3;[a] c = 56; *s* = 7
alc: 85:15; 40%
est: 78:22; 52%

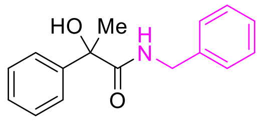

S3; c = 37; *s* = 4
alc: 64.5:35.5; 60%
est: 75:25; 33%

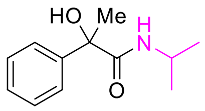

S4; c = 15; *s* = 2
alc: 53:47; 81%
est: 66:34; 10%

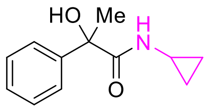

S5; c = 33; *s* = 4
alc: 63:37; 63%
est: 76:24; 30%

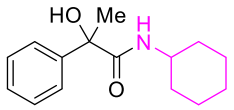

S6; c = 12; *s* = 2
alc: 52:48; 76%
est: 38:62; 10%

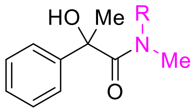

2;[b] R = Me, c = 0
S2;[b] R = OMe,c = 0

Conversion (c) and er determined by chiral HPLC analysis. *s* calculated using equations given in ref [12].
[a] 2 equiv. of TMP used; [b] 10 mol% (2*S*,3*R*)-HyperBTM at up to 50 °C

Ester optimisation:

Table S7: Solvent

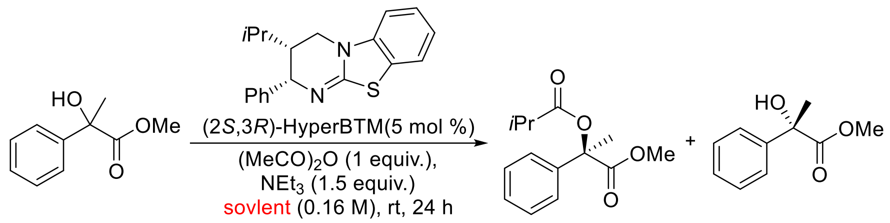

Entry	solvent	c	*s*	Entry	solvent	c	*s*
1	CH_2Cl_2	44	3	6	1,4-Dioxane	19	8
2	$CHCl_3$	39	5	7	MTBE	36	14
3	PhMe	43	12	8	EtOAc	41	9
4	Et_2O	43	15	9	DMC	37	8
5	THF	0	-	10	MeCN	25	3

MTBE: methyl *tert*-butyl ether; DMC: dimethyl carbonate

Table S8: Anhydride

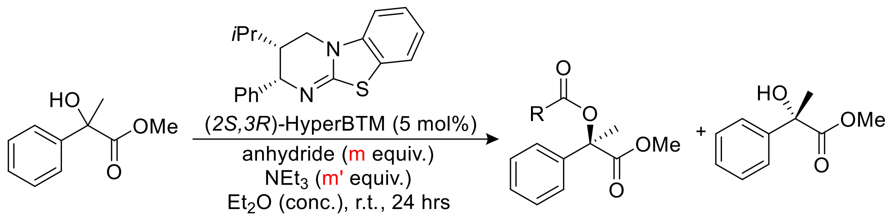

Entry	R	m	Conc. (M)	c	*s*
1	Me	1	0.16	43	15
2	Et	1	0.16	32	24
3	*i*Pr	1	0.16	32	60
4	*i*Pr	2	0.32	47	60

m' = 1.5 × m

Table S9: Ester

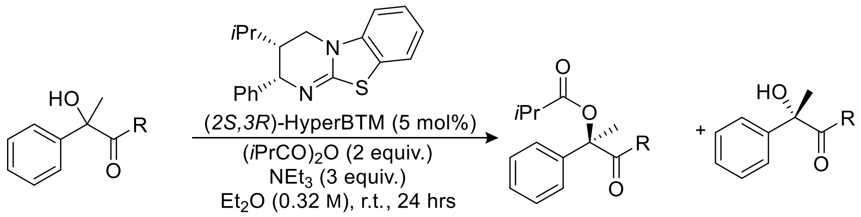

Entry	R	c	*s*
1	OMe	47	60
2	OEt	33	50
3	OBn	51	120

The esters were resolved under the final optimised conditions (without base) and shown in the paper, see SI KR section for details.

Table S10: Catalyst, temperature and base

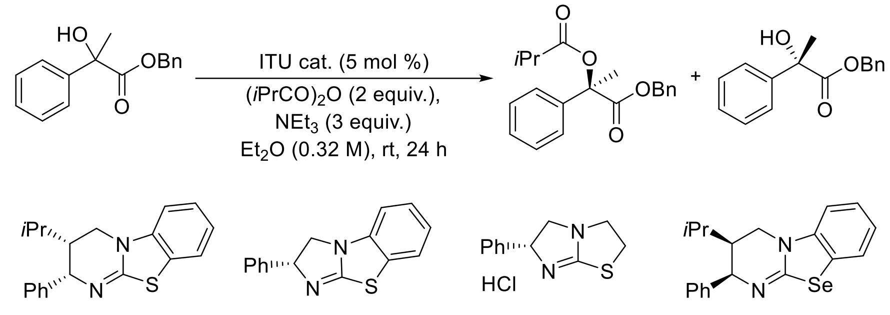

(2*S*,3*R*)-HyperBTM (*R*)-BTM tetramisole•HCl (2*S*,3*R*)-**9**

Entry	Cat	c	*s*
1	(2*S*,3*R*)-HyperBTM	51	120
2	(*R*)-BTM	<1	–
3	(*S*)-TM·HCl	<1	–
4[b]	(2*S*,3*R*)-HyperBTM	41	130
5[c]	(2*S*,3*R*)-HyperBTM	50	130
6[c,d]	(2*R*,3*S*)-**9**	49	120

(a) opposite enantiomers isolated; (b) reaction performed at 0 °C; (c) no base; (d) 2 mol% of catalyst, 1 equiv. of (*i*-PrCO)$_2$O. Alcohol and ester obtained in opposite enantiomeric series to that shown in scheme.

Catalytic Kinetic Resolution: Scope

Table S6: KR of 2-hydroxy-*N*,2-diphenylpropanamide 3

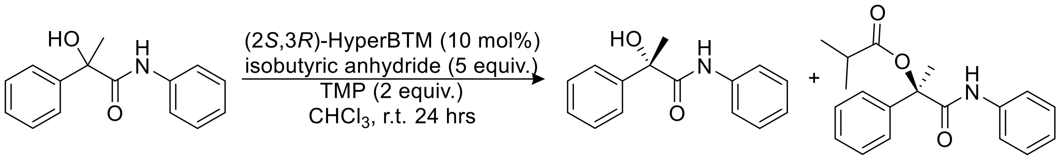

Following general procedure H, **2-hydroxy-*N*,2-diphenylpropanamide 3** (39 mg, 0.16 mmol), isobutyric anhydride (0.13 mL, 0.8 mmol), (2*S*,3*R*)-HyperBTM (5 mg, 0.016 mmol, 10 mol%) and TMP (55 µL, 0.32 mmol) in $CHCl_3$ (0.5 mL) for 24 h gave, after column chromatography (Isolera 4, EtOAc in petrol, 0% to 15% over 30 CV), alcohol (15 mg, 0.06 mmol, 40%) and ester (26 mg, 0.08 mmol, 52%). **c = 56%, *s* = 7**

(*S*)-2-Hydroxy-*N*,2-diphenylpropanamide (*S*)-3: $[\alpha]_D^{20}$ +17 (*c* 0.25, $CHCl_3$); Chiral HPLC Chiralpak AD-H (5% *i*PrOH:hexane, flow rate 1.0 mL min^{-1}, 211 nm, 30 °C) T_R: 17.2, 29.6 min, 84.9:15.1 er.

Racemic alcohol

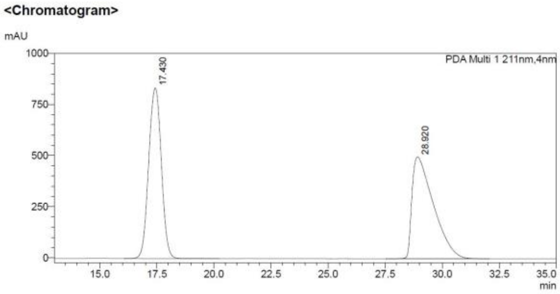

<Peak Table>

PDA Ch1 211nm

Peak#	Ret. Time	Area%
1	17.430	49.763
2	28.920	50.237
Total		100.000

Enantioenriched alcohol

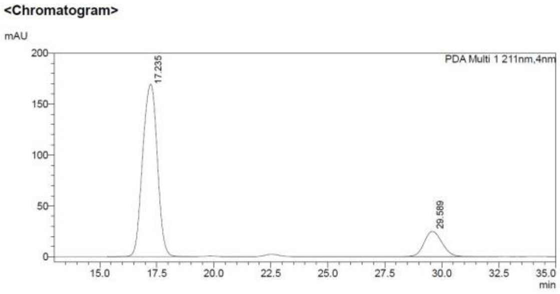

<Peak Table>

PDA Ch1 211nm

Peak#	Ret. Time	Area%
1	17.235	84.864
2	29.589	15.136
Total		100.000

(*R*)-1-Oxo-2-phenyl-1-(phenylamino)propan-2-yl isobutyrate (*R*)-S19: $[\alpha]_D^{20}$ −28 (*c* 0.25, $CHCl_3$); Chiral HPLC Chiralpak AD-H (5% *i*PrOH:hexane, flow rate 1.0 mL min^{-1}, 211 nm, 30 °C) T_R: 15.9, 19.8 min, 22.1:77.9 er.

Racemic ester

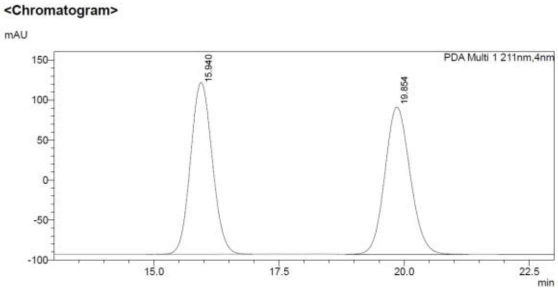

<Peak Table>

PDA Ch1 211nm

Peak#	Ret. Time	Area%
1	15.940	50.044
2	19.854	49.956
Total		100.000

Enantioenriched ester

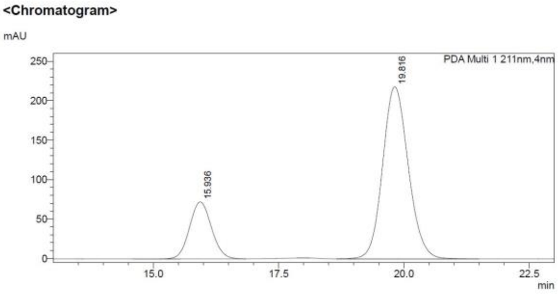

<Peak Table>

PDA Ch1 211nm

Peak#	Ret. Time	Area%
1	15.936	22.063
2	19.816	77.937
Total		100.000

Table S6: KR of *N*-benzyl-2-hydroxy-2-phenylpropanamide S3

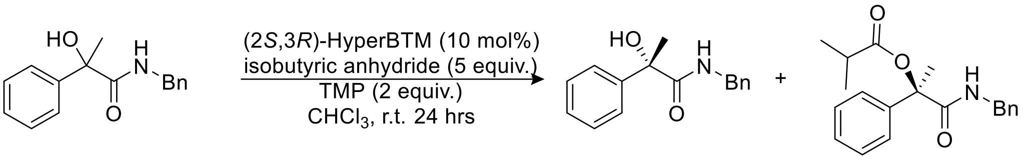

Following general procedure H, ***N*-benzyl-2-hydroxy-2-phenylpropanamide S3** (41 mg, 0.16 mmol), isobutyric anhydride (0.13 mL, 0.8 mmol), (2*S*,3*R*)-HyperBTM (5 mg, 0.016 mmol, 10 mol%) and TMP (0.27 mL, 1.6 mmol) in $CHCl_3$ (0.5 mL) for 24 h gave, after column chromatography (Isolera 4, EtOAc in petrol, 0% to 25% over 25 CV), alcohol (24 mg, 0.10 mmol, 60%) and ester (17 mg, 0.05 mmol, 33%). **c = 37%, *s* = 4**

(*S*)-*N*-Benzyl-2-hydroxy-2-phenylpropanamide (*S*)-S3: $[\alpha]_D^{20}$ −12 (*c* 0.25, $CHCl_3$); Chiral HPLC Chiralpak AD-H (10% *i*PrOH:hexane, flow rate 1.0 mL min^{-1}, 211 nm, 30 °C) T_R: 12.0, 21.9 min, 64.6:35.4 er.

Racemic alcohol

<Chromatogram>

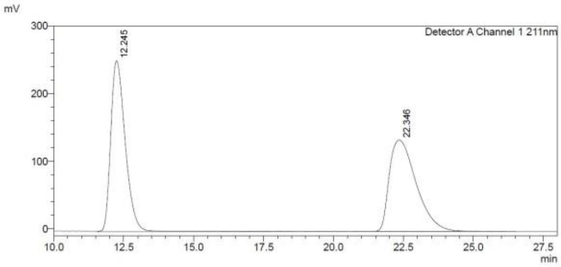

<Peak Table>

Detector A Channel 1 211nm

Peak#	Ret. Time	Area%
1	12.245	50.283
2	22.346	49.717
Total		100.000

Enantioenriched alcohol

<Chromatogram>

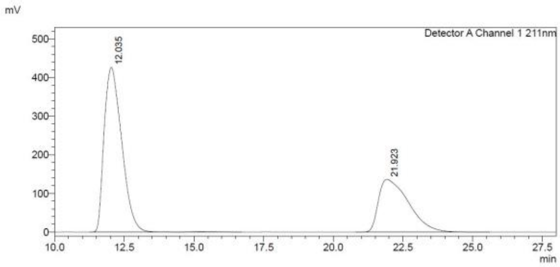

<Peak Table>

Detector A Channel 1 211nm

Peak#	Ret. Time	Area%
1	12.035	64.560
2	21.923	35.440
Total		100.000

(*R*)-1-(Benzylamino)-1-oxo-2-phenylpropan-2-yl isobutyrate (*R*)-S20: $[\alpha]_D^{20}$ +5 (*c* 0.25, $CHCl_3$); Chiral HPLC Chiralpak AD-H (5% *i*PrOH:hexane, flow rate 1.0 mL min^{-1}, 211 nm, 30 °C) T_R: 17.6, 25.4 min, 25.2:74.8 er.

Racemic ester

<Chromatogram>

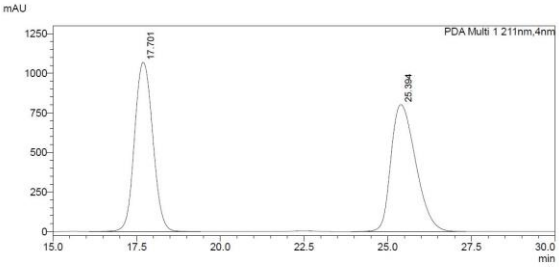

<Peak Table>

PDA Ch1 211nm

Peak#	Ret. Time	Area%
1	17.701	49.555
2	25.394	50.445
Total		100.000

Enantioenriched ester

<Chromatogram>

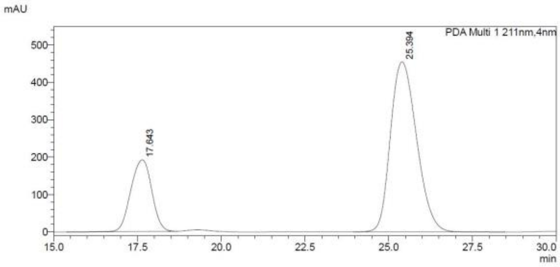

<Peak Table>

PDA Ch1 211nm

Peak#	Ret. Time	Area%
1	17.643	25.185
2	25.394	74.815
Total		100.000

***** the HPLC traces of the amide containing alcohol showed unusual peak broadening (presumably due to rotameric species) but we believe allow unambiguous determination of enantiomeric ratios.

Table S6: KR of 2-hydroxy-*N*-isopropyl-2-phenylpropanamide S4

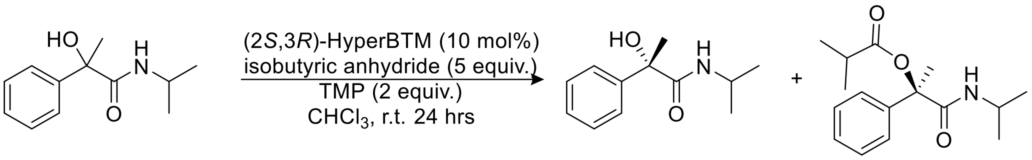

Following general procedure H, **2-hydroxy-*N*-isopropyl-2-phenylpropanamide S4** (33 mg, 0.16 mmol), isobutyric anhydride (0.13 mL, 0.8 mmol), (2*S*,3*R*)-HyperBTM (5 mg, 0.016 mmol, 10 mol%) and TMP (0.27 mL, 1.6 mmol) in $CHCl_3$ (0.5 mL) for 24 h gave, after column chromatography (Isolera 4, EtOAc in petrol, 0% to 25% over 30 CV), alcohol (27 mg, 0.13 mmol, 81%) and ester (4 mg, 0.02 mmol, 10%). **c = 15%, *s* = 2.0**

(*S*)-2-Hydroxy-*N*-isopropyl-2-phenylpropanamide (*S*)-S4: $[\alpha]_D^{20}$ −1 (*c* 0.93, $CHCl_3$); Chiral HPLC Chiralpak AD-H (2% *i*PrOH:hexane, flow rate 1.0 mL min^{-1}, 211 nm, 30 °C) T_R: 22.5, 32.5 min, 52.8:47.2 er.

Racemic alcohol

<Chromatogram>

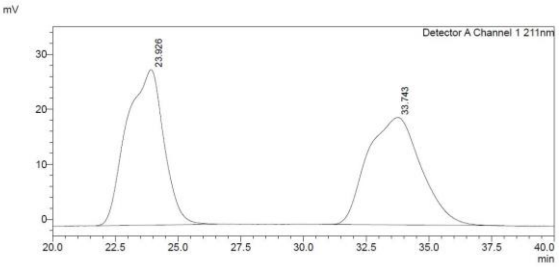

<Peak Table>

Detector A Channel 1 211nm

Peak#	Ret. Time	Area%
1	23.926	50.136
2	33.743	49.864
Total		100.000

Enantioenriched alcohol

<Chromatogram>

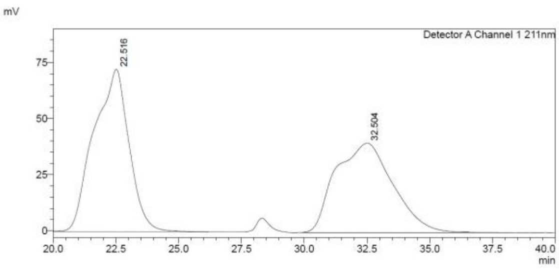

<Peak Table>

Detector A Channel 1 211nm

Peak#	Ret. Time	Area%
1	22.516	52.805
2	32.504	47.195
Total		100.000

(*R*)-1-(Benzylamino)-1-oxo-2-phenylpropan-2-yl isobutyrate (*R*)-S21: $[\alpha]_D^{20}$ −5 (*c* 0.1, $CHCl_3$); Chiral HPLC Chiralpak AD-H (1% *i*PrOH:hexane, flow rate 1.0 mL min^{-1}, 211 nm, 30 °C) T_R: 16.2, 21.5 min, 34.2:65.8 er.

Racemic ester

<Chromatogram>

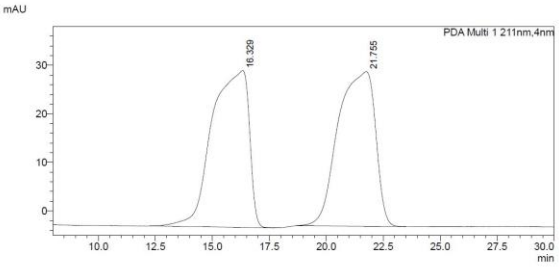

<Peak Table>

PDA Ch1 211nm

Peak#	Ret. Time	Area%
1	16.329	50.275
2	21.755	49.725
Total		100.000

Enantioenriched ester

<Chromatogram>

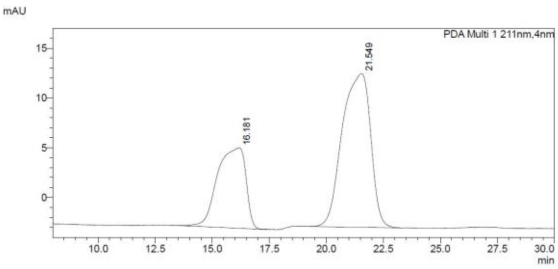

<Peak Table>

PDA Ch1 211nm

Peak#	Ret. Time	Area%
1	16.181	34.248
2	21.549	65.752
Total		100.000

***** the HPLC traces of the amide containing alcohol and ester showed unusual peak broadening (presumably due to rotameric species) but we believe allow unambiguous determination of enantiomeric ratios.

Table S6: KR of *N*-cyclopropyl-2-hydroxy-2-phenylpropanamide S5

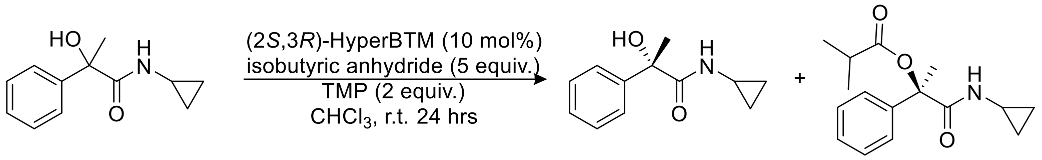

Following general procedure H, ***N*-cyclopropyl-2-hydroxy-2-phenylpropanamide S5** (33 mg, 0.16 mmol), isobutyric anhydride (0.13 mL, 0.8 mmol), (2*S*,3*R*)-HyperBTM (5 mg, 0.016 mmol, 10 mol%) and TMP (0.27 mL, 1.6 mmol) in $CHCl_3$ (0.5 mL) for 24 h gave, after column chromatography (Isolera 4, EtOAc in petrol, 0% to 30% over 30 CV), alcohol (27 mg, 0.13 mmol, 63%) and ester (13 mg, 0.05 mmol, 30%). **c = 33%, *s* = 4**

(*S*)-*N*-Cyclopropyl-2-hydroxy-2-phenylpropanamide (*S*)-S5: $[\alpha]_D^{20}$ −4 (*c* 0.25, $CHCl_3$); Chiral HPLC Chiralpak AD-H (5% *i*PrOH:hexane, flow rate 1.0 mL min^{-1}, 211 nm, 30 °C) T_R: 14.9, 19.5 min, 62.8:37.2 er.

Racemic alcohol

<Chromatogram>

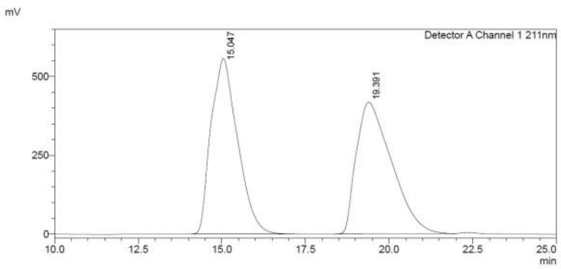

<Peak Table>

Detector A Channel 1 211nm

Peak#	Ret. Time	Area%
1	15.047	50.104
2	19.391	49.896
Total		100.000

Enantioenriched alcohol

<Chromatogram>

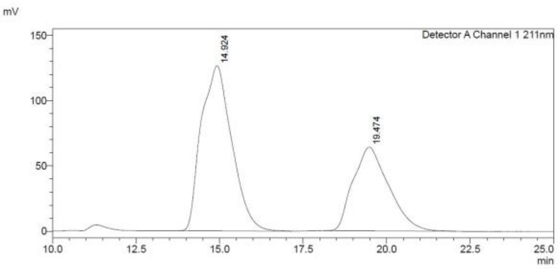

<Peak Table>

Detector A Channel 1 211nm

Peak#	Ret. Time	Area%
1	14.924	62.803
2	19.474	37.197
Total		100.000

(*R*)-1-(Cyclopropylamino)-1-oxo-2-phenylpropan-2-yl isobutyrate (*R*)-S22: $[\alpha]_D^{20}$ −22 (*c* 0.25, $CHCl_3$); Chiral HPLC Chiralcel OJ-H (0.5% *i*PrOH:hexane, flow rate 1.0 mL min^{-1}, 211 nm, 30 °C) T_R: 43.4, 52.3 min, 24.2:75.8 er.

Racemic ester

<Chromatogram>

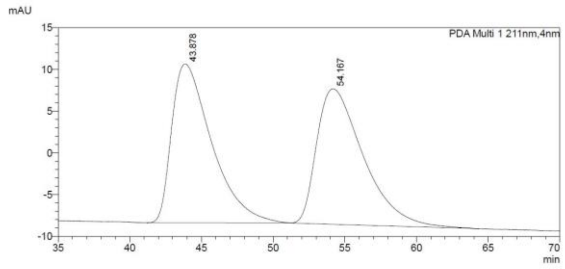

<Peak Table>

PDA Ch1 211nm

Peak#	Ret. Time	Area%
1	43.878	50.100
2	54.167	49.900
Total		100.000

Enantioenriched ester

<Chromatogram>

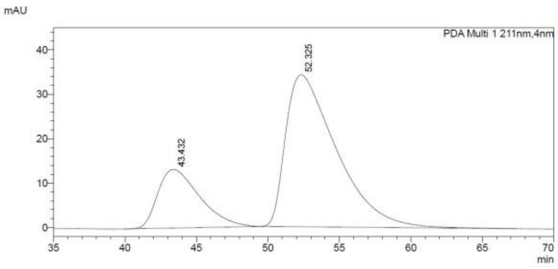

<Peak Table>

PDA Ch1 211nm

Peak#	Ret. Time	Area%
1	43.432	24.169
2	52.325	75.831
Total		100.000

***** the HPLC traces of the amide containing alcohol showed unusual peak broadening (presumably due to rotameric species) but we believe allow unambiguous determination of enantiomeric ratios.

Table S6: KR of *N*-cyclohexyl-2-hydroxy-2-phenylpropanamide S6

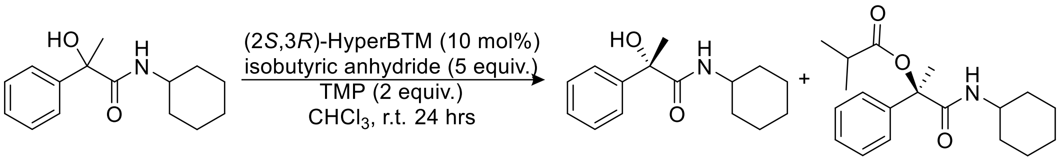

Following general procedure H, ***N*-cyclohexyl-2-hydroxy-2-phenylpropanamide S6** (40 mg, 0.16 mmol), isobutyric anhydride (0.13 mL, 0.8 mmol), (2*S*,3*R*)-HyperBTM (5 mg, 0.016 mmol, 10 mol%) and TMP (0.27 mL, 1.6 mmol) in $CHCl_3$ (0.5 mL) for 24 h gave, after column chromatography (Isolera 4, EtOAc in petrol, 0% to 15% over 30 CV), alcohol (30 mg, 0.12 mmol, 76%) and ester (5 mg, 0.02 mmol, 10%). **c = 12%, *s* = 2**

(*S*)-*N*-Cyclohexyl-2-hydroxy-2-phenylpropanamide (*S*)-S6: $[\alpha]_D^{20}$ −2 (*c* 0.25, $CHCl_3$); Chiral HPLC Chiralpak AD-H (5% *i*PrOH:hexane, flow rate 1.0 mL min^{-1}, 211 nm, 30 °C) T_R: 11.2, 17.1 min, 51.7:48.3 er.

Racemic alcohol

<Chromatogram>

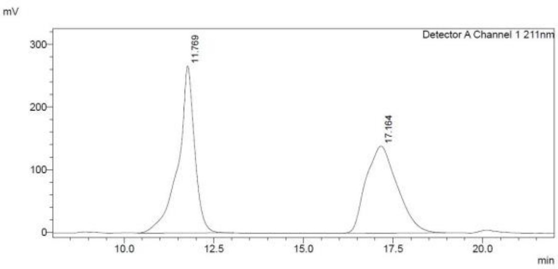

<Peak Table>

Detector A Channel 1 211nm

Peak#	Ret. Time	Area%
1	11.769	50.338
2	17.164	49.662
Total		100.000

Enantioenriched alcohol

<Chromatogram>

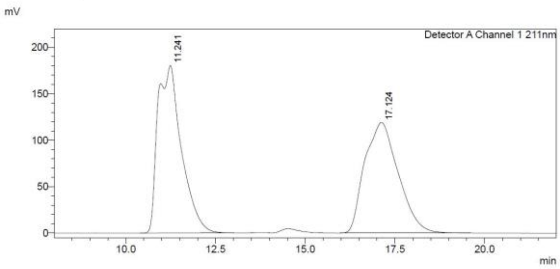

<Peak Table>

Detector A Channel 1 211nm

Peak#	Ret. Time	Area%
1	11.241	51.651
2	17.124	48.349
Total		100.000

(*R*)-1-(Cyclohexylamino)-1-oxo-2-phenylpropan-2-yl isobutyrate (*R*)-S23: $[\alpha]_D^{20}$ −2 (*c* 0.1, $CHCl_3$); Chiral HPLC Chiralcel OJ-H (2% *i*PrOH:hexane, flow rate 1.0 mL min^{-1}, 211 nm, 30 °C) T_R: 15.1, 19.0 min, 37.8:62.2 er.

Racemic ester

<Chromatogram>

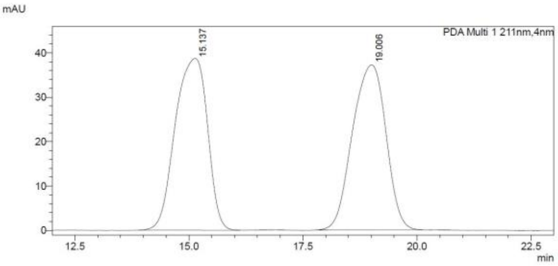

<Peak Table>

PDA Ch1 211nm

Peak#	Ret. Time	Area%
1	15.137	50.063
2	19.006	49.937
Total		100.000

Enantioenriched ester

<Chromatogram>

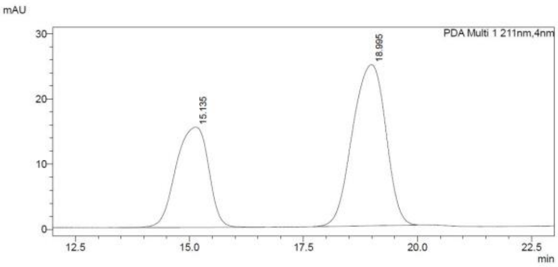

<Peak Table>

PDA Ch1 211nm

Peak#	Ret. Time	Area%
1	15.135	37.830
2	18.995	62.170
Total		100.000

***** the HPLC traces of the amide containing alcohol showed unusual peak broadening (presumably due to rotameric species) but we believe allow unambiguous determination of enantiomeric ratios.

Table 1: KR of 2-hydroxy-1,2-diphenylpropan-1-one 4

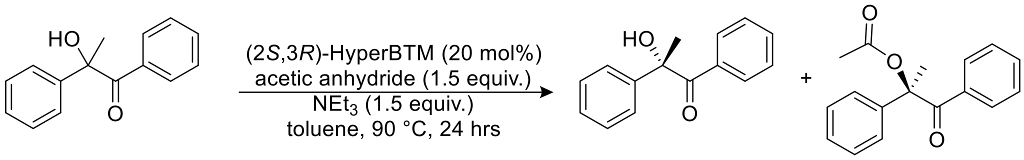

Following general procedure H, **2-hydroxy-1,2-diphenylpropan-1-one 4** (55 mg, 0.24 mmol), acetic anhydride (34 µL, 0.36 mmol), (2*S*,3*R*)-HyperBTM (15 mg, 0.048 mmol, 20 mol%) and NEt_3 (63 µL, 0.36 mmol) in toluene (1 mL) at 90 °C after 24 h gave, after column chromatography (eluent CH_2Cl_2/Et_2O, 9:1), alcohol (24 mg, 0.11 mmol, 44%) and ester (26 mg, 0.1 mmol, 41%). **c = 36%, *s* = 3**

(*S*)-2-Hydroxy-1,2-diphenylpropan-1-one (*S*)-4: $[\alpha]_D^{20}$ +26 (*c* 0.1, $CHCl_3$); Chiral HPLC Chiralpak AD-H (5% *i*PrOH:hexane, flow rate 1.0 mL min^{-1}, 211 nm, 30 °C) T_R: 12.2, 13.5 min, 38.4:61.6 er.

Racemic alcohol

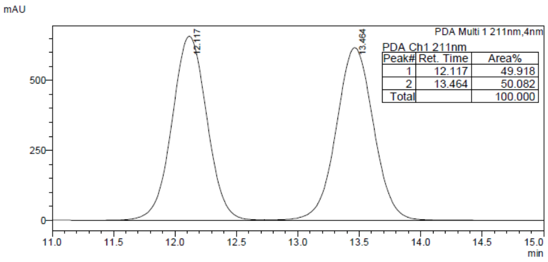

Enantioenriched alcohol

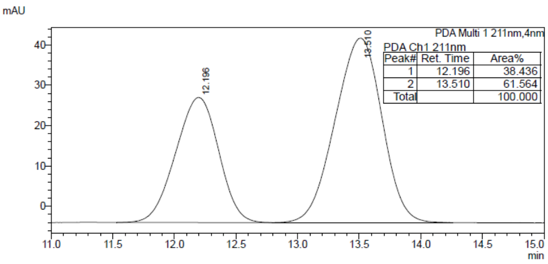

(*R*)-1-Oxo-1,2-diphenylpropan-2-yl isobutyrate (*R*)-S24: $[\alpha]_D^{20}$ +166 (*c* 0.1, $CHCl_3$); Chiral HPLC Chiralpak AD-H (5% *i*PrOH:hexane, flow rate 1.0 mL min^{-1}, 211 nm, 30 °C) T_R: 6.1, 8.0 min, 71:29 er.

Racemic ester

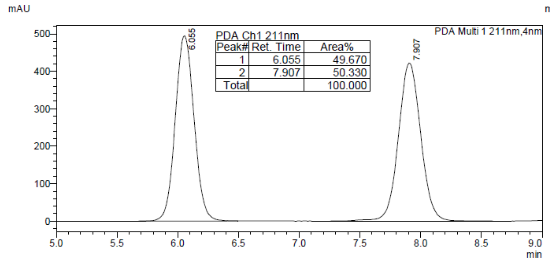

Enantioenriched ester

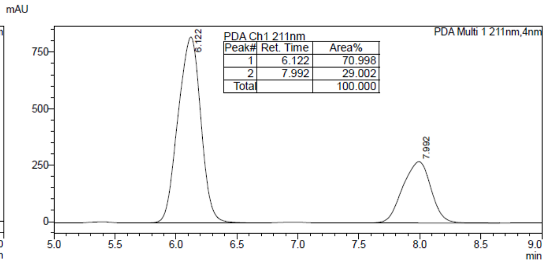

Table 1: KR of methyl 2-hydroxy-2-phenypropanoate 5

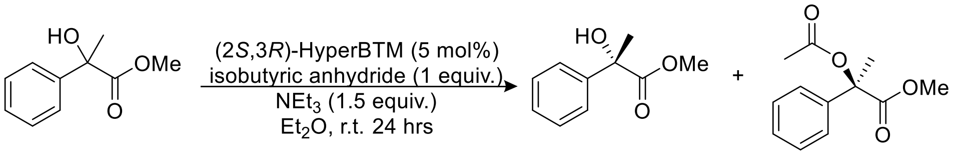

Following general procedure H, Following general procedure H, **methyl 2-hydroxy-2-phenypropanoate 5** (29 mg, 0.16 mmol), acetic anhydride (15 µL, 0.16 mmol), (2*S*,3*R*)-HyperBTM (2.5 mg, 0.008 mmol, 50 mol%) and NEt_3 (34 µL, 0.24 mmol) in Et_2O (1 mL) for 24 h gave, after column chromatography (eluent CH_2Cl_2/EtOAc, 9:1), alcohol (14 mg, 0.16 mmol, 49%) and ester (26 mg, 0.12 mmol, 36%). **c = 43%, *s* = 15**

(*S*)-Methyl 2-hydroxy-2-phenypropanoate (*S*)-5: Chiral HPLC Chiralpak AD-H (2% *i*PrOH:hexane, flow rate 1.0 mL min^{-1}, 211 nm, 30 °C) T_R: 11.4, 12.9 min, 79.4:20.6 er.

Racemic alcohol

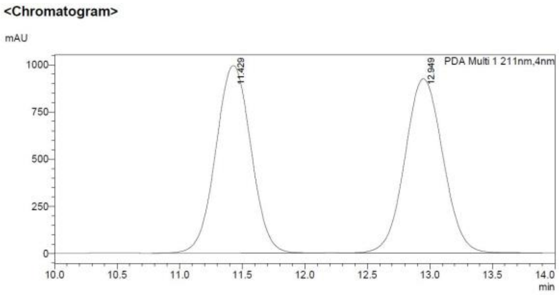

<Peak Table>

PDA Ch1 211nm

Peak#	Ret. Time	Area%
1	11.429	49.910
2	12.949	50.090
Total		100.000

Enantioenriched alcohol

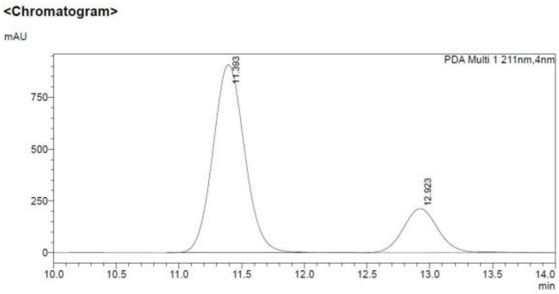

<Peak Table>

PDA Ch1 211nm

Peak#	Ret. Time	Area%
1	11.393	79.401
2	12.923	20.599
Total		100.000

(*R*)-Methyl 2-acetoxy-2-phenylpropanoate (*R*)-S25: $[\alpha]_D^{20}$ +172 (*c* 0.1, $CHCl_3$); Chiral HPLC Chiralpak IC (1% *i*PrOH:hexane, flow rate 1.0 mL min^{-1}, 211 nm, 30 °C) T_R: 17.7, 21.1 min, 10.9:89.1 er.

Racemic ester

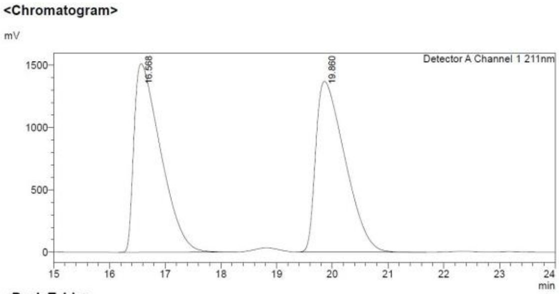

<Peak Table>

Detector A Channel 1 211nm

Peak#	Ret. Time	Area%
1	16.568	50.156
2	19.860	49.844
Total		100.000

Enantioenriched ester

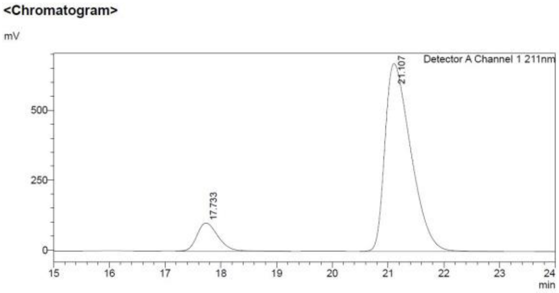

<Peak Table>

Detector A Channel 1 211nm

Peak#	Ret. Time	Area%
1	17.733	10.904
2	21.107	89.096
Total		100.000

Table 1: KR of methyl 2-hydroxy-2-phenypropanoate 5

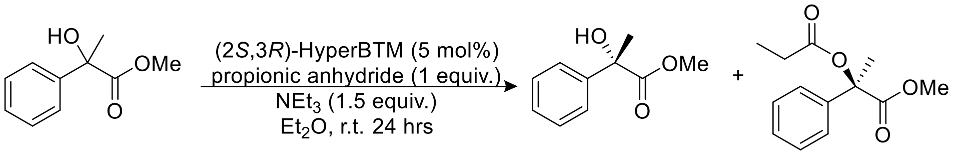

Following general procedure H, **methyl 2-hydroxy-2-phenypropanoate 5** (29 mg, 0.16 mmol), propionic anhydride (20 µL, 0.16 mmol), (2*S*,3*R*)-HyperBTM (2.5 mg, 0.008 mmol, 50 mol%) and NEt_3 (34 µL, 0.24 mmol) in Et_2O (1 mL) for 24 h gave, after column chromatography (eluent CH_2Cl_2/EtOAc, 9:1), alcohol (18 mg, 0.20 mmol, 61%) and ester (20 mg, 0.09 mmol, 27%).

(*S*)-Methyl 2-hydroxy-2-phenypropanoate (*S*)-5: $[\alpha]_D^{20}$ +48 (*c* 0.1, $CHCl_3$); Chiral HPLC Chiralpak AD-H (2% *i*PrOH:hexane, flow rate 1.0 mL min^{-1}, 211 nm, 30 °C) T_R: 11.5, 13.1 min, 86.3:13.6 er.

Racemic alcohol

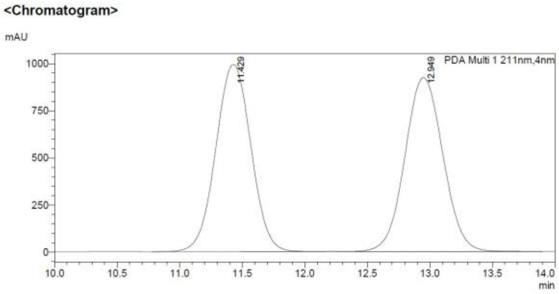

<Peak Table>

PDA Ch1 211nm

Peak#	Ret. Time	Area%
1	11.429	49.910
2	12.949	50.090
Total		100.000

Enantioenriched alcohol

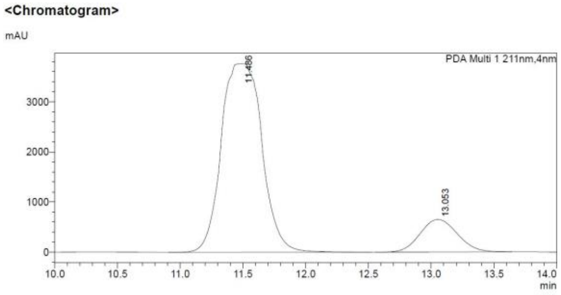

<Peak Table>

PDA Ch1 211nm

Peak#	Ret. Time	Area%
1	11.486	86.354
2	13.053	13.646
Total		100.000

(*R*)-Methyl 2-phenyl-2-(propionyloxy)propanoate (*R*)-S26: $[\alpha]_D^{20}$ +159 (*c* 0.1, $CHCl_3$); Chiral HPLC Chiralcel OJ-H (0.5% *i*PrOH:hexane, flow rate 0.5 mL min^{-1}, 211 nm, 30 °C) T_R: 26.9, 48.2 min, 8.4:91.6 er;

Racemic ester

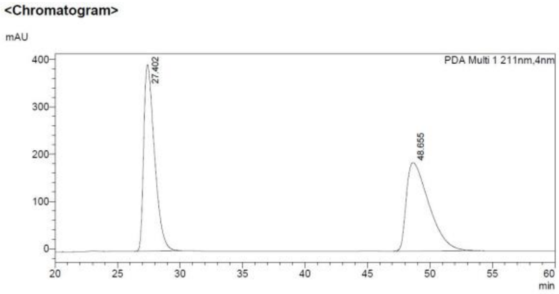

<Peak Table>

PDA Ch1 211nm

Peak#	Ret. Time	Area%
1	27.402	50.068
2	48.655	49.932
Total		100.000

Enantioenriched ester

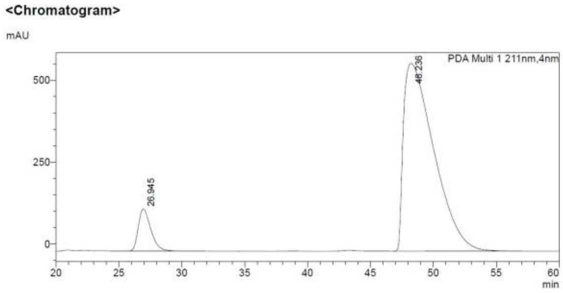

<Peak Table>

PDA Ch1 211nm

Peak#	Ret. Time	Area%
1	26.945	8.414
2	48.236	91.586
Total		100.000

Table 1: KR of methyl 2-hydroxy-2-phenypropanoate 5

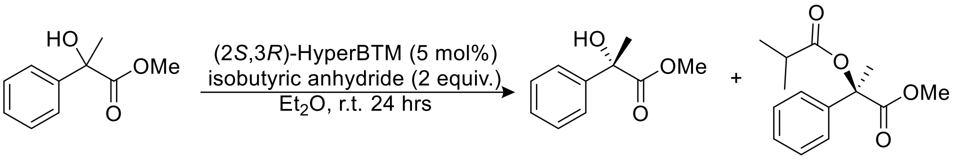

Following general procedure H, **methyl 2-hydroxy-2-phenypropanoate 5** (58 mg, 0.32 mmol), isobutyric anhydride (106 µL, 0.64 mmol) and (2*S*,3*R*)-HyperBTM (5 mg, 0.016 mmol, 5 mol%) in Et_2O (1 mL) for 24 h gave, after column chromatography (eluent CH_2Cl_2/EtOAc, 9:1), alcohol (25 mg, 0.14 mmol, 43%) and ester (31 mg, 0.12 mmol, 39%). **c = 41%, *s* = 70**

(*S*)-Methyl 2-hydroxy-2-phenypropanoate (*S*)-5: $[\alpha]_D^{20}$ +52 (*c* 0.1, $CHCl_3$) {Lit (*R*)-isomer:[25] $[\alpha]_D^{20}$ −47.8 (c 0.78 in $CHCl_3$); (*S*)-isomer:[26] $[\alpha]_D^{25}$ +38 (c 0.5 in $CHCl_3$)}; Chiral HPLC Chiralpak AD-H (2% *i*PrOH:hexane, flow rate 1.0 mL min^{-1}, 211 nm, 30 °C) T_R: 11.4, 12.9 min, 82.9:17.1 er.

Racemic alcohol

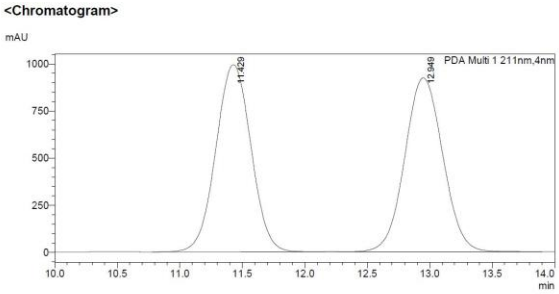

<Peak Table>

PDA Ch1 211nm

Peak#	Ret. Time	Area%
1	11.429	49.910
2	12.949	50.090
Total		100.000

Enantioenriched alcohol

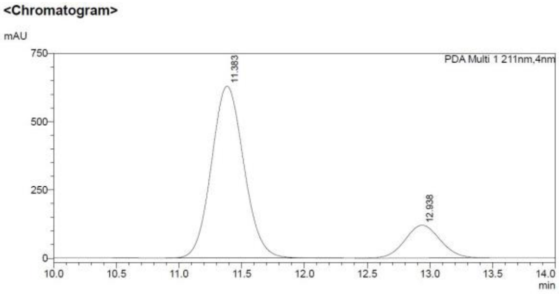

<Peak Table>

PDA Ch1 211nm

Peak#	Ret. Time	Area%
1	11.383	82.862
2	12.938	17.138
Total		100.000

(*R*)-Methyl 2-(isobutyryloxy)-2-phenylpropanoate (*R*)-S27: $[\alpha]_D^{20}$ +164 (*c* 0.1, $CHCl_3$); Chiral HPLC Chiralcel OJ-H (0.5% *i*PrOH:hexane, flow rate 0.5 mL min^{-1}, 211 nm, 30 °C) T_R: 15.9, 20.1 min, 2.9:97.1 er.

Racemic ester

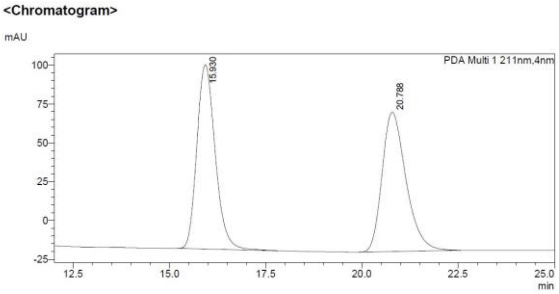

<Peak Table>

PDA Ch1 211nm

Peak#	Ret. Time	Area%
1	15.930	50.417
2	20.788	49.583
Total		100.000

Enantioenriched ester

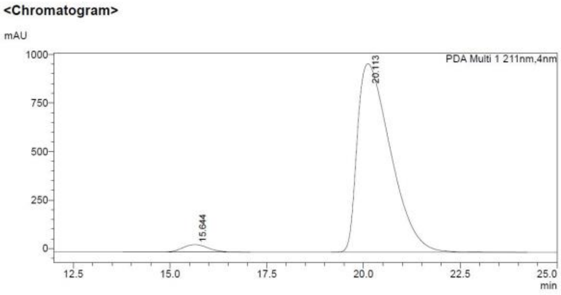

<Peak Table>

PDA Ch1 211nm

Peak#	Ret. Time	Area%
1	15.644	2.941
2	20.113	97.059
Total		100.000

Recovered alcohol was hydrolysed after reflux in aq. NaOH solution for 4 h gave $[\alpha]_D^{20}$ +63 (*c* 0.1, $CHCl_3$); **Lit data for acid:** $[\alpha]_D^{20}$ +35 (*c* 1.3, $CHCl_3$);[27] $[\alpha]_D^{20}$ +71 (*c* 0.14, $CHCl_3$).[28]
Absolute configuration determined to be (*S*)-isomer.

Table 1: KR of ethyl 2-hydroxy-2-phenylpropanoate 6

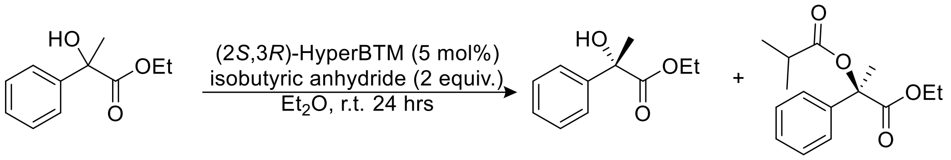

Following general procedure H, **ethyl 2-hydroxy-2-phenylpropanoate 6** (62 mg, 0.32 mmol), isobutyric anhydride (106 µL, 0.64 mmol) and (2*S*,3*R*)-HyperBTM (5 mg, 0.018 mmol, 5 mol%) in Et_2O (1 mL) for 24 h gave, after column chromatography (eluent CH_2Cl_2/EtOAc, 9:1), alcohol (37 mg, 0.19 mmol, 60%) and ester (21 mg, 0.08 mmol, 25%). **c = 32%, *s* = 60**

(*S*)-Ethyl 2-hydroxy-2-phenylpropanoate (*S*)-6: $[\alpha]_D^{20}$ +100 (*c* 0.1, $CHCl_3$) {Lit:[26] $[\alpha]_D^{25}$ +36 (c 0.5 in $CHCl_3$)}; Chiral HPLC Chiralpak AD-H (1% *i*PrOH:hexane, flow rate 1.0 mL min^{-1}, 211 nm, 30 °C) T_R: 13.6, 14.8 min, 72.5:27.5 er.

Racemic alcohol

<Chromatogram>

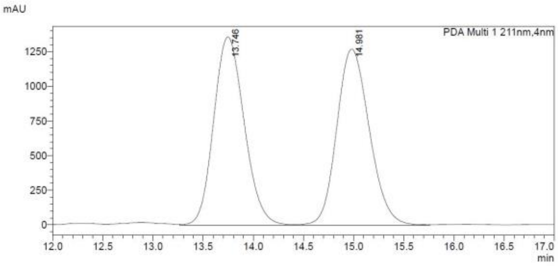

<Peak Table>

PDA Ch1 211nm

Peak#	Ret. Time	Area%
1	13.746	49.998
2	14.981	50.002
Total		100.000

Enantioenriched alcohol

<Chromatogram>

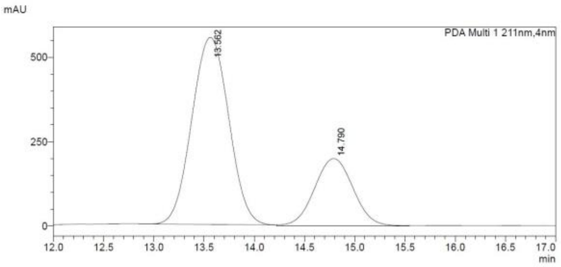

<Peak Table>

PDA Ch1 211nm

Peak#	Ret. Time	Area%
1	13.562	72.521
2	14.790	27.479
Total		100.000

(*R*)-Ethyl 2-(isobutyryloxy)-2-phenylpropanoate (*R*)-S28: $[\alpha]_D^{20}$ +34 (*c* 0.1, $CHCl_3$); Chiral HPLC Chiralcel OJ-H (1% *i*PrOH:hexane, flow rate 0.5 mL min^{-1}, 211 nm, 30 °C) T_R: 22.7, 29.0 min, 2.5:97.5 er.

Racemic ester

<Chromatogram>

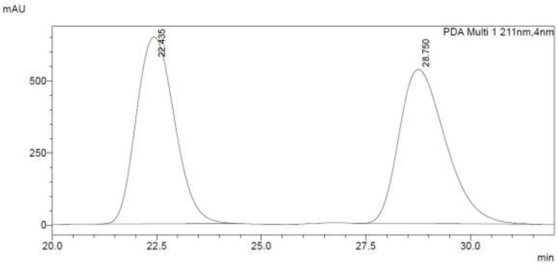

<Peak Table>

PDA Ch1 211nm

Peak#	Ret. Time	Area%
1	22.435	49.840
2	28.750	50.160
Total		100.000

Enantioenriched ester

<Chromatogram>

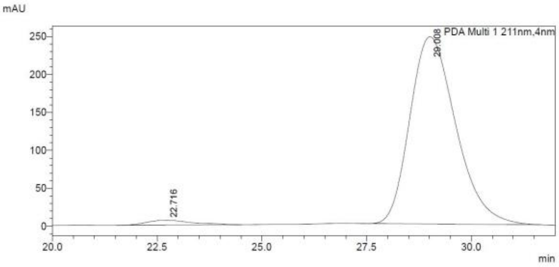

<Peak Table>

PDA Ch1 211nm

Peak#	Ret. Time	Area%
1	22.716	2.455
2	29.008	97.545
Total		100.000

Table 1: KR of *tert*-butyl 2-hydroxy-2-phenylpropanoate 7

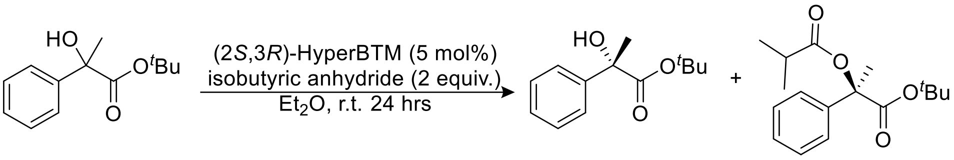

Following general procedure H, ***tert*-butyl 2-hydroxy-2-phenylpropanoate 7** (72 mg, 0.32 mmol), isobutyric anhydride (106 µL, 0.64 mmol) and (2*S*,3*R*)-HyperBTM (5 mg, 0.016 mmol, 5 mol%) in Et_2O (1 mL) for 24 h gave, after column chromatography (eluent CH_2Cl_2/EtOAc, 9:1), alcohol (56 mg, 0.25 mmol, 78%) and ester (10 mg, 0.04 mmol, 11%). **c(NMR) = 15%, *s* = 7**

(*S*)-*tert*-Butyl 2-hydroxy-2-phenylpropanoate (*S*)-7: $[\alpha]_D^{20}$ +6 (*c* 0.1, $CHCl_3$) {Lit:[26] $[\alpha]_D^{25}$ +53 (c 0.5 in $CHCl_3$)}; Chiral HPLC Chiralcel OJ-H (1% *i*PrOH:hexane, flow rate 0.5 mL min^{-1}, 211 nm, 30 °C) T_R: 10.9, 16.4 min, 56.5:43.5 er.

Racemic alcohol

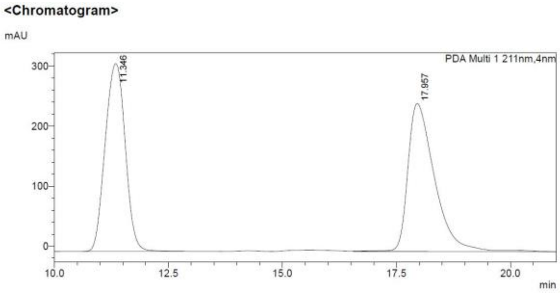

<Peak Table>

PDA Ch1 211nm

Peak#	Ret. Time	Area%
1	11.346	48.658
2	17.957	51.342
Total		100.000

Enantioenriched alcohol

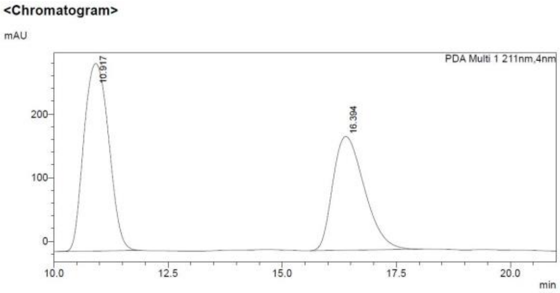

<Peak Table>

PDA Ch1 211nm

Peak#	Ret. Time	Area%
1	10.917	56.482
2	16.394	43.518
Total		100.000

(*R*)-*tert*-Butyl 2-(isobutyryloxy)-2-phenylpropanoate (*R*)-S29: $[\alpha]_D^{20}$ –5 (*c* 0.1, $CHCl_3$).
HPLC conditions could not be identified for separation of the ester, therefore conversion calculated by ^{1}H NMR analysis of crude reaction product mixture.

Table 1: KR of benzyl 2-hydroxy-2-phenypropanoate 8

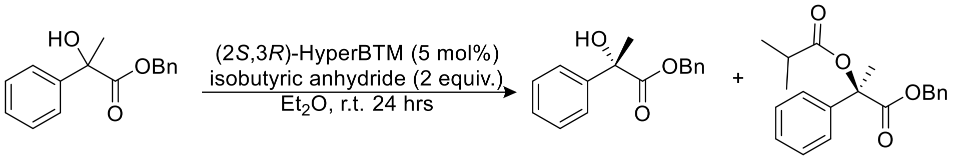

Following general procedure H, **benzyl 2-hydroxy-2-phenypropanoate 8** (82 mg, 0.32 mmol), isobutyric anhydride (106 µL, 0.64 mmol) and (2*S*,3*R*)-HyperBTM **37** (5 mg, 0.016 mmol, 5 mol%) in Et_2O (1 mL) for 24 h gave, after column chromatography (Isolera 4, Et_2O in petrol, 0% to 15% over 30 CV), alcohol (37 mg, 0.14 mmol, 45%) and ester (48 mg, 0.15 mmol, 46%). **c = 50%, *s* = 130**

(*S*)-Benzyl 2-hydroxy-2-phenypropanoate (*S*)-8: $[\alpha]_D^{20}$ +3 (*c* 1.0, $CHCl_3$); Chiral HPLC Chiralpak AD-H (1% *i*PrOH:hexane, flow rate 1.0 mL min^{-1}, 211 nm, 30 °C) T_R: 25.8, 34.3 min, 97.8:2.2 er.

Racemic alcohol

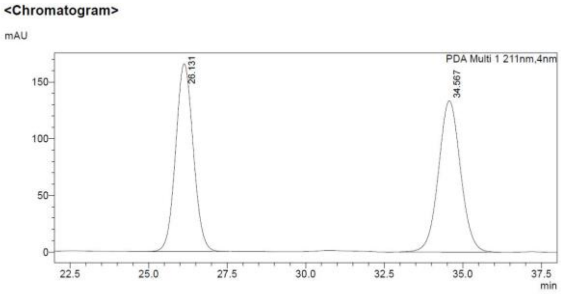

<Peak Table>

PDA Ch1 211nm

Peak#	Ret. Time	Area%
1	26.131	49.857
2	34.567	50.143
Total		100.000

Enantioenriched alcohol

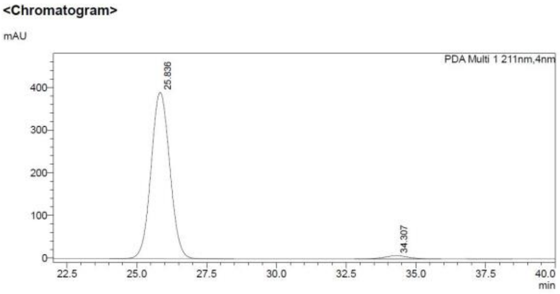

<Peak Table>

PDA Ch1 211nm

Peak#	Ret. Time	Area%
1	25.836	97.757
2	34.307	2.243
Total		100.000

(*R*)-Benzyl 2-(isobutyryloxy)-2-phenylpropanoate (*R*)-10: $[\alpha]_D^{20}$ +1 (*c* 1.5, $CHCl_3$); Chiral HPLC Chiralpak AD-H (1% *i*PrOH:hexane, flow rate 1.0 mL min^{-1}, 211 nm, 30 °C) T_R: 11.4, 17.4 min, 97.2:2.8 er;

Racemic ester

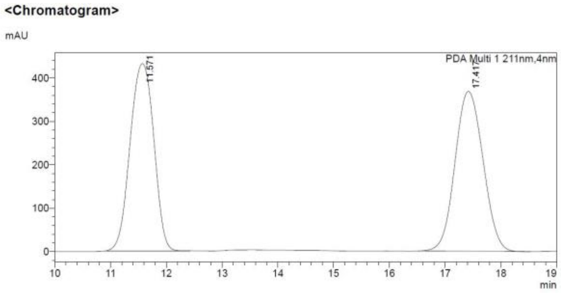

<Peak Table>

PDA Ch1 211nm

Peak#	Ret. Time	Area%
1	11.571	49.744
2	17.417	50.256
Total		100.000

Enantioenriched ester

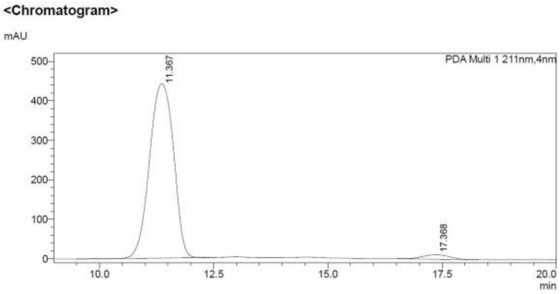

<Peak Table>

PDA Ch1 211nm

Peak#	Ret. Time	Area%
1	11.367	97.215
2	17.368	2.785
Total		100.000

Figure 3a: Kinetic resolution of benzyl 2-hydroxy-2-phenypropanoate 8

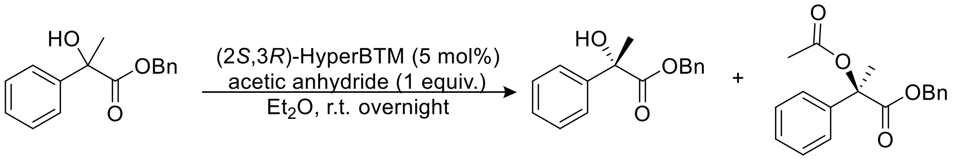

Following general procedure C, **benzyl 2-hydroxy-2-phenypropanoate 8** (51 mg, 0.2 mmol), acetic anhydride (19 µL, 0.2 mmol), triethylamine (56 mL, 0.4 mmol) and (*2S,3R*)-HyperBTM (3.1 mg, 0.01 mmol, 5 mol %) in Et_2O (0.6 mL) overnight gave, after column chromatography (Isolera 4, Et_2O in petrol, 0% to 10% over 35CV), alcohol (21 mg, 0.08 mmol, 30%) and ester (47 mg, 0.16 mmol, 58%). **c = 66%, *s* = 19**

Benzyl (*S*)-2-hydroxy-2-phenypropanoate (*S*)-8: $[\alpha]_D^{20}$ +6.4 (*c* 0.2, $CHCl_3$); Chiral HPLC Chiralpak AD-H (1% *i*PrOH:hexane, flow rate 1.0 mL min^{-1}, 211 nm, 30 °C) T_R: 25.5, 33.5 min, 99.9:0.1 er.

Racemic alcohol

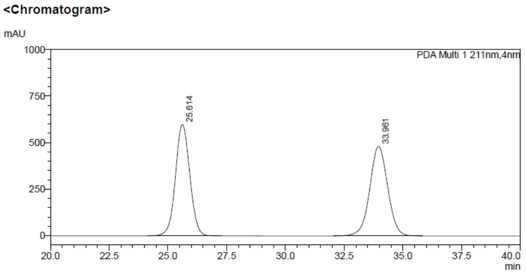

<Peak Table>

PDA Ch1 211nm

Peak#	Ret. Time	Area%
1	25.614	49.866
2	33.961	50.134
Total		100.000

Enantioenriched alcohol

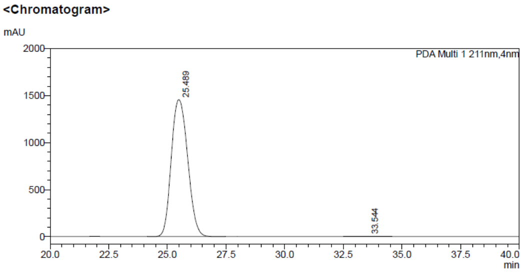

<Peak Table>

PDA Ch1 211nm

Peak#	Ret. Time	Area%
1	25.489	99.895
2	33.544	0.105
Total		100.000

Benzyl (*R*)-2-acetoxy-2-phenylpropanoate (*R*)-S30: $[\alpha]_D^{20}$ +4.8 (*c* 0.2, $CHCl_3$); Chiral HPLC Chiralpak AD-H (1% *i*PrOH:hexane, flow rate 1.0 mL min^{-1}, 211 nm, 30 °C) T_R: 14.5, 21.9 min, 76:24 er.

Racemic ester

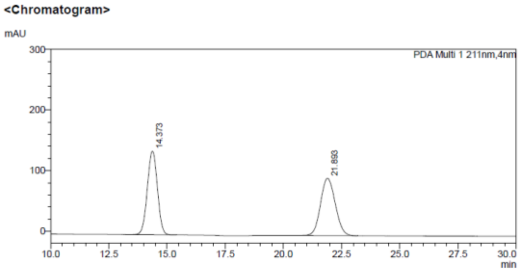

<Peak Table>

PDA Ch1 211nm

Peak#	Ret. Time	Area%
1	14.373	50.039
2	21.893	49.961
Total		100.000

Enantioenriched ester

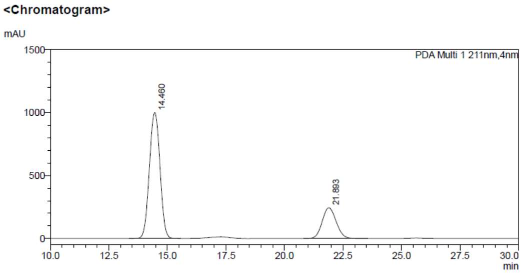

<Peak Table>

PDA Ch1 211nm

Peak#	Ret. Time	Area%
1	14.460	75.720
2	21.893	24.280
Total		100.000

Scheme 1: KR of benzyl 2-hydroxy-2-phenypropanoate 8

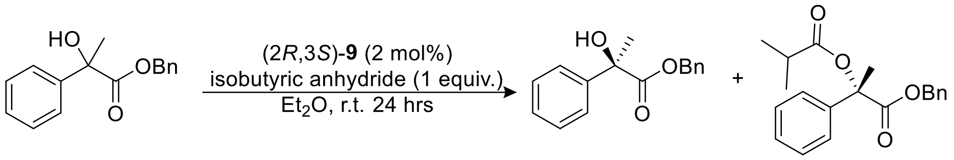

Following general procedure H, **benzyl 2-hydroxy-2-phenypropanoate 8** (51 mg, 0.2 mmol), isobutyric anhydride (33 µL, 0.2 mmol) and (2*R*,3*S*)-**9** (1.4 mg, 0.004 mmol, 2 mol%) in Et_2O (0.6 mL) gave, after column chromatography (Isolera 4, Et_2O in petrol, 0% to 10% over 30CV), alcohol (22 mg, 0.09 mmol, 43%) and ester (27 mg, 0.09 mmol, 42%). **c = 49%, *s* = 120**

(*R*)-Benzyl 2-hydroxy-2-phenylpropanoate (*R*)-8: $[\alpha]_D^{20}$ −2.1 (*c* 1.2, $CHCl_3$); Chiral HPLC Chiralpak AD-H (1% *i*PrOH:hexane, flow rate 1.0 mL min^{-1}, 211 nm, 30 °C) T_R: 25.8, 34.1 min, 3.9:96.1 er.

Racemic alcohol

<Chromatogram>

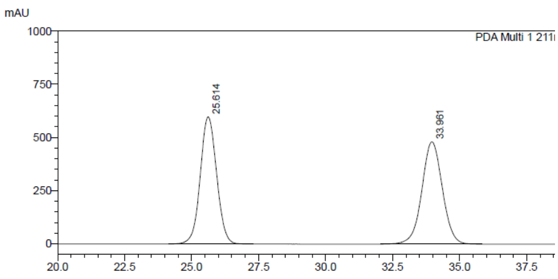

<Peak Table>

PDA Ch1 211nm

Peak#	Ret. Time	Area%
1	25.614	49.866
2	33.961	50.134
Total		100.000

Enantioenriched alcohol

<Chromatogram>

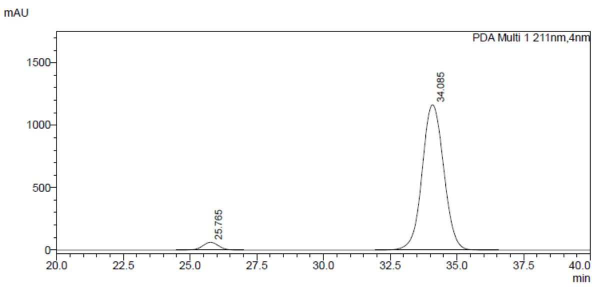

<Peak Table>

PDA Ch1 211nm

Peak#	Ret. Time	Area%
1	25.765	3.850
2	34.085	96.150
Total		100.000

(*S*)-Benzyl 2-(isobutyryloxy)-2-phenylpropanoate (*S*)-10: $[\alpha]_D^{20}$ −1.3 (*c* 1.5, $CHCl_3$); Chiral HPLC Chiralpak AD-H (1% *i*PrOH:hexane, flow rate 1.0 mL min^{-1}, 211 nm, 30 °C) T_R: 11.3, 16.9 min, 2.7:97.3 er.

Racemic ester

<Chromatogram>

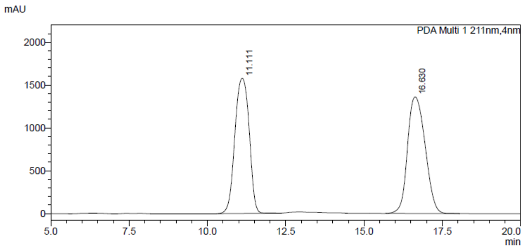

<Peak Table>

PDA Ch1 211nm

Peak#	Ret. Time	Area%
1	11.111	48.511
2	16.630	51.489
Total		100.000

Enantioenriched ester

<Chromatogram>

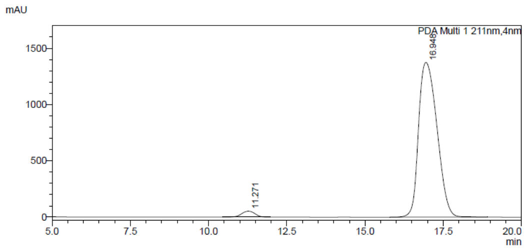

<Peak Table>

PDA Ch1 211nm

Peak#	Ret. Time	Area%
1	11.271	2.671
2	16.948	97.329
Total		100.000

Table 2: KR of benzyl 2-hydroxy-2-(naphthalene-2-yl)propanoate 11

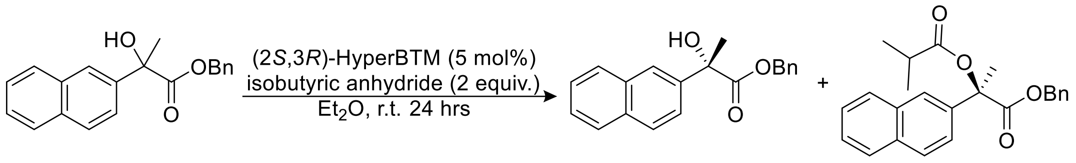

Following general procedure H, **benzyl 2-hydroxy-2-(naphthalene-2-yl)propanoate 11** (98 mg, 0.32 mmol), isobutyric anhydride (106 µL, 0.64 mmol) and (2*S*,3*R*)-HyperBTM (5 mg, 0.016 mmol, 5 mol%) in Et_2O (1 mL) for 24 h gave, after column chromatography (eluent CH_2Cl_2/Et_2O, 9:1), alcohol (40 mg, 0.13 mmol, 41%) and ester (49 mg, 0.13 mmol, 41%).
c = 52%, *s* = 70

(*S*)-Benzyl 2-hydroxy-2-(naphthalene-2-yl)propanoate (*S*)-11: $[\alpha]_D^{20}$ +17 (*c* 0.1, $CHCl_3$); Chiral HPLC Chiralpak AD-H (1% *i*PrOH:hexane, flow rate 1.0 mL min^{-1}, 211 nm, 30 °C) T_R: 19.9, 27.5 min, 98.2:1.8 er.

Racemic alcohol

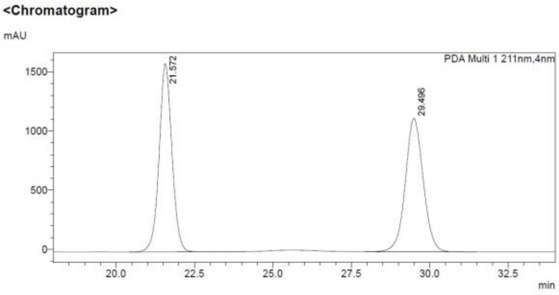

<Peak Table>

PDA Ch1 211nm

Peak#	Ret. Time	Area%
1	21.572	50.222
2	29.496	49.778
Total		100.000

Enantioenriched alcohol

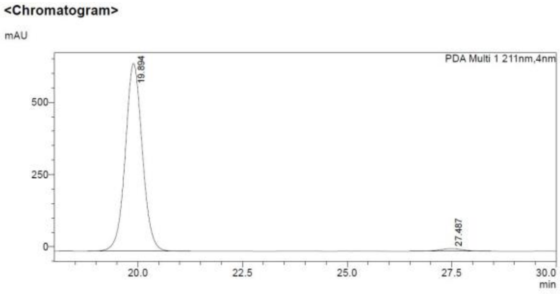

<Peak Table>

PDA Ch1 211nm

Peak#	Ret. Time	Area%
1	19.894	98.201
2	27.487	1.799
Total		100.000

(*R*)-Benzyl 2-(isobutyryloxy)-2-(naphthalen-2-yl)propanoate (*R*)-S31: $[\alpha]_D^{20}$ +89 (*c* 0.1, $CHCl_3$); Chiral HPLC Chiralpak AD-H (1% *i*PrOH:hexane, flow rate 1.0 mL min^{-1}, 211 nm, 30 °C) T_R: 17.9, 53.1 min, 94.8:5.2 er.

Racemic ester

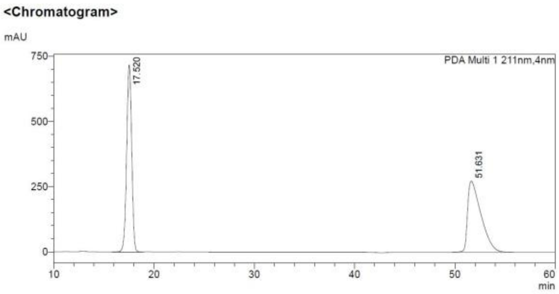

<Peak Table>

PDA Ch1 211nm

Peak#	Ret. Time	Area%
1	17.520	50.005
2	51.631	49.995
Total		100.000

Enantioenriched ester

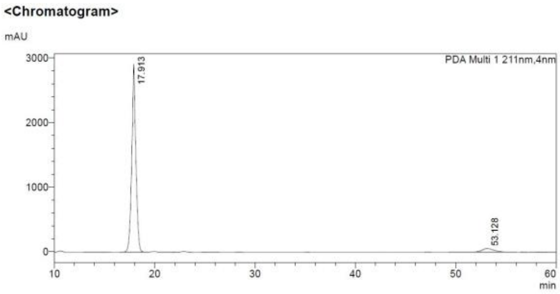

<Peak Table>

PDA Ch1 211nm

Peak#	Ret. Time	Area%
1	17.913	94.838
2	53.128	5.162
Total		100.000

Table 2: KR of benzyl 2-hydroxy-2-(*p*-tolyl)propanoate 12

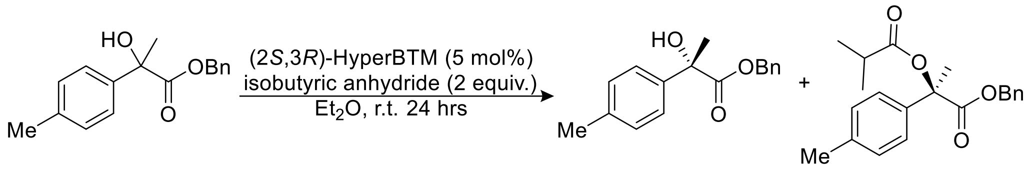

Following general procedure H, **benzyl 2-hydroxy-2-(*p*-tolyl)propanoate 12** (86 mg, 0.32 mmol), isobutyric anhydride (106 µL, 0.64 mmol) and (2*S*,3*R*)-HyperBTM (5 mg, 0.016 mmol, 5 mol%) in Et_2O (1 mL) for 24 h gave, after column chromatography (eluent Petrol/Et_2O, 9:1), alcohol (18.0 mg, 0.07 mmol, 45%) and ester (24.4 mg, 0.07 mmol, 45%). **c = 50%, *s* = 70**

(*S*)-Benzyl 2-hydroxy-2-(*p*-tolyl)propanoate (*S*)-12: $[\alpha]_D^{20}$ +5 (*c* 0.1, $CHCl_3$); Chiral HPLC Chiralpak AD-H (5% *i*PrOH:hexane, flow rate 1.0 mL min^{-1}, 211 nm, 30 °C) T_R: 11.7, 13.9 min, 95.1:4.9 er.

Racemic alcohol

<Chromatogram>

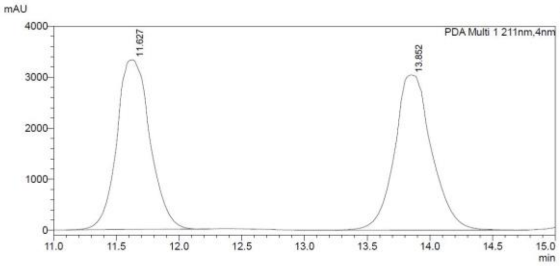

<Peak Table>

PDA Ch1 211nm

Peak#	Ret. Time	Area%
1	11.627	49.535
2	13.852	50.465
Total		100.000

Enantioenriched alcohol

<Chromatogram>

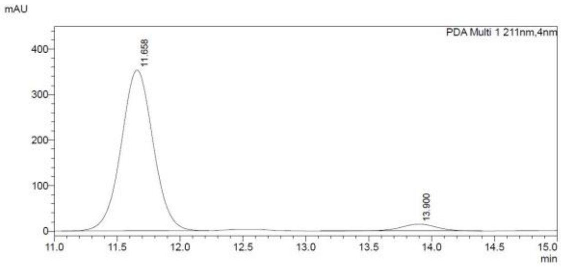

<Peak Table>

PDA Ch1 211nm

Peak#	Ret. Time	Area%
1	11.658	95.148
2	13.900	4.852
Total		100.000

(*R*)-Benzyl 2-(isobutyryloxy)-2-(*p*-tolyl)propanoate (*R*)-S32: $[\alpha]_D^{20}$ +49 (*c* 0.1, $CHCl_3$); Chiral HPLC Chiralpak AD-H (2% *i*PrOH:hexane, flow rate 1.0 mL min^{-1}, 211 nm, 30 °C) T_R: 8.2, 11.8 min, 95.8:4.2 er.

Racemic ester

<Chromatogram>

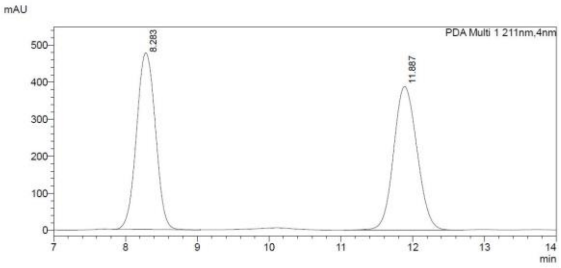

<Peak Table>

PDA Ch1 211nm

Peak#	Ret. Time	Area%
1	8.283	49.893
2	11.887	50.107
Total		100.000

Enantioenriched ester

<Chromatogram>

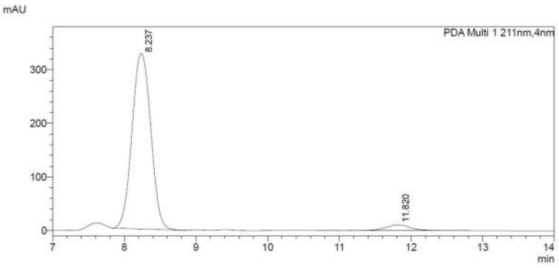

<Peak Table>

PDA Ch1 211nm

Peak#	Ret. Time	Area%
1	8.237	95.791
2	11.820	4.209
Total		100.000

Table 2: KR of benzyl 2-(4-(*tert*-butyl)phenyl)-2-hydroxypropanoate 13

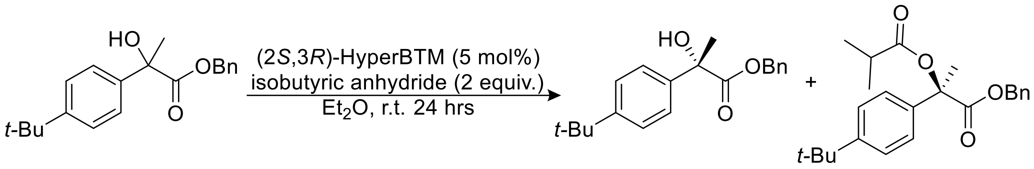

Following general procedure H, **benzyl 2-(4-(*tert*-butyl)phenyl)-2-hydroxypropanoate 13** (112 mg, 0.32 mmol), isobutyric anhydride (106 µL, 0.64 mmol) and (2*S*,3*R*)-HyperBTM (5 mg, 0.016 mmol, 5 mol%) in Et_2O (1 mL) for 24 h gave, after column chromatography (Isolera 4, Et_2O in petrol, 0% to 15% over 30 CV), alcohol (45 mg, 0.12 mmol, 37%) and ester (39 mg, 0.12 mmol, 39%). **c = 49%, *s* = 140**

(*S*)-Benzyl 2-(4-(*tert*-butyl)phenyl)-2-hydroxypropanoate (*S*)-13: $[\alpha]_D^{20}$ +2 (*c* 0.25, $CHCl_3$); Chiral HPLC Chiralcel OJ-H (1% *i*PrOH:hexane, flow rate 1.0 mL min^{-1}, 211 nm, 30 °C) T_R: 24.3, 41.4 min, 96:4 er.

Racemic alcohol

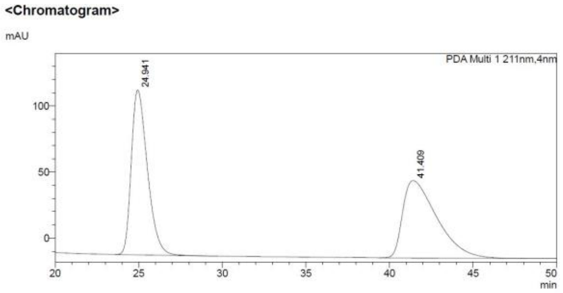

<Peak Table>

PDA Ch1 211nm

Peak#	Ret. Time	Area%
1	24.941	50.099
2	41.409	49.901
Total		100.000

Enantioenriched alcohol

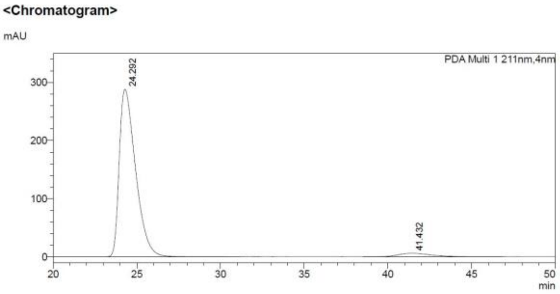

<Peak Table>

PDA Ch1 211nm

Peak#	Ret. Time	Area%
1	24.292	96.013
2	41.432	3.987
Total		100.000

(*R*)-Benzyl 2-(4-(*tert*-butyl)phenyl)-2-(isobutyryloxy)propanoate (*R*)-S33: $[\alpha]_D^{20}$ −3 (*c* 1.4, $CHCl_3$); Chiral HPLC Chiralpak AD-H (1% *i*PrOH:hexane, flow rate 0.5 mL min^{-1}, 211 nm, 30 °C) T_R: 14.3, 16.9 min, 97.7:2.3 er.

Racemic ester

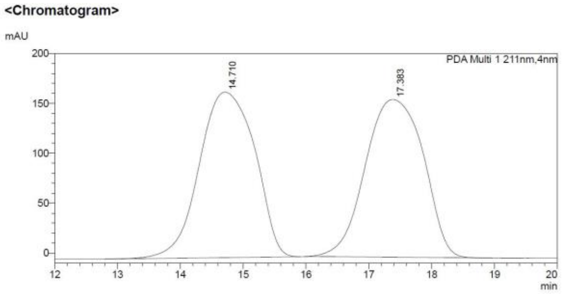

<Peak Table>

PDA Ch1 211nm

Peak#	Ret. Time	Area%
1	14.710	50.085
2	17.383	49.915
Total		100.000

Enantioenriched ester

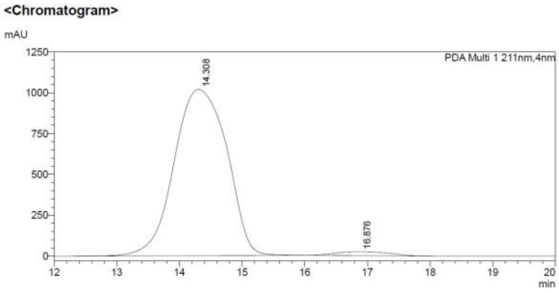

<Peak Table>

PDA Ch1 211nm

Peak#	Ret. Time	Area%
1	14.308	97.693
2	16.876	2.307
Total		100.000

Table 2: KR of benzyl 2-hydroxy-2-(4-methoxyphenyl)propanoate 14

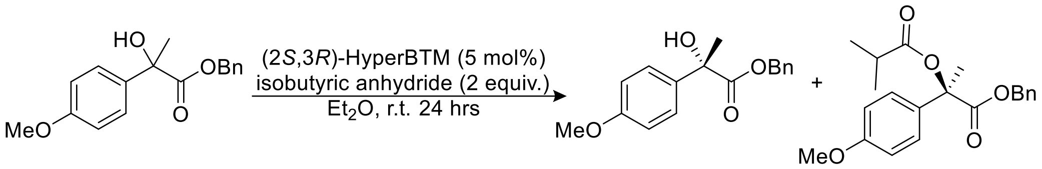

Following general procedure H, **benzyl 2-hydroxy-2-(4-methoxyphenyl)propanoate 14** (92 mg, 0.32 mmol), isobutyric anhydride (106 µL, 0.64 mmol) and (2*S*,3*R*)-HyperBTM (5 mg, 0.016 mmol, 5 mol%) in Et_2O (1 mL) for 24 h gave, after column chromatography (eluent CH_2Cl_2/Et_2O, 9:1), alcohol (45 mg, 0.16 mmol, 49%) and ester (39 mg, 0.11 mmol, 35%).
c = 43%, *s* = 80

(*S*)-Benzyl 2-hydroxy-2-(4-methoxyphenyl)propanoate (*S*)-14: $[\alpha]_D^{20}$ +22 (*c* 0.1, $CHCl_3$); Chiral HPLC Chiralpak AD-H (5% *i*PrOH:hexane, flow rate 1.0 mL min^{-1}, 211 nm, 30 °C) T_R: 18.3, 20.5 min, 85.6:14.4 er.

Racemic alcohol

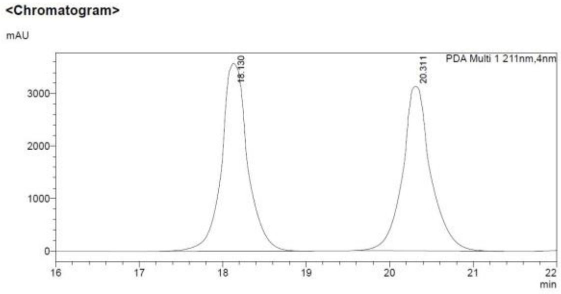

<Peak Table>

PDA Ch1 211nm

Peak#	Ret. Time	Area%
1	18.130	51.355
2	20.311	48.645
Total		100.000

Enantioenriched alcohol

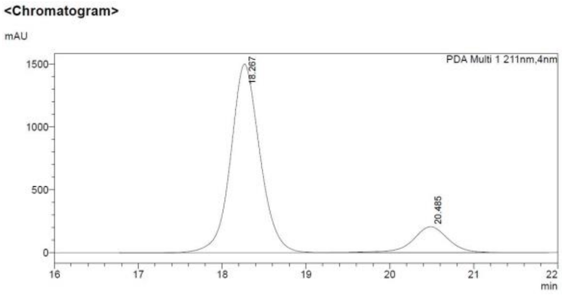

<Peak Table>

PDA Ch1 211nm

Peak#	Ret. Time	Area%
1	18.267	85.590
2	20.485	14.410
Total		100.000

(*R*)-Benzyl 2-(isobutyryloxy)-2-(4-methoxyphenyl)propanoate (*R*)-S34: $[\alpha]_D^{20}$ +64 (*c* 0.1, $CHCl_3$); Chiral HPLC Chiralpak AD-H (1% *i*PrOH:hexane, flow rate 1.0 mL min^{-1}, 211 nm, 30 °C) T_R: 19.5, 31.2 min, 97.5:2.5 er.

Racemic ester

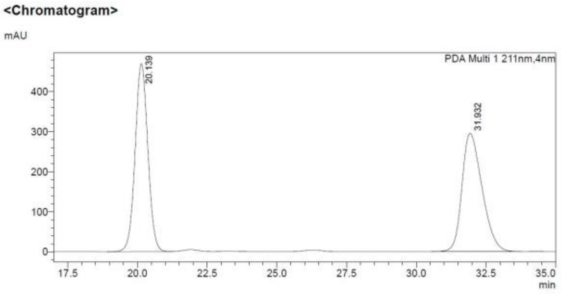

<Peak Table>

PDA Ch1 211nm

Peak#	Ret. Time	Area%
1	20.139	50.183
2	31.932	49.817
Total		100.000

Enantioenriched ester

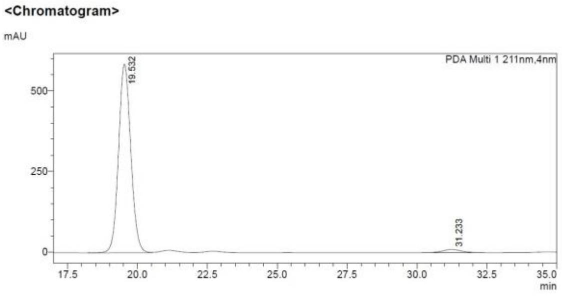

<Peak Table>

PDA Ch1 211nm

Peak#	Ret. Time	Area%
1	19.532	97.542
2	31.233	2.458
Total		100.000

Table 2: KR of benzyl 2-hydroxy-2-(3-methoxyphenyl)propanoate 15

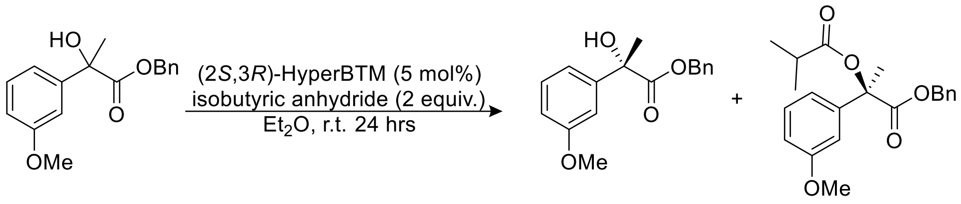

Following general procedure H, **benzyl 2-hydroxy-2-(3-methoxyphenyl)propanoate 15** (92 mg, 0.32 mmol), isobutyric anhydride (106 µL, 0.64 mmol) and (2*S*,3*R*)-HyperBTM (5 mg, 0.016 mmol, 5 mol%) in Et_2O (1 mL) for 24 h gave, after column chromatography (Isolera 4, Et_2O in petrol, 0% to 15% over 25 CV), alcohol (40 mg, 0.14 mmol, 44%) and ester (54 mg, 0.15 mmol, 47%). **c = 51%, *s* = 60**

(*S*)-Benzyl 2-(isobutyryloxy)-2-(3-methoxyphenyl)propanoate (*S*)-15: $[\alpha]_D^{20}$ −8 (*c* 1.1, $CHCl_3$); Chiral HPLC Chiralpak AD-H (1% *i*PrOH:hexane, flow rate 1 mL min^{-1}, 211 nm, 30 °C) T_R: 47.0, 63.0 min, 96.4:3.6 er;

Racemic alcohol

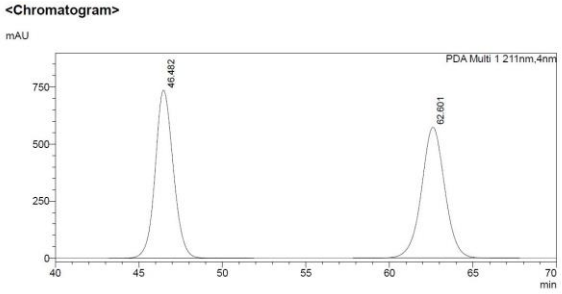

<Peak Table>

PDA Ch1 211nm

Peak#	Ret. Time	Area%
1	46.482	49.940
2	62.601	50.060
Total		100.000

Enantioenriched alcohol

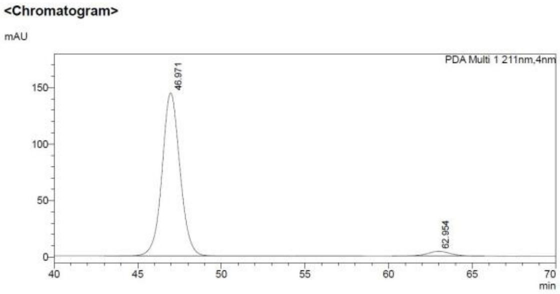

<Peak Table>

PDA Ch1 211nm

Peak#	Ret. Time	Area%
1	46.971	96.387
2	62.954	3.613
Total		100.000

(*R*)-Benzyl 2-(4-(*tert*-butyl)phenyl)-2-(isobutyryloxy)propanoate (*R*)-S35: $[\alpha]_D^{20}$ +4 (*c* 1.7, $CHCl_3$); Chiral HPLC Chiralpak AD-H (1% *i*PrOH:hexane, flow rate 1.0 mL min^{-1}, 211 nm, 30 °C) T_R: 16.5, 27.9 min, 94.5:5.5 er.

Racemic ester

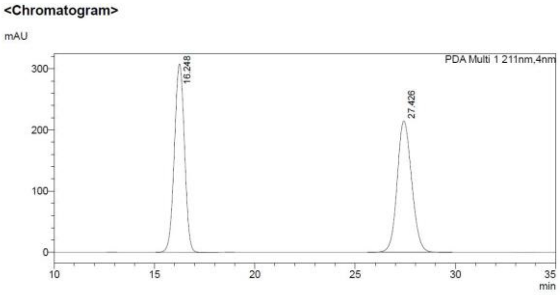

<Peak Table>

PDA Ch1 211nm

Peak#	Ret. Time	Area%
1	16.248	50.019
2	27.426	49.981
Total		100.000

Enantioenriched ester

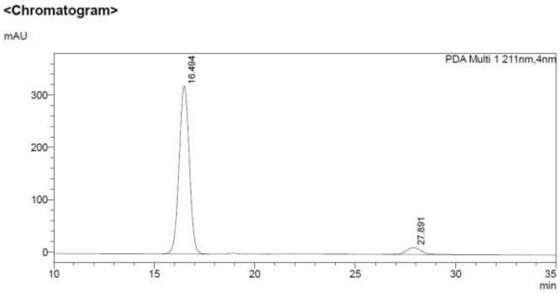

<Peak Table>

PDA Ch1 211nm

Peak#	Ret. Time	Area%
1	16.494	94.500
2	27.891	5.500
Total		100.000

Table 2: KR of benzyl 2-hydroxy-2-(2-methoxyphenyl)propanoate 16

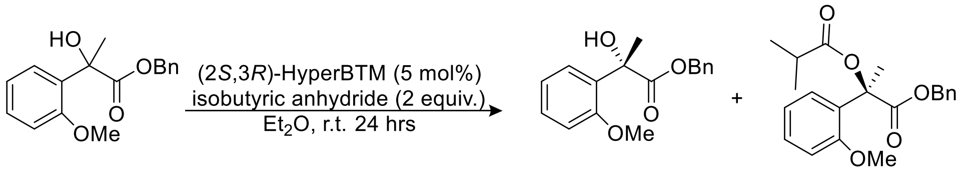

Following general procedure H, **benzyl 2-hydroxy-2-(2-methoxyphenyl)propanoate 16** (92 mg, 0.32 mmol), isobutyric anhydride (106 µL, 0.64 mmol) and (2*S*,3*R*)-HyperBTM (5 mg, 0.016 mmol, 5 mol%) reacted in Et_2O (1 mL) for 24 h. HPLC data obtained from reaction crude. **c = 6%, *s* = 33**

(*S*)-Benzyl 2-hydroxy-2-(2-methoxyphenyl)propanoate (*S*)-16: Chiral HPLC Chiralpak AD-H (1% *i*PrOH:hexane, flow rate 0.5 mL min^{-1}, 211 nm, 30 °C) T_R: 17.6, 21.0 min, 47.2:52.8 er;

Racemic alcohol

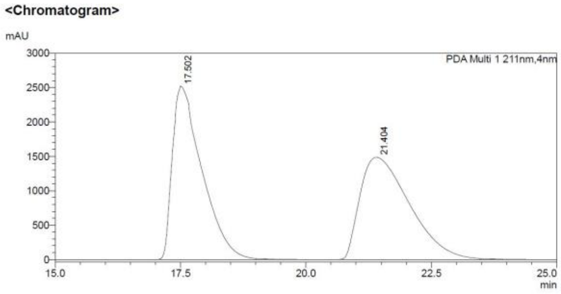

<Peak Table>

PDA Ch1 211nm

Peak#	Ret. Time	Area%
1	17.502	50.234
2	21.404	49.766
Total		100.000

Enantioenriched alcohol

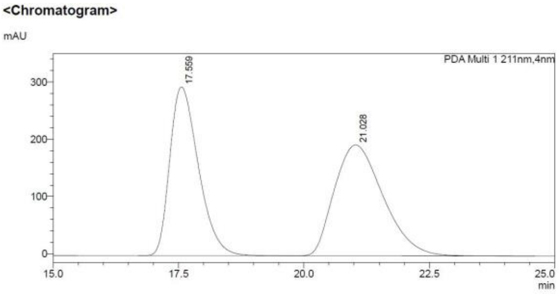

<Peak Table>

PDA Ch1 211nm

Peak#	Ret. Time	Area%
1	17.559	47.194
2	21.028	52.806
Total		100.000

(*R*)-Benzyl 2-(isobutyryloxy)-2-(2-methoxyphenyl)propanoate (*R*)-S36: Chiral HPLC Chiralpak AD-H (1% *i*PrOH:hexane, flow rate 1.0 mL min^{-1}, 211 nm, 30 °C) T_R: 15.7, 24.7 min, 96.9:3.1 er.

Racemic ester

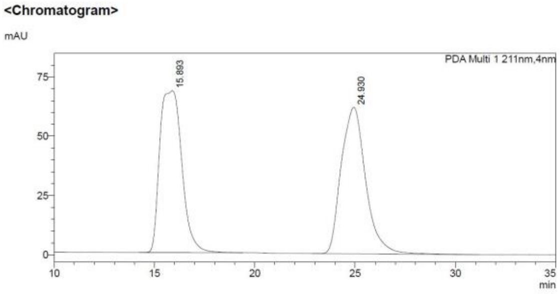

<Peak Table>

PDA Ch1 211nm

Peak#	Ret. Time	Area%
1	15.893	49.950
2	24.930	50.050
Total		100.000

Enantioenriched ester

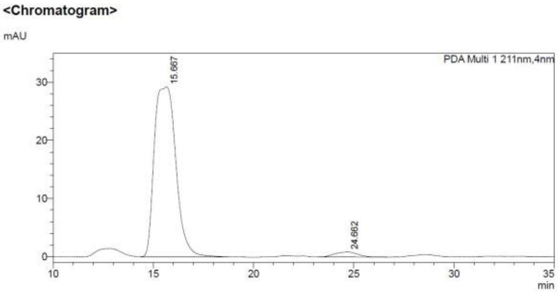

<Peak Table>

PDA Ch1 211nm

Peak#	Ret. Time	Area%
1	15.667	96.914
2	24.662	3.086
Total		100.000

Table 2: KR of benzyl 2-hydroxy-2-(2-methoxyphenyl)propanoate 16

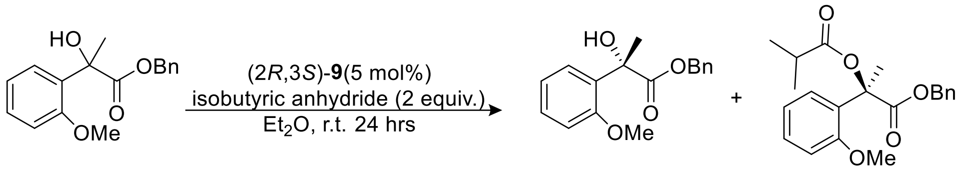

Following general procedure H, **benzyl 2-hydroxy-2-(2-methoxyphenyl)propanoate 16** (92 mg, 0.32 mmol), isobutyric anhydride (106 µL, 0.64 mmol) and (2*R*,3*S*)-**9** (5 mg, 0.016 mmol, 5 mol%) in Et_2O (1 mL) for 24 h gave, after column chromatography (Isolera 4, Et_2O in petrol, 0% to 20% over 25 CV then 20% for 10 CV), alcohol (53 mg, 0.19 mmol, 58%) and ester (34 mg, 0.10 mmol, 30%). **c = 35%, *s* = 20**

(*R*)-Benzyl 2-hydroxy-2-(2-methoxyphenyl)propanoate (*R*)-16: $[\alpha]_D^{20}$ −8 (*c* 1.1, $CHCl_3$); Chiral HPLC Chiralpak AD-H (1% *i*PrOH:hexane, flow rate 0.5 mL min^{-1}, 211 nm, 30 °C) T_R: 17.9, 22.5 min, 72.9:27.1 er.

Racemic alcohol

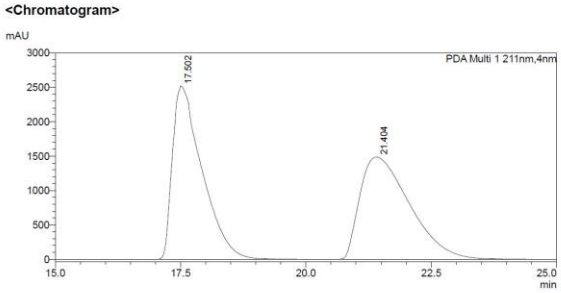

<Peak Table>

PDA Ch1 211nm

Peak#	Ret. Time	Area%
1	17.502	50.234
2	21.404	49.766
Total		100.000

Enantioenriched alcohol

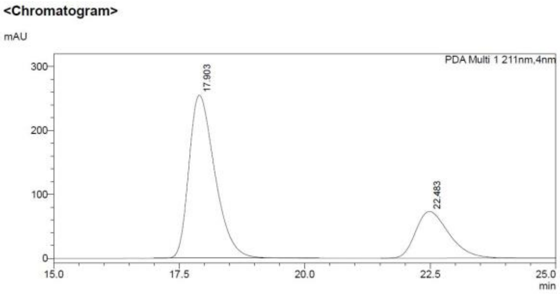

<Peak Table>

PDA Ch1 211nm

Peak#	Ret. Time	Area%
1	17.903	72.943
2	22.483	27.057
Total		100.000

(*S*)-Benzyl 2-(isobutyryloxy)-2-(2-methoxyphenyl)propanoate (*S*)-S36: $[\alpha]_D^{20}$ −27 (*c* 0.5, $CHCl_3$); Chiral HPLC Chiralcel OD-H (1% *i*PrOH:hexane, flow rate 1.0 mL min^{-1}, 211 nm, 30 °C) T_R: 16.1, 24.7 min, 7.2:92.8 er.

Racemic ester

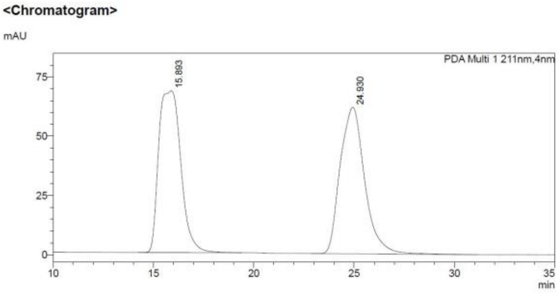

<Peak Table>

PDA Ch1 211nm

Peak#	Ret. Time	Area%
1	15.893	49.950
2	24.930	50.050
Total		100.000

Enantioenriched ester

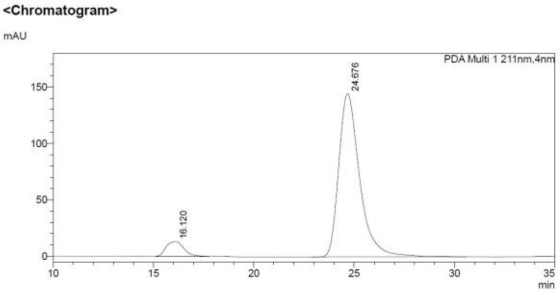

<Peak Table>

PDA Ch1 211nm

Peak#	Ret. Time	Area%
1	16.120	7.159
2	24.676	92.841
Total		100.000

Table 2: KR of benzyl 2-(3,5-bis(trifluoromethyl)phenyl)-2-hydroxypropanoate 17

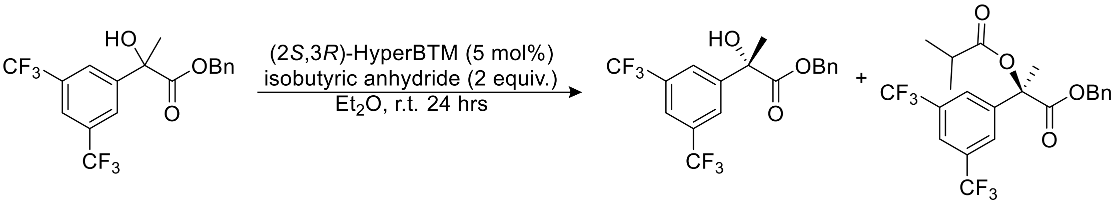

Following general procedure H, **benzyl 2-(3,5-bis(trifluoromethyl)phenyl)-2-hydroxypropanoate 17** (125 mg, 0.32 mmol), isobutyric anhydride (53 µL, 0.32 mmol) and (2*S*,3*R*)-HyperBTM (5 mg, 0.016 mmol, 5 mol%) in Et_2O (0.5 mL) for 24 h gave, after column chromatography (eluent CH_2Cl_2/EtOAc, 9:1), alcohol (36 mg, 0.09 mmol, 29%) and ester (78 mg, 0.17 mmol, 53%). **c = 63%, *s* = 12**

(*S*)-Benzyl 2-(3,5-bis(trifluoromethyl)phenyl)-2-hydroxypropanoate (*S*)-17: $[\alpha]_D^{20}$ +47 (*c* 0.1, $CHCl_3$); Chiral HPLC Chiralcel OD-H (1% *i*PrOH:hexane, flow rate 1.0 mL min^{-1}, 211 nm, 30 °C) T_R: 8.7, 11.1 min, 97.6:2.4 er.

Racemic alcohol

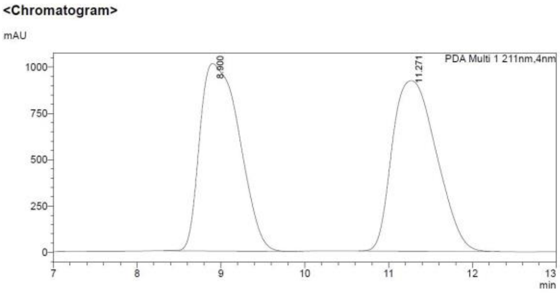

<Peak Table>

PDA Ch1 211nm

Peak#	Ret. Time	Area%
1	8.900	49.607
2	11.271	50.393
Total		100.000

Enantioenriched alcohol

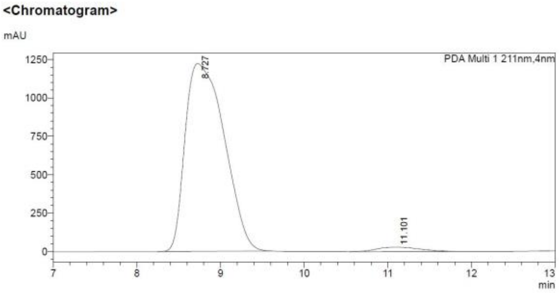

<Peak Table>

PDA Ch1 211nm

Peak#	Ret. Time	Area%
1	8.727	97.590
2	11.101	2.410
Total		100.000

(*R*)-Benzyl 2-(3,5-bis(trifluoromethyl)phenyl)-2-(isobutyryloxy)propanoate (*R*)-S37: $[\alpha]_D^{20}$ +28 (*c* 0.1, $CHCl_3$).
HPLC conditions could not be identified for separation of the ester, therefore conversion calculated by ^{1}H NMR analysis of crude reaction product mixture.

Table 2: KR of benzyl 2-hydroxy-2-(thiophene-2-yl)propanoate 18

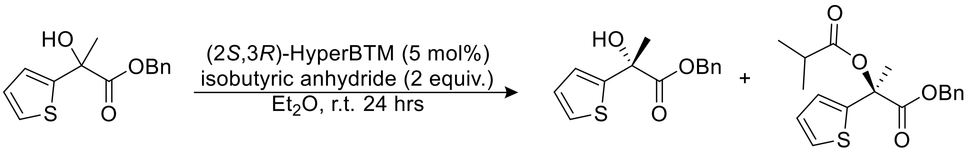

Following general procedure H, **benzyl 2-hydroxy-2-(thiophene-2-yl)propanoate 18** (84 mg, 0.32 mmol), isobutyric anhydride (106 µL, 0.64 mmol) and (2*S*,3*R*)-HyperBTM (5 mg, 0.016 mmol, 5 mol%) in Et_2O (1 mL) for 24 h gave, after column chromatography (eluent CH_2Cl_2/Et_2O, 9:1), alcohol (34 mg, 0.13 mmol, 40%) and ester (52 mg, 0.16 mmol, 49%).
c = 55%, *s* = 60

(*R*)-Benzyl 2-hydroxy-2-(thiophene-2-yl)propanoate (*R*)-18: $[\alpha]_D^{20}$ +99 (*c* 0.1, $CHCl_3$); Chiral HPLC Chiralpak AD-H (5% *i*PrOH:hexane, flow rate 1.0 mL min^{-1}, 211 nm, 30 °C) T_R: 25.8, 32.3 min, 99.9:0.1 er.

Racemic alcohol

<Chromatogram>

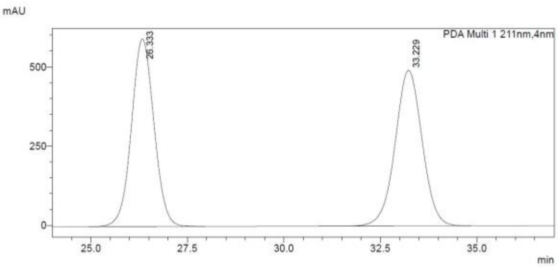

<Peak Table>

PDA Ch1 211nm

Peak#	Ret. Time	Area%
1	26.333	50.069
2	33.229	49.931
Total		100.000

Enantioenriched alcohol

<Chromatogram>

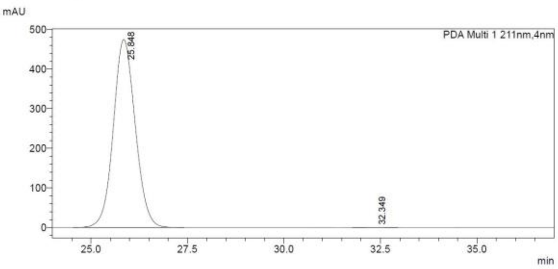

<Peak Table>

PDA Ch1 211nm

Peak#	Ret. Time	Area%
1	25.848	99.902
2	32.349	0.098
Total		100.000

(*S*)-Benzyl 2-(isobutyryloxy)-2-(thiophene-2-yl)propanoate (*S*)-S38: $[\alpha]_D^{20}$ +76 (*c* 0.1, $CHCl_3$); Chiral HPLC Chiralpak AD-H (1% *i*PrOH:hexane, flow rate 1.0 mL min^{-1}, 211 nm, 30 °C) T_R: 11.9, 15.8 min, 90.6:9.4 er.

Racemic ester

<Chromatogram>

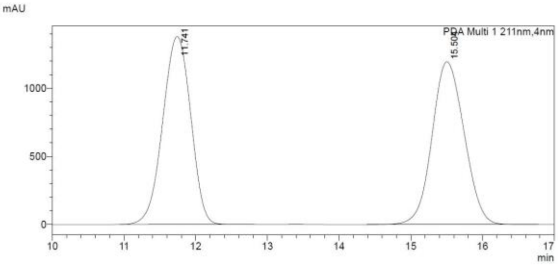

<Peak Table>

PDA Ch1 211nm

Peak#	Ret. Time	Area%
1	11.741	50.107
2	15.504	49.893
Total		100.000

Enantioenriched ester

<Chromatogram>

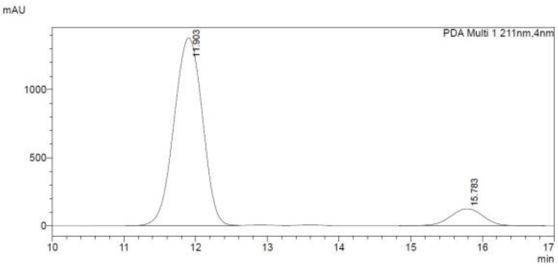

<Peak Table>

PDA Ch1 211nm

Peak#	Ret. Time	Area%
1	11.903	90.587
2	15.783	9.413
Total		100.000

Table 2: KR of benzyl 2-hydroxy-2-(pyridin-2-yl)propanoate 19

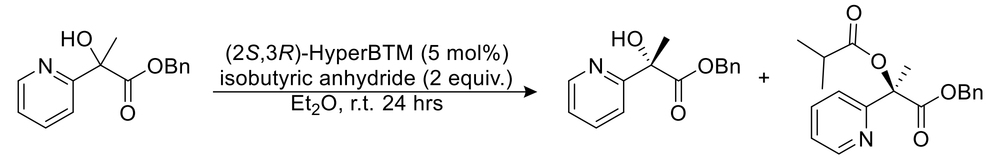

Following general procedure H, **benzyl 2-hydroxy-2-(pyridin-2-yl)propanoate 19** (41 mg, 0.16 mmol), isobutyric anhydride (53 µL, 0.32 mmol) and (2*S*,3*R*)-HyperBTM (2.5 mg, 0.008 mmol, 5 mol%) in Et_2O (0.5 mL) for 24 h gave, after column chromatography (Isolera 4, Et_2O in petrol, 0% to 35% over 30 CV), alcohol (20 mg, 0.08 mmol, 47%) and ester (22 mg, 0.07 mmol, 42%). **c = 51%, *s* = 46**

(*S*)-Benzyl 2-hydroxy-2-(pyridin-2-yl)propanoate (*S*)-19: $[\alpha]_D^{20}$ −6 (*c* 0.14, $CHCl_3$); Chiral HPLC Chiralcel OJ-H (10% *i*PrOH:hexane, flow rate 0.5 mL min^{-1}, 211 nm, 30 °C) T_R: 27.6, 48.3 min, 95.3:4.7 er.

Racemic alcohol

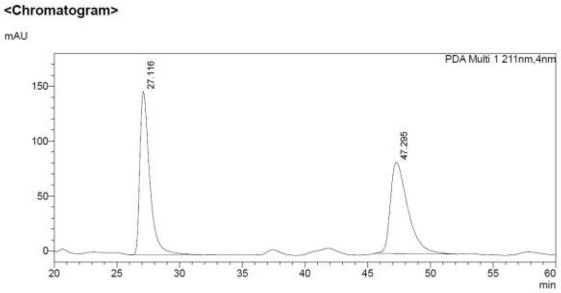

<Peak Table>

PDA Ch1 211nm

Peak#	Ret. Time	Area%
1	27.116	50.160
2	47.295	49.840
Total		100.000

Enantioenriched alcohol

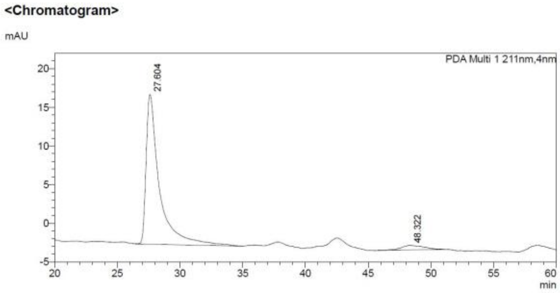

<Peak Table>

PDA Ch1 211nm

Peak#	Ret. Time	Area%
1	27.604	95.273
2	48.322	4.727
Total		100.000

(*R*)-Benzyl 2-(isobutyryloxy)-2-(pyridin-2-yl)propanoate (*R*)-S39: $[\alpha]_D^{20}$ +5 (*c* 0.34, $CHCl_3$); Chiral HPLC Chiralcel OJ-H (5% *i*PrOH:hexane, flow rate 1.0 mL min^{-1}, 211 nm, 30 °C) T_R: 15.4, 17.7 min, 6.3:93.7 er.

Racemic ester

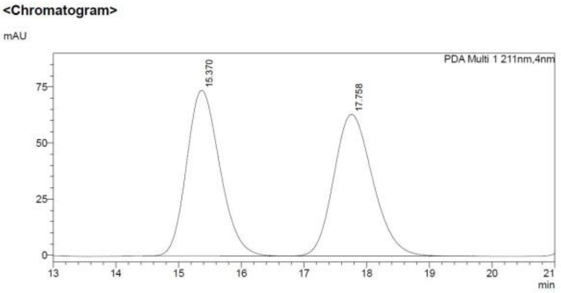

<Peak Table>

PDA Ch1 211nm

Peak#	Ret. Time	Area%
1	15.370	49.883
2	17.758	50.117
Total		100.000

Enantioenriched ester

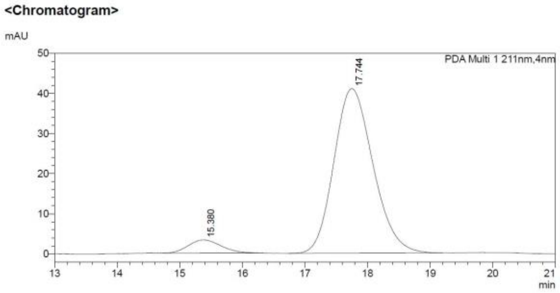

<Peak Table>

PDA Ch1 211nm

Peak#	Ret. Time	Area%
1	15.380	6.308
2	17.744	93.692
Total		100.000

***** Due to the presence of minor impurities in carried on from starting material, using conversion calculated by ^{1}H NMR analysis of crude reaction product mixture, **c = 47%, *s* = 35**

Table 3: KR benzyl 2-hydroxy-2-phenylpent-4-enoate 20

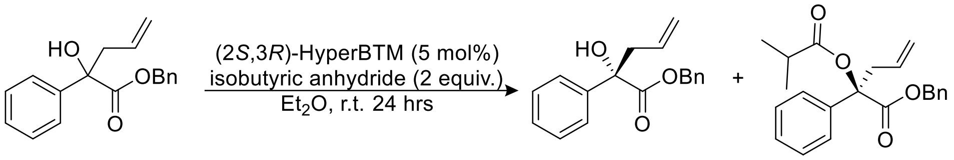

Following general procedure H, **benzyl 2-hydroxy-2-phenylpent-4-enoate 20** (90 mg, 0.32 mmol), isobutyric anhydride (106 µL, 0.64 mmol) and (2*S*,3*R*)-HyperBTM (5 mg, 0.016 mmol, 20 mol%) in Et_2O (1 mL) for 24 h gave, after column chromatography (eluent Petrol/Et_2O, 9:1), alcohol (81 mg, 0.29 mmol, 90%) and ester (2 mg, 0.006 mmol, 2%). **c = 4%, *s* = 13**

(*S*)-Benzyl 2-hydroxy-2-phenylpent-4-enoate (*S*)-20: Chiral HPLC Chiralpak AD-H (1% *i*PrOH:hexane, flow rate 1.0 mL min^{-1}, 211 nm, 30 °C) T_R: 25.9, 30.8 min, 51.6:48.4 er.

Racemic alcohol

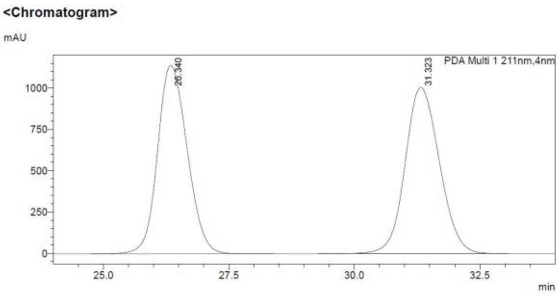

<Peak Table>

PDA Ch1 211nm

Peak#	Ret. Time	Area%
1	26.340	49.397
2	31.323	50.603
Total		100.000

Enantioenriched alcohol

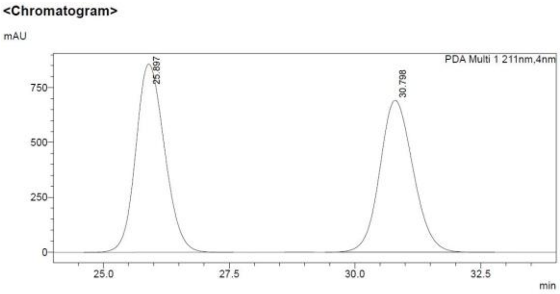

<Peak Table>

PDA Ch1 211nm

Peak#	Ret. Time	Area%
1	25.897	51.640
2	30.798	48.360
Total		100.000

(*R*)-Benzyl 2-(isobutyryloxy)-2-phenylpent-4-enoate (*R*)-S40: Chiral HPLC Chiralpak AD-H (1% *i*PrOH:hexane, flow rate 1.0 mL min^{-1}, 211 nm, 30 °C) T_R: 11.0, 14.8 min, 92.4:7.6 er.

Racemic ester

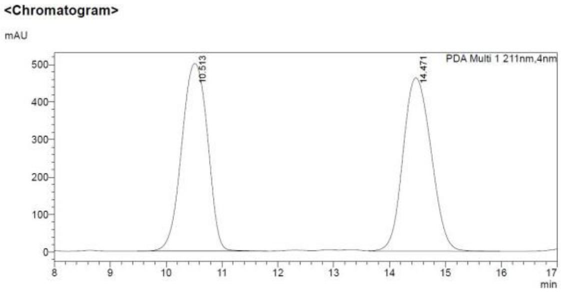

<Peak Table>

PDA Ch1 211nm

Peak#	Ret. Time	Area%
1	10.513	49.959
2	14.471	50.041
Total		100.000

Enantioenriched ester

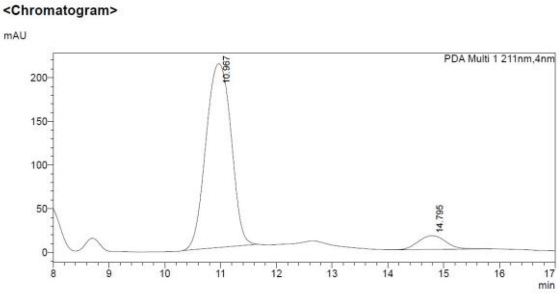

<Peak Table>

PDA Ch1 211nm

Peak#	Ret. Time	Area%
1	10.967	92.410
2	14.795	7.590
Total		100.000

Table 3: KR benzyl 2-hydroxy-2-phenylpent-4-enoate 20

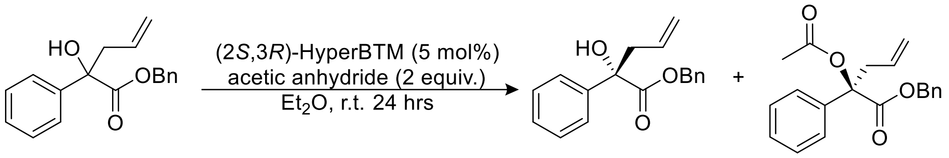

Following general procedure H, **benzyl 2-hydroxy-2-phenylpent-4-enoate 20** (90 mg, 0.32 mmol), acetic anhydride (61 µL, 0.64 mmol) and (2*S*,3*R*)-HyperBTM (5 mg, 0.016 mmol, 20 mol%) in Et_2O (1 mL) for 24 h gave, after column chromatography (eluent Petrol/Et_2O, 9:1), alcohol (49 mg, 0.17 mmol, 54%) and ester (38 mg, 0.12 mmol, 37%). **c = 42%, *s* = 9**

(*S*)-Benzyl 2-hydroxy-2-phenylpent-4-enoate (*S*)-20: $[\alpha]_D^{20}$ +5.1 (*c* 0.1, $CHCl_3$); Chiral HPLC Chiralpak AD-H (1% *i*PrOH:hexane, flow rate 1.0 mL min^{-1}, 211 nm, 30 °C) T_R: 26.6, 31.7 min, 75.3:24.6 er.

Racemic alcohol

<Chromatogram>

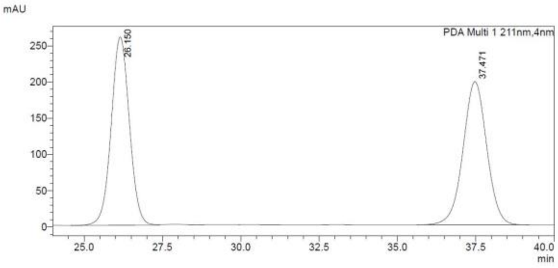

<Peak Table>

PDA Ch1 211nm

Peak#	Ret. Time	Area%
1	26.150	50.094
2	37.471	49.906
Total		100.000

Enantioenriched alcohol

<Chromatogram>

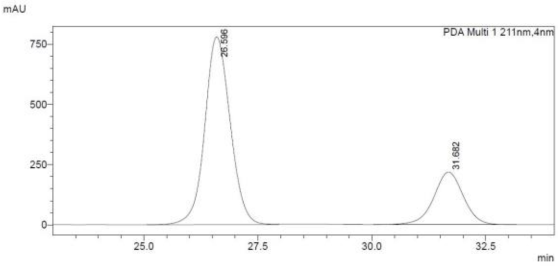

<Peak Table>

PDA Ch1 211nm

Peak#	Ret. Time	Area%
1	26.596	75.381
2	31.682	24.619
Total		100.000

(*R*)-Benzyl 2-acetoxy-2-phenylpent-4-enoate (*R*)-S41: $[\alpha]_D^{20}$ +121 (*c* 0.1, $CHCl_3$); Chiral HPLC Chiralpak AD-H (1% *i*PrOH:hexane, flow rate 1.0 mL min^{-1}, 211 nm, 30 °C) T_R: 13.0, 18.7 min, 84.9:15.1 er.

Racemic ester

<Chromatogram>

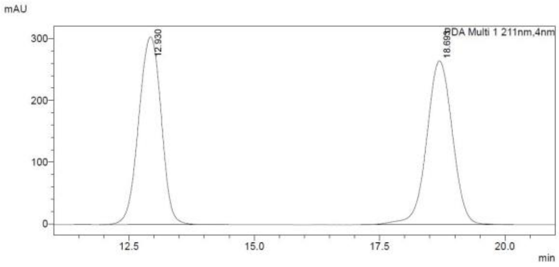

<Peak Table>

PDA Ch1 211nm

Peak#	Ret. Time	Area%
1	12.930	49.999
2	18.693	50.001
Total		100.000

Enantioenriched ester

<Chromatogram>

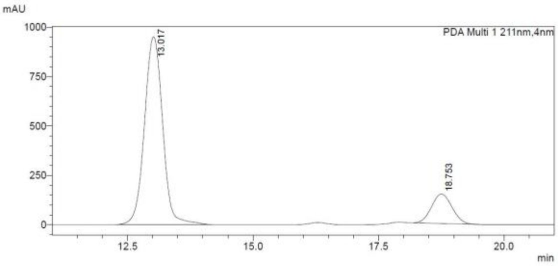

<Peak Table>

PDA Ch1 211nm

Peak#	Ret. Time	Area%
1	13.017	84.943
2	18.753	15.057
Total		100.000

Table 3: KR benzyl 2-hydroxy-2-phenylbutanoate 21

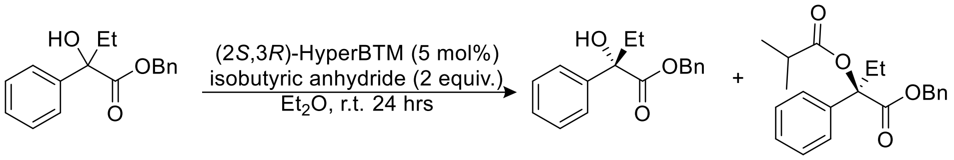

Following general procedure H, **benzyl 2-hydroxy-2-phenylbutanoate 21** (86 mg, 0.32 mmol), isobutyric anhydride (106 µL, 0.64 mmol) and (2*S*,3*R*)-HyperBTM (5 mg, 0.016 mmol, 5 mol%) in Et_2O (1 mL) for 24 h gave, after column chromatography (eluent CH_2Cl_2/Et_2O, 9:1), alcohol (79 mg, 0.29 mmol, 92%) and ester (1 mg, 0.003 mmol, 1%). **c = 1.4%, *s* = 16**

(*S*)-Benzyl 2-hydroxy-2-phenylbutanoate (*S*)-21: Chiral HPLC Chiralpak AD-H (5% *i*PrOH:hexane, flow rate 1.0 mL min^{-1}, 211 nm, 30 °C) T_R: 19.6, 28.8 min, 50.6:49.4 er.

Racemic alcohol

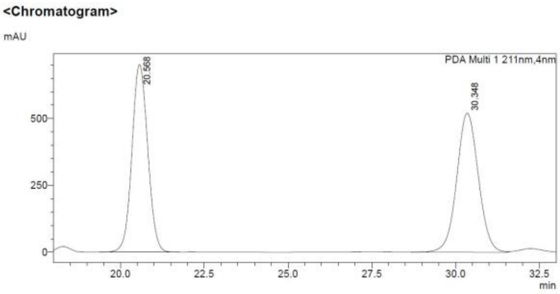

<Peak Table>

PDA Ch1 211nm

Peak#	Ret. Time	Area%
1	20.568	49.653
2	30.348	50.347
Total		100.000

Enantioenriched alcohol

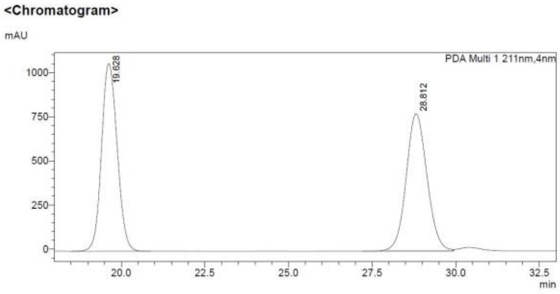

<Peak Table>

PDA Ch1 211nm

Peak#	Ret. Time	Area%
1	19.628	50.608
2	28.812	49.392
Total		100.000

(*R*)-Benzyl 2-(isobutyryloxy)-2-phenylbutanoate (*R*)-S42: $[\alpha]_D^{20}$ –44 (*c* 0.1, $CHCl_3$); Chiral HPLC Chiralpak AD-H (1% *i*PrOH:hexane, flow rate 1.0 mL min^{-1}, 211 nm, 30 °C) T_R: 12.0, 17.0 min, 94.1:5.9 er.

Racemic ester

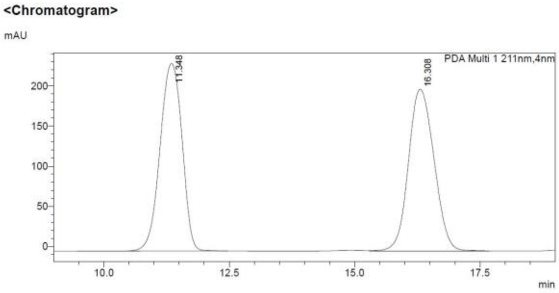

<Peak Table>

PDA Ch1 211nm

Peak#	Ret. Time	Area%
1	11.348	49.963
2	16.308	50.037
Total		100.000

Enantioenriched ester

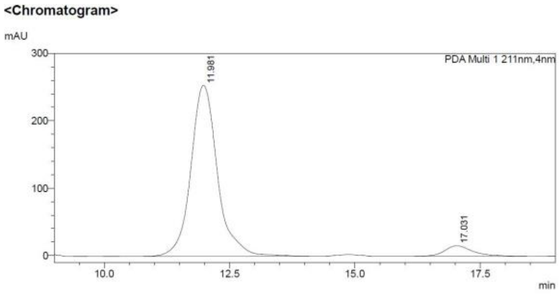

<Peak Table>

PDA Ch1 211nm

Peak#	Ret. Time	Area%
1	11.981	94.110
2	17.031	5.890
Total		100.000

Table 3: Kinetic resolution of benzyl 2-hydroxy-2-phenylbutanoate 21

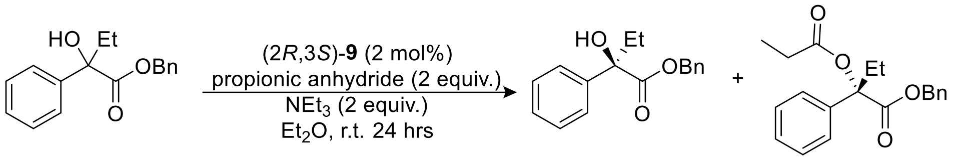

Following general procedure H, **benzyl 2-hydroxy-2-phenylbutanoate 21** (54 mg, 0.2 mmol), propionic anhydride (51 µL, 0.4 mmol), triethylamine (56 µL, 0.4 mmol) and (2*R*,3*S*)-**9** (1.4 mg, 0.004 mmol, 2 mol%) in Et_2O (0.6 mL) overnight gave, after column chromatography (Isolera 4, Et_2O in petrol, 0% to 8% over 35CV), alcohol (23 mg, 0.08 mmol, 41%) and ester (27 mg, 0.08 mmol, 42%). **c = 56%, *s* = 10**

(*R*)-Benzyl 2-hydroxy-2-phenylbutanoate (*R*)-21: $[\alpha]_D^{20}$ +3.4 (*c* 0.4, $CHCl_3$); Chiral HPLC Chiralpak AD-H (5% *i*PrOH:hexane, flow rate 1.0 mL min^{-1}, 211 nm, 30 °C) T_R: 9.9, 13.3 min, 10.7:89.3 er.

Racemic alcohol

<Chromatogram>

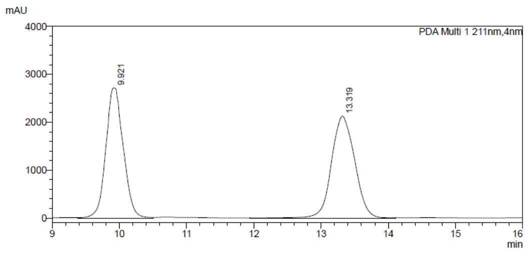

<Peak Table>

PDA Ch1 211nm

Peak#	Ret. Time	Area%
1	9.921	49.005
2	13.319	50.995
Total		100.000

Enantioenriched alcohol

<Chromatogram>

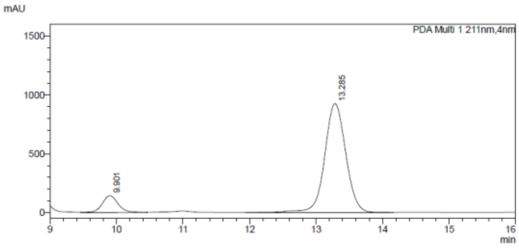

<Peak Table>

PDA Ch1 211nm

Peak#	Ret. Time	Area%
1	9.901	10.653
2	13.285	89.347
Total		100.000

(*S*)-Benzyl 2-acetoxy-2-phenylbutanoate (*S*)-S43: $[\alpha]_D^{20}$ –5.6 (*c* 0.3, $CHCl_3$); Chiral HPLC Chiralpak AD-H (1% *i*PrOH:hexane, flow rate 1.0 mL min^{-1}, 211 nm, 30 °C) T_R: 12.7, 21.0 min, 18.6:81.4 er.

Racemic ester

<Chromatogram>

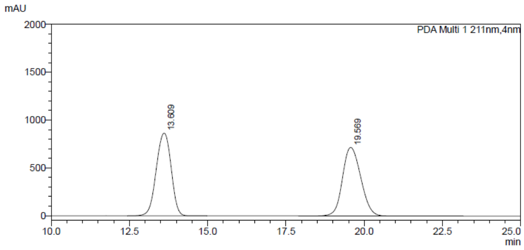

<Peak Table>

PDA Ch1 211nm

Peak#	Ret. Time	Area%
1	13.609	49.527
2	19.569	50.473
Total		100.000

Enantioenriched ester

<Chromatogram>

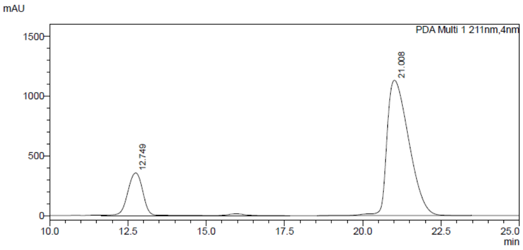

<Peak Table>

PDA Ch1 211nm

Peak#	Ret. Time	Area%
1	12.749	18.572
2	21.008	81.428
Total		100.000

Table 3: KR benzyl 2-hydroxy-2-phenylhexanoate 22

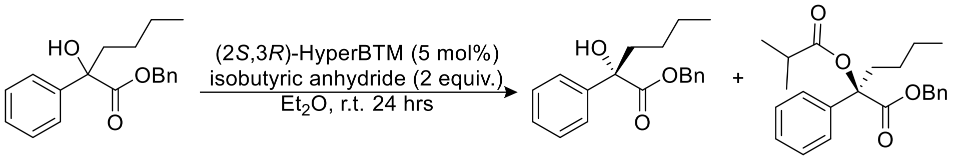

Following general procedure H, **benzyl 2-hydroxy-2-phenylhexanoate 22** (96 mg, 0.32 mmol), isobutyric anhydride (106 µL, 0.64 mmol) and (2*S*,3*R*)-HyperBTM (5 mg, 0.016 mmol, 5 mol%) in Et_2O (1 mL) for 24 h gave, after column chromatography (eluent Petrol/Et_2O, 9:1), alcohol (85 mg, 0.28 mmol, 89%) and ester (1 mg, 0.003 mmol, 1%). **c = 1.5%, *s* = 9**

(*S*)-Benzyl 2-hydroxy-2-phenylhexanoate (*S*)-22: Chiral HPLC Chiralpak AD-H (0.5% *i*PrOH:hexane, flow rate 0.5 mL min^{-1}, 211 nm, 30 °C) T_R: 55.6, 61.0 min, 50.6:49.4 er

Racemic alcohol

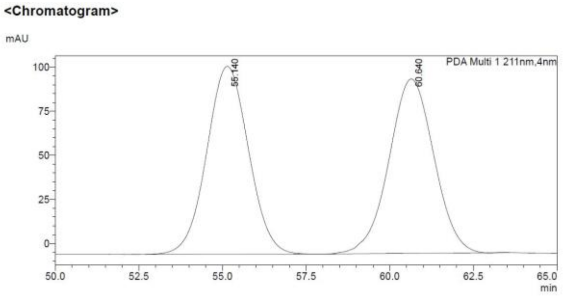

<Peak Table>

PDA Ch1 211nm

Peak#	Ret. Time	Area%
1	55.140	50.037
2	60.640	49.963
Total		100.000

Enantioenriched alcohol

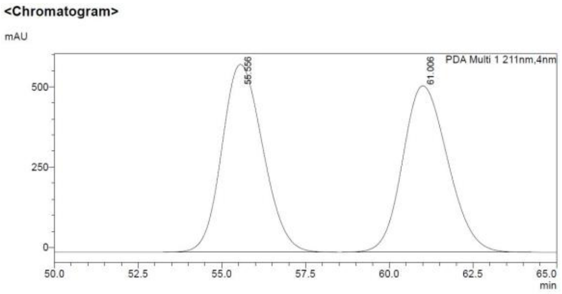

<Peak Table>

PDA Ch1 211nm

Peak#	Ret. Time	Area%
1	55.556	50.617
2	61.006	49.383
Total		100.000

(*R*)-Benzyl 2-(isobutyryloxy)-2-phenylhexanoate (*R*)-S44: $[\alpha]_D^{20}$ +63 (*c* 0.1, $CHCl_3$); Chiral HPLC Chiralpak AD-H (0.5% *i*PrOH:hexane, flow rate 0.5 mL min^{-1}, 211 nm, 30 °C) T_R: 32.1, 36.8 min, 89.5:10.5 er.

Racemic ester

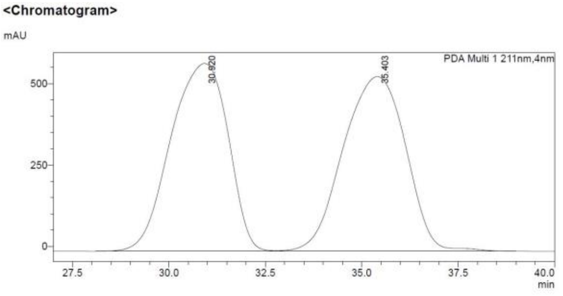

<Peak Table>

PDA Ch1 211nm

Peak#	Ret. Time	Area%
1	30.920	49.638
2	35.403	50.362
Total		100.000

Enantioenriched ester

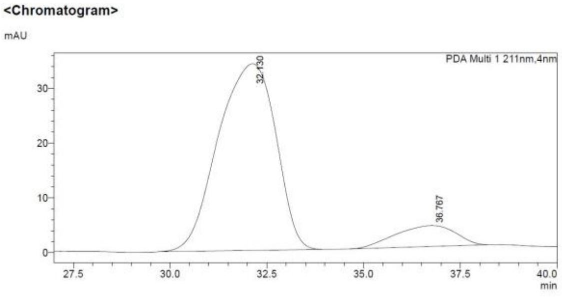

<Peak Table>

PDA Ch1 211nm

Peak#	Ret. Time	Area%
1	32.130	89.532
2	36.767	10.468
Total		100.000

Table 3: Kinetic resolution of benzyl 2-hydroxy-2-phenylhexanoate 22

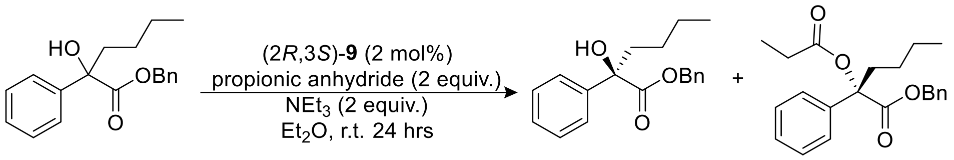

Following general procedure H, **benzyl 2-hydroxy-2-phenylhexanoate 22** (60 mg, 0.2 mmol), propionic anhydride (51 µL, 0.4 mmol), triethylamine (56 µL, 0.4 mmol) and (2*R*,3*S*)-**9** (1.4 mg, 0.004 mmol, 2 mol%) in Et_2O (0.6 mL) overnight gave, after column chromatography (Isolera 4, Et_2O in petrol, 0% to 7% over 35CV), alcohol (21 mg, 0.07 mmol, 35%) and ester (32 mg, 0.09 mmol, 45%). **c = 57%, *s* = 9**

(*R*)-Benzyl 2-hydroxy-2-phenylhexanoate (*R*)-22: $[\alpha]_D^{20}$ –2.0 (*c* 0.3, $CHCl_3$); Chiral HPLC Chiralpak AD-H (0.5% *i*PrOH:hexane, flow rate 0.5 mL min^{-1}, 211 nm, 30 °C) T_R: 57.2, 62.8 min, 10.7:89.3 er.

Racemic alcohol

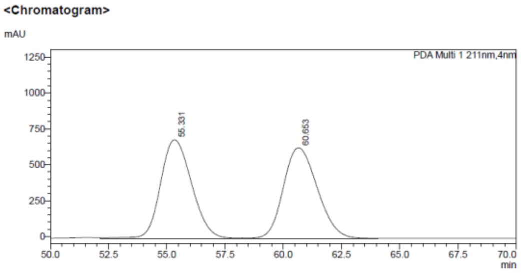

<Peak Table>

PDA Ch1 211nm

Peak#	Ret. Time	Area%
1	55.331	49.964
2	60.653	50.036
Total		100.000

Enantioenriched alcohol

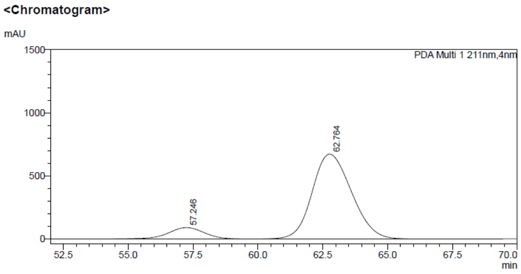

<Peak Table>

PDA Ch1 211nm

Peak#	Ret. Time	Area%
1	57.246	10.730
2	62.764	89.270
Total		100.000

(*S*)-Benzyl 2-acetoxy-2-phenylhexanoate (*S*)-S45: $[\alpha]_D^{20}$ +1.3 (*c* 0.3, $CHCl_3$); Chiral HPLC Chiralpak AD-H (0.5% *i*PrOH:hexane, flow rate 0.5 mL min^{-1}, 211 nm, 30 °C) T_R: 34.6, 48.4 min, 20.8:79.2 er.

Racemic ester

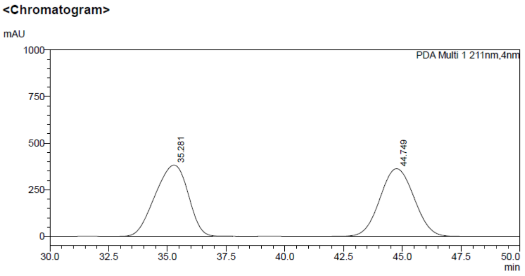

<Peak Table>

PDA Ch1 211nm

Peak#	Ret. Time	Area%
1	35.281	50.491
2	44.749	49.509
Total		100.000

Enantioenriched ester

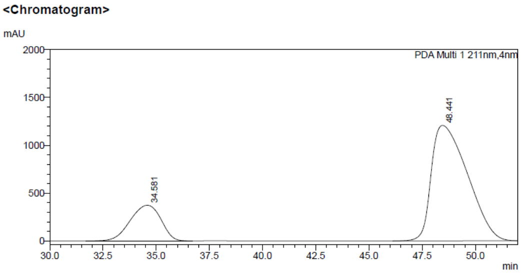

<Peak Table>

PDA Ch1 211nm

Peak#	Ret. Time	Area%
1	34.581	20.847
2	48.441	79.153
Total		100.000

Table 3: KR of benzyl 3,3,3-trifluoro-2-hydroxy-2-phenylpropanoate 23

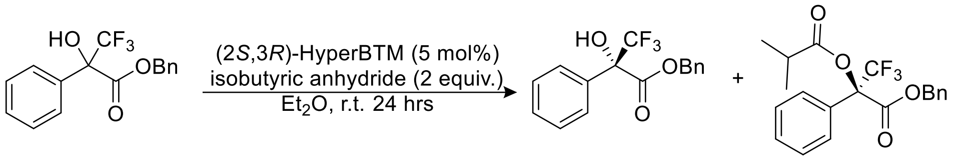

Following general procedure H, **benzyl 3,3,3-trifluoro-2-hydroxy-2-phenylpropanoate 23** (100 mg, 0.32 mmol), isobutyric anhydride (106 µL, 0.64 mmol) and (2*S*,3*R*)-HyperBTM **26** (5 mg, 0.016 mmol, 5 mol%) in Et_2O (1 mL) for 24 h gave, after column chromatography (eluent Petrol/Et_2O, 9:1), alcohol (65 mg, 0.21 mmol, 65%) and ester (24 mg, 0.06 mmol, 20%).
c = 26%, *s* = 5

(*S*)-Benzyl 3,3,3-trifluoro-2-hydroxy-2-phenylpropanoate (*S*)-23: Chiral HPLC Chiralpak AD-H (1% *i*PrOH:hexane, flow rate 1.0 mL min^{-1}, 211 nm, 30 °C) T_R: 23.4, 31.6 min, 60.2:39.8 er.

Racemic alcohol

<Chromatogram>

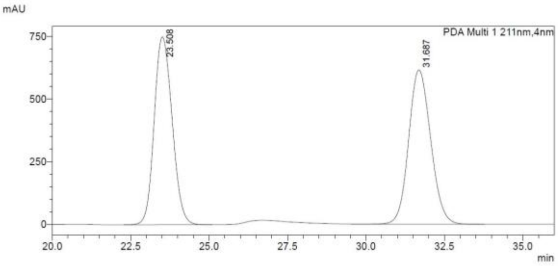

<Peak Table>

PDA Ch1 211nm

Peak#	Ret. Time	Area%
1	23.508	49.814
2	31.687	50.186
Total		100.000

Enantioenriched alcohol

<Chromatogram>

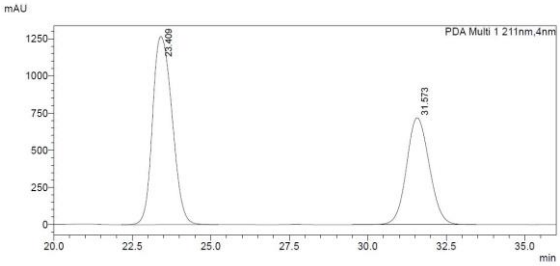

<Peak Table>

PDA Ch1 211nm

Peak#	Ret. Time	Area%
1	23.409	60.173
2	31.573	39.827
Total		100.000

(*R*)-Benzyl 3,3,3-trifluoro-2-(isobutyryloxy)-2-phenylpropanoate (*R*)-S46: $[\alpha]_D^{20}$ +44 (*c* 0.1, $CHCl_3$); Chiral HPLC Chiralpak AD-H (1% *i*PrOH:hexane, flow rate 1.0 mL min^{-1}, 211 nm, 30 °C) T_R: 8.8, 15.3 min, 79.3:20.7 er.

Racemic ester

<Chromatogram>

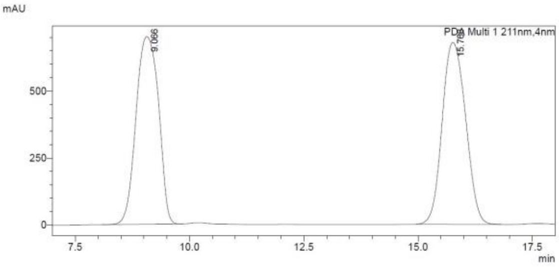

<Peak Table>

PDA Ch1 211nm

Peak#	Ret. Time	Area%
1	9.066	49.800
2	15.763	50.200
Total		100.000

Enantioenriched ester

<Chromatogram>

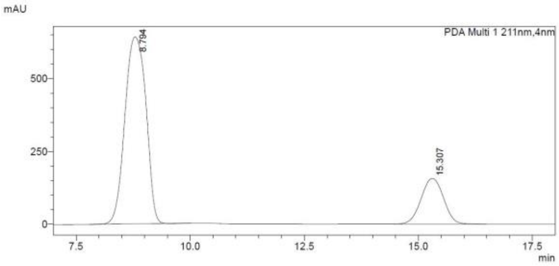

<Peak Table>

PDA Ch1 211nm

Peak#	Ret. Time	Area%
1	8.794	79.273
2	15.307	20.727
Total		100.000

Table 3: Kinetic resolution of benzyl 3,3,3-trifluoro-2-hydroxy-2-phenylpropanoate 23

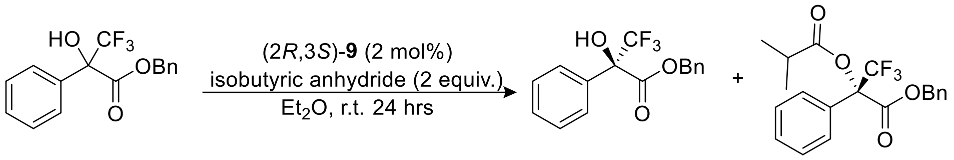

Following general procedure H, **benzyl 3,3,3-trifluoro-2-hydroxy-2-phenylpropanoate 23** (62 mg, 0.2 mmol), isobutyric anhydride (66 µL, 0.4 mmol) and (2*R,*3*S*)- **9** (1.4 mg, 0.004 mmol, 2 mol%) in Et_2O (0.6 mL) overnight gave, after column chromatography (Isolera 4, Et_2O in petrol, 0% to 10% over 35CV), alcohol (23 mg, 0.07 mmol, 38%) and ester (31 mg, 0.08 mmol, 41%). **c = 48%, *s* = 8**

(*R*)-Benzyl 3,3,3-trifluoro-2-hydroxy-2-phenylpropanoate (*R*)-23: $[\alpha]_D^{20}$ +12.8 (*c* 1.1, $CHCl_3$); Chiral HPLC Chiralpak AD-H (1% *i*PrOH:hexane, flow rate 1.0 mL min^{-1}, 211 nm, 30 °C) T_R: 23.6, 31.8 min, 20.5:79.5 er.

Racemic alcohol

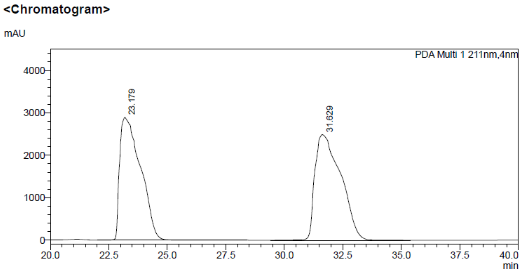

<Peak Table>

PDA Ch1 211nm

Peak#	Ret. Time	Area%
1	23.179	48.425
2	31.629	51.575
Total		100.000

Enantioenriched alcohol

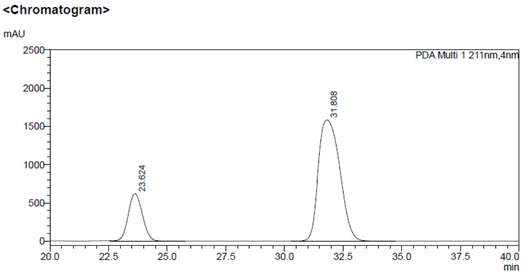

<Peak Table>

PDA Ch1 211nm

Peak#	Ret. Time	Area%
1	23.624	20.543
2	31.808	79.457
Total		100.000

(*S*)-Benzyl 3,3,3-trifluoro-2-(isobutyryloxy)-2-phenylpropanoate (*S*)-S46: $[\alpha]_D^{20}$ –16.7 (*c* 0.6, $CHCl_3$); Chiral HPLC Chiralpak AD-H (1% *i*PrOH:hexane, flow rate 1.0 mL min^{-1}, 211 nm, 30 °C) T_R: 8.7, 15.1 min, 18.4:81.6 er.

Racemic ester

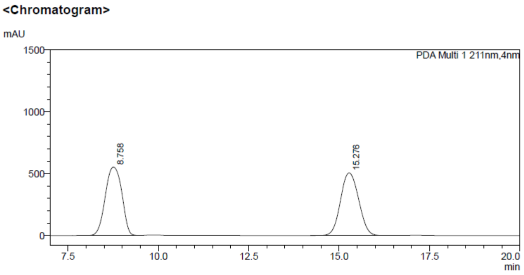

<Peak Table>

PDA Ch1 211nm

Peak#	Ret. Time	Area%
1	8.758	49.762
2	15.276	50.238
Total		100.000

Enantioenriched ester

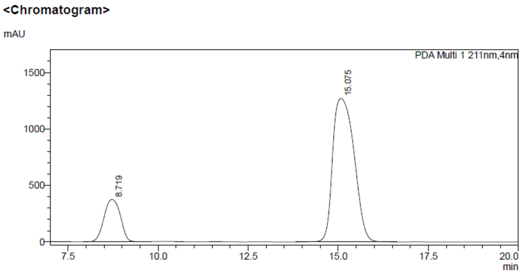

<Peak Table>

PDA Ch1 211nm

Peak#	Ret. Time	Area%
1	8.719	18.448
2	15.075	81.552
Total		100.000

Table 3: KR benzyl 2-hydroxy-2-phenylbut-3-enoate 24

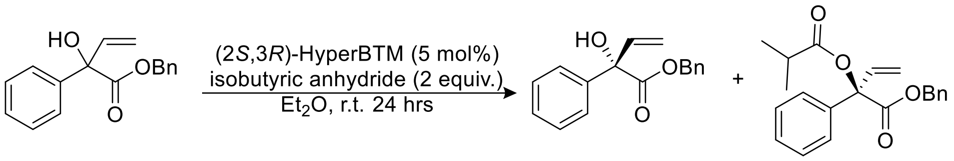

Following general procedure H, **benzyl 2-hydroxy-2-phenylbut-3-enoate 24** (86 mg, 0.32 mmol), isobutyric anhydride (106 µL, 0.64 mmol) and (2*S*,3*R*)-HyperBTM (5 mg, 0.016 mmol, 5 mol%) in Et_2O (1 mL) for 24 h gave, after column chromatography (eluent Petrol/Et_2O, 9:1), alcohol (52 mg, 0.19 mmol, 60%) and ester (29 mg, 0.09 mmol, 27%). **c = 32%, *s* = 22**

(*S*)-Benzyl 2-hydroxy-2-phenylbut-3-enoate (*S*)-24: Chiral HPLC Chiralpak AD-H (1% *i*PrOH:hexane, flow rate 1.0 mL min^{-1}, 211 nm, 30 °C) T_R: 24.2, 34.2 min, 70.2:29.8 er.

Racemic alcohol

<Chromatogram>

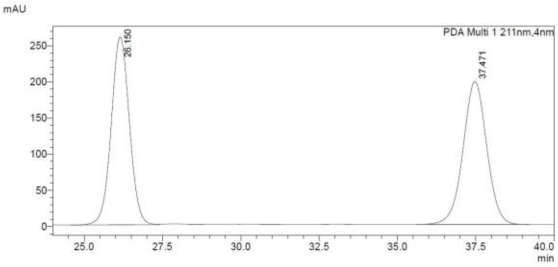

<Peak Table>

PDA Ch1 211nm

Peak#	Ret. Time	Area%
1	26.150	50.094
2	37.471	49.906
Total		100.000

Enantioenriched alcohol

<Chromatogram>

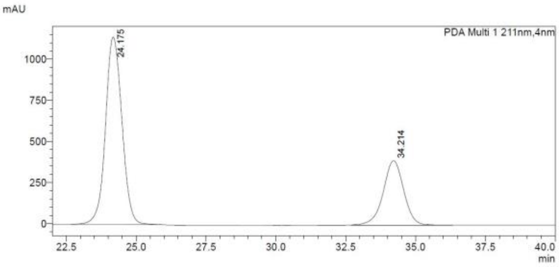

<Peak Table>

PDA Ch1 211nm

Peak#	Ret. Time	Area%
1	24.175	70.224
2	34.214	29.776
Total		100.000

(*R*)-Benzyl 2-(isobutyryloxy)-2-phenylbut-3-enoate (*R*)-S47: $[\alpha]_D^{20}$ +10 (*c* 0.1, $CHCl_3$); Chiral HPLC Chiralpak AD-H (1% *i*PrOH:hexane, flow rate 1.0 mL min^{-1}, 211 nm, 30 °C) T_R: 12.9, 18.3 min, 93.8:6.2 er.

Racemic ester

<Chromatogram>

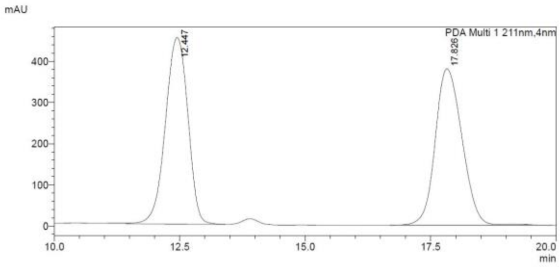

<Peak Table>

PDA Ch1 211nm

Peak#	Ret. Time	Area%
1	12.447	50.102
2	17.826	49.898
Total		100.000

Enantioenriched ester

<Chromatogram>

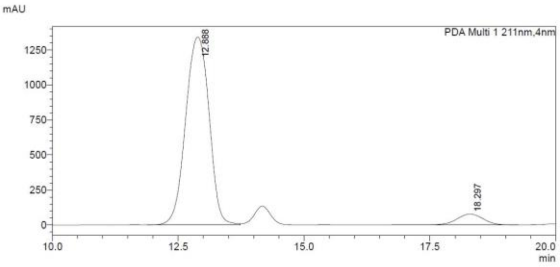

<Peak Table>

PDA Ch1 211nm

Peak#	Ret. Time	Area%
1	12.888	93.775
2	18.297	6.225
Total		100.000

Table 3: Kinetic resolution of benzyl 2-hydroxy-2-phenylbut-3-enoate 24

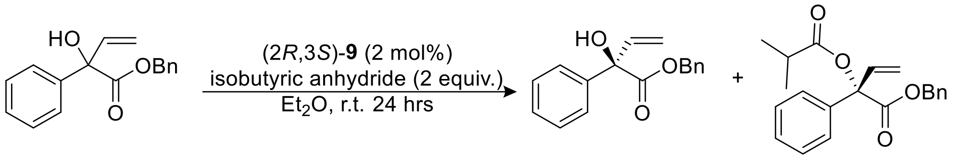

Following general procedure H, **benzyl 2-hydroxy-2-phenylbut-3-enoate 24** (54 mg, 0.2 mmol), isobutyric anhydride (66 µL, 0.4 mmol) and (2*R*,3*S*)-**9** (1.4 mg, 0.004 mmol, 2 mol%) in Et_2O (0.6 mL) overnight gave, after column chromatography (Isolera 4, Et_2O in petrol, 0% to 10% over 30CV), alcohol (22 mg, 0.08 mmol, 40%) and ester (28 mg, 0.08 mmol, 41%). **c = 45%, *s* = 20**

(*R*)-Benzyl 2-hydroxy-2-phenylbut-3-enoate (*R*)-24: $[\alpha]_D^{20}$ –8.7 (*c* 0.9, $CHCl_3$); Chiral HPLC Chiralpak AD-H (1% *i*PrOH:hexane, flow rate 1.0 mL min^{-1}, 211 nm, 30 °C) T_R: 24.7, 35.2 min, 16.2:83.8 er.

Racemic alcohol

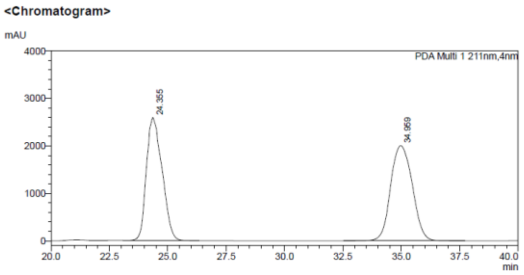

<Peak Table>

PDA Ch1 211nm

Peak#	Ret. Time	Area%
1	24.355	48.489
2	34.959	51.511
Total		100.000

Enantioenriched alcohol

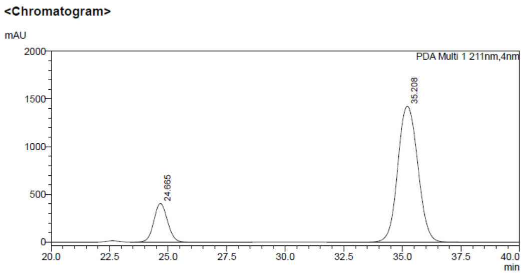

<Peak Table>

PDA Ch1 211nm

Peak#	Ret. Time	Area%
1	24.665	16.210
2	35.208	83.790
Total		100.000

(*S*)-Benzyl 2-(isobutyryloxy)-2-phenylbut-3-enoate (*S*)-S47: $[\alpha]_D^{20}$ +3.6 (*c* 0.9, $CHCl_3$); Chiral HPLC Chiralpak AD-H (1% *i*PrOH:hexane, flow rate 1.0 mL min^{-1}, 211 nm, 30 °C) T_R: 13.1, 18.4 min, 9.4:90.6 er.

Racemic ester

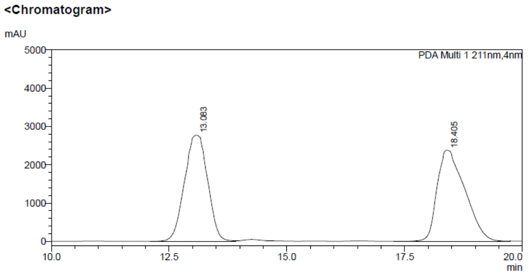

<Peak Table>

PDA Ch1 211nm

Peak#	Ret. Time	Area%
1	13.083	49.280
2	18.405	50.720
Total		100.000

Enantioenriched ester

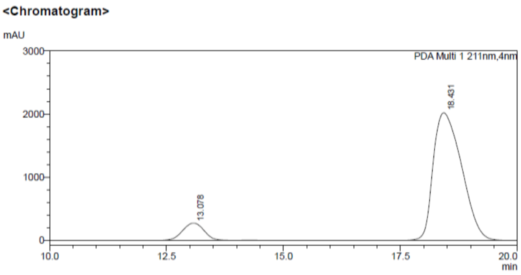

<Peak Table>

PDA Ch1 211nm

Peak#	Ret. Time	Area%
1	13.078	9.365
2	18.431	90.635
Total		100.000

Table 3: KR benzyl 4-cyclopropyl-2-hydroxy-2-phenybut-3-ynoate 25

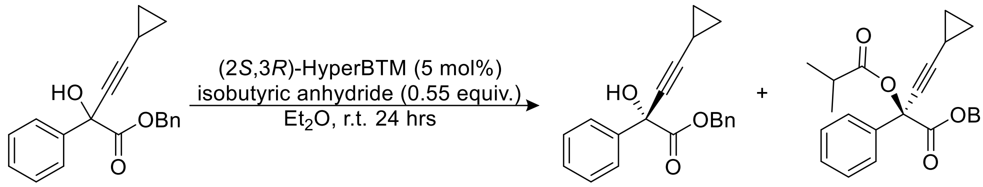

Following general procedure H, **benzyl 4-cyclopropyl-2-hydroxy-2-phenybut-3-ynoate 25** (98 mg, 0.32 mmol), isobutyric anhydride (29 µL, 0.18 mmol) and (2*S*,3*R*)-HyperBTM (5 mg, 0.016 mmol, 5 mol%) in Et_2O (1 mL) for 24 h gave, after column chromatography (eluent Petrol/Et_2O, 9:1), alcohol (44 mg, 0.14 mmol, 45%) and ester (48 mg, 0.13 mmol, 40%).
c = 47%, *s* = 6

(*R*)-Benzyl 4-cyclopropyl-2-hydroxy-2-phenybut-3-ynoate (*S*)-25: $[\alpha]_D^{20}$ +29 (*c* 0.1, $CHCl_3$); Chiral HPLC Chiralcel OD-H (1% *i*PrOH:hexane, flow rate 1.0 mL min^{-1}, 211 nm, 30 °C) T_R: 33.9, 49.0 min, 76:24 er.

Racemic alcohol

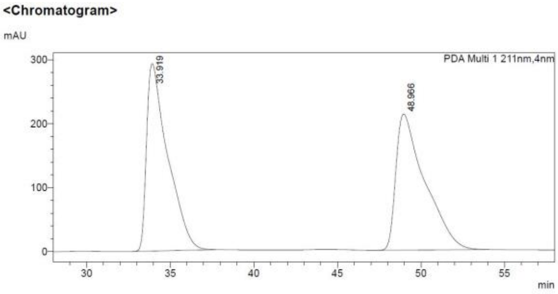

<Peak Table>

PDA Ch1 211nm

Peak#	Ret. Time	Area%
1	33.919	50.092
2	48.966	49.908
Total		100.000

Enantioenriched alcohol

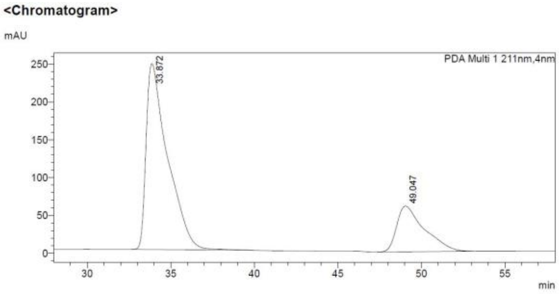

<Peak Table>

PDA Ch1 211nm

Peak#	Ret. Time	Area%
1	33.872	76.020
2	49.047	23.980
Total		100.000

(*S*)-Benzyl 4-cyclopropyl-2-(isobutyryloxy)-2-phenylbut-3-ynoate (*R*)-S48: $[\alpha]_D^{20}$ –10 (*c* 0.1, $CHCl_3$); Chiral HPLC Chiralpak AD-H (1% *i*PrOH:hexane, flow rate 1.0 mL min^{-1}, 211 nm, 30 °C) T_R: 21.1, 23.9 min, 79.3:20.7 er.

Racemic ester

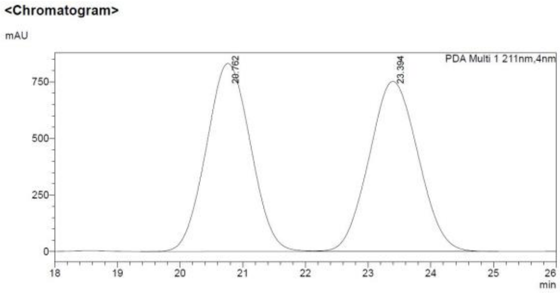

<Peak Table>

PDA Ch1 211nm

Peak#	Ret. Time	Area%
1	20.762	49.971
2	23.394	50.029
Total		100.000

Enantioenriched ester

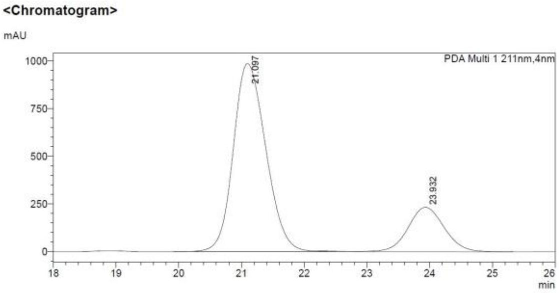

<Peak Table>

PDA Ch1 211nm

Peak#	Ret. Time	Area%
1	21.097	79.259
2	23.932	20.741
Total		100.000

Table 4: KR of benzyl 4-cyclopropyl-2-hydroxy-2-methylbut-3-ynoate 26

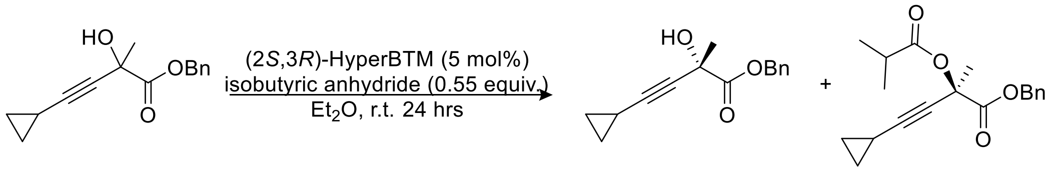

Absolute configuration of products not unambiguously confirmed

Following general procedure H, **benzyl 4-cyclopropyl-2-hydroxy-2-methylbut-3-ynoate 26** (78 mg, 0.32 mmol), isobutyric anhydride (29 µL, 0.18 mmol) and (2*S*,3*R*)-HyperBTM (5 mg, 0.016 mmol, 5 mol%) in Et_2O (1 mL) for 24 h gave, after column chromatography (eluent Petrol/Et_2O, 9:1), alcohol (37 mg, 0.15 mmol, 48%) and ester (34 mg, 0.11 mmol, 34%).
c = 41%, *s* = 2

Benzyl 4-cyclopropyl-2-hydroxy-2-methylbut-3-ynoate 26: $[\alpha]_D^{20}$ +34 (*c* 0.1, $CHCl_3$); Chiral HPLC Chiralpak AD-H (1% *i*PrOH:hexane, flow rate 1.0 mL min^{-1}, 211 nm, 30 °C) T_R: 35.6, 42.2 min, 54.9:45.1 er.

Racemic alcohol

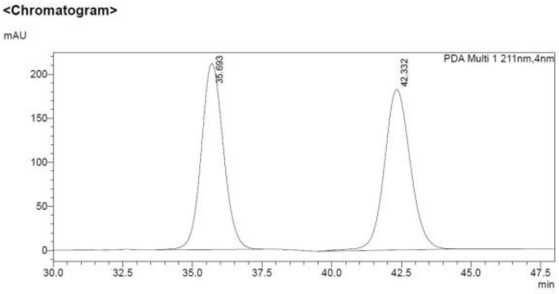

<Peak Table>

PDA Ch1 211nm

Peak#	Ret. Time	Area%
1	35.693	49.504
2	42.332	50.496
Total		100.000

Enantioenriched alcohol

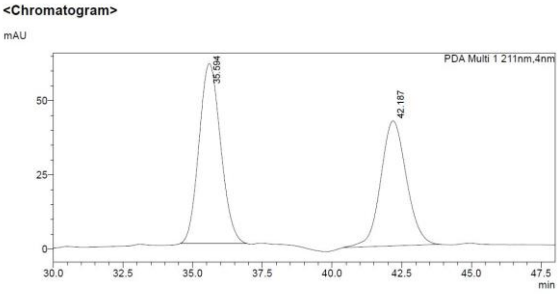

<Peak Table>

PDA Ch1 211nm

Peak#	Ret. Time	Area%
1	35.594	54.859
2	42.187	45.141
Total		100.000

Benzyl 4-cyclopropyl-2-(isobutyryloxy)-2-methylbut-3-ynoate S49: $[\alpha]_D^{20}$ +106 (*c* 0.1, $CHCl_3$); Chiral HPLC Chiralcel OD-H (0.5% *i*PrOH:hexane, flow rate 1.0 mL min^{-1}, 211 nm, 30 °C) T_R: 10.5, 12.8 min, 42.9:57.1 er.

Racemic ester

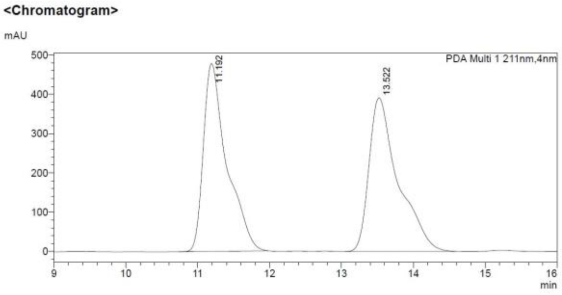

<Peak Table>

PDA Ch1 211nm

Peak#	Ret. Time	Area%
1	11.192	49.555
2	13.522	50.445
Total		100.000

Enantioenriched ester

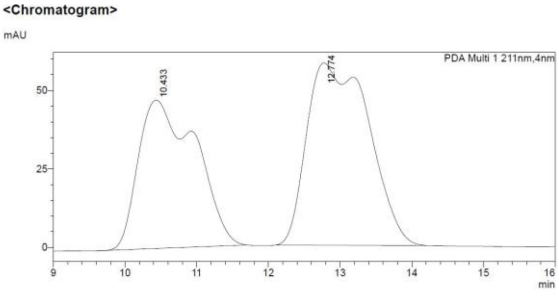

<Peak Table>

PDA Ch1 211nm

Peak#	Ret. Time	Area%
1	10.433	42.931
2	12.774	57.069
Total		100.000

***** the HPLC traces of the amide containing ester showed unusual peak broadening (presumably due to rotameric species) but we believe allow unambiguous determination of enantiomeric ratios.

Table 4: KR of benzyl 2-hydroxy-2-methylbut-3-enoate 27

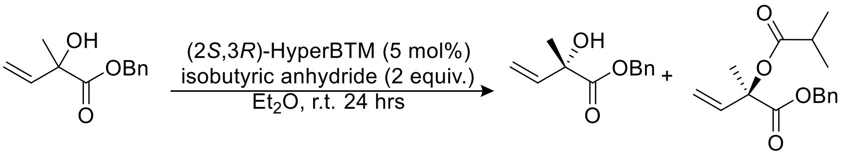

Absolute configuration of products not unambiguously confirmed

Following general procedure H, **benzyl 2-hydroxy-2-methylbut-3-enoate 27** (33 mg, 0.16 mmol), isobutyric anhydride (53 µL, 0.32 mmol) and (2*S*,3*R*)-HyperBTM (2.5 mg, 0.008 mmol, 5 mol%) in Et_2O (0.5 mL) for 24 h gave, after column chromatography (Isolera 4, Et_2O in petrol, 0% to 10% over 32 CV), alcohol (11 mg, 0.05 mmol, 32%) and ester (22 mg, 0.08 mmol, 50%). **c = 62%, *s* = 7**

Benzyl 2-hydroxy-2-methylbut-3-enoate 27: $[\alpha]_D^{20}$ −5 (*c* 0.1, $CHCl_3$); Chiral HPLC Chiralcel OJ-H (1% *i*PrOH:hexane, flow rate 0.5 mL min^{-1}, 211 nm, 30 °C) T_R: 20.3, 23.4 min, 91.1:8.9 er.

Racemic alcohol

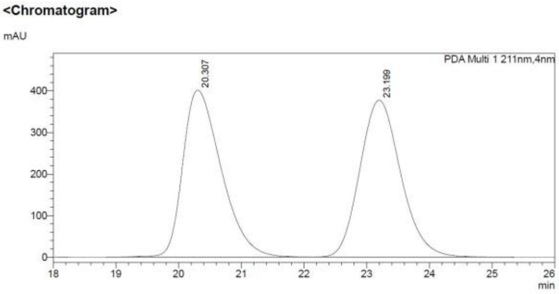

<Peak Table>

PDA Ch1 211nm

Peak#	Ret. Time	Area%
1	20.307	49.955
2	23.199	50.045
Total		100.000

Enantioenriched alcohol

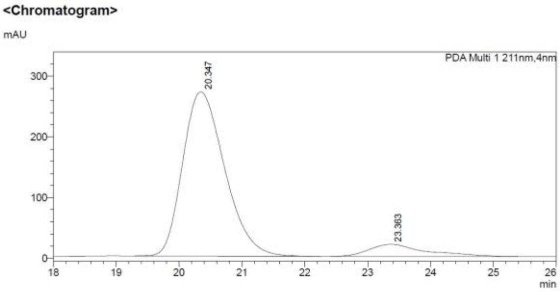

<Peak Table>

PDA Ch1 211nm

Peak#	Ret. Time	Area%
1	20.347	91.143
2	23.363	8.857
Total		100.000

Benzyl 2-(isobutyryloxy)-2-methylbut-3-enoate S50: $[\alpha]_D^{20}$ +5 (*c* 0.6, $CHCl_3$).
HPLC conditions could not be identified for separation of the ester, therefore conversion calculated by ^1H NMR analysis of crude reaction product mixture.

Table 4: KR of benzyl 2-hydroxy-2,4-dimethylpent-3-enoate 28

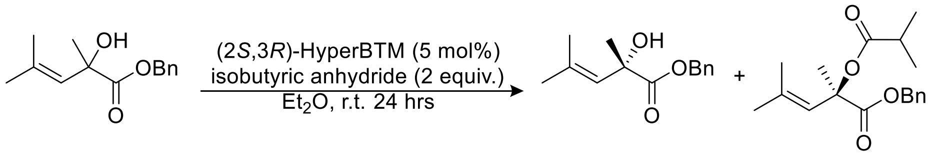

Following general procedure H, **benzyl 2-hydroxy-2,4-dimethylpent-3-enoate 28** (75 mg, 0.32 mmol), isobutyric anhydride (106 µL, 0.64 mmol) and (2*S*,3*R*)-HyperBTM (5 mg, 0.016 mmol, 5 mol%) in Et_2O (1 mL) for 24 h gave, after column chromatography (Isolera 4, Et_2O in petrol, 0% to 15% over 25 CV), alcohol (46 mg, 0.2 mmol, 61%) and ester (21 mg, 0.07 mmol, 21%). **c = 27%, *s* = 11**

(*S*)-Benzyl 2-hydroxy-2,4-dimethylpent-3-enoate (*S*)-28: $[\alpha]_D^{20}$ −17 (*c* 1.3, $CHCl_3$); Chiral HPLC Chiralpak AD-H (1% *i*PrOH:hexane, flow rate 1.0 mL min^{-1}, 211 nm, 30 °C) T_R: 16.3, 19.7 min, 64.5:35.5 er.

Racemic alcohol

<Chromatogram>

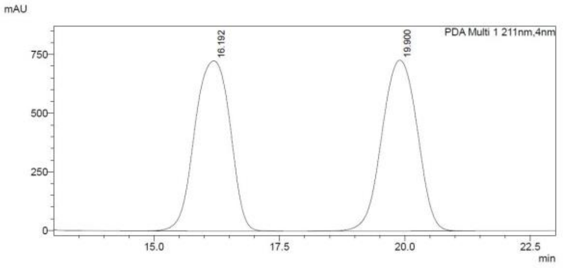

<Peak Table>

PDA Ch1 211nm

Peak#	Ret. Time	Area%
1	16.192	49.964
2	19.900	50.036
Total		100.000

Enantioenriched alcohol

<Chromatogram>

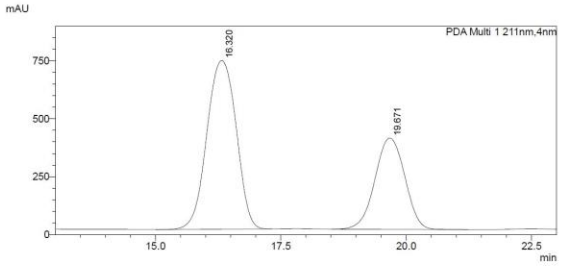

<Peak Table>

PDA Ch1 211nm

Peak#	Ret. Time	Area%
1	16.320	64.512
2	19.671	35.488
Total		100.000

(*R*)-Benzyl 2-(isobutyryloxy)-2,4-dimethylpent-3-enoate (*R*)-S51: $[\alpha]_D^{20}$ +35 (*c* 0.6, $CHCl_3$); Chiral HPLC Chiralpak AD-H (1% *i*PrOH:hexane, flow rate 0.5 mL min^{-1}, 211 nm, 30 °C) T_R: 14.5, 16.9 min, 89.7:10.3 er.

Racemic ester

<Chromatogram>

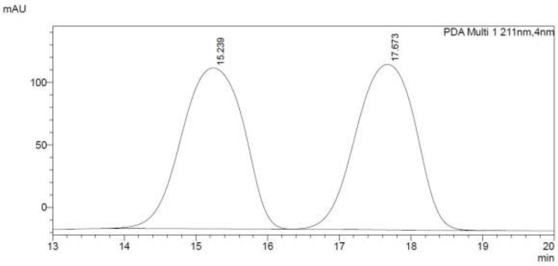

<Peak Table>

PDA Ch1 211nm

Peak#	Ret. Time	Area%
1	15.239	50.150
2	17.673	49.850
Total		100.000

Enantioenriched ester

<Chromatogram>

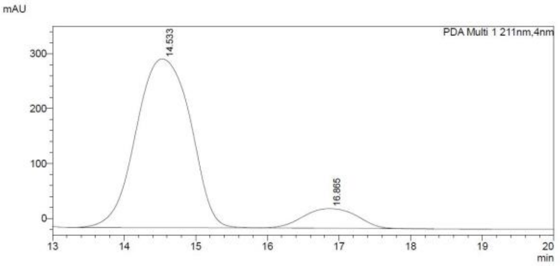

<Peak Table>

PDA Ch1 211nm

Peak#	Ret. Time	Area%
1	14.533	89.692
2	16.865	10.308
Total		100.000

Table 4: KR of benzyl 2-hydroxy-2,4-dimethylpent-3-enoate 28

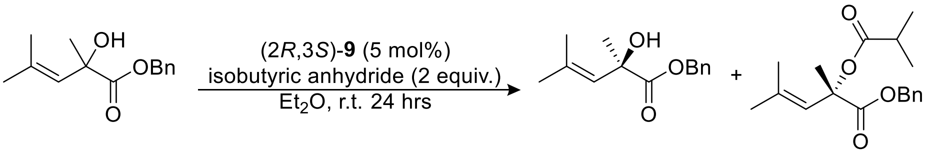

Following general procedure H, **benzyl 2-hydroxy-2,4-dimethylpent-3-enoate 28** (38 mg, 0.16 mmol), isobutyric anhydride (53 µL, 0.32 mmol) and (2*R*,3*S*)-**9** (5 mg, 0.016 mmol, 5 mol%) in Et_2O (0.5 mL) for 24 h gave, after column chromatography (Isolera 4, Et_2O in petrol, 0% to 15% over 25 CV), alcohol (19 mg, 0.8 mmol, 49%) and ester (21 mg, 0.07 mmol, 43%). **c = 47%, *s* = 10**

(*R*)-Benzyl 2-hydroxy-2,4-dimethylpent-3-enoate (*R*)-28: $[\alpha]_D^{20}$ +37 (*c* 0.5, $CHCl_3$); Chiral HPLC Chiralpak AD-H (1% *i*PrOH:hexane, flow rate 1 mL min^{-1}, 211 nm, 30 °C) T_R: 16.3, 19.9 min, 19.5:80.5 er.

Racemic alcohol

<Chromatogram>

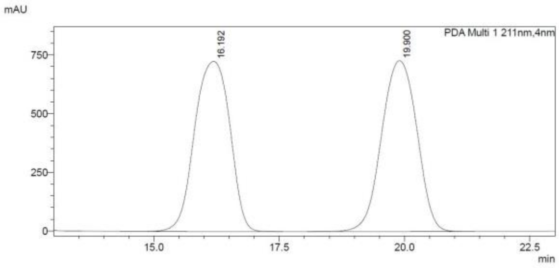

<Peak Table>

PDA Ch1 211nm

Peak#	Ret. Time	Area%
1	16.192	49.964
2	19.900	50.036
Total		100.000

Enantioenriched alcohol

<Chromatogram>

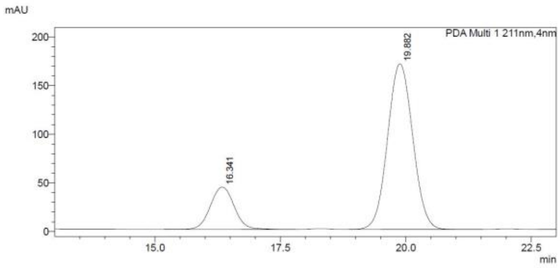

<Peak Table>

PDA Ch1 211nm

Peak#	Ret. Time	Area%
1	16.341	19.489
2	19.882	80.511
Total		100.000

(*S*)-Benzyl 2-(isobutyryloxy)-2,4-dimethylpent-3-enoate (*S*)-S51: $[\alpha]_D^{20}$ −33 (*c* 0.4, $CHCl_3$); Chiral HPLC Chiralpak AD-H (1% *i*PrOH:hexane, flow rate 0.5 mL min^{-1}, 211 nm, 30 °C) T_R: 15.3, 17.1 min, 15.6:84.4 er.

Racemic ester

<Chromatogram>

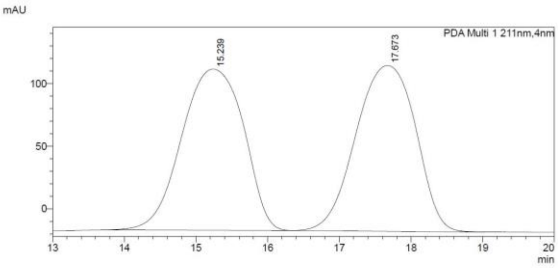

<Peak Table>

PDA Ch1 211nm

Peak#	Ret. Time	Area%
1	15.239	50.150
2	17.673	49.850
Total		100.000

Enantioenriched ester

<Chromatogram>

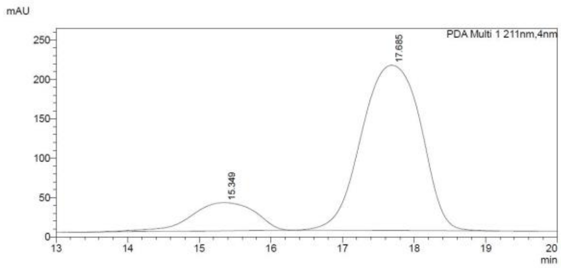

<Peak Table>

PDA Ch1 211nm

Peak#	Ret. Time	Area%
1	15.349	15.644
2	17.685	84.356
Total		100.000

Table 4: KR of benzyl 2-hydroxy-2,3-dimethylbut-3-enoate 29

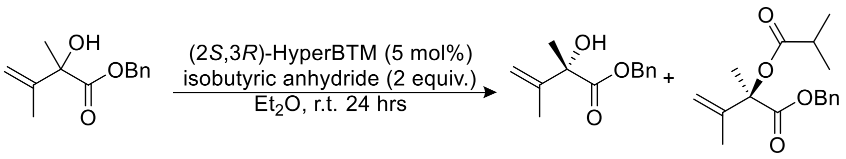

Following general procedure H, **benzyl 2-hydroxy-2,3-dimethylbut-3-enoate 29** (35 mg, 0.16 mmol), isobutyric anhydride (53 µL, 0.32 mmol) and (2*S*,3*R*)-HyperBTM (2.5 mg, 0.008 mmol, 5 mol%) in Et_2O (0.5 mL) for 24 h gave, after column chromatography (Isolera 4, Et_2O in petrol, 0% to 10% over 30 CV), alcohol (19 mg, 0.09 mmol, 53%) and ester (14 mg, 0.05 mmol, 30%). **c = 39%, *s* > 200** (calculated as *s* = 1815)

(*S*)-Benzyl 2-hydroxy-2,3-dimethylbut-3-enoate (*S*)-29: $[\alpha]_D^{20}$ +10 (*c* 0.4, $CHCl_3$); Chiral HPLC Chiralcel OJ-H (1% *i*PrOH:hexane, flow rate 0.5 mL min^{-1}, 211 nm, 30 °C) T_R: 18.9, 24.9 min, 81.3:18.7 er.

Racemic alcohol

<Chromatogram>

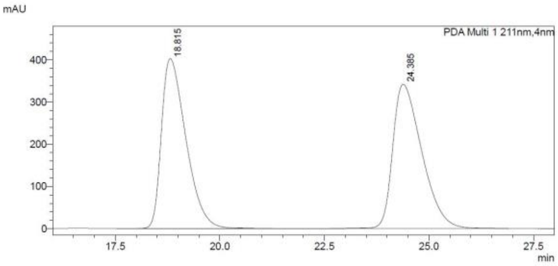

<Peak Table>

PDA Ch1 211nm

Peak#	Ret. Time	Area%
1	18.815	49.822
2	24.385	50.178
Total		100.000

Enantioenriched alcohol

<Chromatogram>

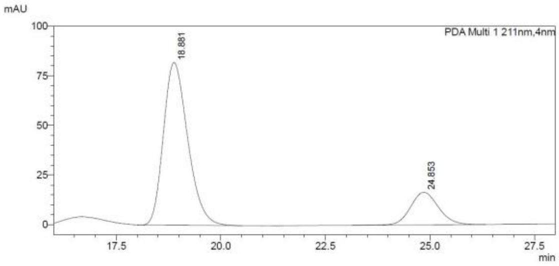

<Peak Table>

PDA Ch1 211nm

Peak#	Ret. Time	Area%
1	18.881	81.290
2	24.853	18.710
Total		100.000

(*R*)-Benzyl 2-(isobutyryloxy)-2,3-dimethylbut-3-enoate (*R*)-S52: $[\alpha]_D^{20}$ +14 (*c* 0.4, $CHCl_3$); Chiral HPLC Chiralpak AD-H (0.5% *i*PrOH:hexane, flow rate 0.5 mL min^{-1}, 211 nm, 30 °C) T_R: 16.7, 20.0 min, 99.9:0.1 er.

Racemic ester

<Chromatogram>

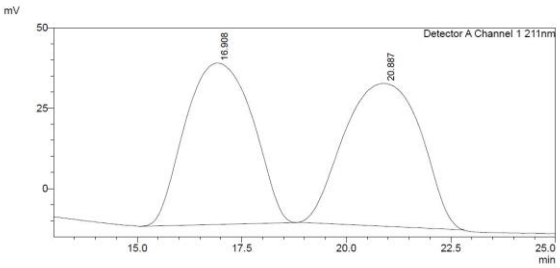

<Peak Table>

Detector A Channel 1 211nm

Peak#	Ret. Time	Area%
1	16.908	49.761
2	20.887	50.239
Total		100.000

Enantioenriched ester

<Chromatogram>

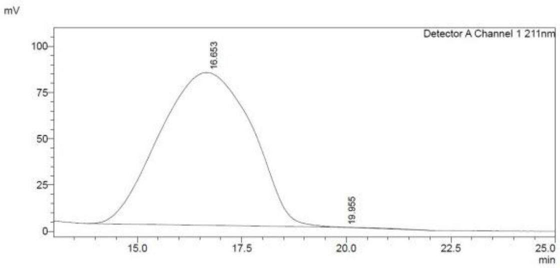

<Peak Table>

Detector A Channel 1 211nm

Peak#	Ret. Time	Area%
1	16.653	99.895
2	19.955	0.105
Total		100.000

Table 4: KR of benzyl 2-cyclopentyl-2-hydroxypropanoate 30

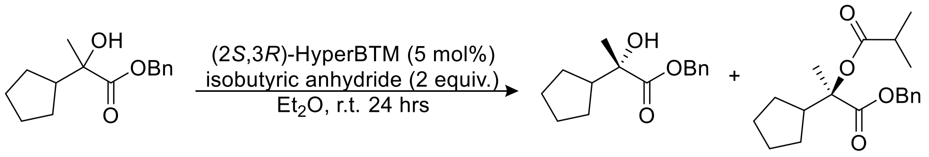

Following general procedure H, **benzyl 2-cyclopentyl-2-hydroxypropanoate 30** (42.0 mg, 0.16 mmol), isobutyric anhydride (53 µL, 0.32 mmol) and (2*S*,3*R*)-HyperBTM (2.5 mg, 0.008 mmol, 5 mol%) in Et_2O (0.5 mL) for 24 h gave, after column chromatography (Isolera 4, Et_2O in petrol, 0% to 10% over 25 CV), alcohol (29.7 mg, 0.12 mmol, 75%) and ester (9.6 mg, 0.03 mmol, 19%). **c = 19%, *s* = 12**

(*S*)-Benzyl 2-cyclopentyl-2-hydroxypropanoate (*S*)-30: $[\alpha]_D^{20}$ −3 (*c* 0.6, $CHCl_3$); Chiral HPLC Chiralcel OJ-H (0.5% *i*PrOH:hexane, flow rate 0.5 mL min^{-1}, 211 nm, 30 °C) T_R: 17.0, 22.2 min, 59.6:40.4 er;

Racemic alcohol

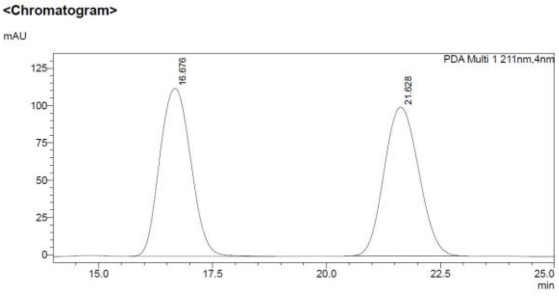

<Peak Table>

PDA Ch1 211nm

Peak#	Ret. Time	Area%
1	16.676	50.181
2	21.628	49.819
Total		100.000

Enantioenriched alcohol

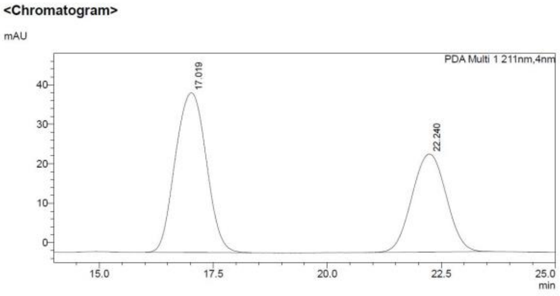

<Peak Table>

PDA Ch1 211nm

Peak#	Ret. Time	Area%
1	17.019	59.596
2	22.240	40.404
Total		100.000

(*R*)-Benzyl 2-cyclopentyl-2-(isobutyryloxy)propanoate (*R*)-S53: $[\alpha]_D^{20}$ −4 (*c* 0.1, $CHCl_3$).
HPLC conditions could not be identified for separation of the ester, therefore conversion calculated by ^{1}H NMR analysis of crude reaction product mixture.

Table 4: KR of benzyl 2-cyclopentyl-2-hydroxypropanoate 30

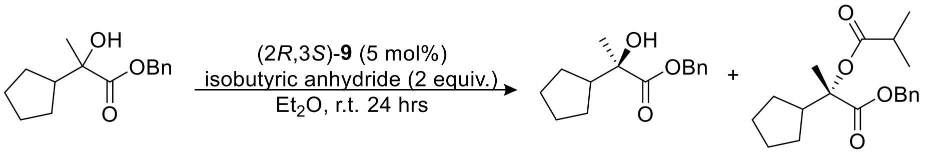

Following general procedure H, **benzyl 2-cyclopentyl-2-hydroxypropanoate 31** (31 mg, 0.125 mmol), isobutyric anhydride (41 µL, 0.25 mmol) and (2*R*,3*S*)-**9** (2.2 mg, 0.006 mmol, 5 mol%) in Et_2O (0.5 mL) for 24 h gave, after column chromatography (Isolera 4, Et_2O in petrol, 0% to 10% over 25 CV), alcohol (16 mg, 0.06 mmol, 50%) and ester (14 mg, 0.04 mmol, 35%). **c = 40%, *s* = 24**

(*R*)-Benzyl 2-cyclopentyl-2-hydroxypropanoate (*S*)-30: Chiral HPLC Chiralcel OJ-H (0.5% *i*PrOH:hexane, flow rate 0.5 mL min^{-1}, 211 nm, 30 °C) T_R: 16.9, 22.0 min, 21.3:78.7 er;

Racemic alcohol

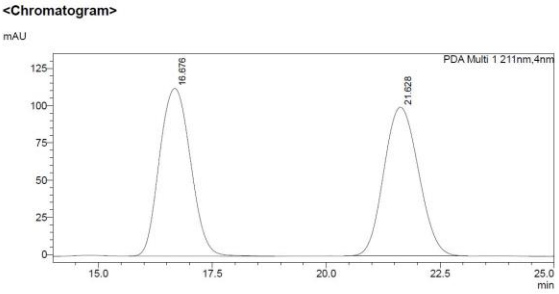

<Peak Table>

PDA Ch1 211nm

Peak#	Ret. Time	Area%
1	16.676	50.181
2	21.628	49.819
Total		100.000

Enantioenriched alcohol

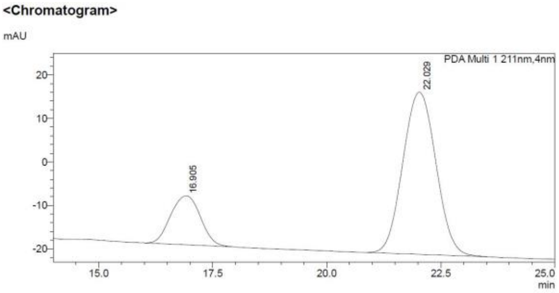

<Peak Table>

PDA Ch1 211nm

Peak#	Ret. Time	Area%
1	16.905	21.255
2	22.029	78.745
Total		100.000

(*S*)-Benzyl 2-cyclopentyl-2-(isobutyryloxy)propanoate (*R*)-S53:
HPLC conditions could not be identified for separation of the ester, therefore conversion calculated by ^1H NMR analysis of crude reaction product mixture.

Table 4: KR of benzyl 2-cyclohexyl-2-hydroxypropanoate 31

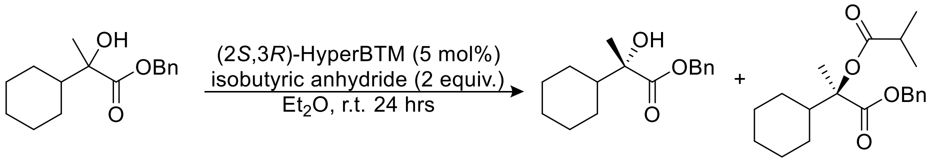

Following general procedure H, **benzyl 2-cyclohexyl-2-hydroxypropanoate 31** (42 mg, 0.16 mmol), isobutyric anhydride (53 µL, 0.32 mmol) and (2*S*,3*R*)-HyperBTM (2.5 mg, 0.008 mmol, 5 mol%) in Et_2O (0.5 mL) for 24 h gave, after column chromatography (Isolera 4, Et_2O in petrol, 0% to 10% over 25 CV), alcohol (28 mg, 0.11 mmol, 67%) and ester (12 mg, 0.07 mmol, 22%). **c = 22%, *s* = 32**

(*S*)-Benzyl 2-cyclohexyl-2-hydroxypropanoate (*S*)-31: $[\alpha]_D^{20}$ +2 (*c* 0.3, $CHCl_3$); Chiral HPLC Chiralpak AD-H (1% *i*PrOH:hexane, flow rate 1 mL min^{-1}, 211 nm, 30 °C) T_R: 14.9, 17.0 min, 63.1:36.9 er.*

Racemic alcohol

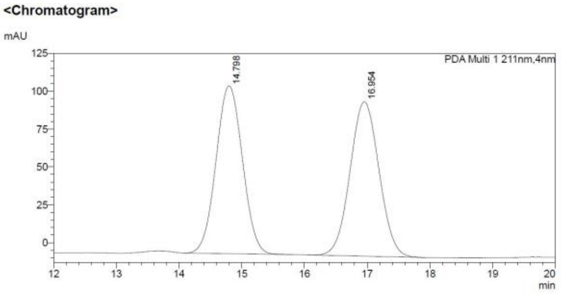

<Peak Table>

PDA Ch1 211nm

Peak#	Ret. Time	Area%
1	14.798	49.764
2	16.954	50.236
Total		100.000

Enantioenriched alcohol

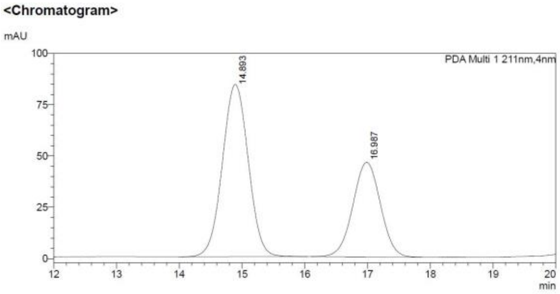

<Peak Table>

PDA Ch1 211nm

Peak#	Ret. Time	Area%
1	14.893	63.111
2	16.987	36.889
Total		100.000

*Lit:[31] (*R*)-isomer: Chiralpak AD, 5% *i*PrOH:hexane, flow rate 1 mL min^{-1}, T_R: 14.9 (minor), 17.0 min (major).
Absolute configuration determined to be (*S*)-isomer.

(*R*)-Benzyl 2-cyclohexyl-2-(isobutyryloxy)propanoate (*R*)-S54: $[\alpha]_D^{20}$ −3 (*c* 0.1, $CHCl_3$); Chiral HPLC Chiralpak AD-H (1% *i*PrOH:hexane, flow rate 1 mL min^{-1}, 211 nm, 30 °C) T_R: 7.8, 9.7 min, 96.1:3.9 er.

Racemic ester

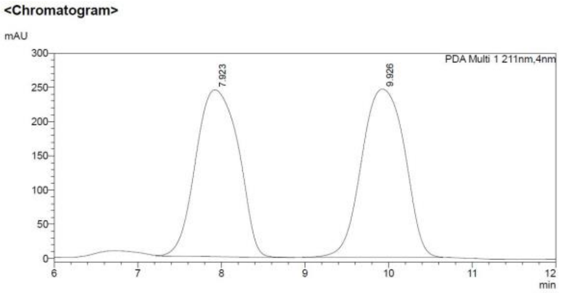

<Peak Table>

PDA Ch1 211nm

Peak#	Ret. Time	Area%
1	7.923	49.598
2	9.926	50.402
Total		100.000

Enantioenriched ester

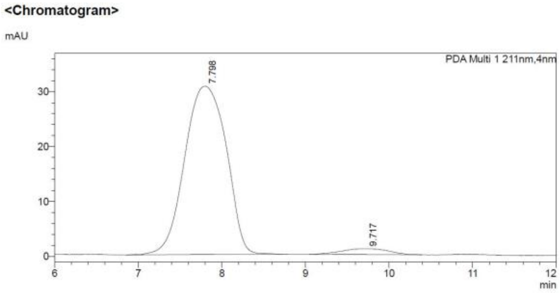

<Peak Table>

PDA Ch1 211nm

Peak#	Ret. Time	Area%
1	7.798	96.138
2	9.717	3.862
Total		100.000

Table 4: KR of benzyl 2-cyclohexyl-2-hydroxypropanoate 31

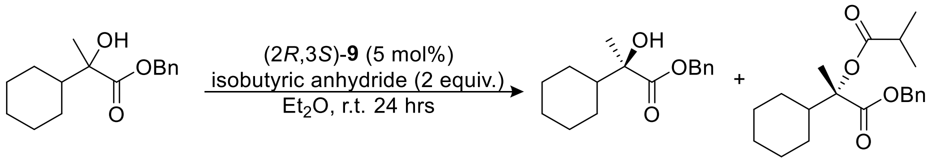

Following general procedure H, **benzyl 2-cyclohexyl-2-hydroxypropanoate 31** (42.0 mg, 0.16 mmol), isobutyric anhydride (53 µL, 0.32 mmol) and (2*R*,3*S*)-**9** (2.8 mg, 0.008 mmol, 5 mol%) in Et_2O (0.5 mL) for 24 h gave, after column chromatography (Isolera 4, Et_2O in petrol, 0% to 10% over 25 CV), alcohol (18.5 mg, 0.07 mmol, 44%) and ester (24.0 mg, 0.07 mmol, 45%).
c = 47%, *s* = 19

(*R*)-Benzyl 2-cyclohexyl-2-hydroxypropanoate (*S*)-31: Chiral HPLC Chiralpak AD-H (1% *i*PrOH:hexane, flow rate 1 mL min^{-1}, 211 nm, 30 °C) T_R: 14.9, 17.0 min, 14.9:85.1 er.

Racemic alcohol

<Chromatogram>

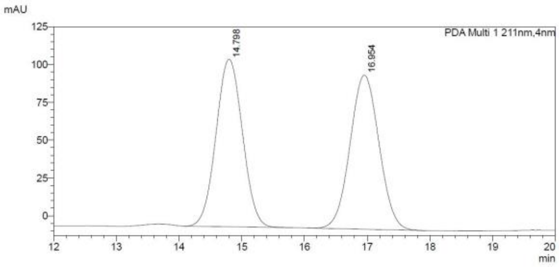

<Peak Table>

PDA Ch1 211nm

Peak#	Ret. Time	Area%
1	14.798	49.764
2	16.954	50.236
Total		100.000

Enantioenriched alcohol

<Chromatogram>

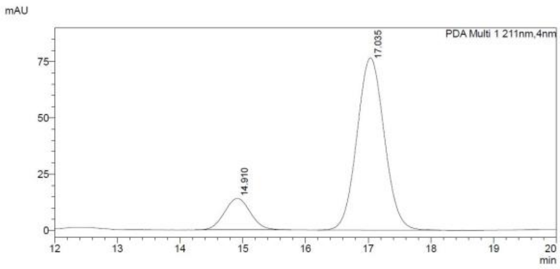

<Peak Table>

PDA Ch1 211nm

Peak#	Ret. Time	Area%
1	14.910	14.868
2	17.035	85.132
Total		100.000

(*S*)-Benzyl 2-cyclohexyl-2-(isobutyryloxy)propanoate (*R*)-S54: Chiral HPLC Chiralpak AD-H (1% *i*PrOH:hexane, flow rate 1 mL min^{-1}, 211 nm, 30 °C) T_R: 7.8, 9.7 min, 10.1:89.9 er.

Racemic ester

<Chromatogram>

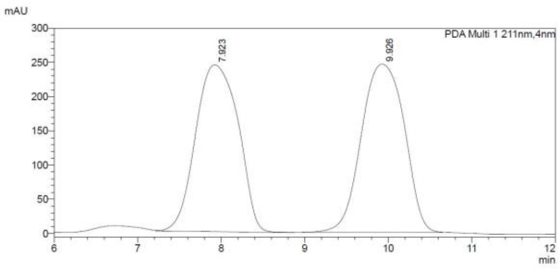

<Peak Table>

PDA Ch1 211nm

Peak#	Ret. Time	Area%
1	7.923	49.598
2	9.926	50.402
Total		100.000

Enantioenriched ester

<Chromatogram>

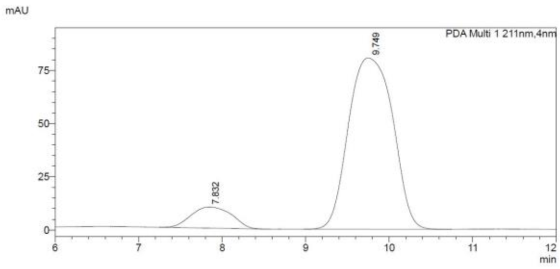

<Peak Table>

PDA Ch1 211nm

Peak#	Ret. Time	Area%
1	7.832	10.076
2	9.749	89.924
Total		100.000

KR of benzyl 2-hydroxy-2-(4-methoxyphenyl)-2-phenylacetate S16

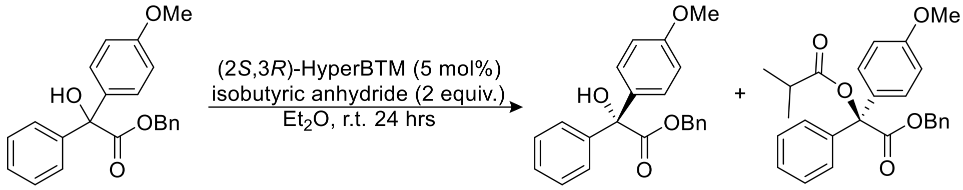

Following general procedure H, **benzyl 2-hydroxy-2-(4-methoxyphenyl)-2-phenylacetate S16** (112 mg, 0.32 mmol), isobutyric anhydride (106 µL, 0.64 mmol) and (2*S*,3*R*)-HyperBTM (5 mg, 0.016 mmol, 5 mol%) in Et_2O (1 mL) for 24 h gave, after column chromatography (Isolera 4, Et_2O in petrol, 0% to 15% over 30 CV), alcohol (90 mg, 0.26 mmol, 81%) and ester (14 mg, 0.03 mmol, 10%). **c = 16%, *s* = 1.4**

(*S*)-Benzyl 2-hydroxy-2-(4-methoxyphenyl)-2-phenylacetate (*S*)-S16: $[\alpha]_D^{20}$ +0.2 (*c* 1.8, $CHCl_3$) {Lit:[30] $[\alpha]_D^{22}$ +0.48 (c 3.5 in $CHCl_3$), 86% ee}; Chiral HPLC Chiralcel OJ-H (10% *i*PrOH:hexane, flow rate 1 mL min^{-1}, 211 nm, 30 °C) T_R: 74.2, 83.7 min, 51.3:48.7 er.

Racemic alcohol

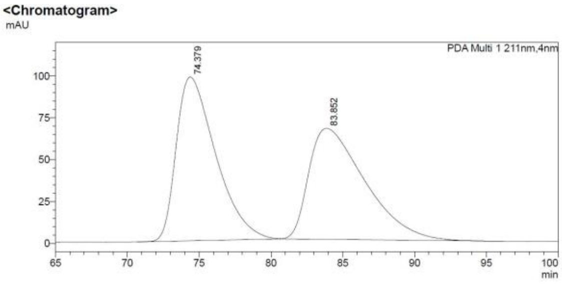

<Peak Table>

PDA Ch1 211nm

Peak#	Ret. Time	Area%
1	74.379	50.863
2	83.852	49.137
Total		100.000

Enantioenriched alcohol

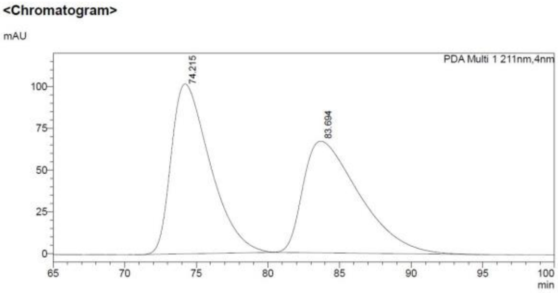

<Peak Table>

PDA Ch1 211nm

Peak#	Ret. Time	Area%
1	74.215	51.309
2	83.694	48.691
Total		100.000

(*R*)-2-(Benzyloxy)-1-(4-methoxyphenyl)-2-oxo-1-phenylethyl isobutyrate (*R*)-S55: $[\alpha]_D^{20}$ −1.4 (*c* 0.3, $CHCl_3$); Chiral HPLC Chiralpak AD-H (1% *i*PrOH:hexane, flow rate 1.0 mL min^{-1}, 211 nm, 30 °C) T_R: 40.5, 46.6 min, 56.9:43.1 er.

Racemic ester

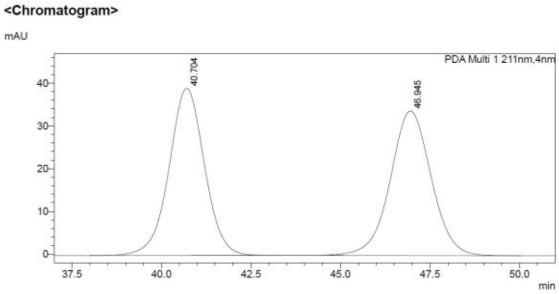

<Peak Table>

PDA Ch1 211nm

Peak#	Ret. Time	Area%
1	40.704	50.028
2	46.945	49.972
Total		100.000

Enantioenriched ester

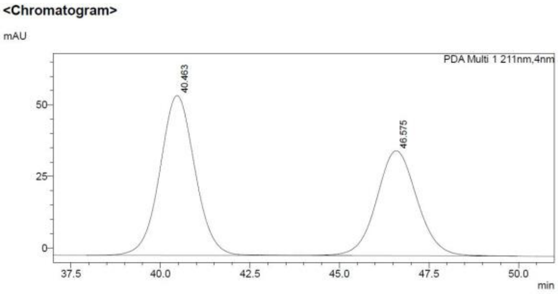

<Peak Table>

PDA Ch1 211nm

Peak#	Ret. Time	Area%
1	40.463	56.868
2	46.575	43.132
Total		100.000

KR of benzyl 2-hydroxy-2-(4-methoxyphenyl)-2-phenylacetate S16

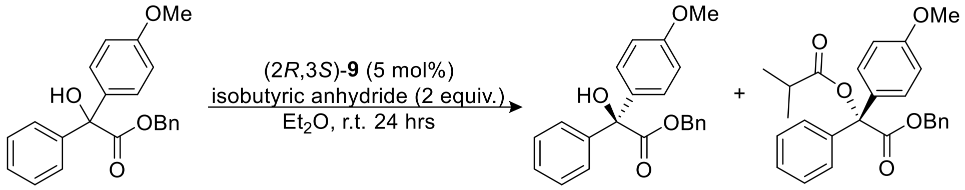

Following general procedure H, **benzyl 2-hydroxy-2-(4-methoxyphenyl)-2-phenylacetate S16** (112 mg, 0.32 mmol), isobutyric anhydride (106 µL, 0.64 mmol) and (2*R*,3*S*)-**9** (5.7 mg, 0.016 mmol, 5 mol%) in Et_2O (1 mL) for 24 h gave, after column chromatography (Isolera 4, Et_2O in petrol, 0% to 10% over 25 CV), alcohol (31 mg, 0.09 mmol, 28%) and ester (87 mg, 0.21 mmol, 65%). **c = 69%, *s* = 1.3**

(*R*)-Benzyl 2-hydroxy-2-(4-methoxyphenyl)-2-phenylacetate (*R*)-S16: $[\alpha]_D^{20}$ −0.7 (*c* 0.6, $CHCl_3$) {Lit:[30] $[\alpha]_D^{24}$ −0.44 (c 3.4 in $CHCl_3$), 83% ee}; Chiral HPLC Chiralcel OJ-H (10% *i*PrOH:hexane, flow rate 1 mL min^{-1}, 211 nm, 30 °C) T_R: 73.1, 83.6 min, 41.5:58.5 er;

Racemic alcohol

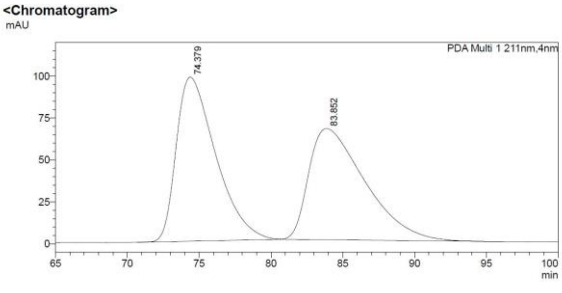

<Peak Table>

PDA Ch1 211nm

Peak#	Ret. Time	Area%
1	74.379	50.863
2	83.852	49.137
Total		100.000

Enantioenriched alcohol

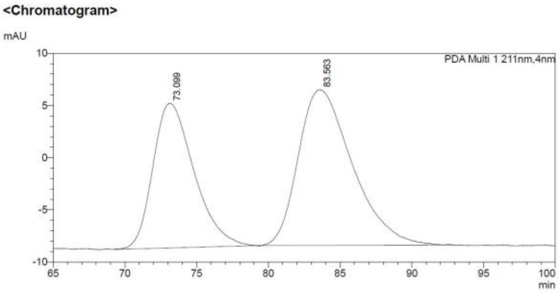

<Peak Table>

PDA Ch1 211nm

Peak#	Ret. Time	Area%
1	73.099	41.460
2	83.563	58.540
Total		100.000

(*S*)-2-(Benzyloxy)-1-(4-methoxyphenyl)-2-oxo-1-phenylethyl isobutyrate (*S*)-S55: $[\alpha]_D^{20}$ +0.9 (*c* 1.7, $CHCl_3$); Chiral HPLC Chiralpak AD-H (1% *i*PrOH:hexane, flow rate 1.0 mL min^{-1}, 211 nm, 30 °C) T_R: 40.2, 46.3 min, 46.2:54.8 er.

Racemic ester

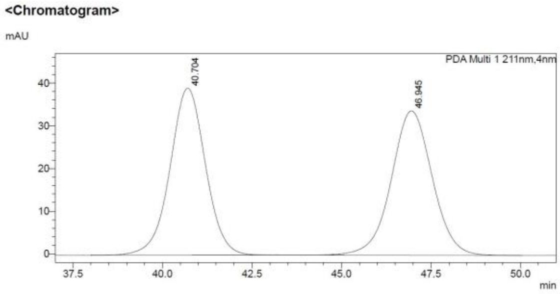

<Peak Table>

PDA Ch1 211nm

Peak#	Ret. Time	Area%
1	40.704	50.028
2	46.945	49.972
Total		100.000

Enantioenriched ester

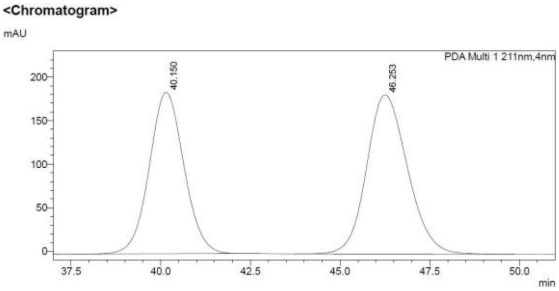

<Peak Table>

PDA Ch1 211nm

Peak#	Ret. Time	Area%
1	40.150	46.238
2	46.253	53.762
Total		100.000

Figure 3a: Kinetic resolution of 1-(benzyloxy)-2-phenylpropan-2-ol 33

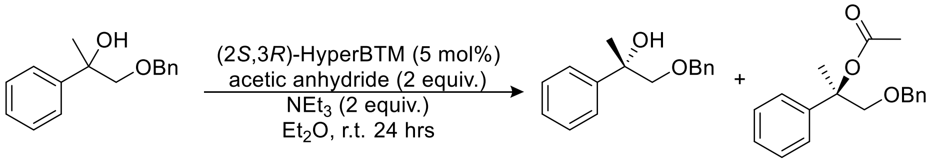

Following general procedure H, **1-(benzyloxy)-2-phenylpropan-2-ol 33** (24 mg, 0.1 mmol), acetic anhydride (19 µL, 0.2 mmol), NEt_3 (28 µL, 0.2 mmol) and (2*S*,3*R*)-HyperBTM (1.6 mg, 0.005 mmol, 5 mol%) in Et_2O (0.3 mL) overnight gave, after column chromatography (Isolera 4, Et_2O in petrol, 0% to 20% over 30CV), alcohol (10 mg, 0.04 mmol, 41%) and ester (11 mg, 0.04 mmol, 38%). **c = 50%, *s* = 3**

(*S*)-1-(Benzyloxy)-2-phenylpropan-2-ol (*S*)-33: $[\alpha]_D^{20}$ −4.7 (*c* 0.1, $CHCl_3$) [lit[21] (*S*)-isomer: $[\alpha]_D^{27.5}$ –12.0 (c = 1.16, $CHCl_3$).]; Chiral HPLC Chiralpak AD-H (1% *i*PrOH:hexane, flow rate 0.5 mL min^{-1}, 211 nm, 30 °C) T_R: 33.0, 35.5 min, 68.7:31.3 er.*

Racemic alcohol

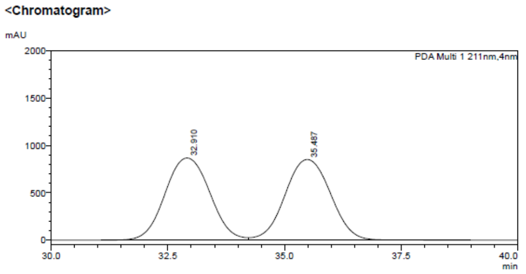

<Peak Table>

PDA Ch1 211nm

Peak#	Ret. Time	Area%
1	32.910	50.053
2	35.487	49.947
Total		100.000

Enantioenriched alcohol

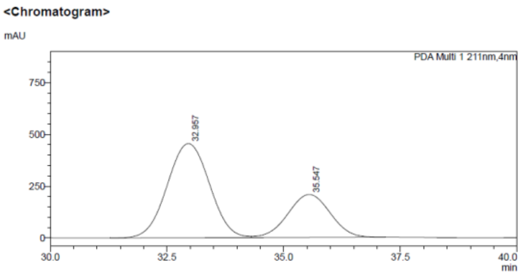

<Peak Table>

PDA Ch1 211nm

Peak#	Ret. Time	Area%
1	32.957	68.749
2	35.547	31.251
Total		100.000

(*R*)-1-(Benzyloxy)-2-phenylpropan-2-yl acetate (*R*)-S56: $[\alpha]_D^{20}$ +5.9 (*c* 0.1, $CHCl_3$); Chiral HPLC Chiralpak AD-H (1% *i*PrOH:hexane, flow rate 0.5 mL min^{-1}, 211 nm, 30 °C) T_R: 14.9, 17.7 min, 30.8:69.2 er.

Racemic ester

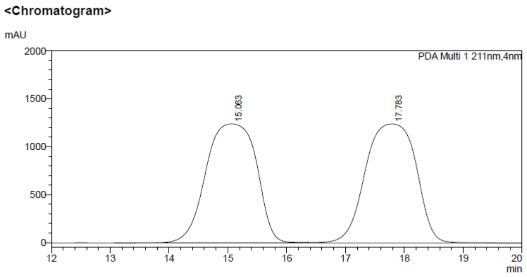

<Peak Table>

PDA Ch1 211nm

Peak#	Ret. Time	Area%
1	15.063	49.897
2	17.783	50.103
Total		100.000

Enantioenriched ester

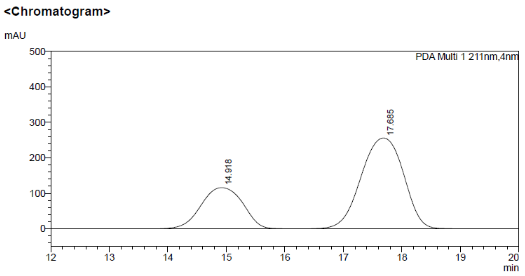

<Peak Table>

PDA Ch1 211nm

Peak#	Ret. Time	Area%
1	14.918	30.809
2	17.685	69.191
Total		100.000

Figure 3a: Kinetic resolution of benzyl 3-hydroxy-3-phenylbutanoate 34

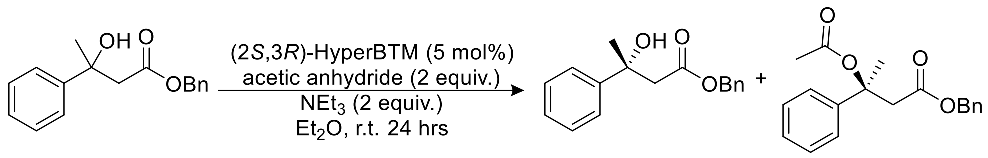

Absolute configuration of products not unambiguously confirmed

Following general procedure H, **benzyl 3-hydroxy-3-phenylbutanoate 34** (54 mg, 0.2 mmol), acetic anhydride (38 µL, 0.4 mmol), NEt_3 (56 µL, 0.4 mmol) and (2*S*,3*R*)-HyperBTM (3.1 mg, 0.01 mmol, 5 mol%) in Et_2O (0.6 mL) overnight gave, after column chromatography (Isolera 4, Et_2O in petrol, 0% to 20% over 30CV), alcohol (39 mg, 0.14 mmol, 72%) and ester (8 mg, 0.02 mmol, 11%). **c = 13%, *s* = 2**

Benzyl 3-hydroxy-3-phenylbutanoate 34: $[\alpha]_D^{20}$ +3.9 (*c* 0.1, $CHCl_3$); Chiral HPLC Chiralpak AD-H (1% *i*PrOH:hexane, flow rate 1.0 mL min^{-1}, 211 nm, 30 °C) T_R: 20.0, 23.4 min, 52.2:47.8 er.

Racemic alcohol

<Chromatogram>

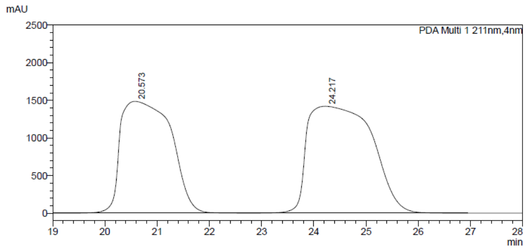

<Peak Table>

PDA Ch1 211nm

Peak#	Ret. Time	Area%
1	20.573	44.965
2	24.217	55.035
Total		100.000

Enantioenriched alcohol

<Chromatogram>

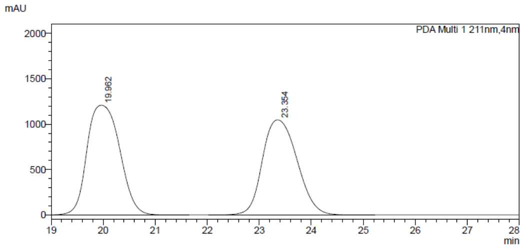

<Peak Table>

PDA Ch1 211nm

Peak#	Ret. Time	Area%
1	19.962	52.199
2	23.354	47.801
Total		100.000

Benzyl 3-acetoxy-3-phenylbutanoate S57: $[\alpha]_D^{20}$ -2.2 (*c* 0.3, $CHCl_3$); Chiral HPLC Chiralpak AD-H (1% *i*PrOH:hexane, flow rate 1.0 mL min^{-1}, 211 nm, 30 °C) T_R: 17.7, 19.8 min, 34.9:65.1 er.

Racemic ester

<Chromatogram>

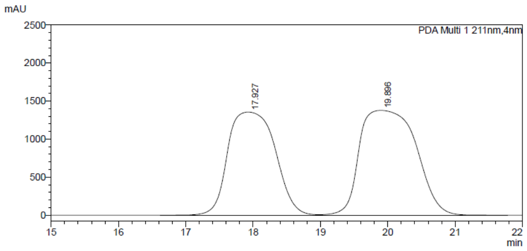

<Peak Table>

PDA Ch1 211nm

Peak#	Ret. Time	Area%
1	17.927	45.177
2	19.896	54.823
Total		100.000

Enantioenriched ester

<Chromatogram>

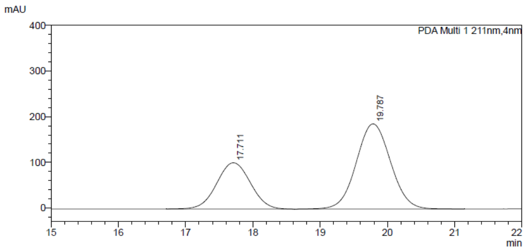

<Peak Table>

PDA Ch1 211nm

Peak#	Ret. Time	Area%
1	17.711	34.860
2	19.787	65.140
Total		100.000

Catalytic Kinetic Resolution in Continuous Flow: Optimisation

General procedure I: *Kinetic resolution of tertiary alcohols in continuous flow*

A packed bed reactor consisting of a vertically-mounted Omnifit glass chromatography column (10 mm pore size and up to maximal 70 mm of adjustable bed height), with a glass cooling jacket was loaded with PS-HyperBTM resin (1.2 g; *f* = 0.85 mmol g^{-1}). The resin was allowed to swell to its maximum volume by pumping solvent at 0.5 mL min^{-1} for 30 min at r.t. using a P 4.1S Azura pump developed by Knauer. The flow rate was then changed to the specified reaction flow rate for 30 min. A Legato 200 series syringe pump by KR Scientific was used to inject samples through syringes. The syringe was filled with a mixture of the appropriate alcohol (1 equiv.) and isobutyric anhydride in specified solvent. The solution was injected at specified flow rate. After addition of reagents is completed, a P 4.1S Azura pump developed by Knauer was connected and pumped solvent at specified flow rate for 120 min to ensure elution of the products and achieve regeneration of the column for the next reaction. The collected reaction mixture was concentrated *in vacuo*. The alcohol and ester were purified by column chromatography and analysed by chiral HPLC.

Table S11: Solvent and base

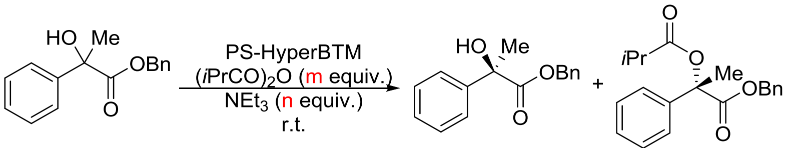

(±)-**8** (*S*)-**8** (*R*)-**10**

Entry	solvent	mass of PS-HyperBTM	m	n	c	*s*
1*	Toluene	1.2 g	3	3	<5	-
2*	Toluene	1.5 g	3	3	15	90
3*	$CHCl_3$	1.5 g	3	3	trace	-
4*	EtOAc	1.5 g	3	3	8	80
5*	Toluene	1.5 g	5	5	19	90
6**	Toluene	1.5 g	5	-	21	90

* 0.2 mmol scale, alcohol dissolved in 5 mL of solvent in one syringe, anhydride and base dissolved in 5 mL of solvent in the second syringe flow rate 0.05 mL min^{-1} (0.1 mL min^{-1} combined); ** alcohol and anhydride dissolved in 10 mL of solvent in one syringe, flow rate 1 mL min^{-1}

Table S12: Concentration

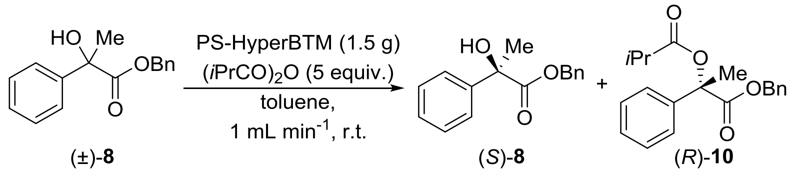

Entry	Solvent used to dissolve alcohol and anhydride/ mL	c	*s*
1	10	21	90
2	20	9	80
3	5	26	90
4	2	33	80
5	1	36	70

Table S13: Flow rate and looping

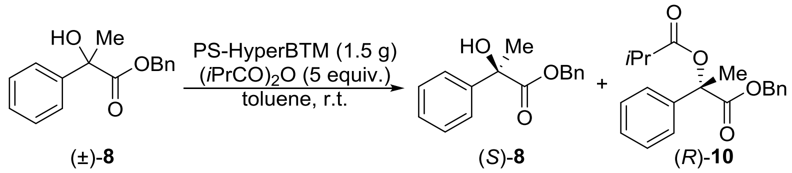

Entry	solvent / mL	Flow rate/ mL min^{-1}	loops	c	*s*
1	2	0.1	1	33	88
2[a]	2	0.1	1	32	77
3	5	0.1	1	26	85
4	5	0.08	1	26	90
5	5	0.05	1	32	88
6[b]	5	0.1	2	32	84
7[b,c]	5	0.1	2	38	87

a) PS-cat washed by 10% MeOH in $CHCl_3$ before solvent flush and KR; b) after injection of sample is finished and toluene pumped through column for 90 mins, the collection was concentrated *in vacuo*, re-dissolved in 5 mL of toluene and subjected to another loop; c) 5 equiv. of anhydride added in the second loop

Table S14: Catalyst loading

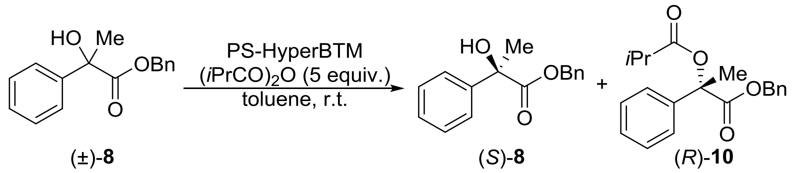

Entry	cat / g	solvent	Flow rate/ mL min^{-1}	c	*s*
1	1.5	2 mL	0.1	33	80
2	2.5	5 mL	0.1	30	87
3	2.5	2 mL	0.05	37	72
4[a]	2.5	1 mL	0.05	50	52

a) 10 equiv. of anhydride used

Optimal condition: 2.5 g PS-HyperBTM, 5 equiv. of isobutyric anhydride, injection sample dissolved in 1 mL of toluene, flow rate 0.05 mL min^{-1} of toluene, room temperature

Catalytic Kinetic Resolution in Continuous Flow: Scope

Table 5 KR of benzyl 2-hydroxy-2-phenypropanoate 8

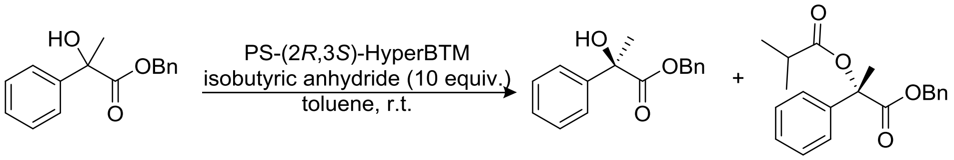

Following general procedure I, **benzyl 2-hydroxy-2-phenypropanoate 8** (51 mg, 0.2 mmol), isobutyric anhydride (0.33 mL, 2.0 mmol) and PS-HyperBTM in toluene (1 mL) gave, after column chromatography (Isolera 4, Et_2O in petrol, 0% to 15% over 30 CV), alcohol (23 mg, 0.09 mmol, 44%) and ester (31 mg, 0.09 mmol, 47%). **c = 50%, *s* = 50**

(*R*)-Benzyl 2-hydroxy-2-phenypropanoate (*R*)-8: Chiral HPLC Chiralpak AD-H (1% *i*PrOH:hexane, flow rate 1.0 mL min^{-1}, 211 nm, 30 °C) T_R: 25.8, 34.2 min, 4.9:95.1 er.

Racemic alcohol

<Chromatogram>

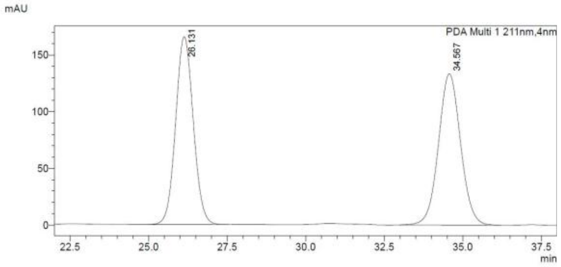

<Peak Table>

PDA Ch1 211nm

Peak#	Ret. Time	Area%
1	26.131	49.857
2	34.567	50.143
Total		100.000

Enantioenriched alcohol

<Chromatogram>

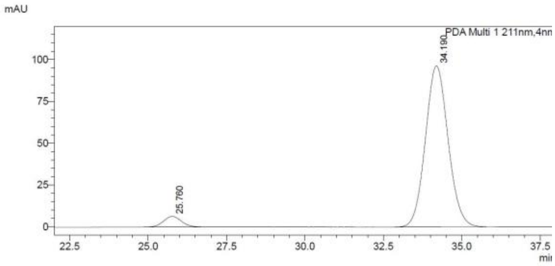

<Peak Table>

PDA Ch1 211nm

Peak#	Ret. Time	Area%
1	25.760	4.876
2	34.190	95.124
Total		100.000

(*S*)-Benzyl 2-(isobutyryloxy)-2-phenylpropanoate (*S*)-10: Chiral HPLC Chiralpak AD-H (1% *i*PrOH:hexane, flow rate 1.0 mL min^{-1}, 211 nm, 30 °C) T_R: 11.3, 17.0 min, 5.6:94.4 er.

Racemic ester

<Chromatogram>

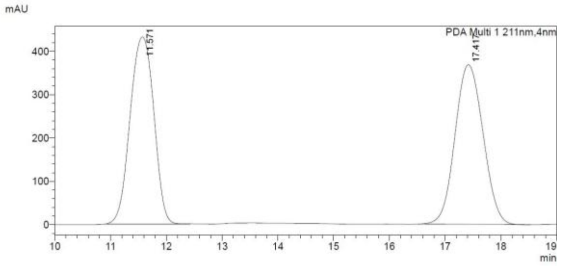

<Peak Table>

PDA Ch1 211nm

Peak#	Ret. Time	Area%
1	11.571	49.744
2	17.417	50.256
Total		100.000

Enantioenriched ester

<Chromatogram>

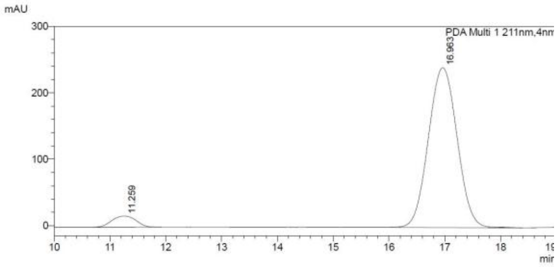

<Peak Table>

PDA Ch1 211nm

Peak#	Ret. Time	Area%
1	11.259	5.583
2	16.963	94.417
Total		100.000

Table 5 KR of benzyl 2-(4-(*tert*-butyl)phenyl)-2-hydroxypropanoate 13

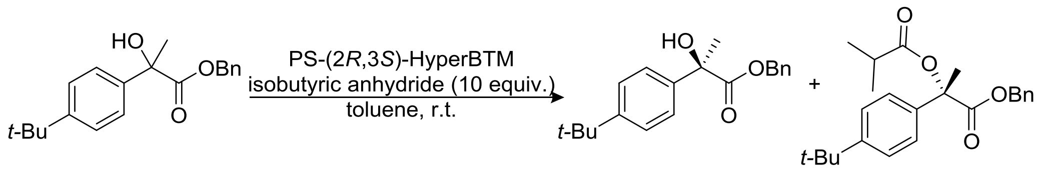

Following general procedure I, **benzyl 2-(4-(*tert*-butyl)phenyl)-2-hydroxypropanoate 13** (62 mg, 0.22 mmol), isobutyric anhydride (0.33 mL, 2.0 mmol) and PS-HyperBTM in toluene (1 mL) gave, after column chromatography (Isolera 4, Et_2O in petrol, 0% to 15% over 30 CV), alcohol (28 mg, 0.09 mmol, 44%) and ester (31 mg, 0.08 mmol, 41%). **c = 50%, *s* = 80**

(*R*)-Benzyl 2-(4-(*tert*-butyl)phenyl)-2-hydroxypropanoate (*R*)-13: Chiral HPLC Chiralcel OJ-H (1% *i*PrOH:hexane, flow rate 1.0 mL min^{-1}, 211 nm, 30 °C) T_R: 25.1, 41.8 min, 4.7:95.3 er.

Racemic alcohol

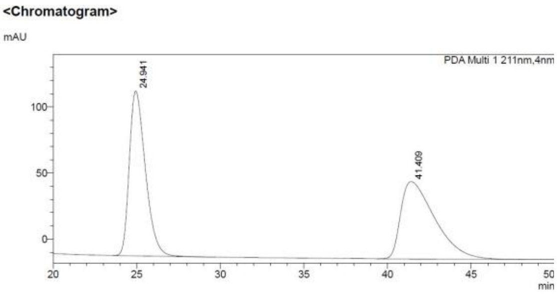

<Peak Table>

PDA Ch1 211nm

Peak#	Ret. Time	Area%
1	24.941	50.099
2	41.409	49.901
Total		100.000

Enantioenriched alcohol

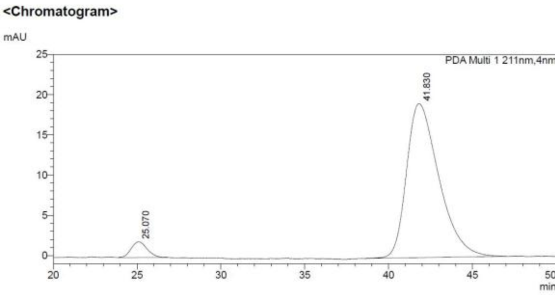

<Peak Table>

PDA Ch1 211nm

Peak#	Ret. Time	Area%
1	25.070	4.745
2	41.830	95.255
Total		100.000

(*S*)-Benzyl 2-(4-(*tert*-butyl)phenyl)-2-(isobutyryloxy)propanoate (*S*)-S33: Chiral HPLC Chiralpak AD-H (1% *i*PrOH:hexane, flow rate 0.5 mL min^{-1}, 211 nm, 30 °C) T_R: 14.3, 16.9 min, 4.0:96.0 er.

Racemic ester

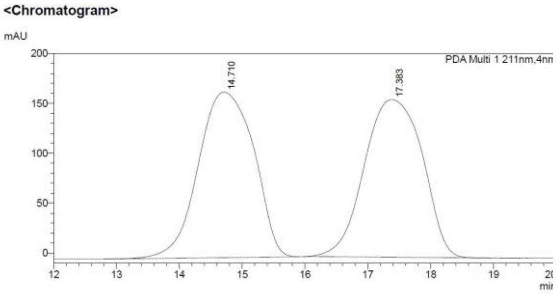

<Peak Table>

PDA Ch1 211nm

Peak#	Ret. Time	Area%
1	14.710	50.085
2	17.383	49.915
Total		100.000

Enantioenriched ester

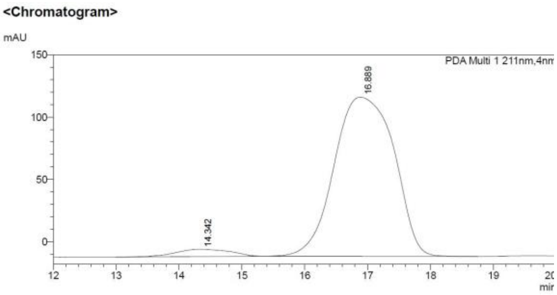

<Peak Table>

PDA Ch1 211nm

Peak#	Ret. Time	Area%
1	14.342	3.979
2	16.889	96.021
Total		100.000

Table 5 KR of benzyl 2-hydroxy-2-(3-methoxyphenyl)propanoate 15

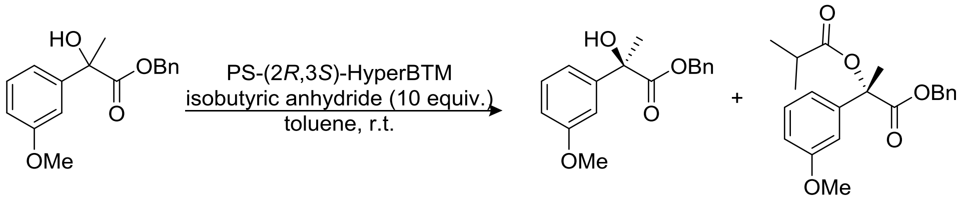

Following general procedure I, **benzyl 2-hydroxy-2-(3-methoxyphenyl)propanoate 15** (57 mg, 0.2 mmol), isobutyric anhydride (0.33 mL, 2.0 mmol) and PS-HyperBTM in toluene (1 mL) for 24 h gave, after column chromatography (Isolera 4, Et_2O in petrol, 0% to 15% over 25 CV), alcohol (25 mg, 0.09 mmol, 44%) and ester (29 mg, 0.08 mmol, 40%).
c = 47%, *s* = 29

(*R*)-Benzyl 2-(isobutyryloxy)-2-(3-methoxyphenyl)propanoate (*R*)-15: Chiral HPLC Chiralpak AD-H (1% *i*PrOH:hexane, flow rate 1 mL min^{-1}, 211 nm, 30 °C) T_R: 48.2, 64.7 min, 11.6:88.4 er.

Racemic alcohol

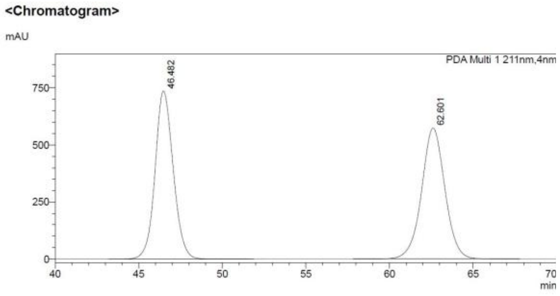

<Peak Table>

PDA Ch1 211nm

Peak#	Ret. Time	Area%
1	46.482	49.940
2	62.601	50.060
Total		100.000

Enantioenriched alcohol

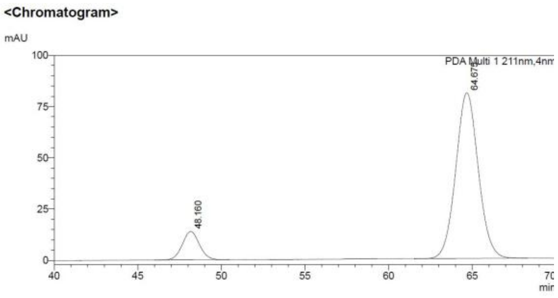

<Peak Table>

PDA Ch1 211nm

Peak#	Ret. Time	Area%
1	48.160	11.573
2	64.675	88.427
Total		100.000

(*S*)-Benzyl 2-(4-(*tert*-butyl)phenyl)-2-(isobutyryloxy)propanoate (*S*)-S35: Chiral HPLC Chiralpak AD-H (1% *i*PrOH:hexane, flow rate 1.0 mL min^{-1}, 211 nm, 30 °C) T_R: 16.8, 28.3 min, 7.4:92.6 er.

Racemic ester

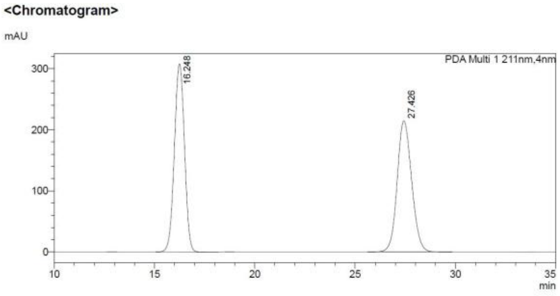

<Peak Table>

PDA Ch1 211nm

Peak#	Ret. Time	Area%
1	16.248	50.019
2	27.426	49.981
Total		100.000

Enantioenriched ester

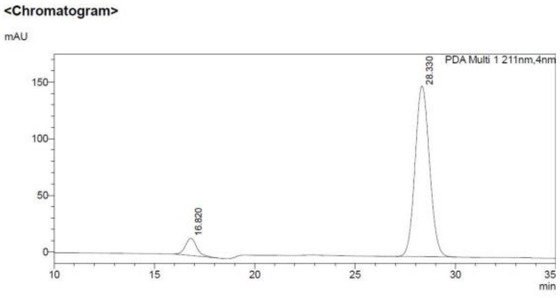

<Peak Table>

PDA Ch1 211nm

Peak#	Ret. Time	Area%
1	16.820	7.369
2	28.330	92.631
Total		100.000

Table 5 KR benzyl 2-hydroxy-2-phenylbut-3-enoate 24

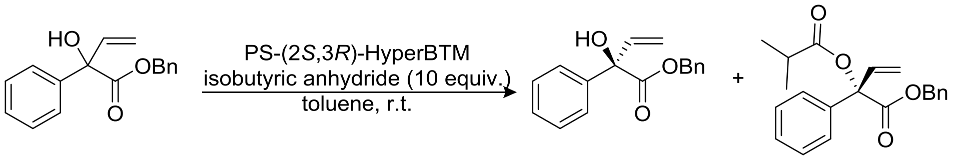

Following general procedure I, **benzyl 2-hydroxy-2-phenylbut-3-enoate 24** (54 mg, 0.2 mmol), isobutyric anhydride (0.33 mL, 2.0 mmol) and PS-HyperBTM in toluene (1 mL) gave, after column chromatography (eluent Petrol/Et_2O, 9:1), alcohol (31 mg, 0.12 mmol, 60%) and ester (20 mg, 0.06 mmol, 30%). **c = 33%, *s* = 21**

(*R*)-Benzyl 2-hydroxy-2-phenylbut-3-enoate (*R*)-24: Chiral HPLC Chiralpak AD-H (1% *i*PrOH:hexane, flow rate 1.0 mL min^{-1}, 211 nm, 30 °C) T_R: 25.1, 36.0 min, 28.9:71.1 er.

Racemic alcohol

<Chromatogram>

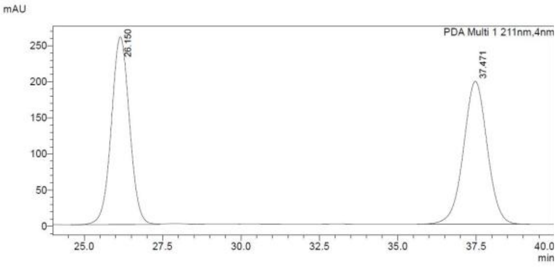

<Peak Table>

PDA Ch1 211nm

Peak#	Ret. Time	Area%
1	26.150	50.094
2	37.471	49.906
Total		100.000

Enantioenriched alcohol

<Chromatogram>

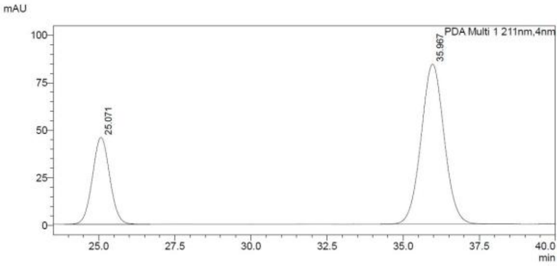

<Peak Table>

PDA Ch1 211nm

Peak#	Ret. Time	Area%
1	25.071	28.888
2	35.967	71.112
Total		100.000

(*S*)-Benzyl 2-(isobutyryloxy)-2-phenylbut-3-enoate (*S*)-S47: Chiral HPLC Chiralpak AD-H (1% *i*PrOH:hexane, flow rate 1.0 mL min^{-1}, 211 nm, 30 °C) T_R: 13.5, 19.3 min, 93.4:6.6 er.

Racemic ester

<Chromatogram>

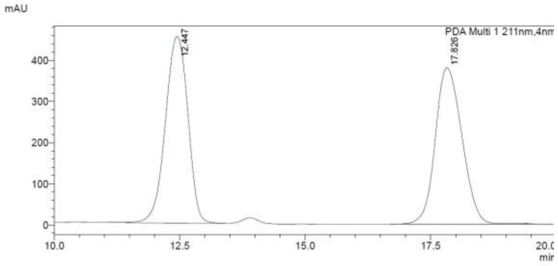

<Peak Table>

PDA Ch1 211nm

Peak#	Ret. Time	Area%
1	12.447	50.102
2	17.826	49.898
Total		100.000

Enantioenriched ester

<Chromatogram>

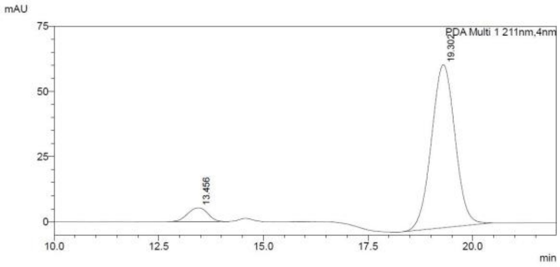

<Peak Table>

PDA Ch1 211nm

Peak#	Ret. Time	Area%
1	13.456	6.647
2	19.302	93.353
Total		100.000

Table 5 KR of benzyl 2-hydroxy-2,3-dimethylbut-3-enoate 29

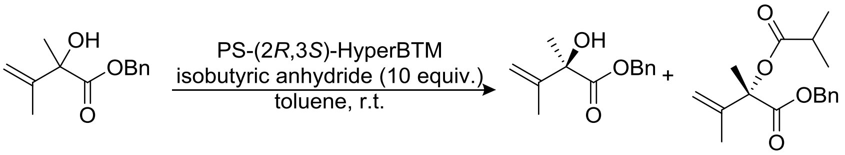

Following general procedure I, **benzyl 2-hydroxy-2,3-dimethylbut-3-enoate 29** (44 mg, 0.2 mmol), isobutyric anhydride (0.33 mL, 2.0 mmol) and PS-HyperBTM in toluene (1 mL) gave, after column chromatography (Isolera 4, Et_2O in petrol, 0% to 10% over 30 CV), alcohol (25 mg, 0.12 mmol, 58%) and ester (20 mg, 0.07 mmol, 34%). **c = 40%, *s* = 60**

(*R*)-Benzyl 2-hydroxy-2,3-dimethylbut-3-enoate (*R*)-29: Chiral HPLC Chiralcel OJ-H (1% *i*PrOH:hexane, flow rate 0.5 mL min^{-1}, 211 nm, 30 °C) T_R: 19.7, 25.1 min, 18.7:81.3 er.

Racemic alcohol

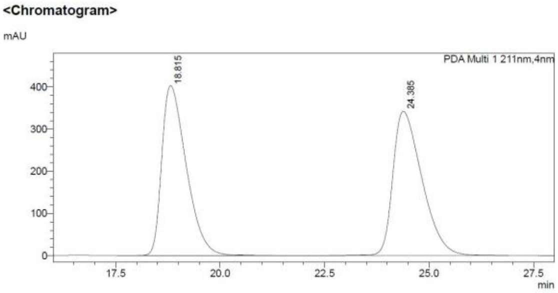

<Peak Table>

PDA Ch1 211nm

Peak#	Ret. Time	Area%
1	18.815	49.822
2	24.385	50.178
Total		100.000

Enantioenriched alcohol

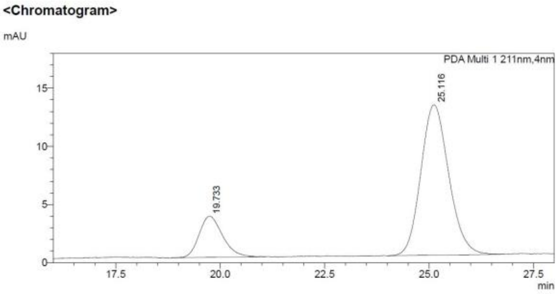

<Peak Table>

PDA Ch1 211nm

Peak#	Ret. Time	Area%
1	19.733	18.679
2	25.116	81.321
Total		100.000

The benzyl ester of **(*R*)-29** was hydrolyzed using NaOH (2 M):THF (1:1) at 85 °C for 24 hrs, aqueous work-up yielded (*R*)-2-hydroxy-2,3-dimethylbut-3-enoic acid $[\alpha]_D^{20}$ −4.4 (*c* 0.1, ethanol), lit[33] $[\alpha]_D^{20}$ −18.2 (*c* 1.05, ethanol);
Absolute configuration determined to be (*R*)-isomer.

(*S*)-Benzyl 2-(isobutyryloxy)-2,3-dimethylbut-3-enoate (*S*)-S52: Chiral HPLC Chiralpak AD-H (0.5% *i*PrOH:hexane, flow rate 0.5 mL min^{-1}, 211 nm, 30 °C) T_R: 18.4, 23.5 min, 2.9:97.1 er.

Racemic ester

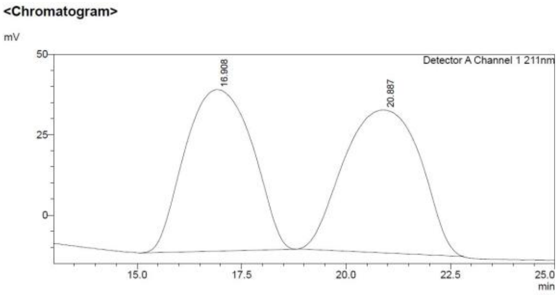

<Peak Table>

Detector A Channel 1 211nm

Peak#	Ret. Time	Area%
1	16.908	49.761
2	20.887	50.239
Total		100.000

Enantioenriched ester

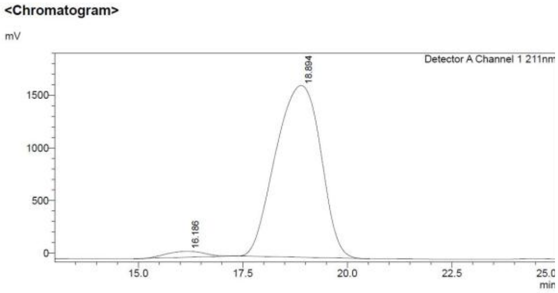

<Peak Table>

Detector A Channel 1 211nm

Peak#	Ret. Time	Area%
1	16.186	2.904
2	18.894	97.096
Total		100.000

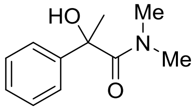

2: ^1H, $CDCl_3$, 500 MHz

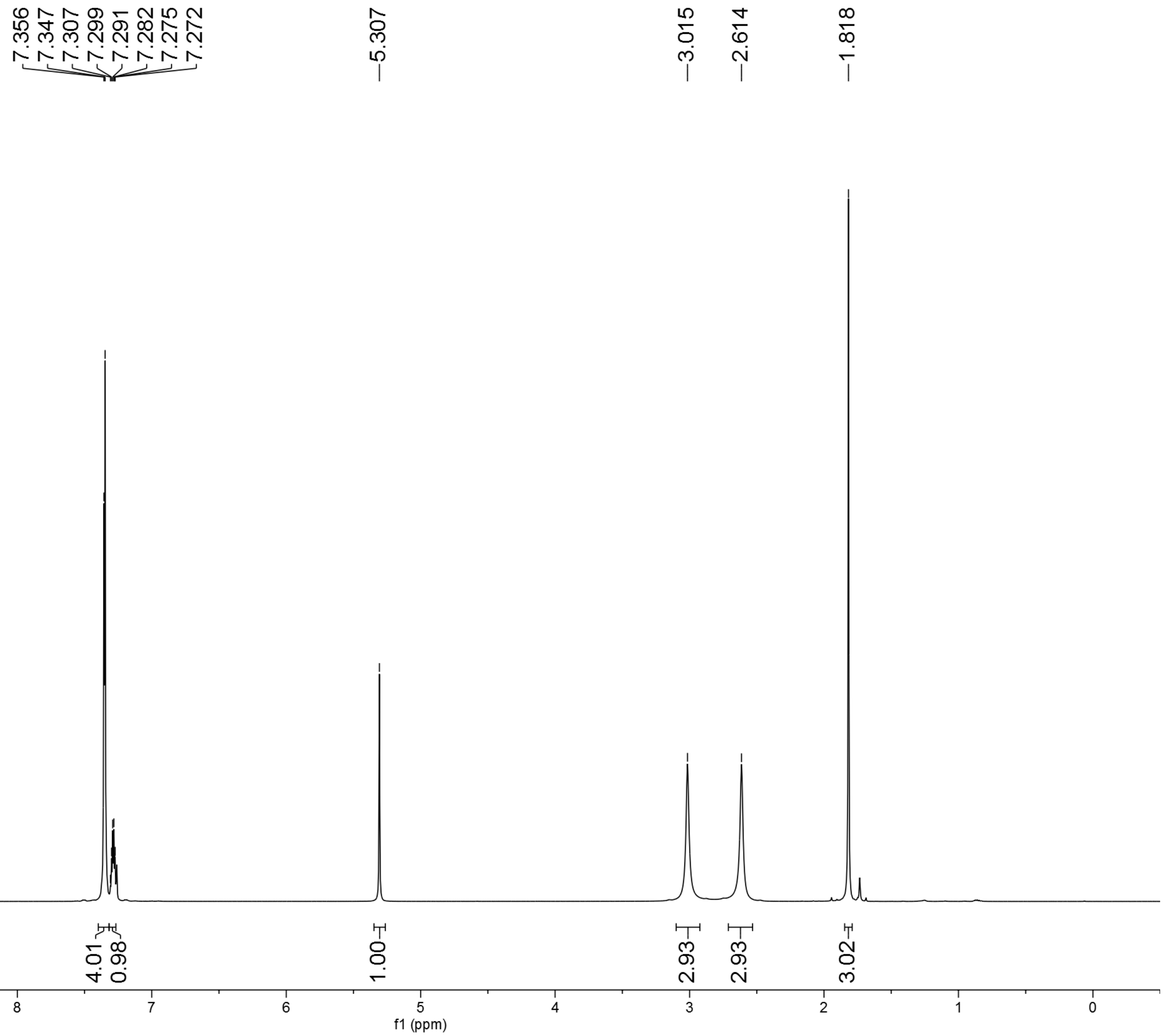

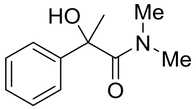

2: ^{13}C, $CDCl_3$, 126 MHz

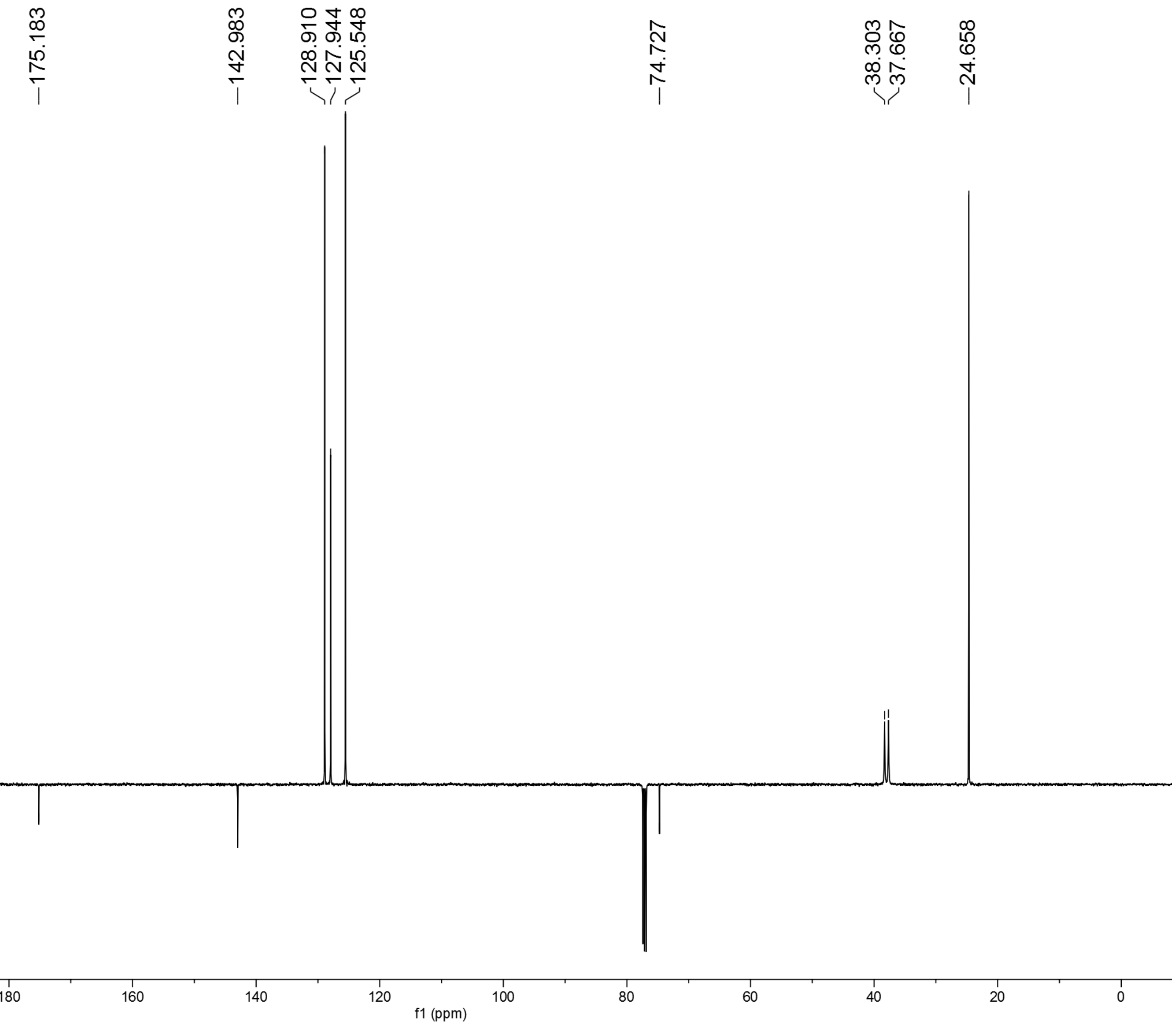

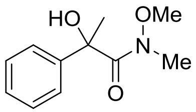

S2: ^{1}H, $CDCl_3$, 500 MHz

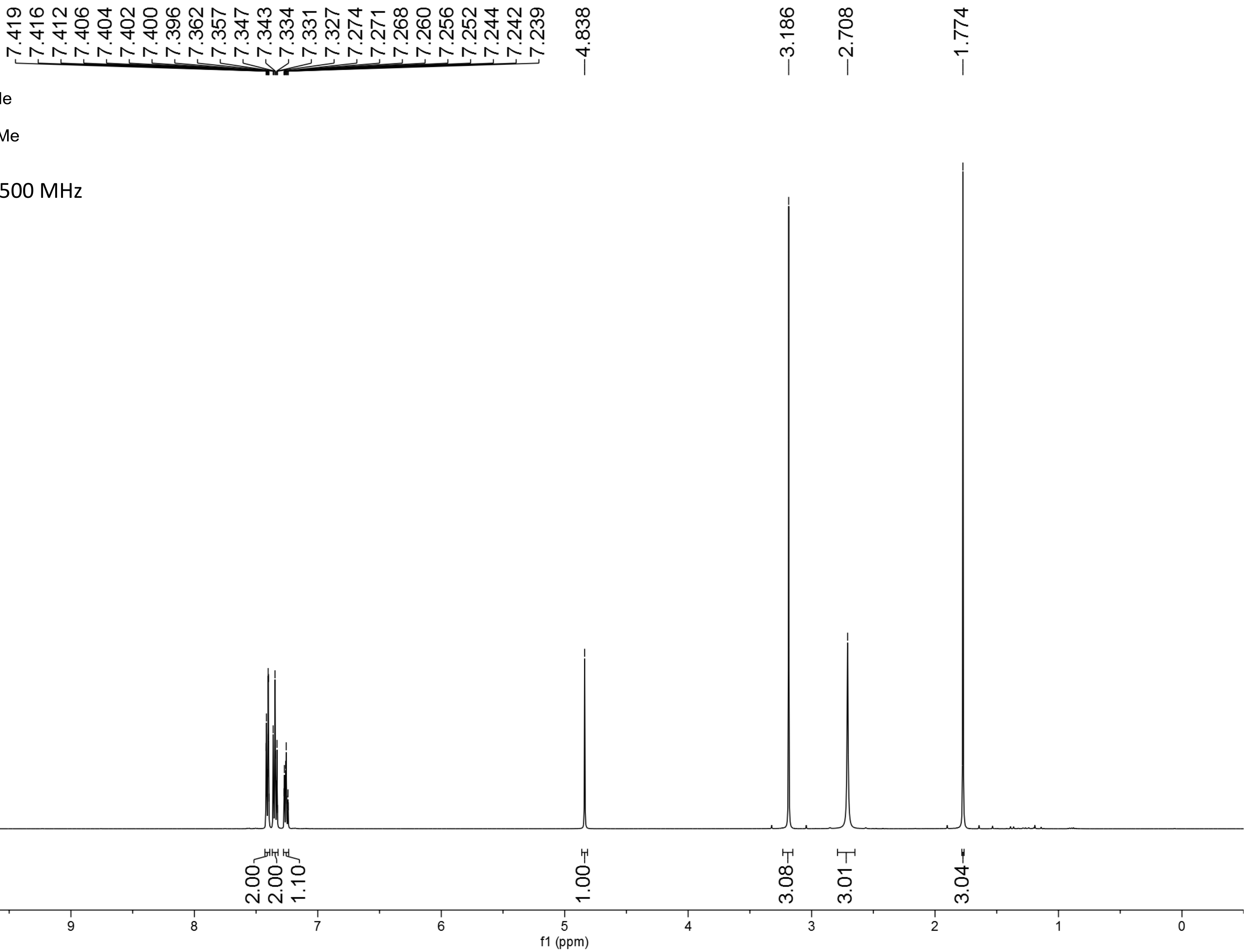

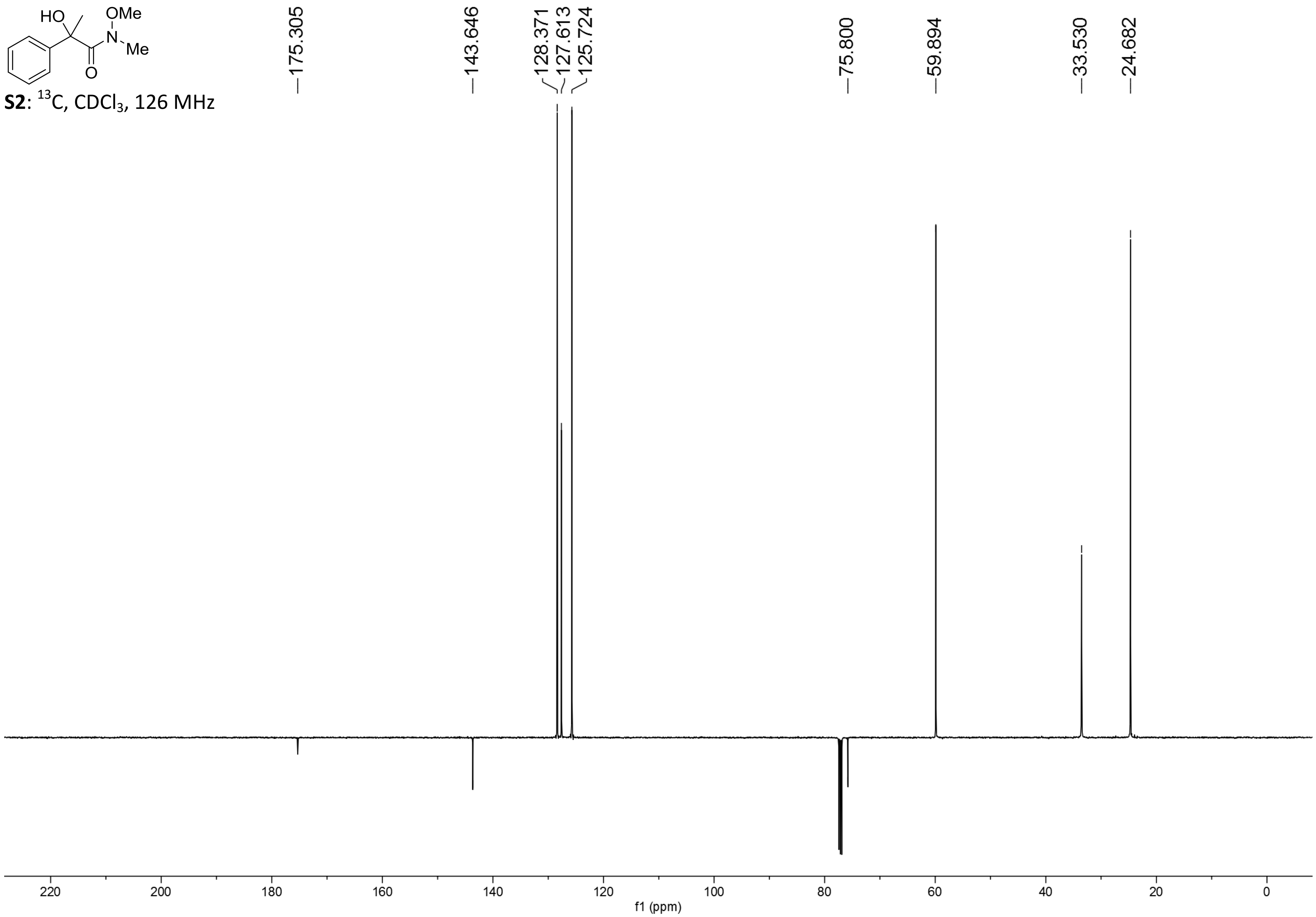

S2: ^{13}C, $CDCl_3$, 126 MHz

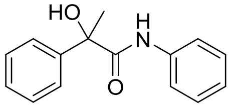

3: ^{1}H, $CDCl_3$, 500 MHz

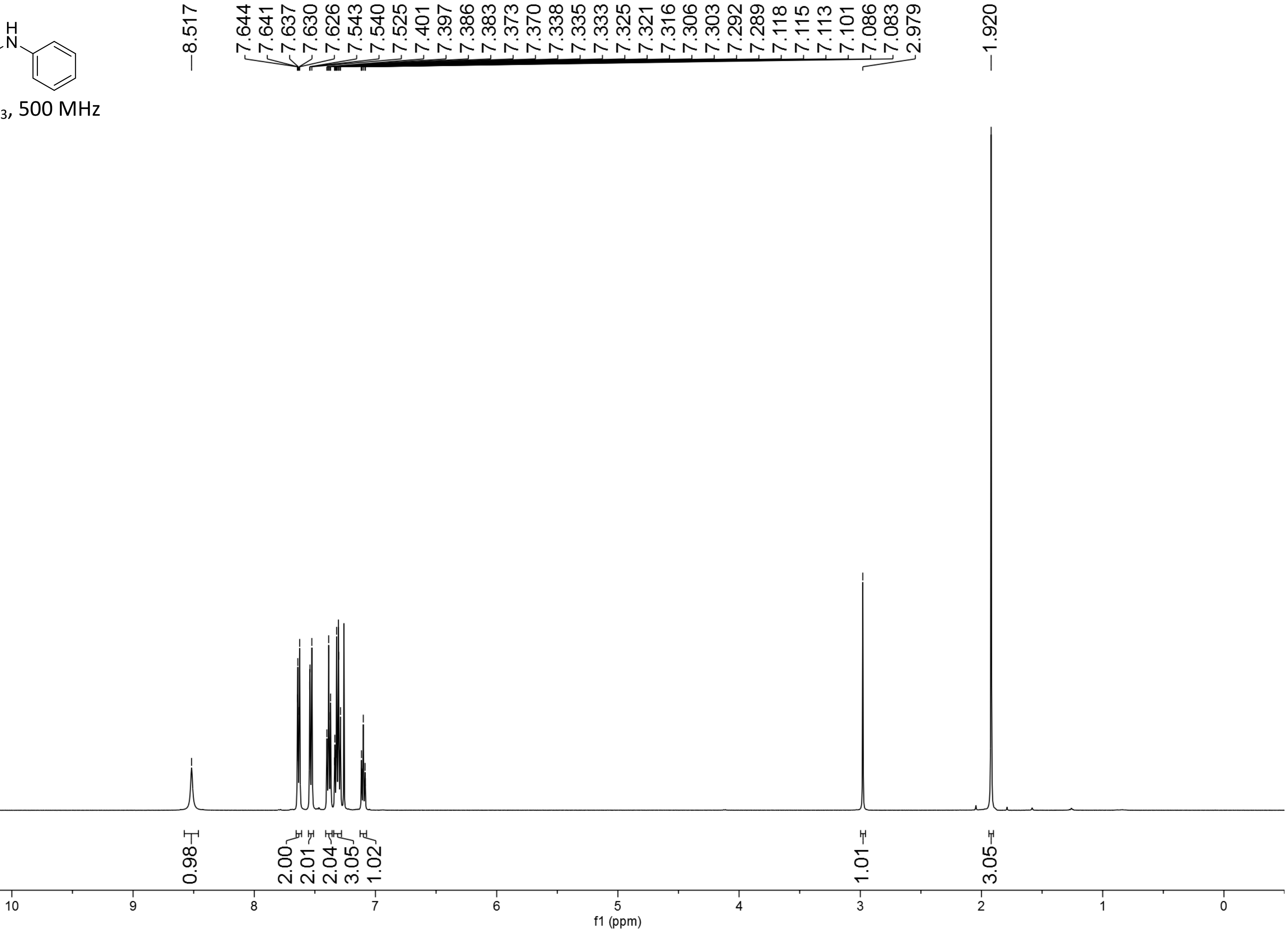

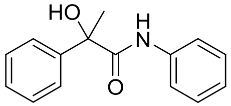

3: ^{13}C, $CDCl_3$, 126 MHz

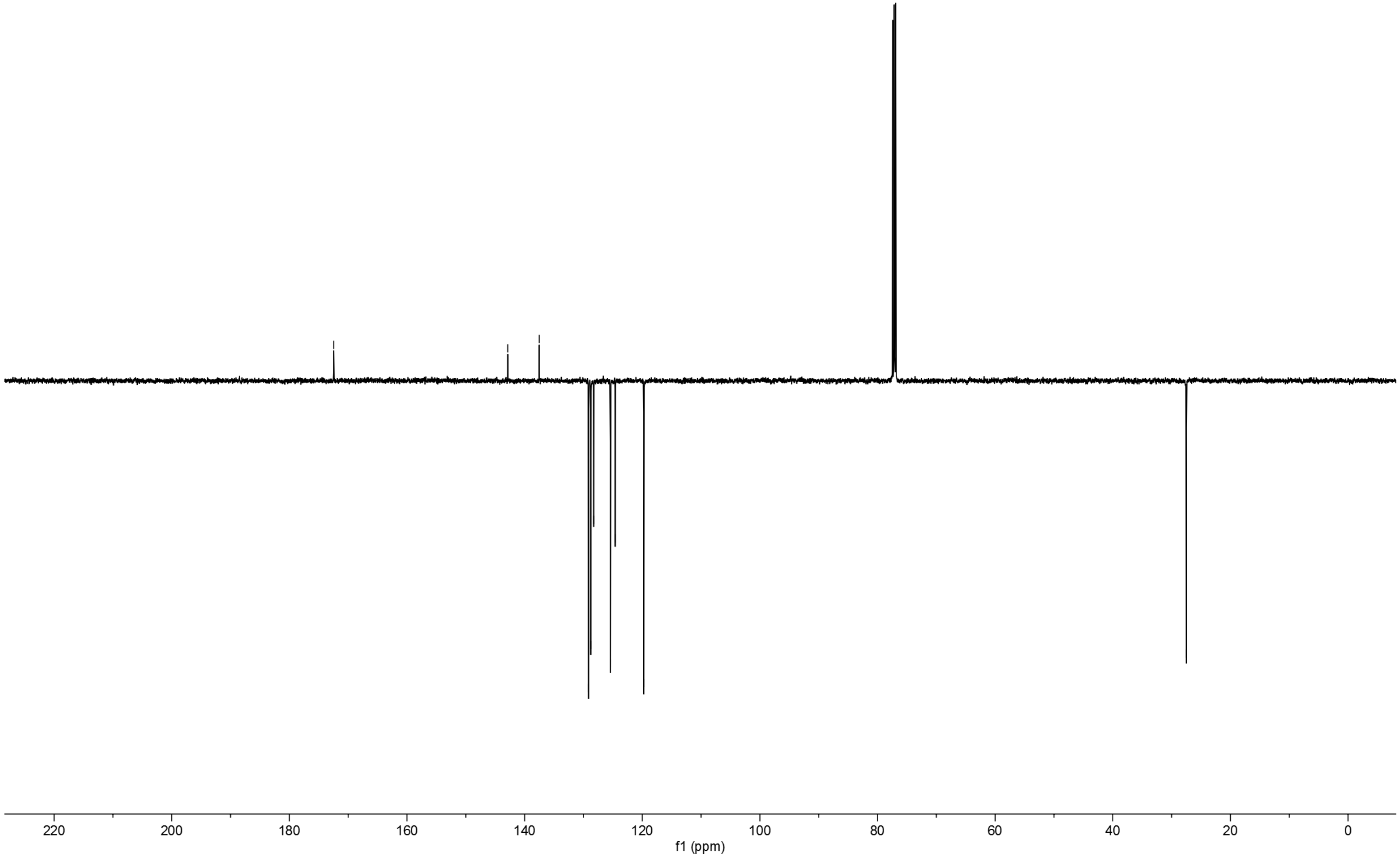

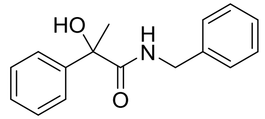

S3: ^{1}H, $CDCl_3$, 500 MHz

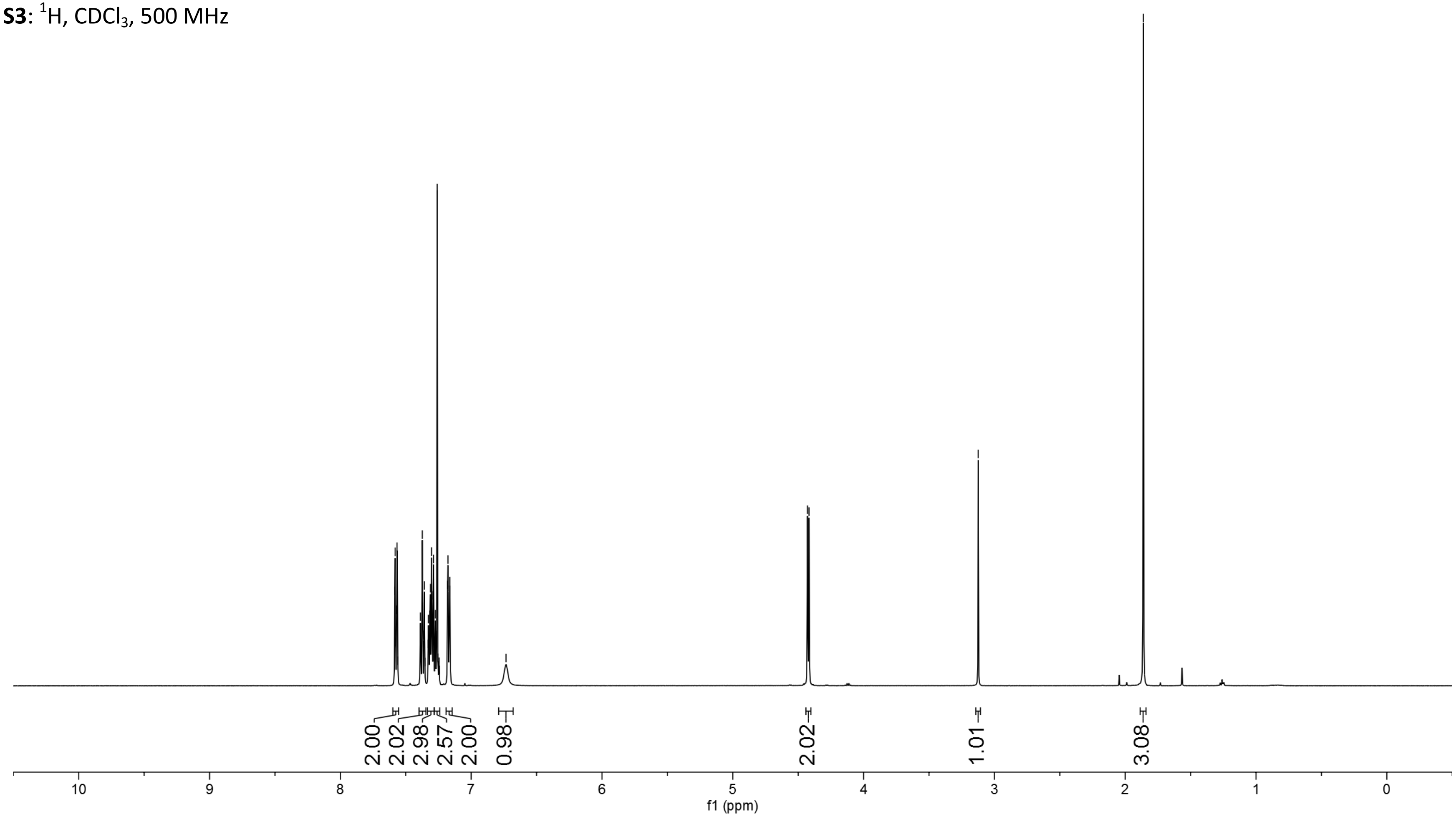

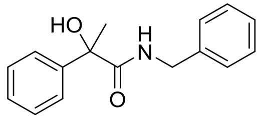

S3: ^{13}C, $CDCl_3$, 126 MHz

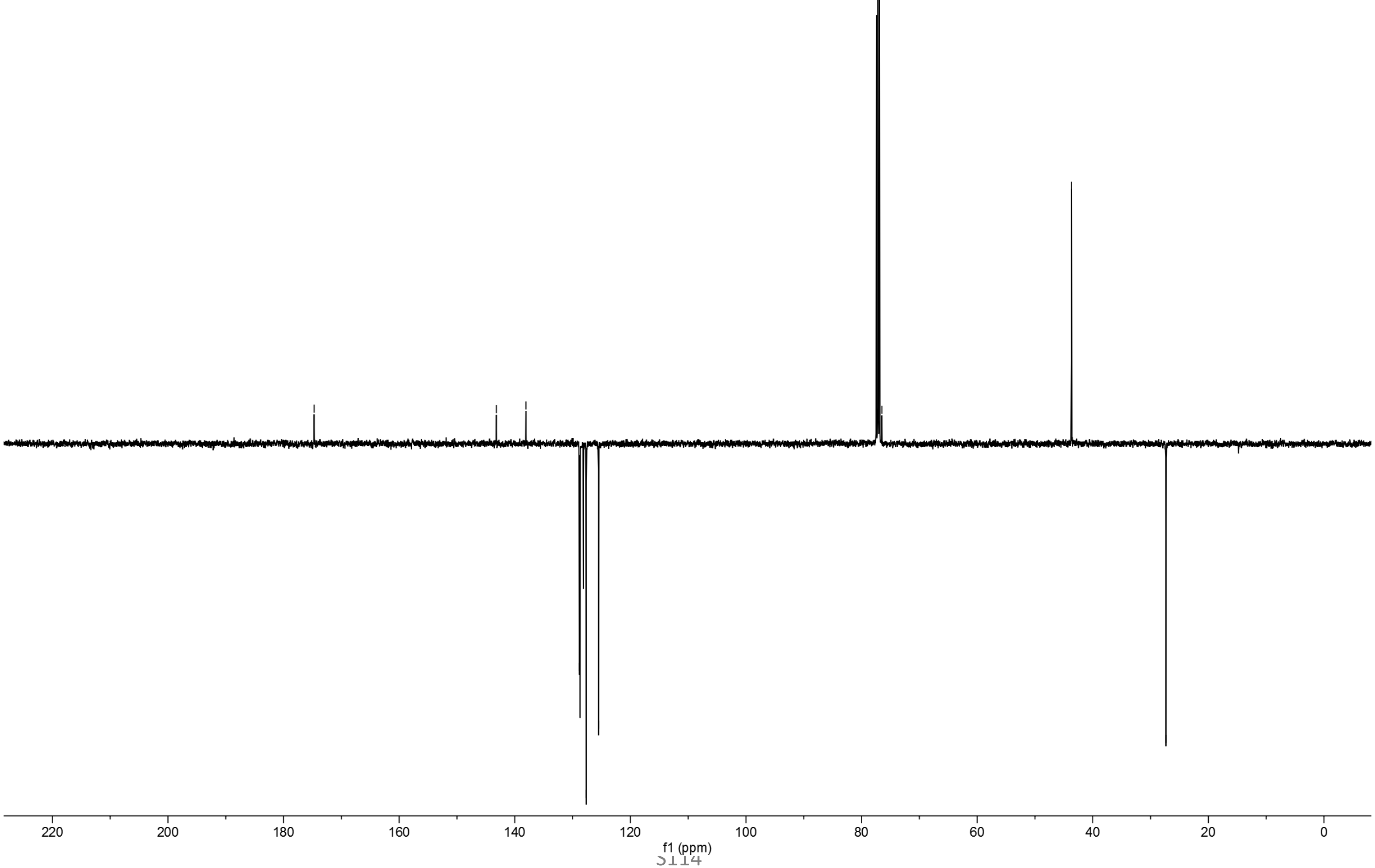

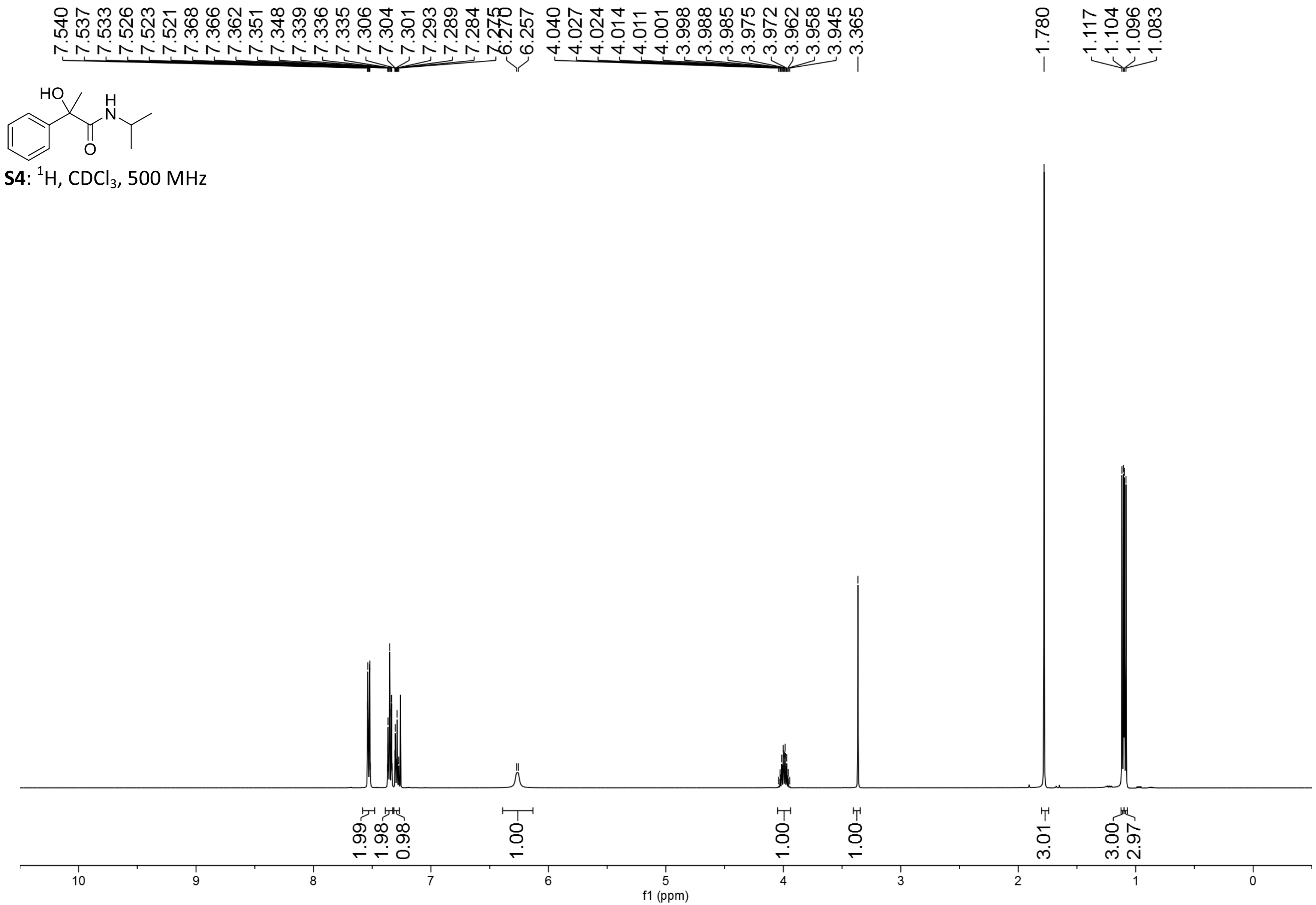

S4: ^{1}H, $CDCl_3$, 500 MHz

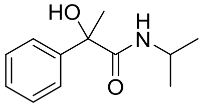

S4: ^{13}C, $CDCl_3$, 126 MHz

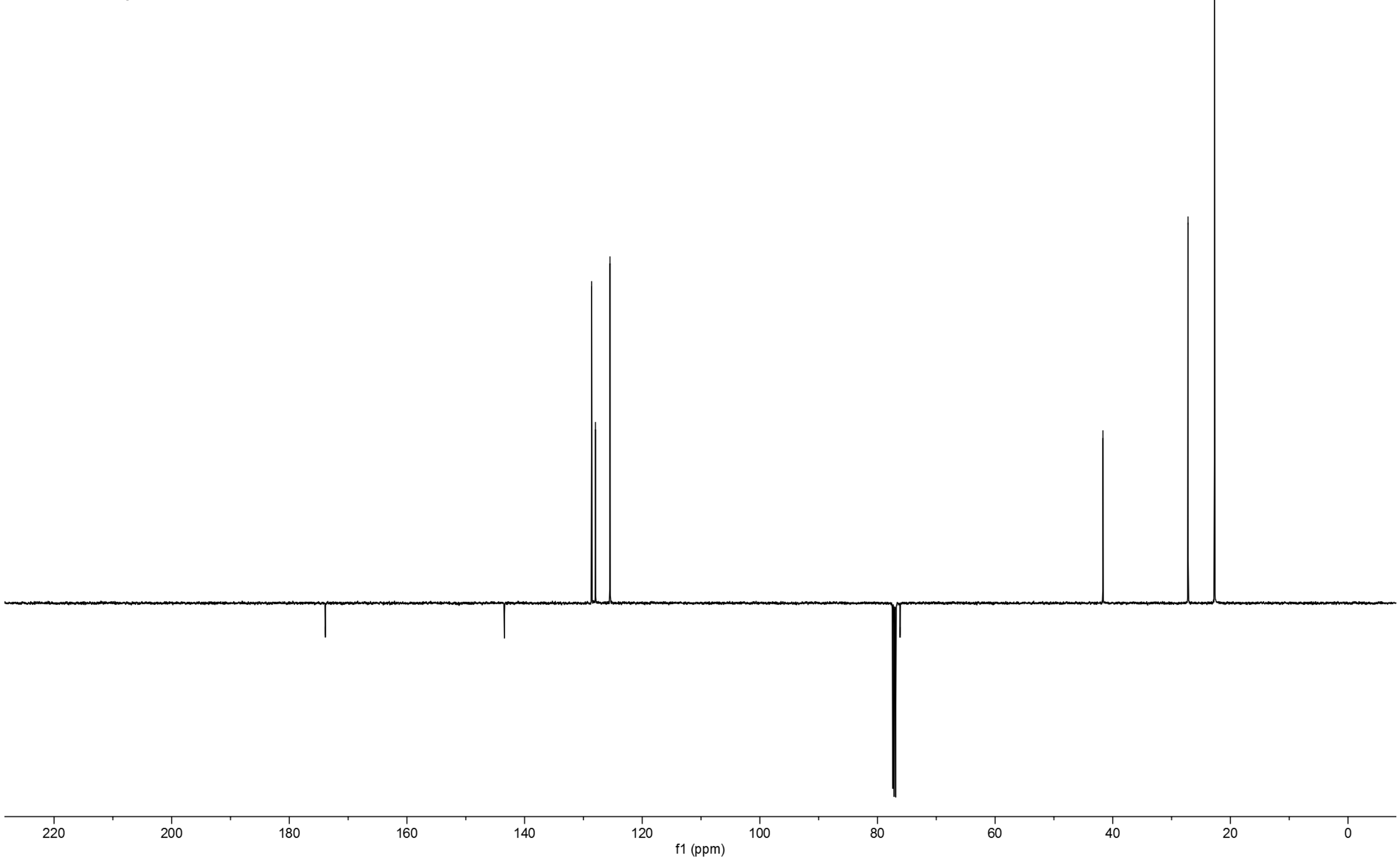

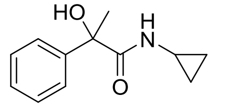

S5: ^1H, $CDCl_3$, 500 MHz

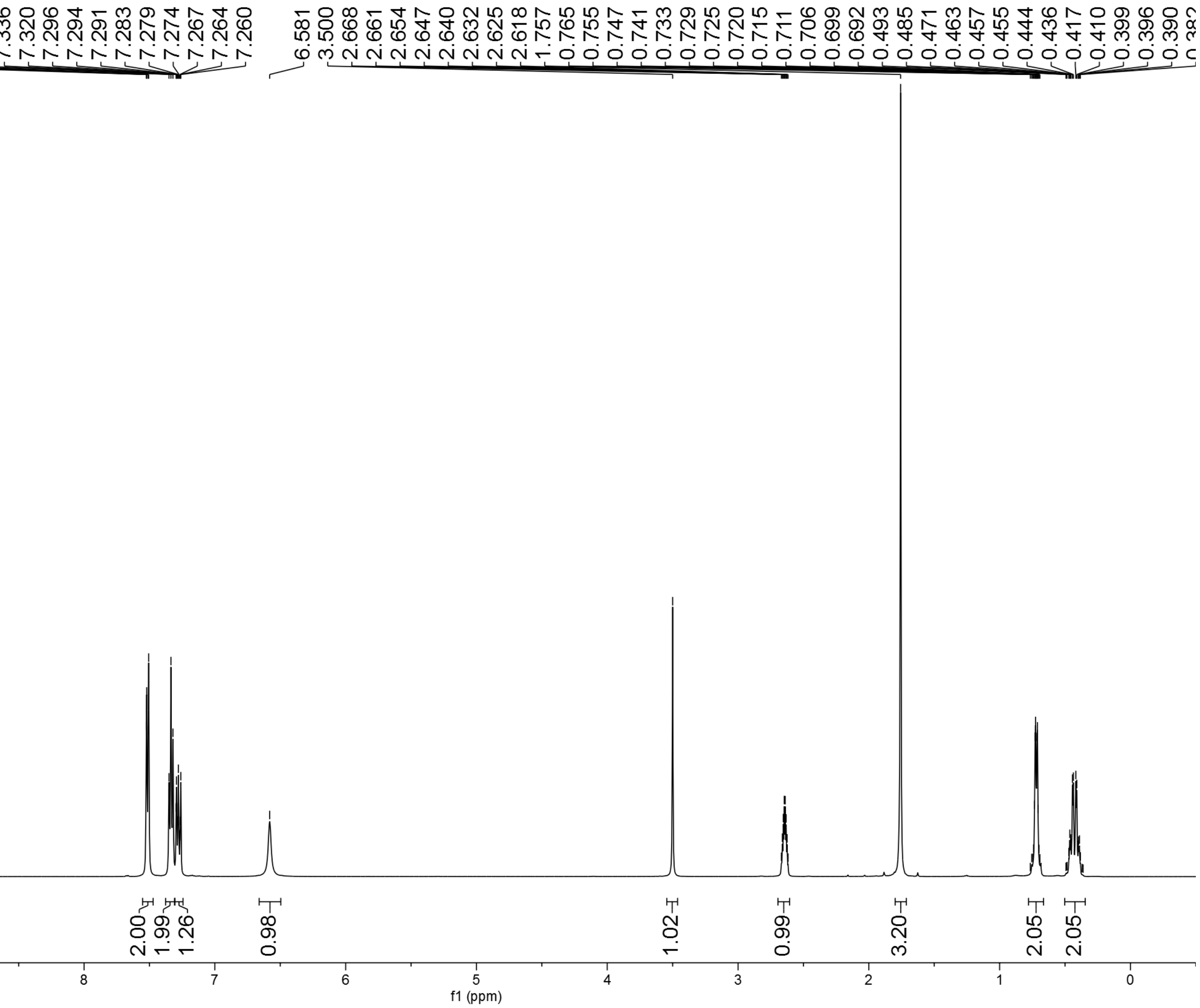

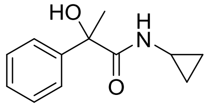

S5: ^{13}C, $CDCl_3$, 126 MHz

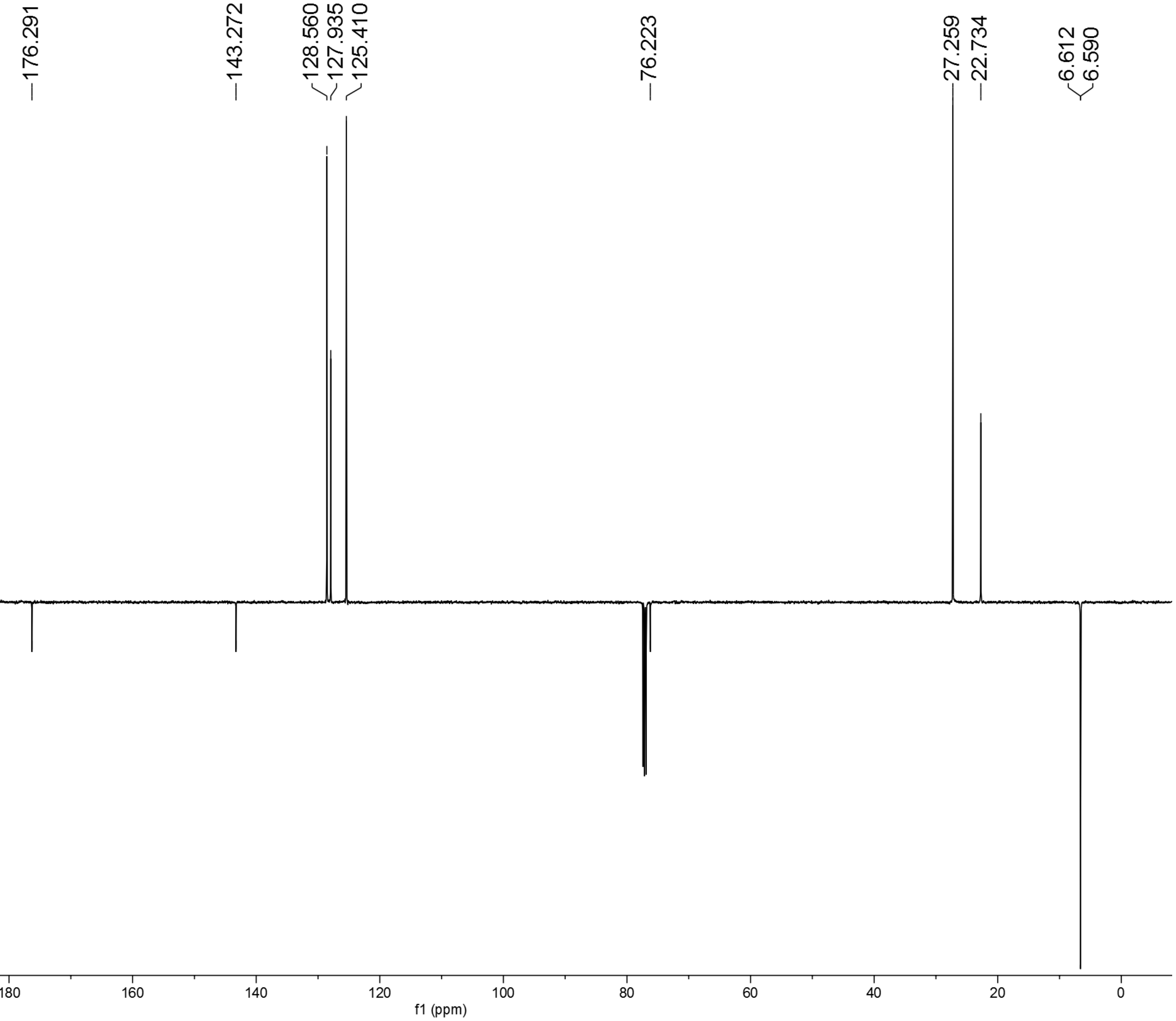

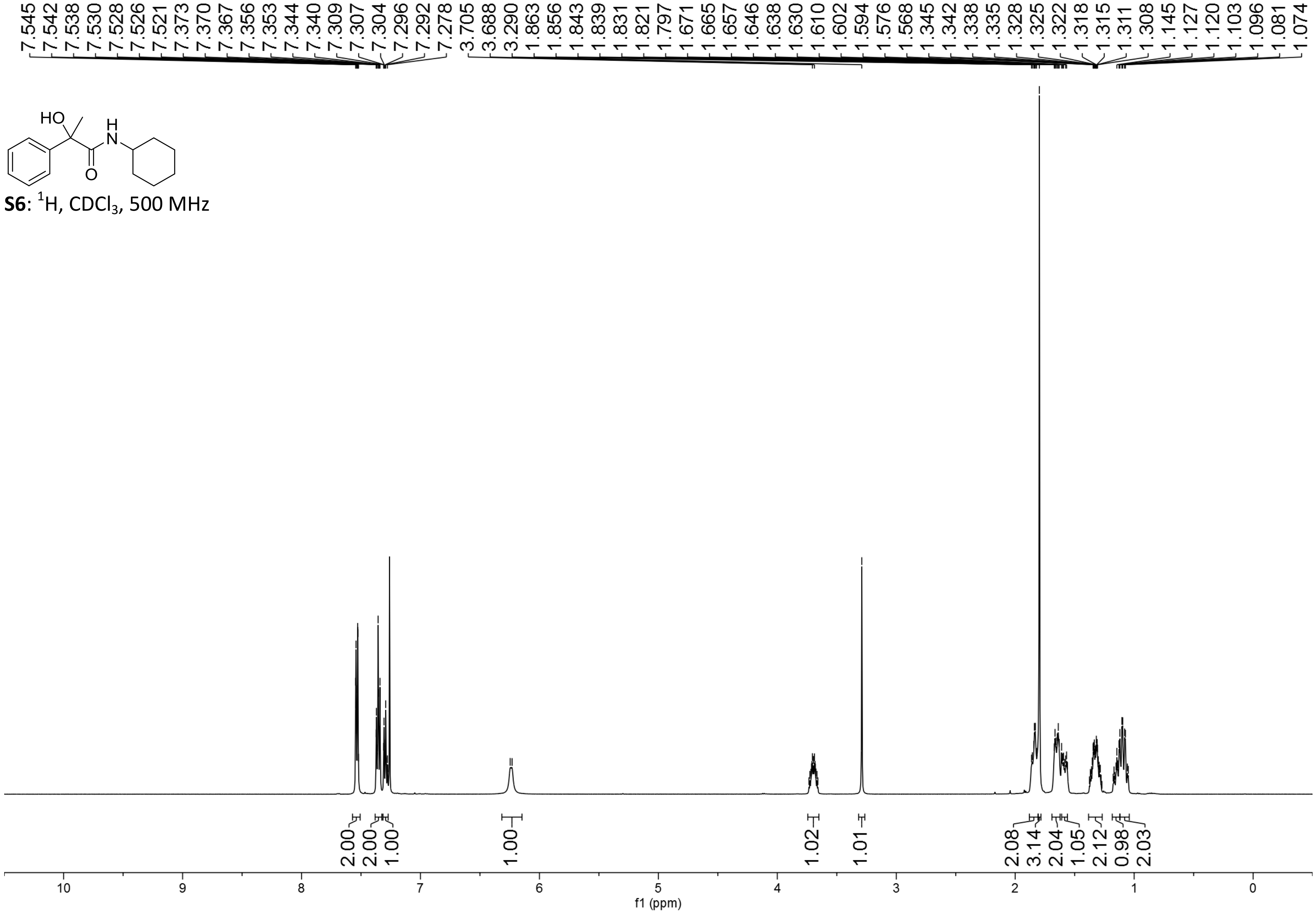

S6: ^{1}H, $CDCl_3$, 500 MHz

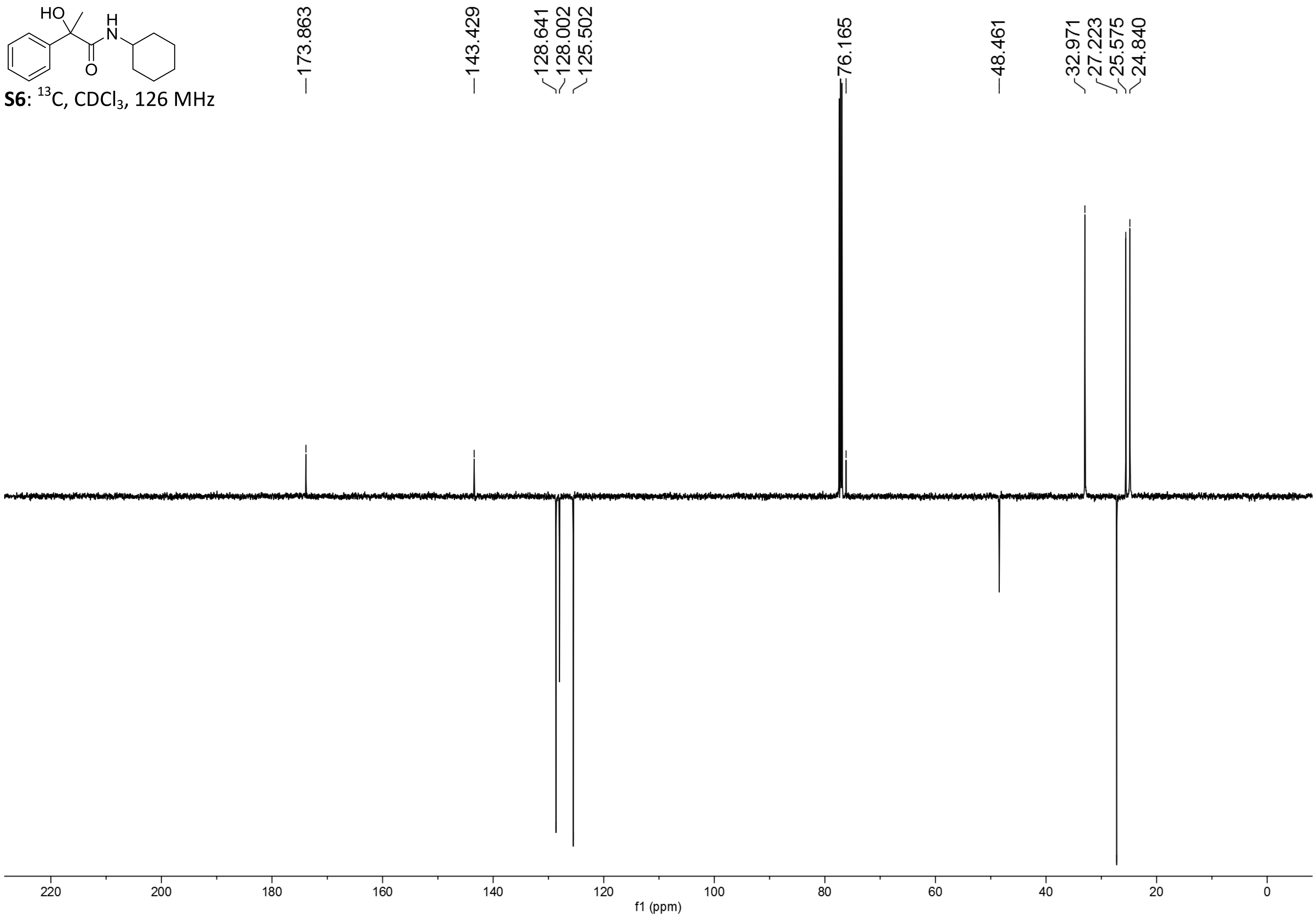

S6: ^{13}C, $CDCl_3$, 126 MHz

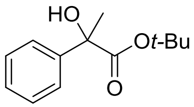

7: ^{1}H, $CDCl_3$, 400 MHz

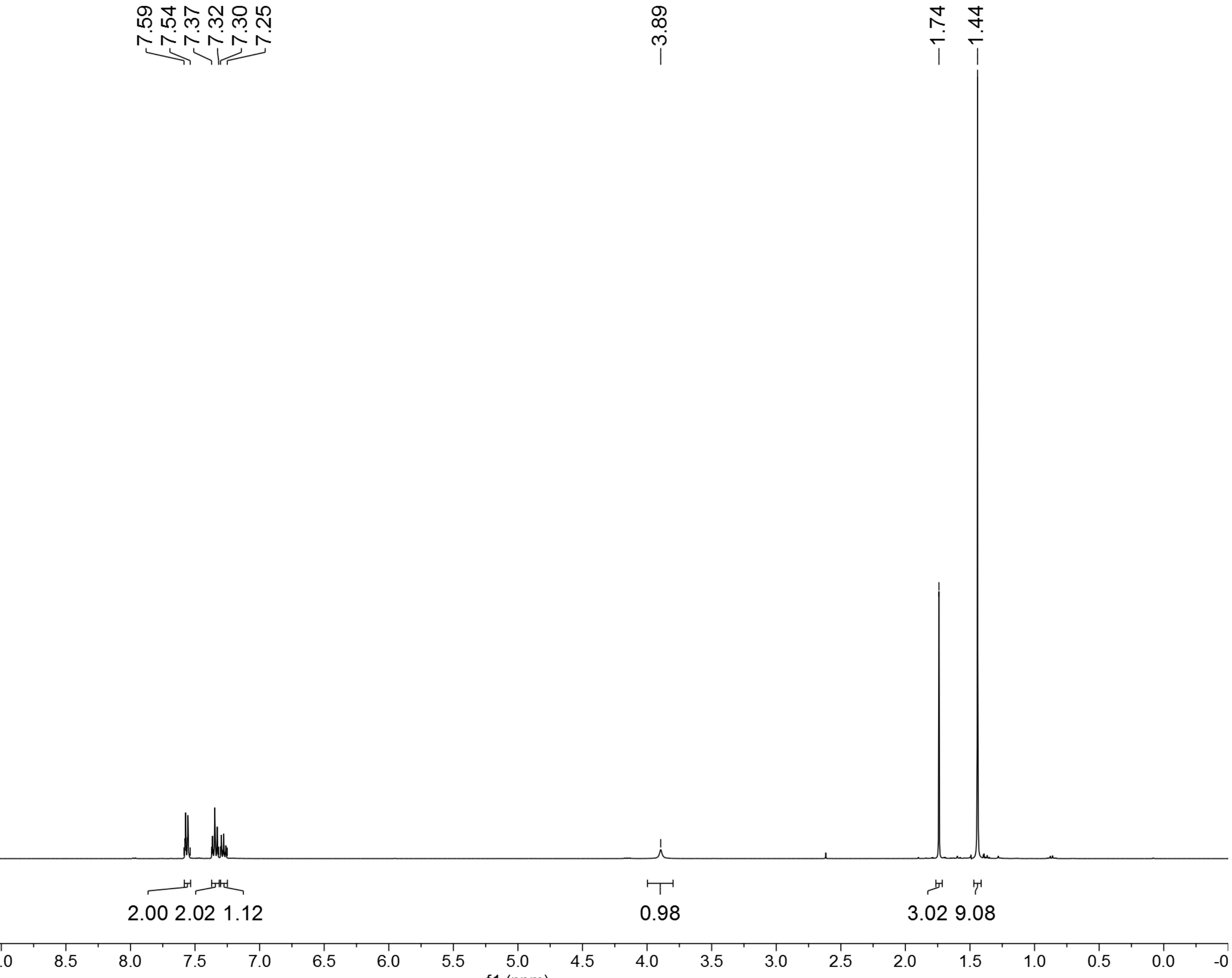

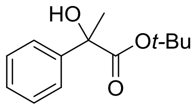

7: ^{13}C, $CDCl_3$, 100 MHz

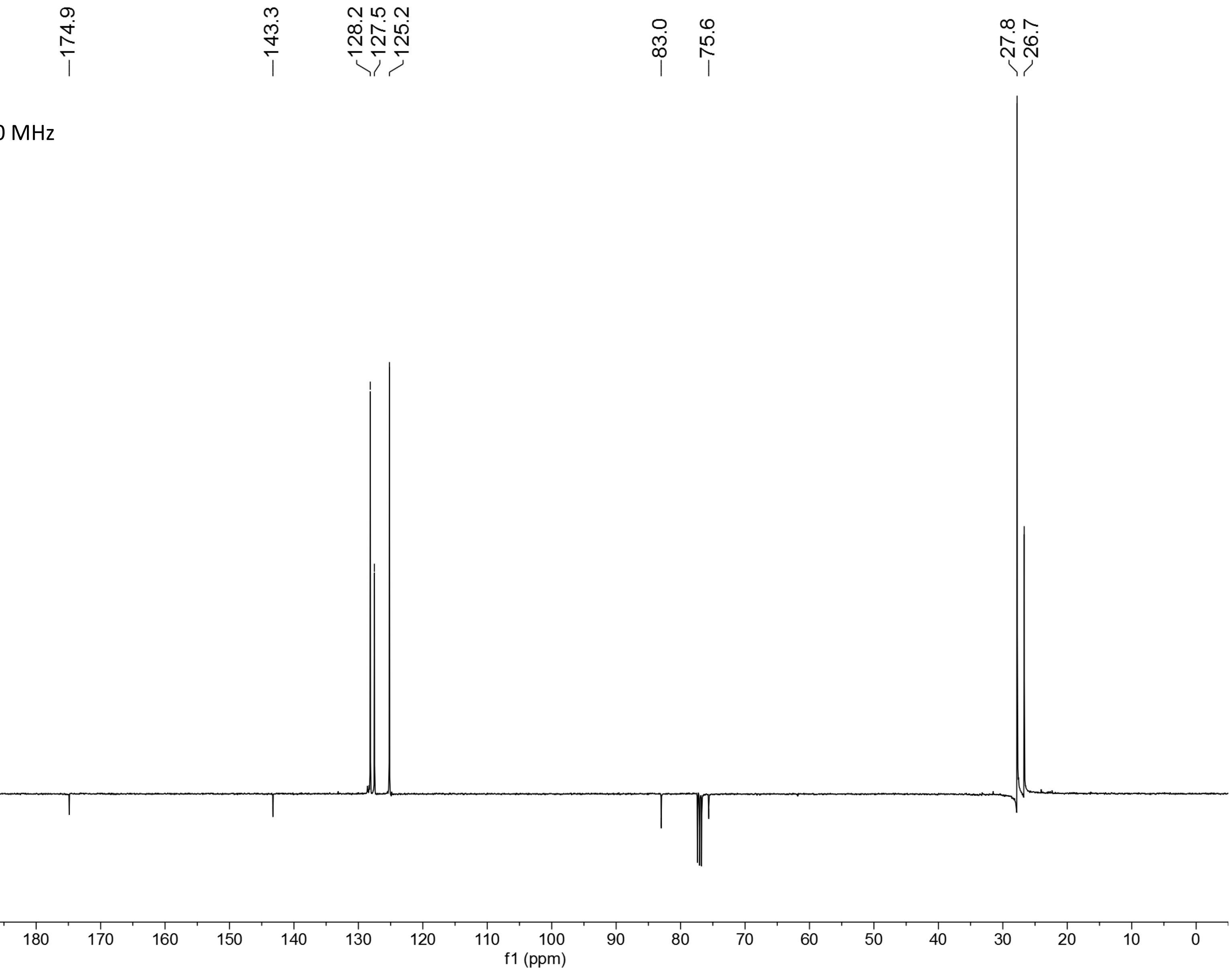

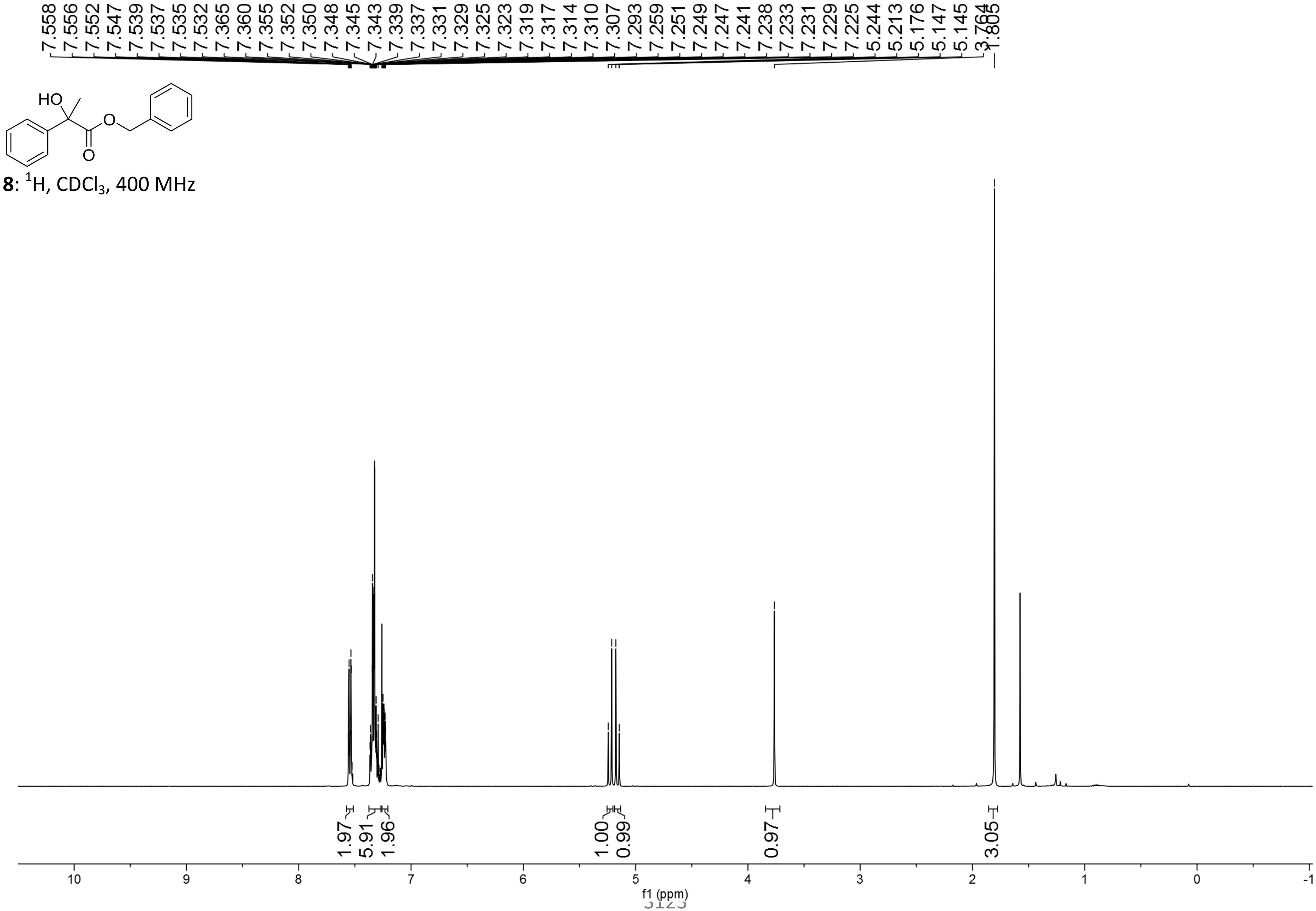

8: ^{1}H, $CDCl_3$, 400 MHz

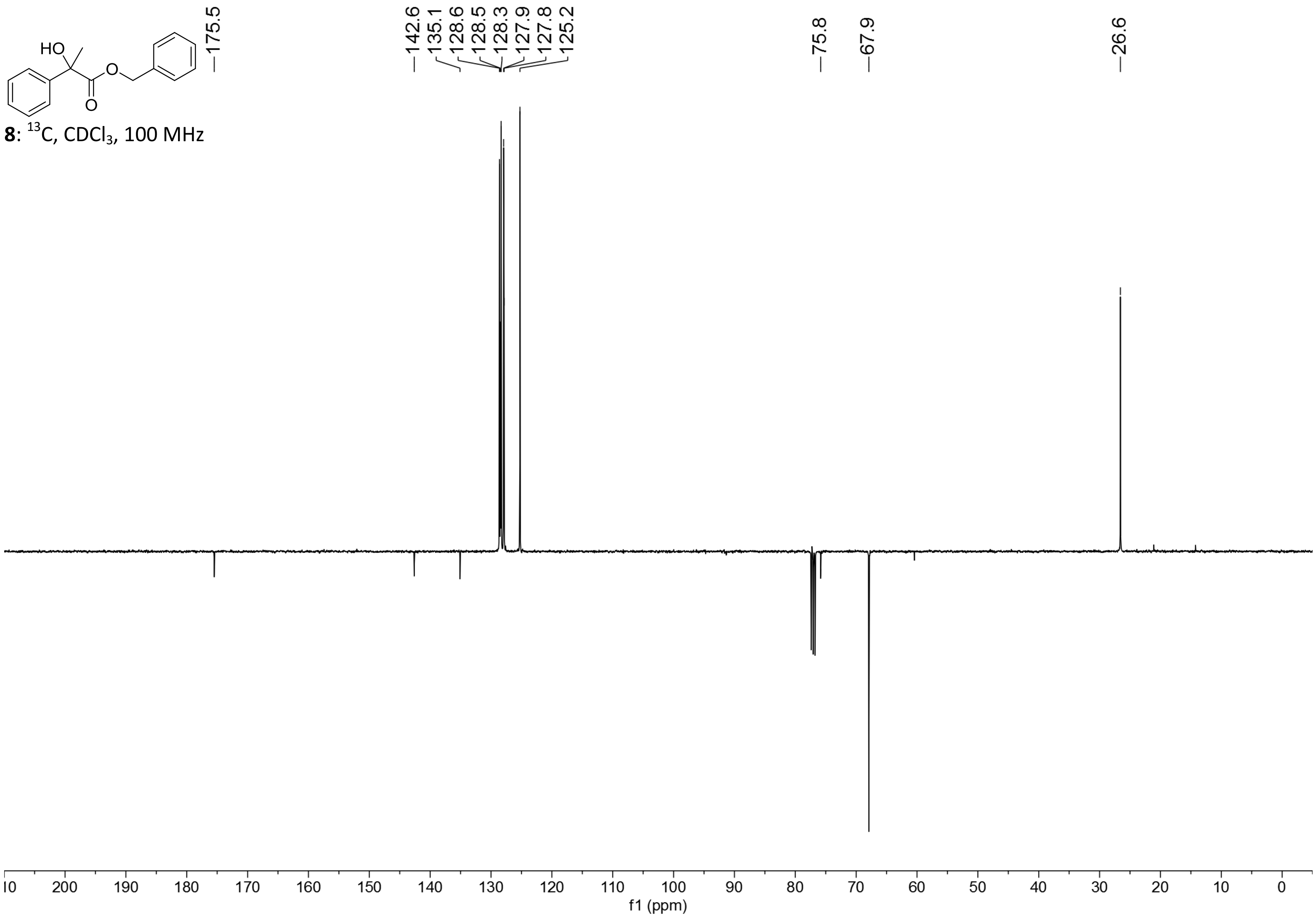

8: ^{13}C, $CDCl_3$, 100 MHz

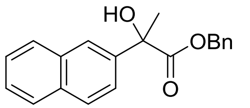

11: ^{1}H, $CDCl_3$, 400 MHz

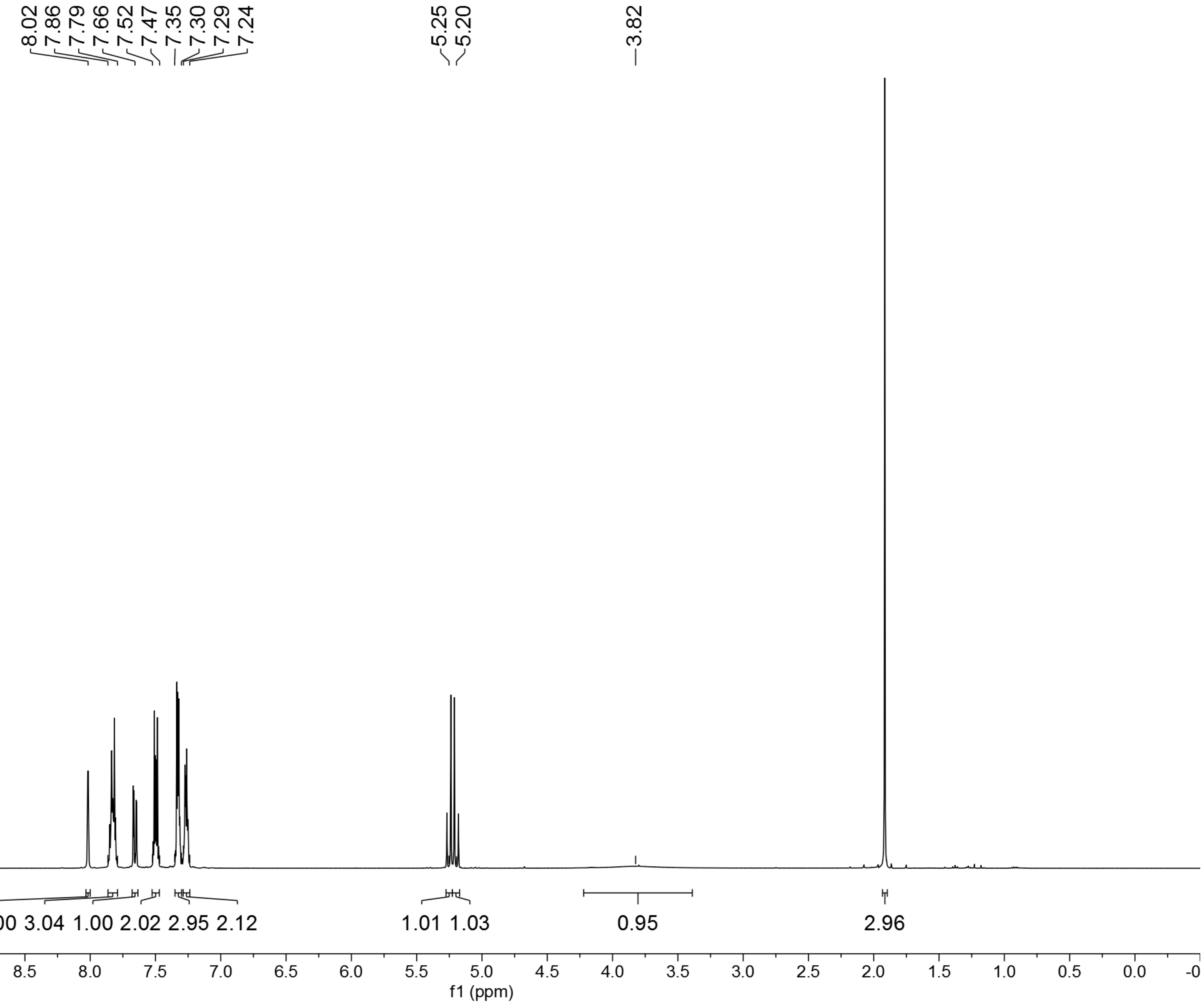

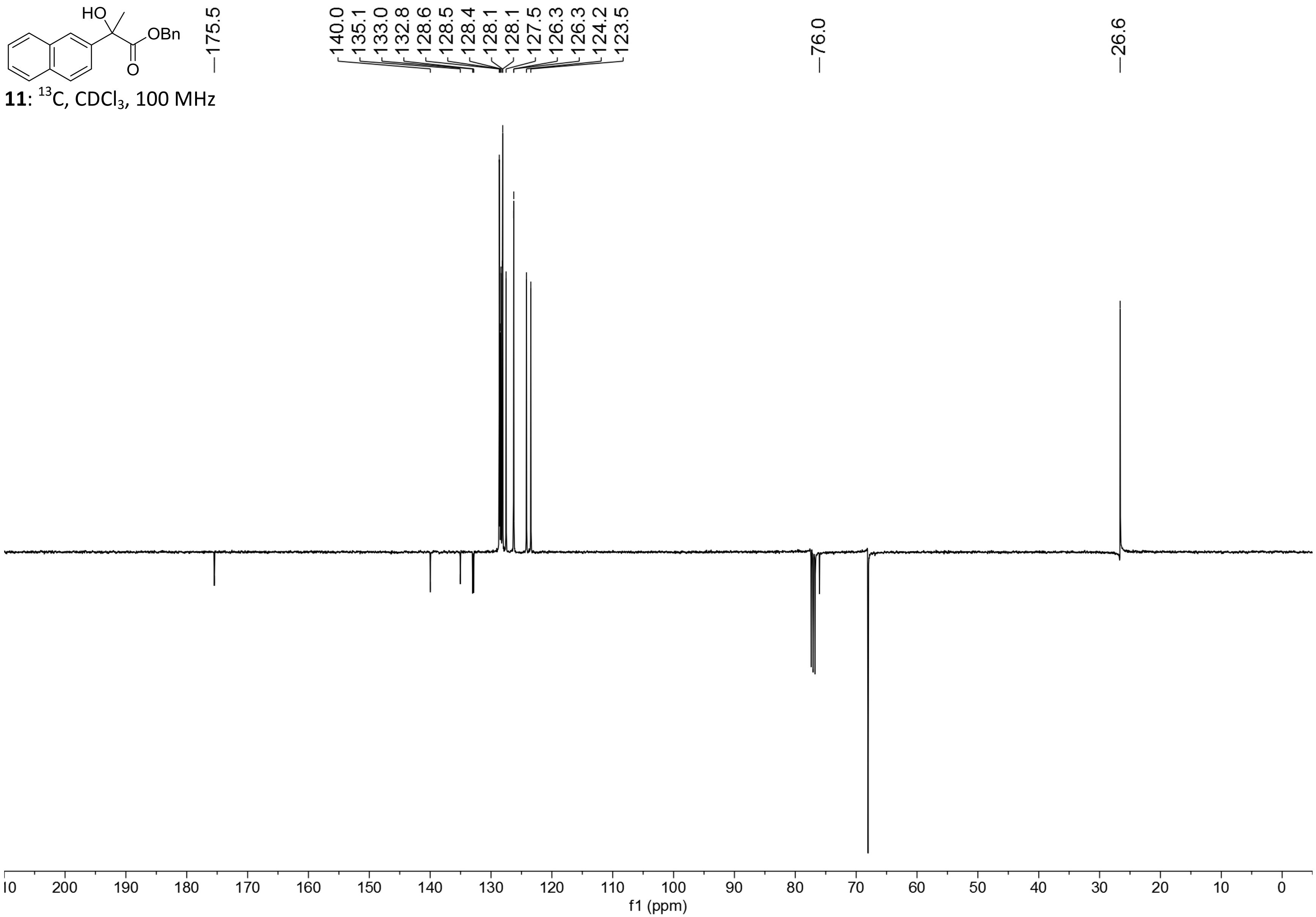
HO
OBn
O
11: ^{13}C, $CDCl_3$, 100 MHz
175.5
140.0
135.1
133.0
132.8
128.6
128.5
128.4
128.1
128.1
127.5
126.3
126.3
124.2
123.5
76.0
26.6
f1 (ppm)

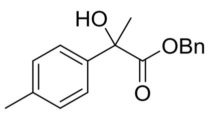

12: ^1H, $CDCl_3$, 400 MHz

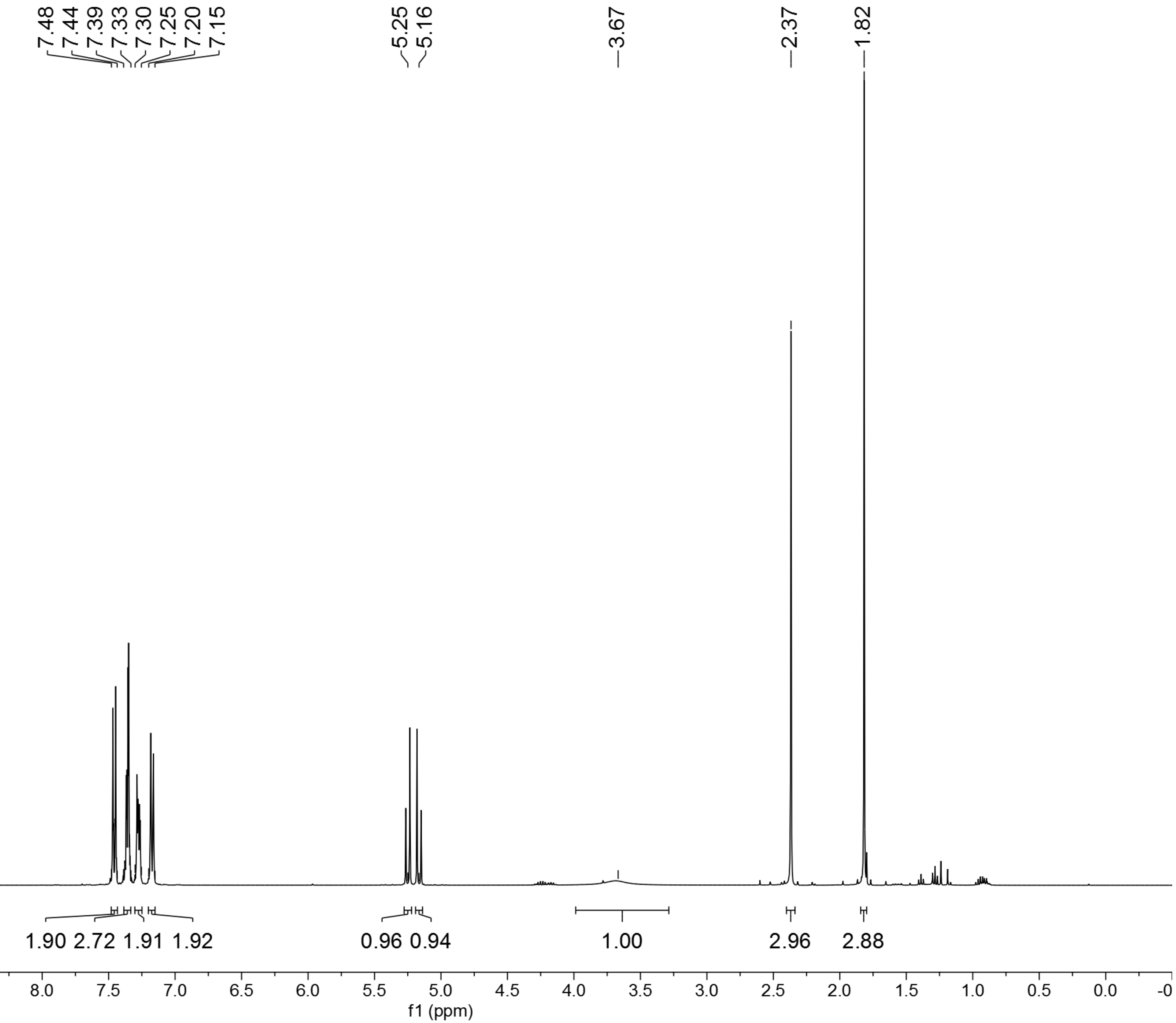

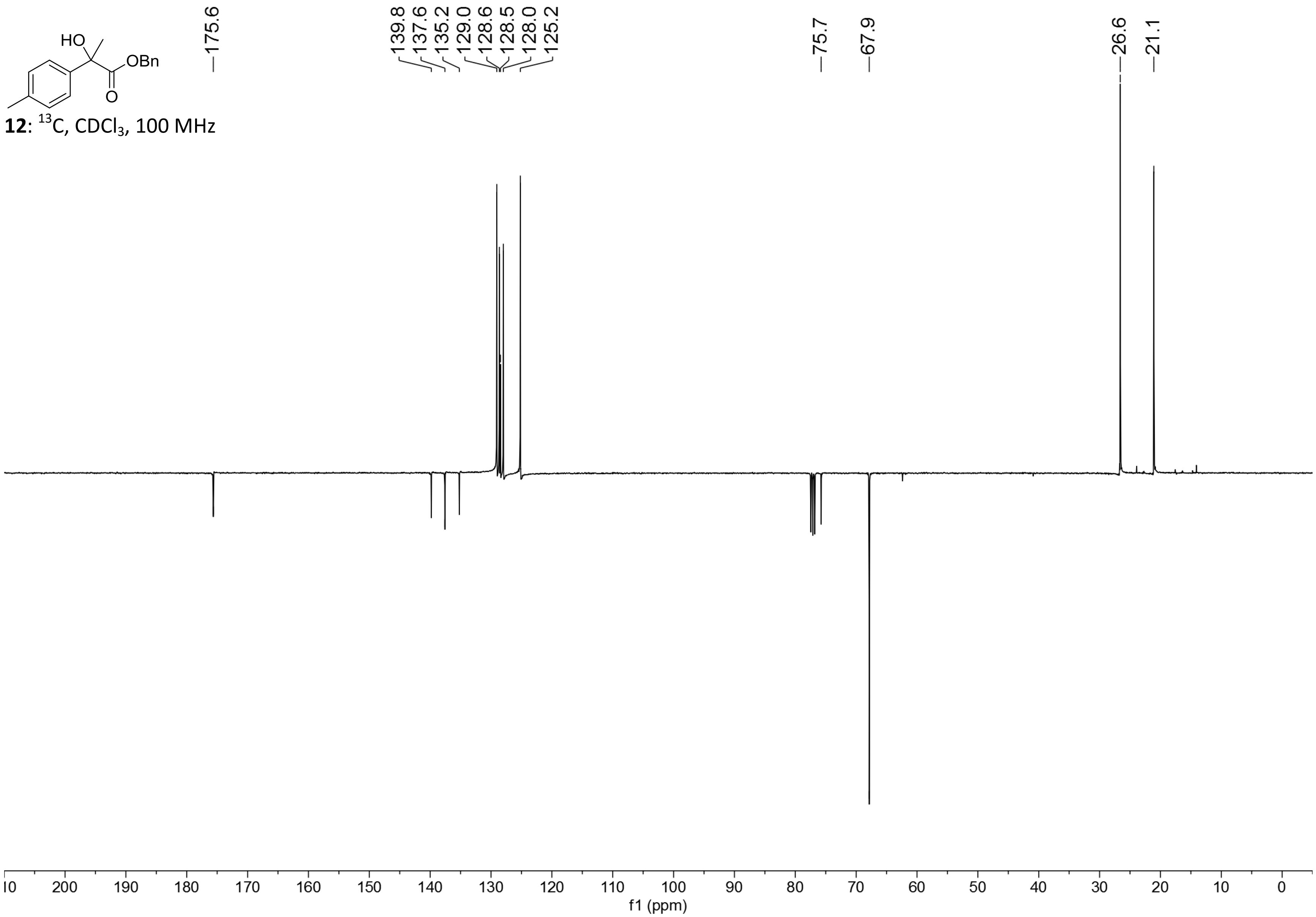

12: ^{13}C, $CDCl_3$, 100 MHz

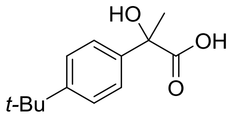

S7: ^{1}H, $CDCl_3$, 500 MHz

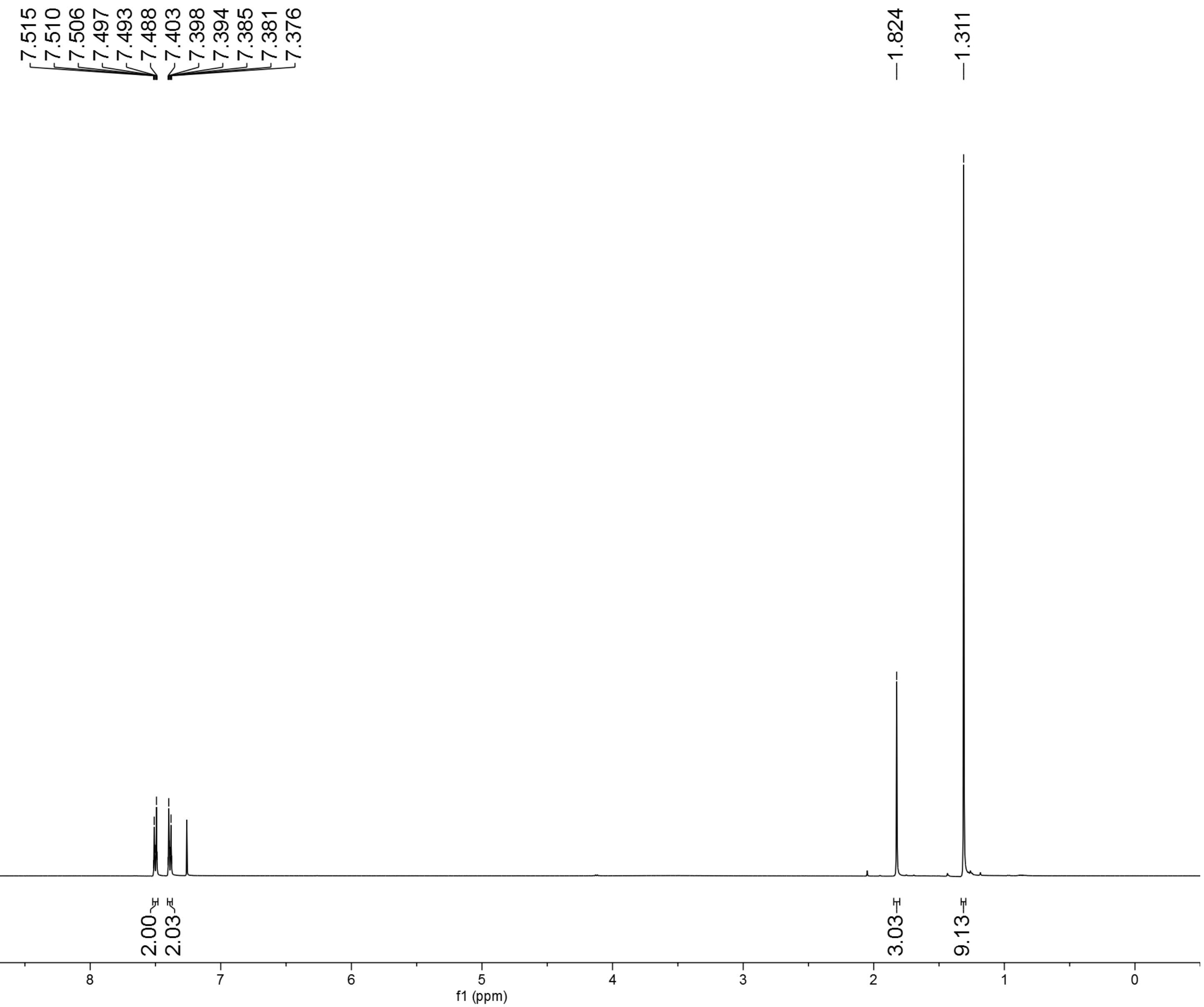

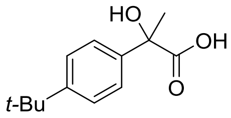

S7: ^{13}C, $CDCl_3$, 126 MHz

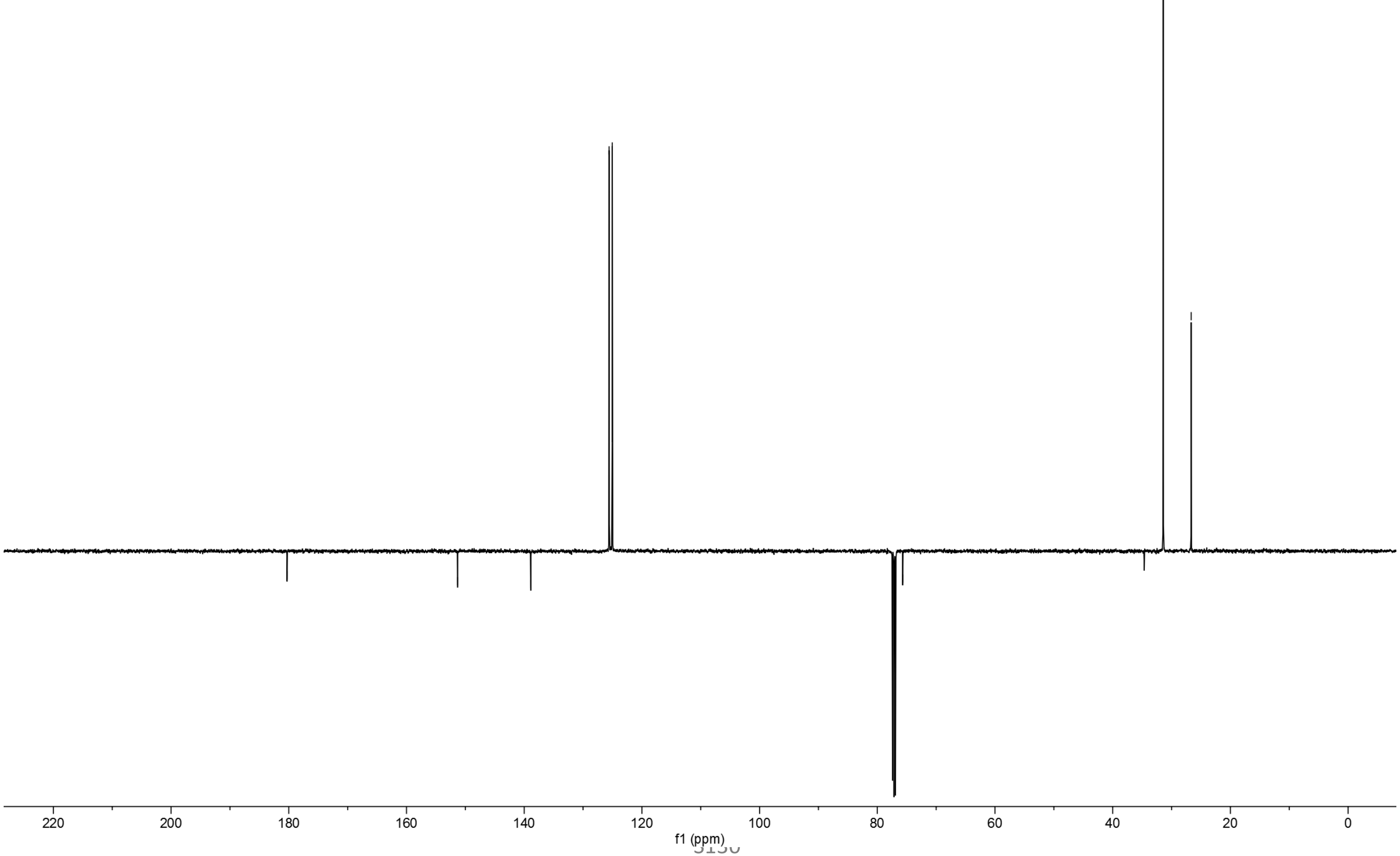

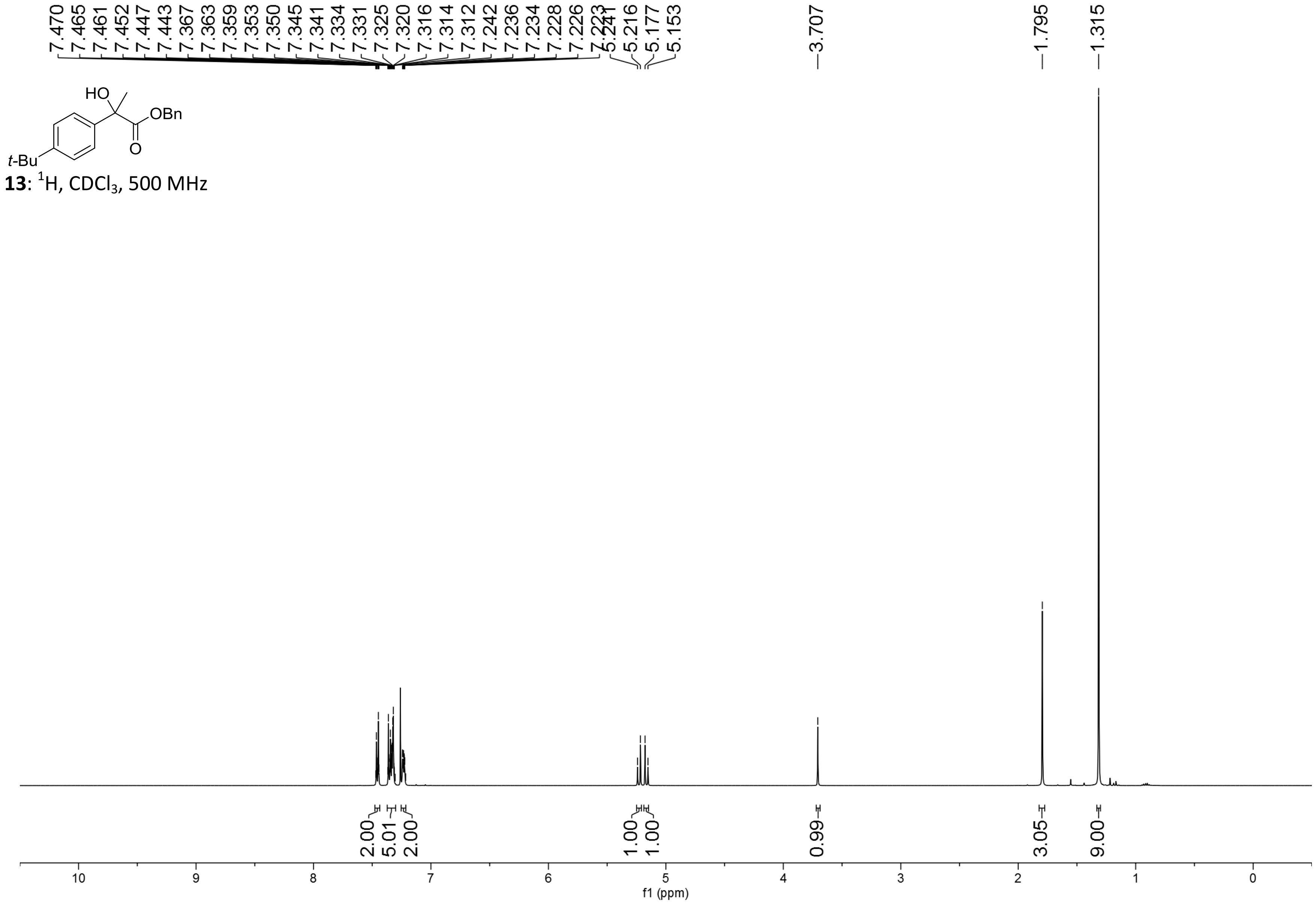

13: ^{1}H, $CDCl_3$, 500 MHz

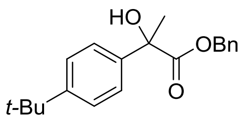

13: ^{13}C, $CDCl_3$, 126 MHz

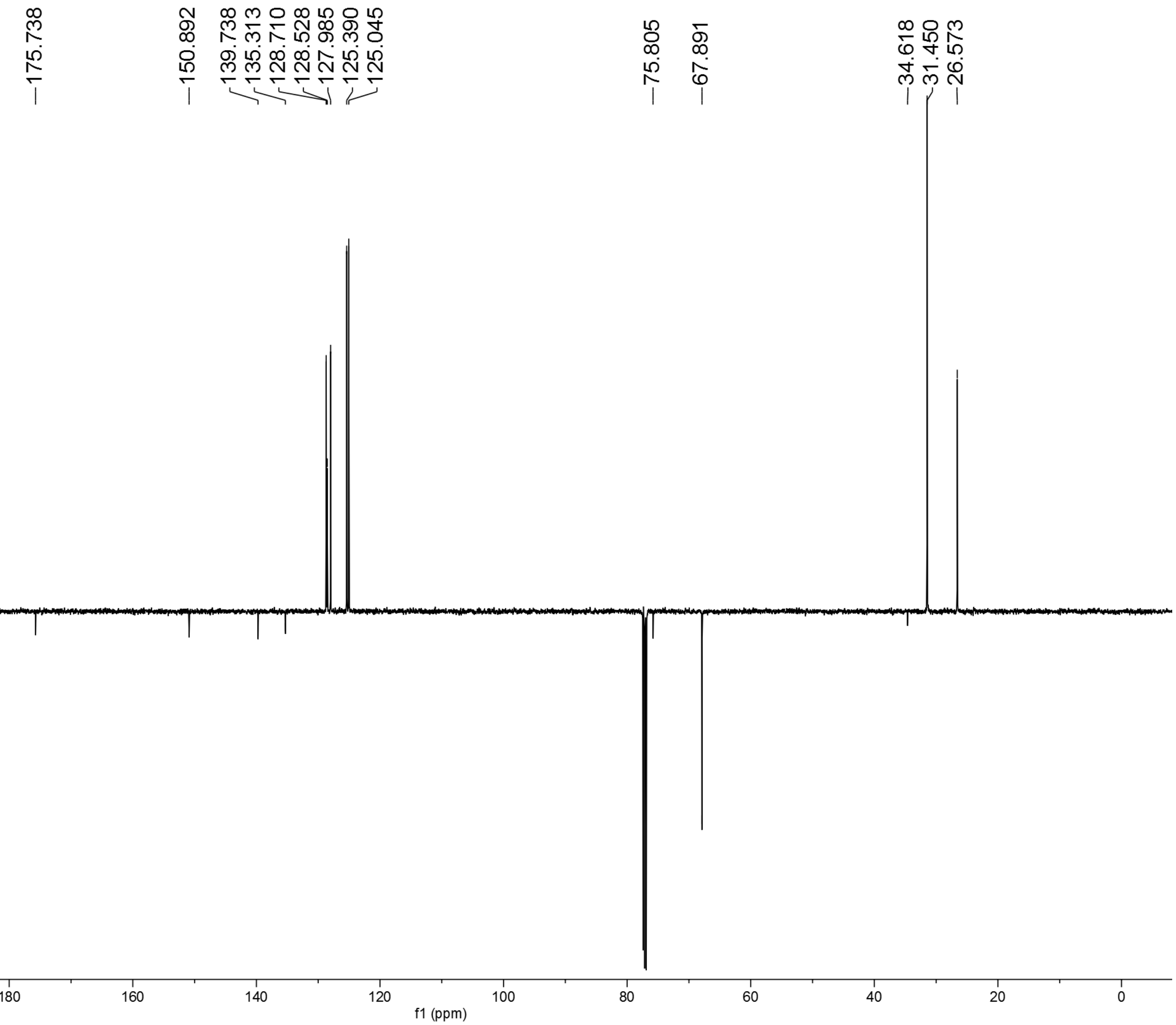

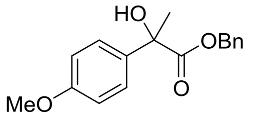

14: ^{1}H, CDCl$_3$, 400 MHz

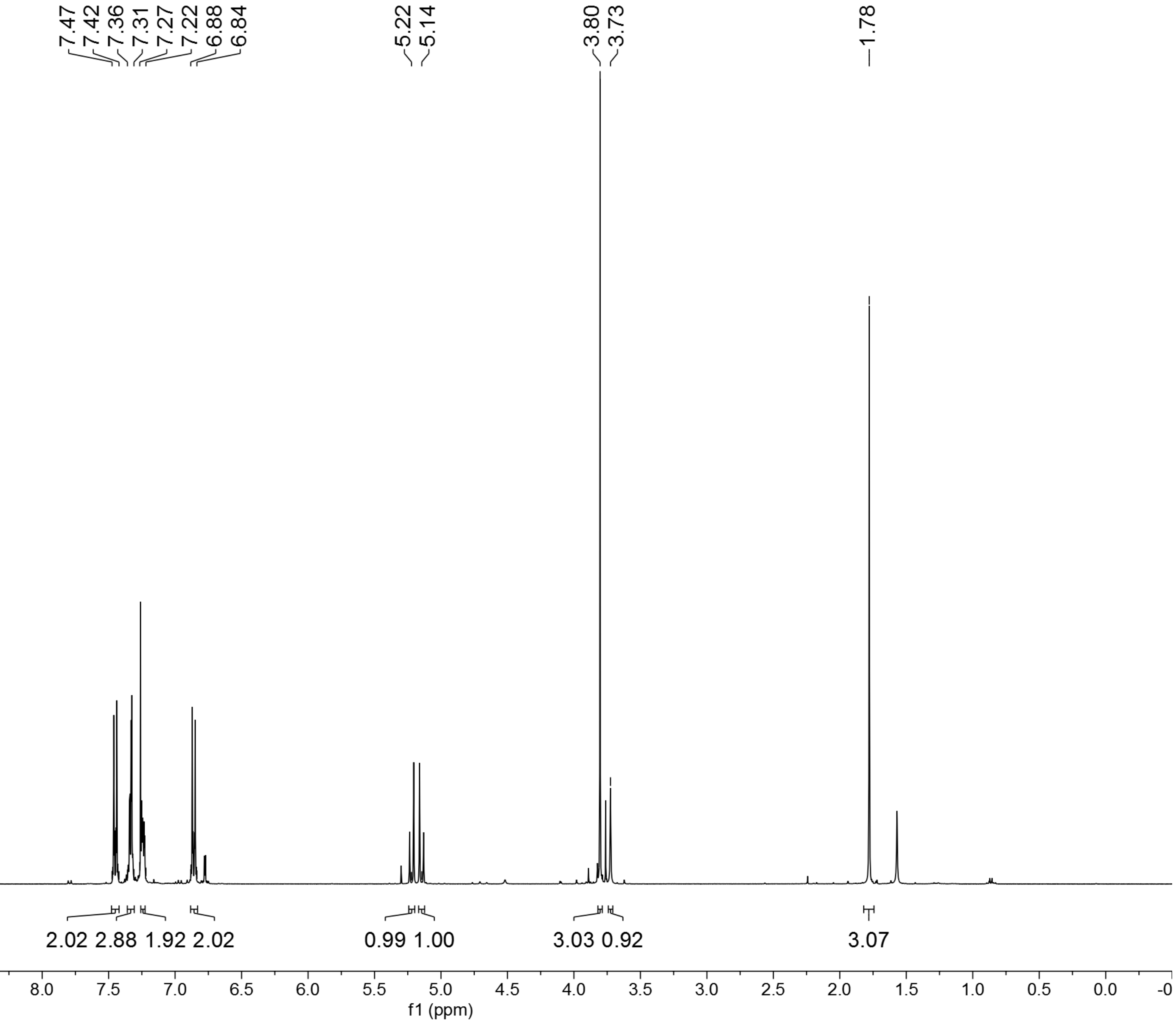

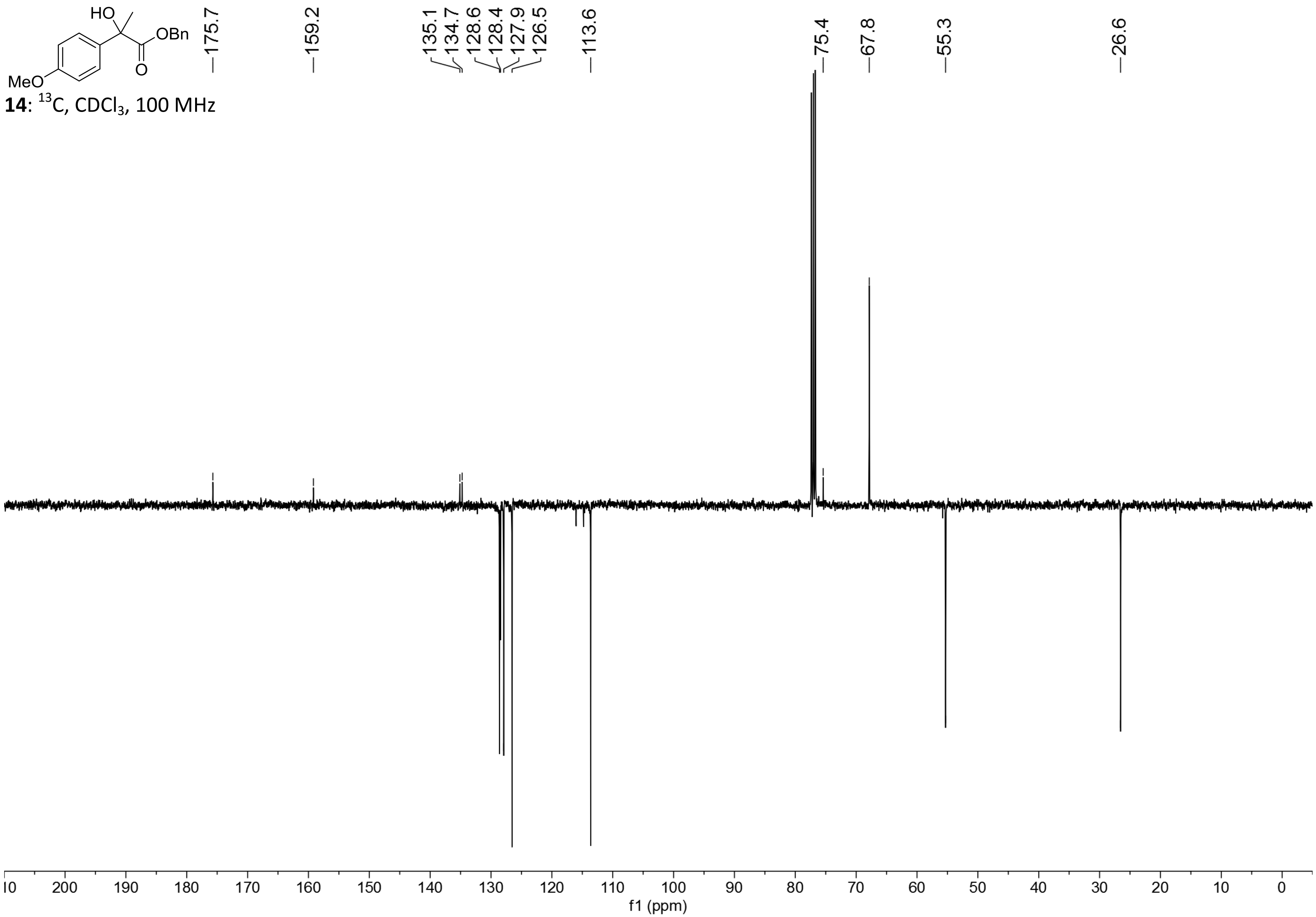

14: ^{13}C, $CDCl_3$, 100 MHz

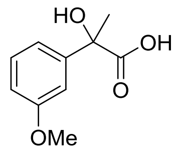

S9: ^{1}H, $CDCl_3$, 500 MHz

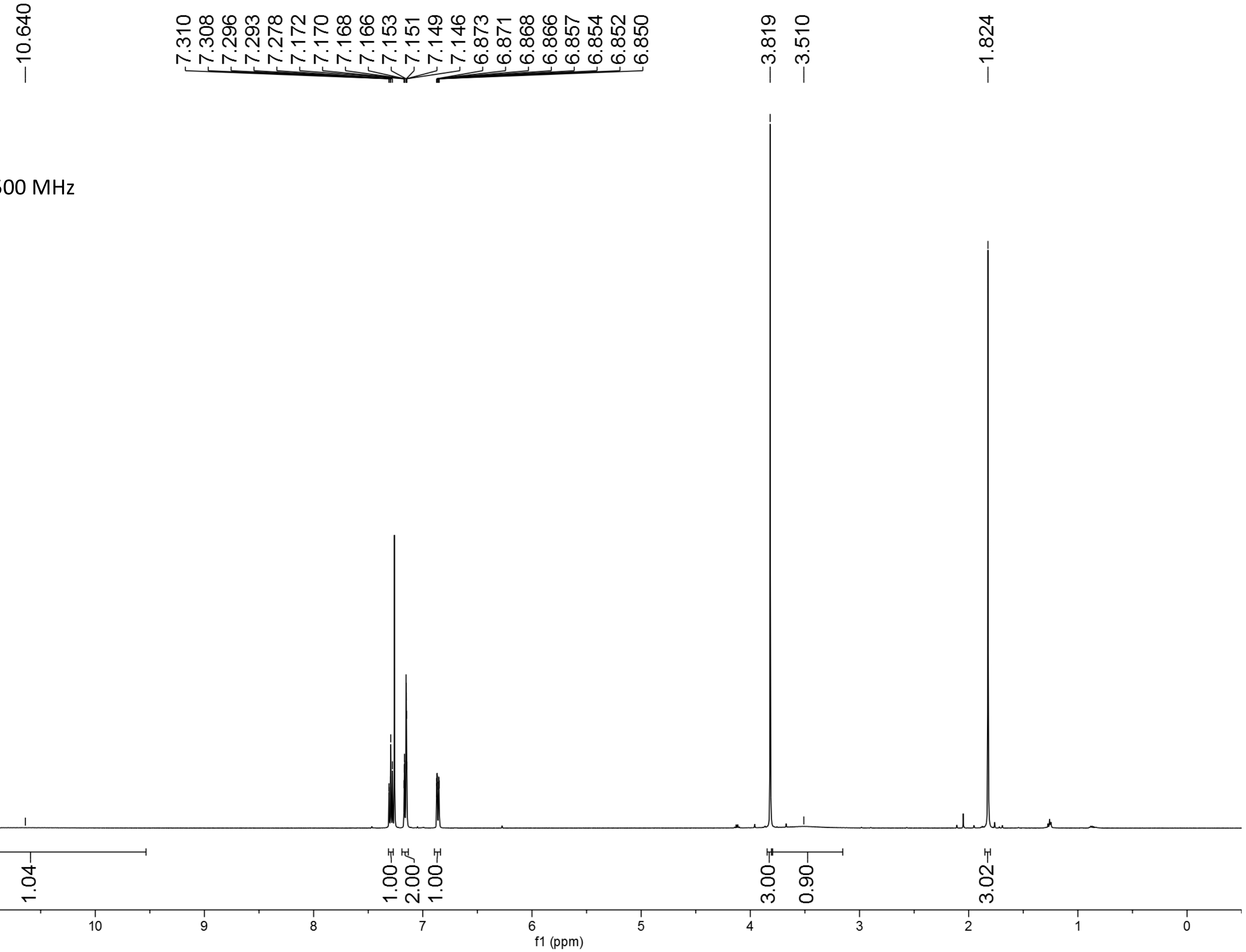

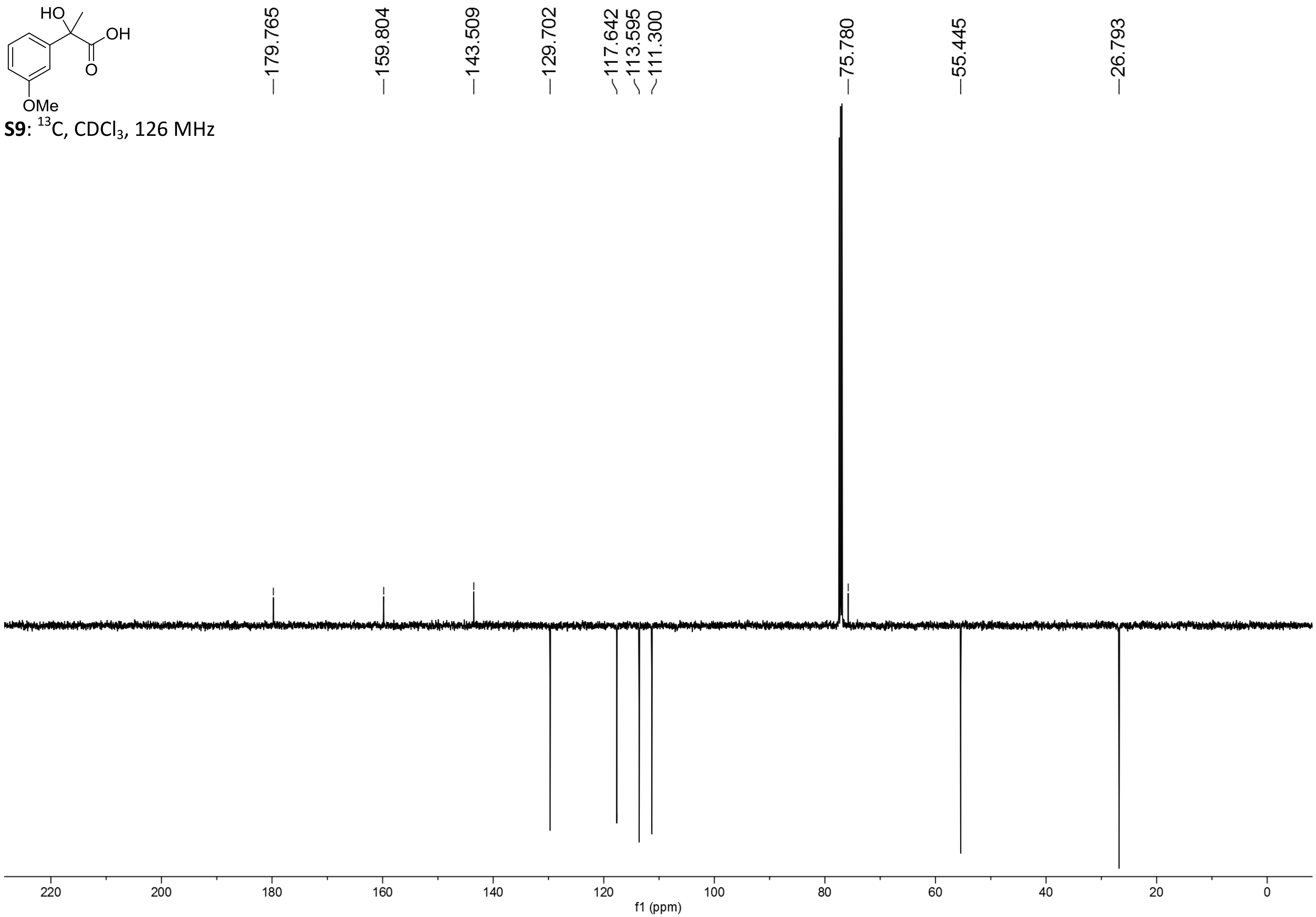

S9: ^{13}C, $CDCl_3$, 126 MHz

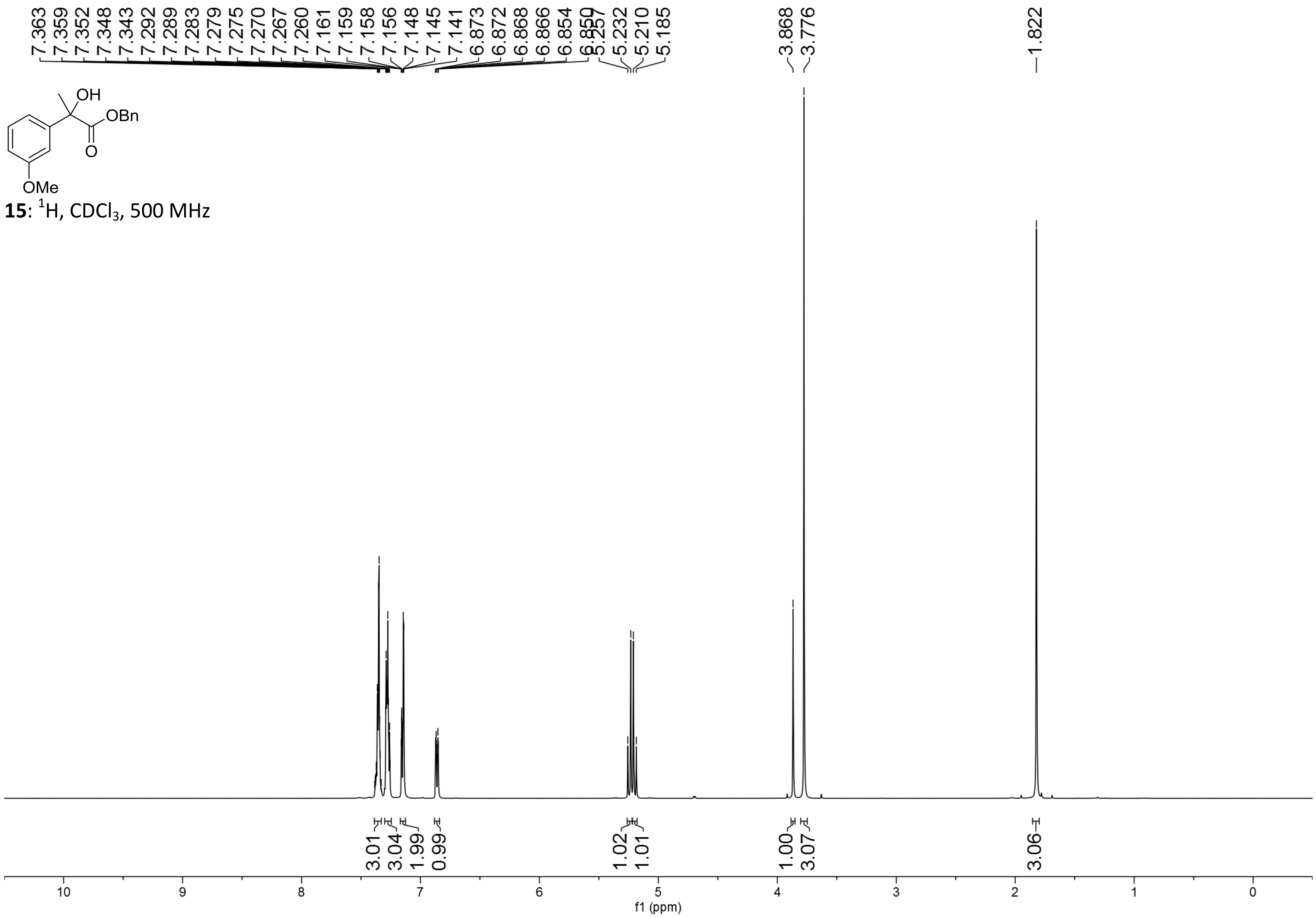

15: ^{1}H, $CDCl_3$, 500 MHz

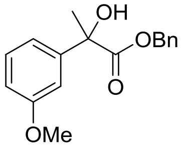

15: ^{13}C, $CDCl_3$, 126 MHz

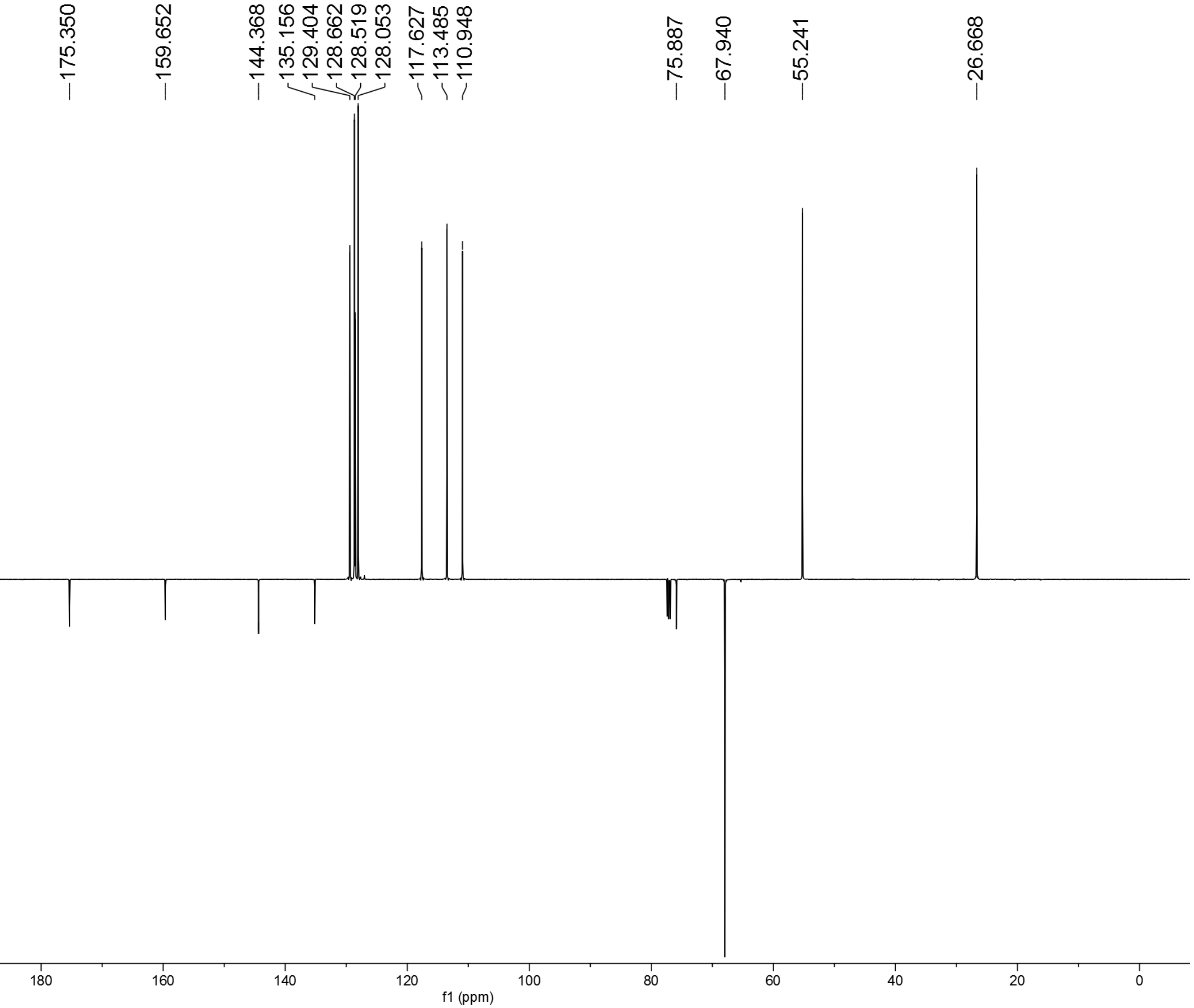

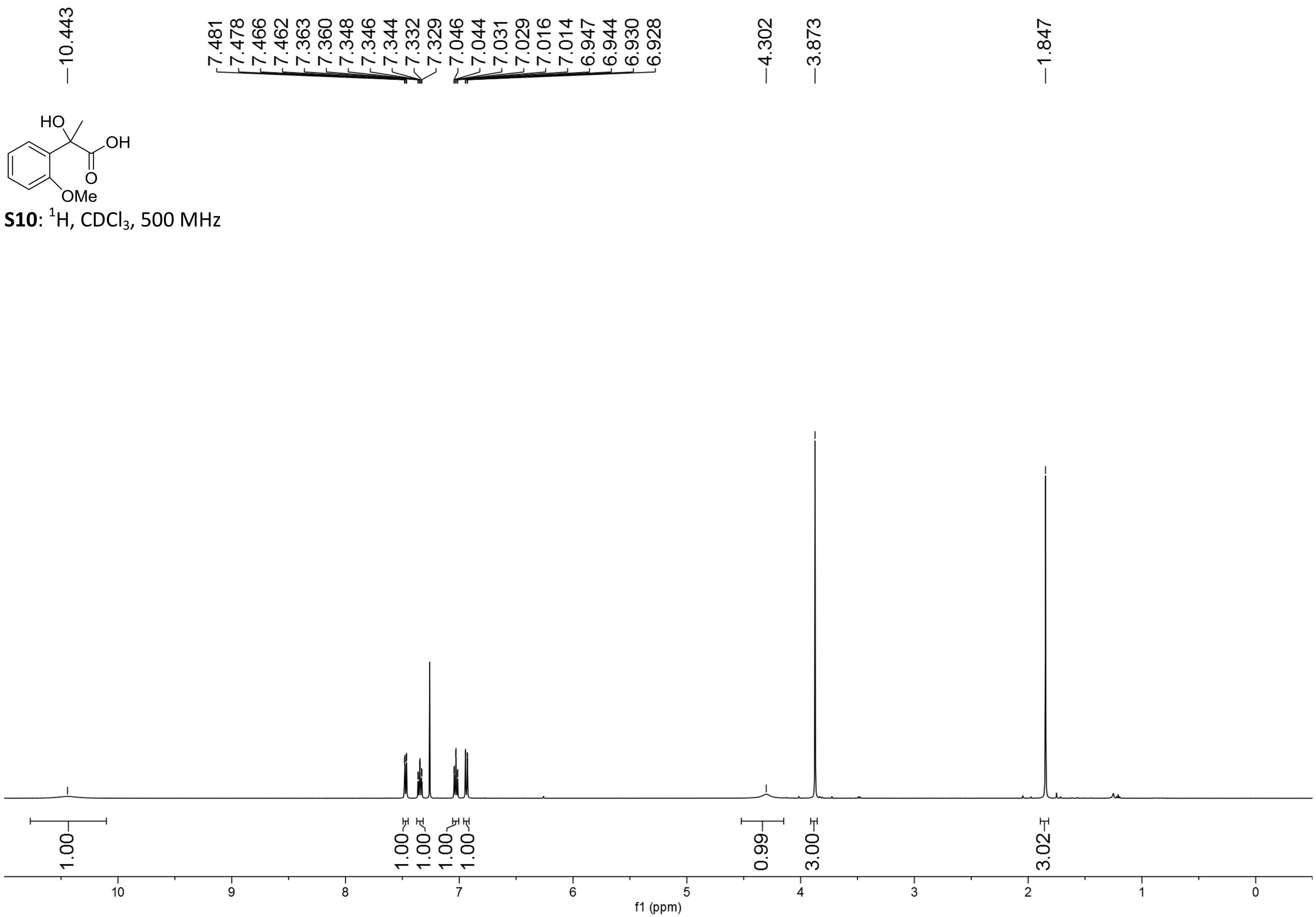

S10: ^{1}H, $CDCl_3$, 500 MHz

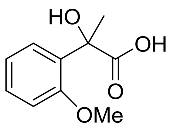

S10: ^{13}C, $CDCl_3$, 126 MHz

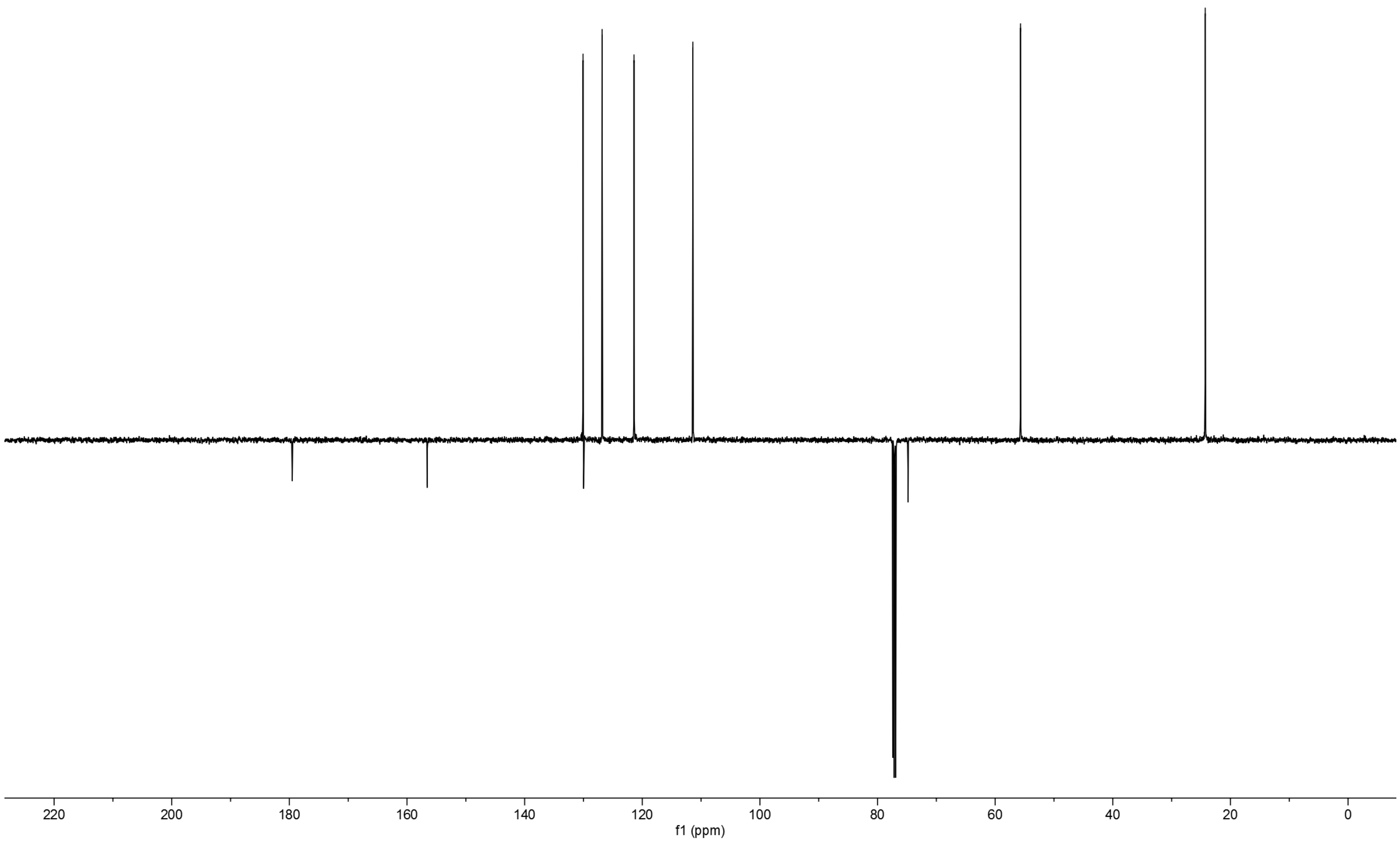

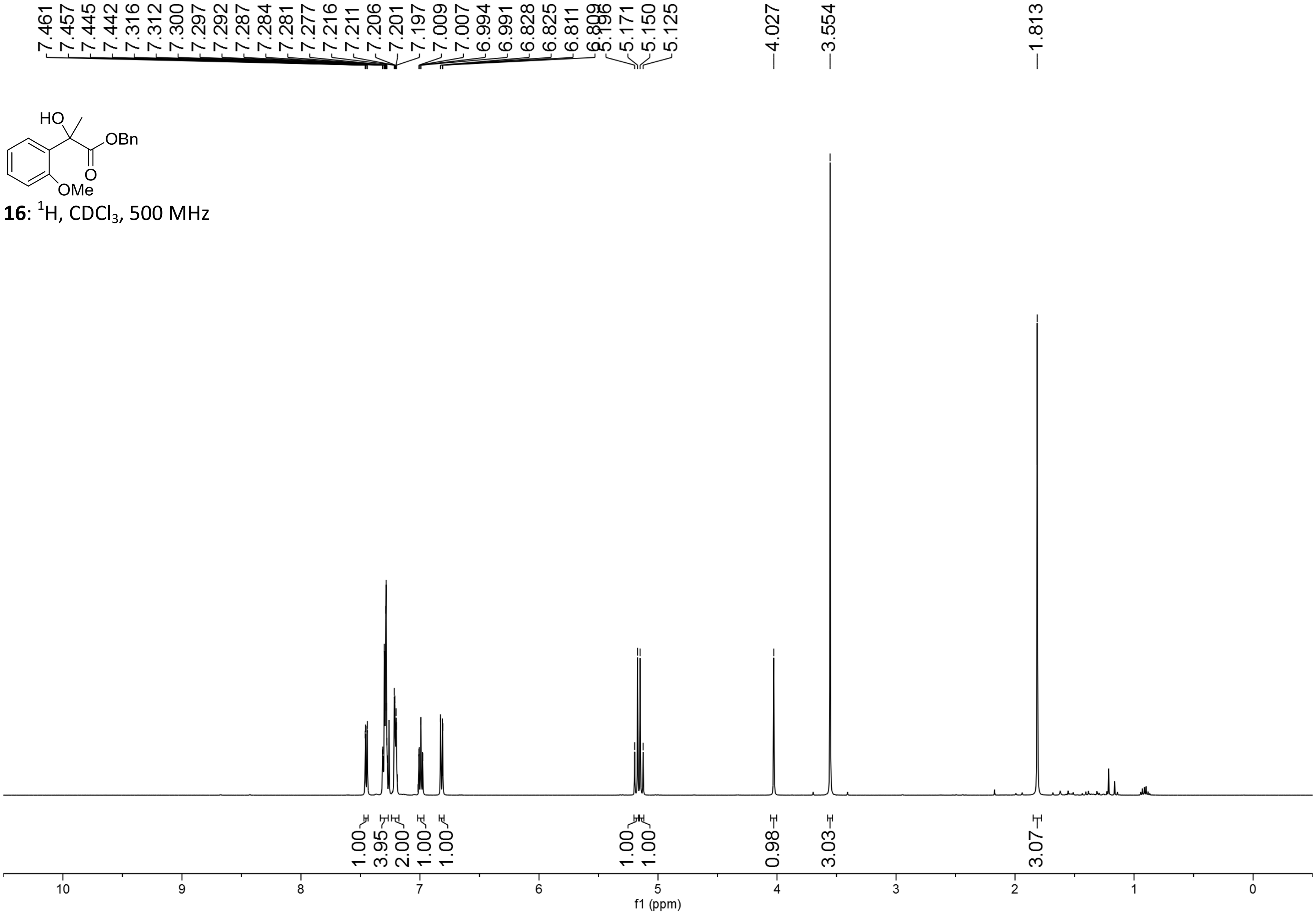

16: ^{1}H, $CDCl_3$, 500 MHz

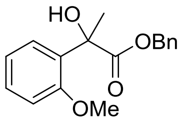

16: ^{13}C, $CDCl_3$, 126 MHz

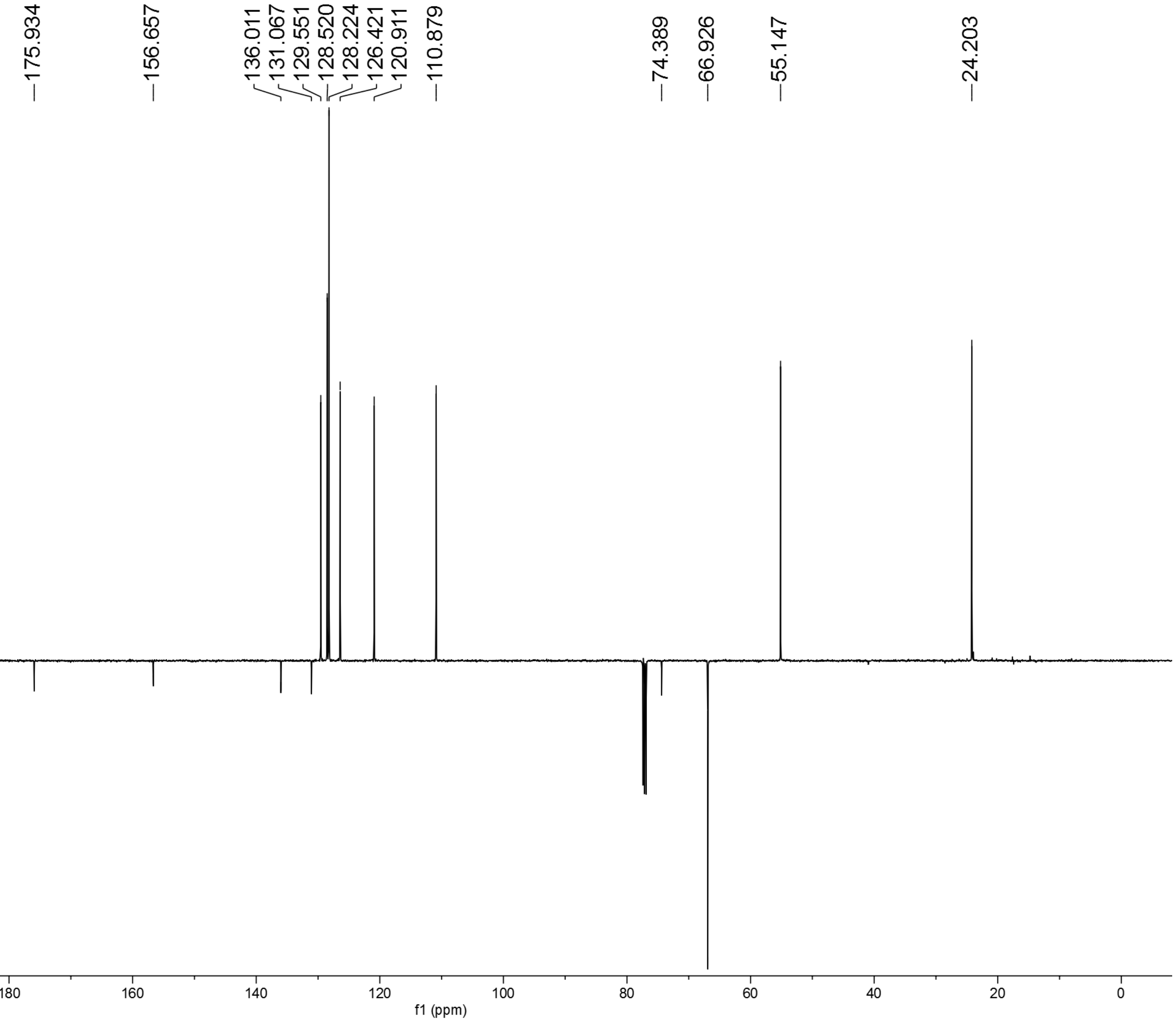

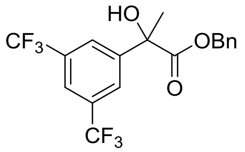

17: ^{1}H, $CDCl_3$, 500 MHz

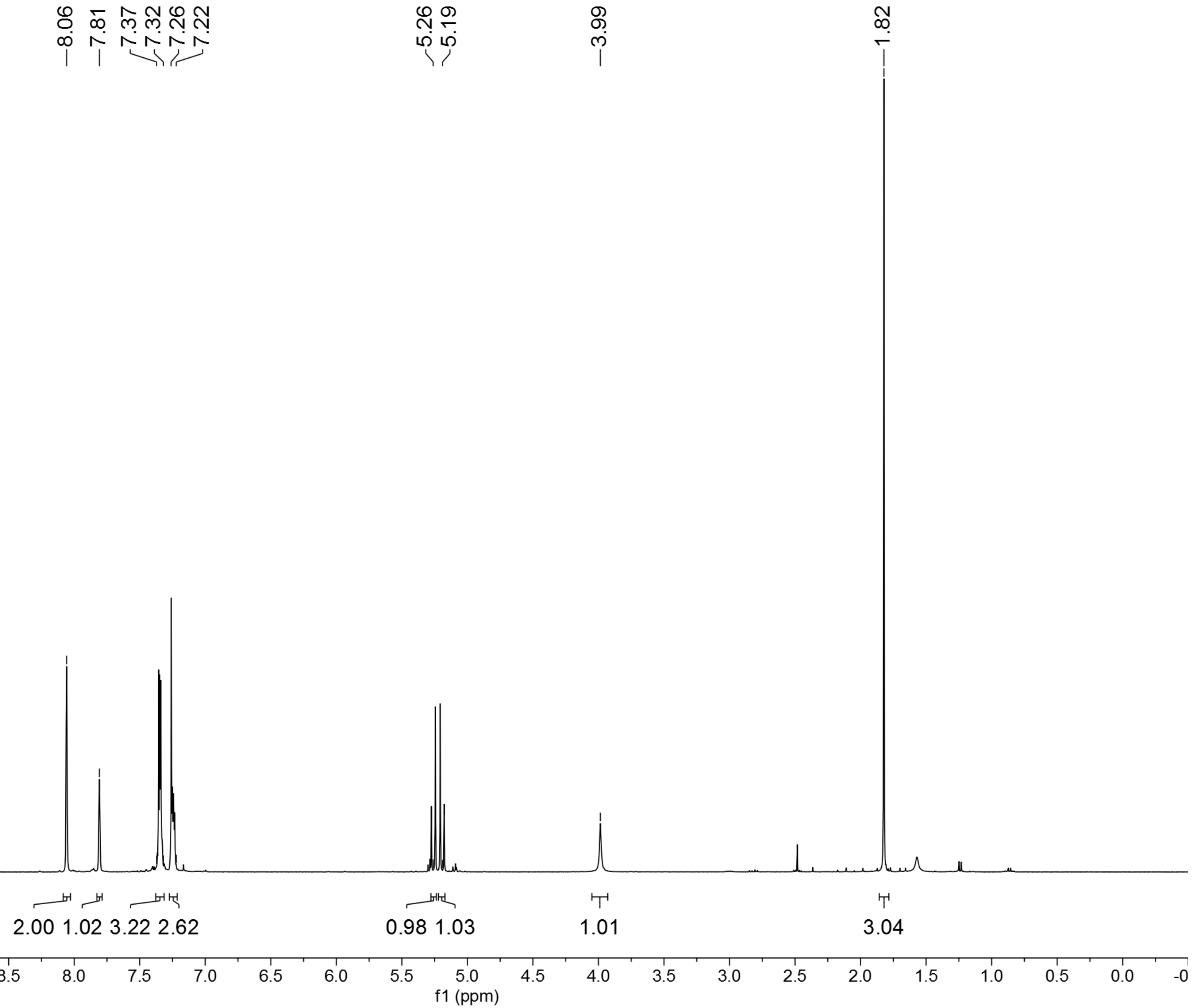

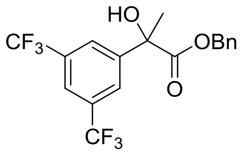

17: ^{13}C, $CDCl_3$, 126 MHz

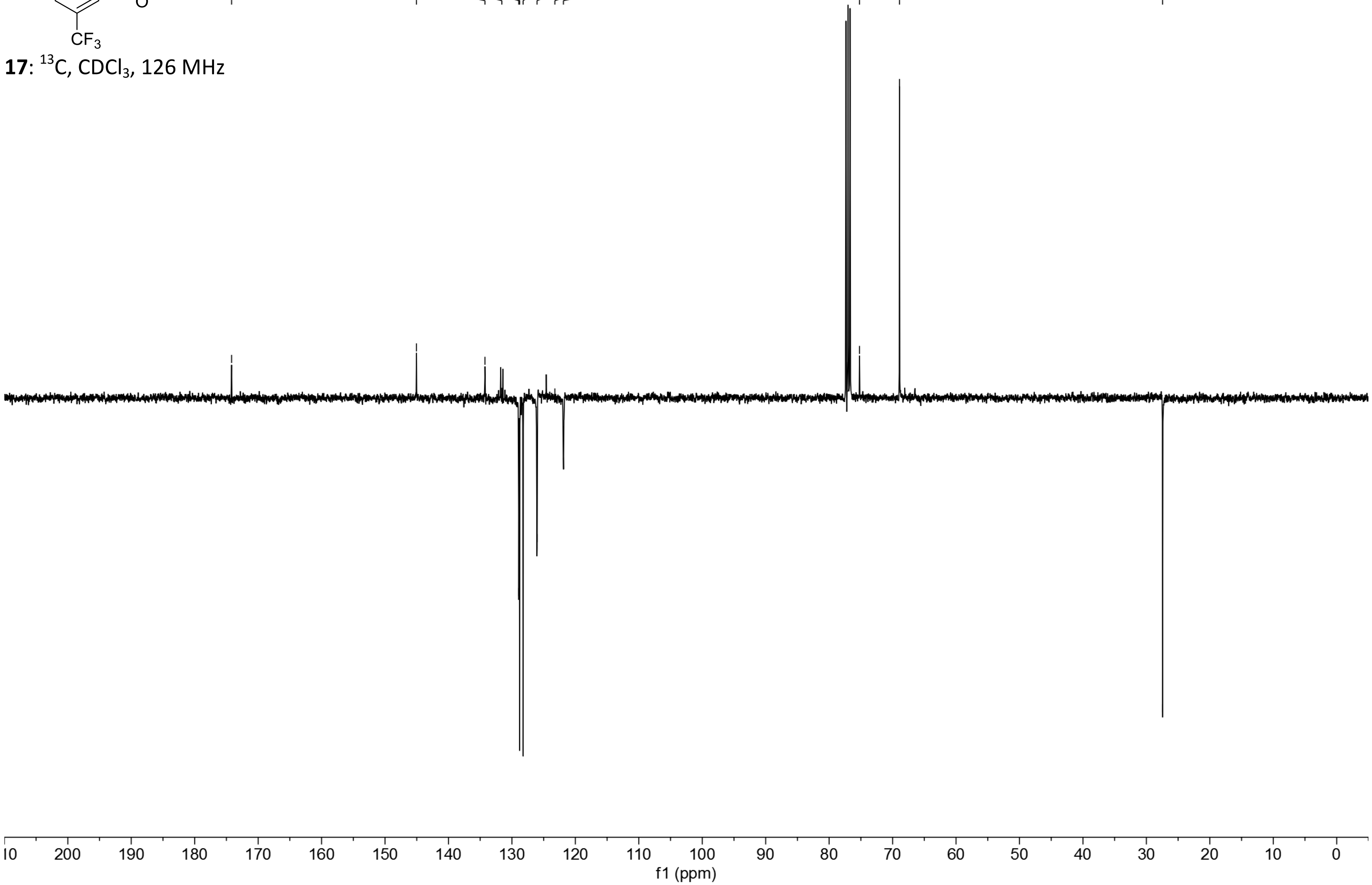

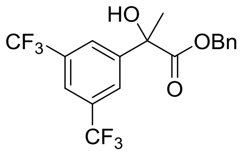

17: ^{19}F, $CDCl_3$, 376 MHz

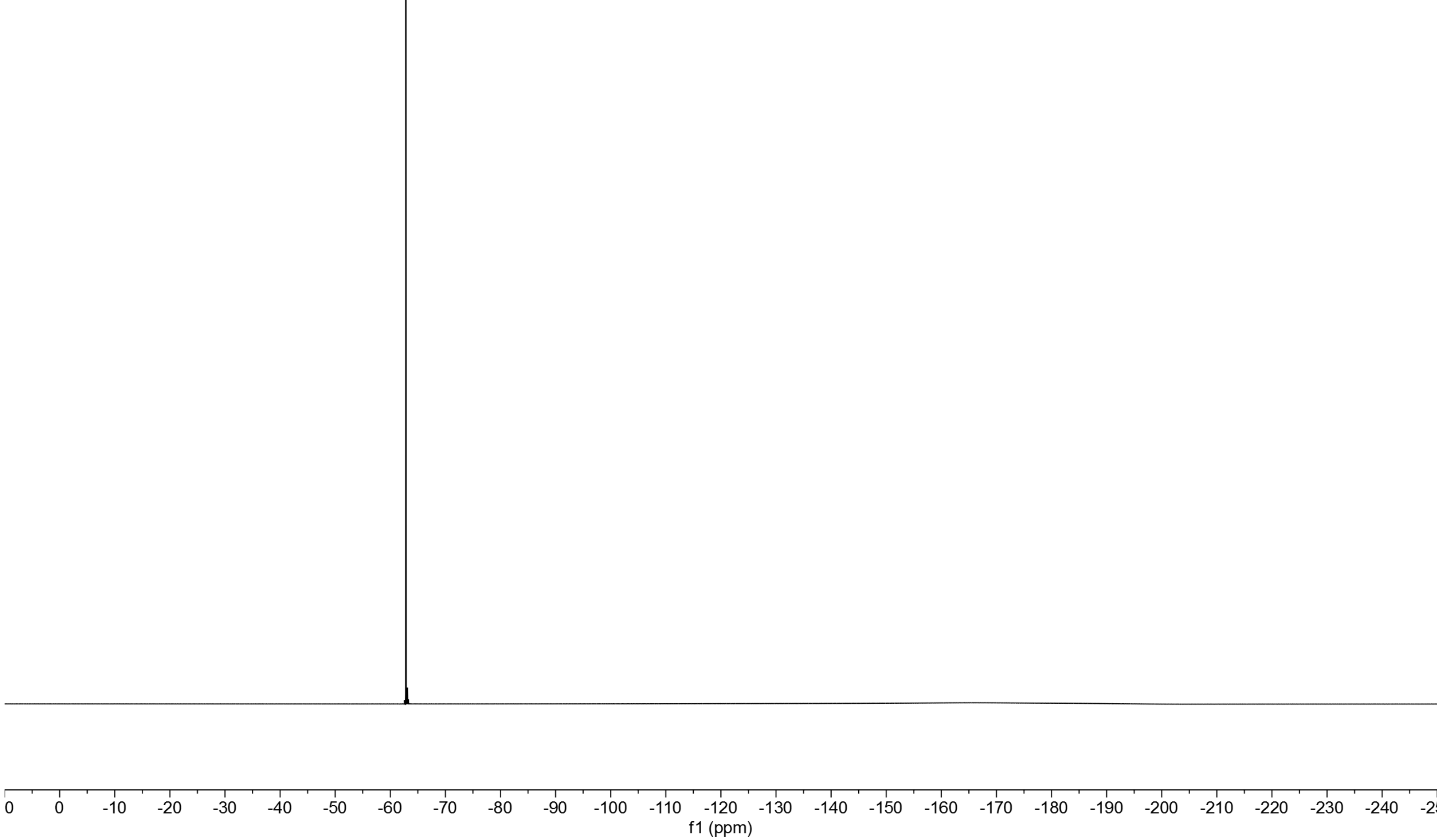

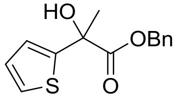

18: ^{1}H, $CDCl_3$, 400 MHz

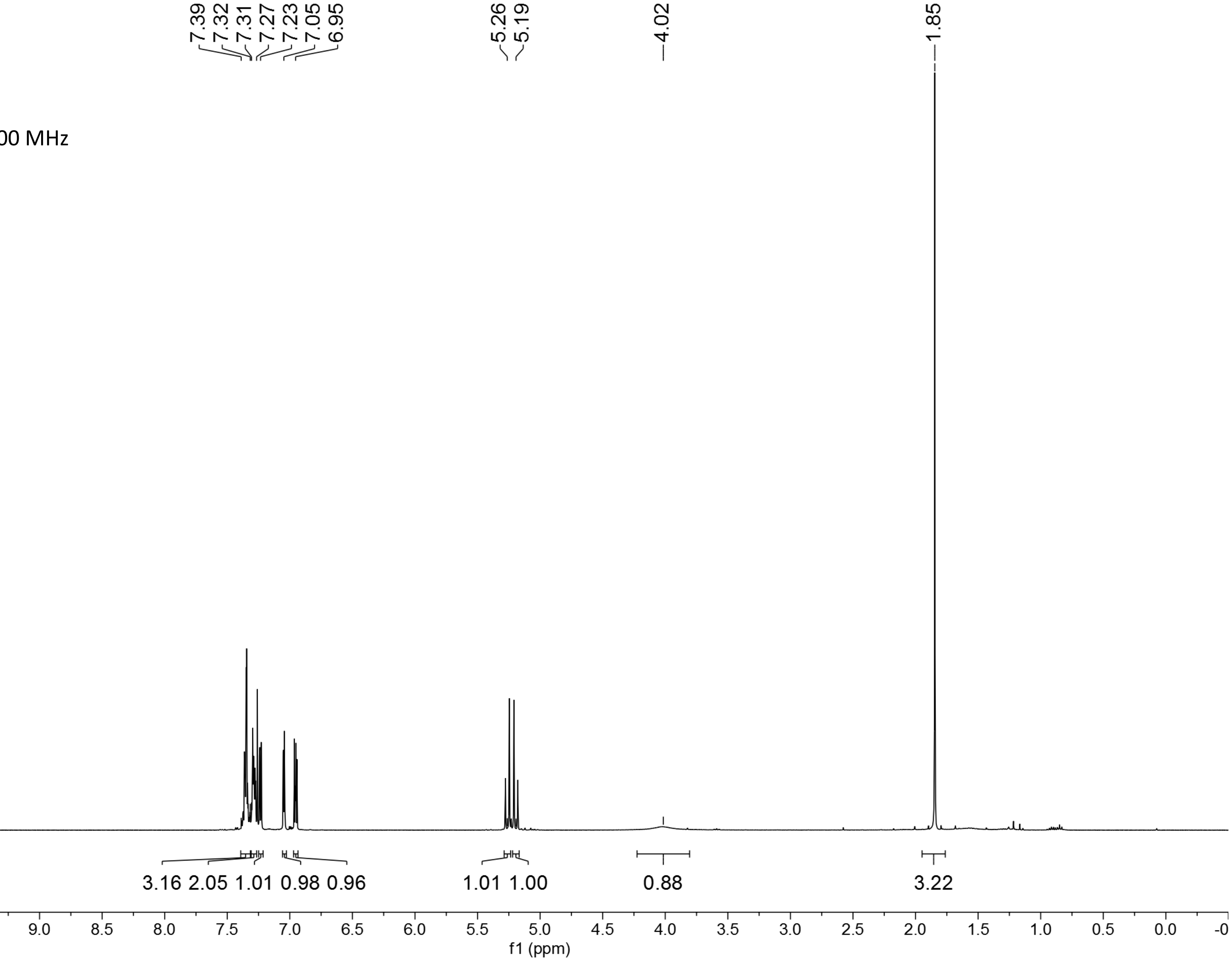

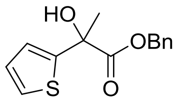

18: ^{13}C, $CDCl_3$, 100 MHz

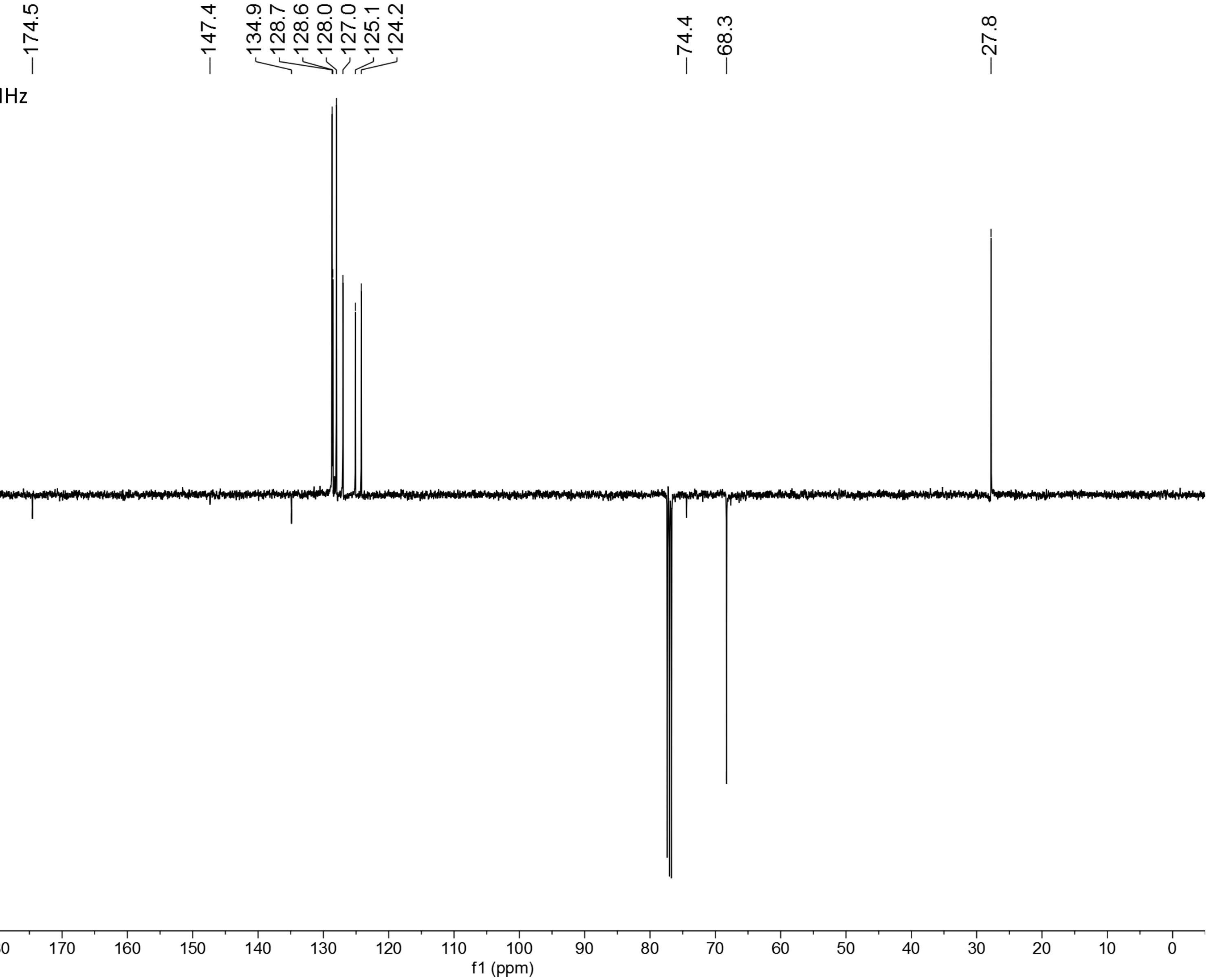

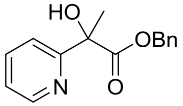

19: ^{1}H, $CDCl_3$, 500 MHz

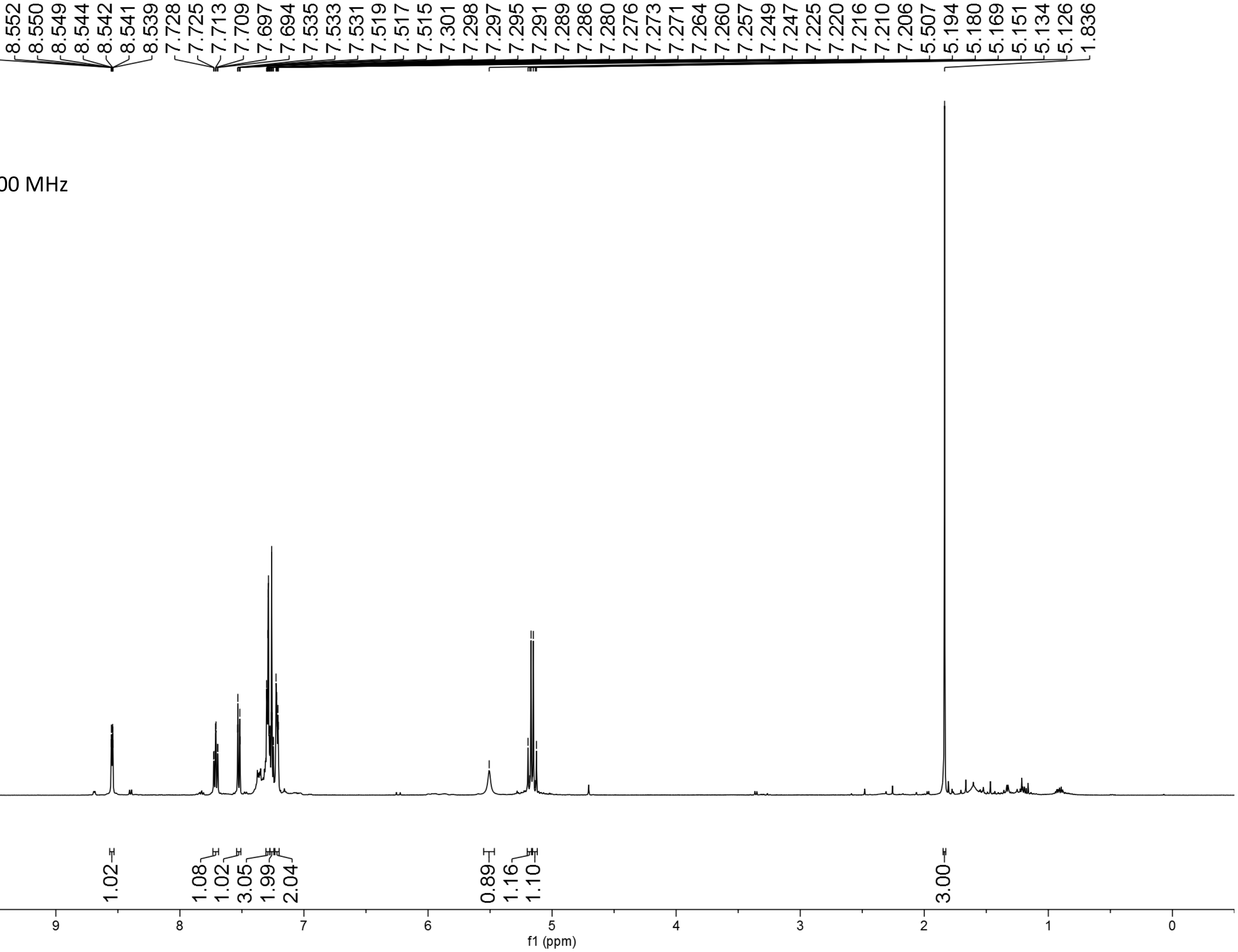

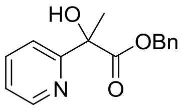

19: ^{13}C, $CDCl_3$, 126 MHz

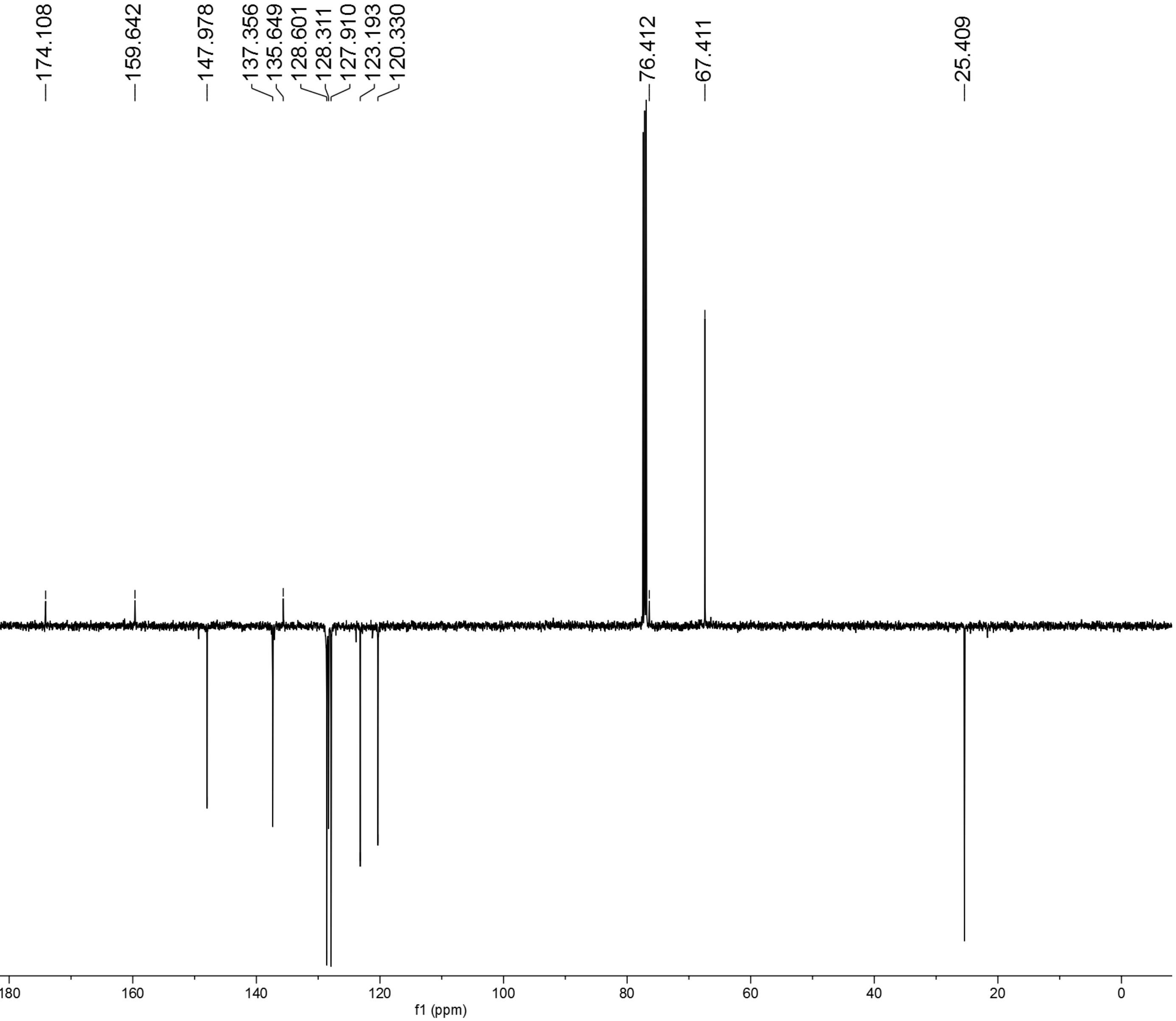

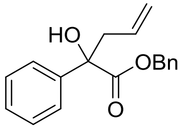

20: ^1H, $CDCl_3$, 400 MHz

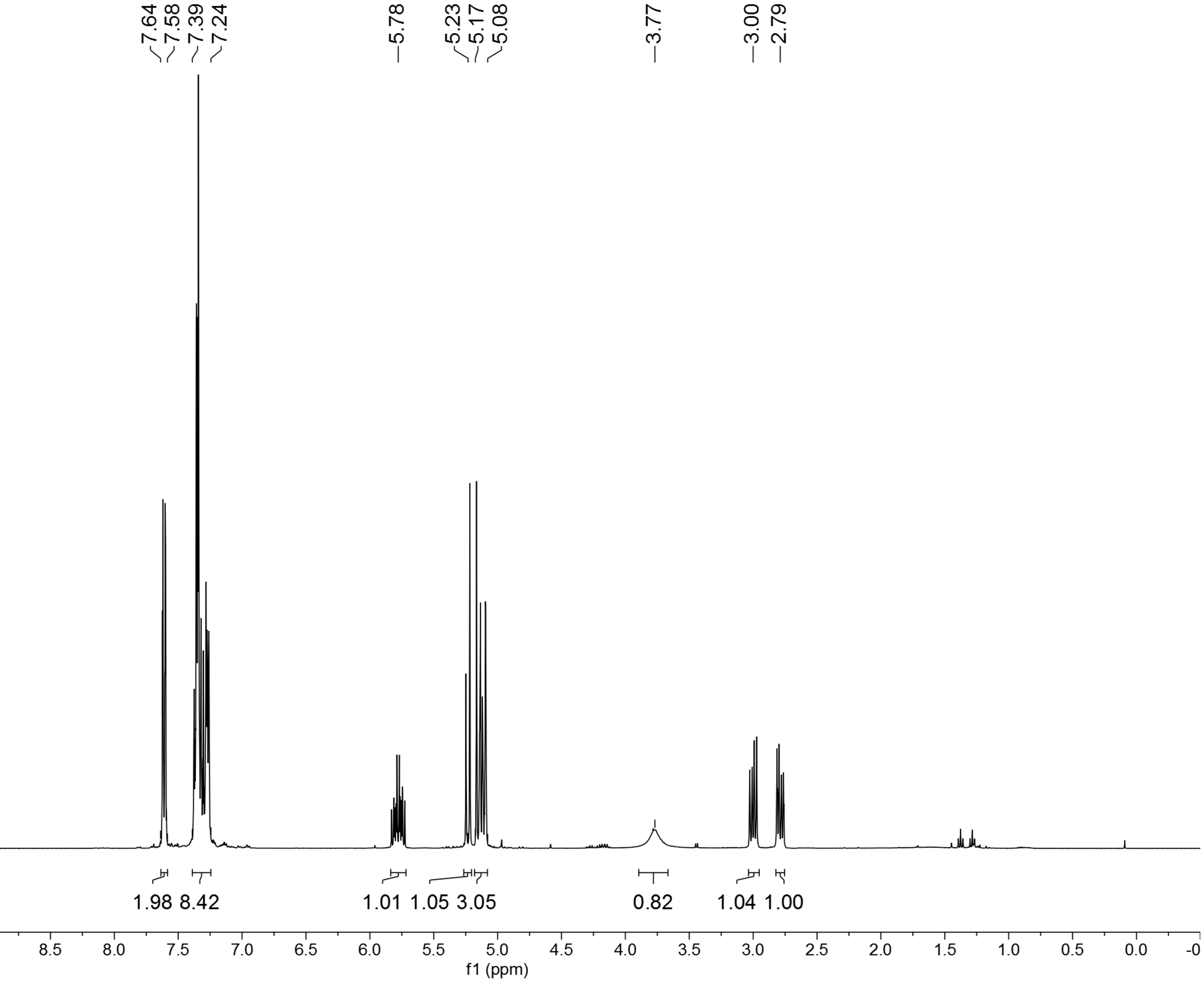

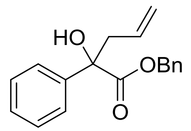

20: ^{13}C, $CDCl_3$, 100 MHz

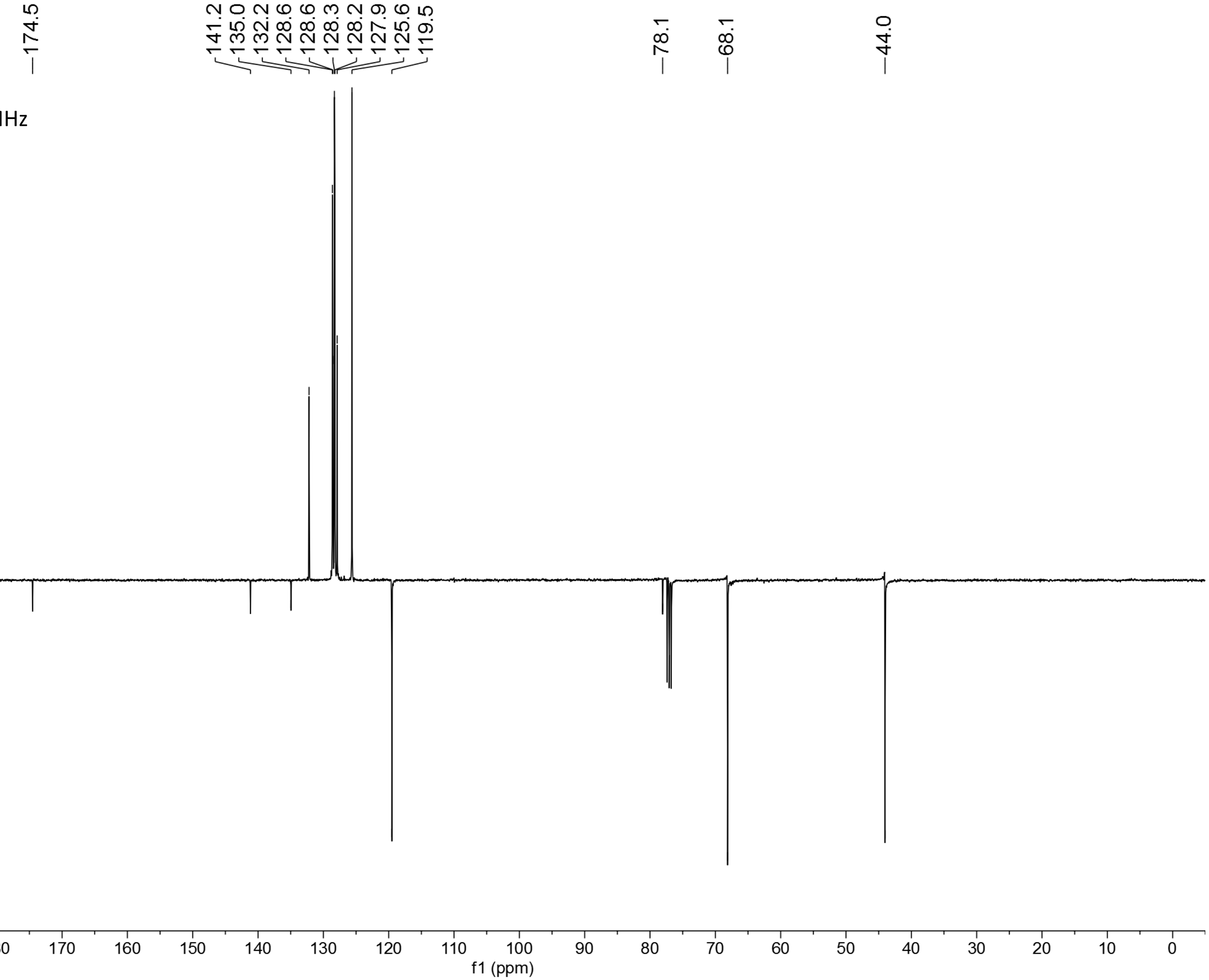

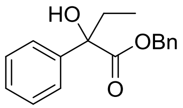

21: ^{1}H, $CDCl_3$, 400 MHz

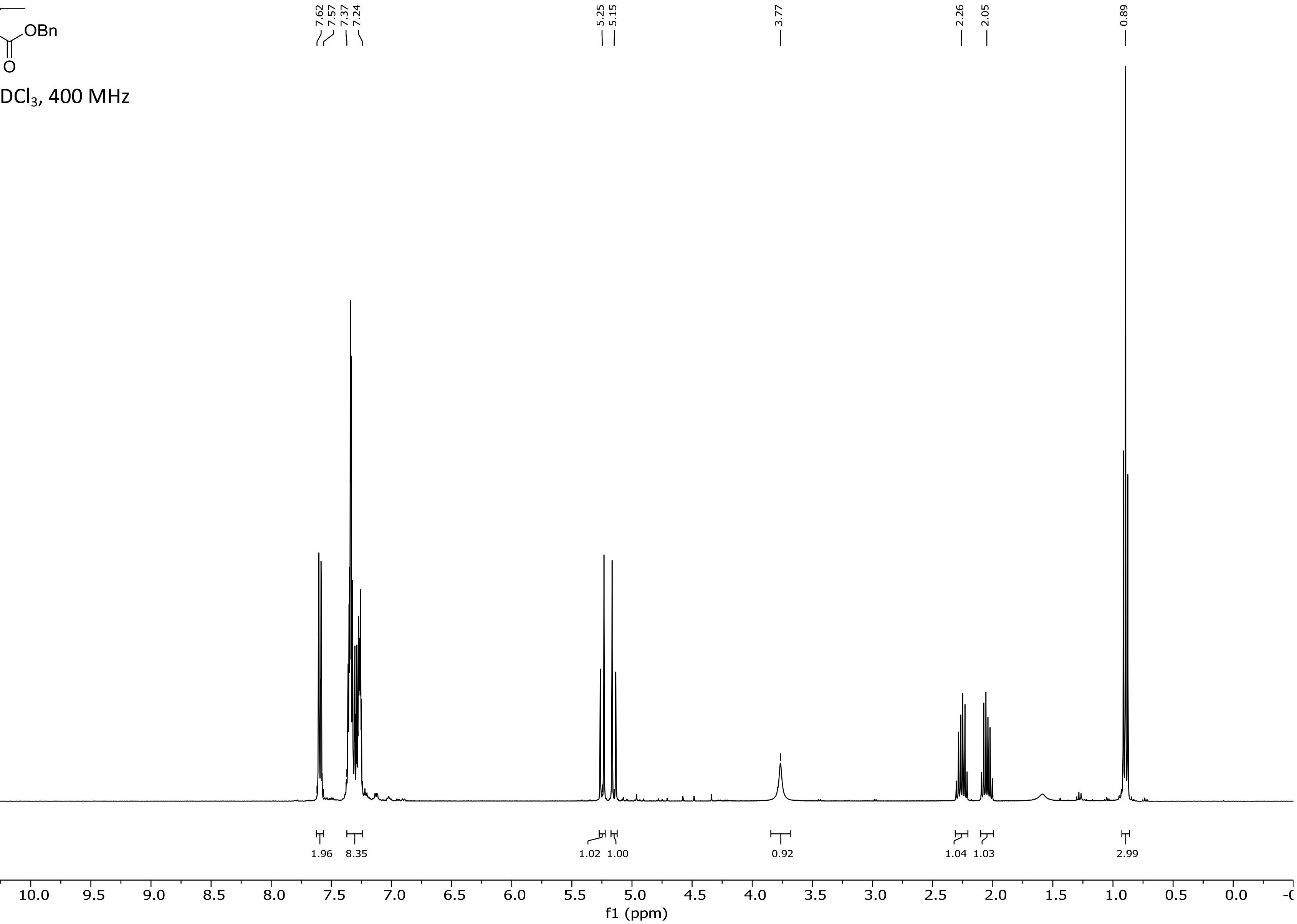

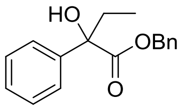

21: ^{13}C, $CDCl_3$, 100 MHz

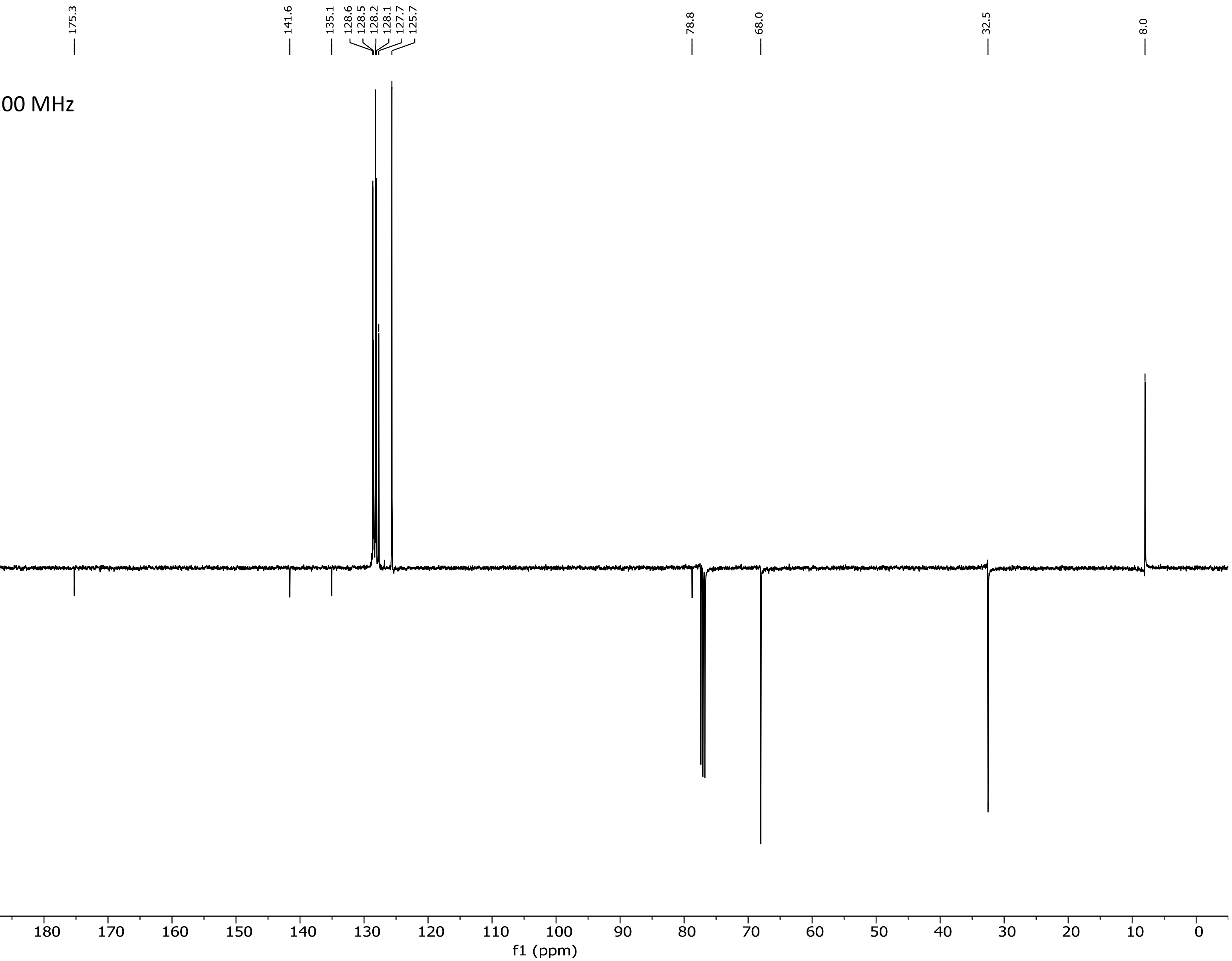

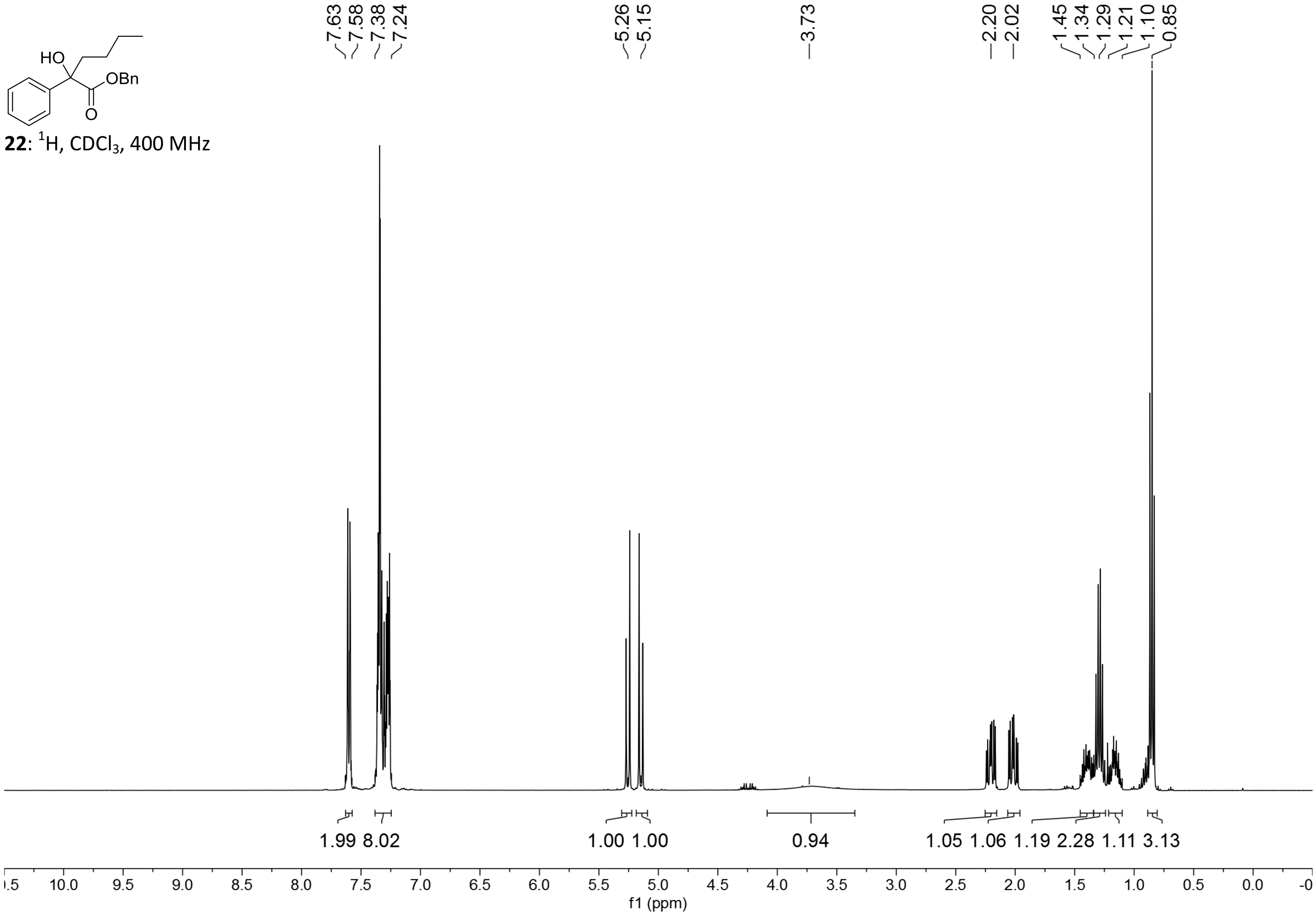

22: ^{1}H, $CDCl_3$, 400 MHz

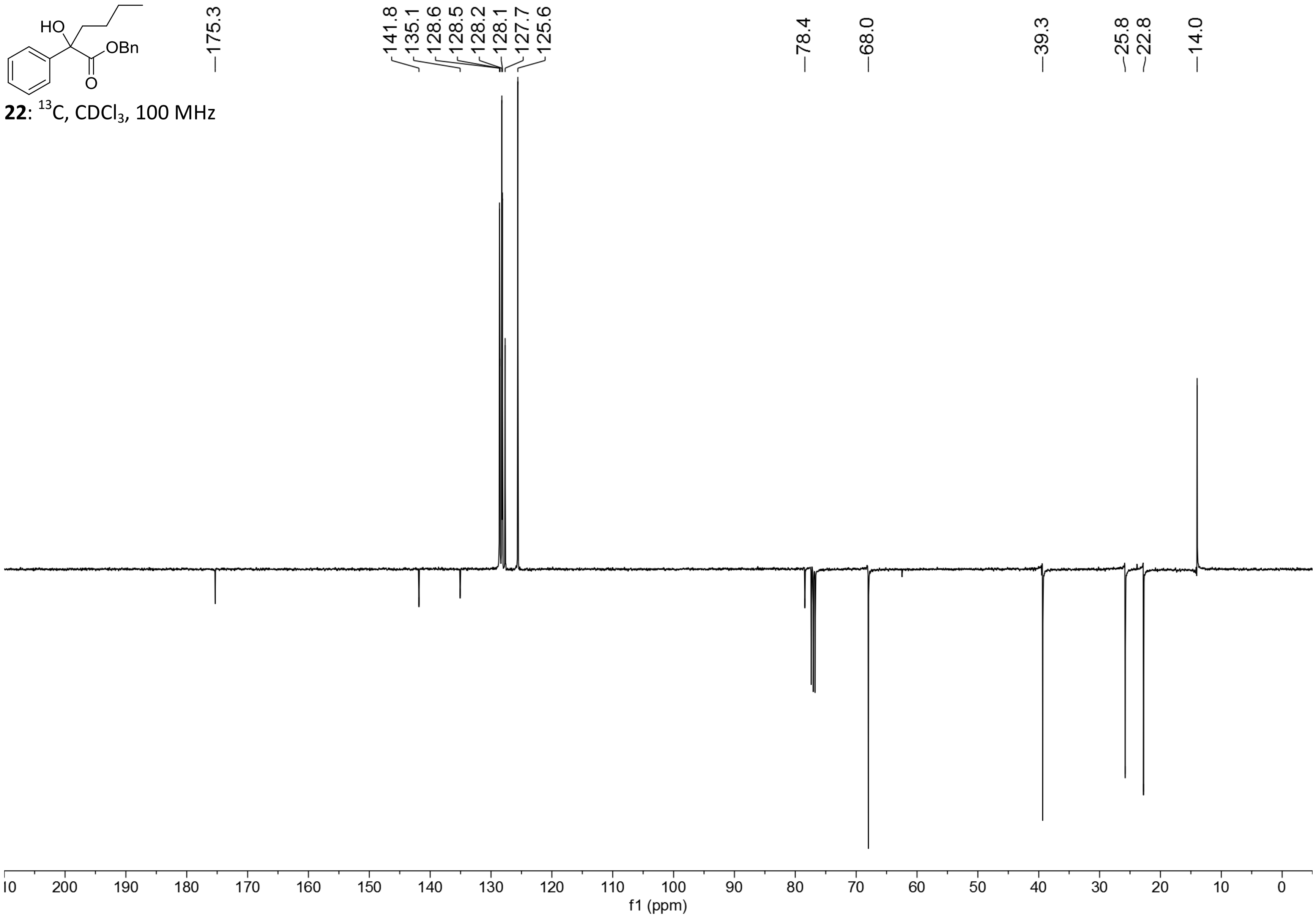

22: ^{13}C, $CDCl_3$, 100 MHz

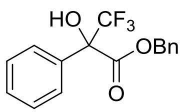

23: ^{1}H, $CDCl_3$, 400 MHz

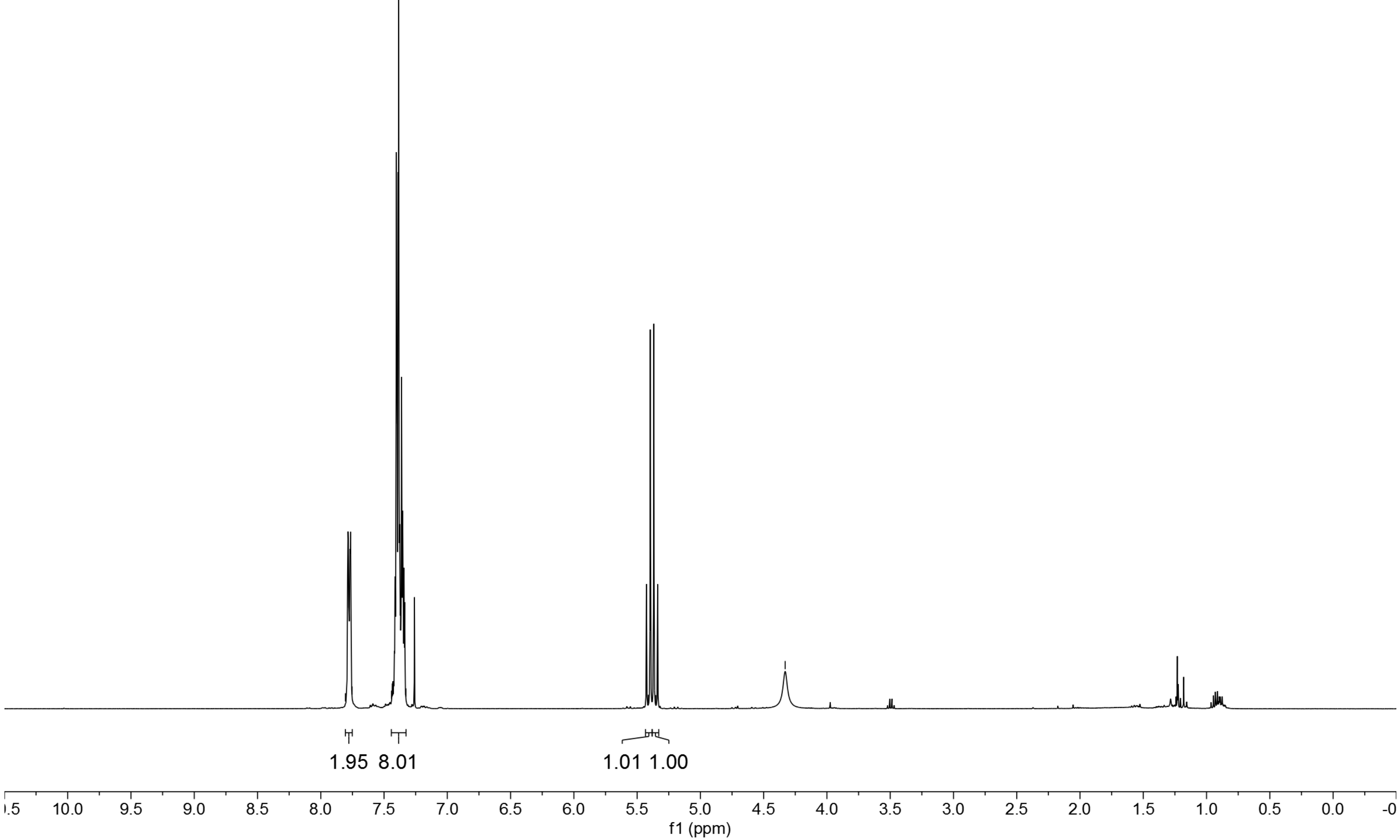

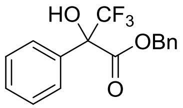

23: ^{13}C, $CDCl_3$, 100 MHz

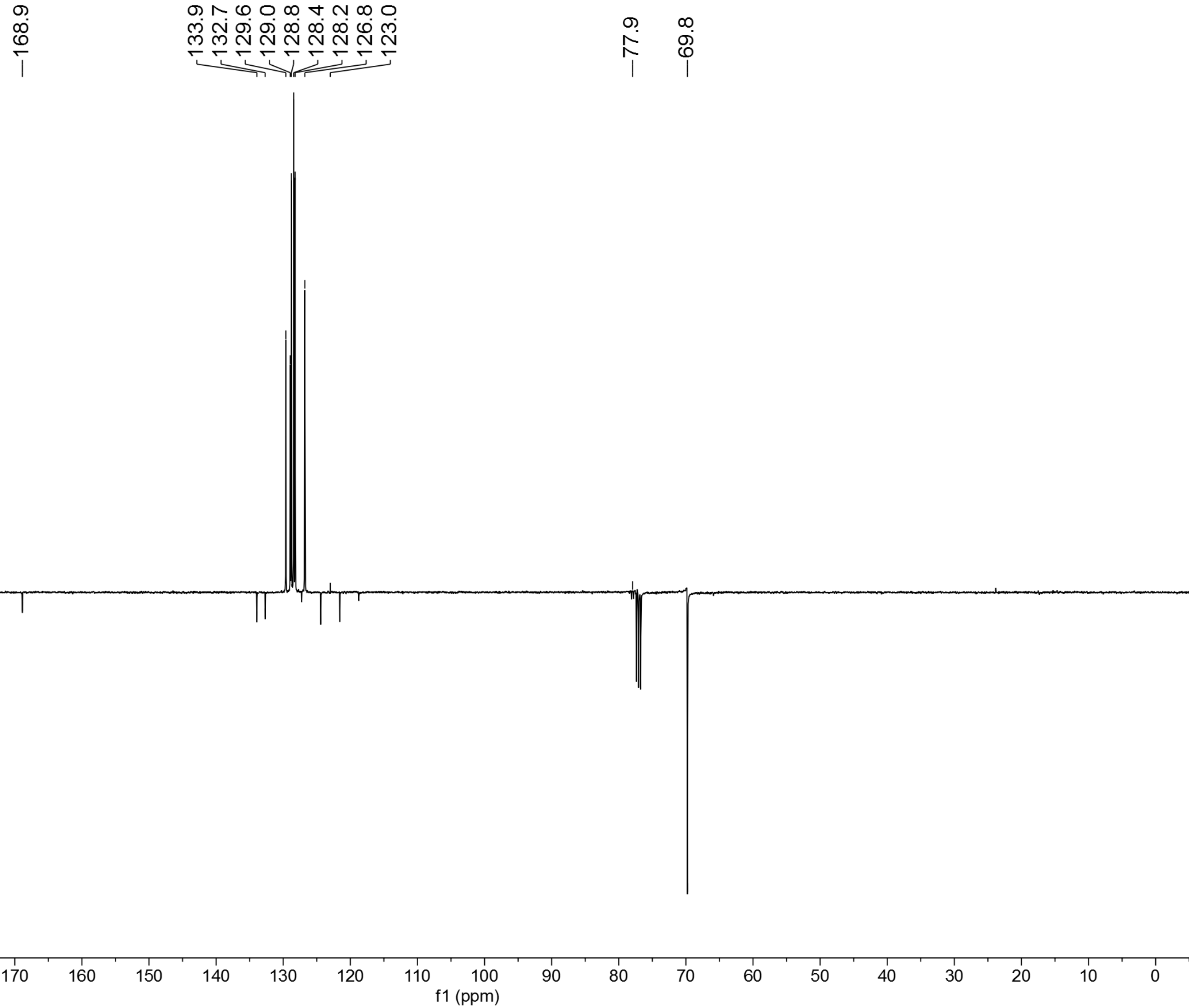

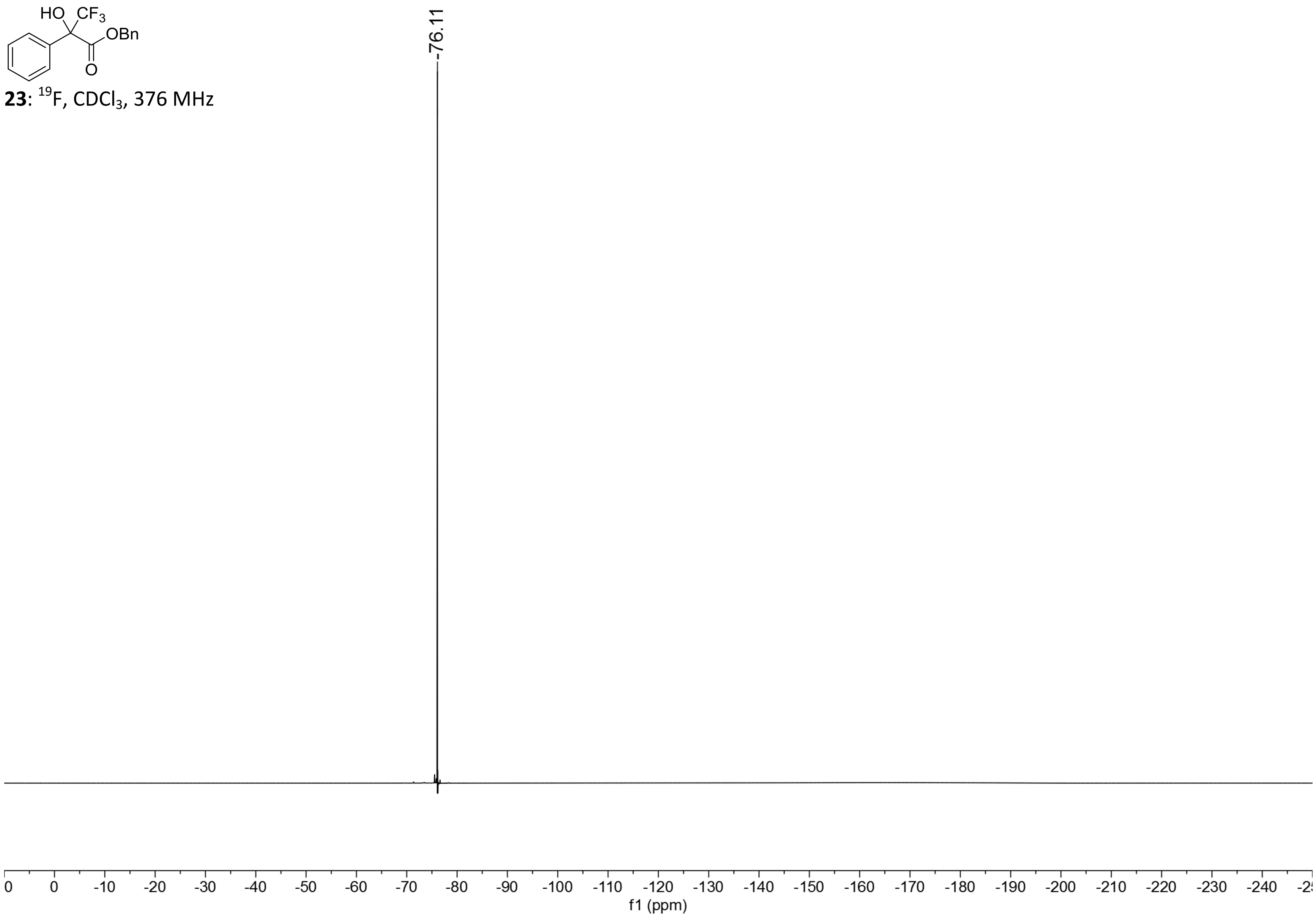

23: ^{19}F, $CDCl_3$, 376 MHz

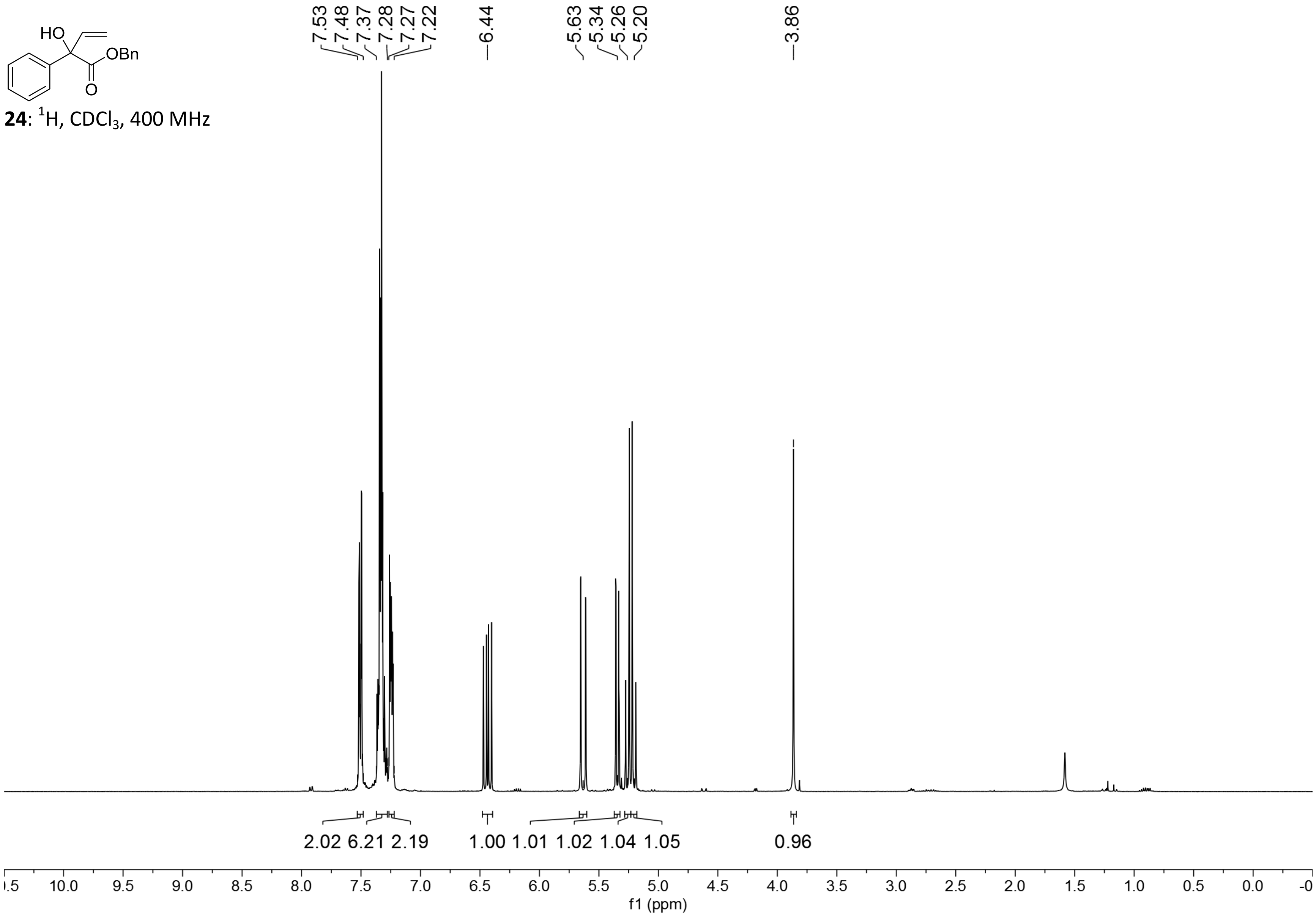

24: ^{1}H, $CDCl_3$, 400 MHz

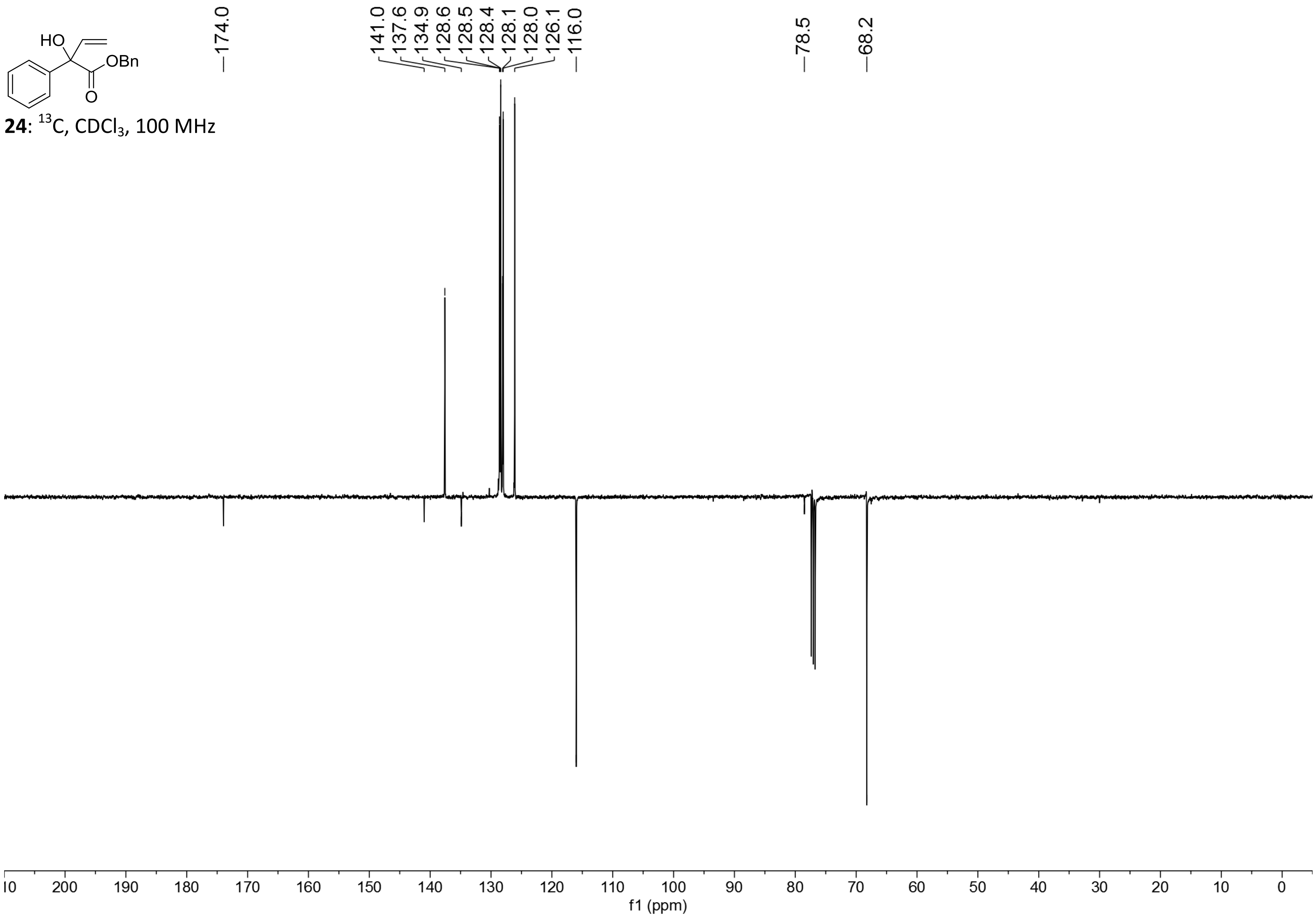

24: ^{13}C, $CDCl_3$, 100 MHz

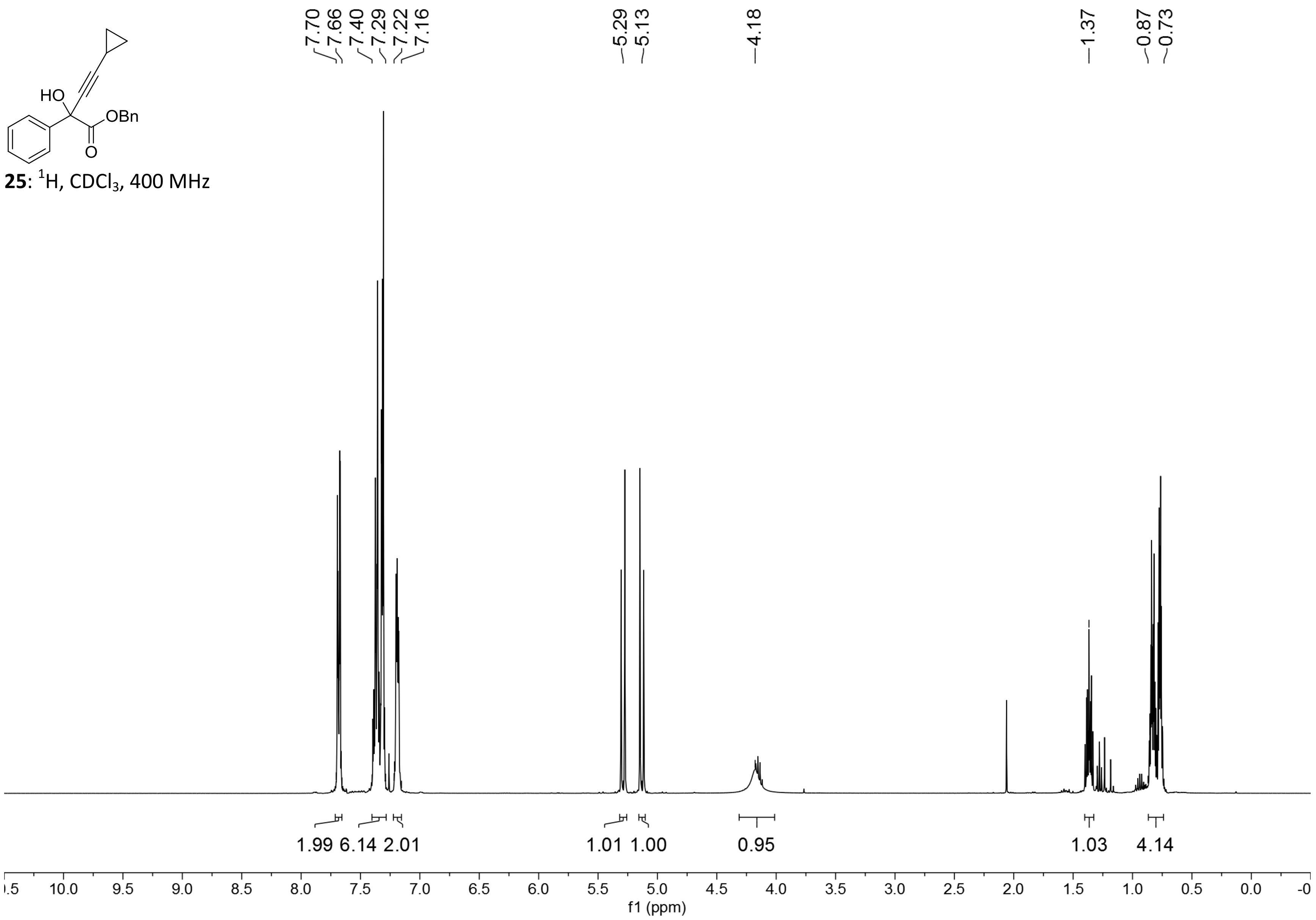

25: ^{1}H, $CDCl_3$, 400 MHz

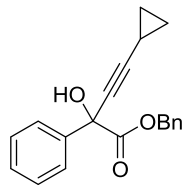

25: ^{13}C, $CDCl_3$, 100 MHz

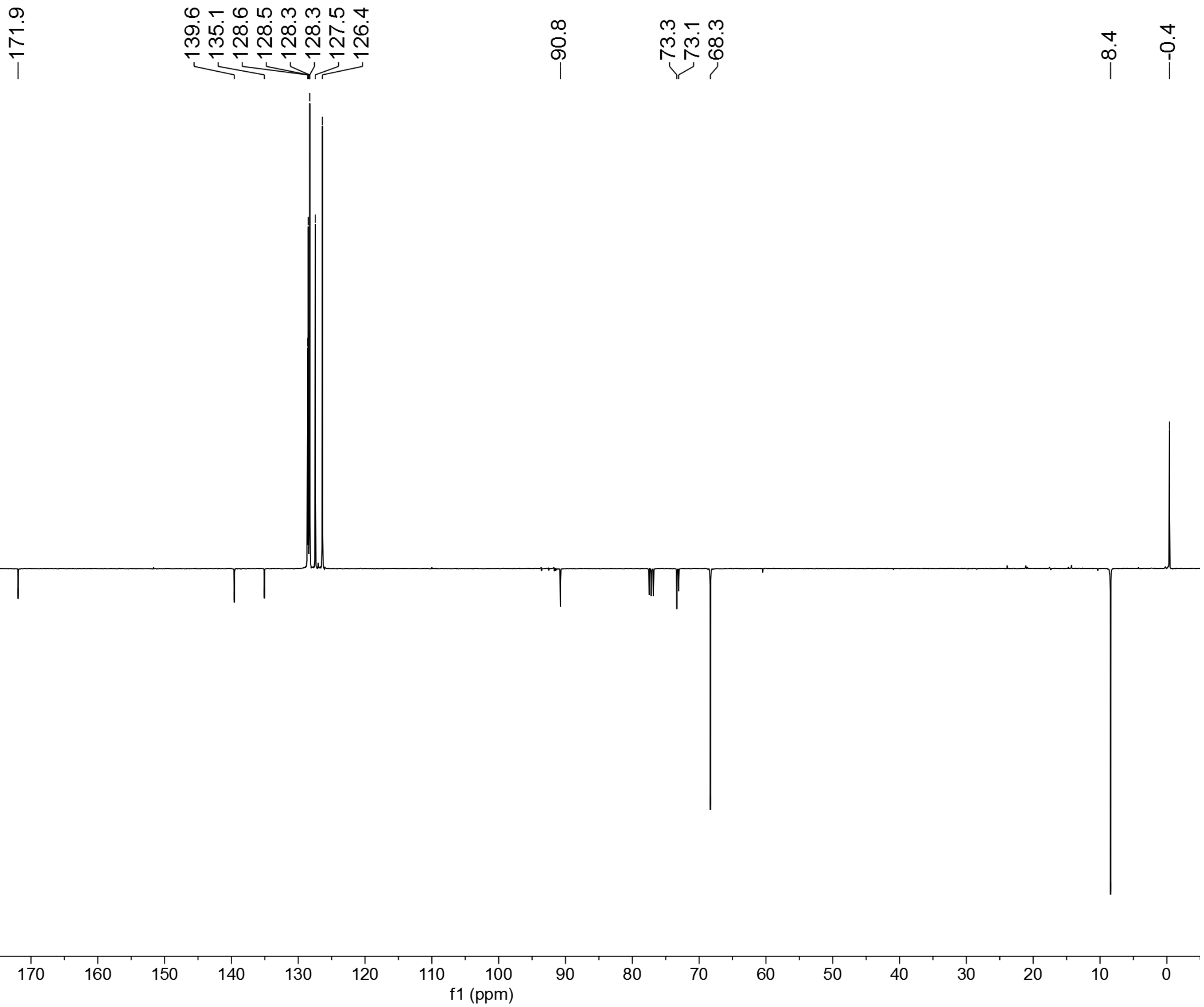

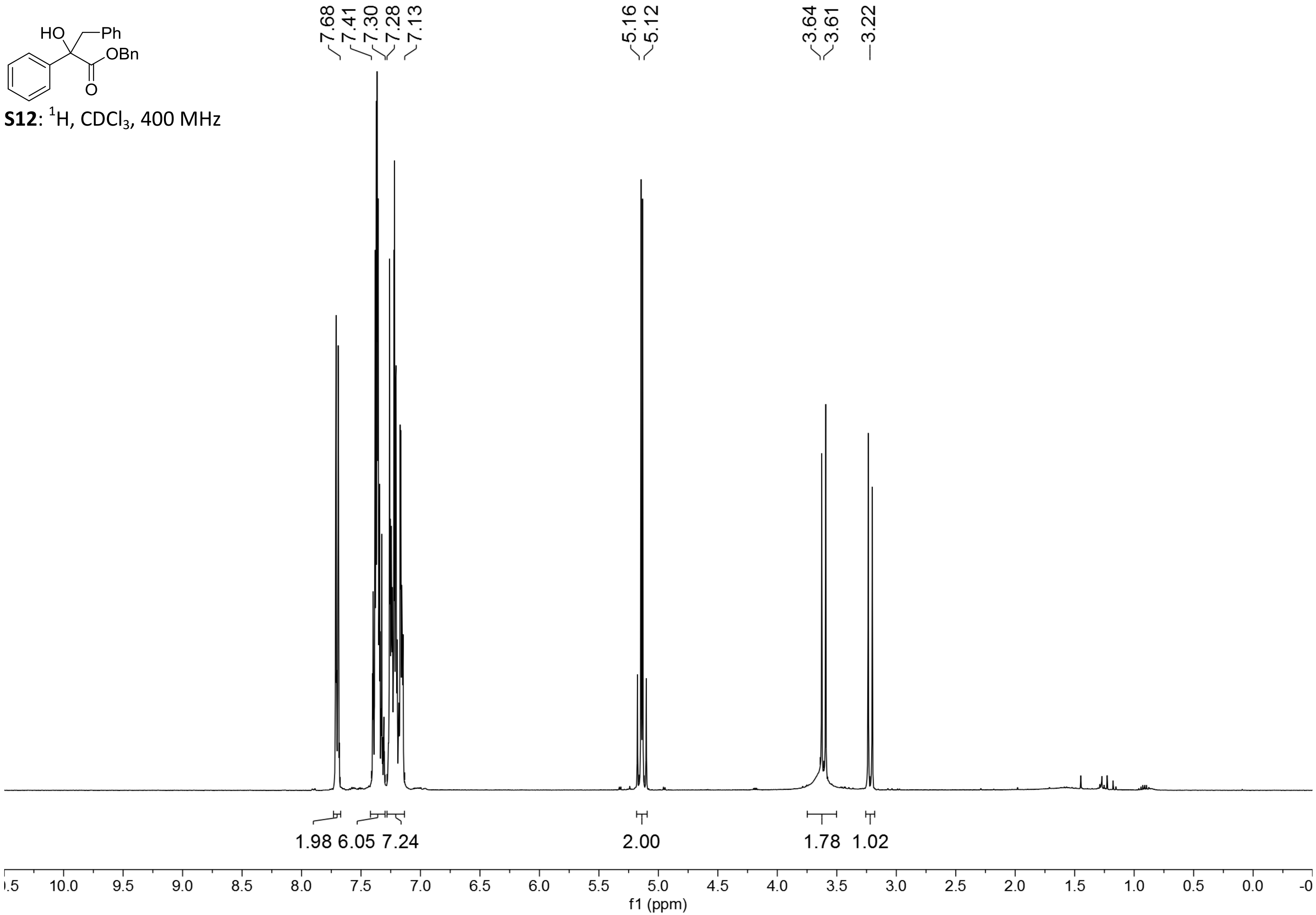

S12: ^{1}H, $CDCl_3$, 400 MHz

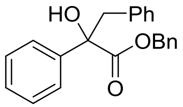

S12: ^{13}C, $CDCl_3$, 100 MHz

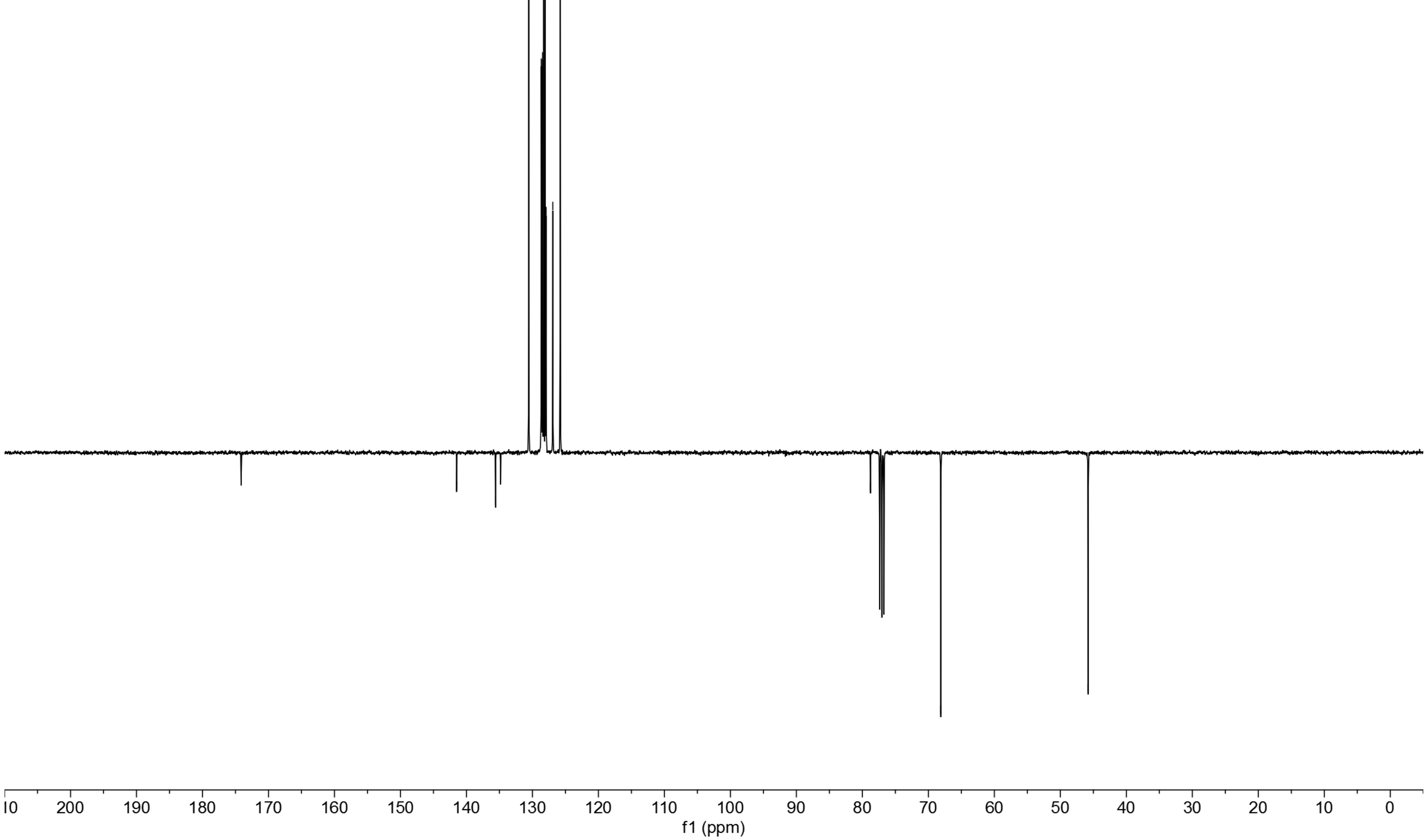

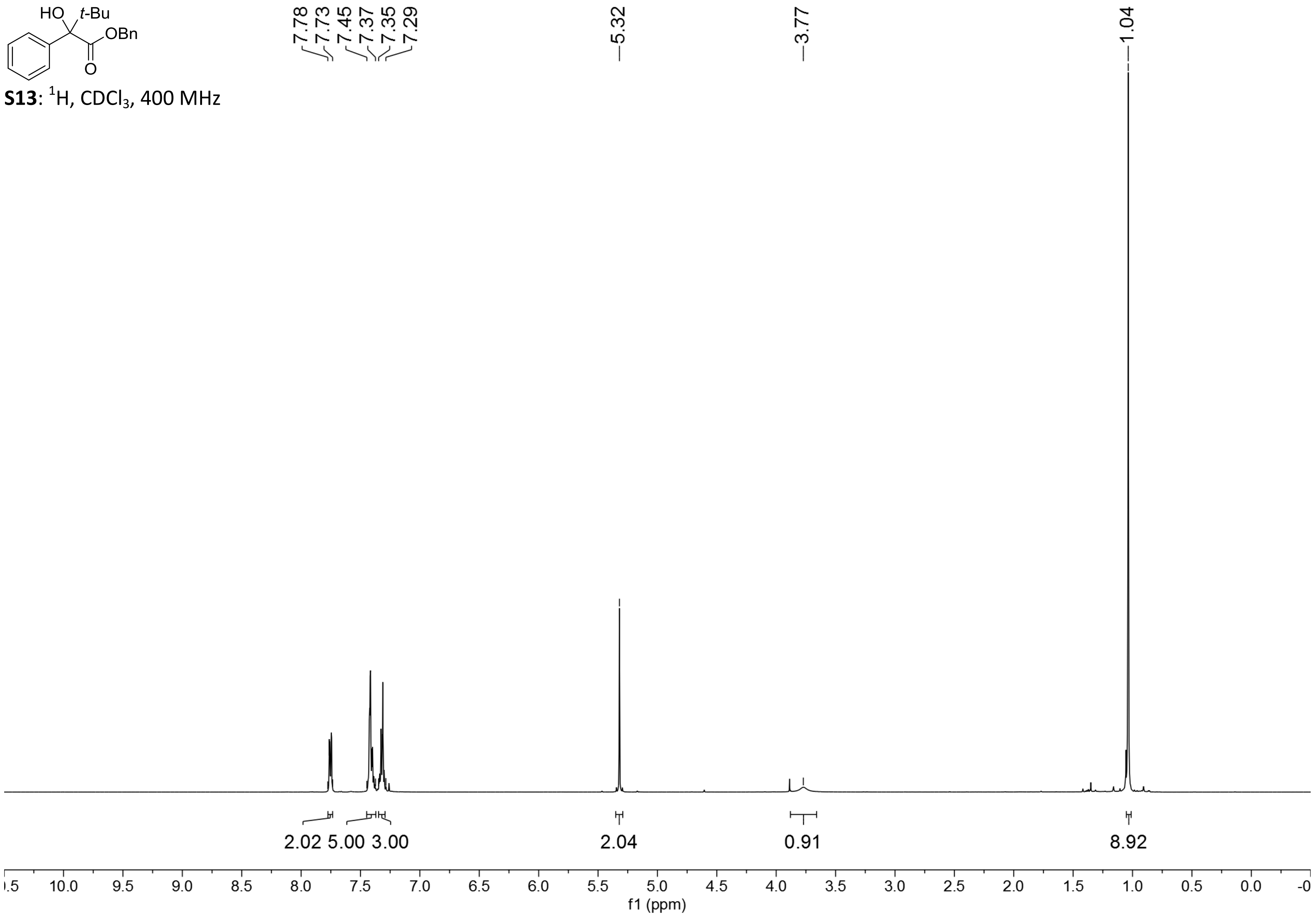

S13: ^{1}H, $CDCl_3$, 400 MHz

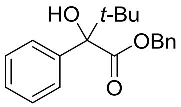

S13: ^{13}C, $CDCl_3$, 100 MHz

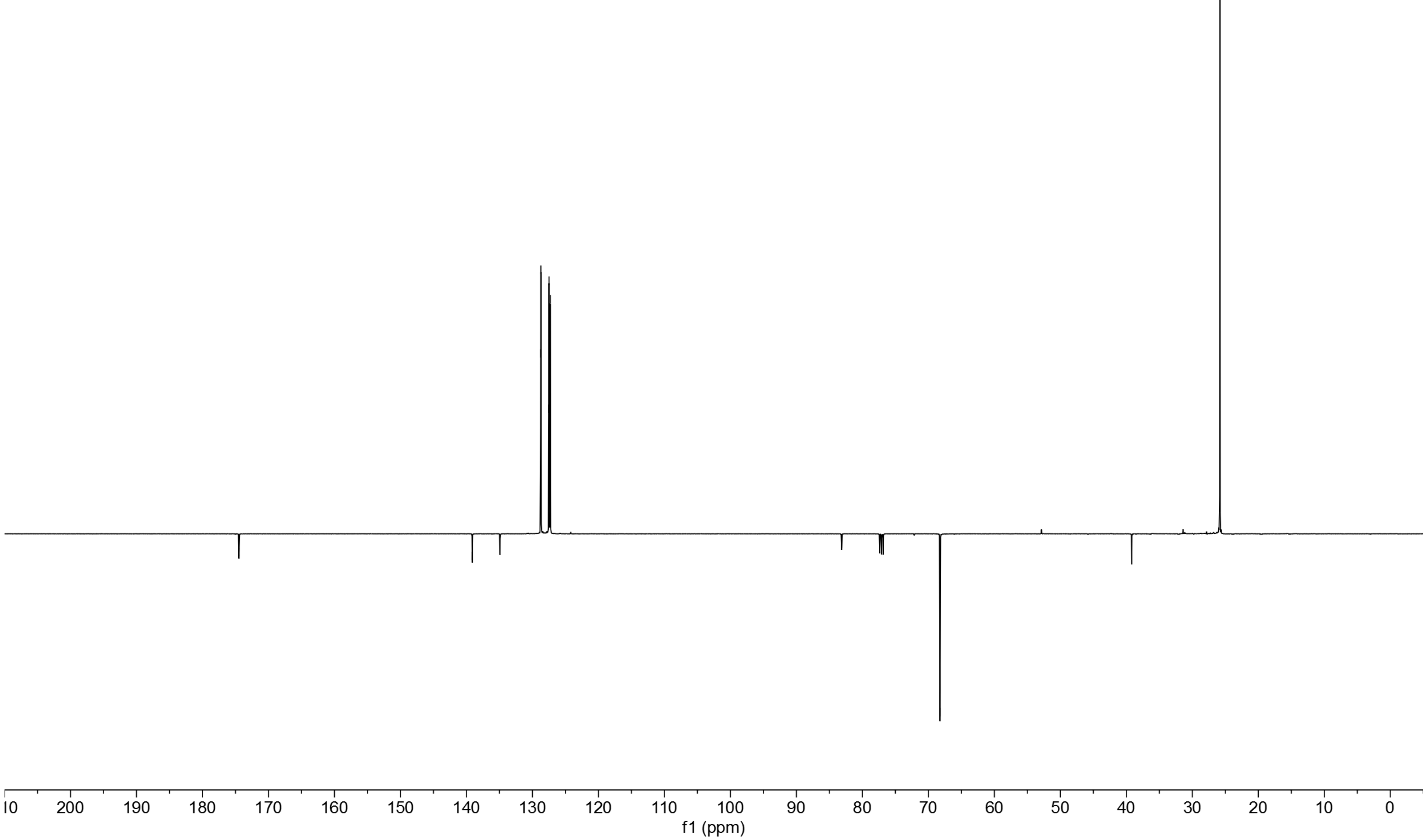

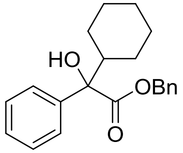

S14: ^{1}H, $CDCl_3$, 500 MHz

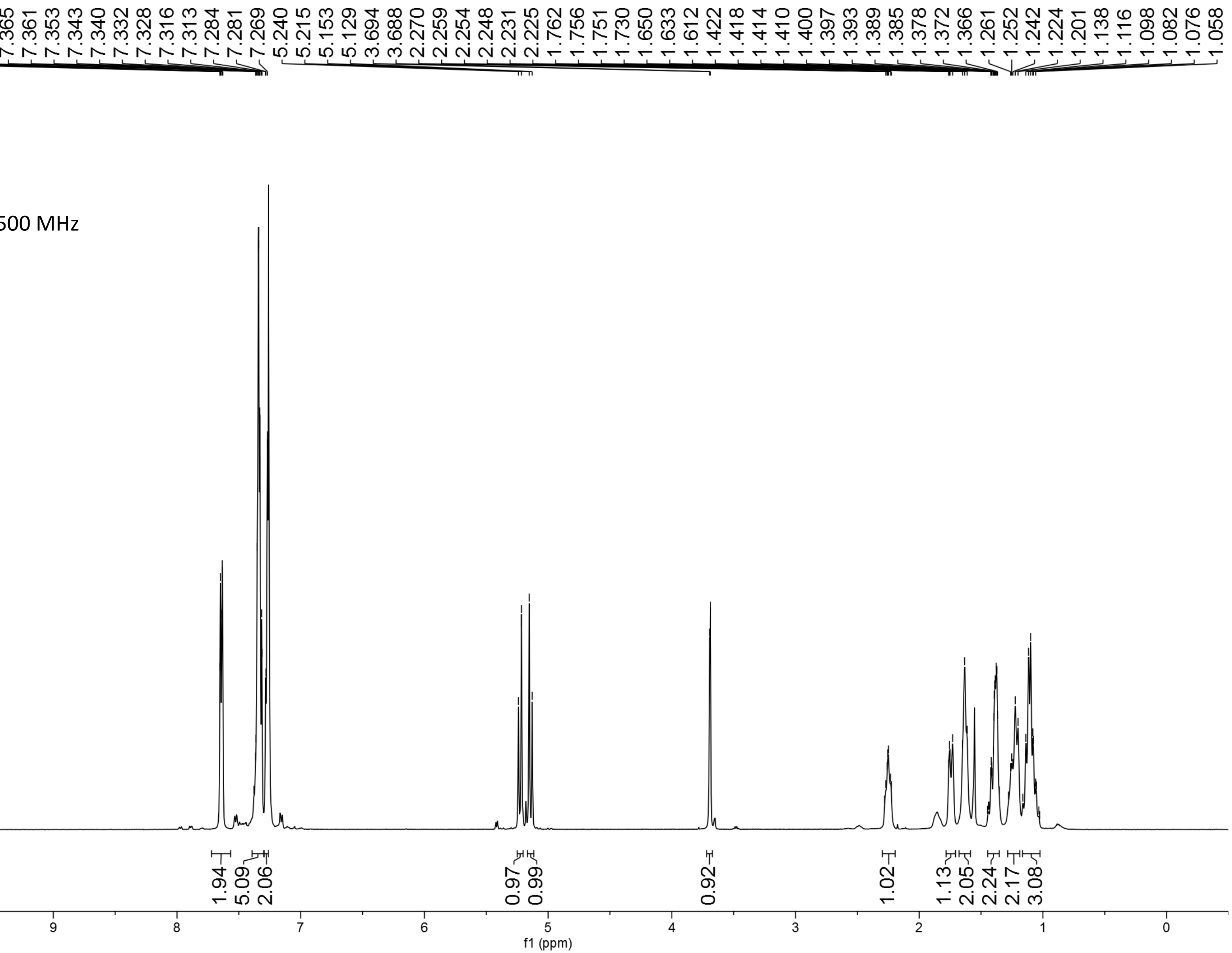

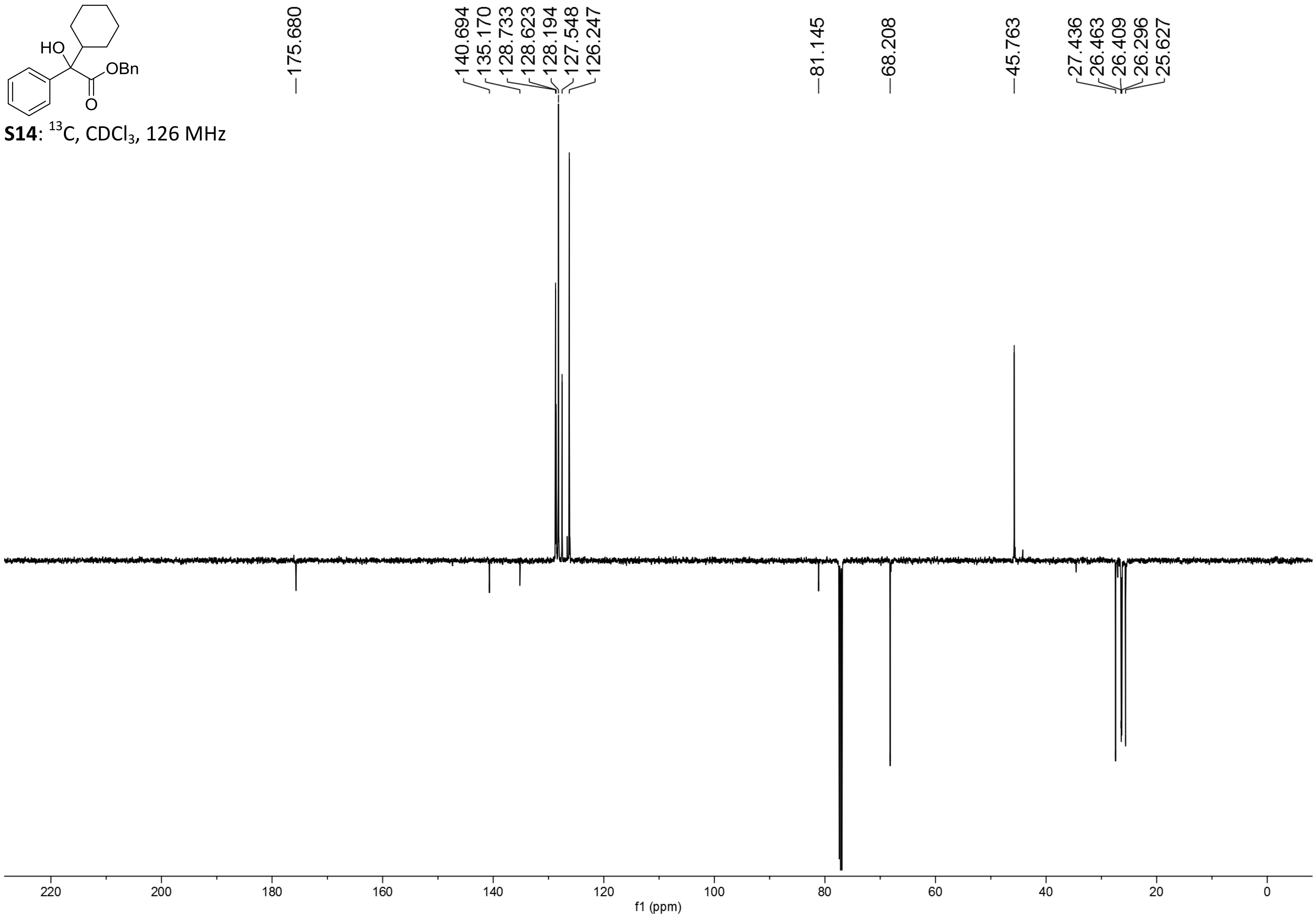

S14: ^{13}C, $CDCl_3$, 126 MHz

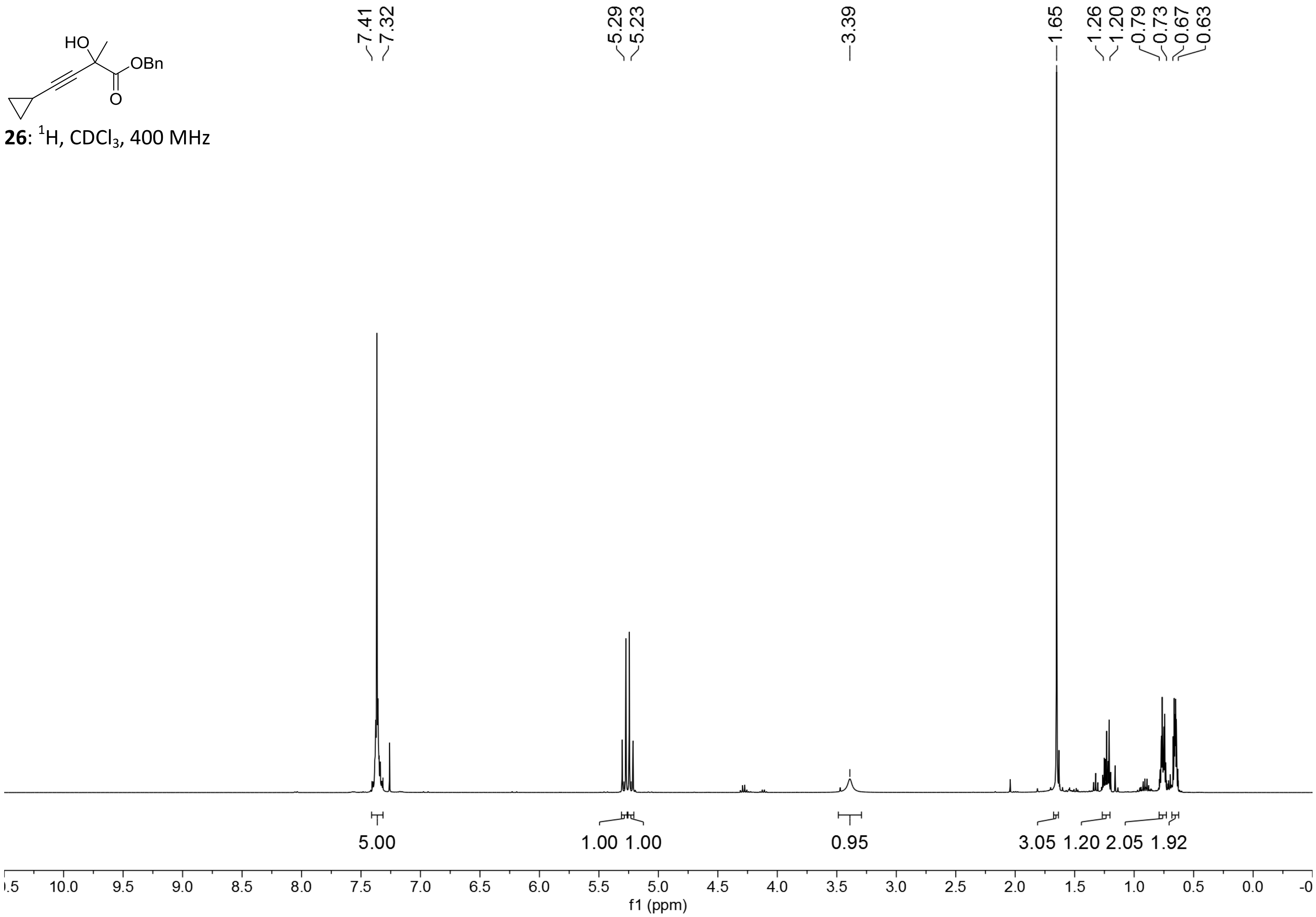

26: ^{1}H, $CDCl_3$, 400 MHz

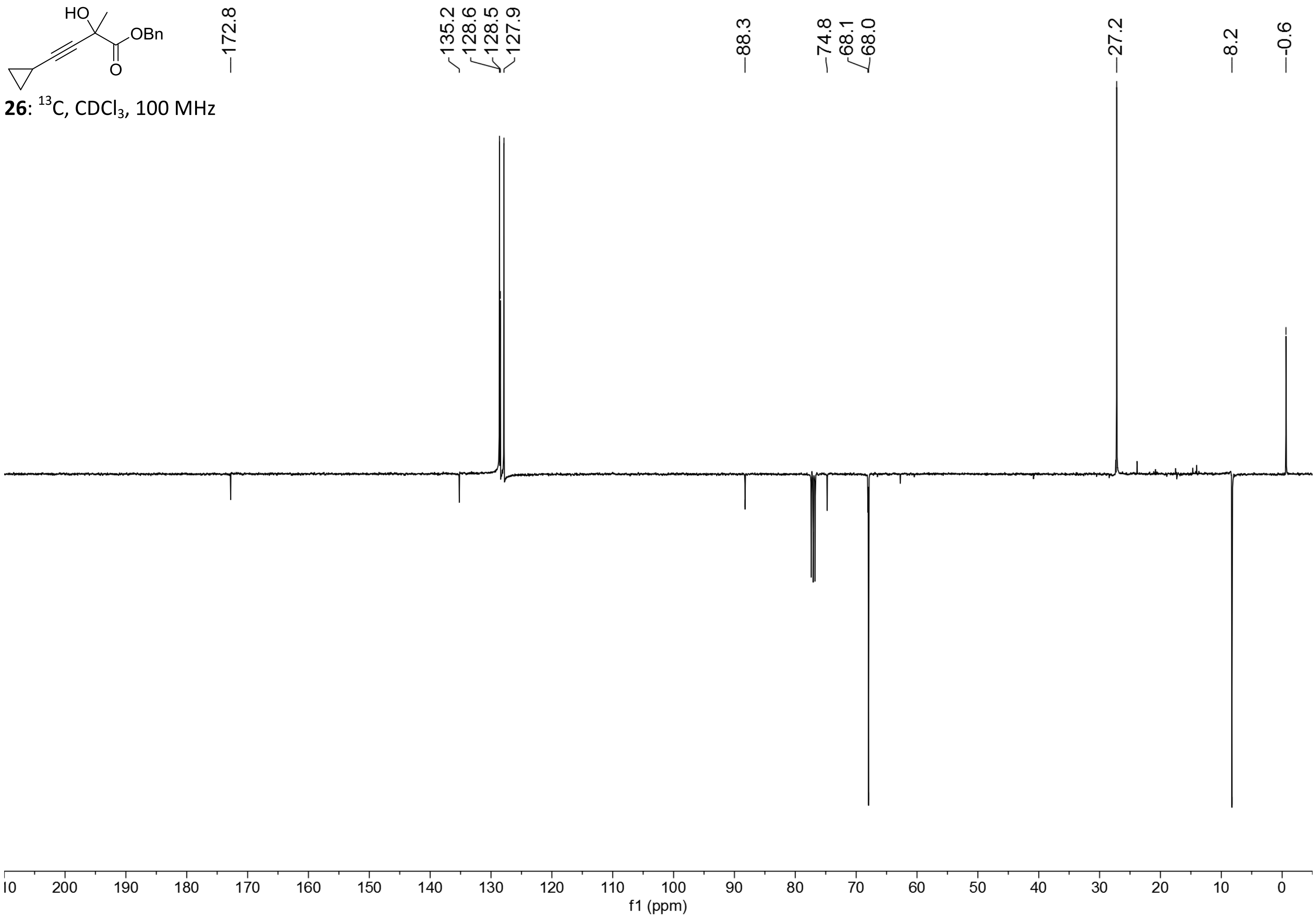

26: ^{13}C, $CDCl_3$, 100 MHz

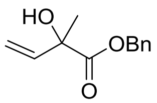

27: ^{1}H, CDCl$_3$, 500 MHz

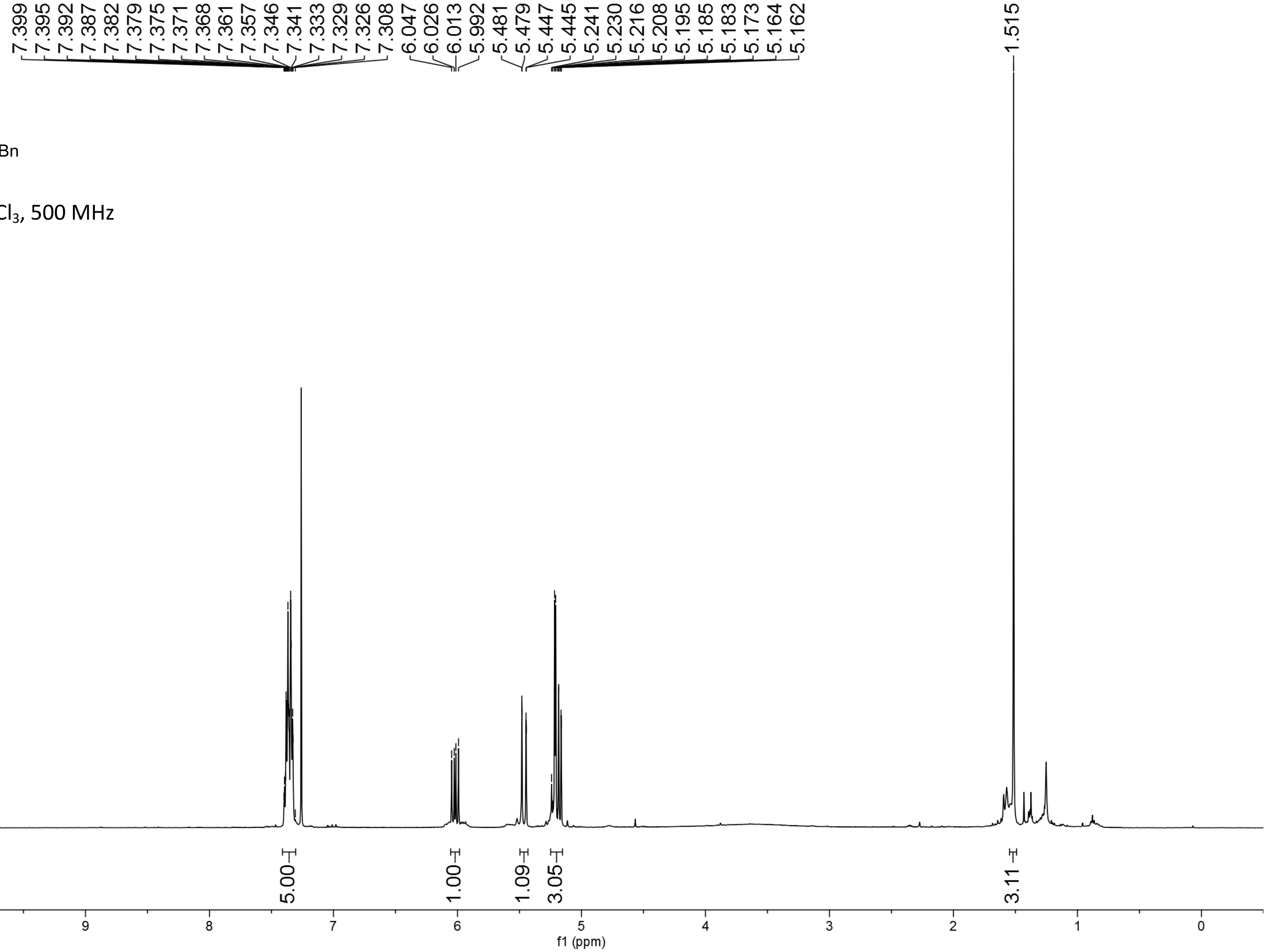

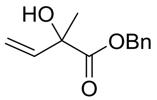

27: ^{13}C, $CDCl_3$, 126 MHz

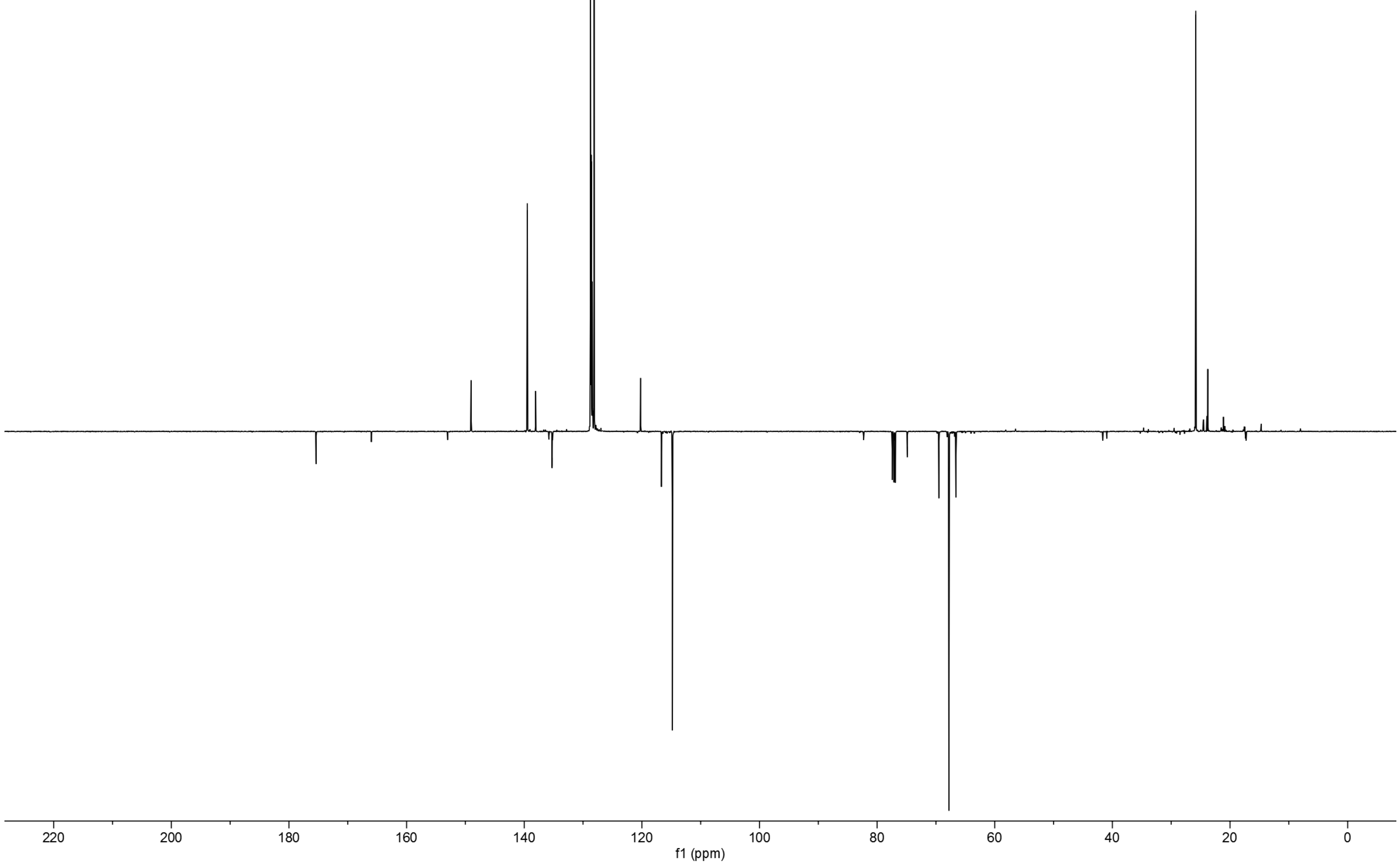

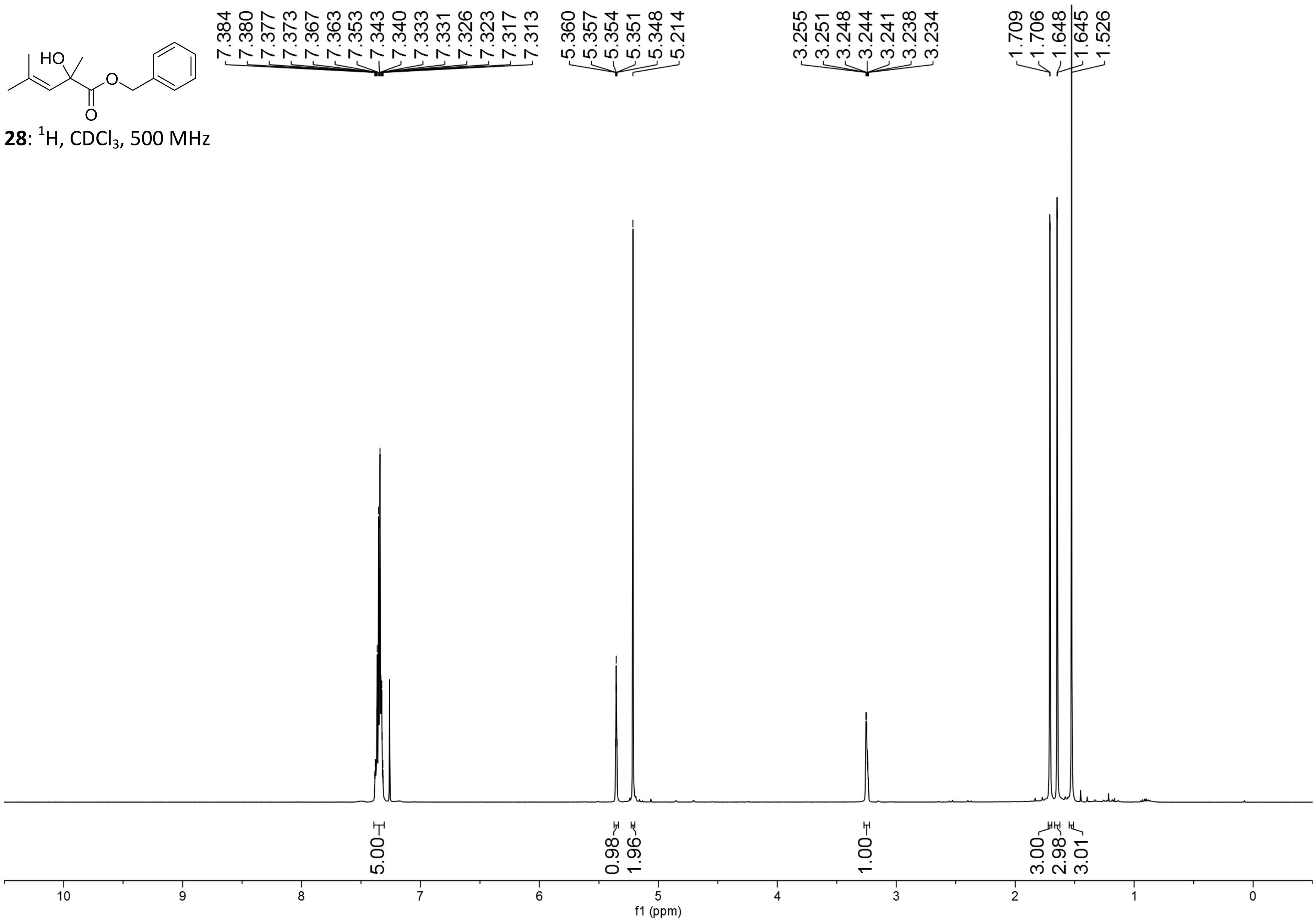

28: ^{1}H, $CDCl_3$, 500 MHz

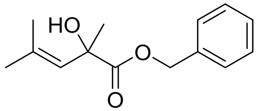

28: ^{13}C, $CDCl_3$, 126 MHz

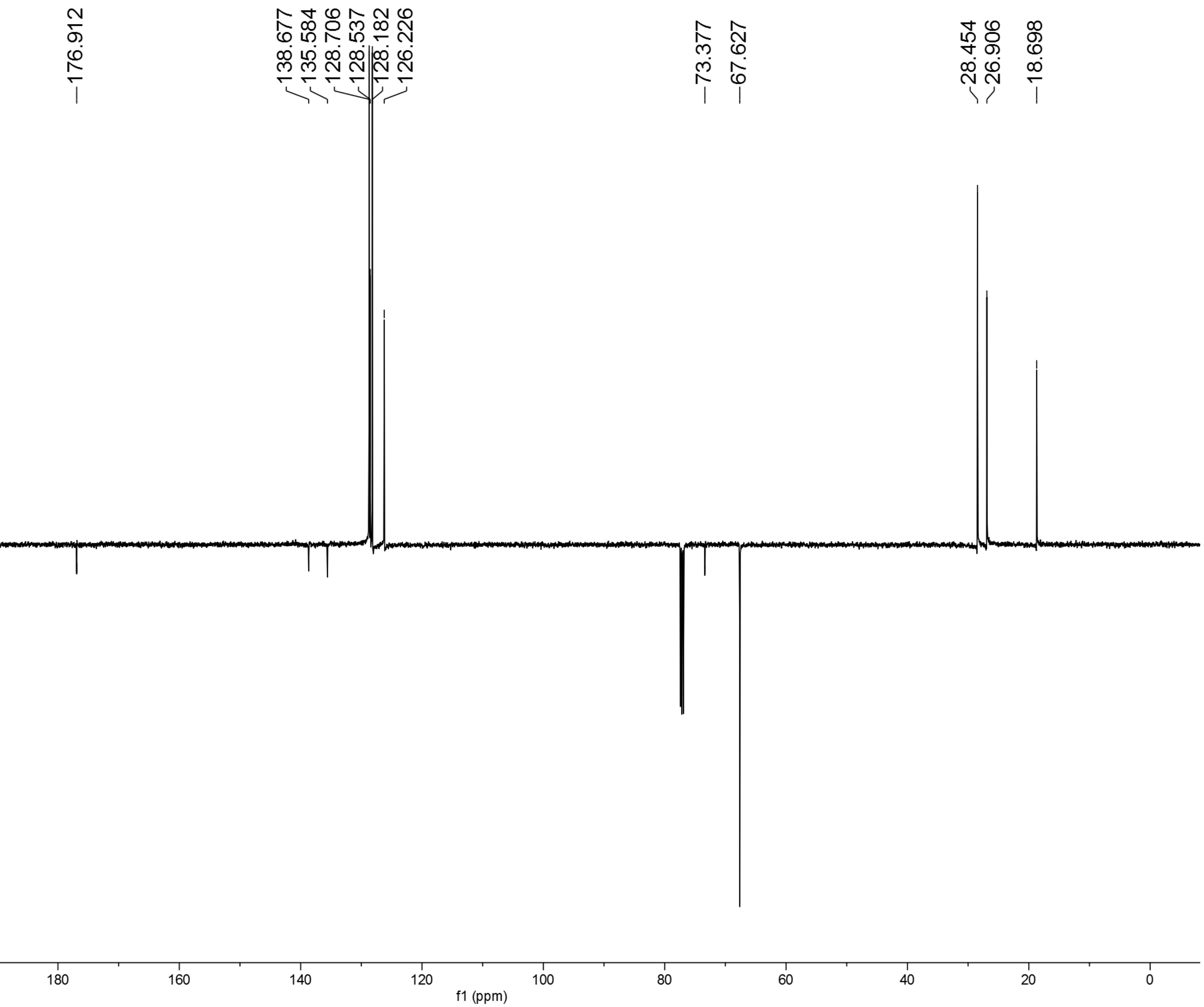

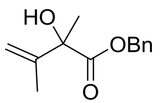

29: ^{1}H, $CDCl_3$, 500 MHz

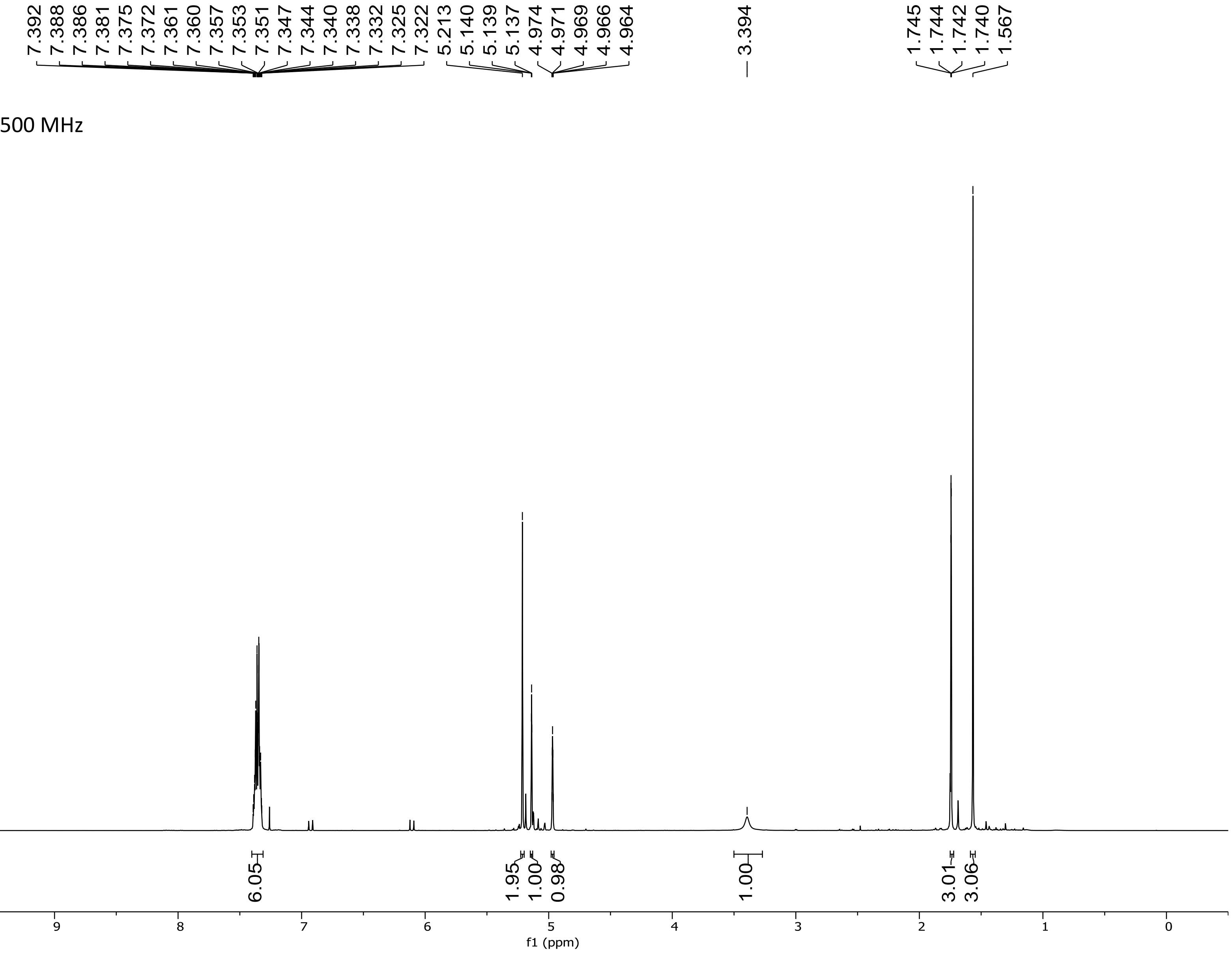

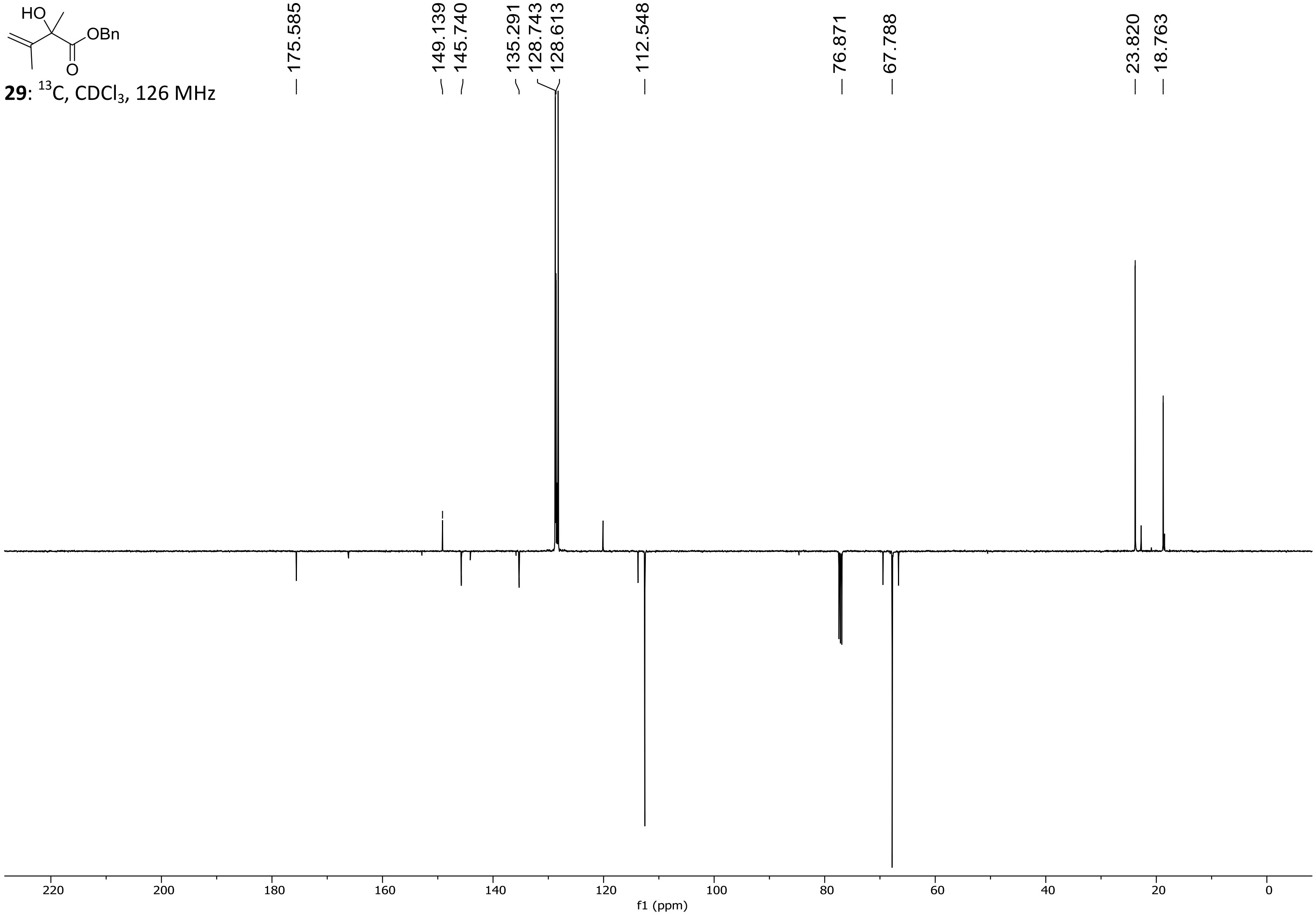

29: ^{13}C, $CDCl_3$, 126 MHz

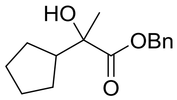

30: ^{1}H, $CDCl_3$, 500 MHz

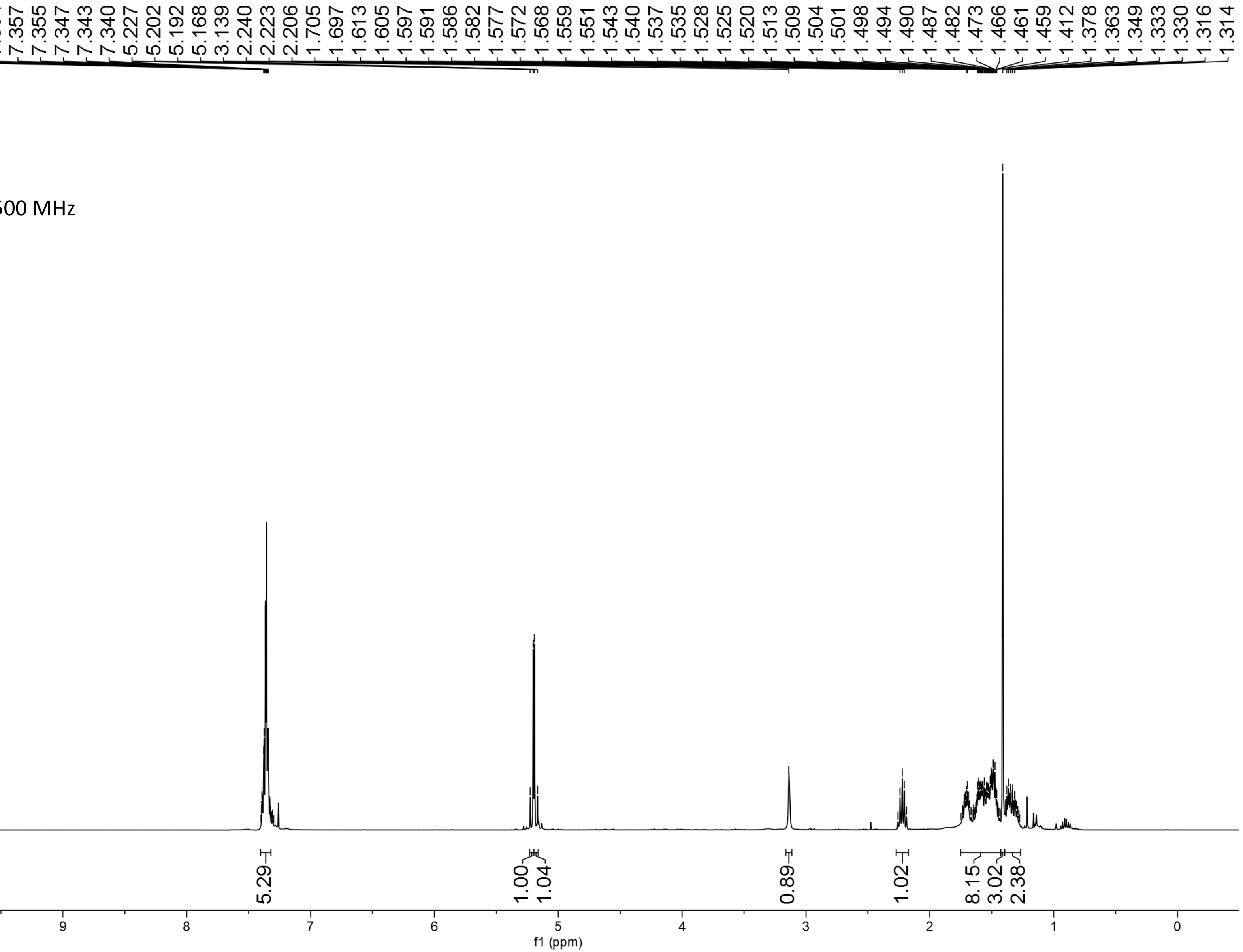

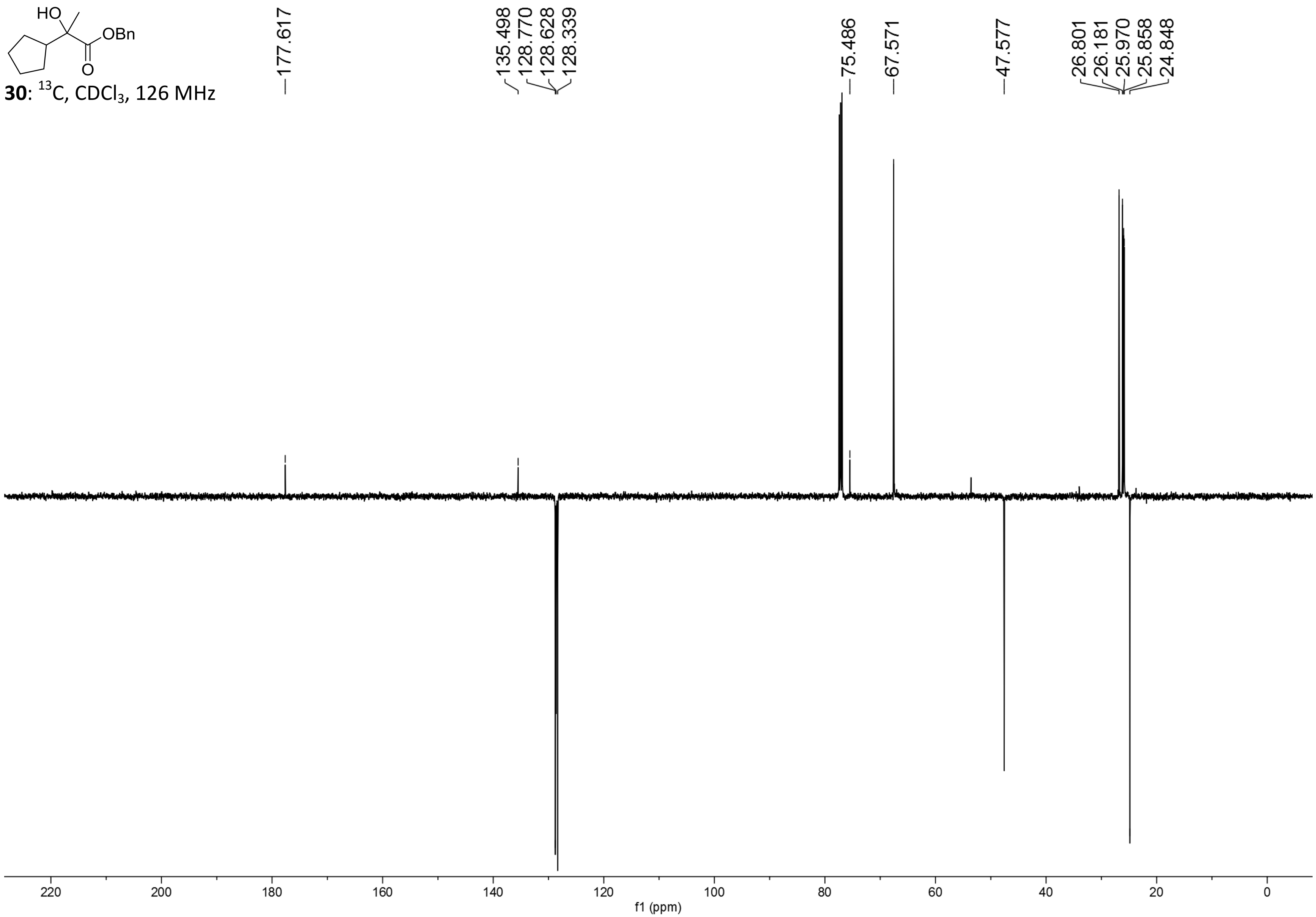

30: ^{13}C, $CDCl_3$, 126 MHz

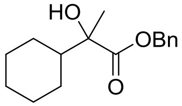

31: ^{1}H, $CDCl_3$, 500 MHz

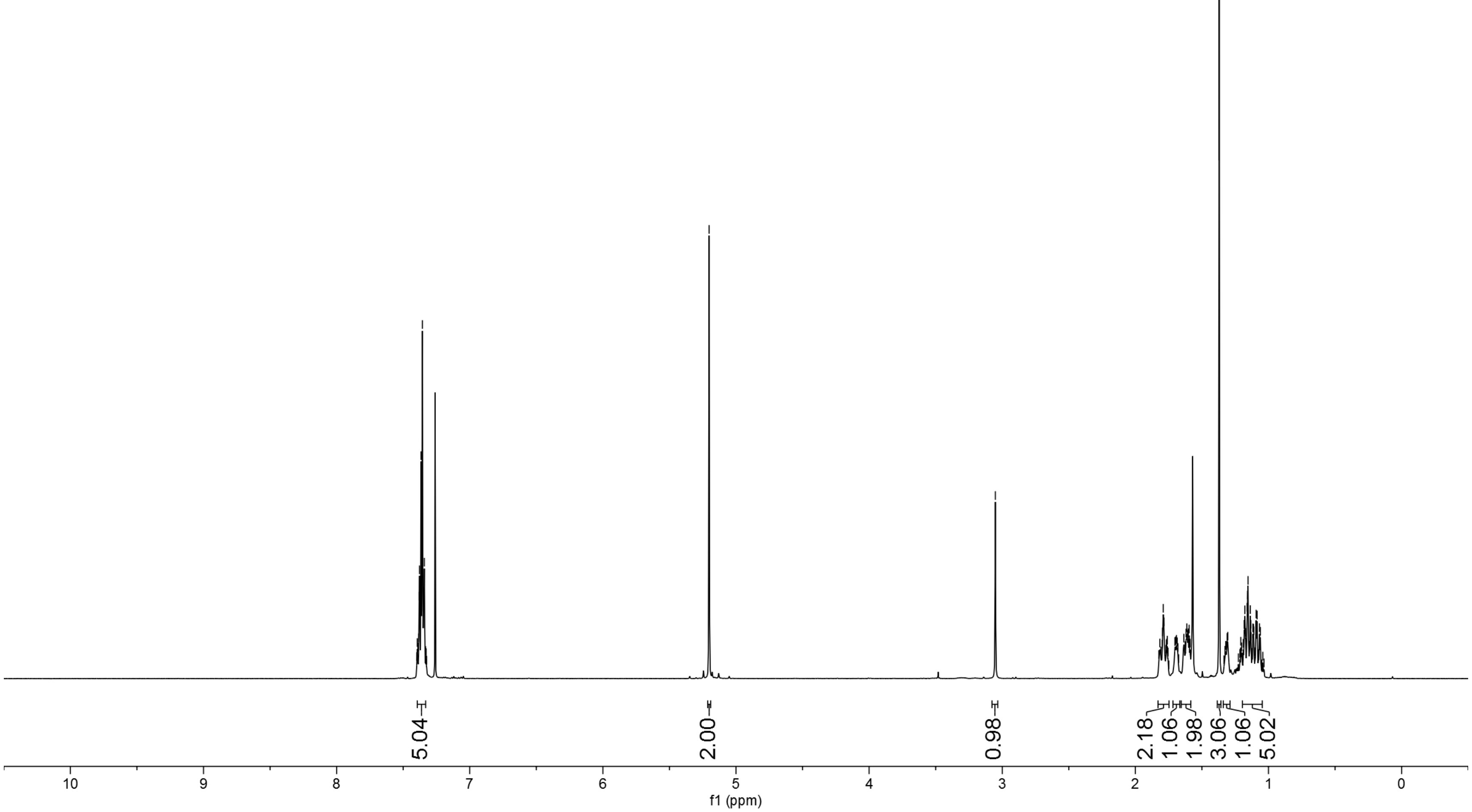

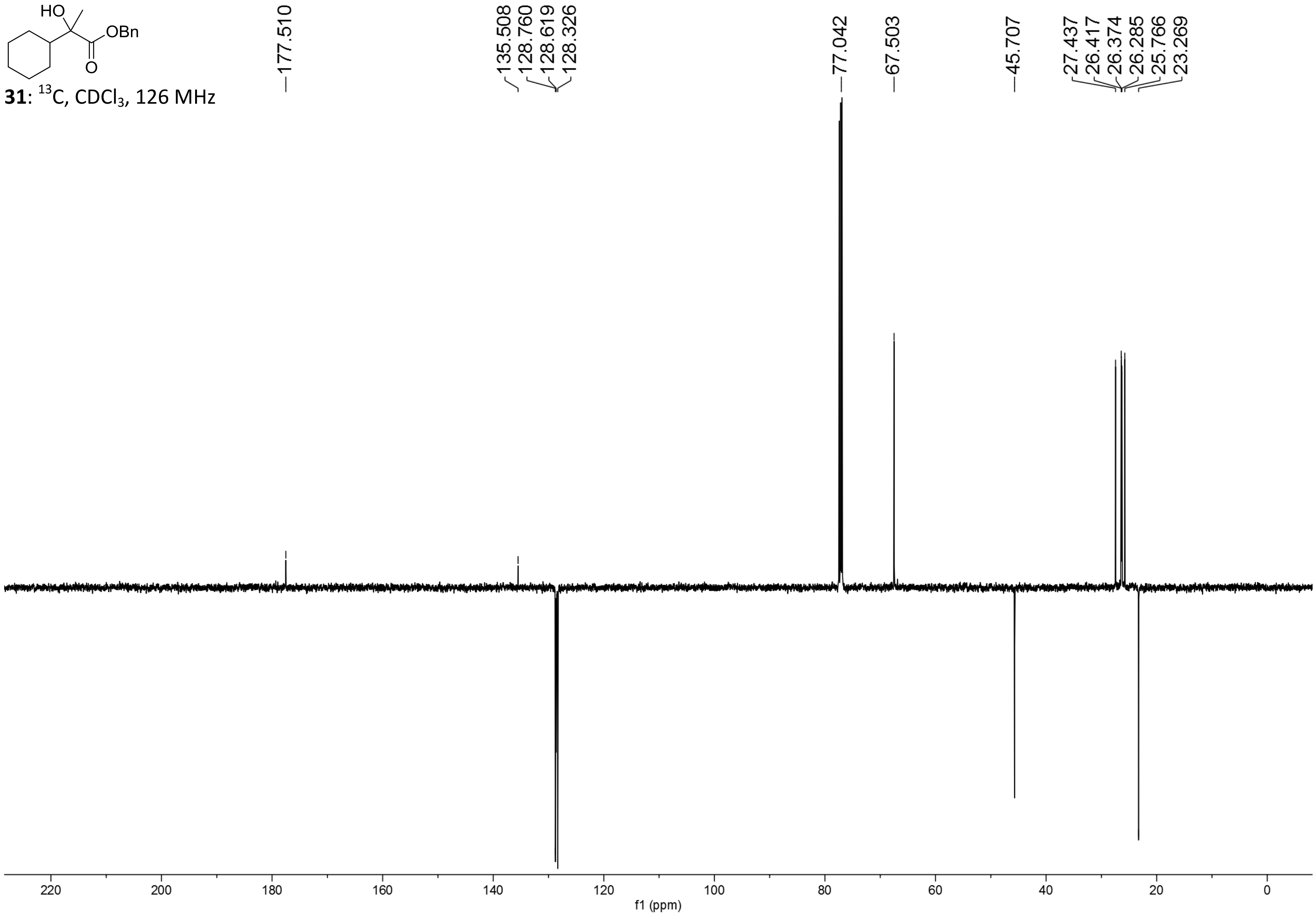

31: ^{13}C, $CDCl_3$, 126 MHz

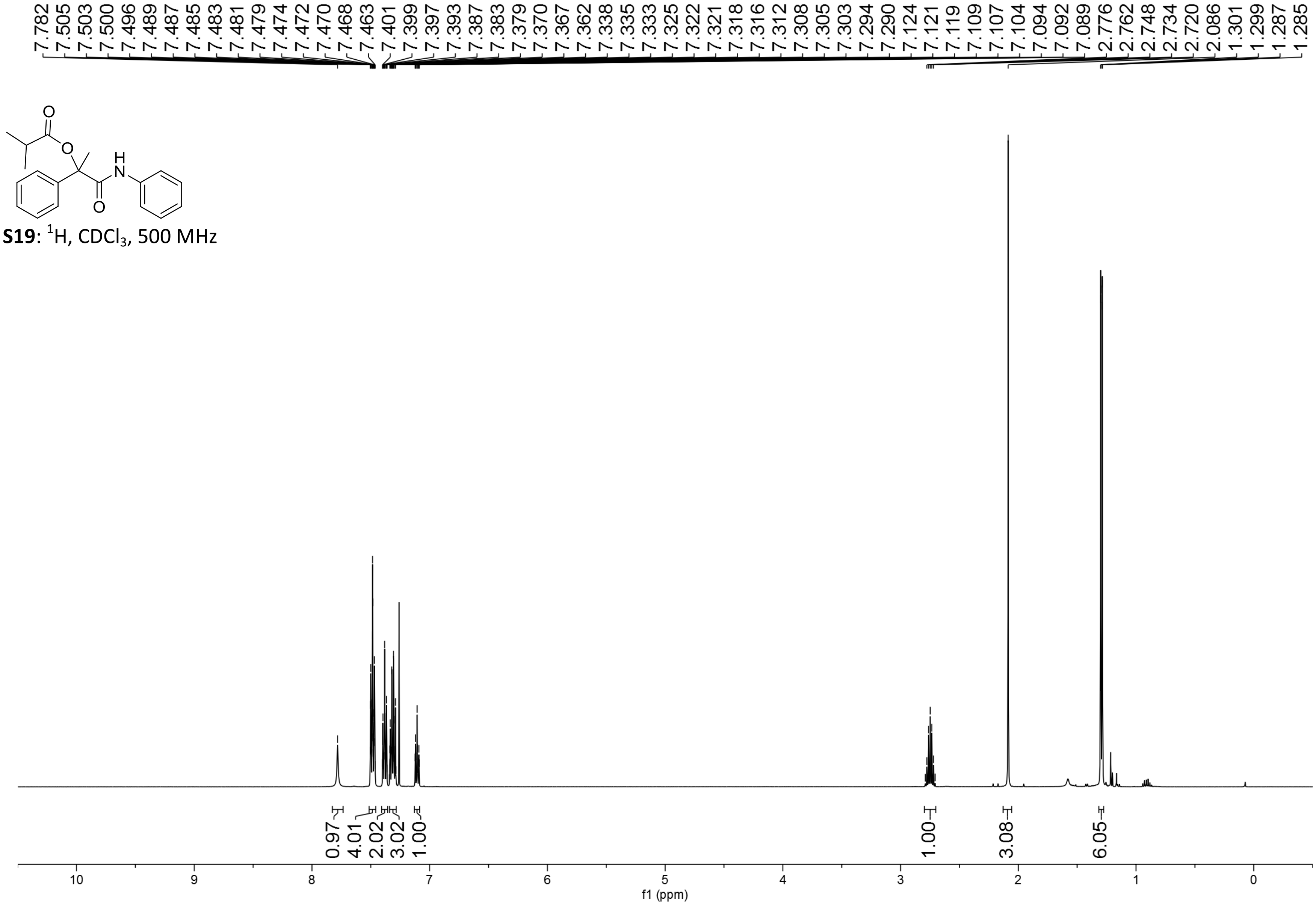

S19: ^{1}H, $CDCl_3$, 500 MHz

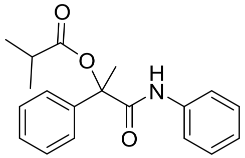

S19: ^{13}C, $CDCl_3$, 100 MHz

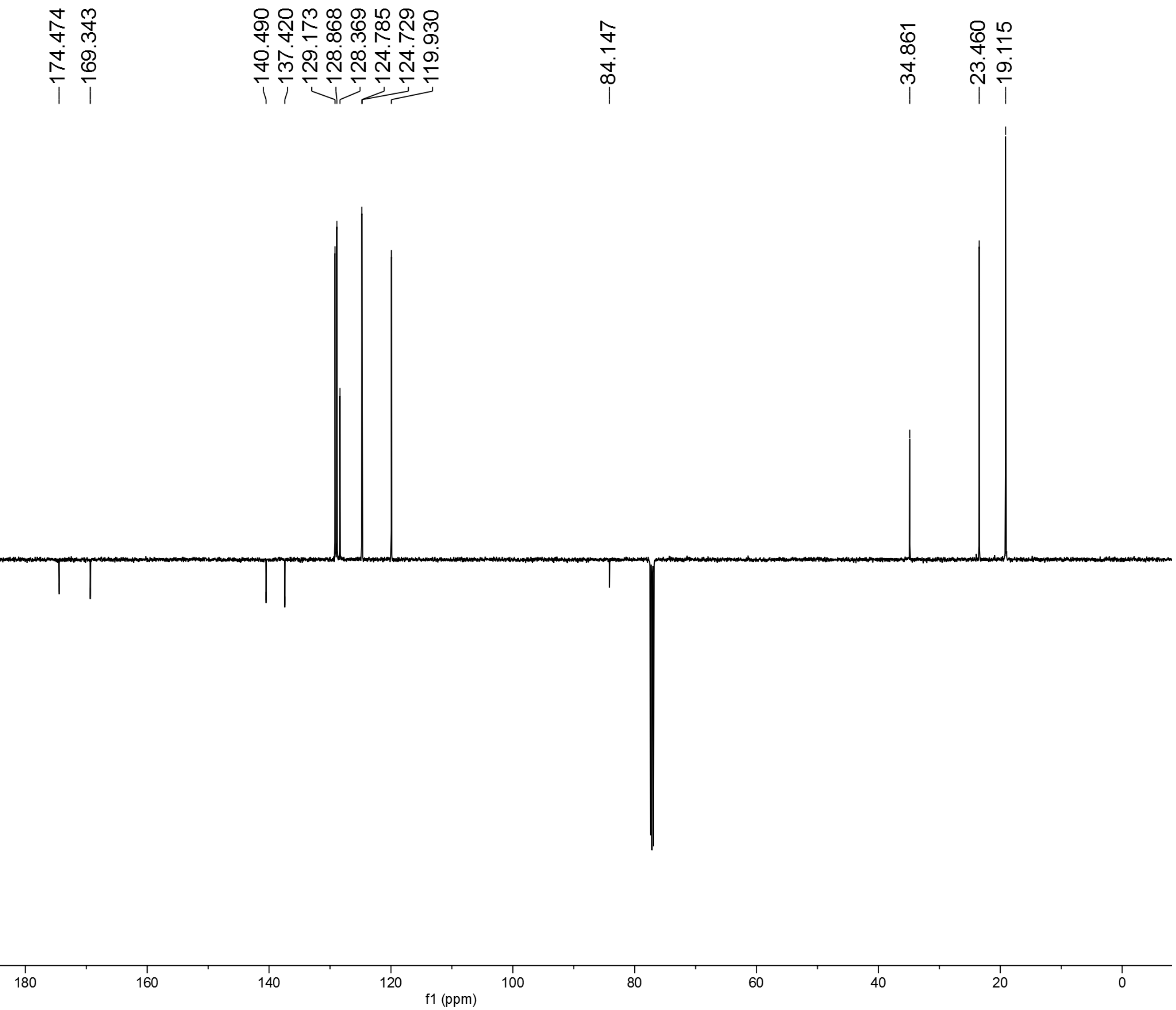

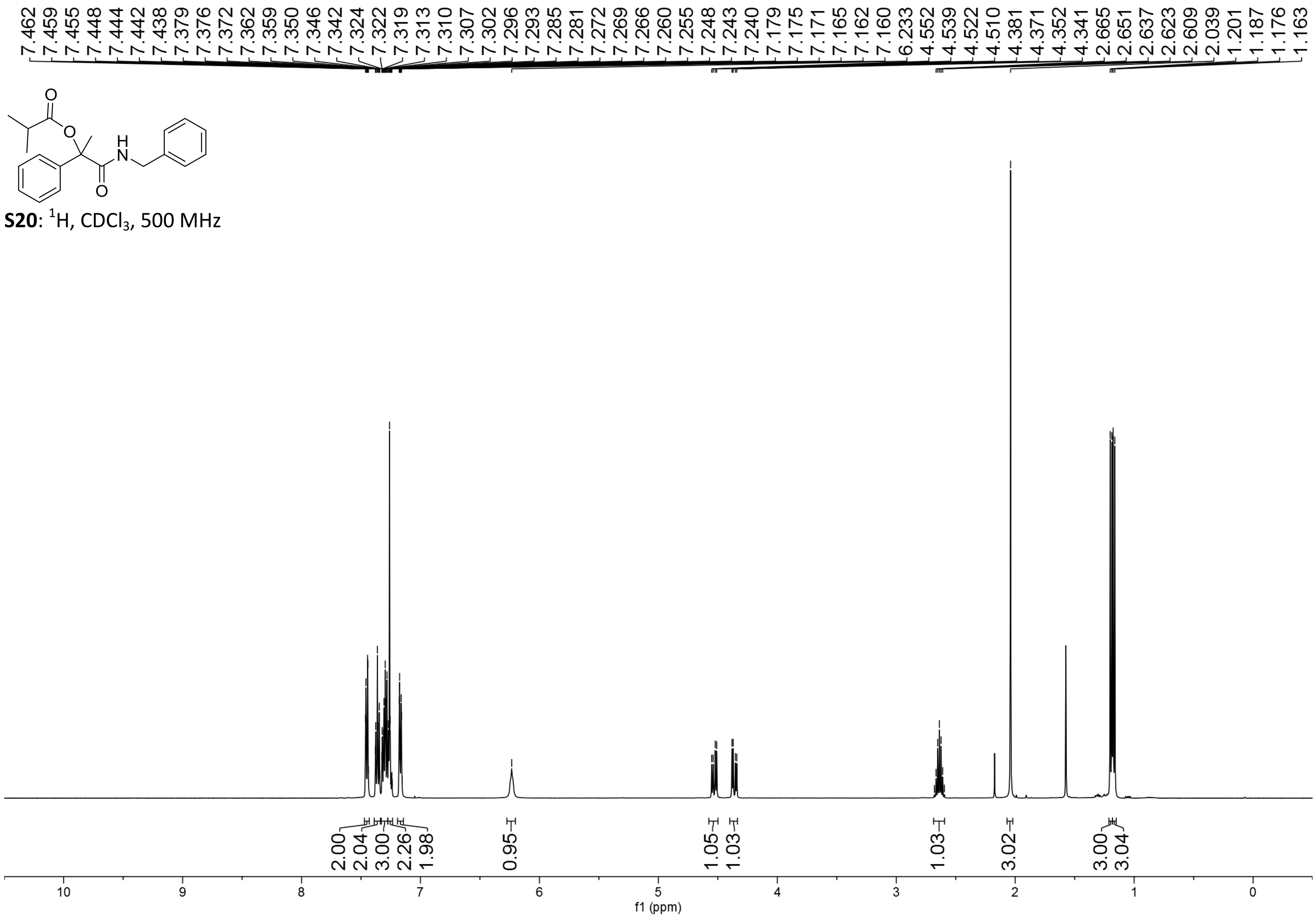
S20: 1H, CDCl3, 500 MHz
f1 (ppm)

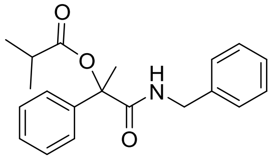

S20: ^{13}C, $CDCl_3$, 100 MHz

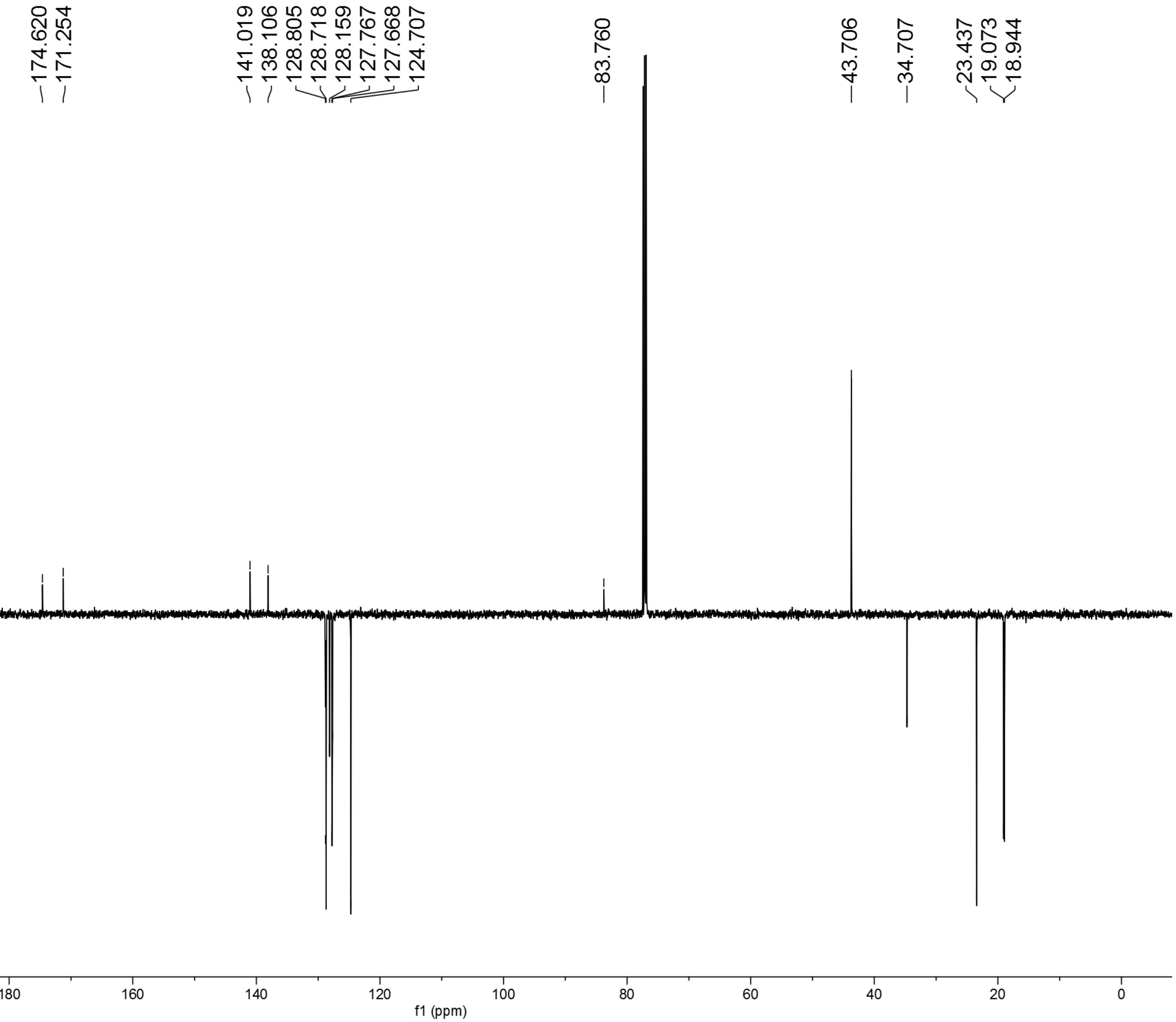

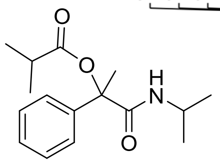

S21: ^{1}H, $CDCl_3$, 500 MHz

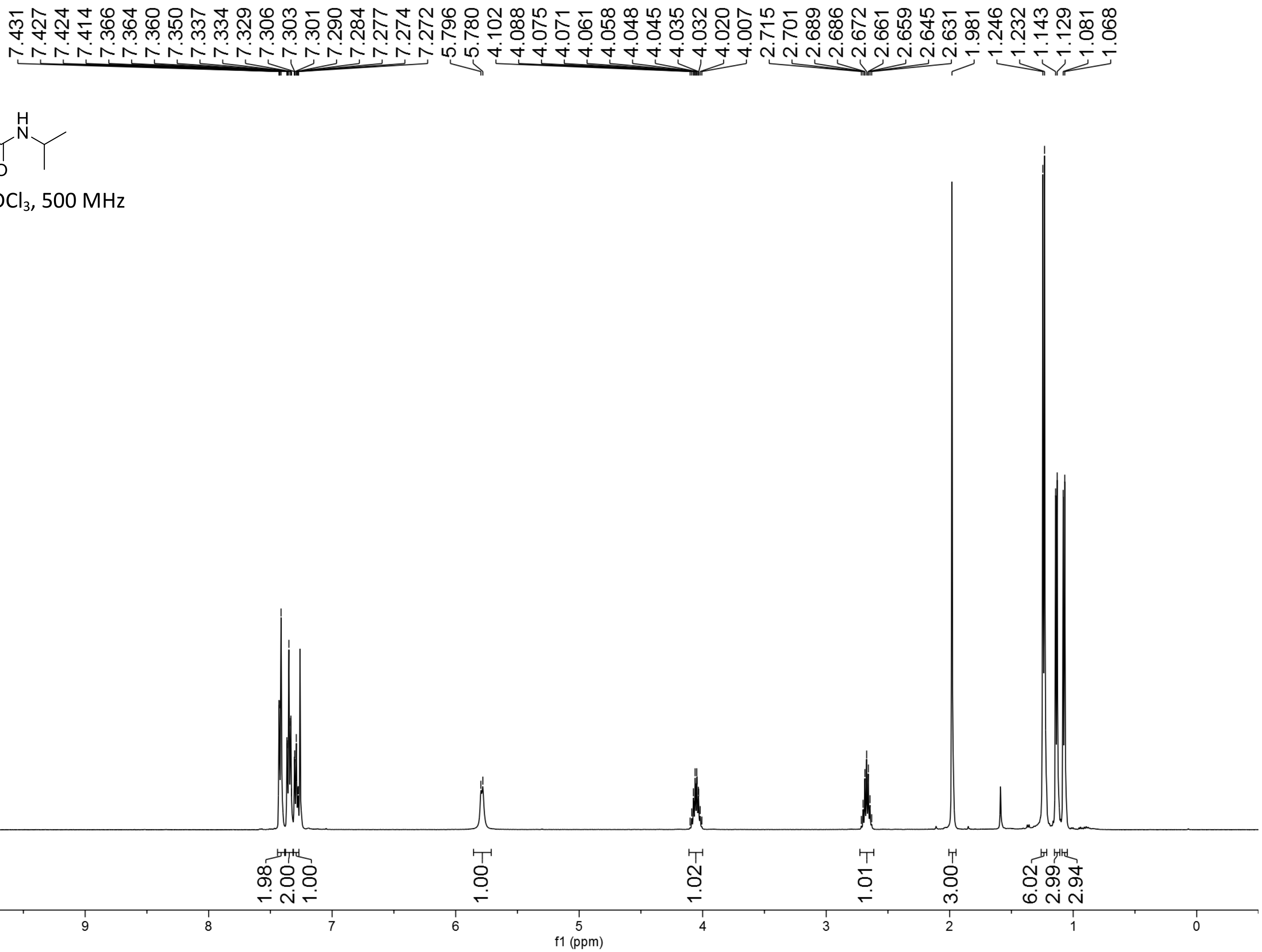

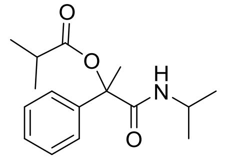

S21: ^{13}C, $CDCl_3$, 100 MHz

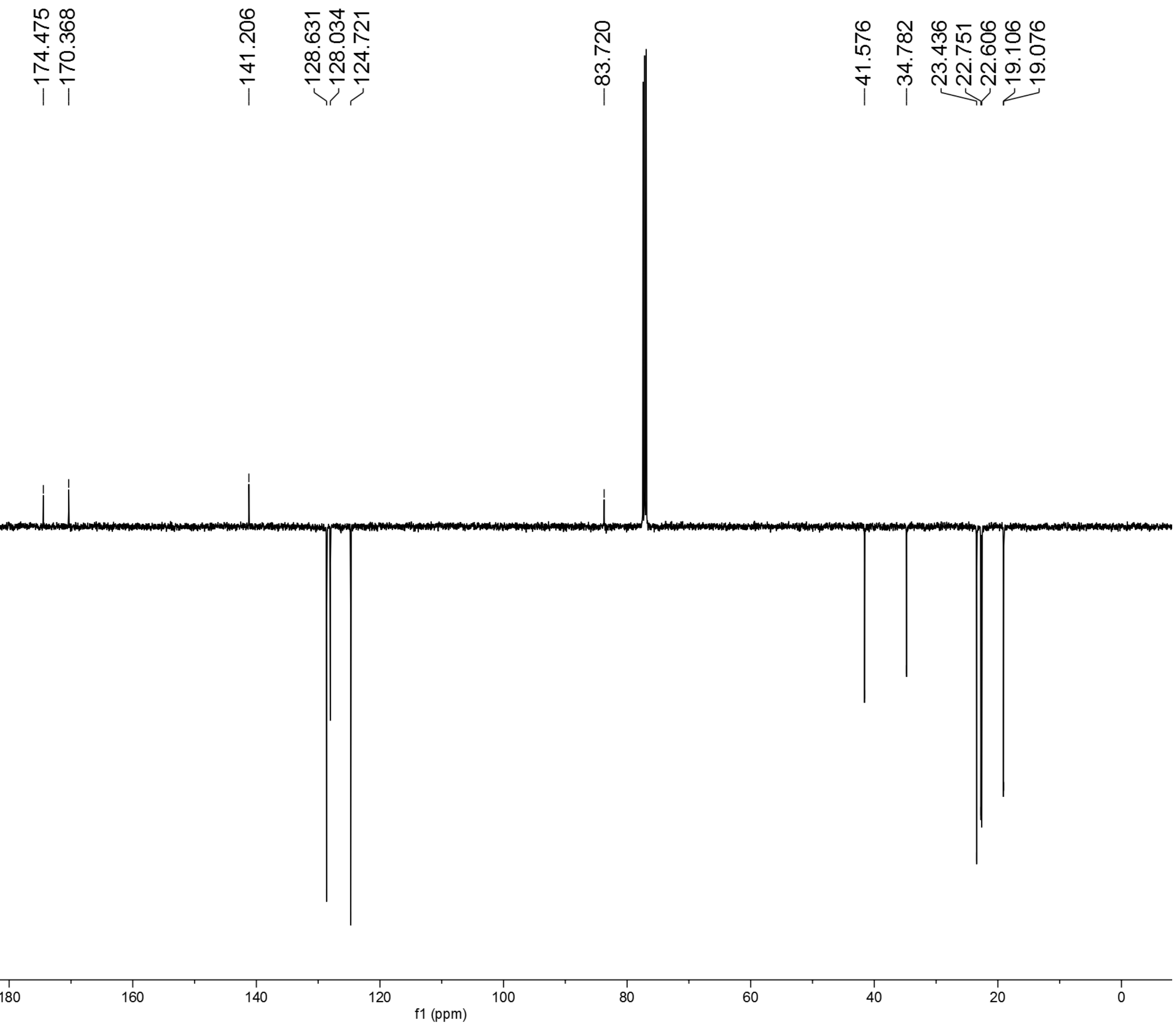

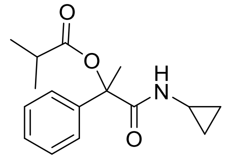

S22: ^{1}H, $CDCl_3$, 500 MHz

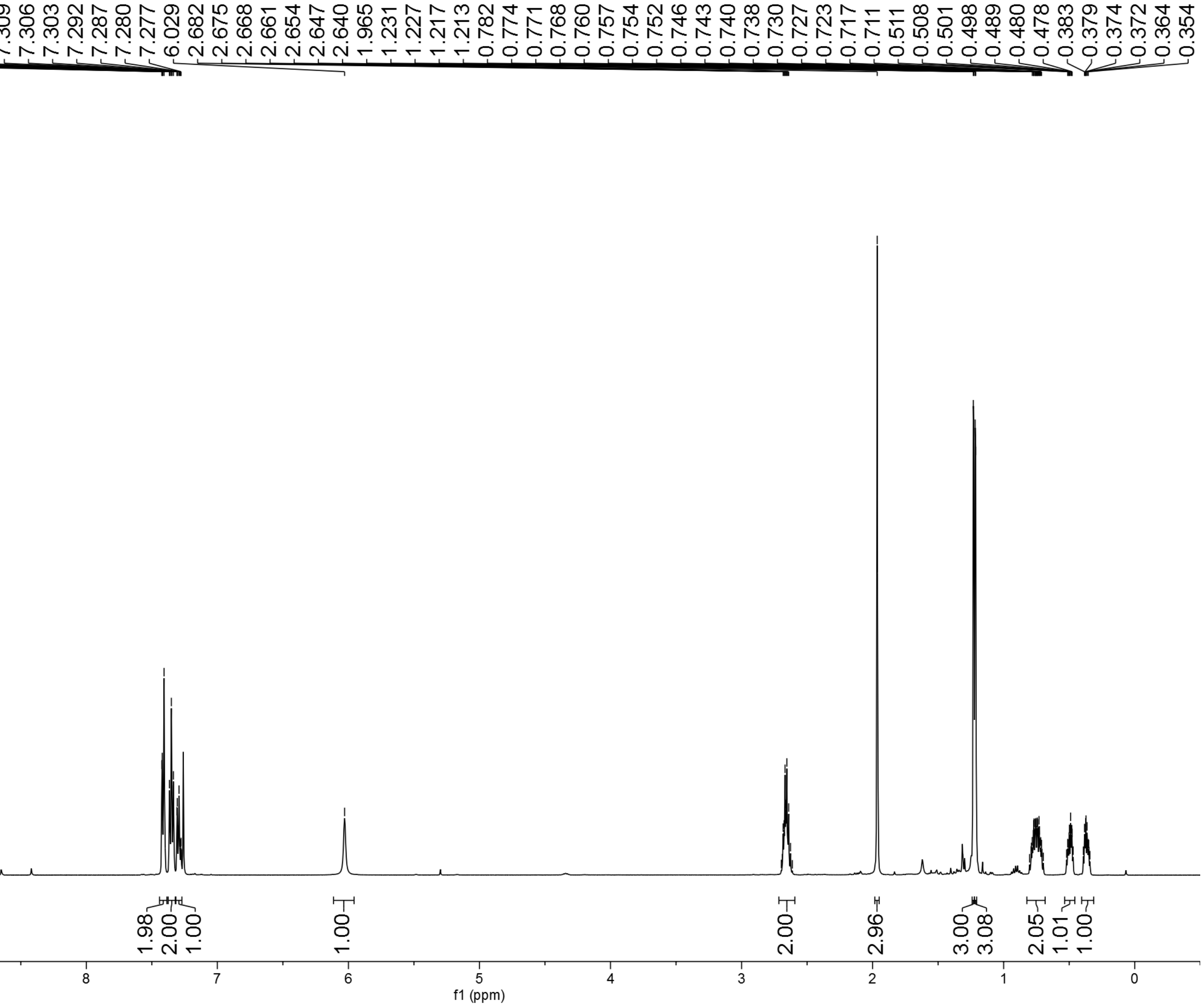

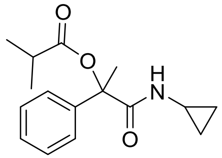

S22: ^{13}C, $CDCl_3$, 100 MHz

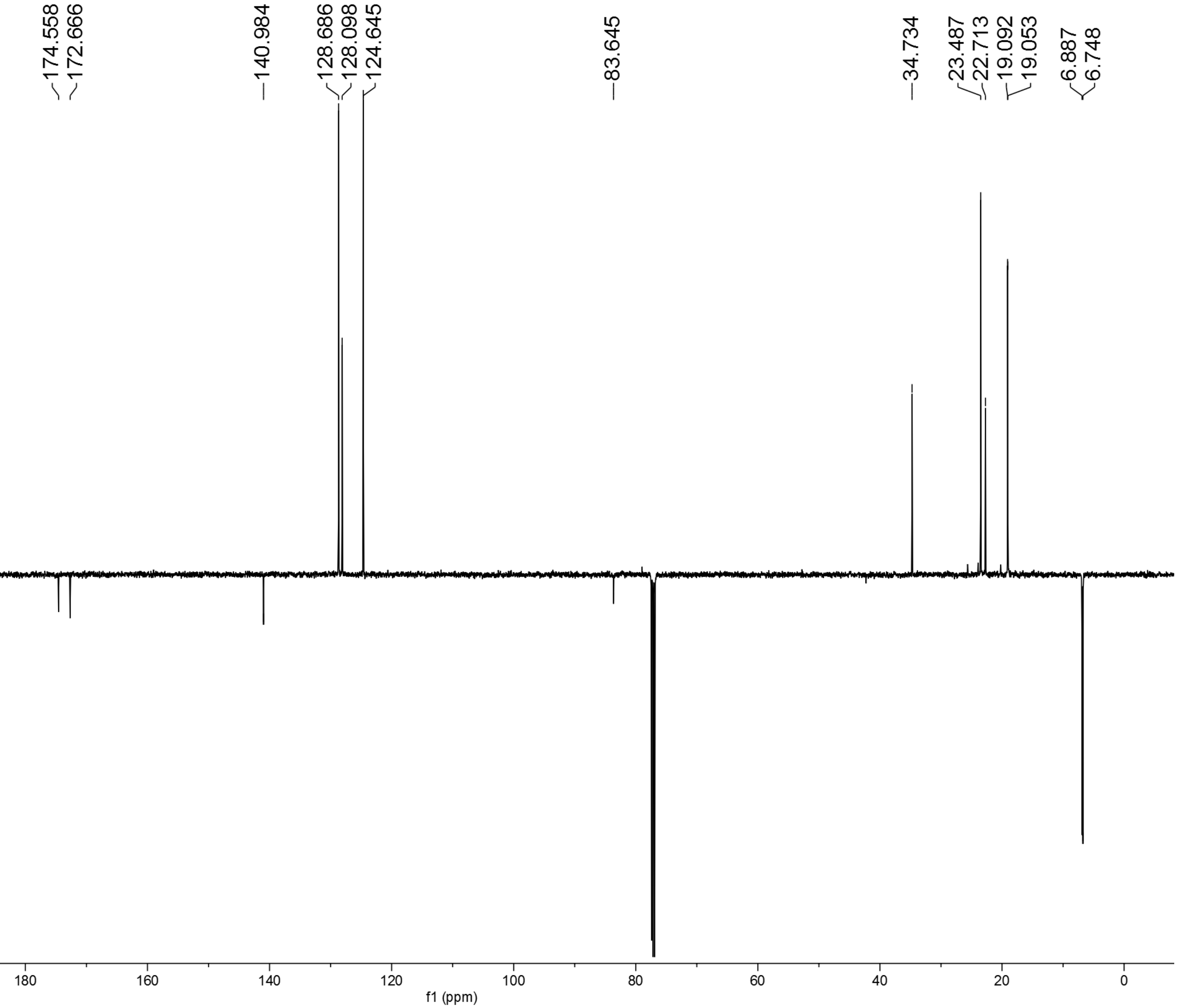

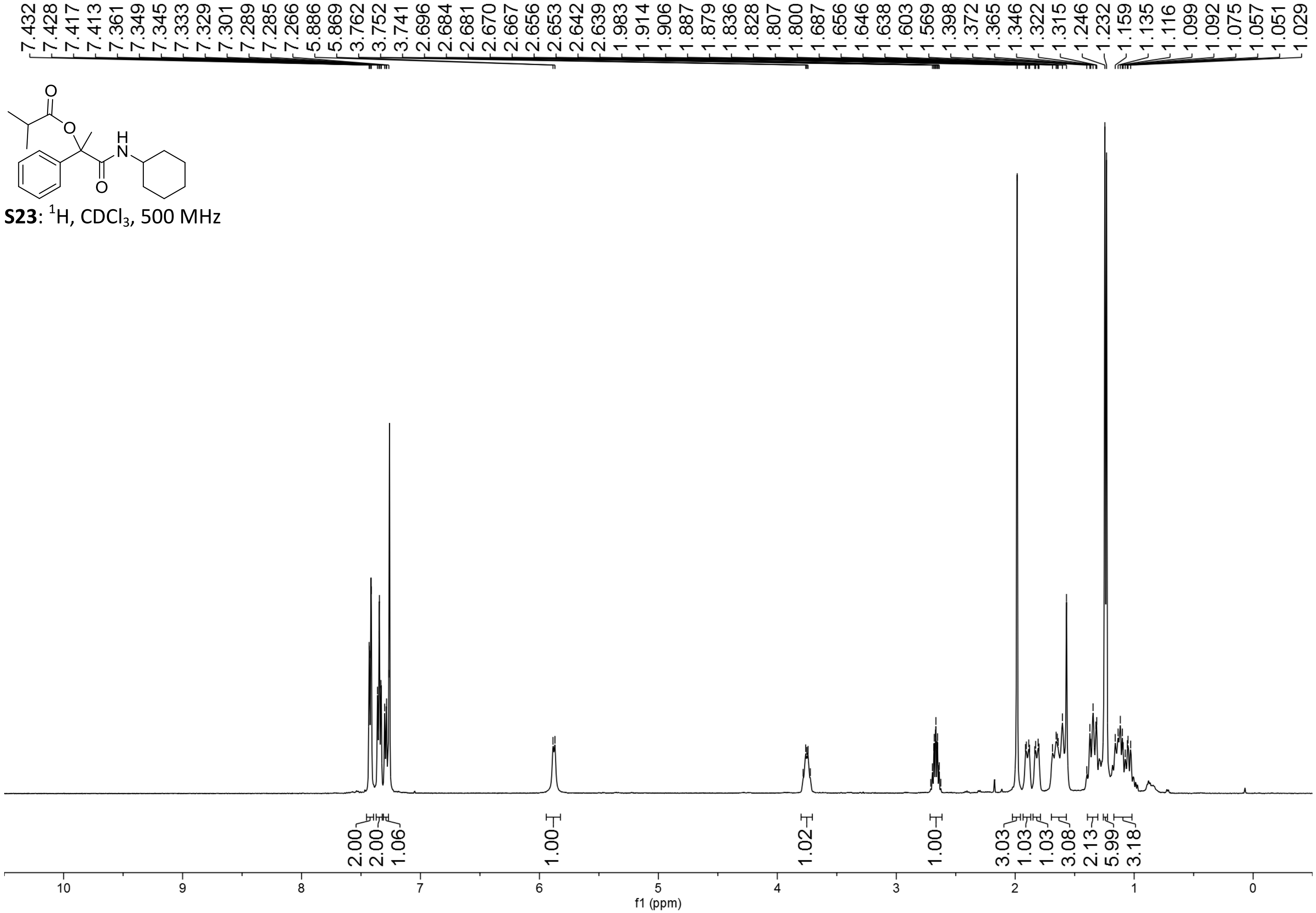

S23: ^{1}H, $CDCl_3$, 500 MHz

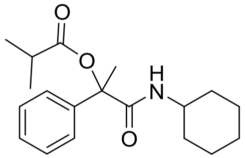

S23: ^{13}C, $CDCl_3$, 100 MHz

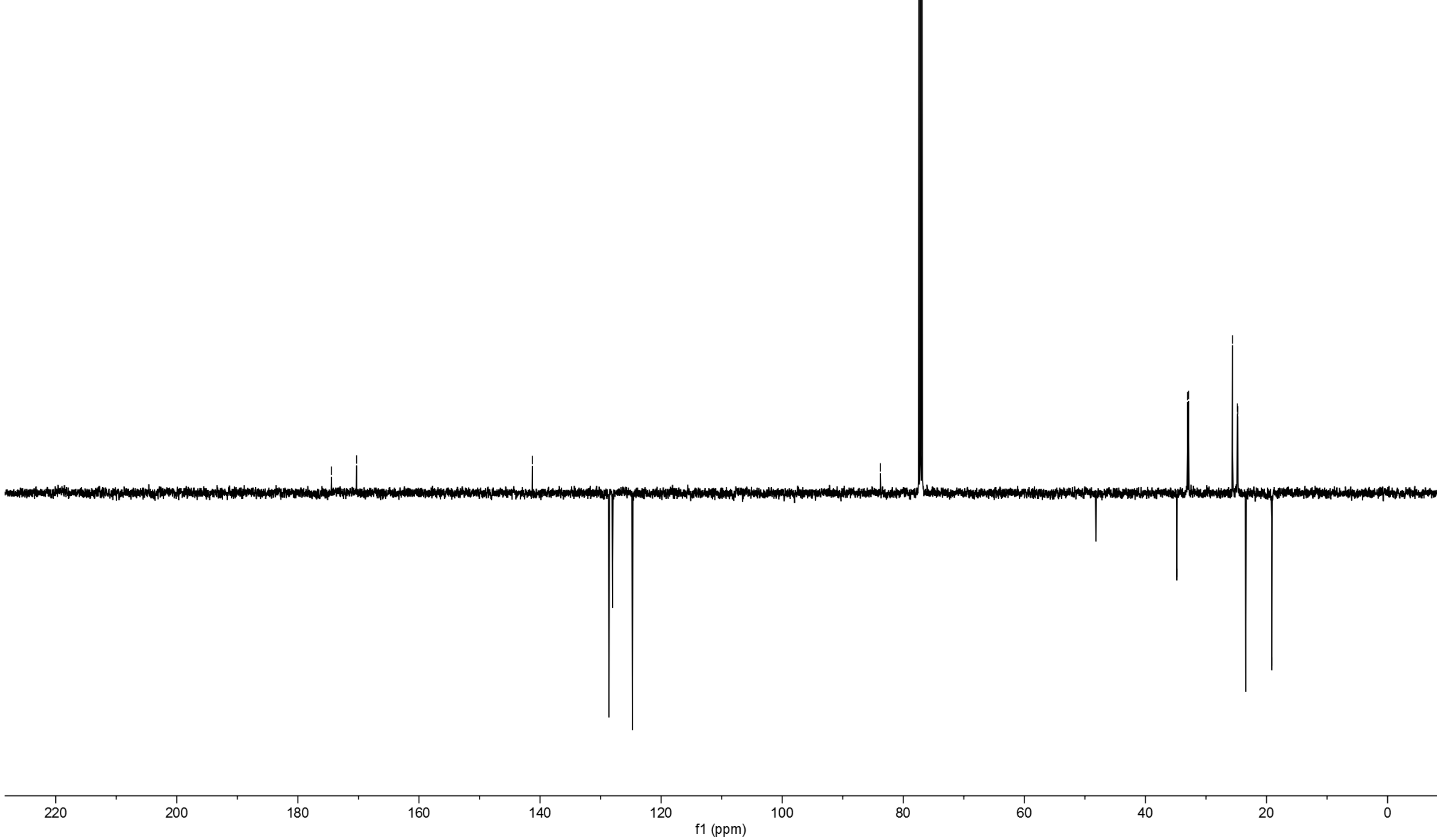

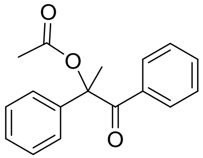

S24: [1]H, $CDCl_3$, 400 MHz

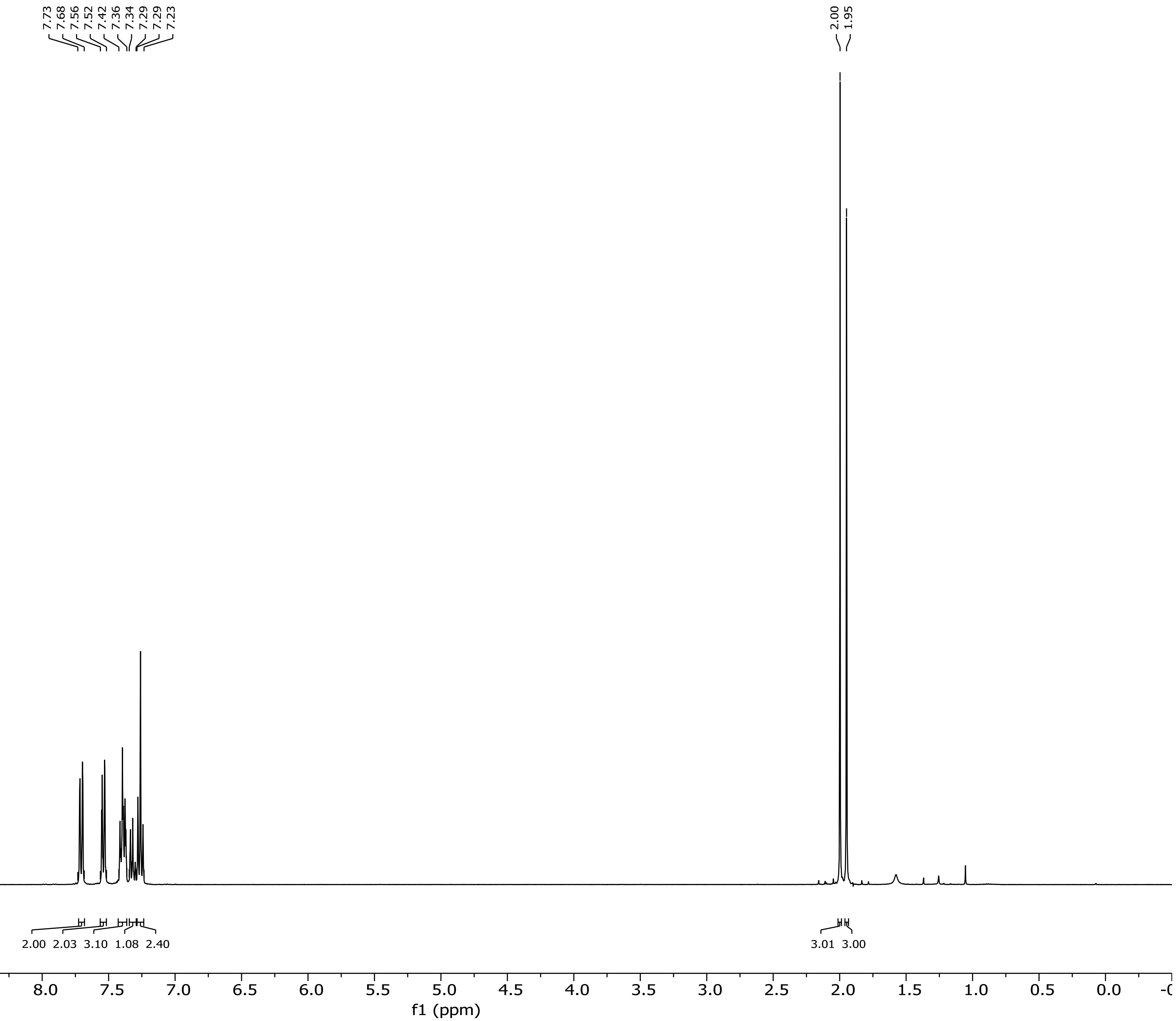

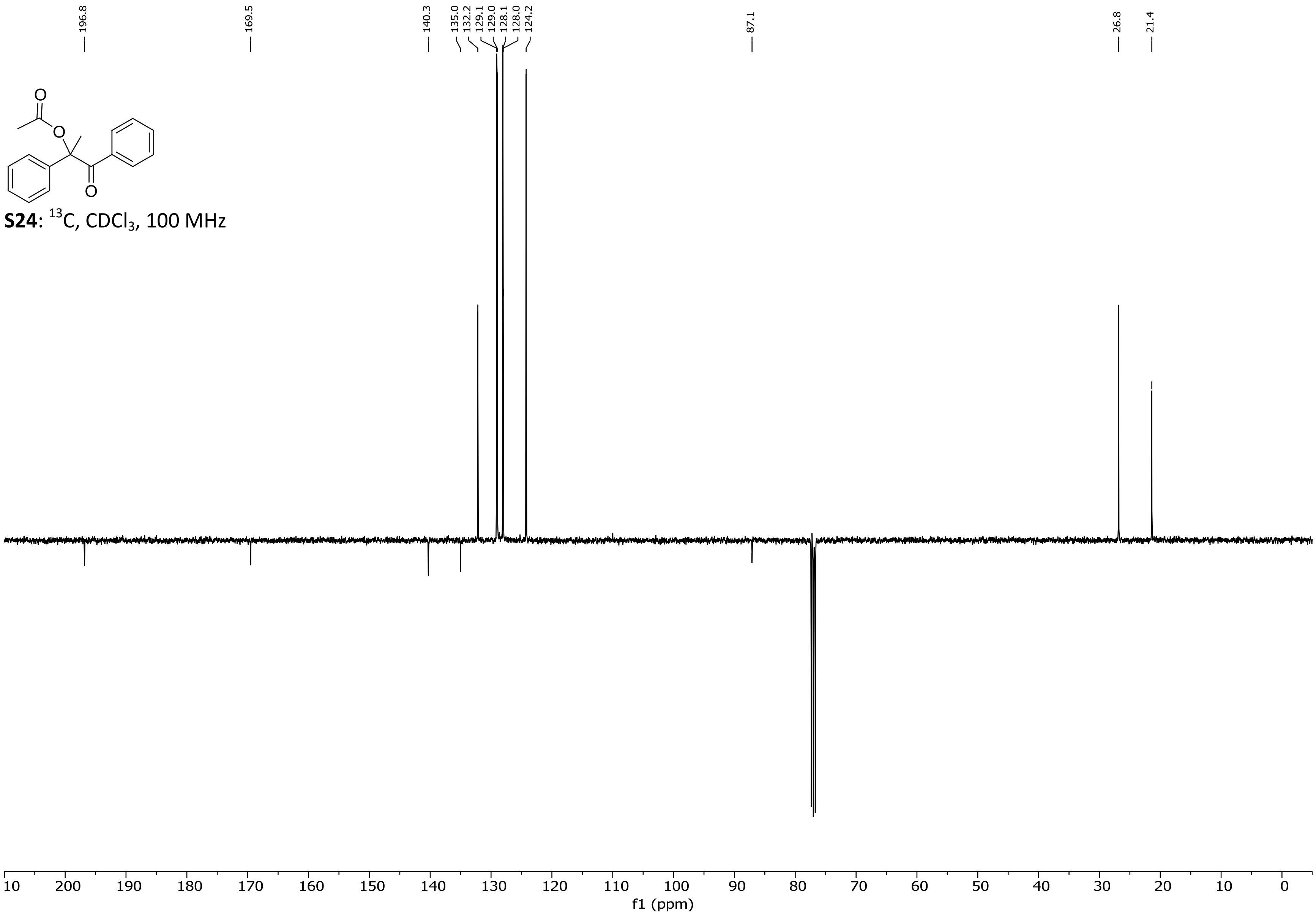

S24: ${}^{13}C$, $CDCl_3$, 100 MHz

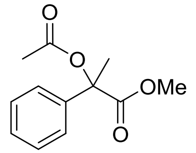

S25: ^{1}H, $CDCl_3$, 400 MHz

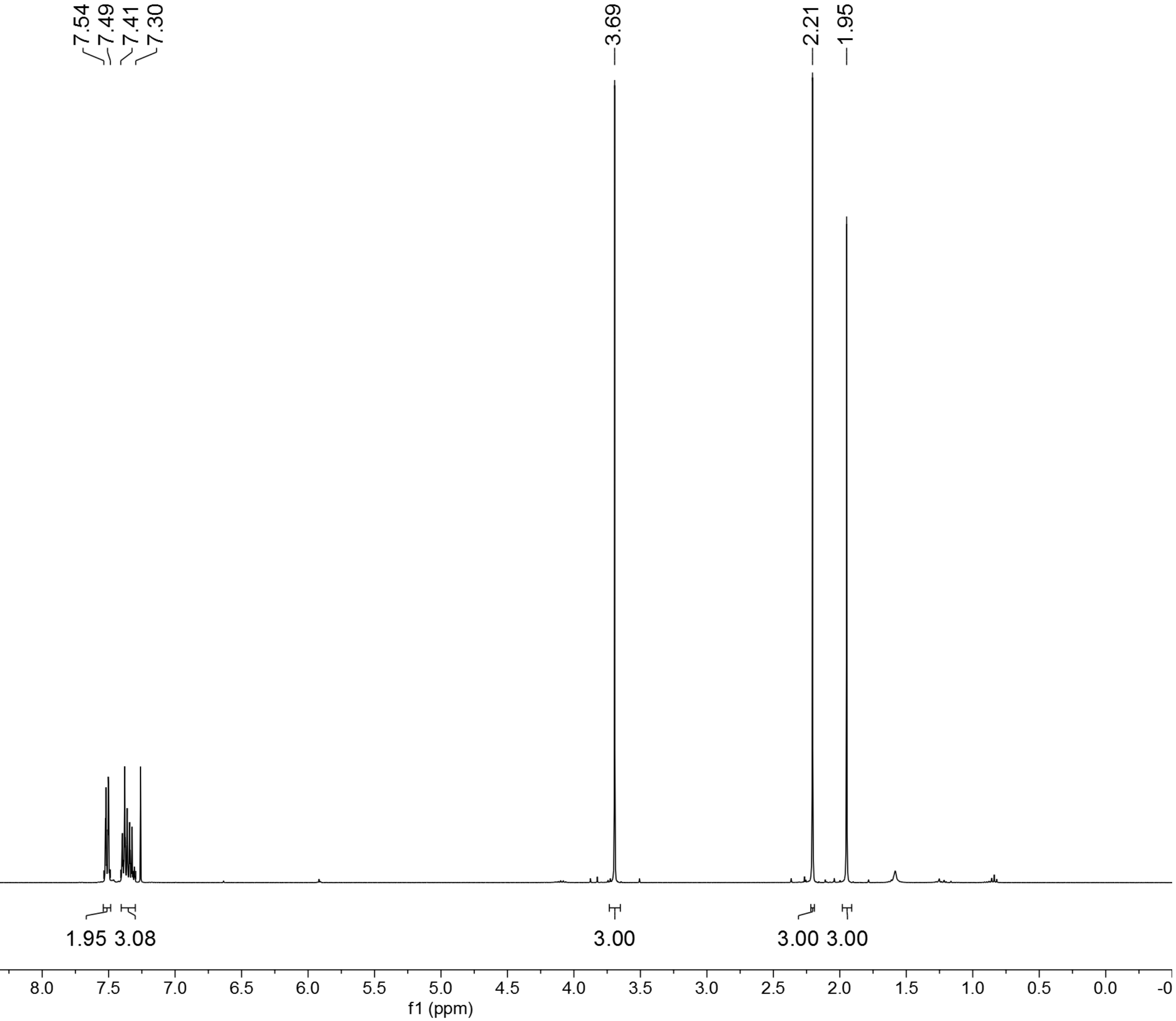

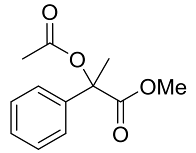

S25: ^{13}C, $CDCl_3$, 100 MHz

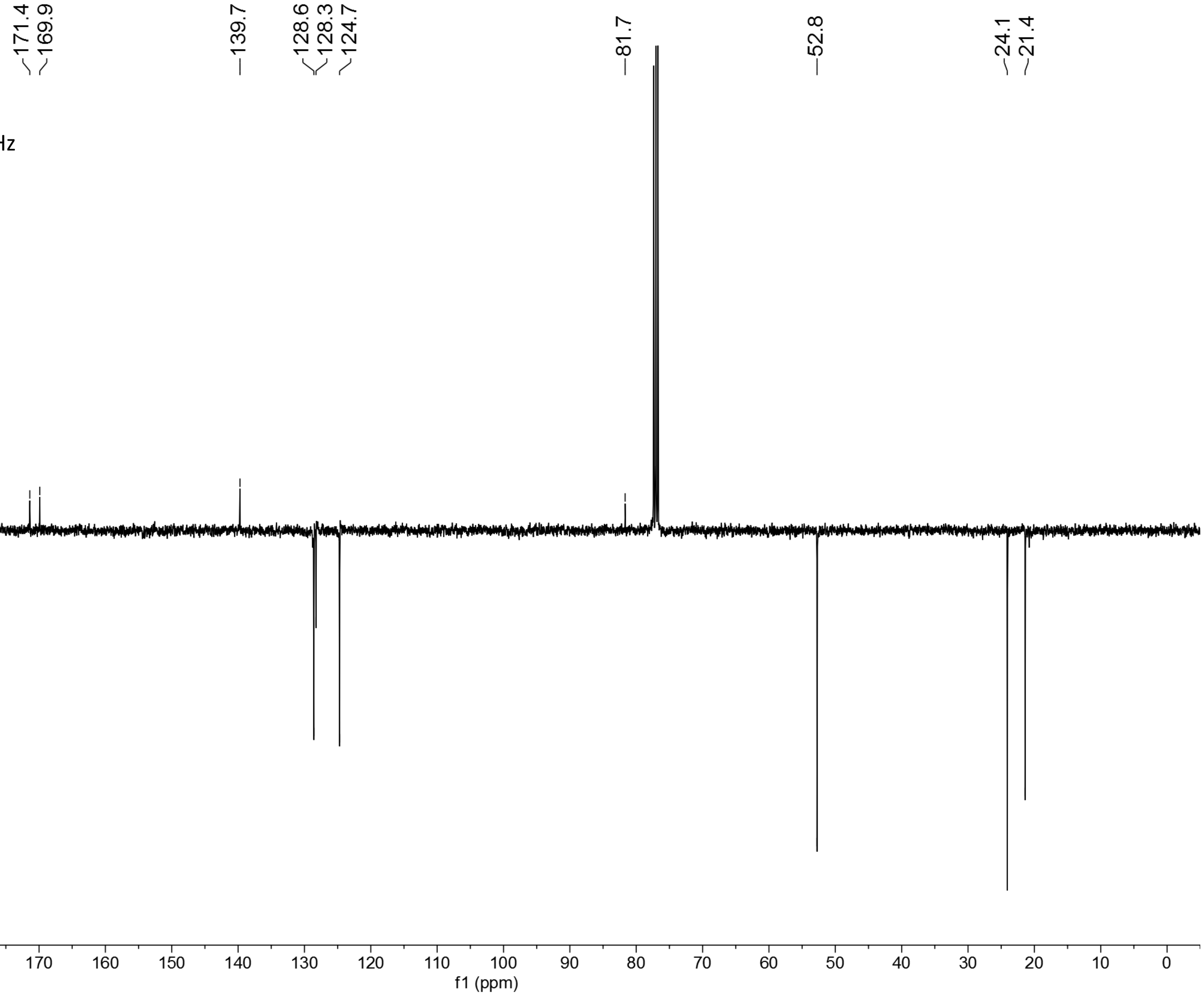

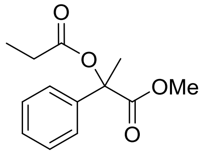

S26: ^{1}H, $CDCl_3$, 400 MHz

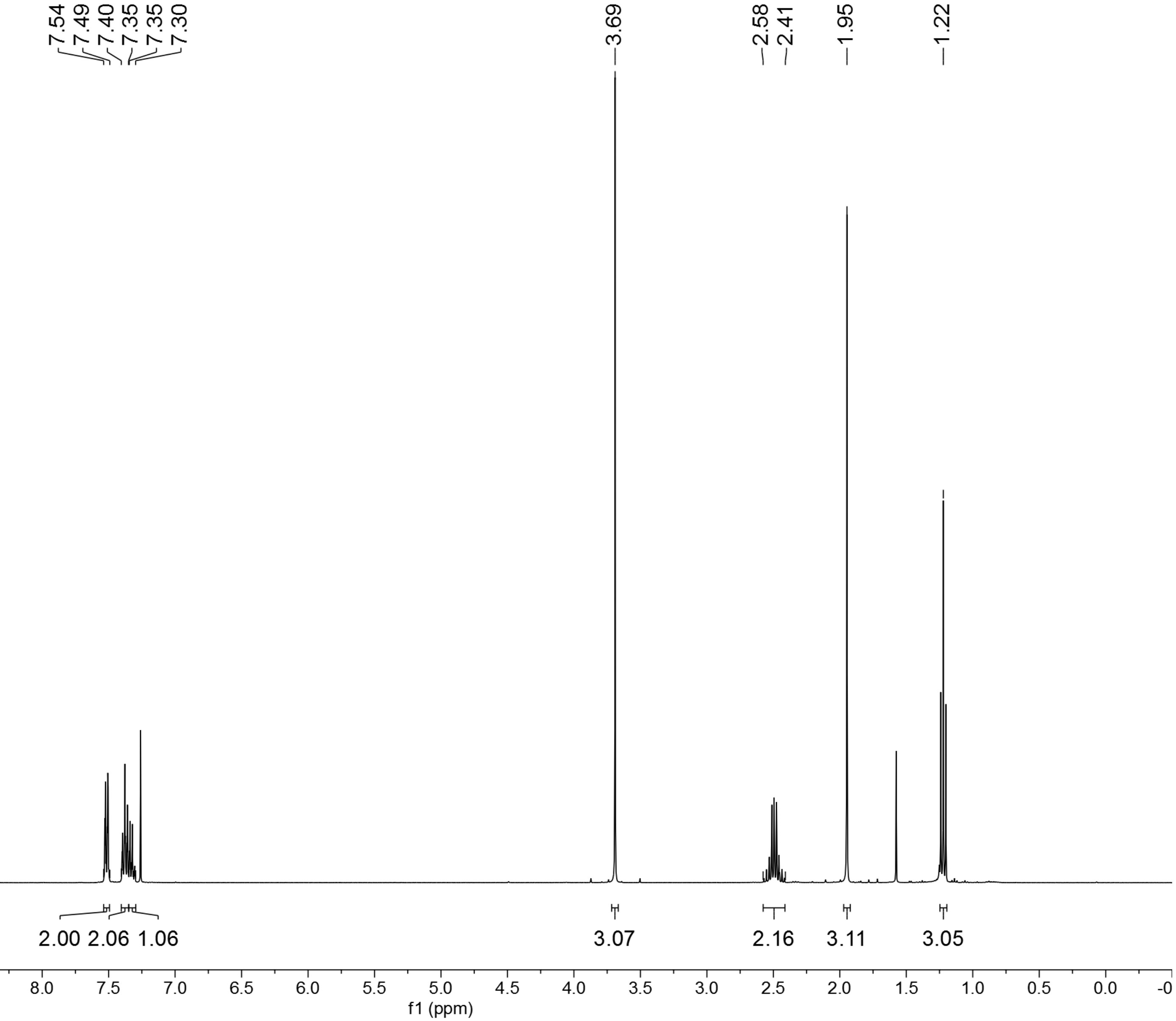

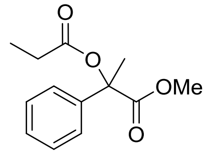

S26: ^{13}C, $CDCl_3$, 100 MHz

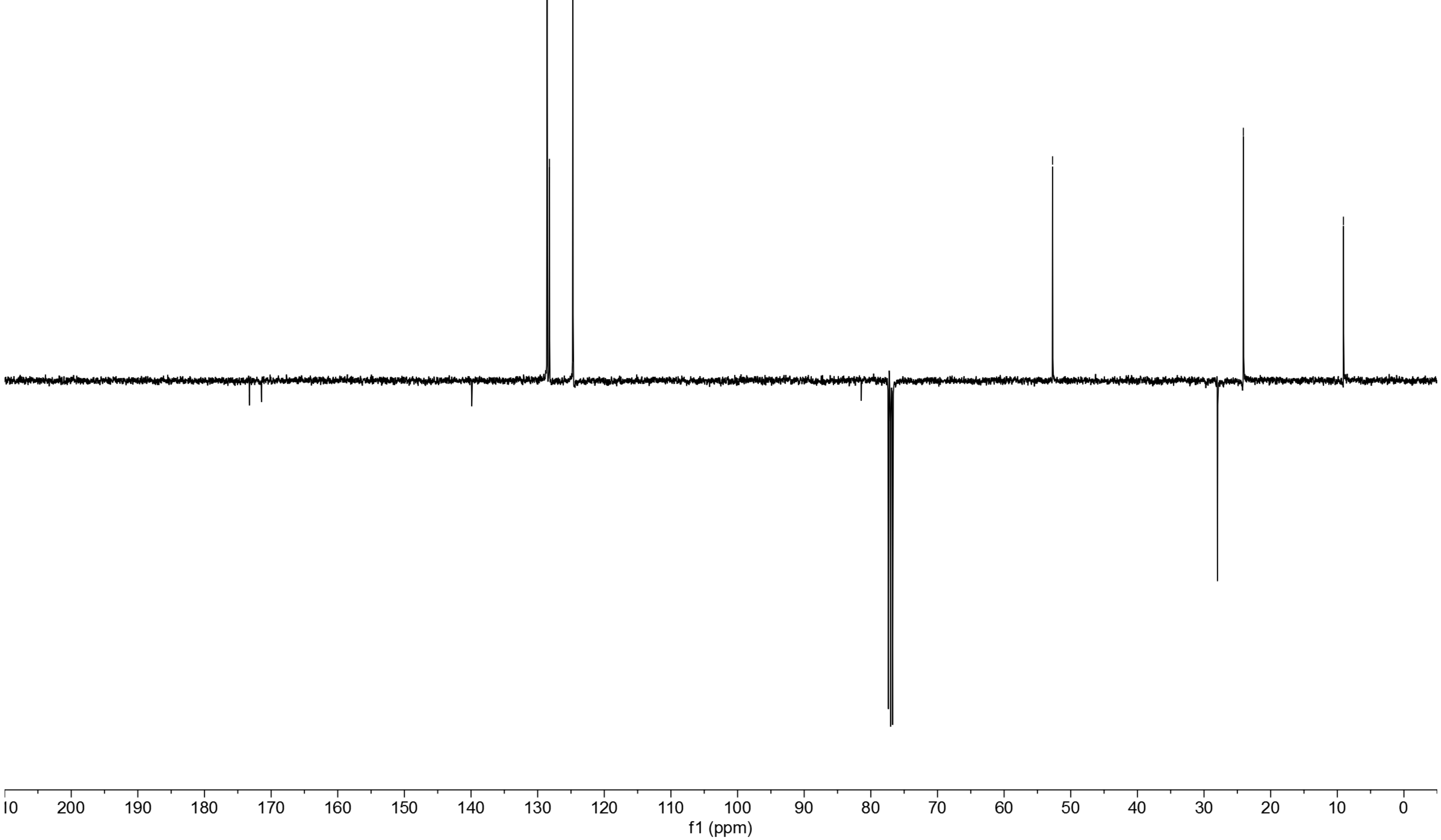

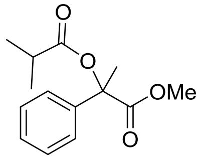

S27: ^{1}H, $CDCl_3$, 400 MHz

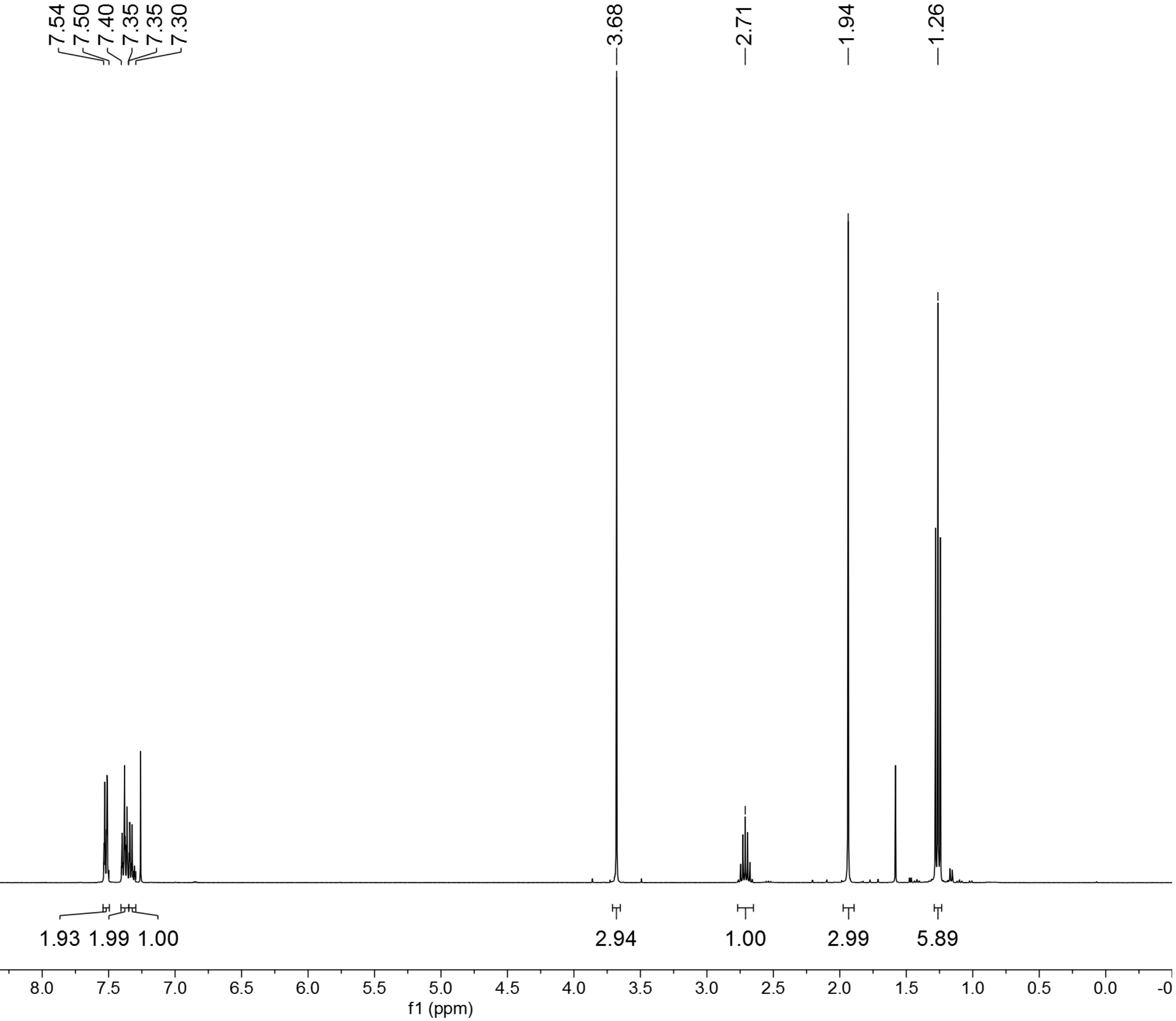

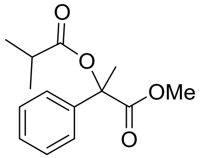

S27: ^{13}C, $CDCl_3$, 100 MHz

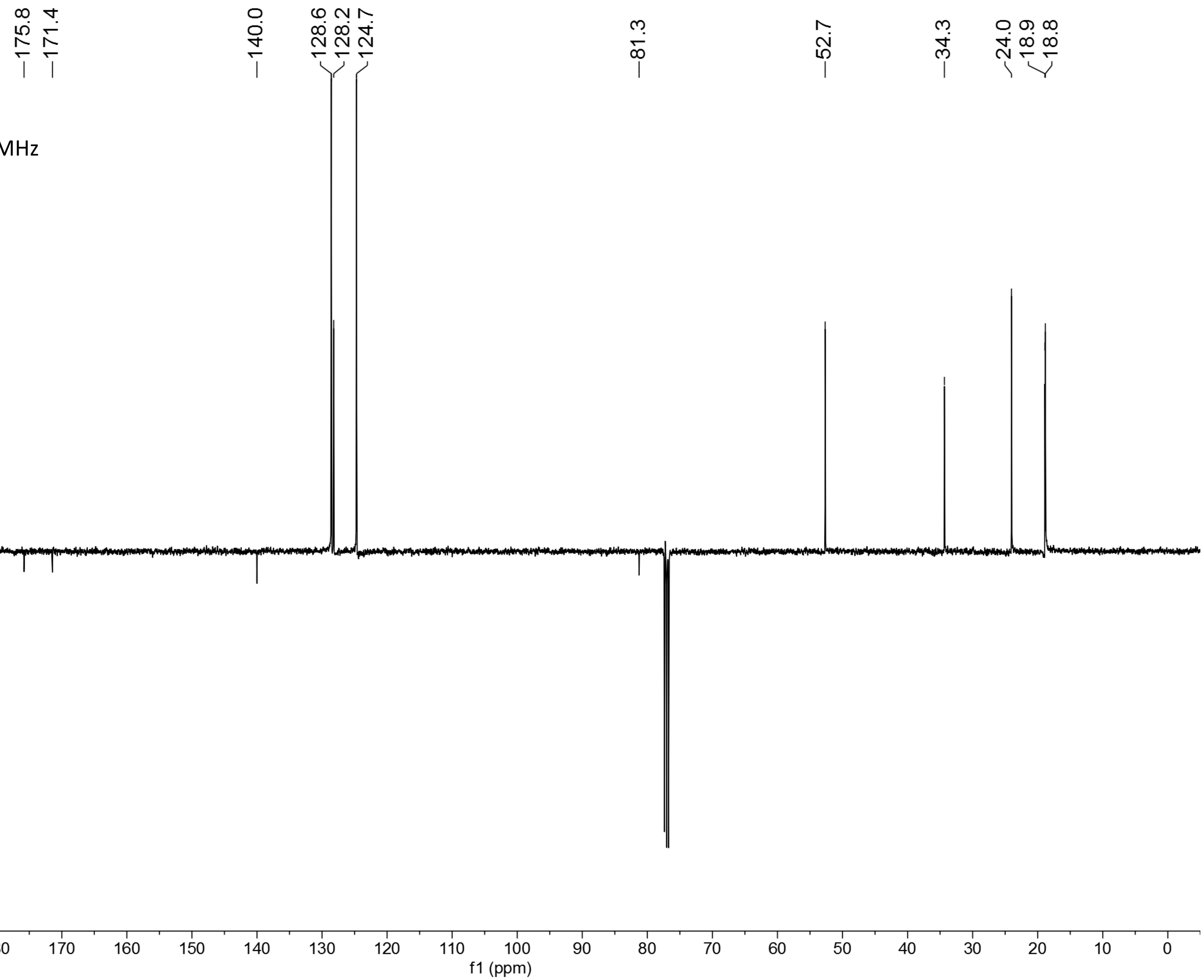

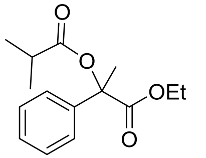

S28: ^{1}H, $CDCl_3$, 400 MHz

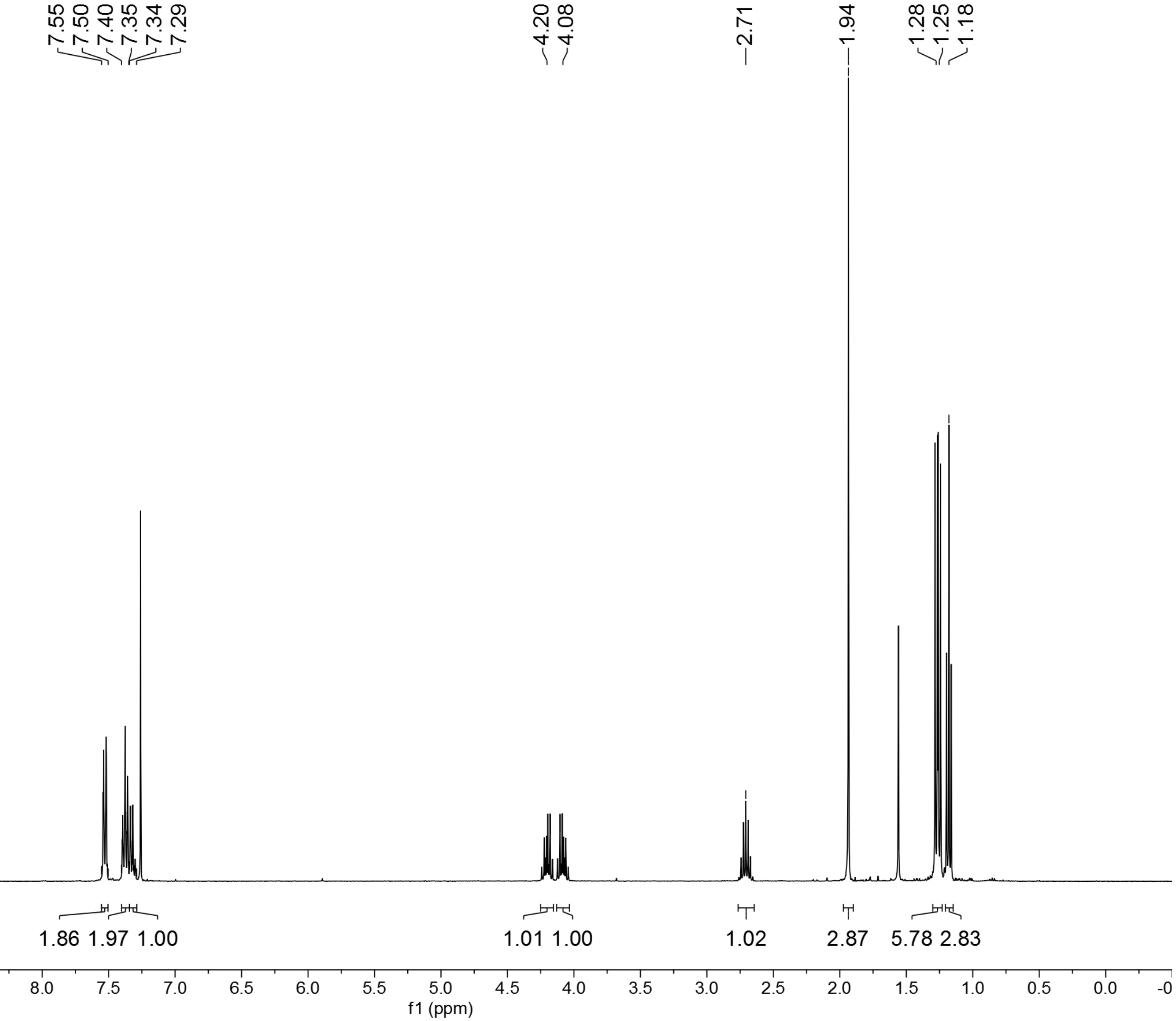

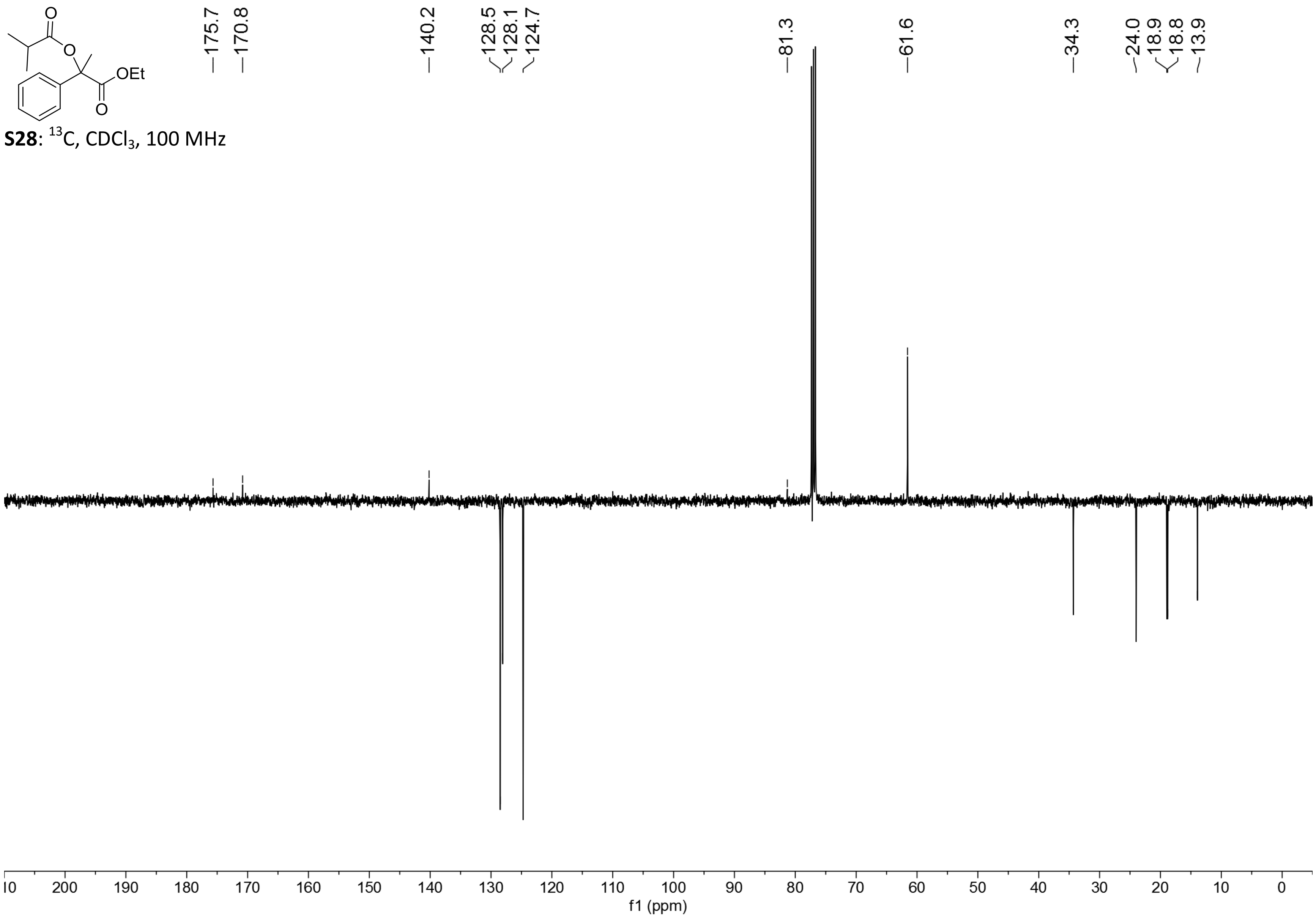

S28: ^{13}C, $CDCl_3$, 100 MHz

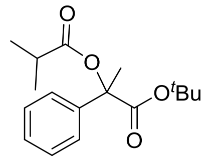

S29: [1]H, $CDCl_3$, 400 MHz

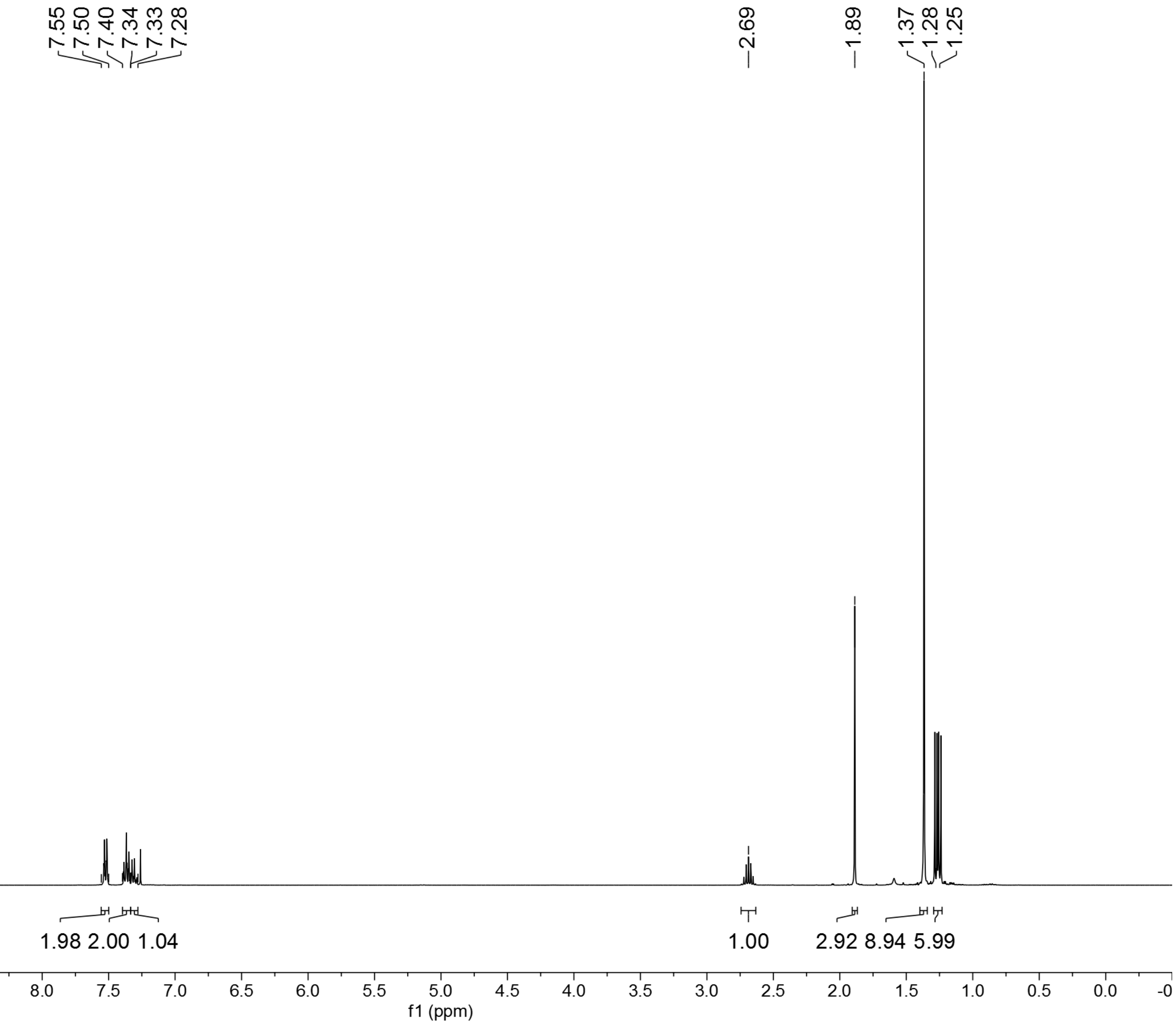

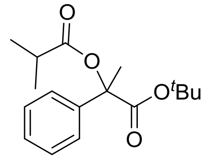

S29: ^{13}C, $CDCl_3$, 100 MHz

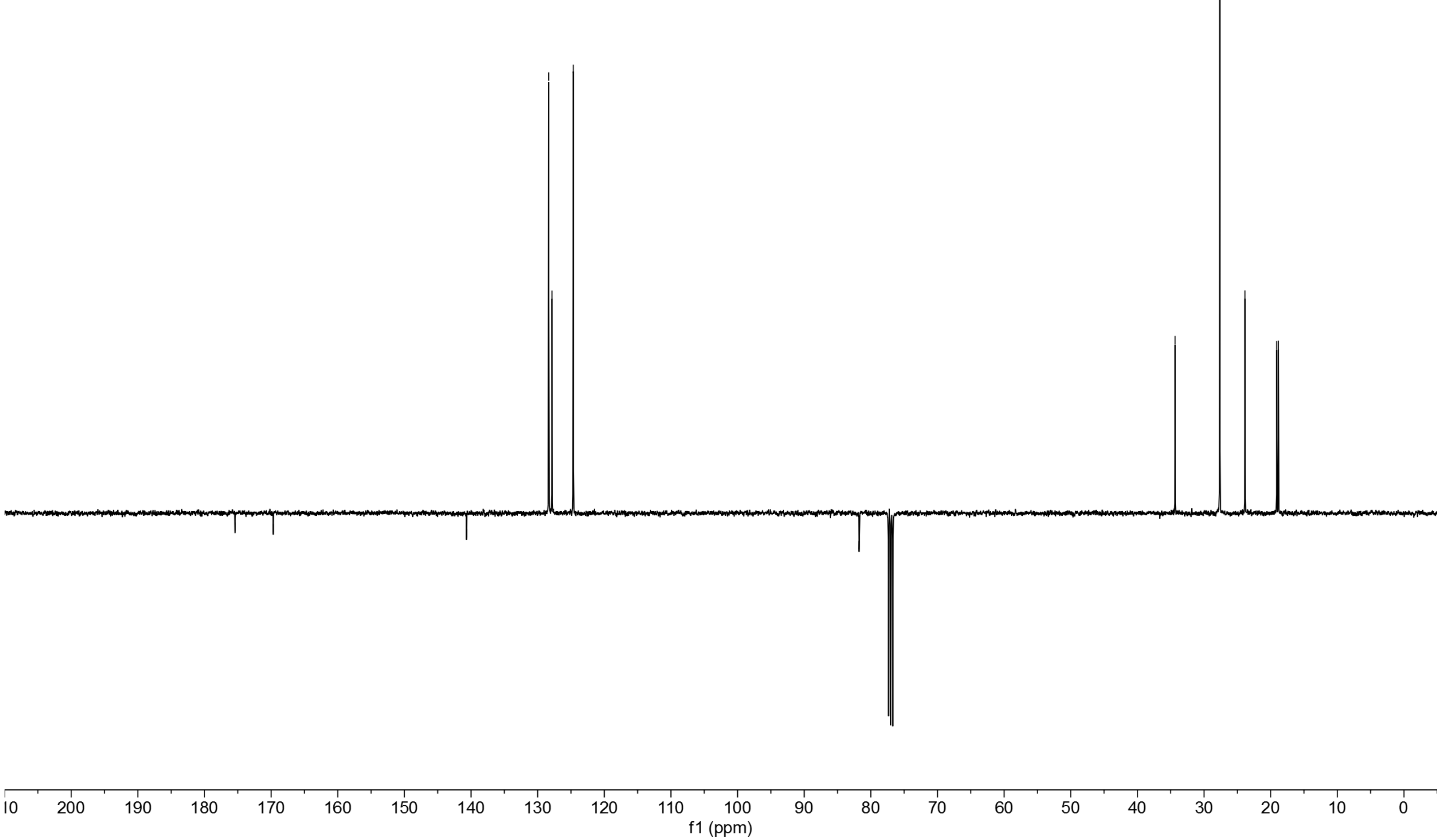

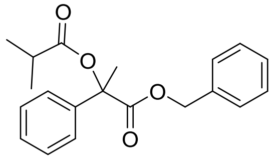

10: ^{1}H, $CDCl_3$, 400 MHz

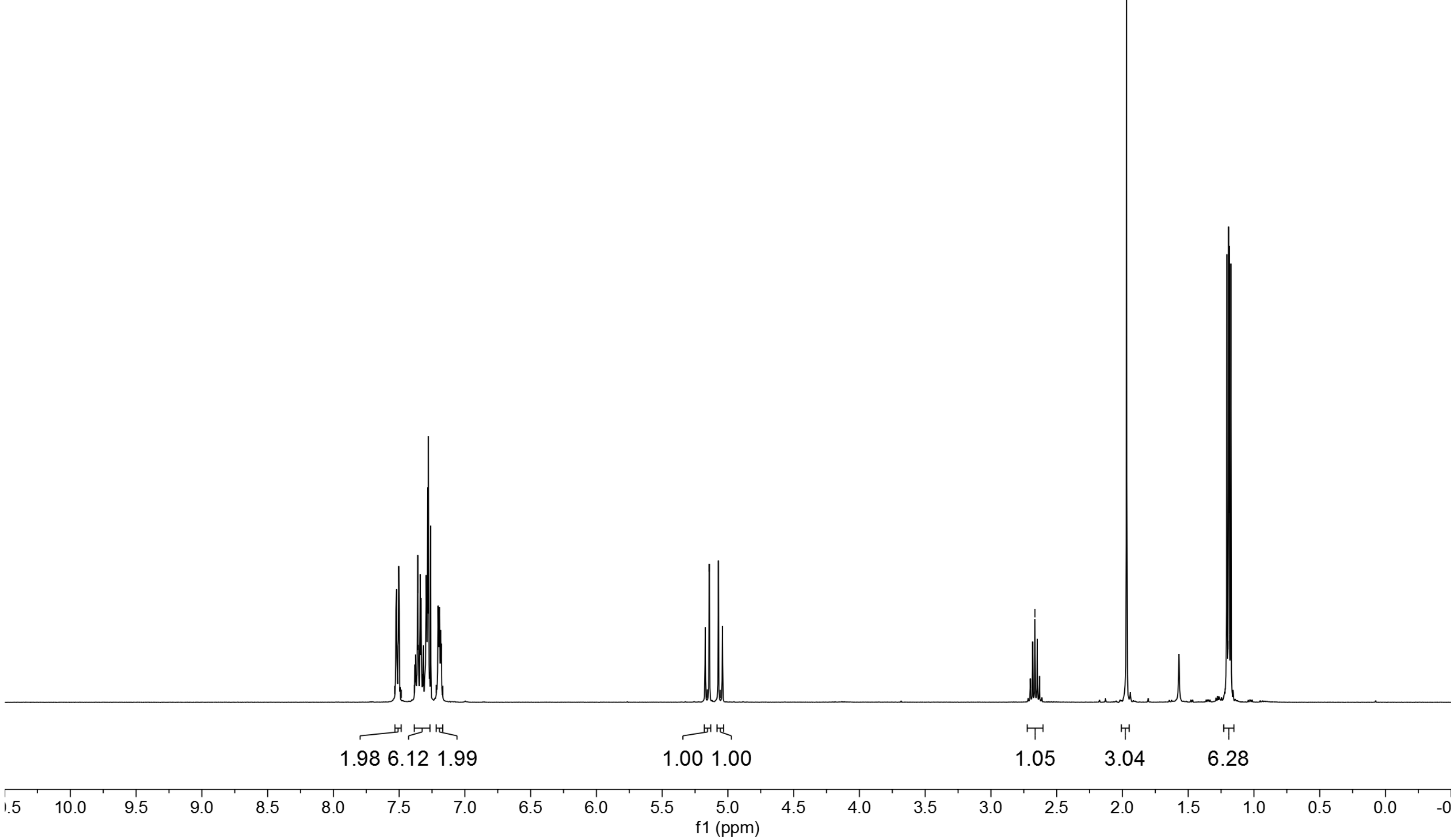

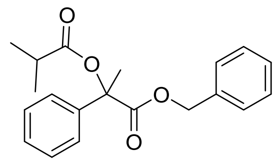

10: ^{13}C, $CDCl_3$, 100 MHz

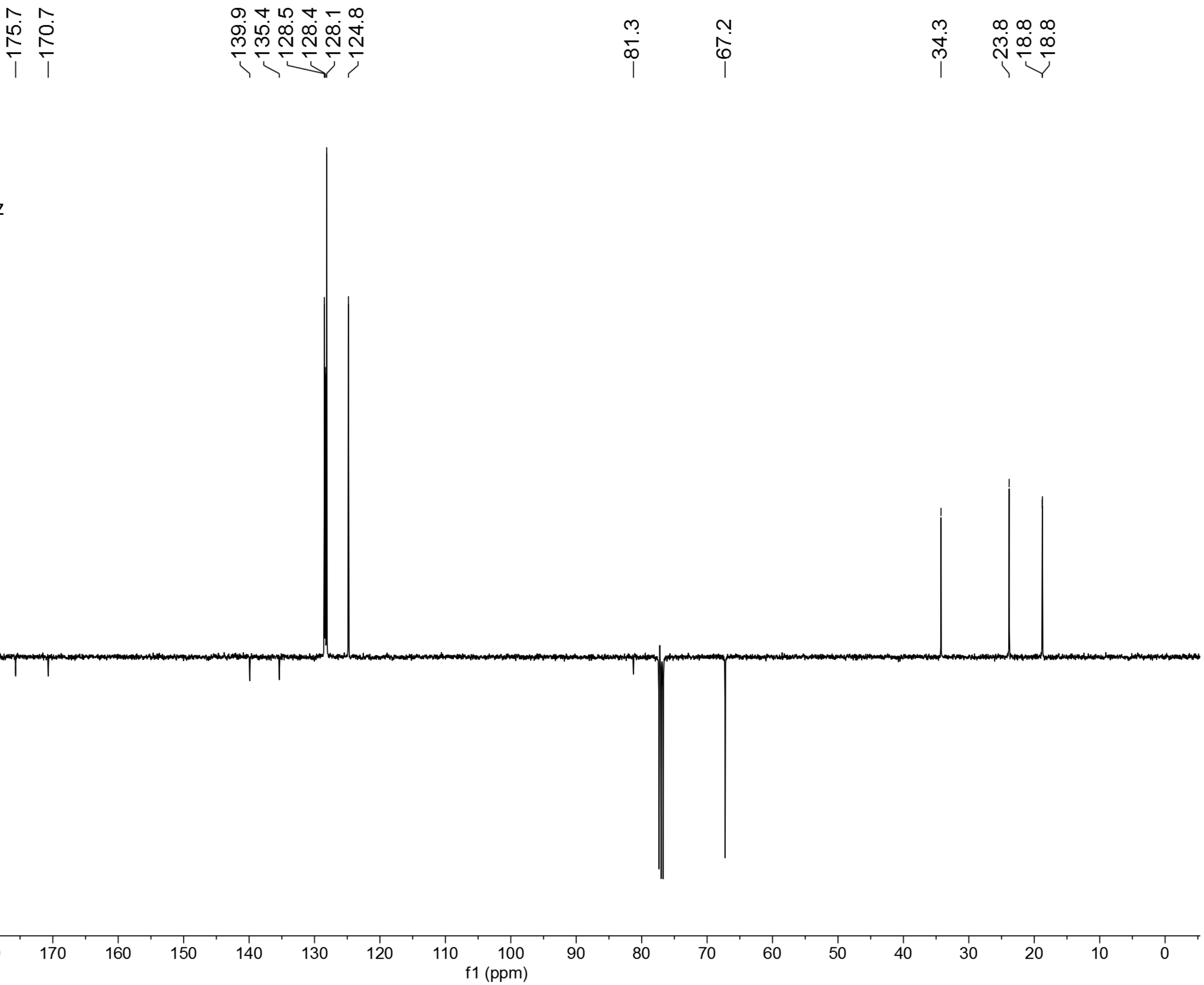

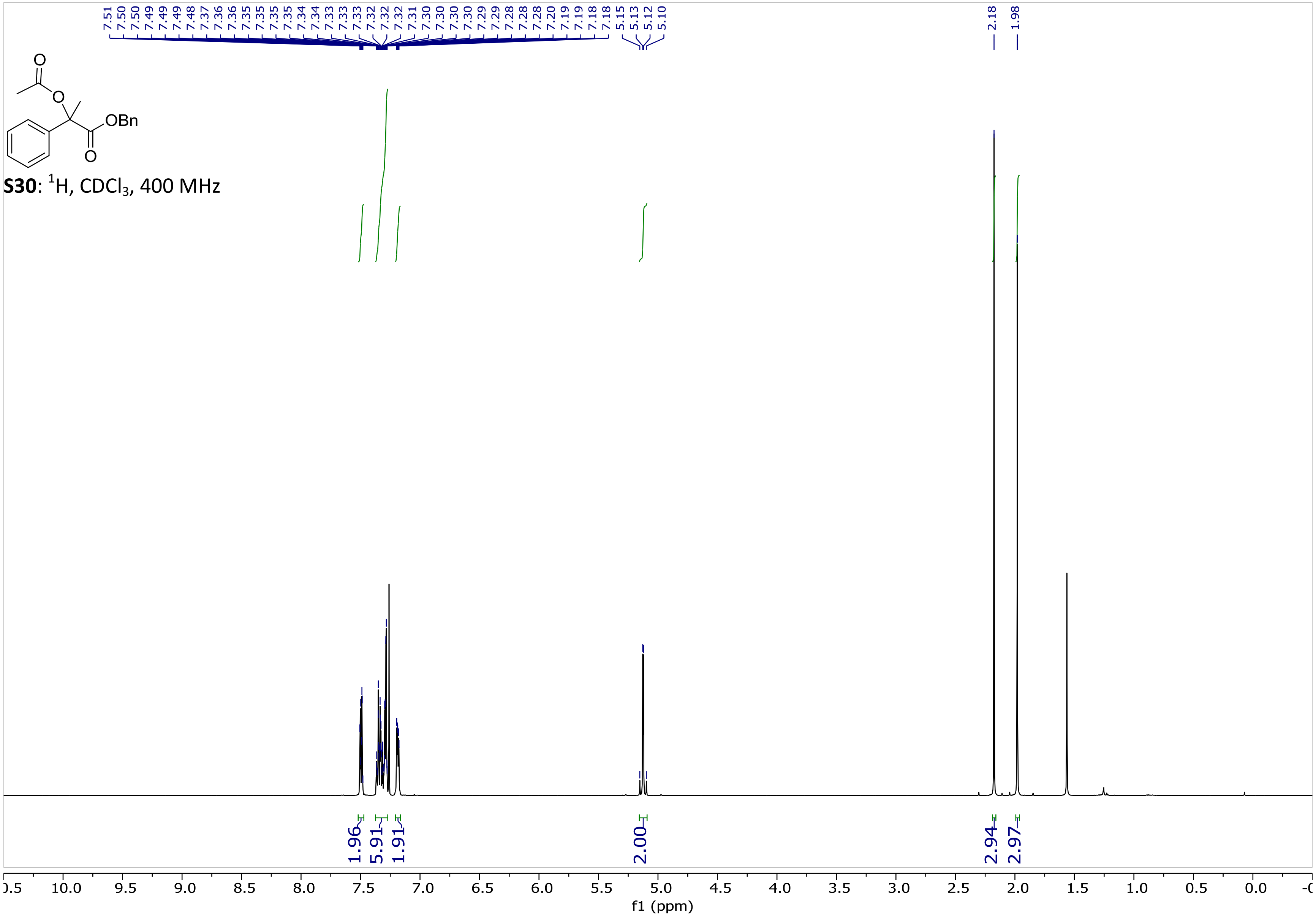

S30: [1]H, $CDCl_3$, 400 MHz

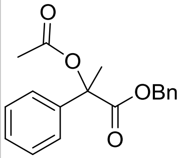

S30: ^{13}C, $CDCl_3$, 100 MHz

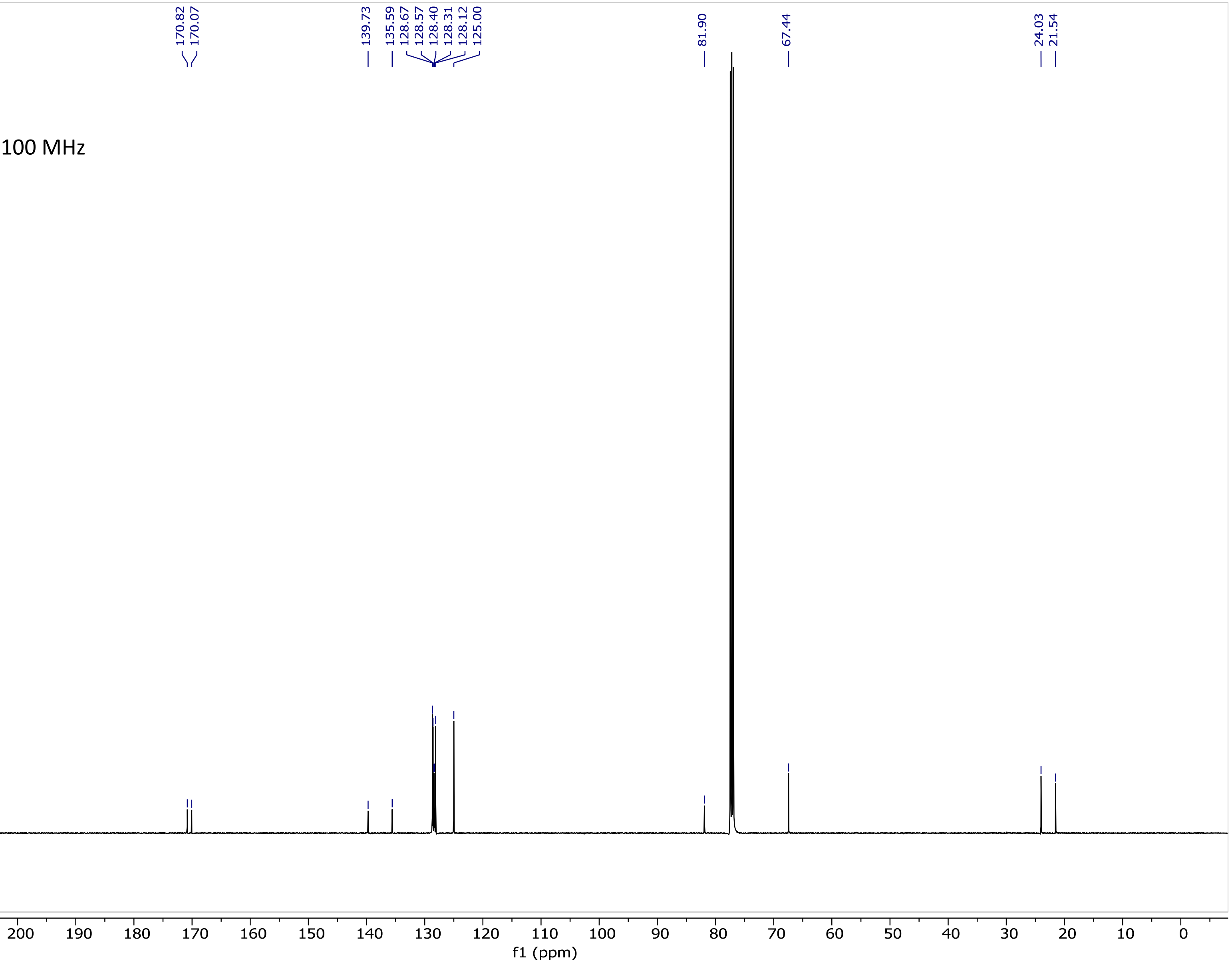

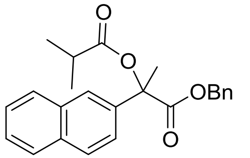

S31: ^{1}H, $CDCl_3$, 400 MHz

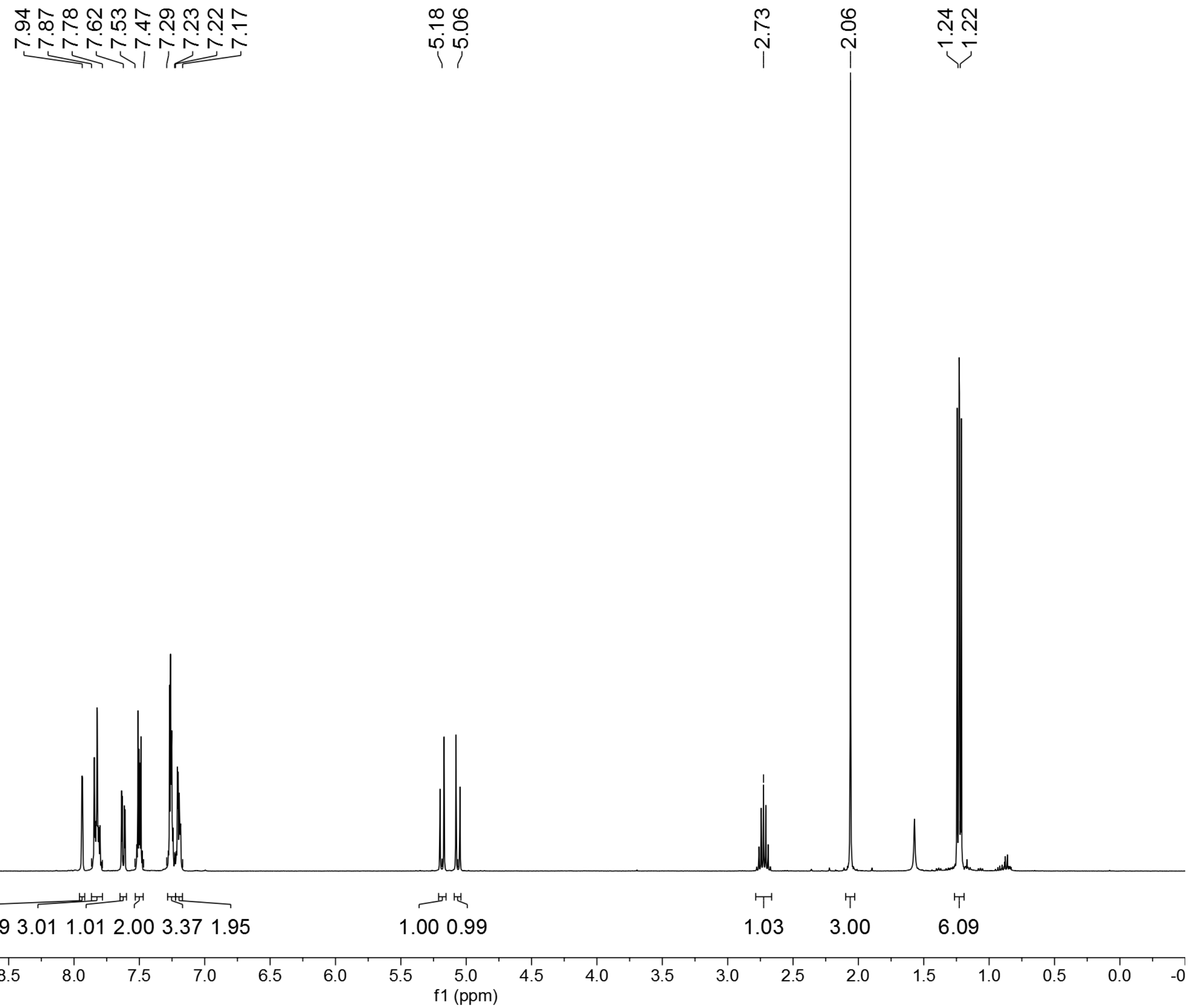

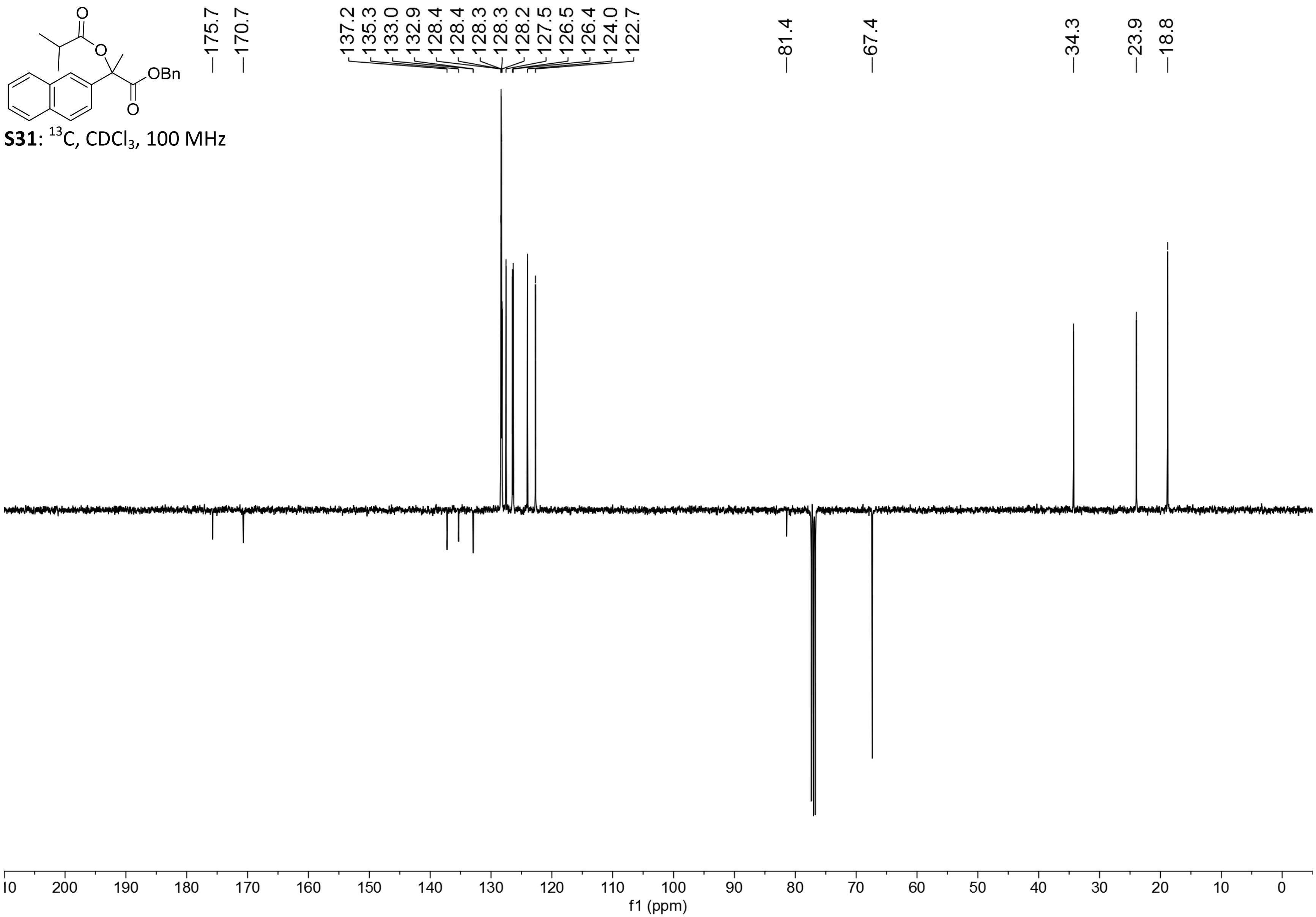
OBn
O
O
O
S31: ^{13}C, CDCl$_3$, 100 MHz
175.7
170.7
137.2
135.3
133.0
132.9
128.4
128.4
128.3
128.3
128.2
127.5
126.5
126.4
124.0
122.7
81.4
67.4
34.3
23.9
18.8
210
200
190
180
170
160
150
140
130
120
110
100
90
80
70
60
50
40
30
20
10
0
f1 (ppm)

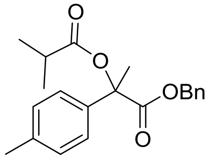

S32: ^{1}H, $CDCl_3$, 400 MHz

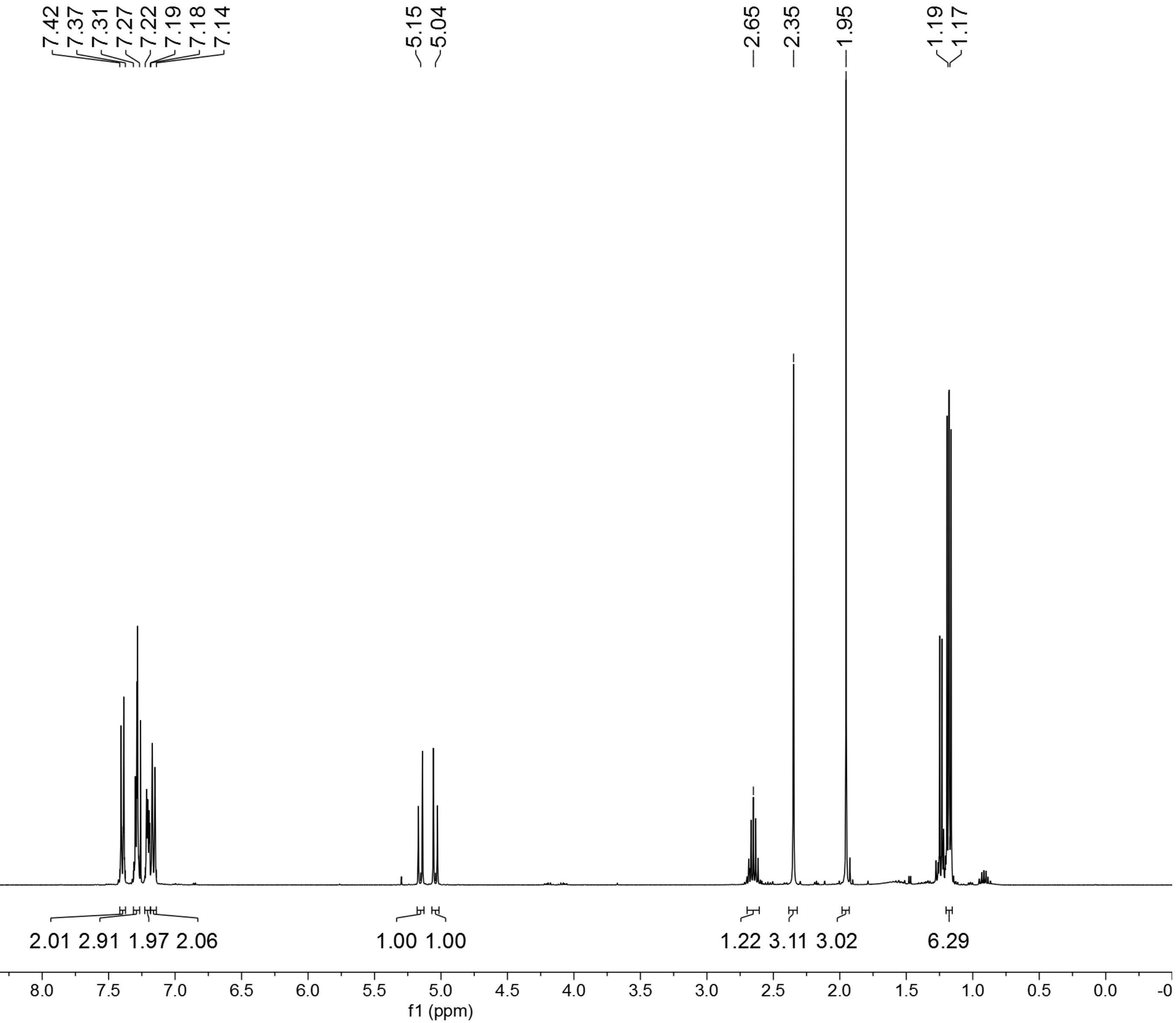

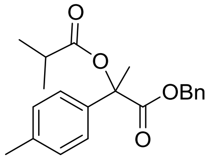

S32: ^{13}C, $CDCl_3$, 100 MHz

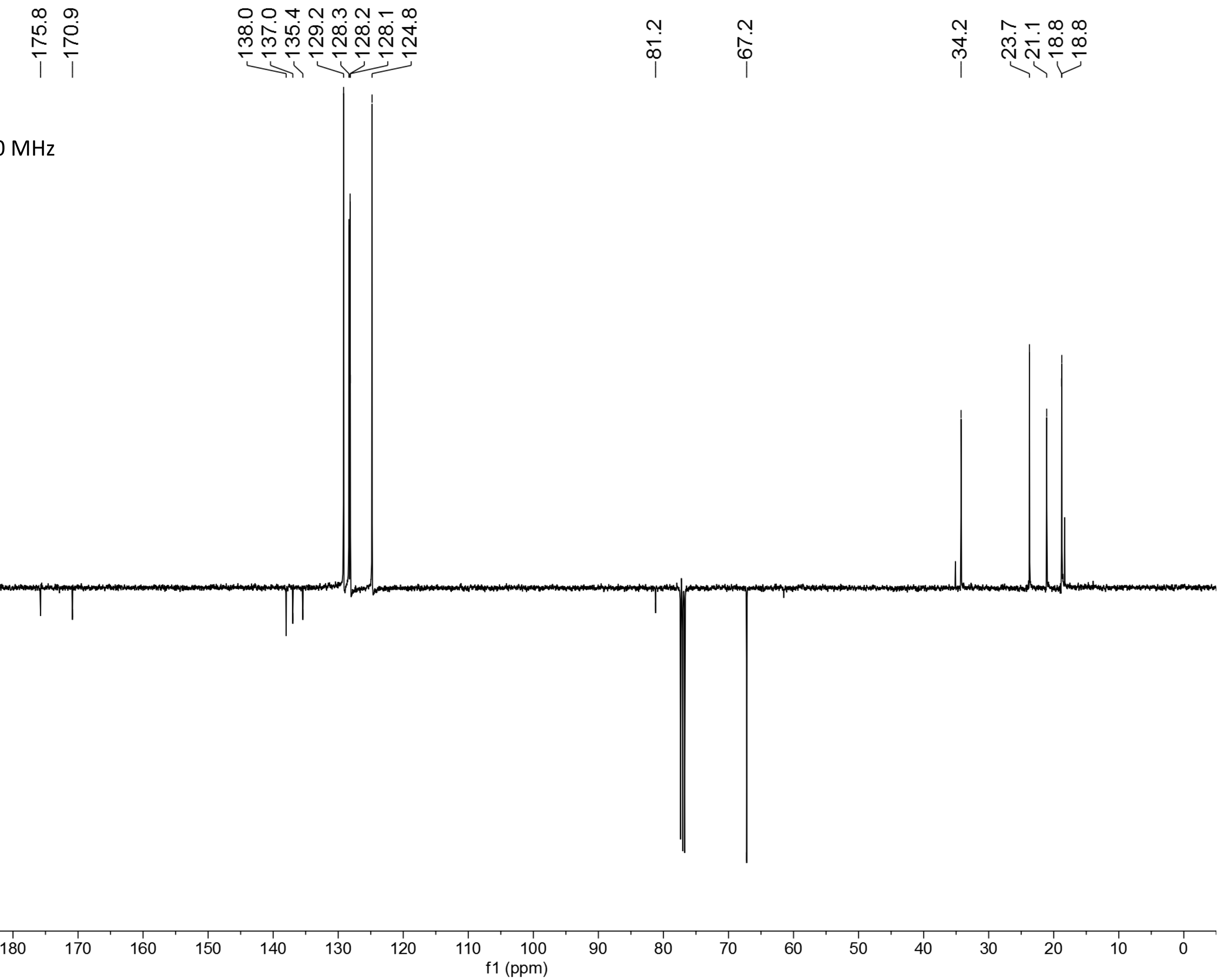

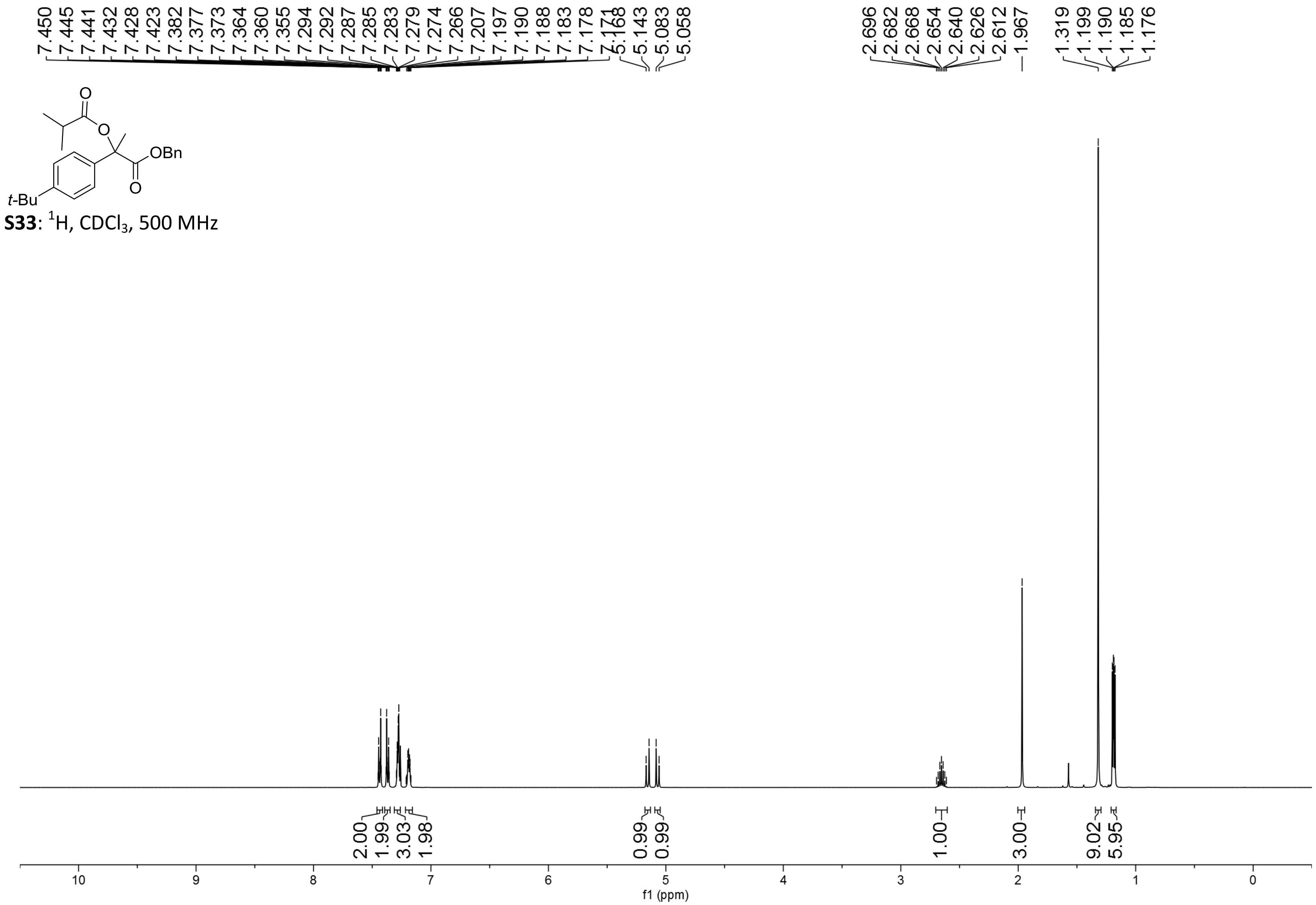

S33: ^{1}H, $CDCl_3$, 500 MHz

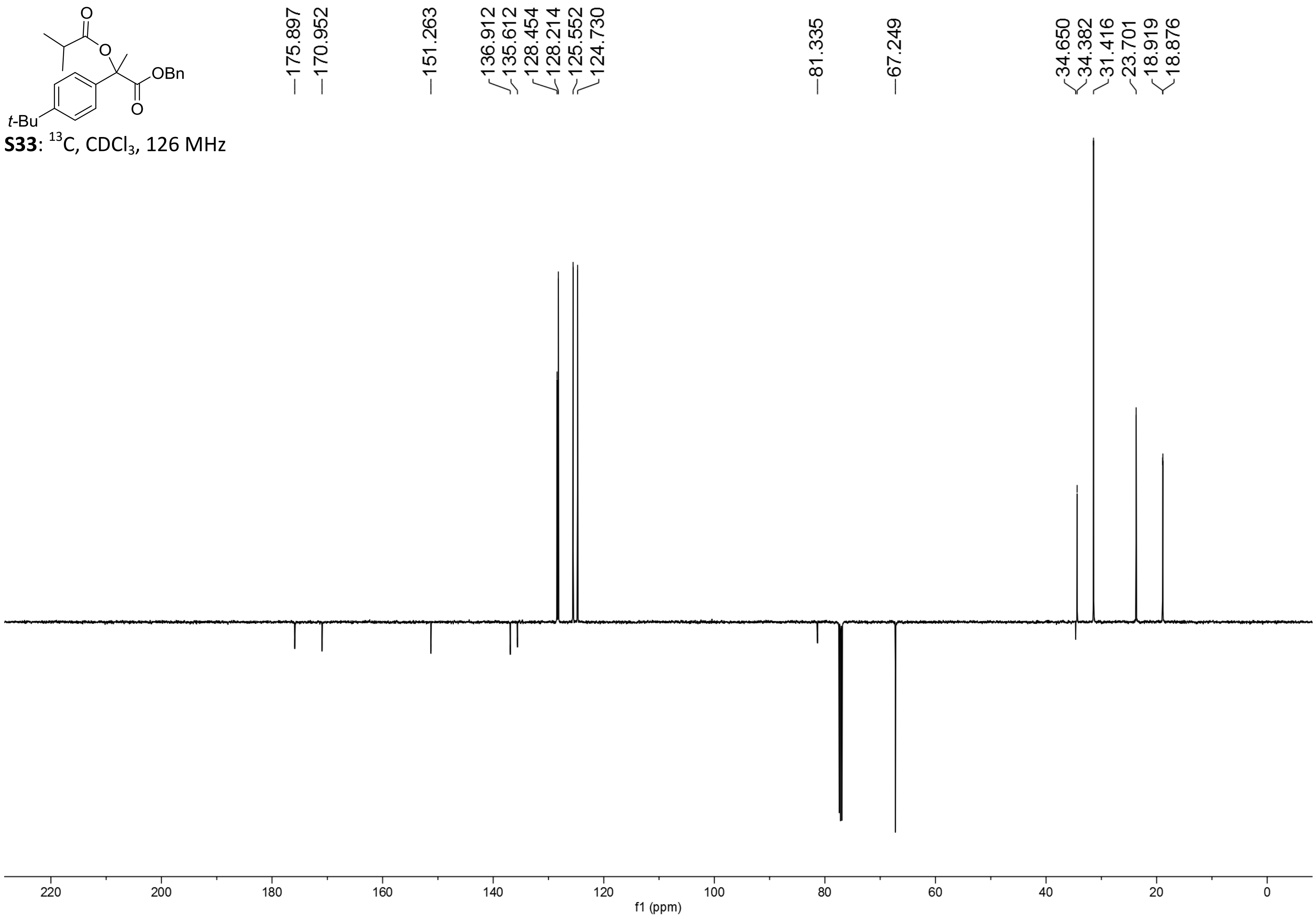

S33: ^{13}C, $CDCl_3$, 126 MHz

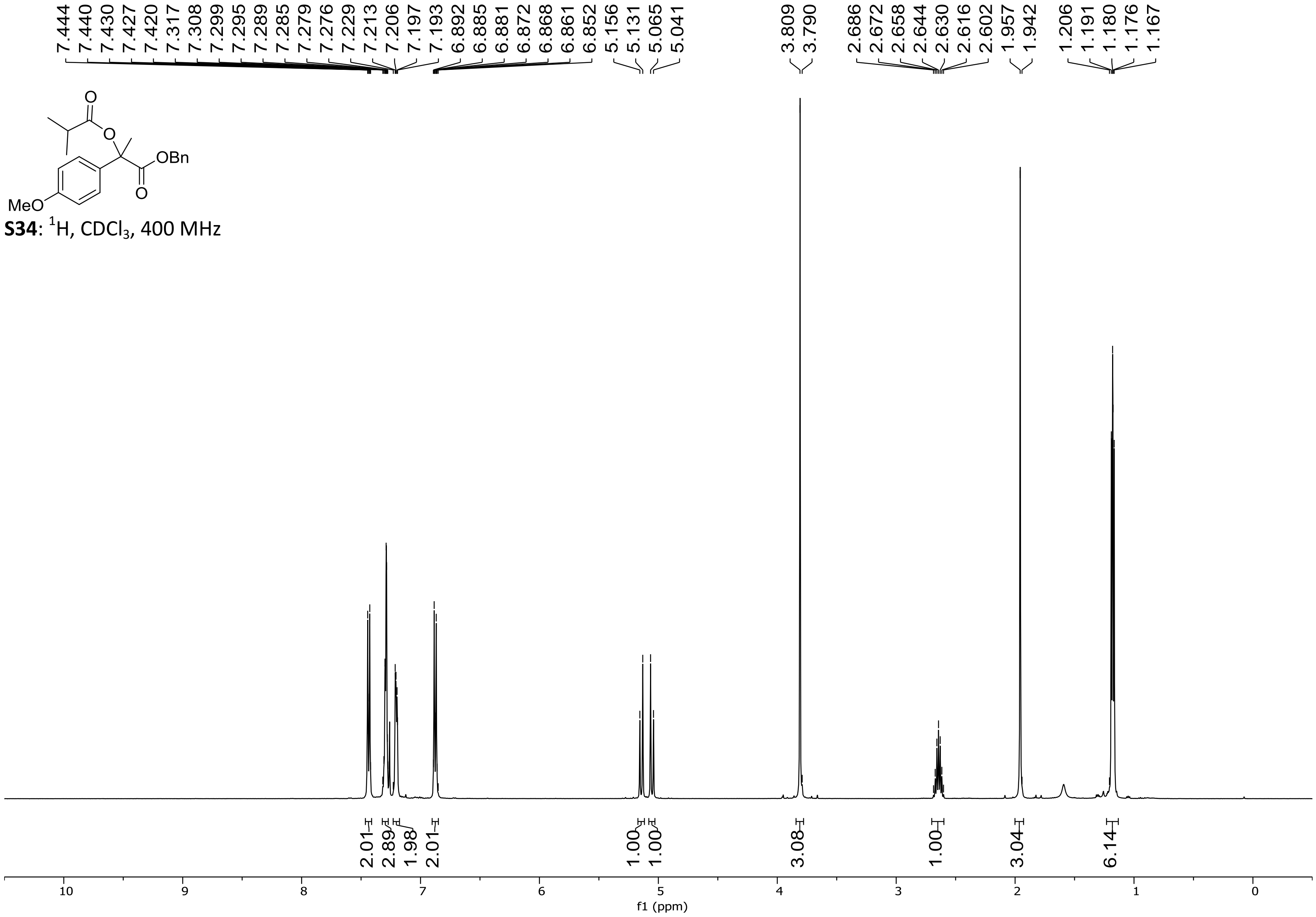

S34: ^{1}H, $CDCl_3$, 400 MHz

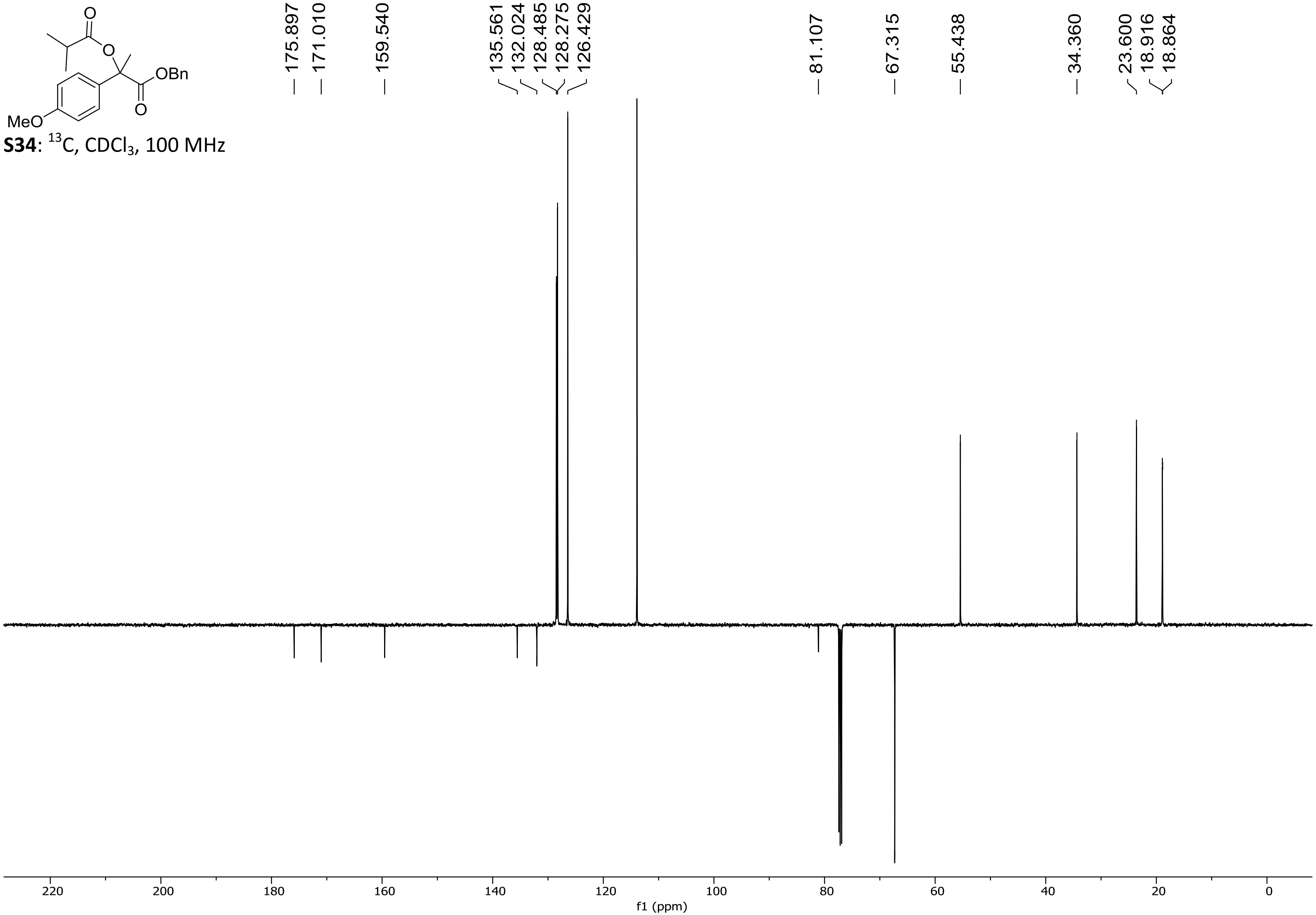

S34: ^{13}C, $CDCl_3$, 100 MHz

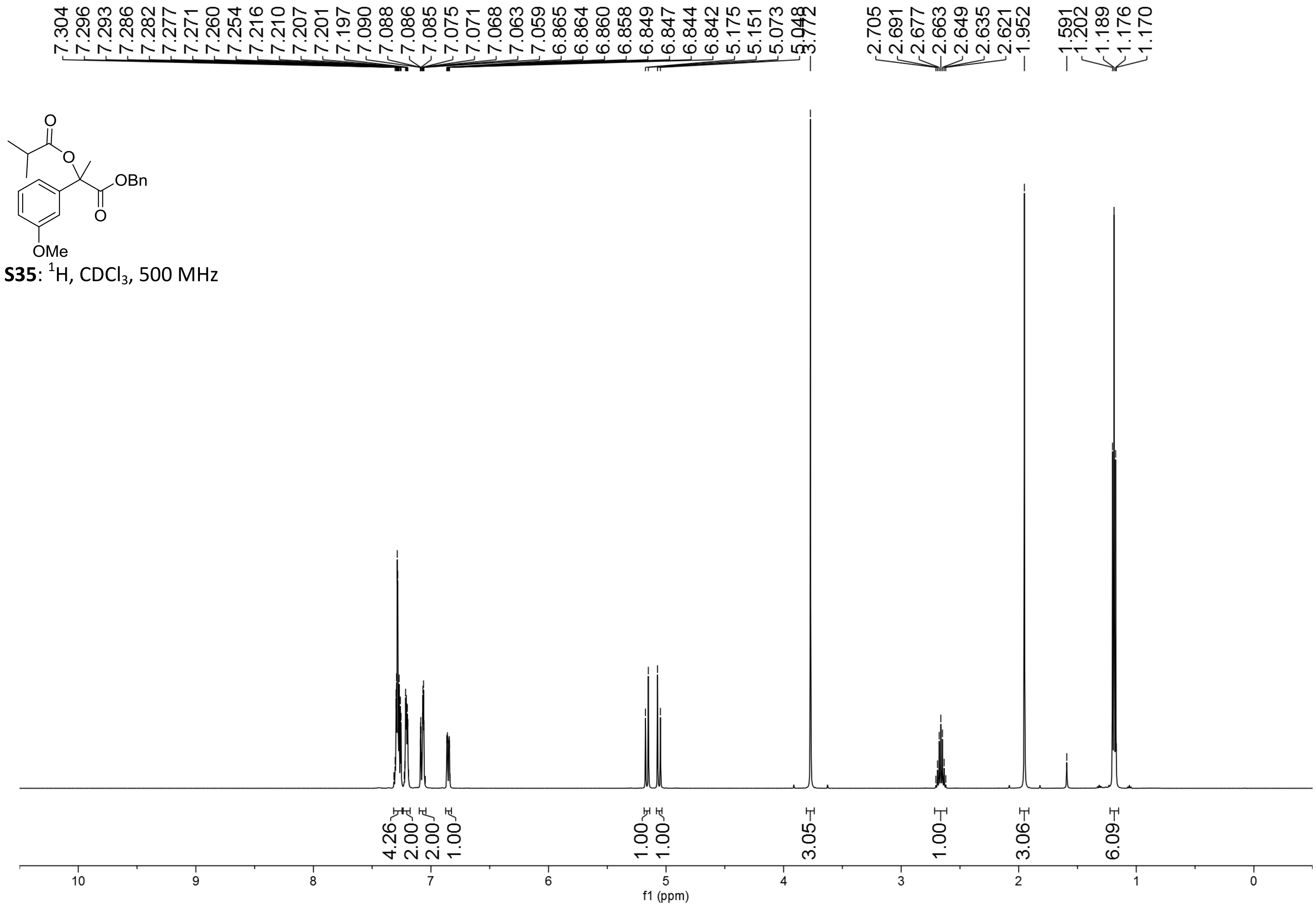

S35: ^{1}H, $CDCl_3$, 500 MHz

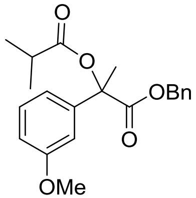

S35: ^{13}C, $CDCl_3$, 126 MHz

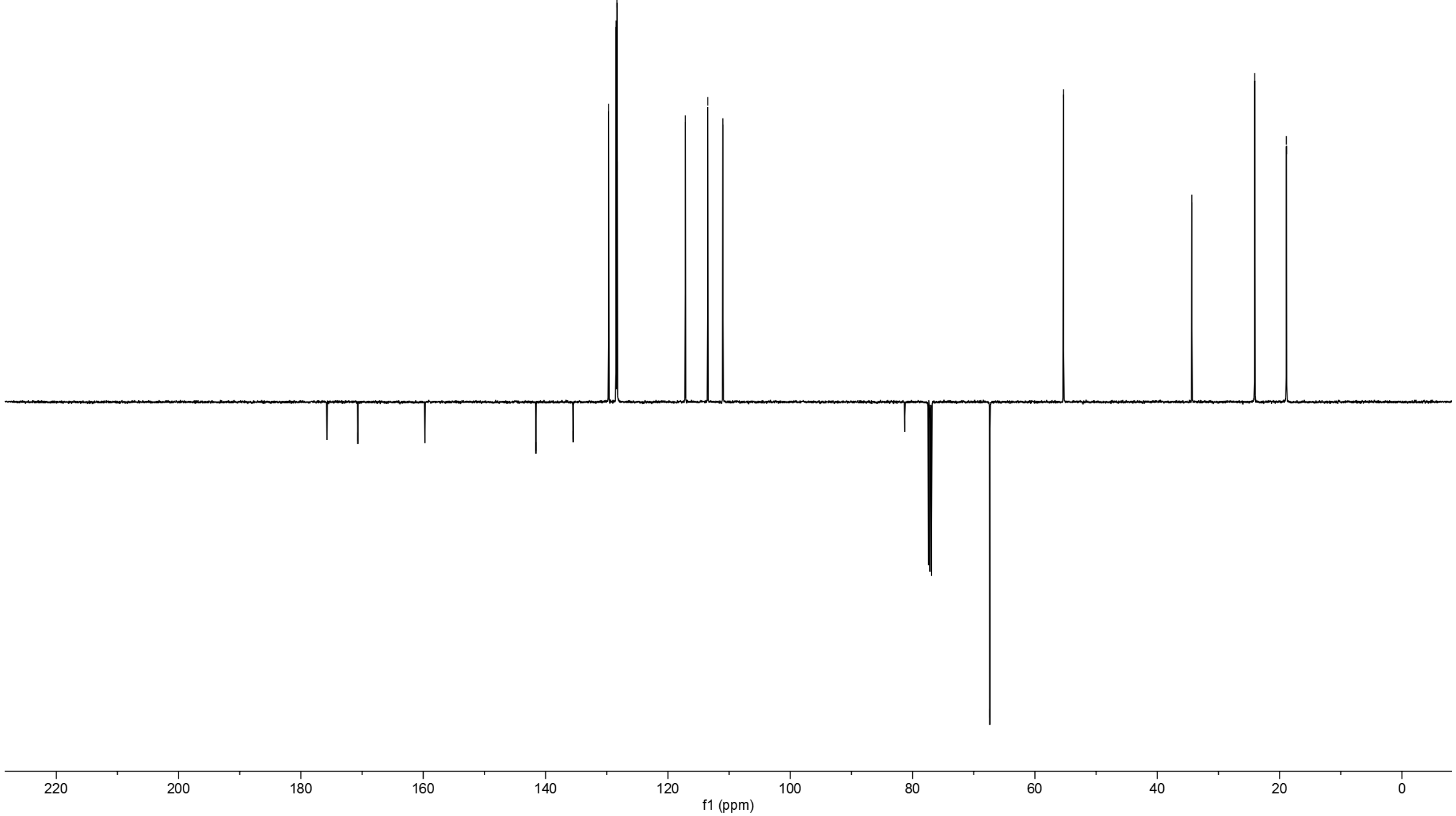

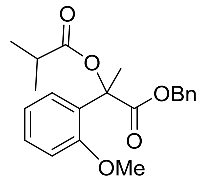

S36: ^{1}H, $CDCl_3$, 500 MHz

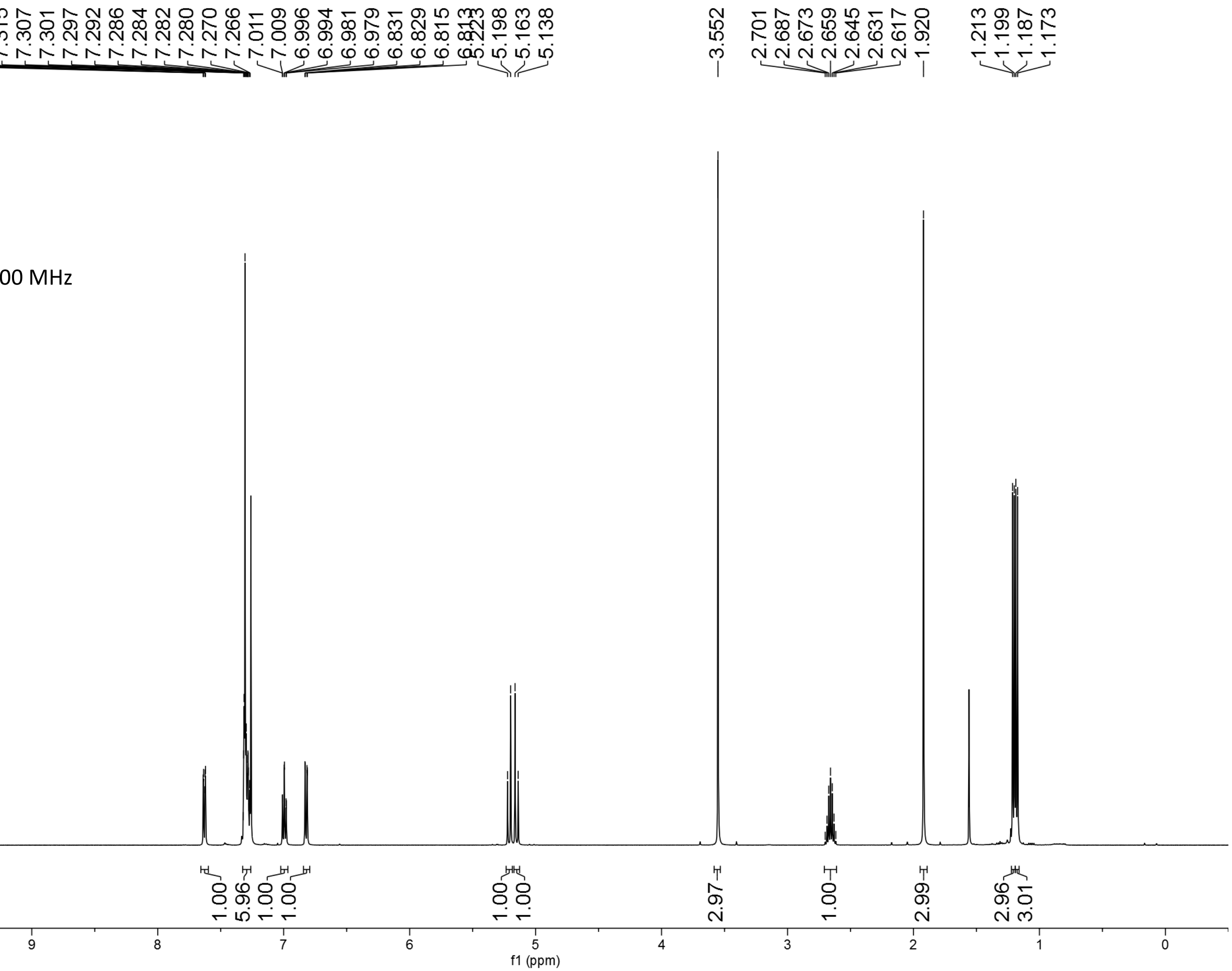

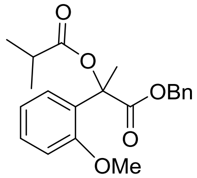

S36: ^{13}C, $CDCl_3$, 126 MHz

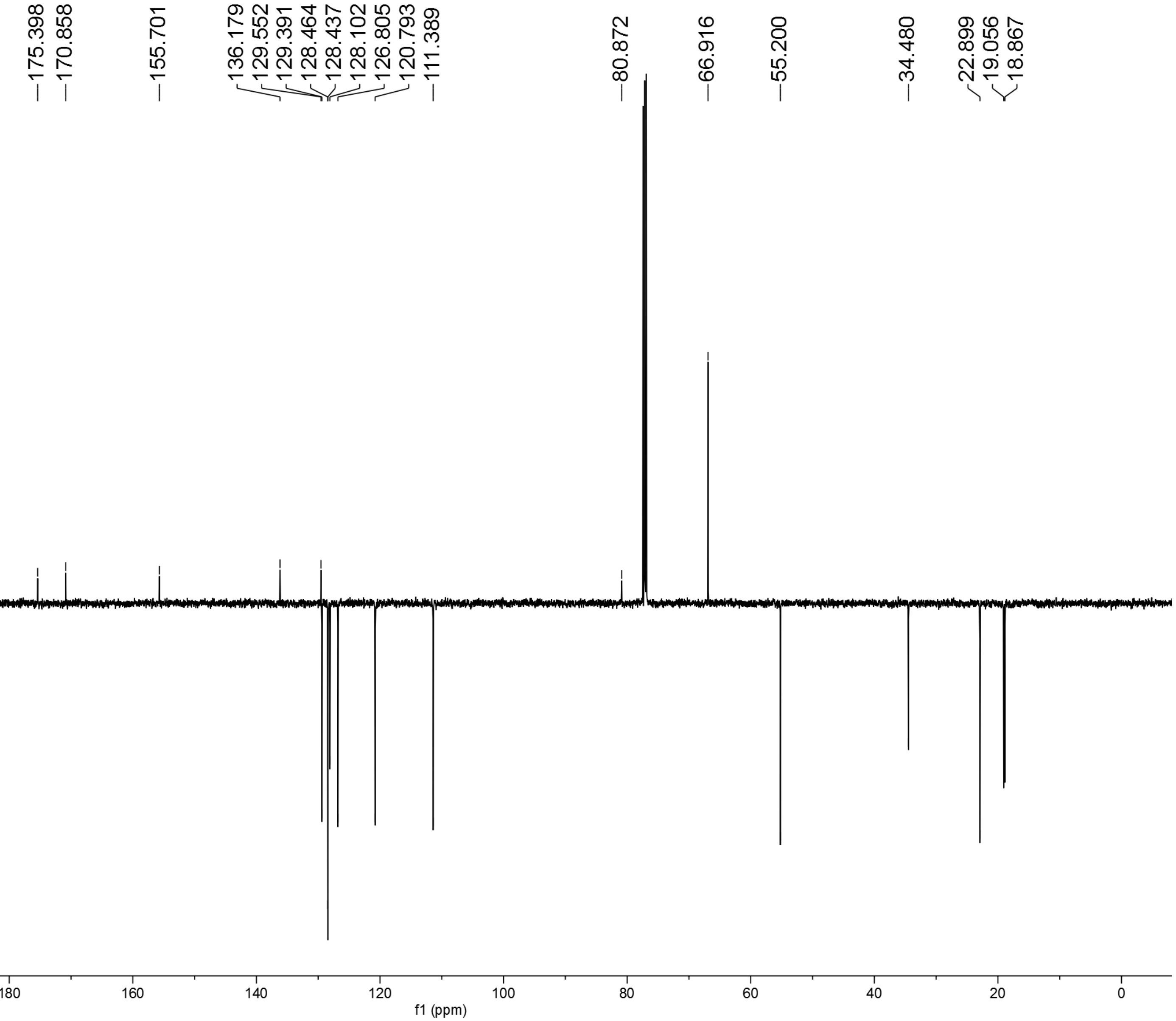

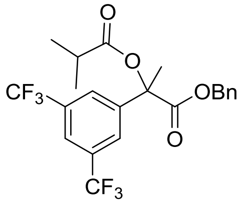

S37: ^{1}H, $CDCl_3$, 400 MHz

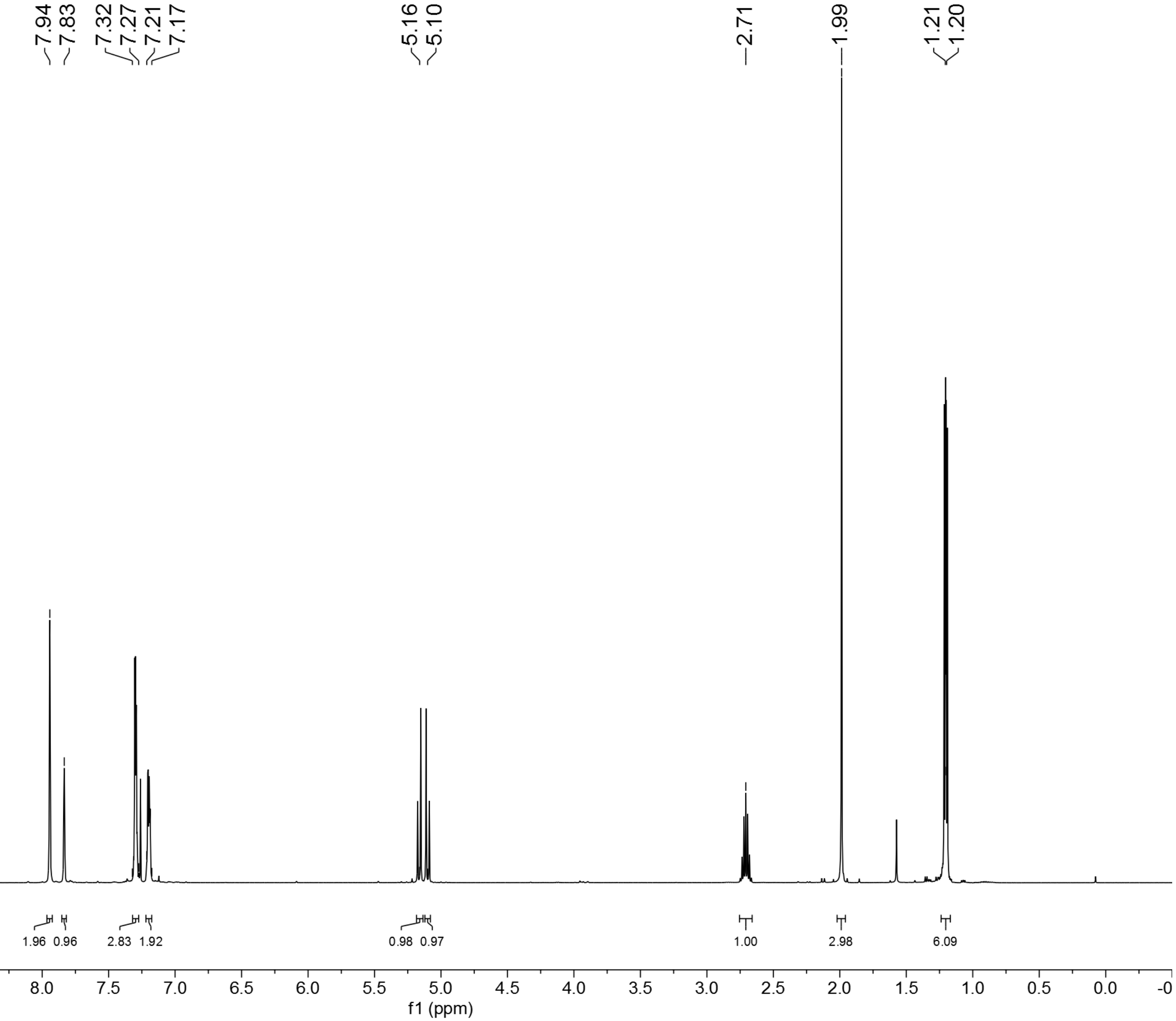

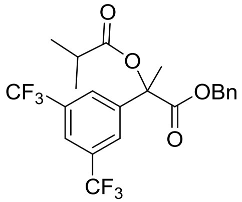

S37: ^{13}C, $CDCl_3$, 100 MHz

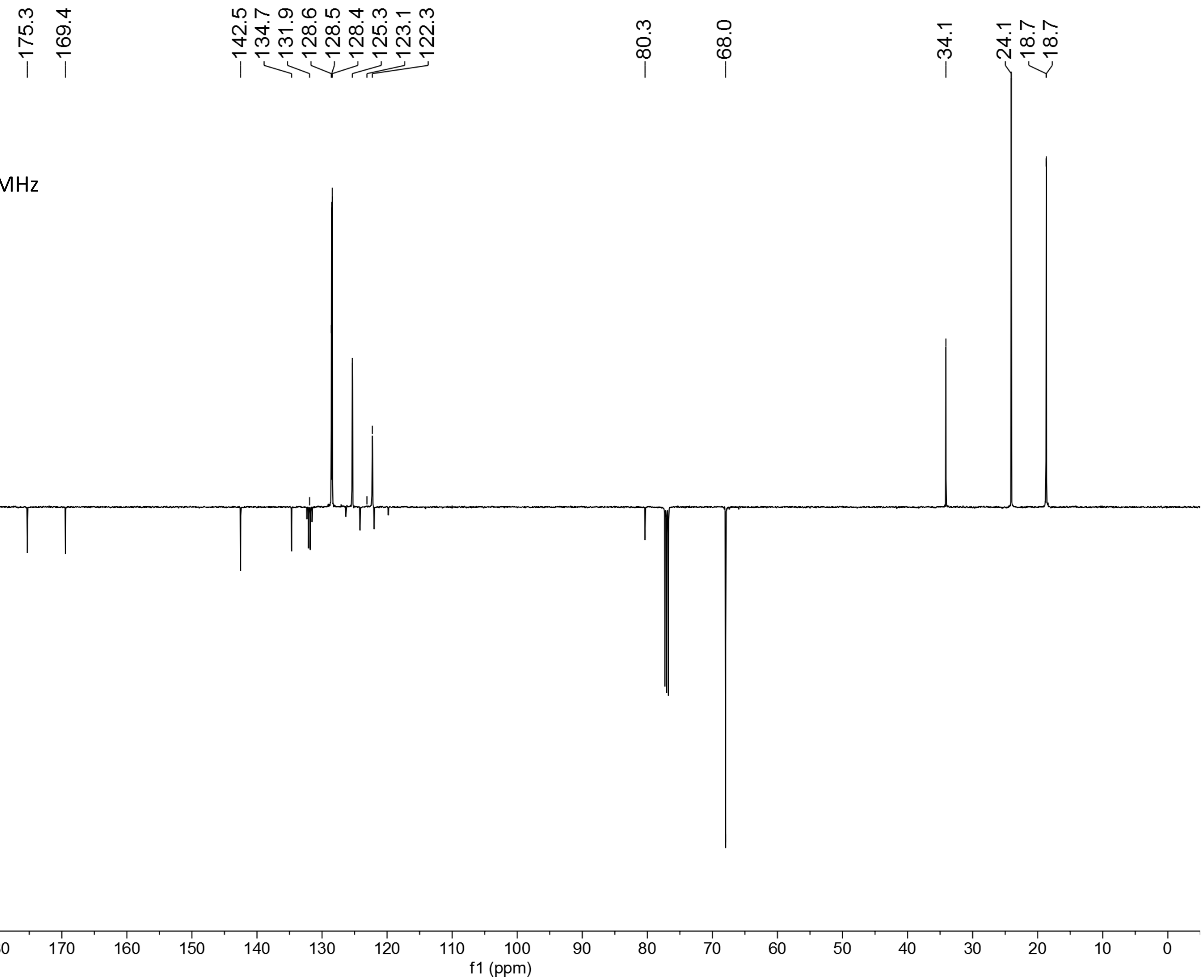

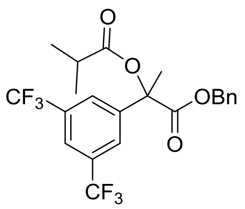

S37: [19]F, $CDCl_3$, 376 MHz

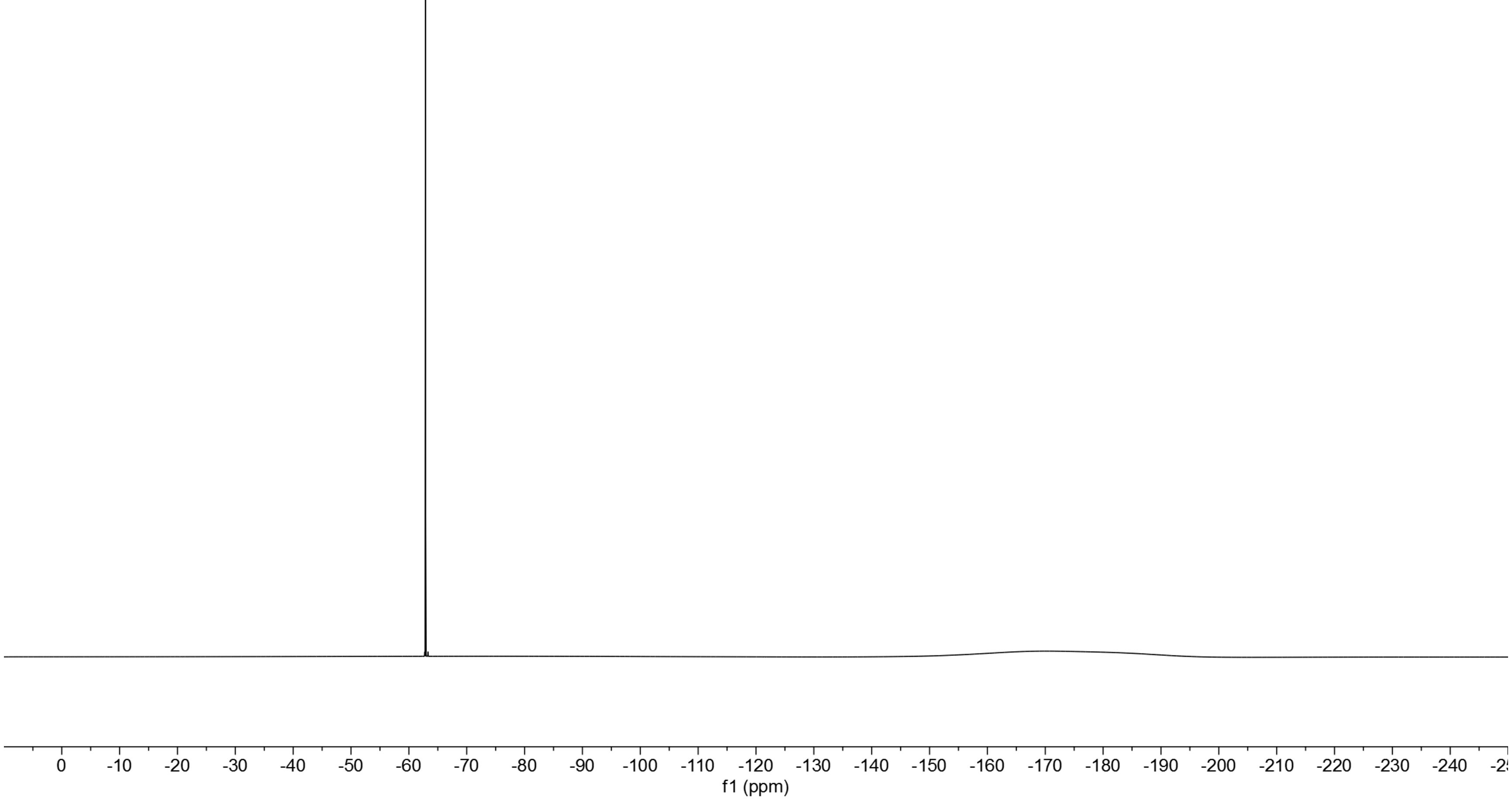

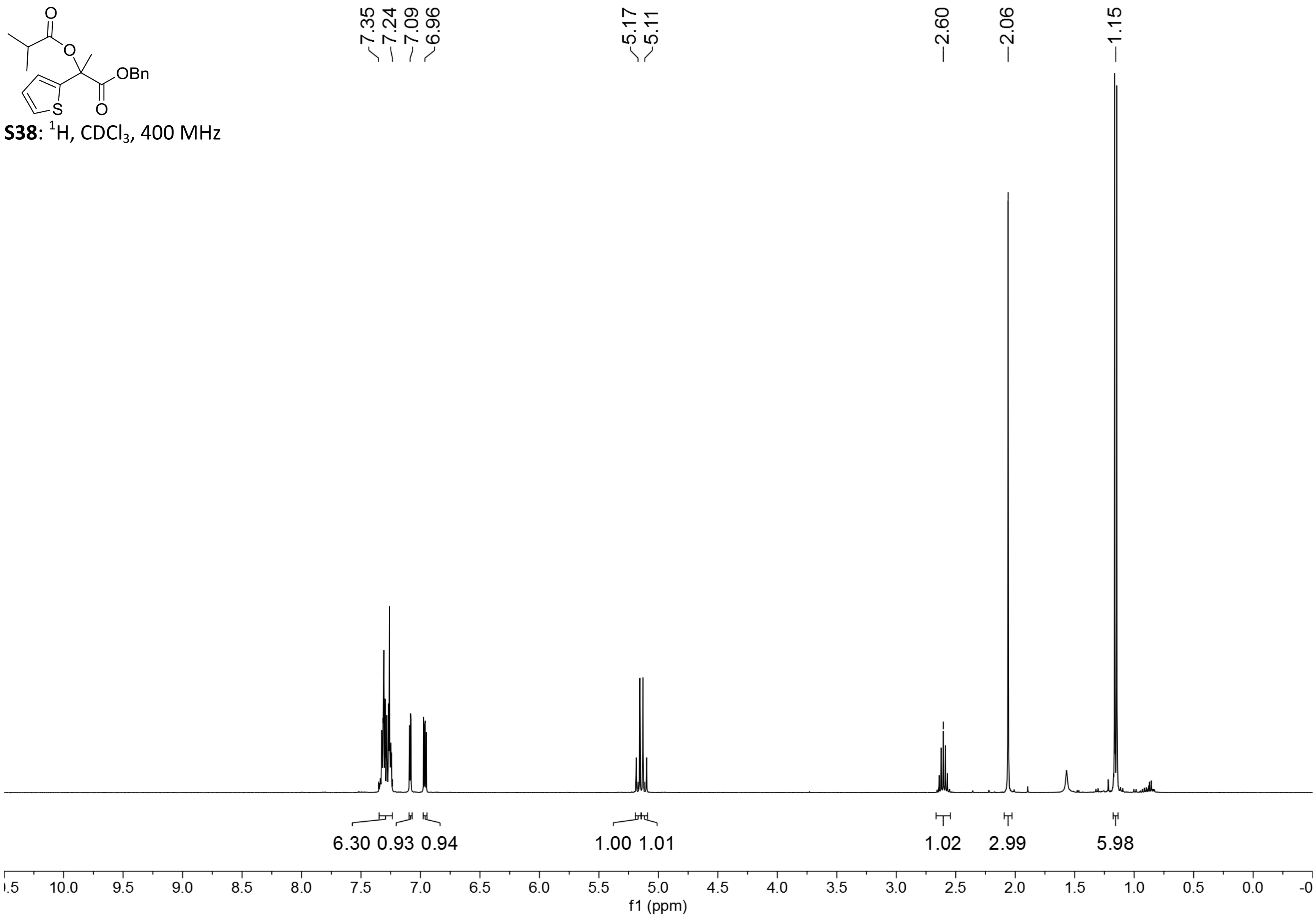

S38: ^{1}H, $CDCl_3$, 400 MHz

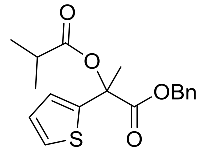

S38: ^{13}C, $CDCl_3$, 100 MHz

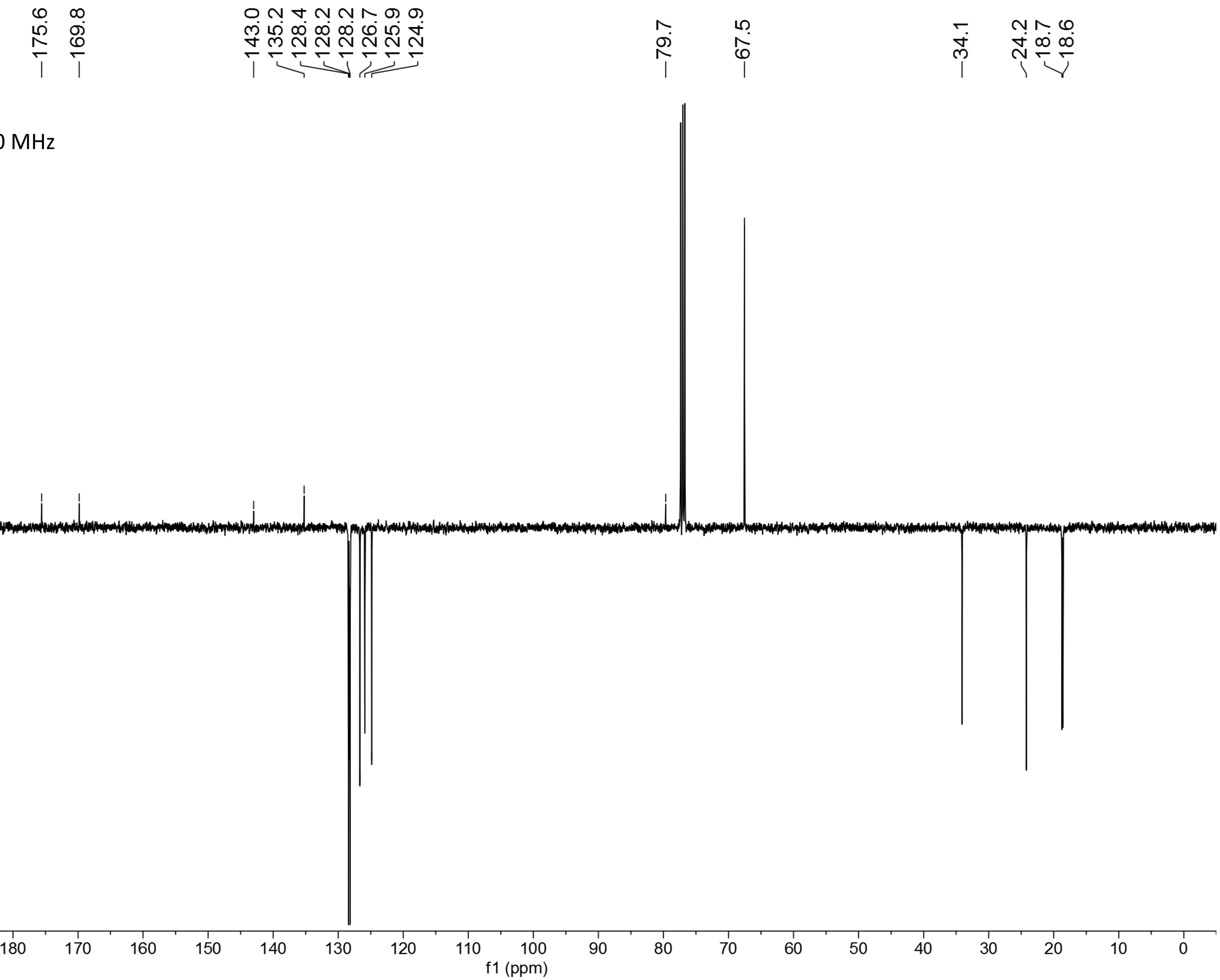

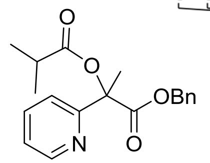

S39: ^{1}H, $CDCl_3$, 500 MHz

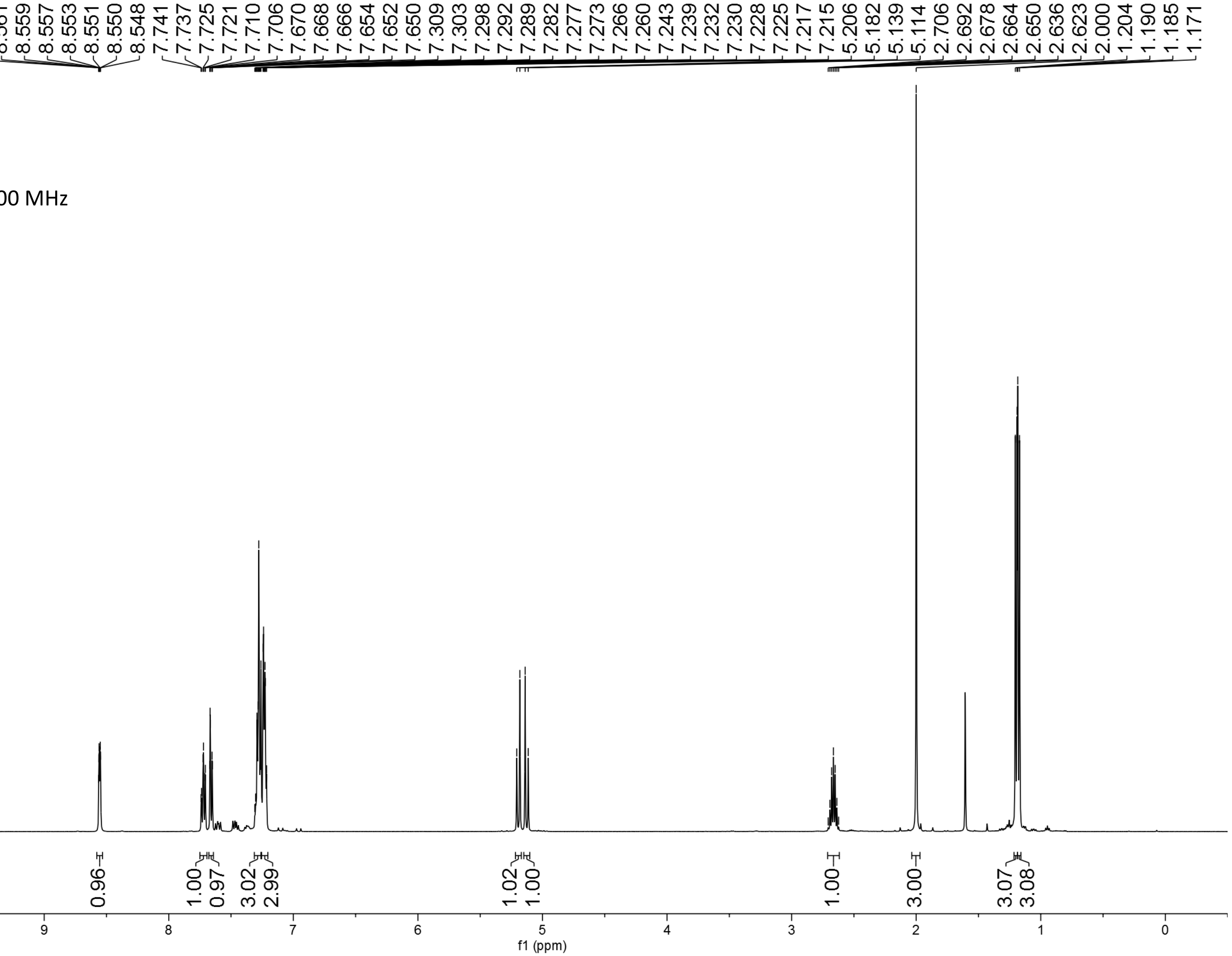

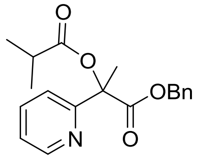

S39: ^{13}C, $CDCl_3$, 126 MHz

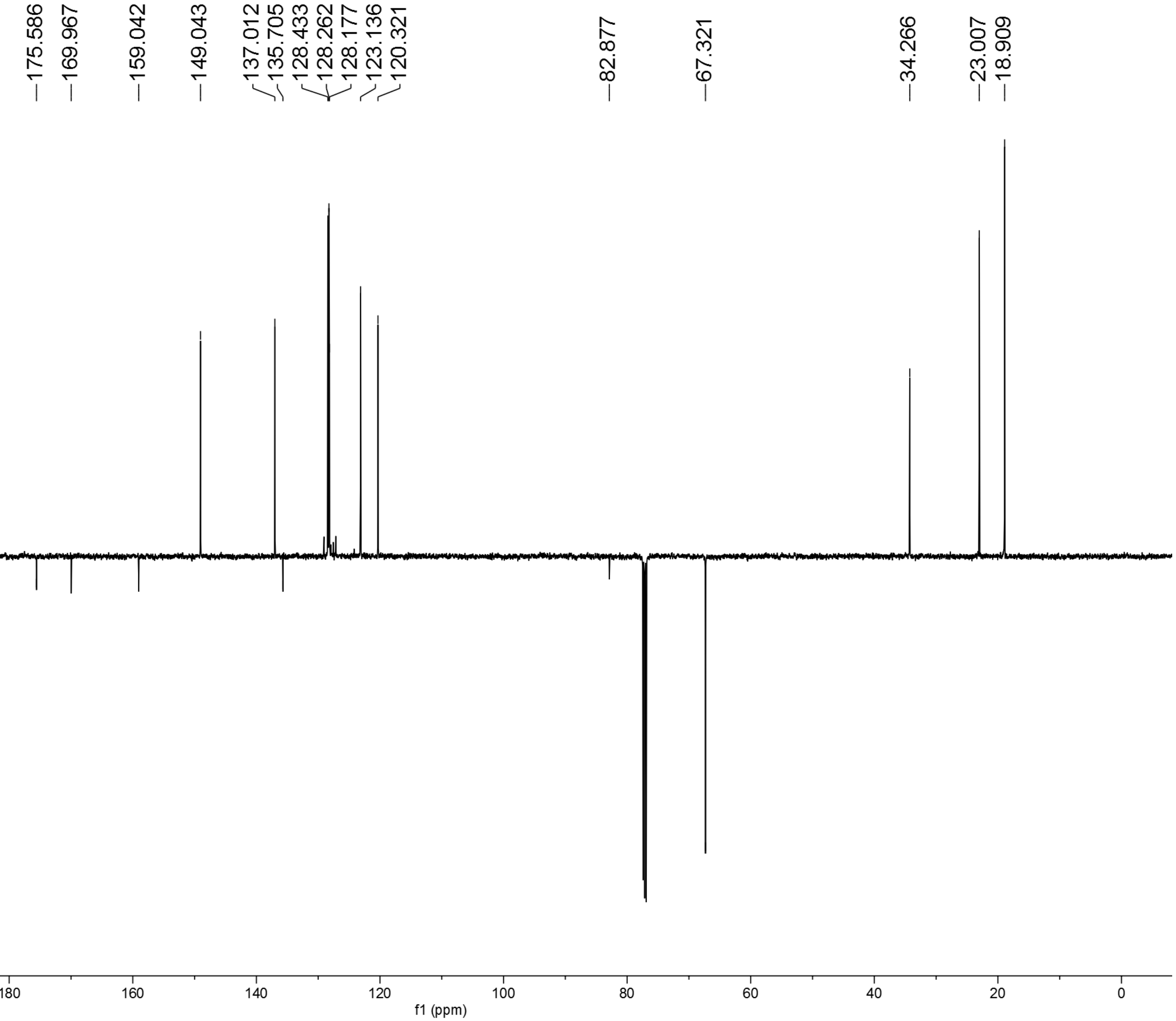

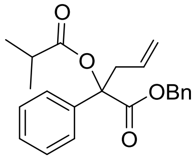

S40: ^{1}H, $CDCl_3$, 400 MHz

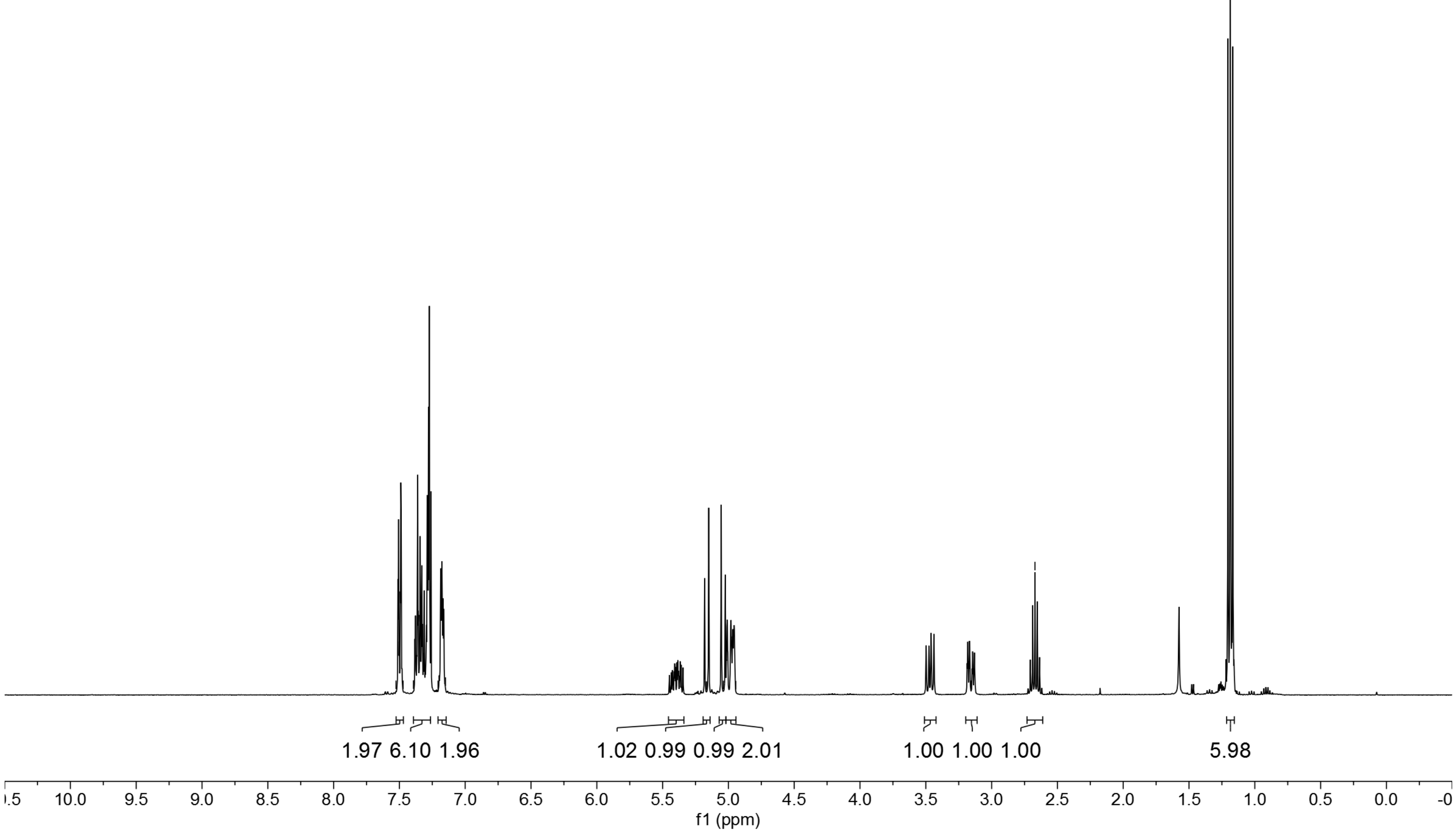

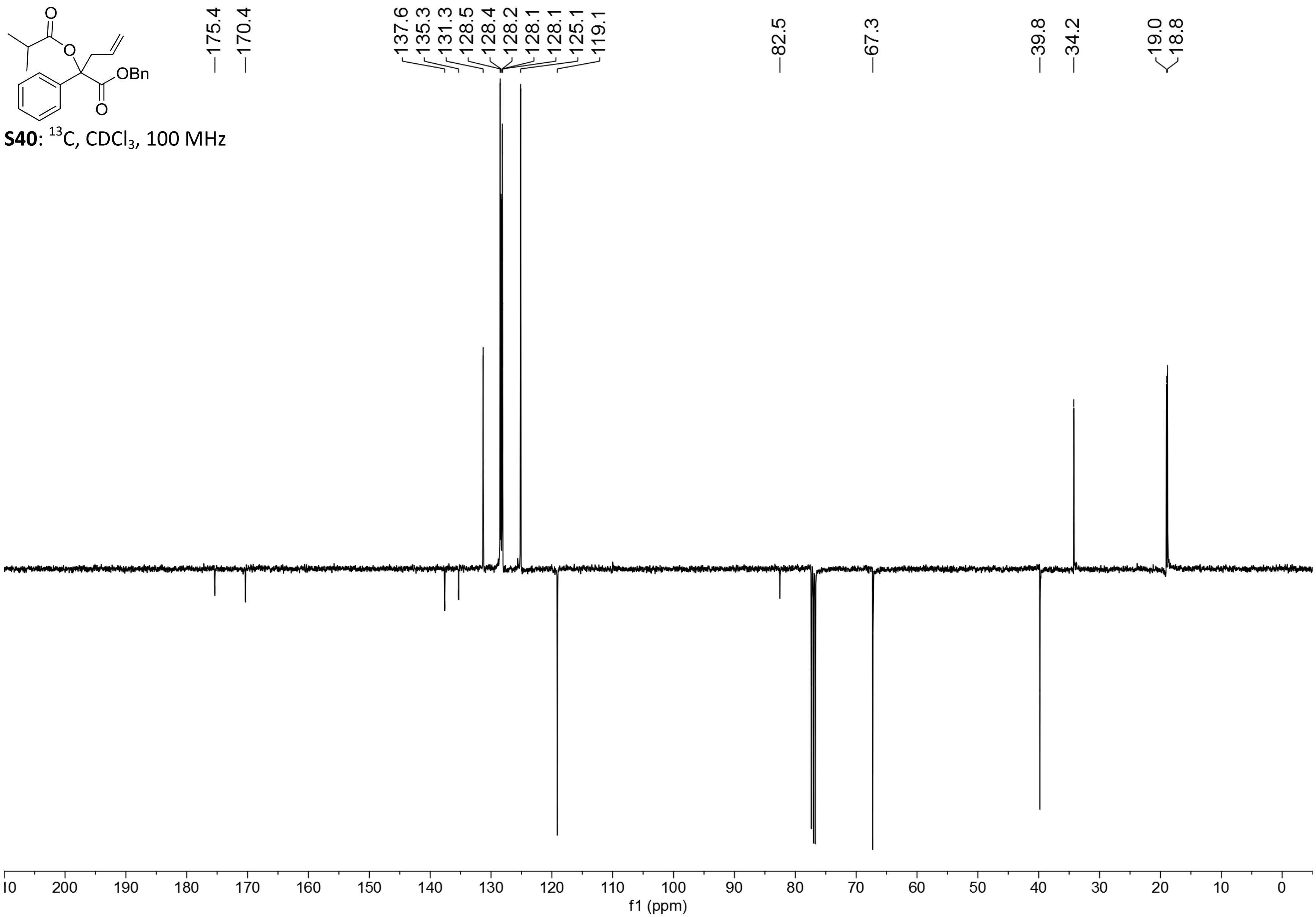

S40: ^{13}C, $CDCl_3$, 100 MHz

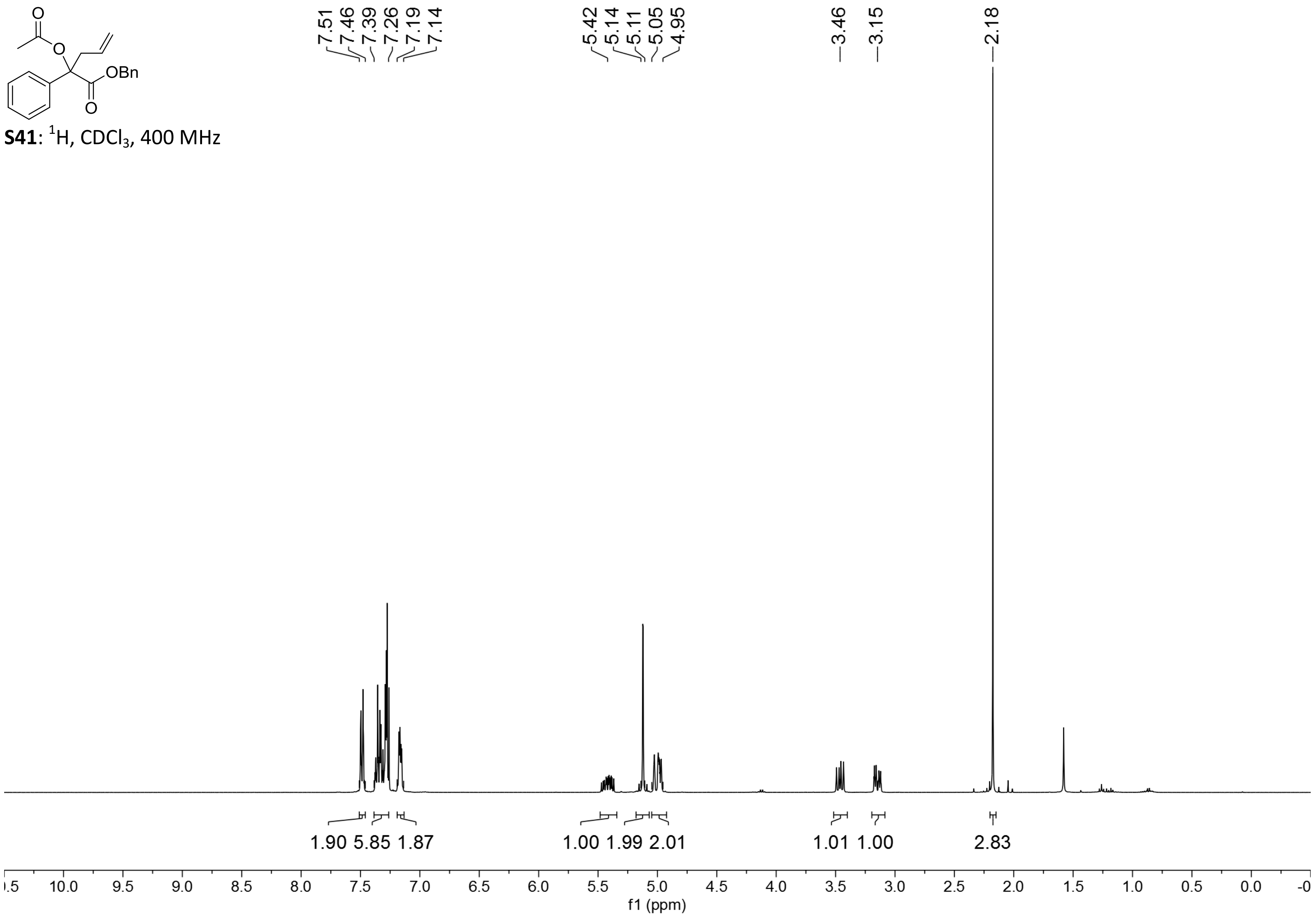
S41: ¹H, CDCl₃, 400 MHz
7.51
7.46
7.39
7.26
7.19
7.14
5.42
5.14
5.11
5.05
4.95
3.46
3.15
2.18
1.90 5.85 1.87
1.00 1.99 2.01
1.01 1.00
2.83
f1 (ppm)

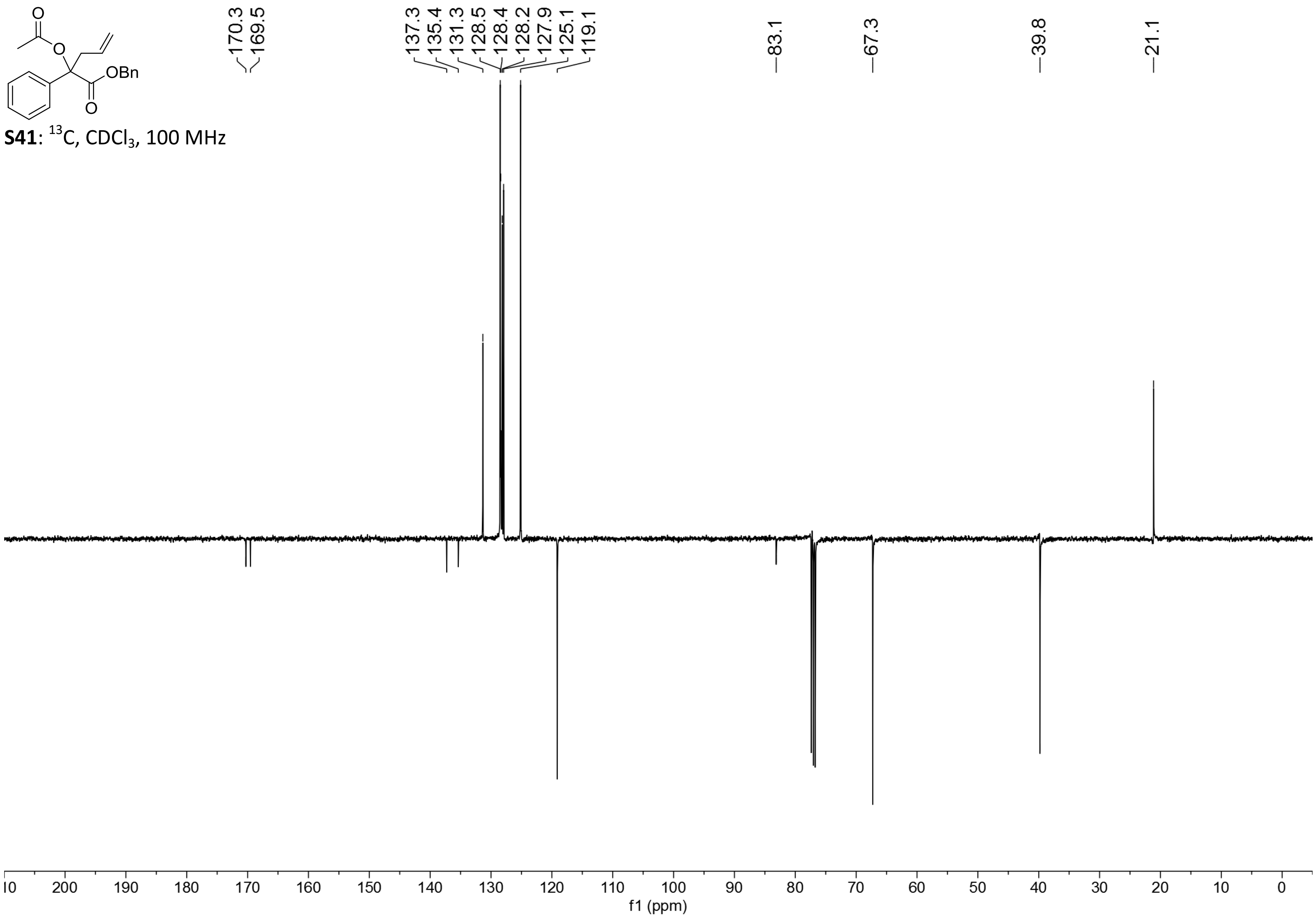

S41: ^{13}C, $CDCl_3$, 100 MHz

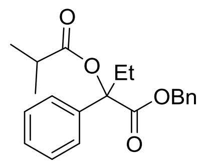

S42: [1]H, $CDCl_3$, 400 MHz

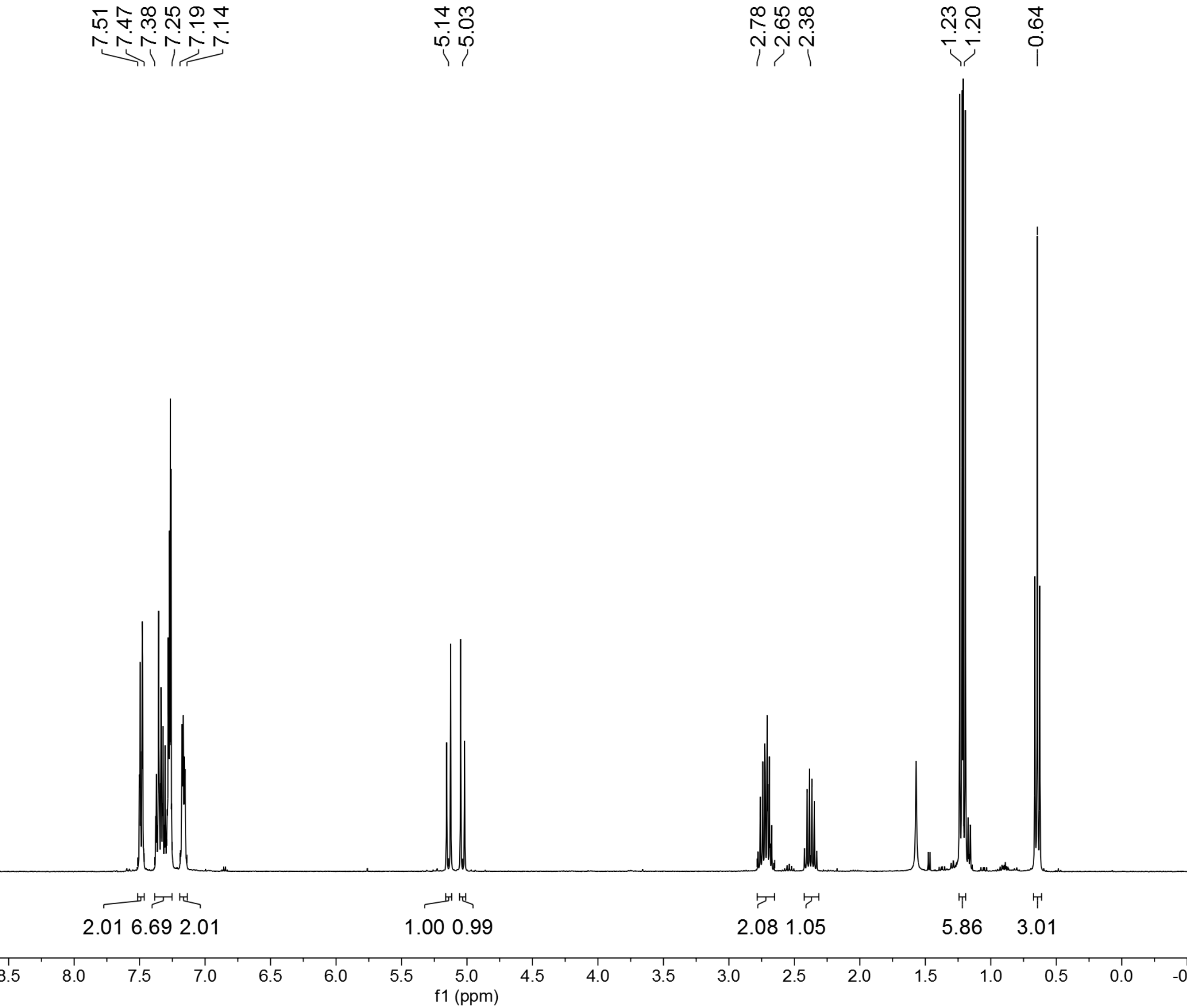

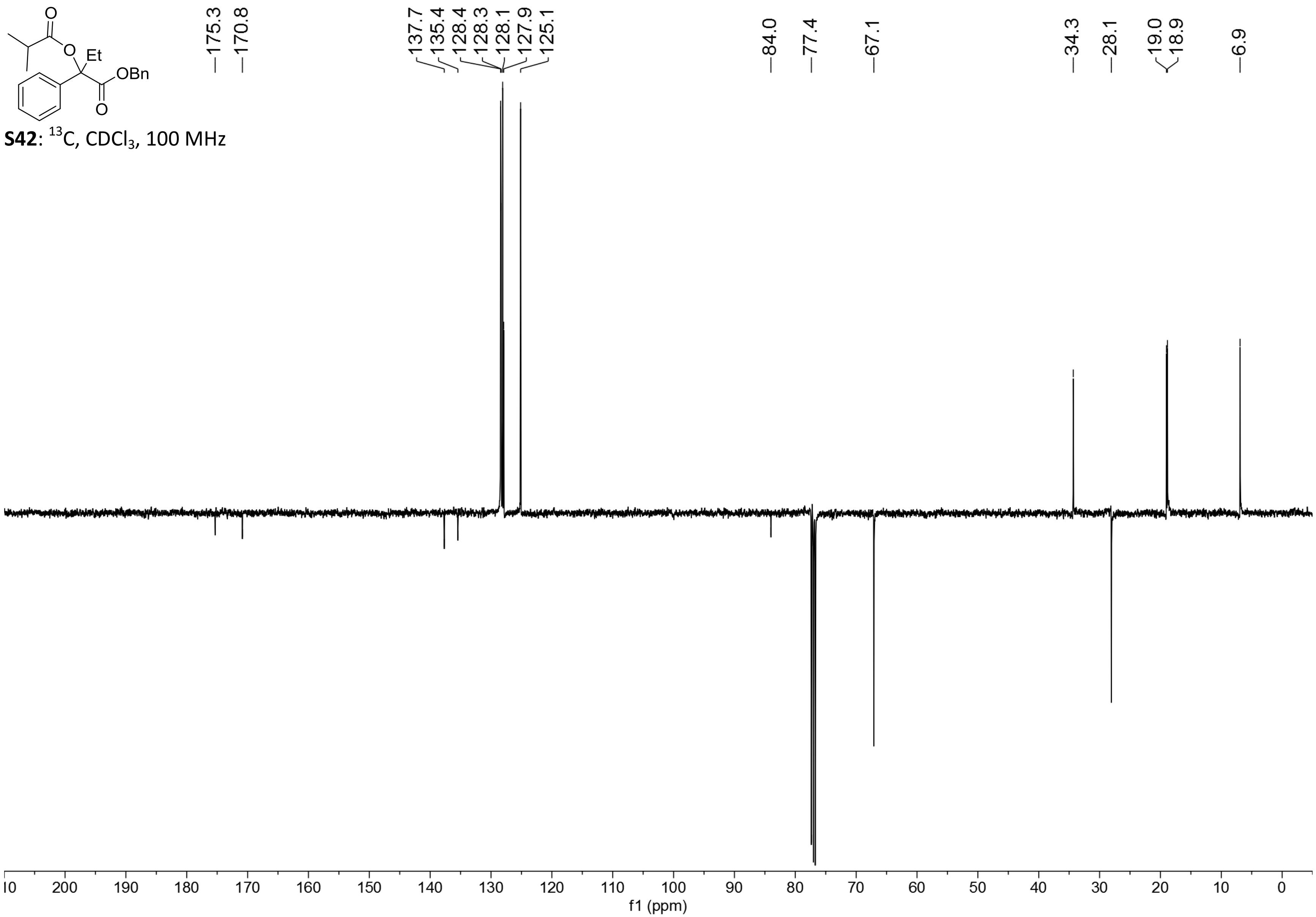

S42: ^{13}C, $CDCl_3$, 100 MHz

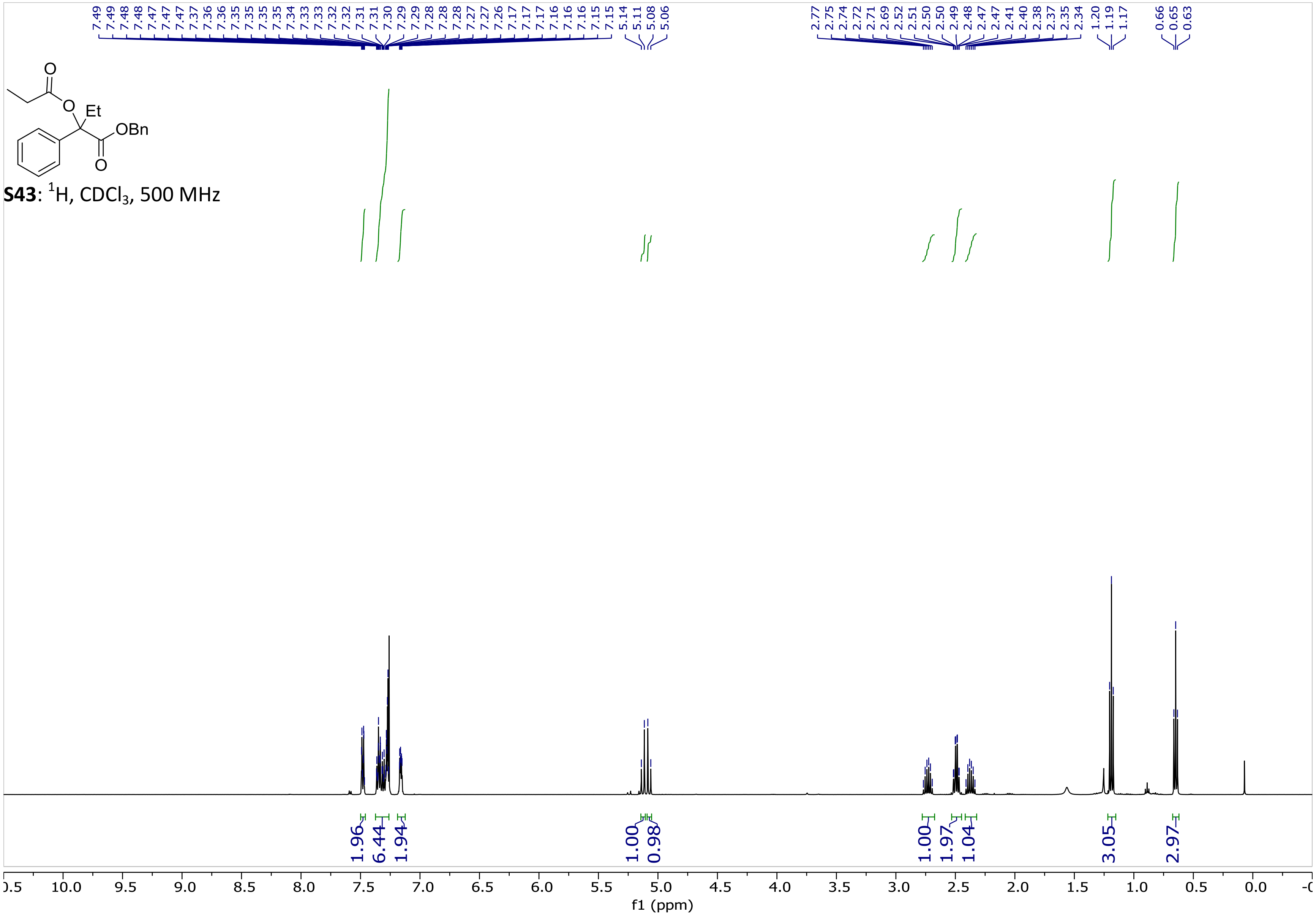

S43: 1H, CDCl3, 500 MHz
OBn
Et
f1 (ppm)

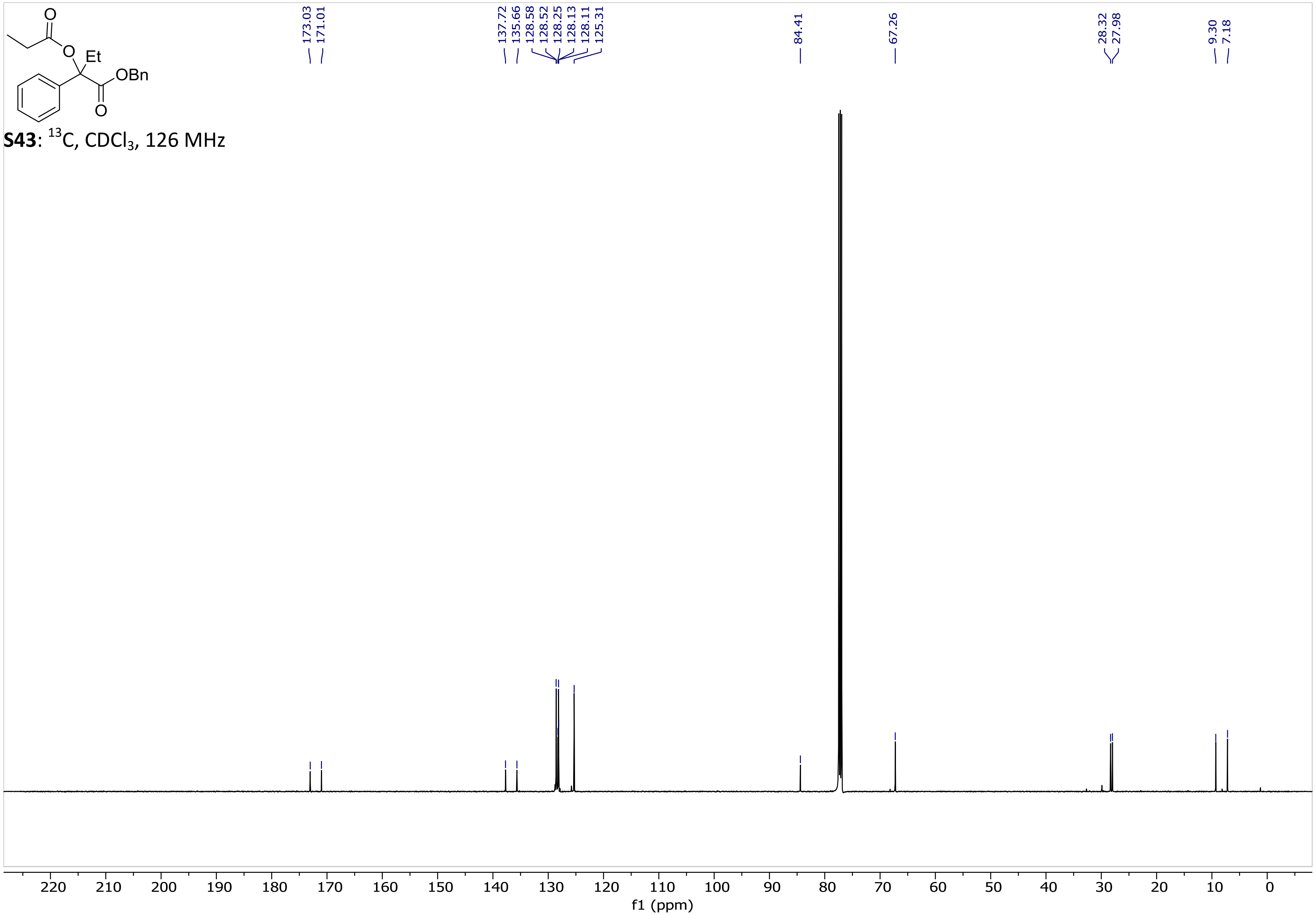
S43: ^{13}C, $CDCl_3$, 126 MHz
Et
OBn
O
173.03
171.01
137.72
135.66
128.58
128.52
128.25
128.13
128.11
125.31
84.41
67.26
28.32
27.98
9.30
7.18
220 210 200 190 180 170 160 150 140 130 120 110 100 90 80 70 60 50 40 30 20 10 0
f1 (ppm)

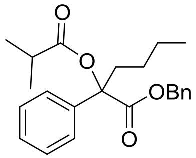

S44: ^{1}H, $CDCl_3$, 400 MHz

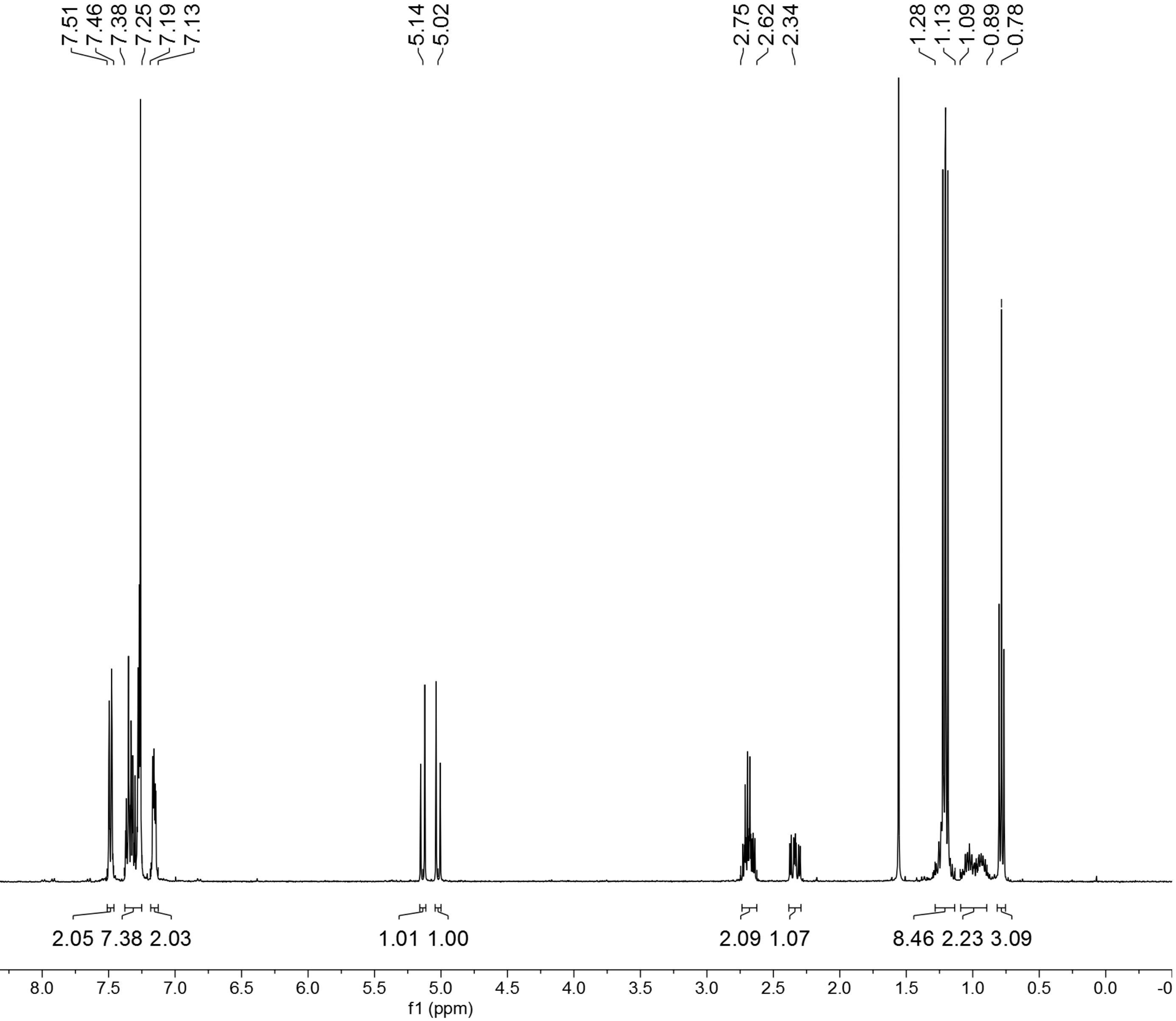

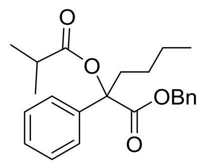

S44: ^{13}C, $CDCl_3$, 100 MHz

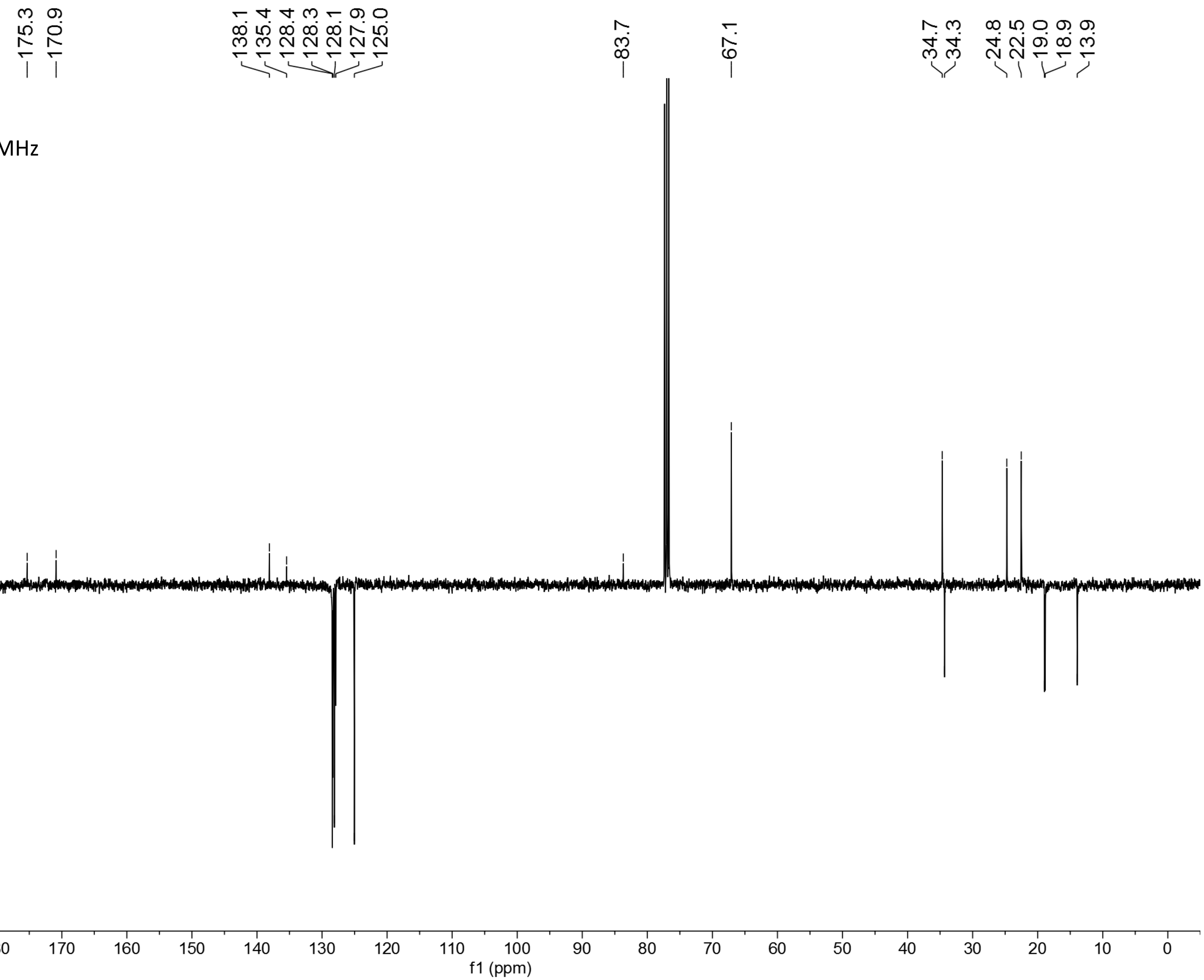

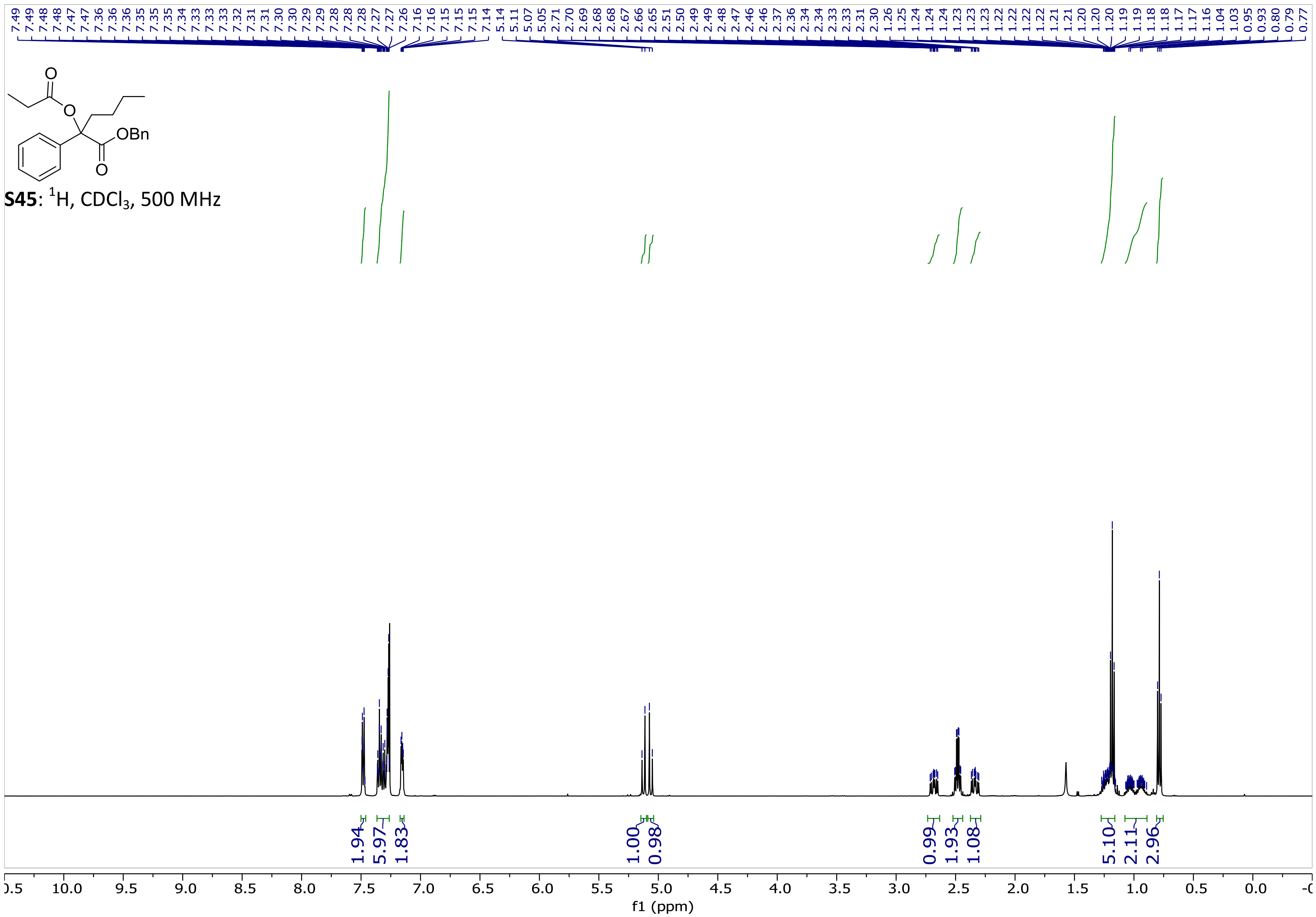
OBn
S45: ¹H, CDCl₃, 500 MHz
1.94
5.97
1.83
1.00
0.98
0.99
1.93
1.08
5.10
2.11
2.96
f1 (ppm)

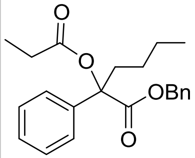

S45: ^{13}C, $CDCl_3$, 126 MHz

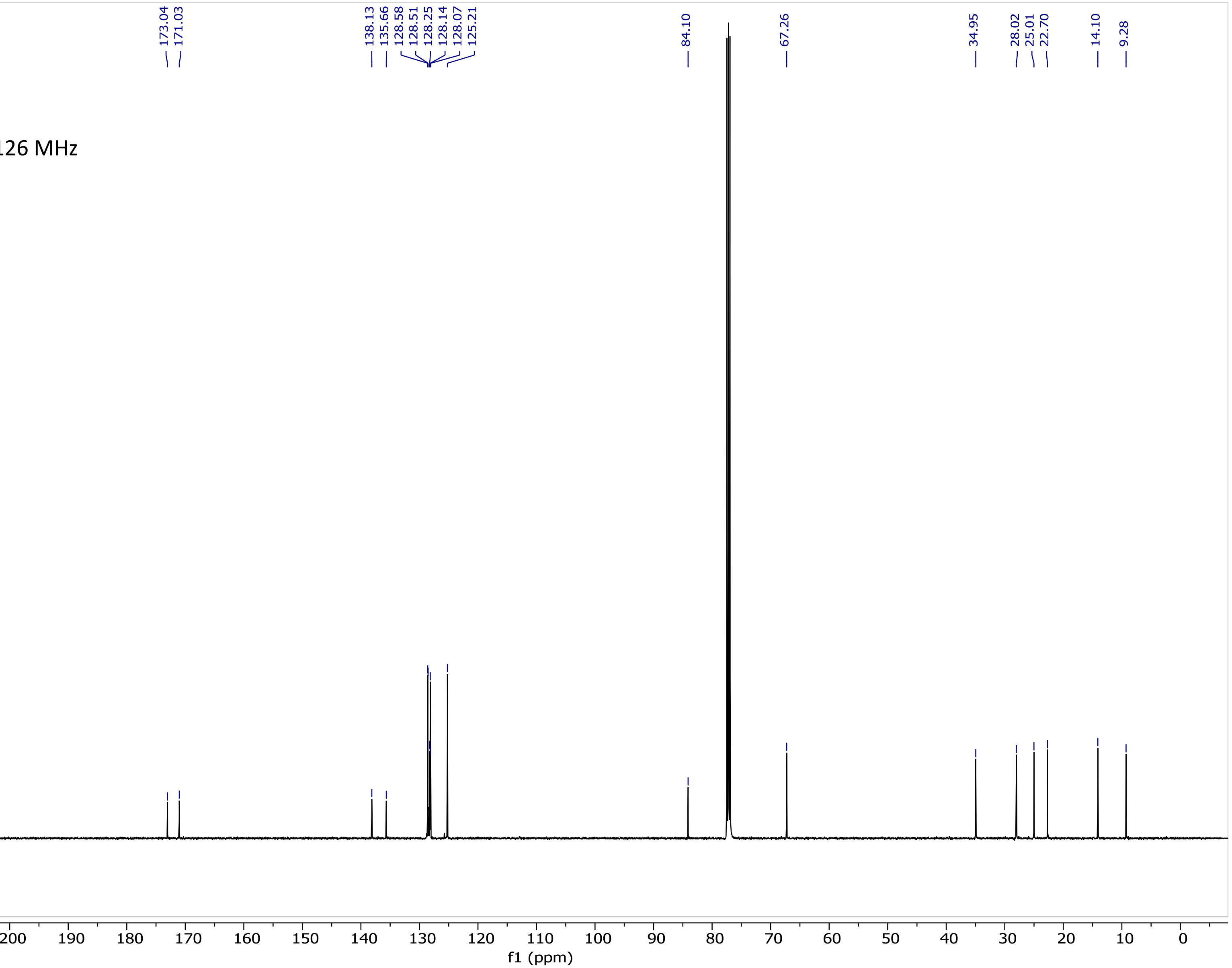

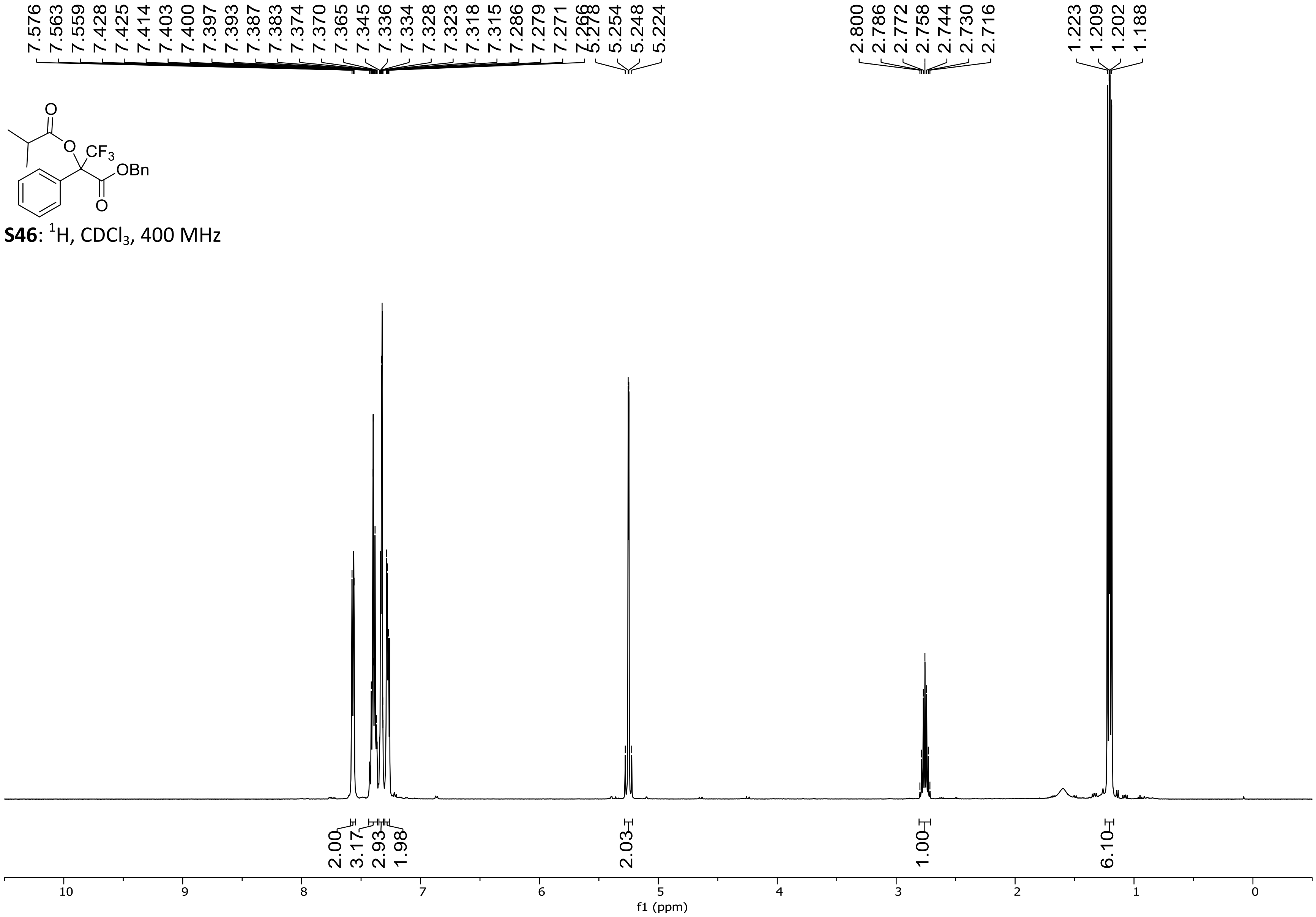

S46: ^{1}H, $CDCl_3$, 400 MHz

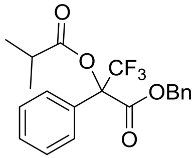

S46: ^{13}C, $CDCl_3$, 100 MHz

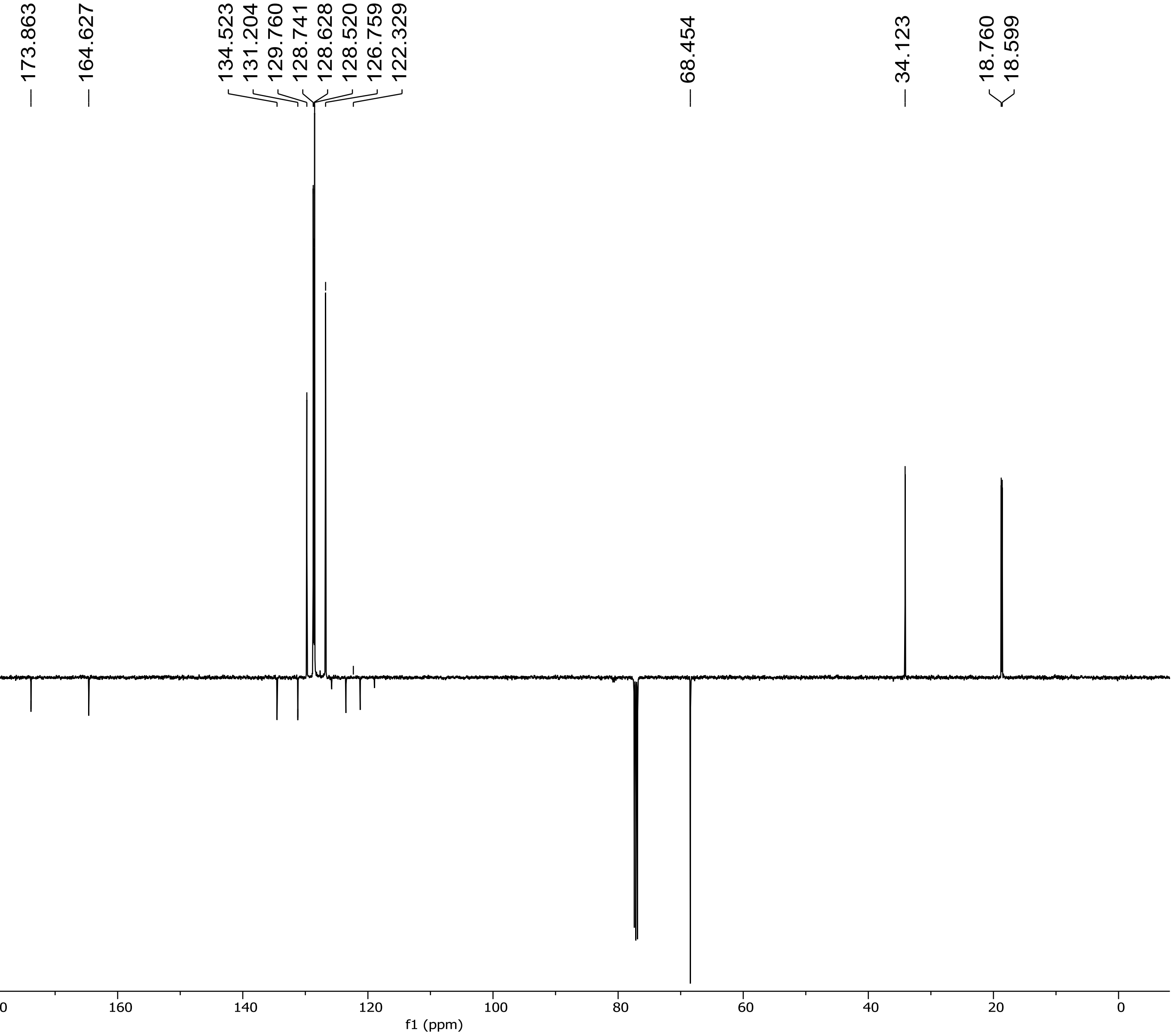

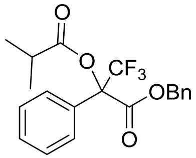

S46: ^{19}F, $CDCl_3$, 376 MHz

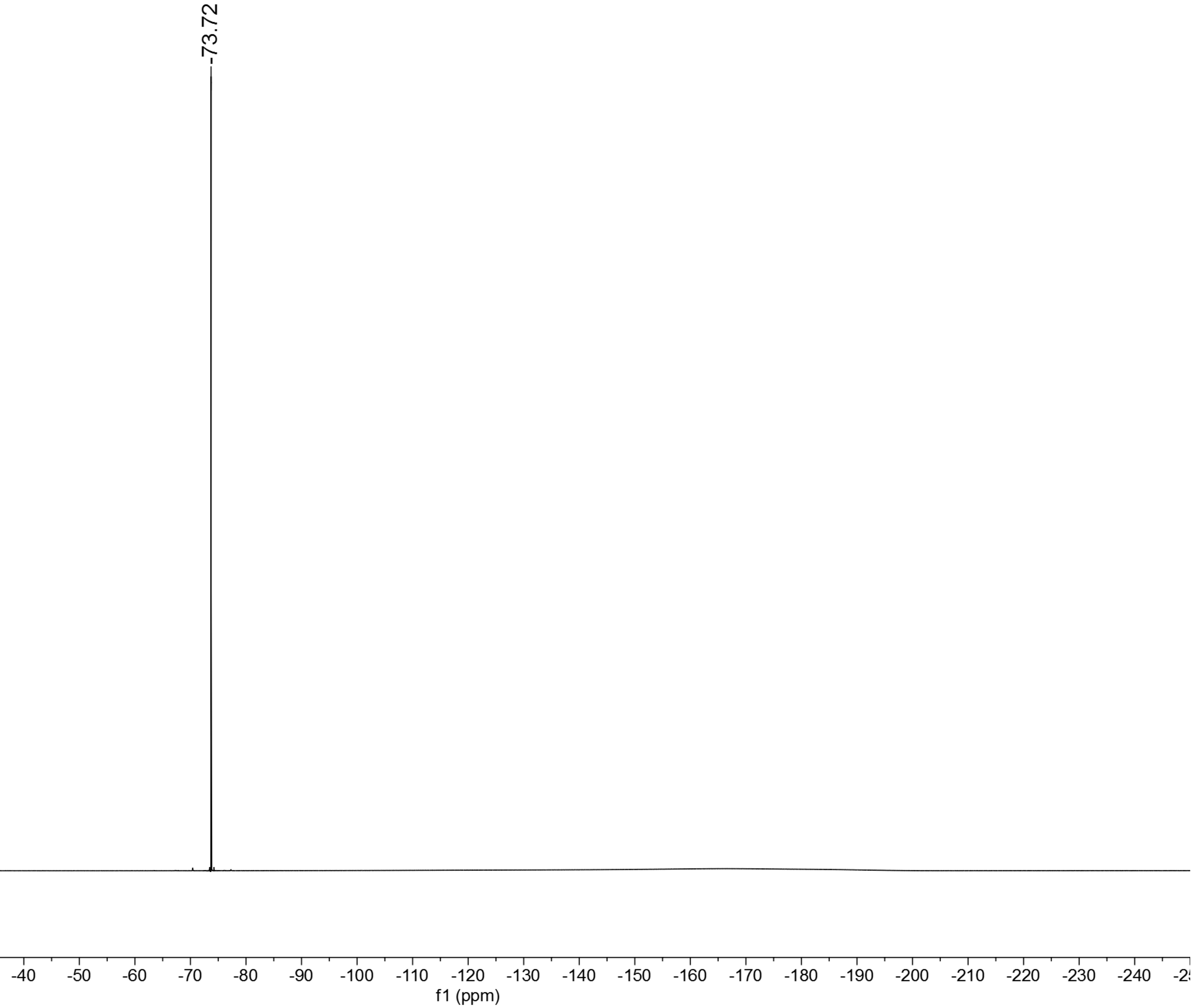

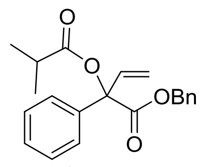

S47: ^{1}H, $CDCl_3$, 400 MHz

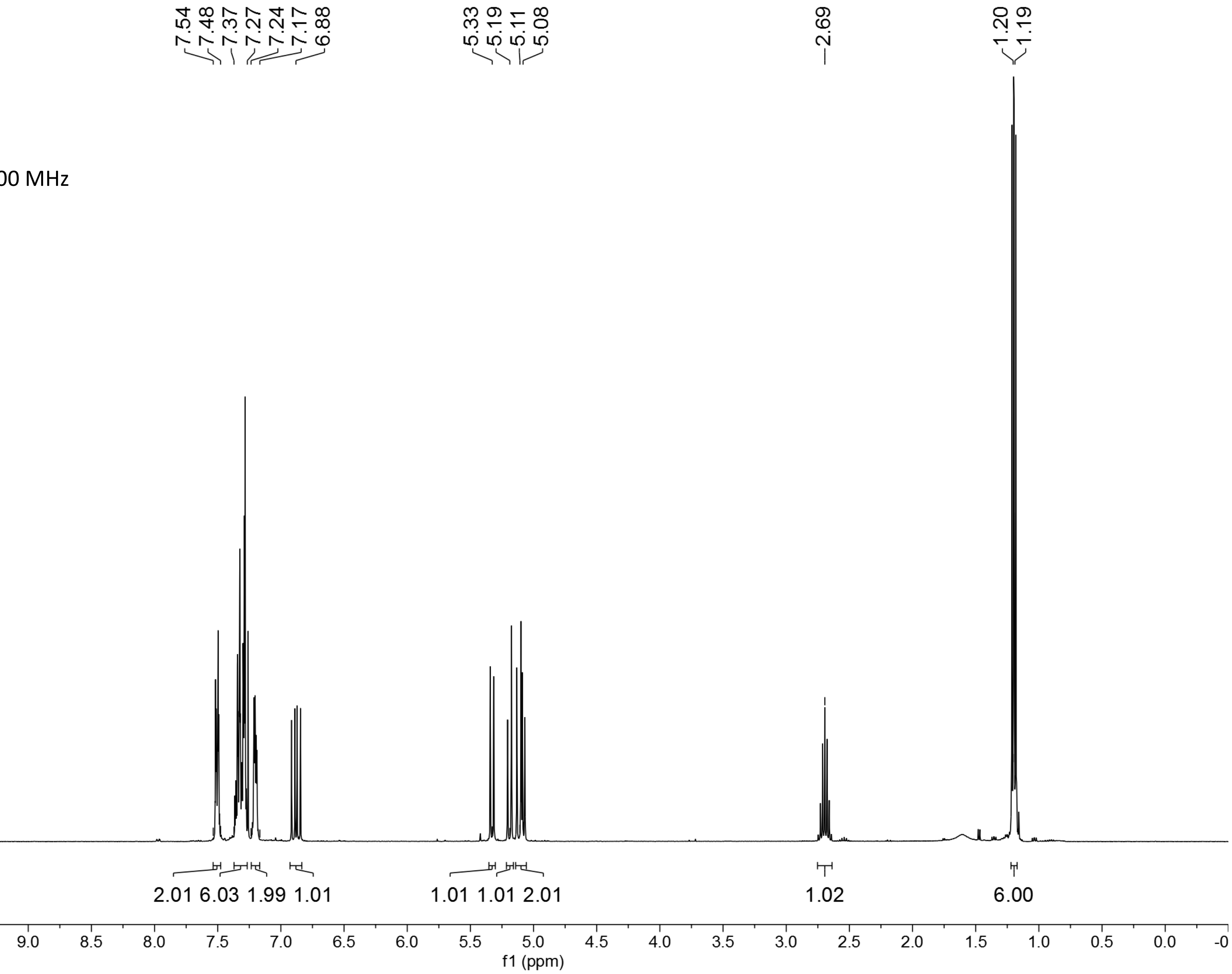

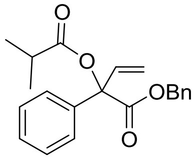

S47: ^{13}C, $CDCl_3$, 100 MHz

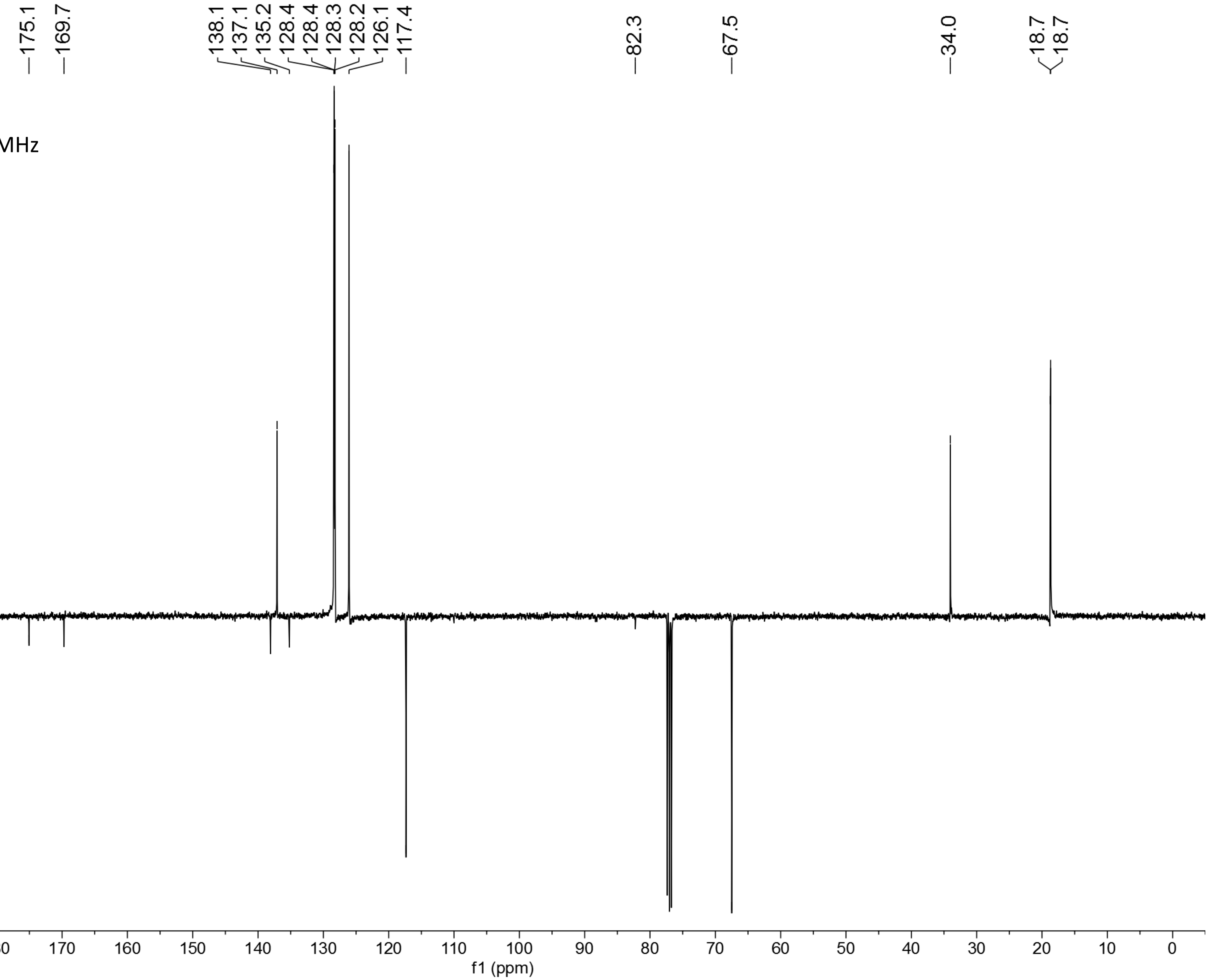

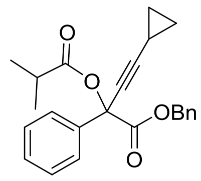

S48: ^{1}H, $CDCl_3$, 400 MHz

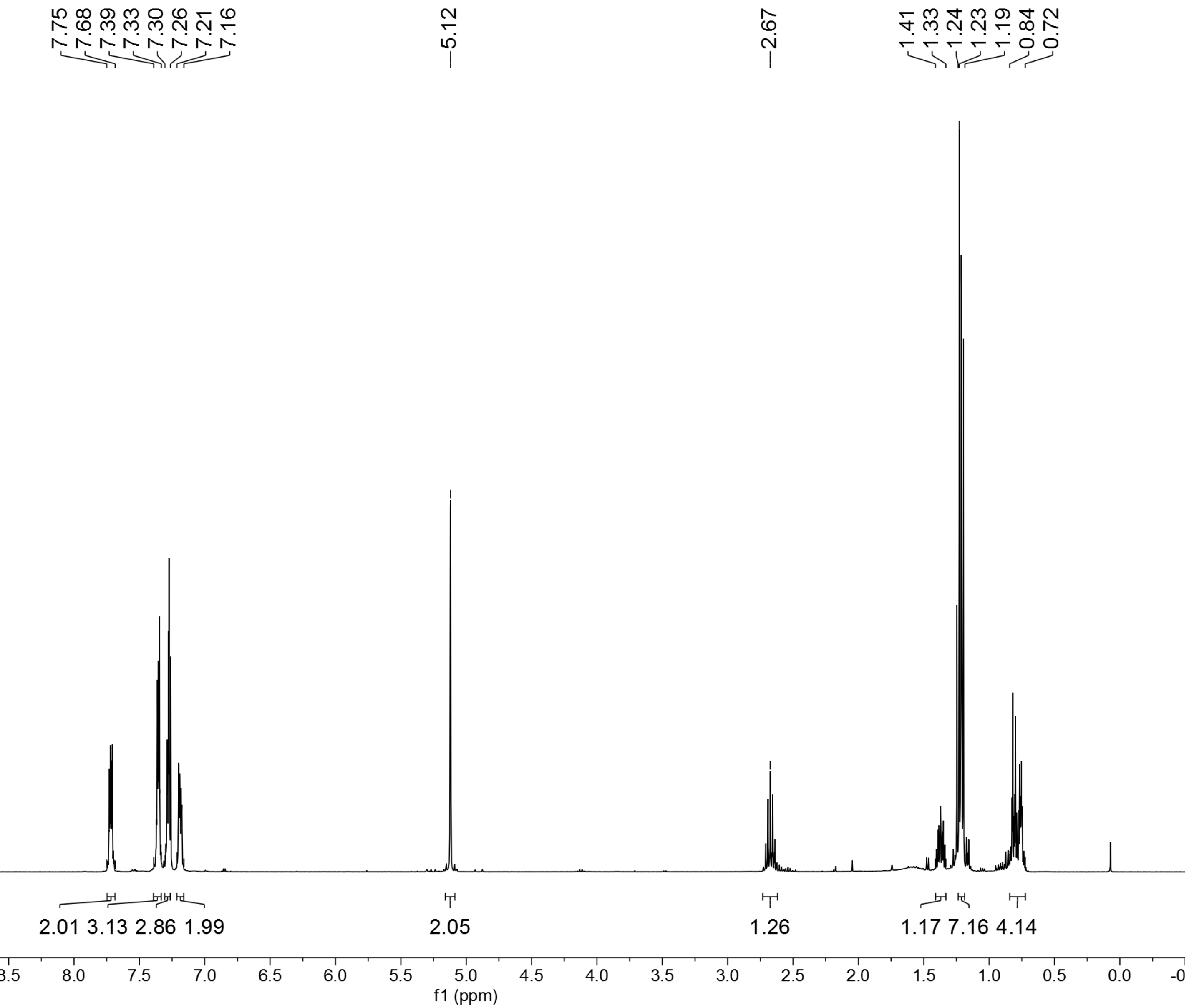

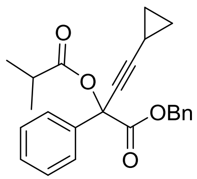

S48: ^{13}C, $CDCl_3$, 100 MHz

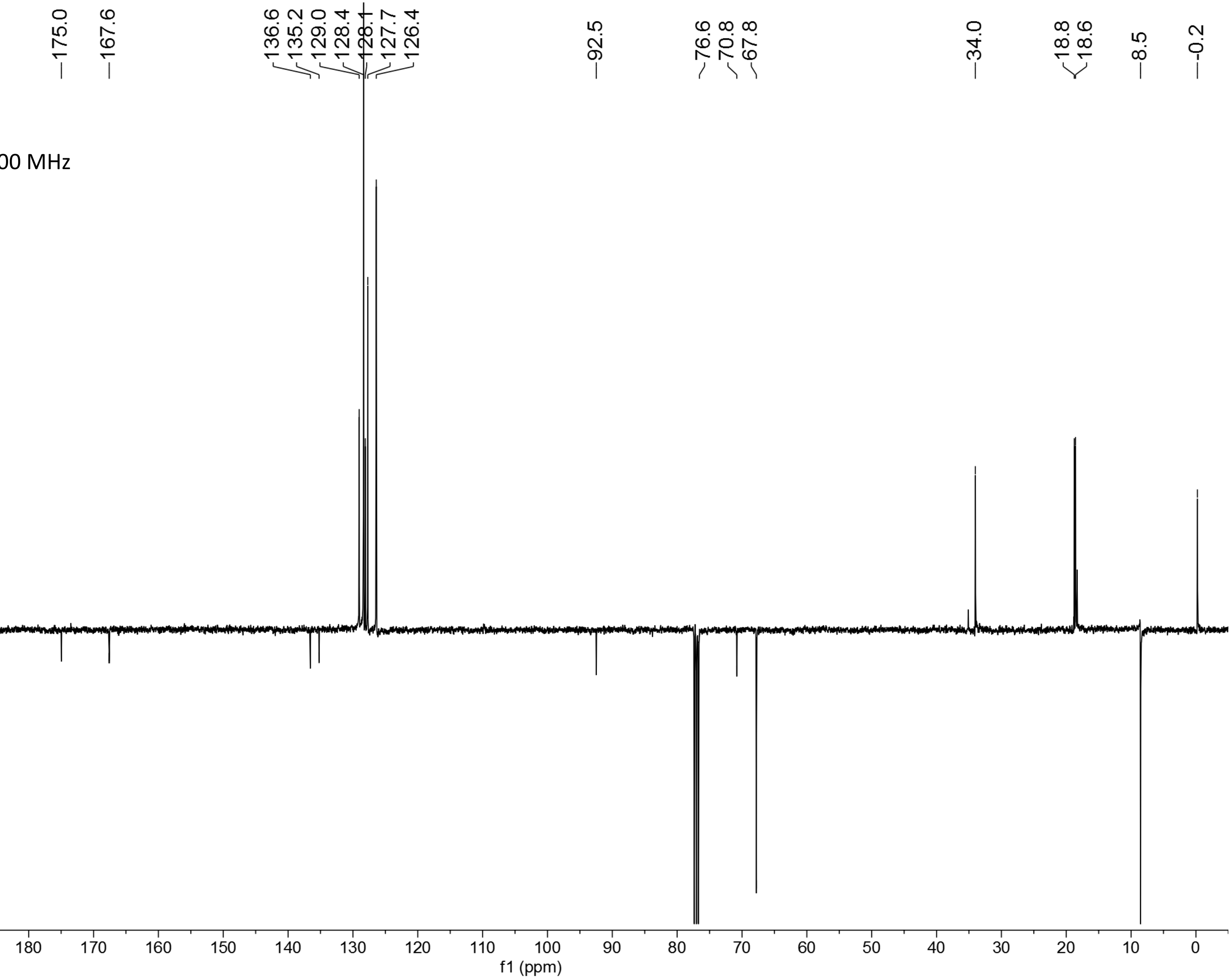

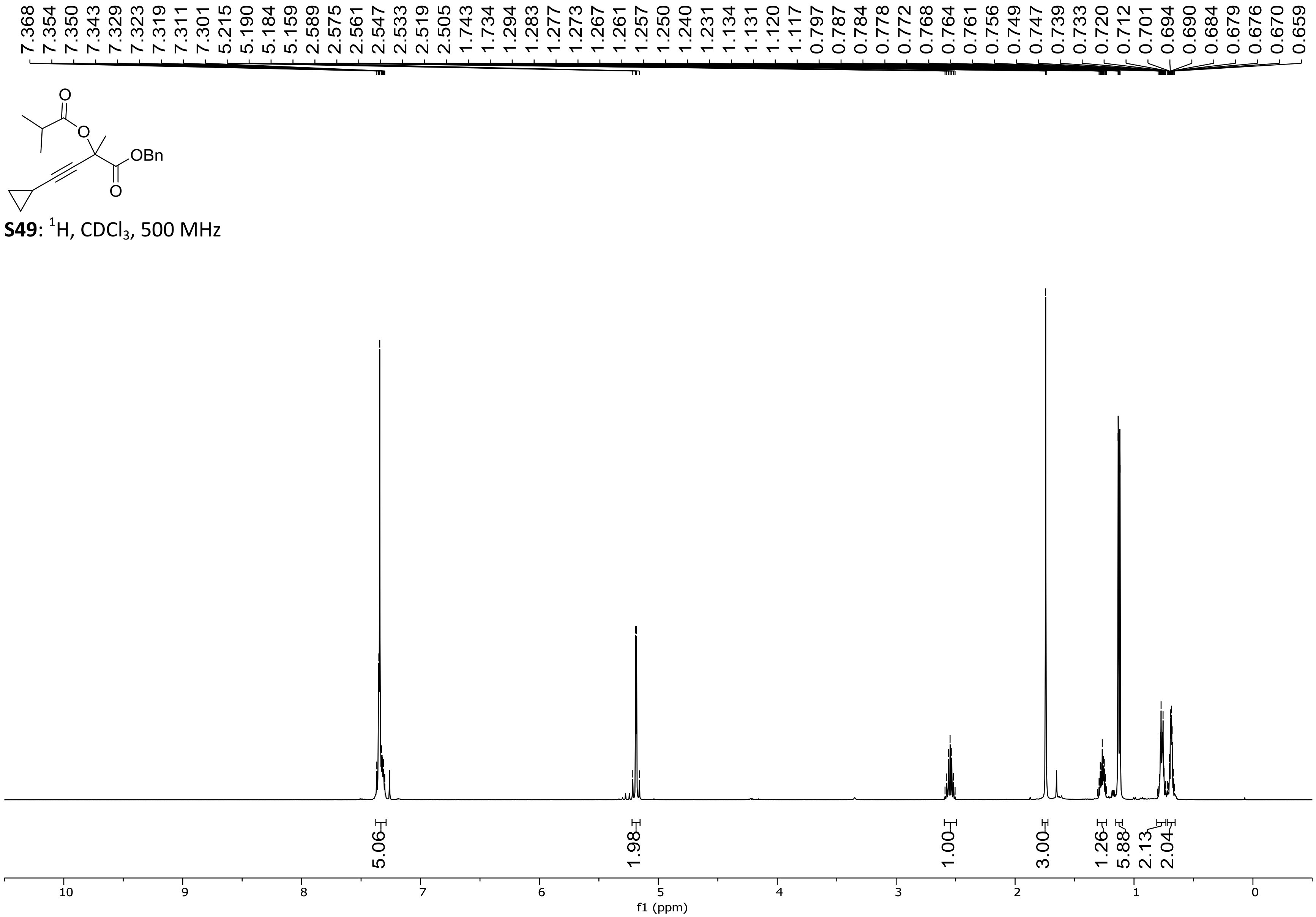

7.368
7.354
7.350
7.343
7.329
7.323
7.319
7.311
7.301
5.215
5.190
5.184
5.159
2.589
2.575
2.561
2.547
2.533
2.519
2.505
1.743
1.734
1.294
1.283
1.277
1.273
1.267
1.261
1.257
1.250
1.240
1.231
1.134
1.131
1.120
1.117
0.797
0.787
0.784
0.778
0.772
0.768
0.764
0.761
0.756
0.749
0.747
0.739
0.733
0.720
0.712
0.701
0.694
0.690
0.684
0.679
0.676
0.670
0.659
O
O
OBn
O
S49: ^{1}H, $CDCl_3$, 500 MHz
5.06
1.98
1.00
3.00
1.26
5.88
2.13
2.04
10
9
8
7
6
5
4
3
2
1
0
f1 (ppm)

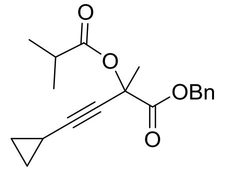

S49: ^{13}C, $CDCl_3$, 126 MHz

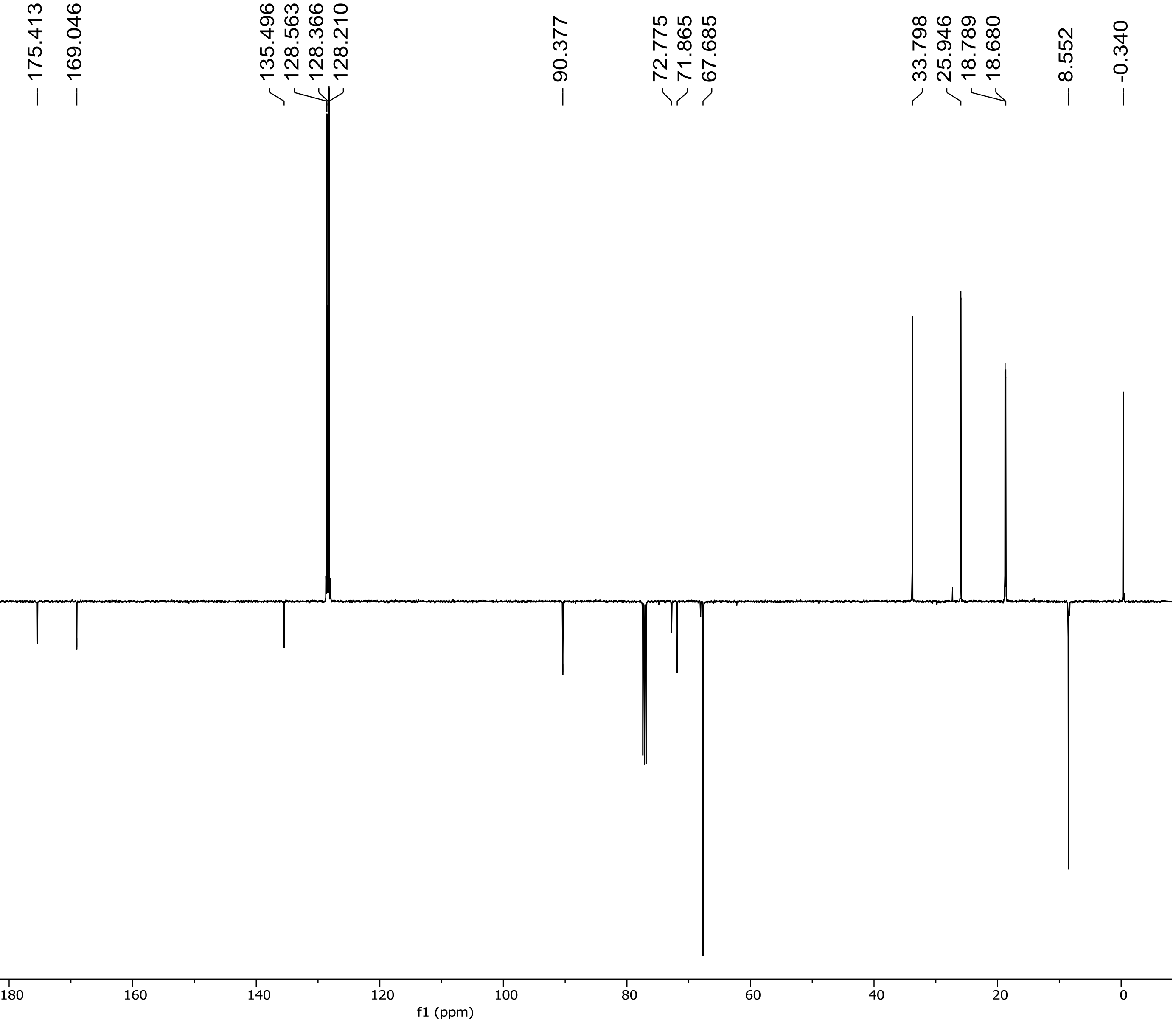

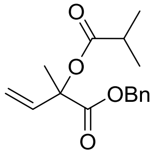

S50: ^{1}H, $CDCl_3$, 500 MHz

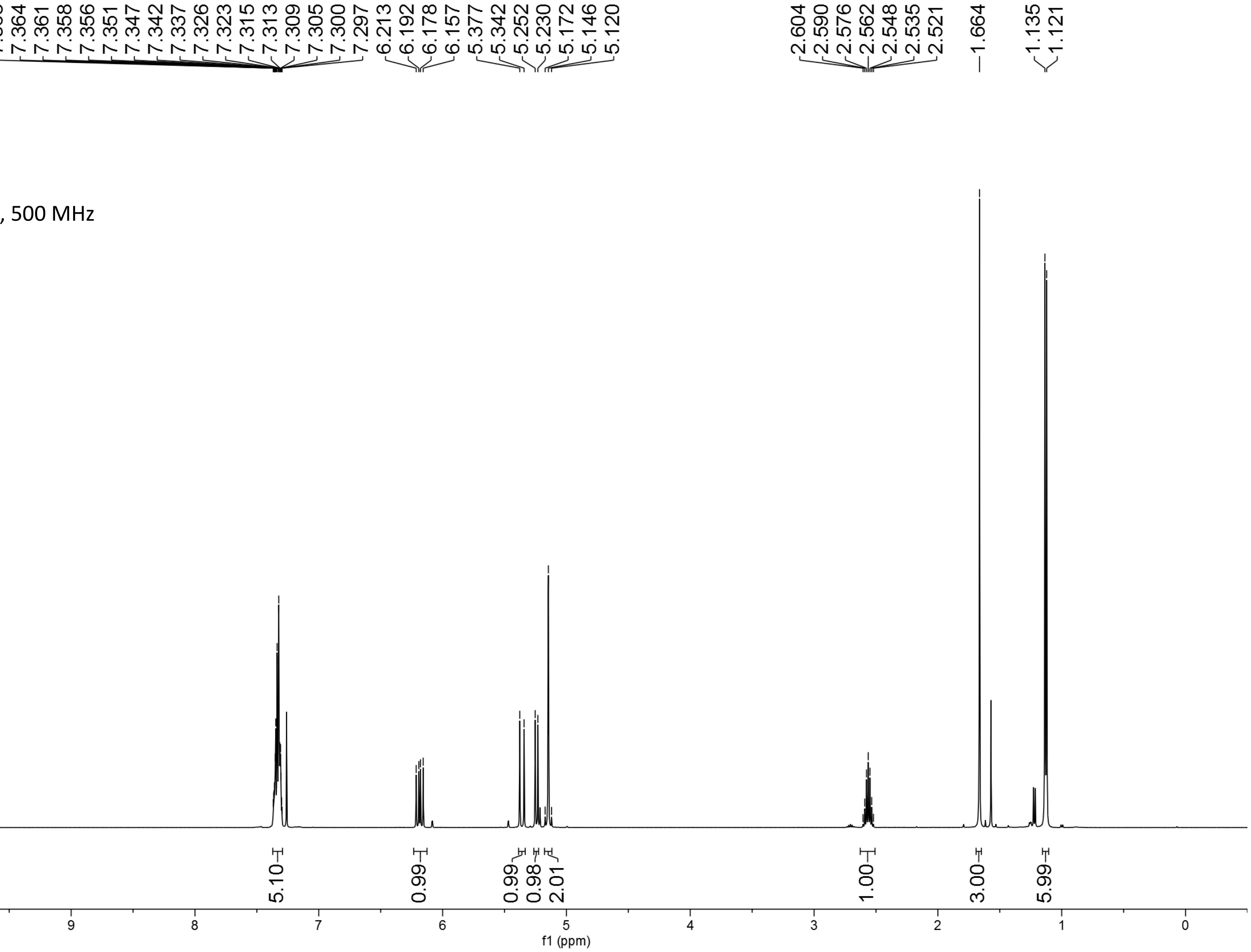

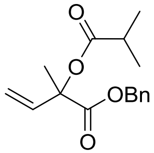

S50: ^{13}C, $CDCl_3$, 126 MHz

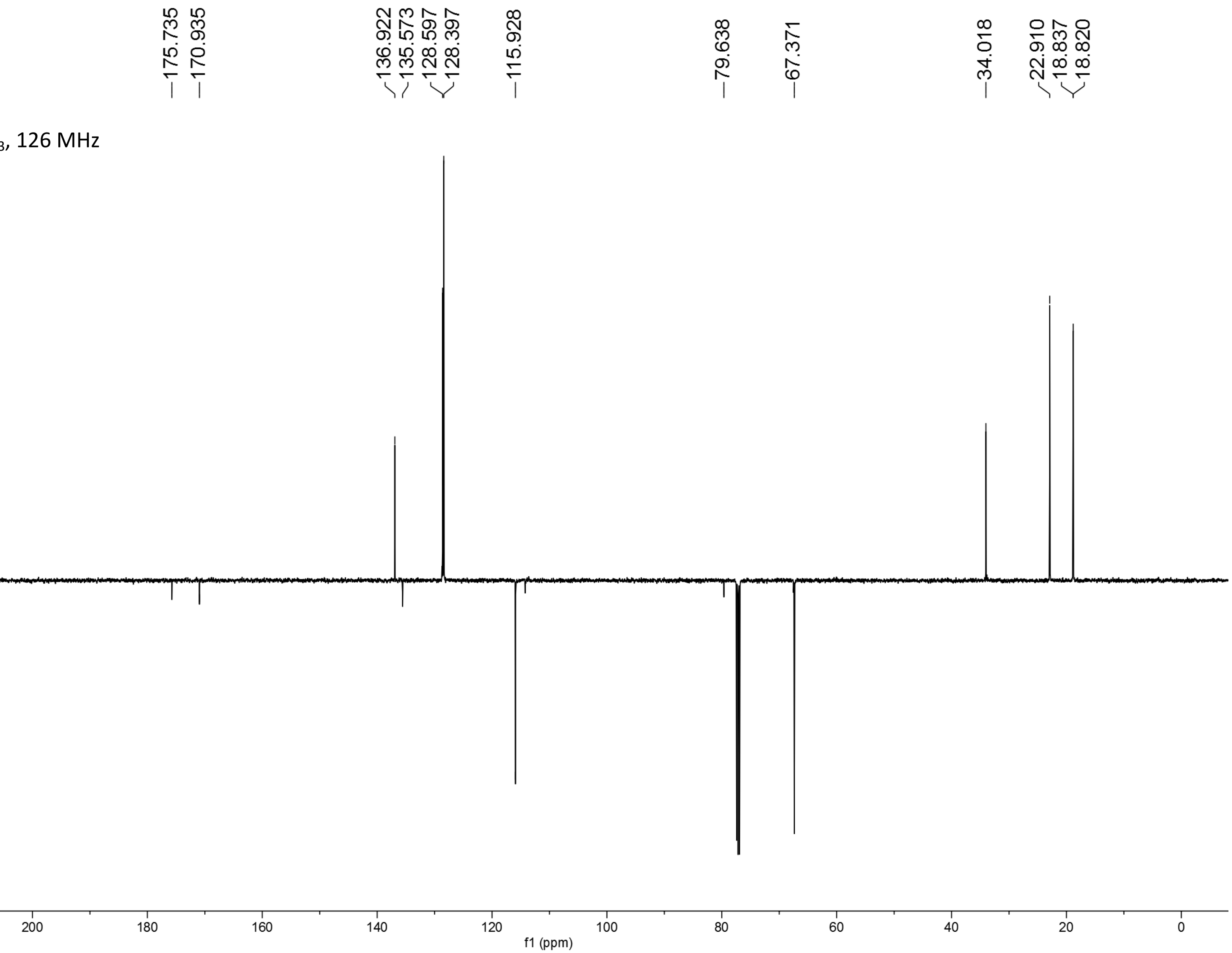

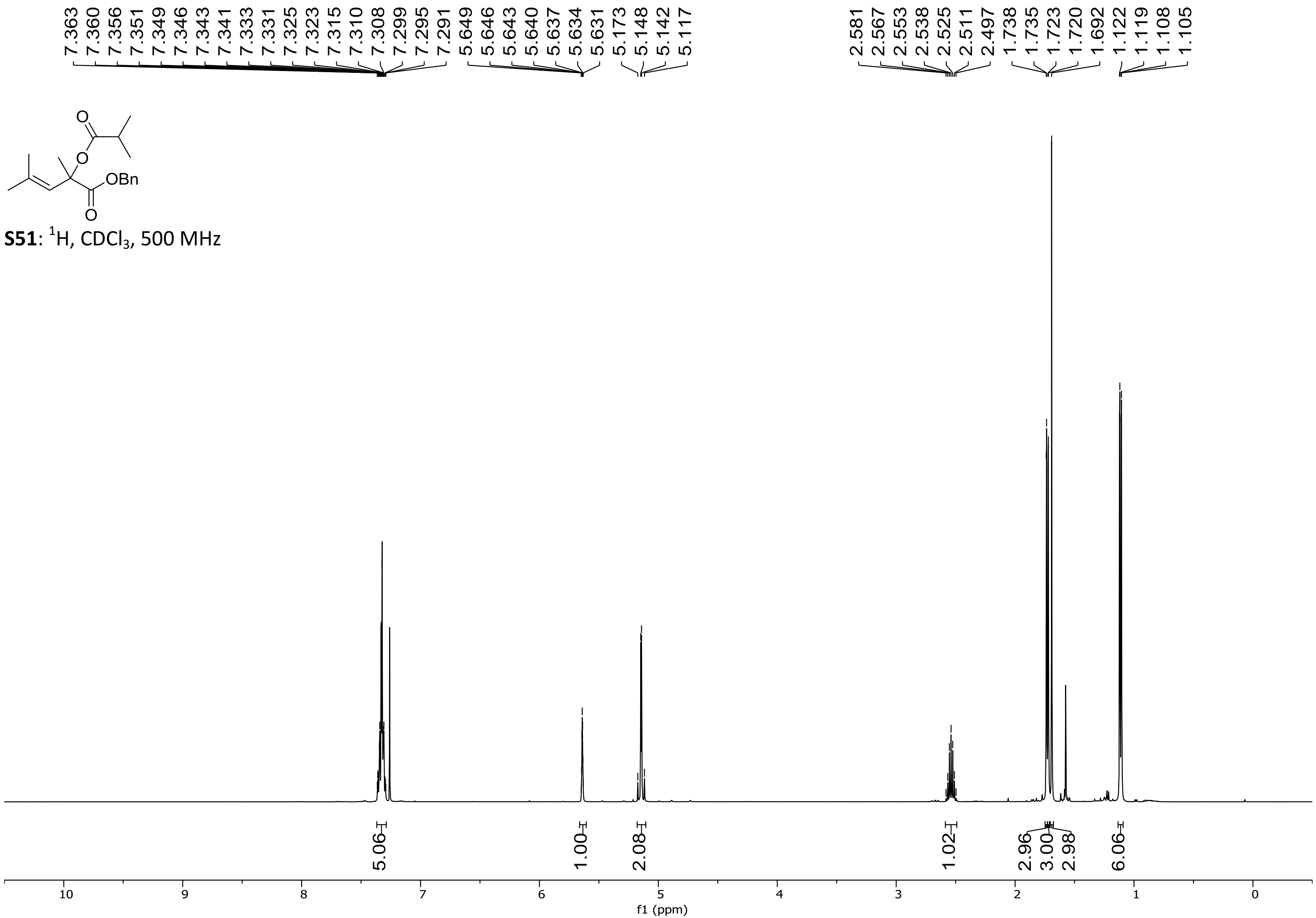
7.363 7.360 7.356 7.351 7.349 7.346 7.343 7.341 7.333 7.331 7.325 7.323 7.315 7.310 7.308 7.299 7.295 7.291 5.649 5.646 5.643 5.640 5.637 5.634 5.631 5.173 5.148 5.142 5.117
2.581 2.567 2.553 2.538 2.525 2.511 2.497 1.738 1.735 1.723 1.720 1.692 1.122 1.119 1.108 1.105
O
O
OBn
O
S51: ^{1}H, $CDCl_3$, 500 MHz
5.06 1.00 2.08 1.02 2.96 3.00 2.98 6.06
10 9 8 7 6 5 4 3 2 1 0
f1 (ppm)

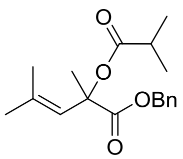

S51: ^{13}C, $CDCl_3$, 126 MHz

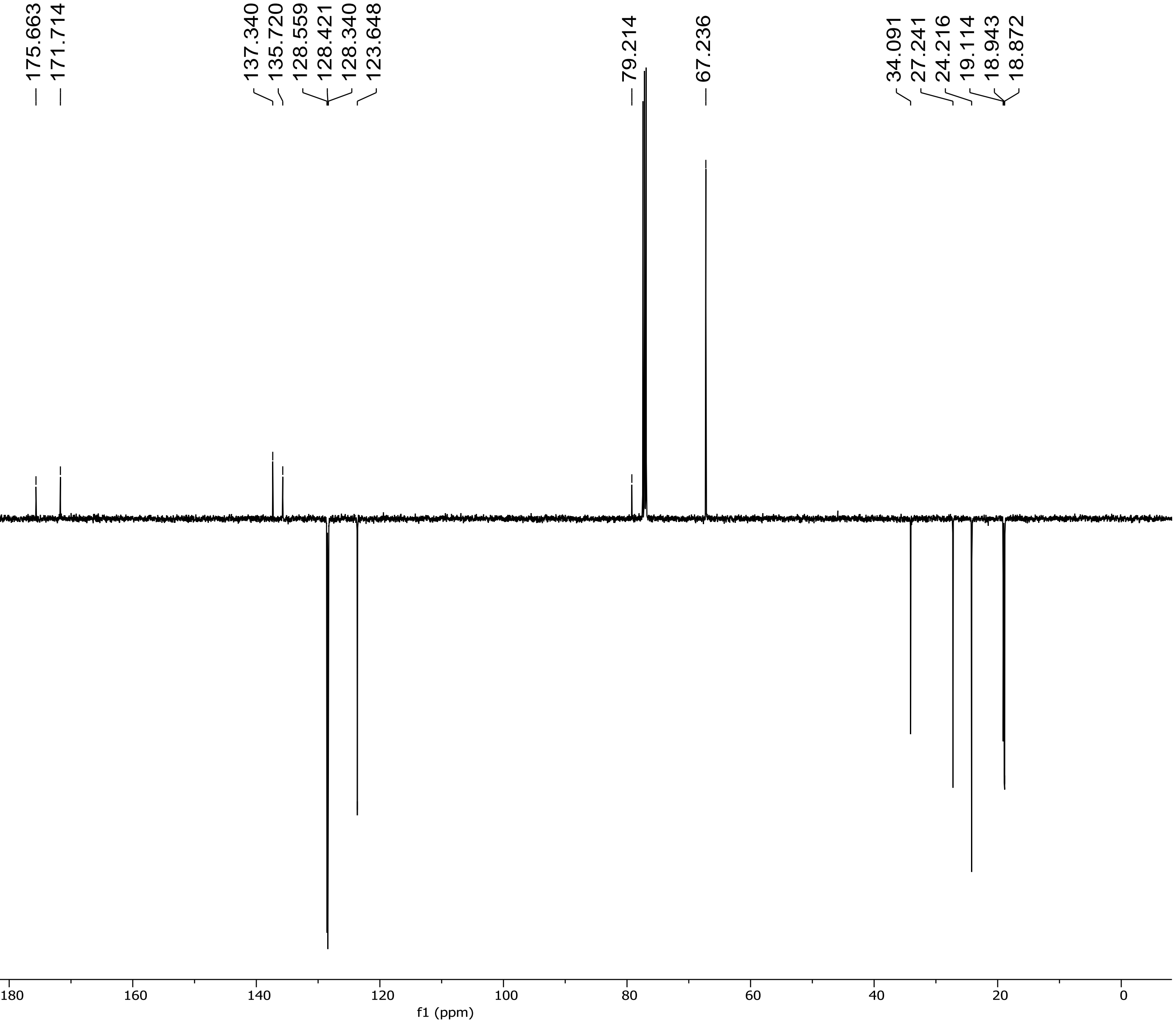

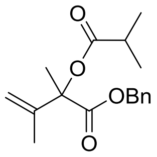

S52: ^{1}H, $CDCl_3$, 500 MHz

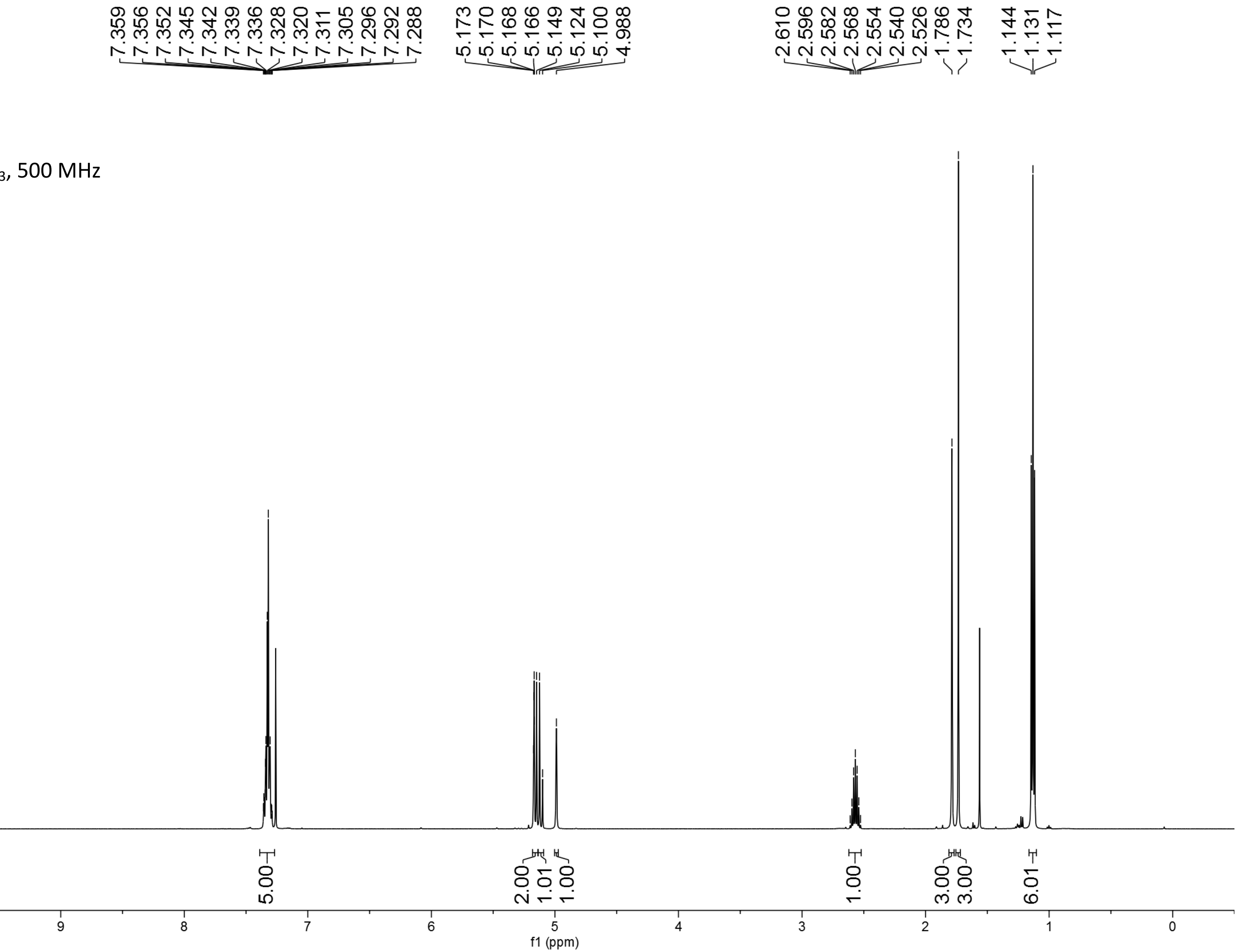

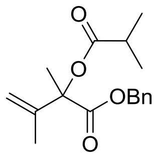

S52: ^{13}C, $CDCl_3$, 126 MHz

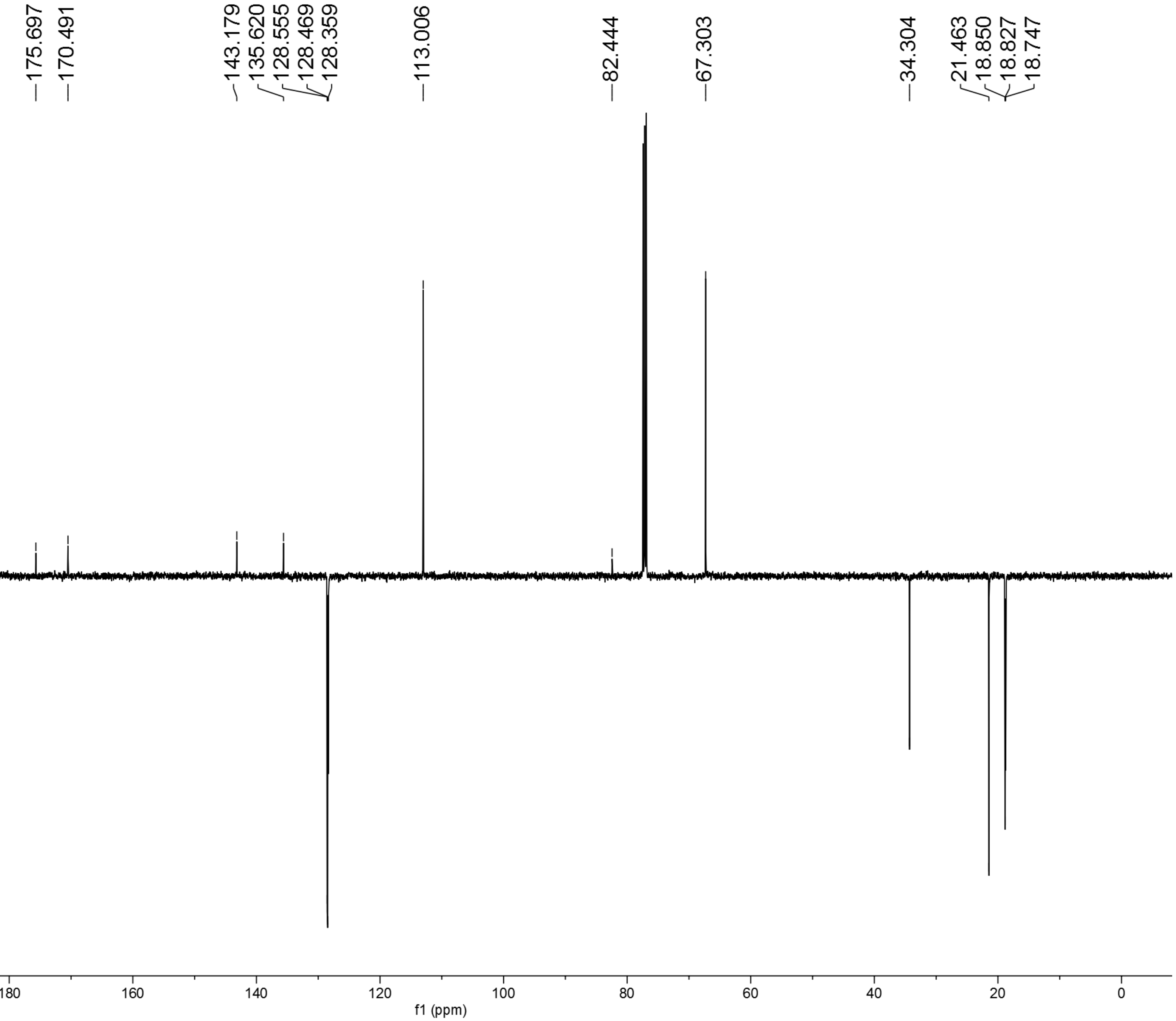

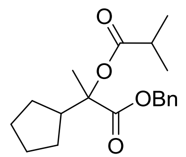

S53: ^{1}H, $CDCl_3$, 500 MHz

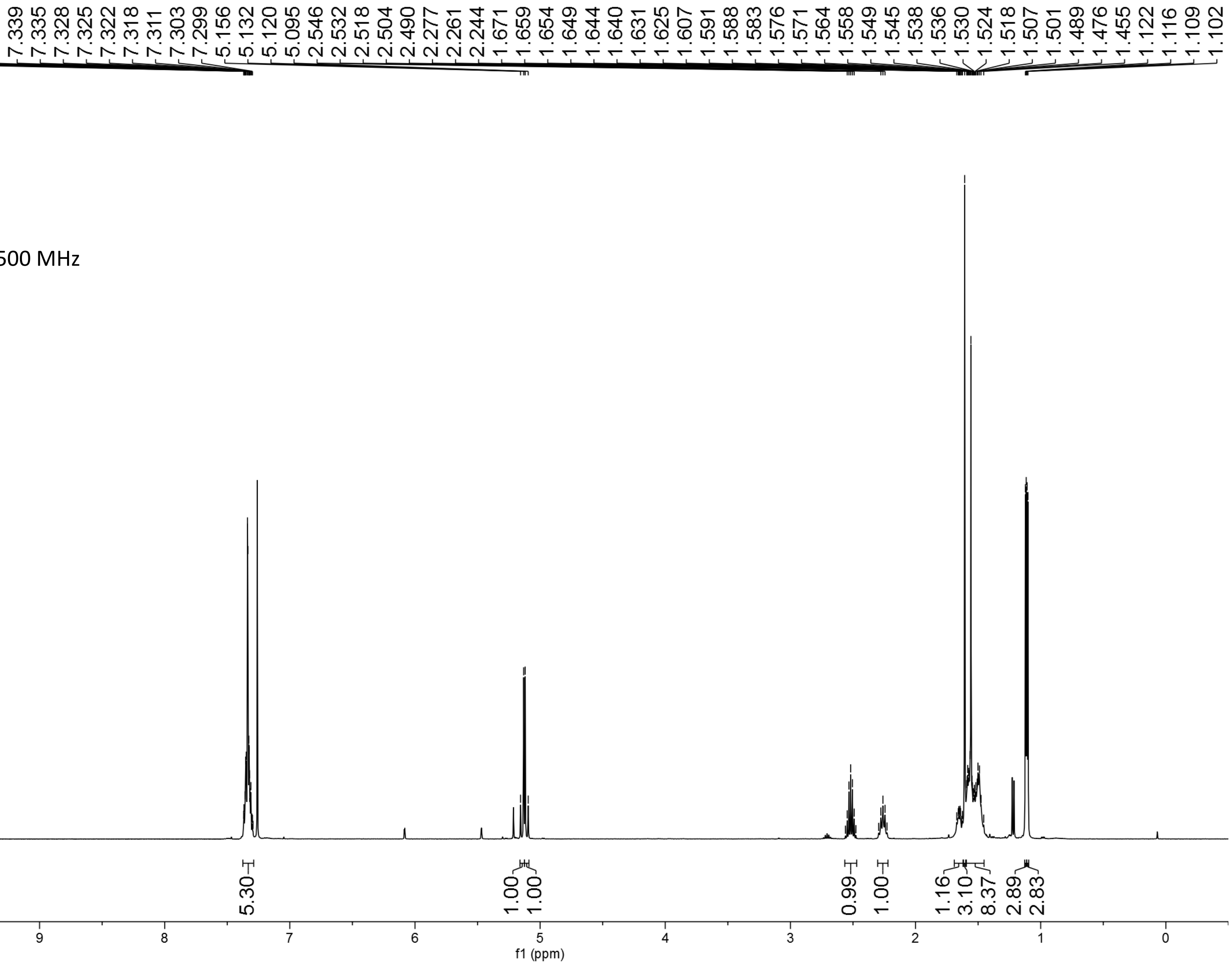

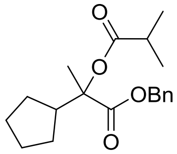

S53: ^{13}C, $CDCl_3$, 126 MHz

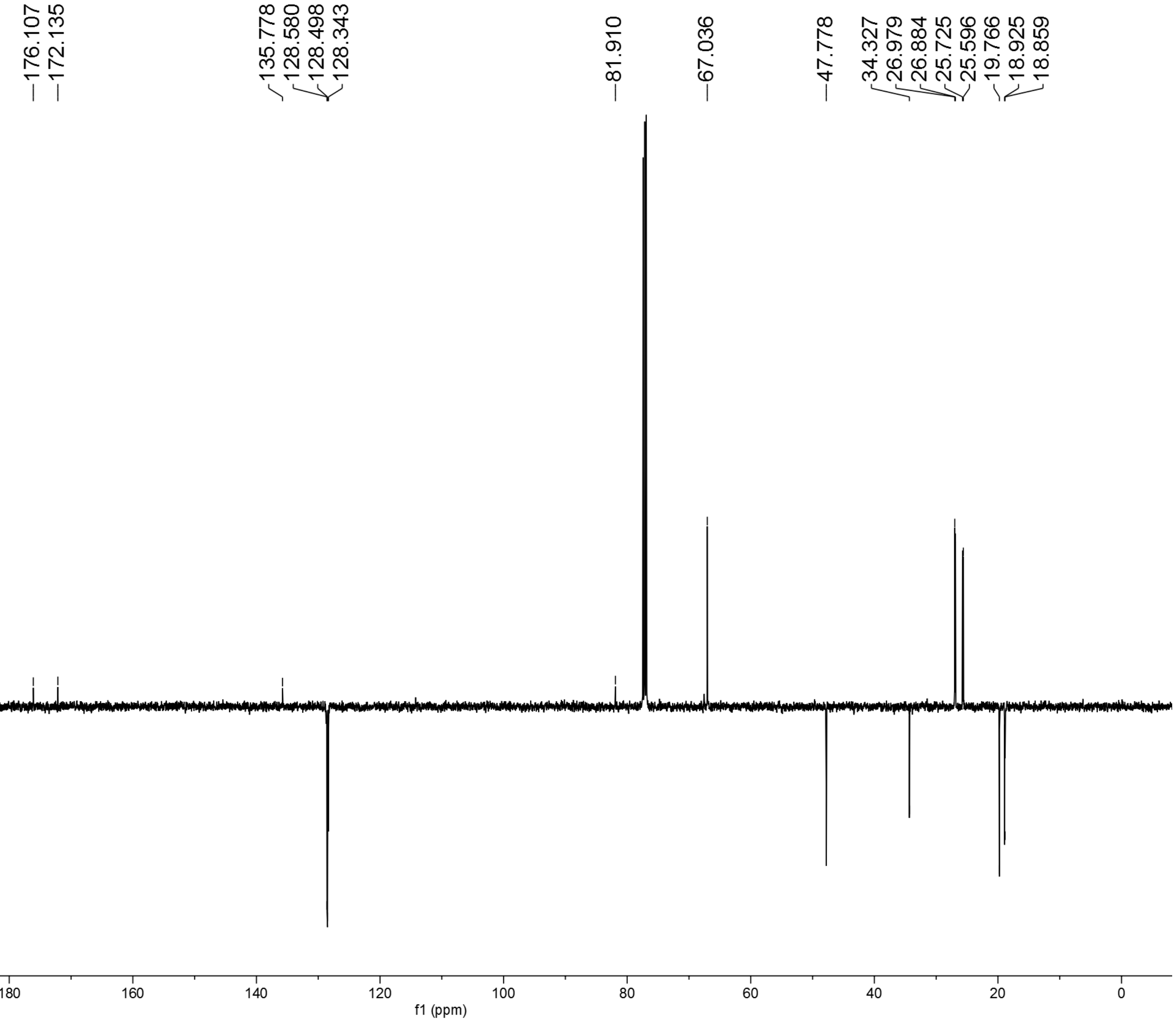

S54: ^{1}H, $CDCl_3$, 500 MHz

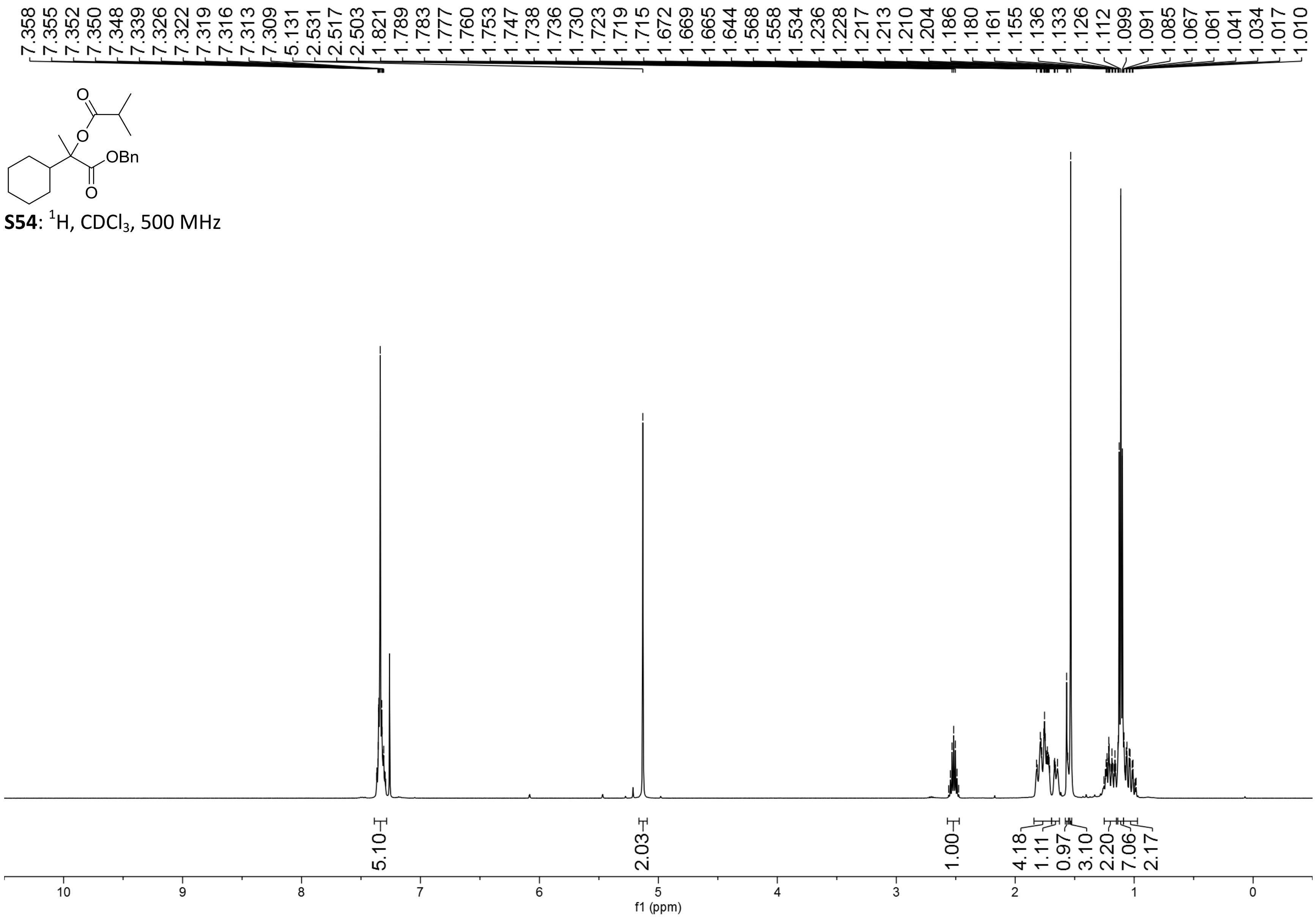

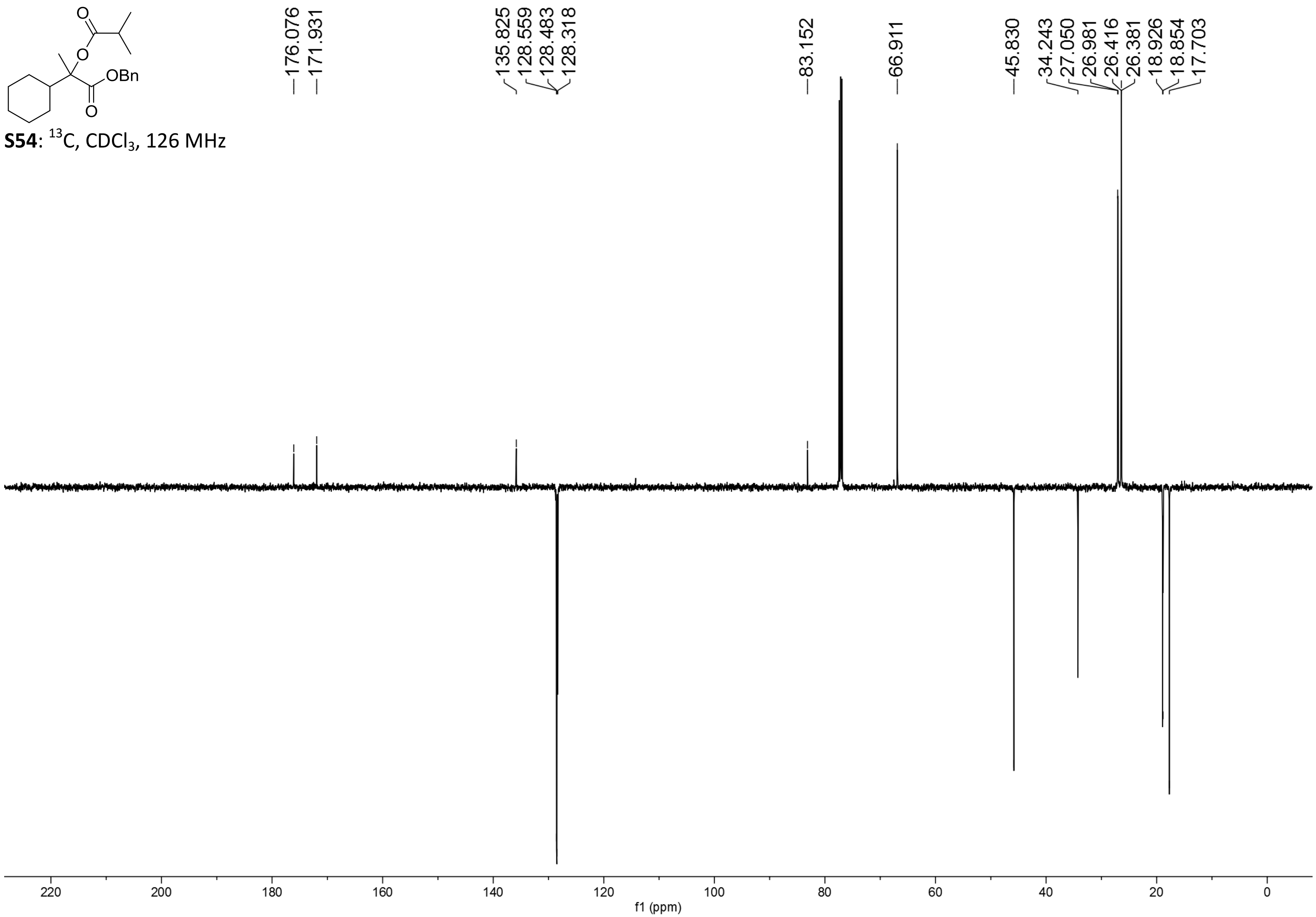

S54: ^{13}C, $CDCl_3$, 126 MHz

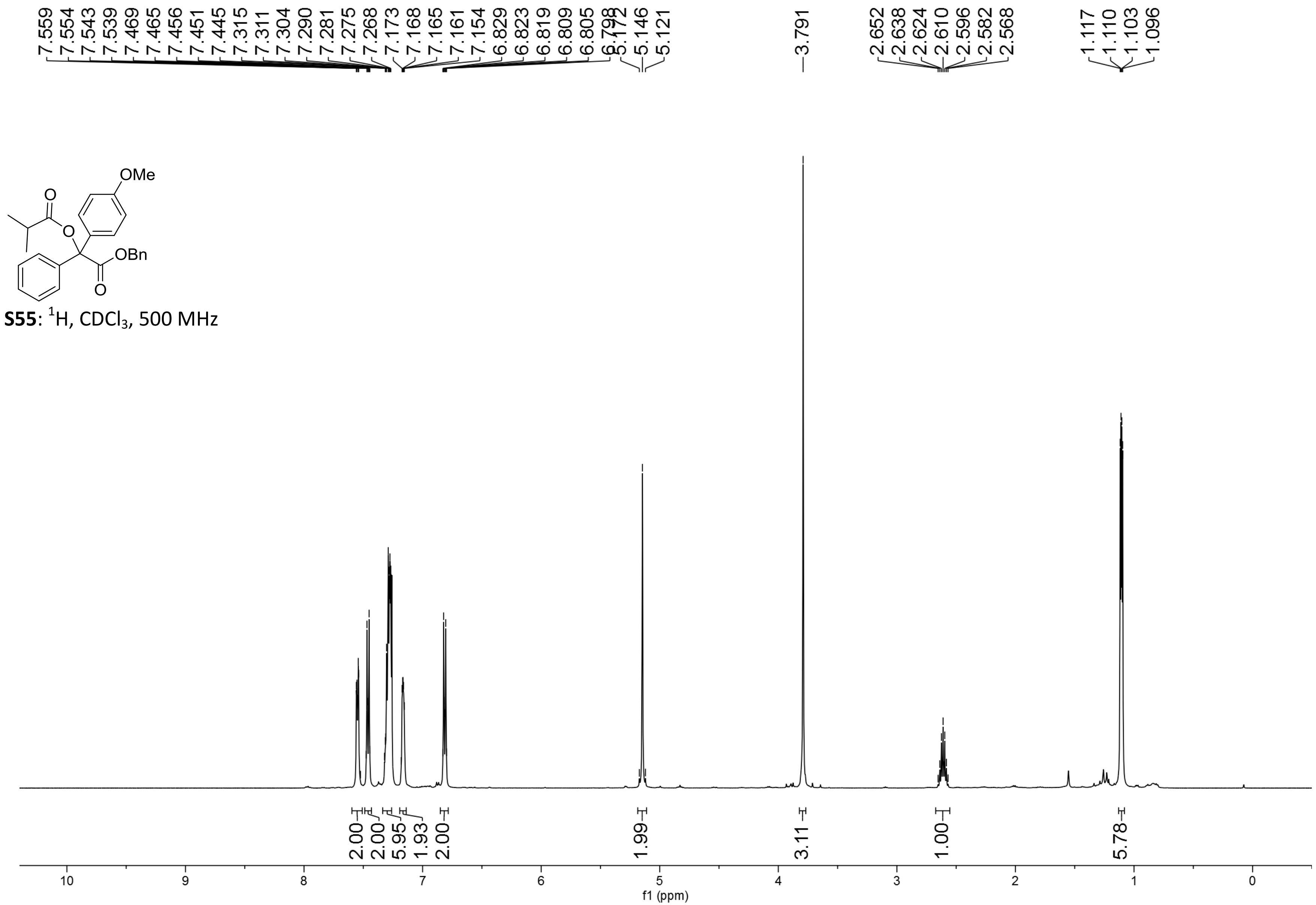

S55: [1]H, $CDCl_3$, 500 MHz

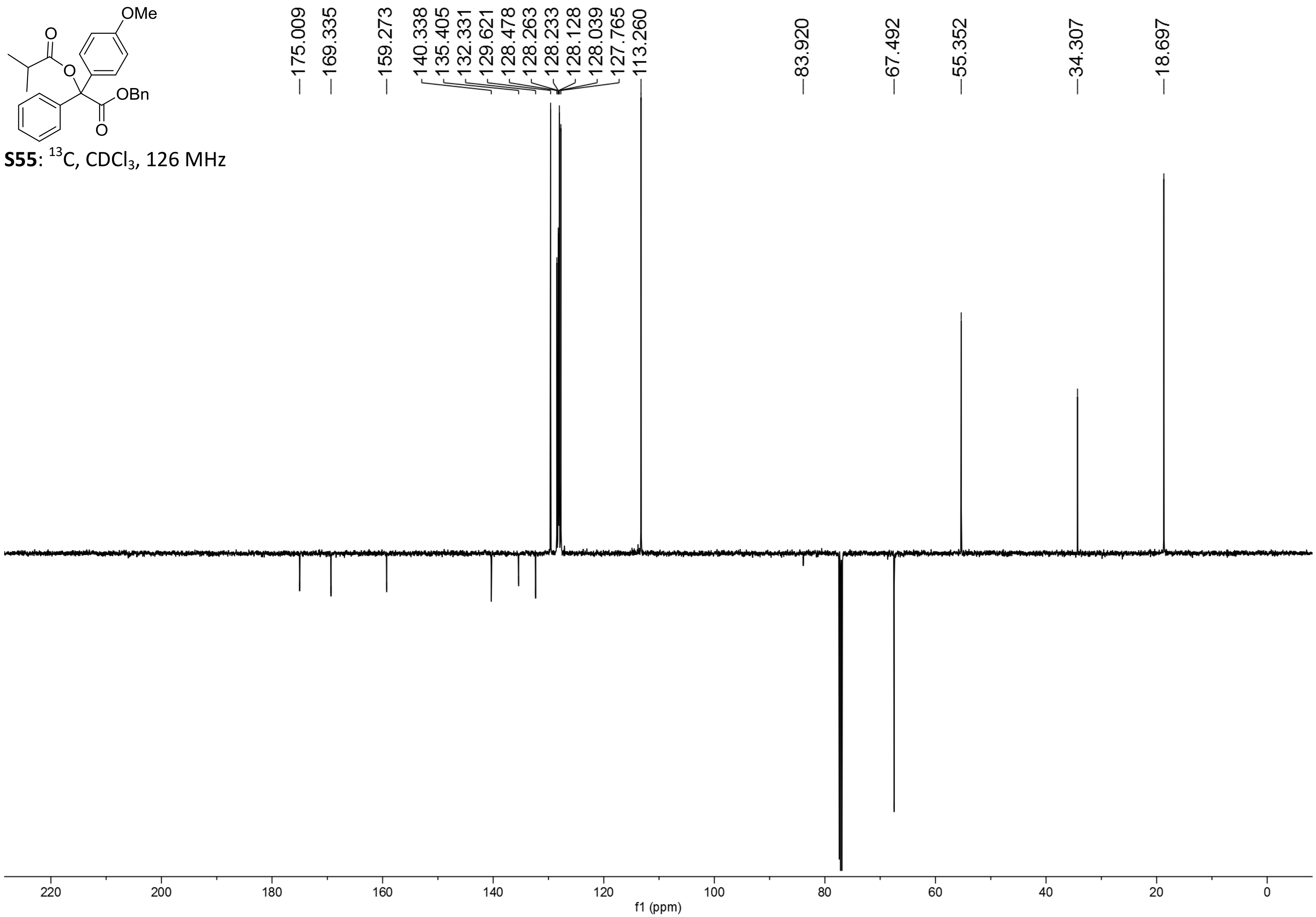

S55: ^{13}C, $CDCl_3$, 126 MHz

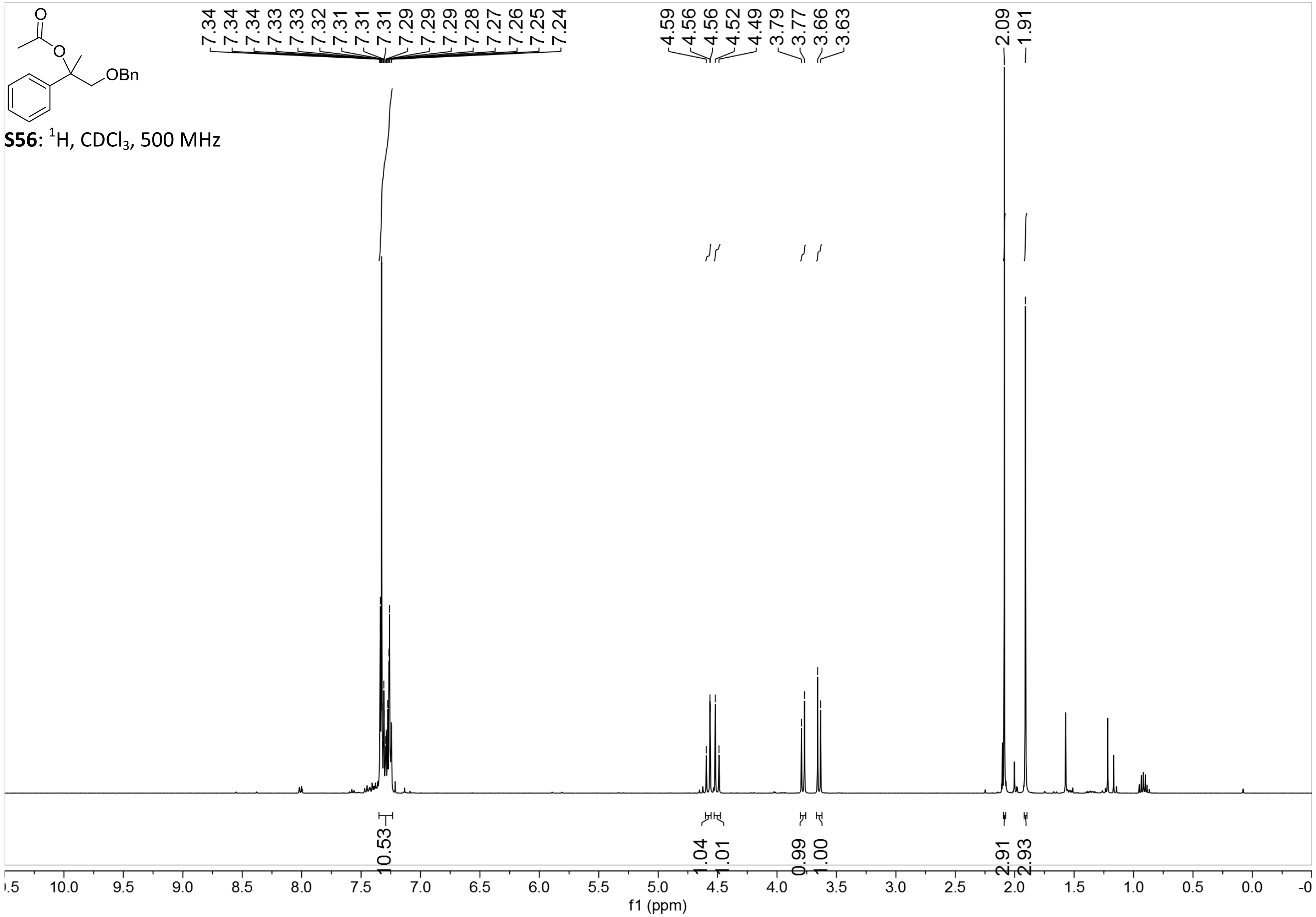
OBn
S56: ^{1}H, CDCl$_3$, 500 MHz
7.34 7.34 7.34 7.33 7.33 7.32 7.31 7.31 7.31 7.29 7.29 7.29 7.28 7.27 7.26 7.25 7.24
4.59 4.56 4.56 4.52 4.49 3.79 3.77 3.66 3.63
2.09 1.91
10.53 1.04 1.01 0.99 1.00 2.91 2.93
f1 (ppm)

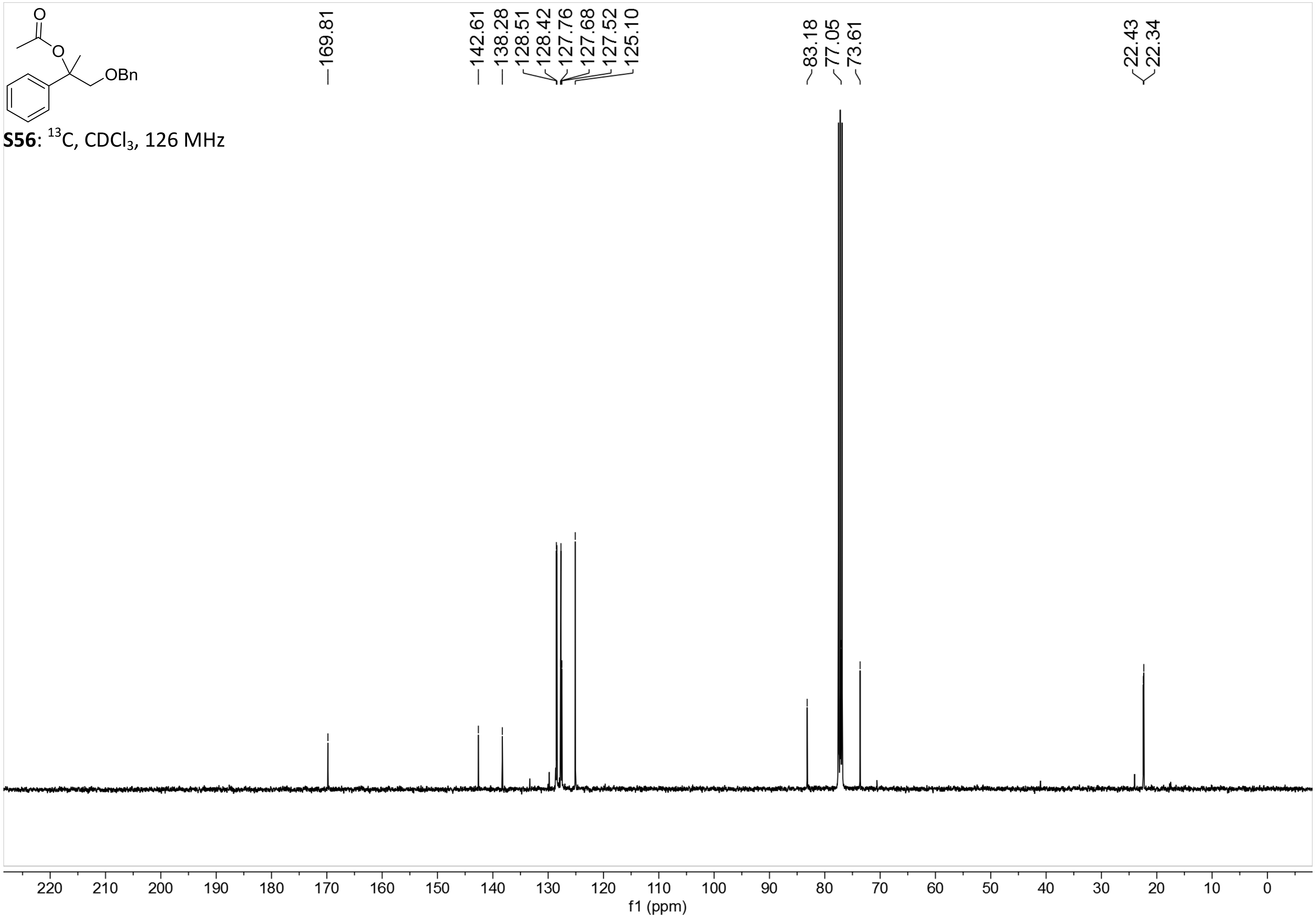

S56: ^{13}C, $CDCl_3$, 126 MHz

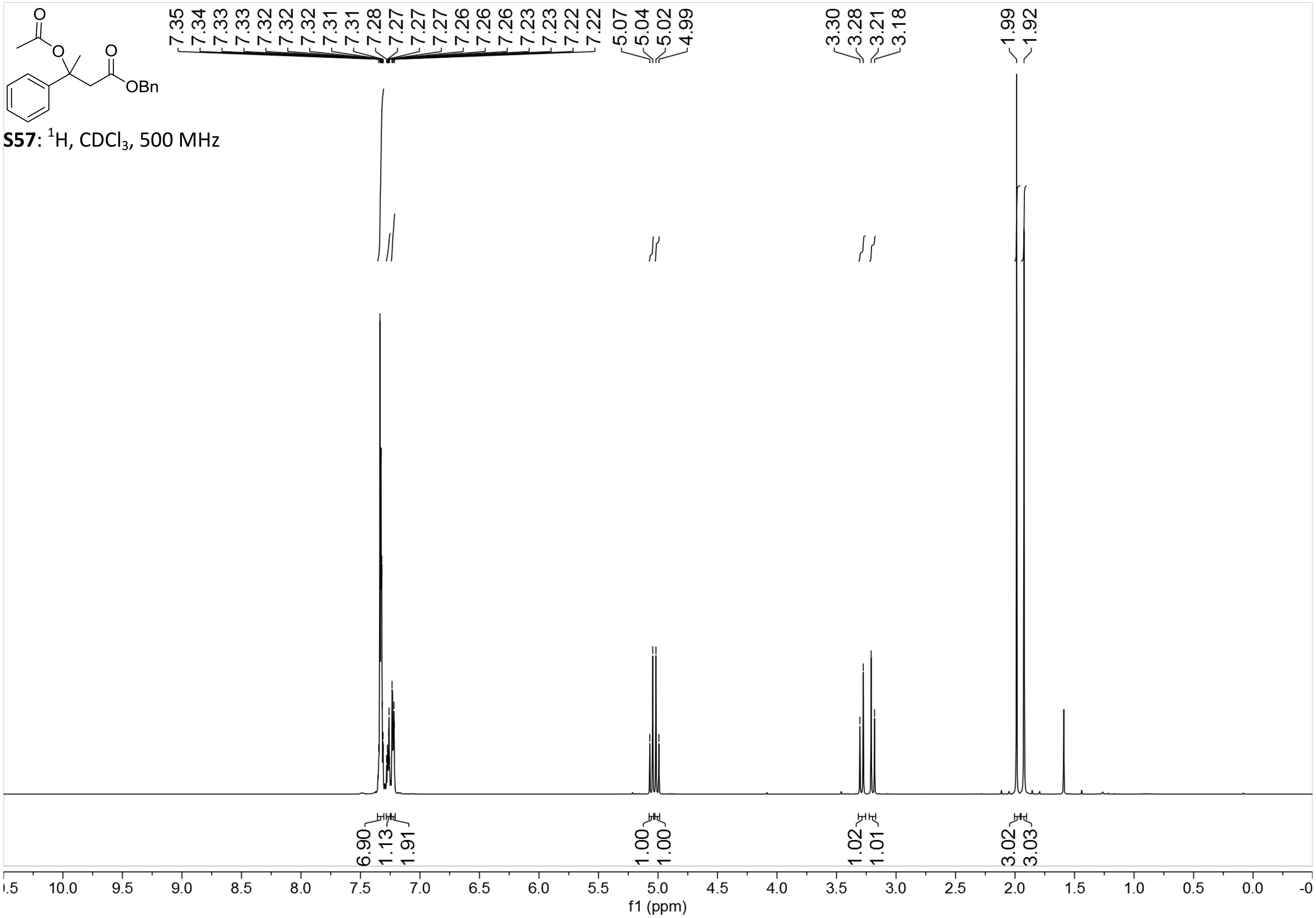
OBn
S57: 1H, CDCl3, 500 MHz
7.35
7.34
7.33
7.33
7.32
7.32
7.32
7.31
7.31
7.28
7.27
7.27
7.27
7.26
7.26
7.26
7.23
7.23
7.22
7.22
5.07
5.04
5.02
4.99
3.30
3.28
3.21
3.18
1.99
1.92
6.90
1.13
1.91
1.00
1.00
1.02
1.01
3.02
3.03
f1 (ppm)

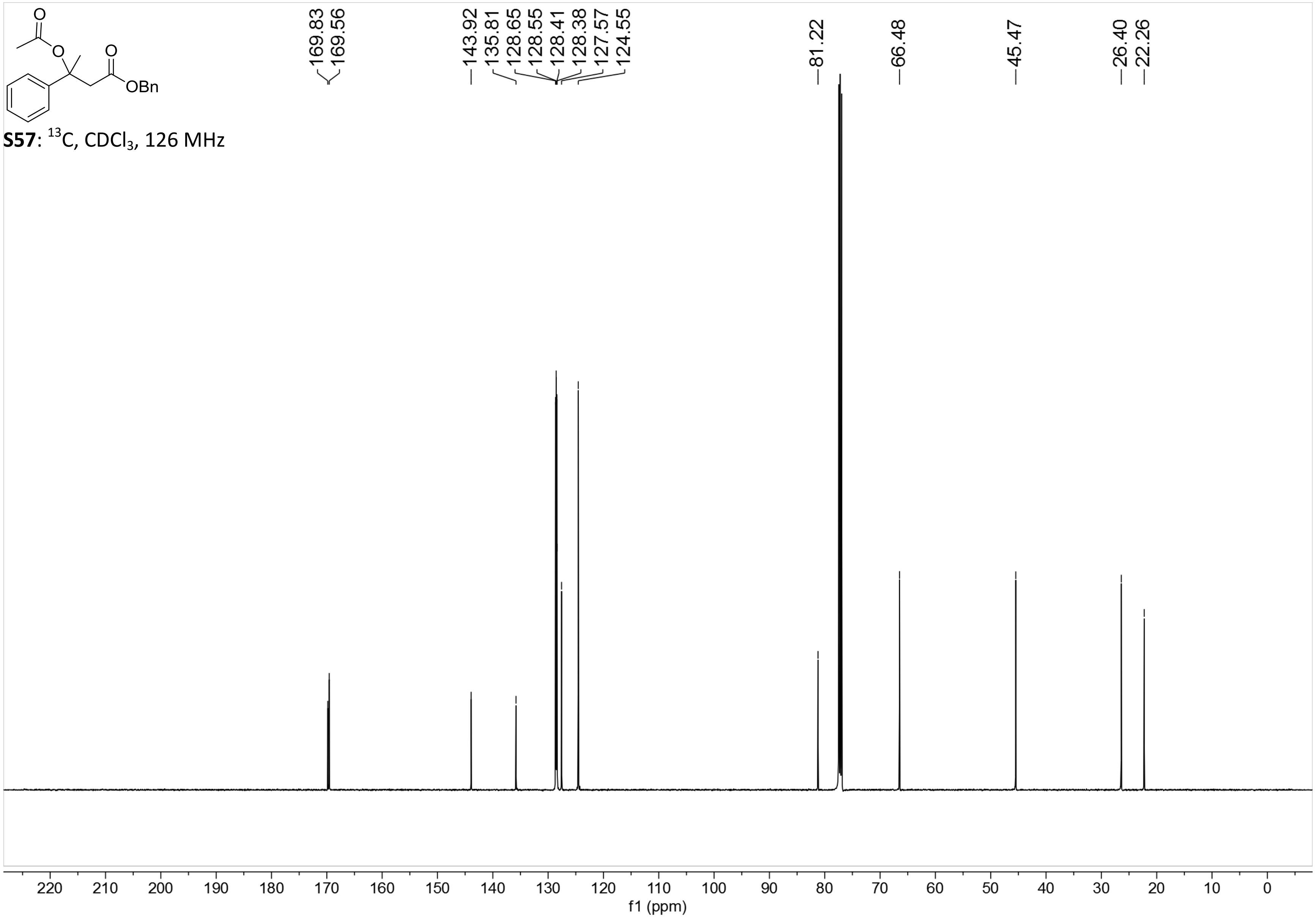

S57: ^{13}C, $CDCl_3$, 126 MHz

References

1. S. Chen, Q. Lou, Y. Ding, S. Zhang, W. Hu, J. Zhao, *Adv. Synth. Catal.* **2015**, *357*, 2437–2441.
2. Y-F. Liang, N. Jiao, *Angew. Chem. Int. Ed.* **2014**, *53*, 548–552.
3. J. Hu, T. Lan, Y. sun, H. Chen, J. Yao, Y. Rao, *Chem. Commun.,* **2015**, *51*, 14929–14932.
4. D. Ip, J. Rocek, *J. Am. Chem. Soc*. **1979**, *101*, 6311–6319.
5. A. Aramini, M. R. Sablone, G. Bianchini, A. Amore, M. Fanì, P. Perrone, A. Dolce, M. Allegretti, *Tetrahedron*, **2009**, *65*, 2015–2021.
6. X. Creary, H. N. Hatoum, A. Barton, T. E. Aldridge, *J. Org. Chem.* **1992**, *57*, 1887–1897.
7. K. Jia, Y. Pan, Y. Chen, *Angew. Chem. Int. Ed.* **2017**, *56*, 2478–2481.
8. G. Blay, I. Fernandez, P. Formentin, B. Monje, J. R. Pedro, R. Ruiz, *Tetrahedron* **2001**, *57*, 1075–1081.
9. Y. Ikeda, E. Manda, *Chem. Lett*. **1984**, 453–454.
10. J. Zhu, Y. Yuan, S. Wang, Z-J. Yao, *ACS Omega*, **2017**, *2*, 4665–4677.
11. L. C. Wieland, H. Deng, M. C. Snapper, A. H. Hoveyda, *J. Am. Chem. Soc.*, **2005**, *127*, 15453–15456.
12. N. Boechat, W. B. Kover, M. M. Bastos, N. C. Romeiro, A. S. C. Silva, F. C. Santos, A. L. Valverde, M. L. G. Azevedo, W. Wollinger, T. M. L. Souza, S. L. O. de Souza, I. C. P. P. de Frugulhetti, *Med. Chem. Res.* **2007**, *15*, 9, 492–510.
13. S. Mouné, G. Niel, M. Busquet, E. Eggleston, P. Jouin, *J. Org. Chem*., **1997**, *62*, 3332–3339.
14. N. Marukami, S. Tamura, W. Wang, T. Takagi, M. Kobayashi, *Tetrahedron*, **2001**, *57*, 4323–4336.
15. Y. Huang, R-Z Huang, Y. Zhao, *J. Am. Chem. Soc*. **2016**, *138*, 6571–6576.
16. F. A. Armstrong, E. L. Lipscomb, *J. Chem. Soc. Perkin. Trans. 1*, **1983**, 1197–1201.
17. Y. Hamashima, M. Kanai, M. Shibasaki, *J. Am. Chem. Soc.*, **2000**, *122*, 7412–7413.
18. H-F. Duan, J-H. Xie, X-C. Qiao, L-X. Wang, Q-L. Zhou, *Angew. Chem. Int. Ed.,* **2008**, *47*, 23, 4351–4353.
19. Z. Wang, Y-T. Cui, Z-B. Xu, J. Qu, *J. Org. Chem*. **2008**, *73*, 6, 2270–2274.
20. J. E. Green, D. M. Bender, S. Jackson, M. J. O'Donnell, J. R. McCarthy, *Org. Lett.,* **2009**, *11*, 4, 807–810.
21. M. Pawliczek, T. Hashimoto, K. Maruoka, *Chem. Sci.,* **2018**, *9*, 1231–1235.
22. A. Grajewska, M. Oestreich, *Synlett* **2010**, *16*, 2482–2484.
23. J. M. Concellón, C. Concellón, *J. Org. Chem.,* **2006**, *71*, 4428–4432.
24. J. E. Green, D. M. Bender, S. Jackson, M. J. O'Donnell, J. R. McCarthy, *Org. Lett.,* **2009**, *11*, 4, 807–810.
25. L. Huke, M. Berton, A. de la Hoz, A. Díaz-Oritz, J. Alcázar, *Green Chem.* **2017**, *19*, 1420–1424.
26. L. C. Wieland, H. Deng, M. C. Snapper, A. H. Hoveyda, *J. Am. Chem. Soc.*, **2005**, *127*, 15453–15456.
27. G. Blay, I. Fernández, A. Marco-Aleixandre, J. R. Petro, *Org. Lett*., **2006**, *8*, 7, 1287–1290.
28. K. Fuji, K. Tanaka, M. Ahn, M. Mizuchi, *Chem. Pharm. Bull.* **1994**, *42*, 957–959.
29. T. Ueda, K. Tanaka, T. Ichibakase, Y. Orito, M. Nakajima, *Tetrahedron*, **2010**, *66*, 7726–7731.
